# Undirected ruthenium-catalyzed C–H activation using arylsulfonium salts: direct arylation without ruthenacycle intermediates revealed by computation and data science

**DOI:** 10.1039/d5sc08962j

**Published:** 2026-01-23

**Authors:** Jinbin Zhu, Binbin Yuan, Xuexue Chang, Hasret Can Gülen, Lutz Ackermann

**Affiliations:** a Institut für Organische und Biomolekulare Chemie, Georg-August-Universität Göttingen Tammannstraße 2 Göttingen 37077 Germany lutz.ackermann@chemie.uni-goettingen.de; b Wöhler Research Institute for Sustainable Chemistry (WISCh), Georg-August-Universität Göttingen Tammannstraße 2 Göttingen 37077 Germany; c Jiangxi Provincial Key Laboratory of Synthetic Pharmaceutical Chemistry, Gannan Normal Univeristy Ganzhou 341000 China

## Abstract

Ruthenium-catalyzed C–H activation has surfaced as a transformative platform in molecular sciences. Despite major progress, all ruthenium-catalyzed arylations require the formation of a ruthenacycle. In sharp contrast, we herein report on ruthenium-catalyzed C–H arylation with arylsulfonium salts through a non-cycloruthenated intermediate, which allowed the late-stage incorporation of polyfluoroarenes into natural products and pharmaceuticals in the absence of directing groups. Employing a *t*Bu-substituted dibenzothiophenium salt, we realized polyfluoroarylation for a wide range of functionalized arenes. Detailed experimental and computational studies provided strong support for the C–H arylation to proceed without any metalacyclic intermediate. A data science approach further elucidated the key molecular features governing the reactivity of arylsulfonium salts.

## Abstract 2

Ruthenium-catalyzed C–H activation has surfaced as a transformative platform in molecular sciences. Despite major progress, all ruthenium-catalyzed arylations require the formation of a ruthenacycle.

## Graphical abstract

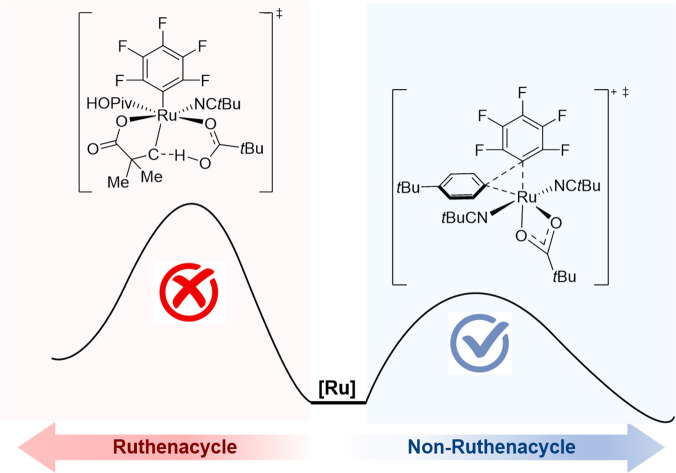

## Introduction

**Scheme 1:**
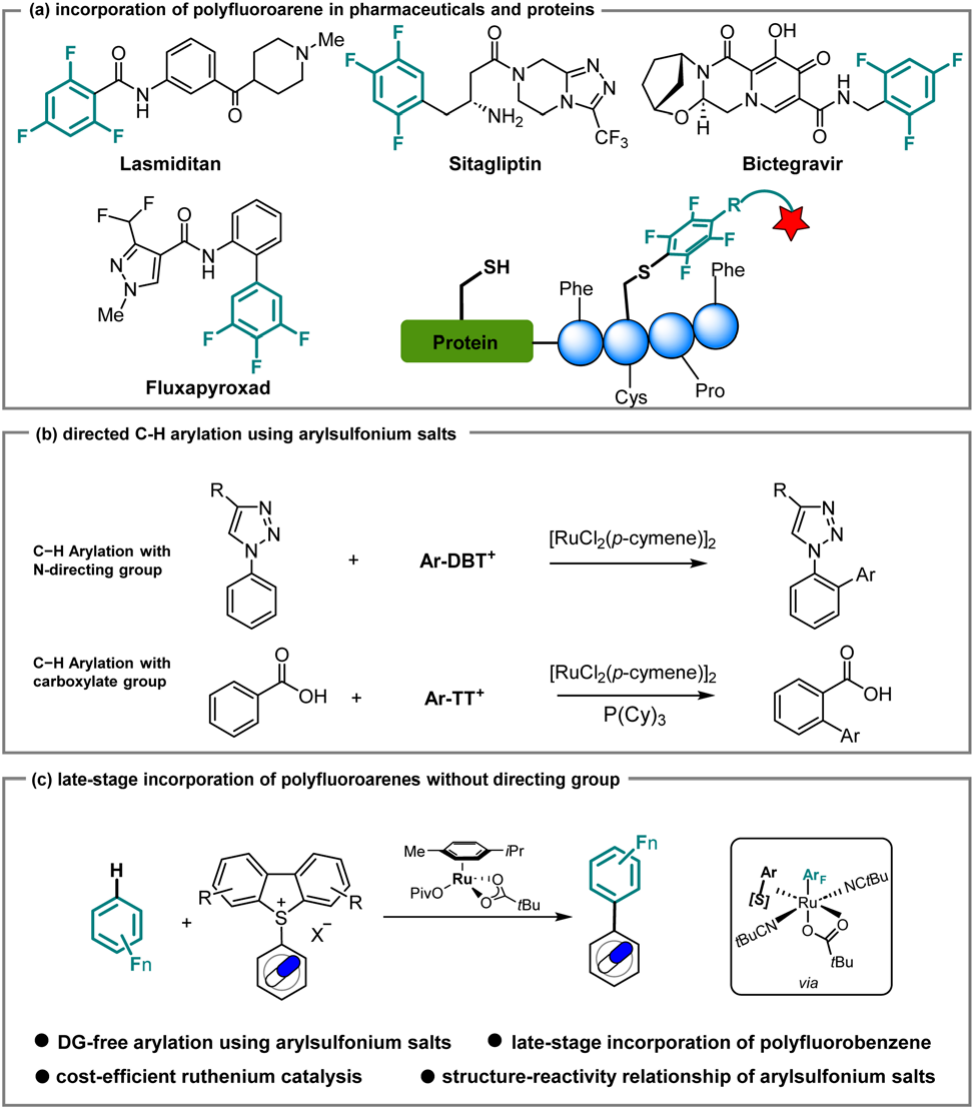
Significance of polyfluoroarene-containing molecules and utilization of arylsulfonium salts for late-stage functionalization.

Fluorine incorporation, which can increase metabolic stability, improve bioavailability, and enhance the binding affinity of lead compounds, is a significant strategy for obtaining superior drugs in pharmaceutical research.^1,2^ Currently, approximately 20% of commercial drugs contain fluorine, among which polyfluoroarene-containing molecules represent key structural features, as found in, for instance, Lasmiditan and Sitagliptin for the treatment of migraine^3^ and Type 2 diabetes,^4^ respectively (Scheme 1a). Taking advantage of the fluorine effect, polyfluoroarenes were also installed on agrochemicals such as Fluxapyroxad.^5^ On a different note, polyfluoroarenes can react efficiently with cysteine *via* S_N_Ar, which provides a general strategy for diverse modifications of peptides and proteins.^6,7^ Specifically, employing a variety of polyfluoroarenes, the perfluoroaryl–thiol reaction can enhance the biological properties of peptides, such as the stability against proteases and binding affinity to their target.^7^ Therefore, the incorporation of polyfluoroarenes into bioactive small molecules or drugs in latestage is highly desirable, with significant potential for applications in both medicinal chemistry and chemical biology.

Although transition metal-catalyzed cross-coupling reactions are traditionally employed to forge biaryls,^8^ in the last two decades, catalytic direct C–H arylation has emerged as a more sustainable and environmentally friendly approach.^9–14^

In this context, catalytic C–H arylation of polyfluoroarenes was realized with aryl halides^15–20^ and other arylating agents.^21–24^ However, methods that allow incorporation of polyfluoroarenes into complex molecules at the late stage remain scarce.

Arylsulfonium salts have experienced rapid development over the last few years.^25,26^ In comparison with aryl halides, the preparation of arylsulfonium salts features excellent functional group tolerance and high site-selectivity, rendering them powerful linchpins for further functionalization of complex arenes. Notable contributions from Ritter^27–33^ and Procter,^34–38^ among others,^39–42^ showcased a series of significant transformations for complex molecules, such as drugs and natural products, *via* cross-coupling and photochemical transformations. Alternatively, the use of arylsulfonium salts in C–H activation would be extremely advantageous, namely in late-stage functionalization, avoiding the need for the synthesis of nucleophilic reaction partners. In the last two decades, catalytic C–H arylation, particularly under ruthenium catalysis,^43–47^ has emerged as a uniquely powerful platform for *ortho*-, *meta*-, and remote diversifications as well as an efficient strategy to access biaryls.^48–66^ More recently, Ackermann employed arylsulfonium salts to realize the late-stage incorporation of triazoles and tetrazoles into drug molecules (Scheme 1b, top).^67^ Later, Zhang, Gooβen and Chen reported on carboxylate-directed C–H arylation (Scheme 1b, bottom).^68^ These advances lead us to wonder whether undirected ruthenium-catalyzed C–H arylation would be a viable strategy in a late-stage functionalization scenario in the presence of arylsulfonium salts. As part of our program on ruthenium-catalyzed C–H activation, we report herein on a directing group-free C–H arylation of polyfluoroarenes through the use of arylsulfonium salts. Salient features of our findings include detailed experimental, computational, and data science studies that provide key insights into C–H arylation without metallacycle formation.

## Results

### Reaction optimization

We commenced our studies on the desired C–H arylation of pentafluorobenzene (1a) by probing a series of arylsulfonium salts. [Ru(OPiv)_2_(*p*-cymene)]^45,51^ was chosen as the catalyst in the presence of potassium carbonate (K_2_CO_3_) as base and pivalonitrile (*t*BuCN) as the solvent. Investigation of arylsulfonium salts showed that the dibenzothiophenium salt (S4) delivered the product in superior yields (Table 1, entries 1–4). Encouragingly, a series of dibenzothiophenium salts (S5–S9) with different electronic properties and various substitution patterns were synthesized and tested, which revealed that 3,7-*tert*-butyl substituted dibenzothiophenium salt (S9) exhibited the highest reactivity, forming 3aa in 58% yield (Table 1, entry 9). Importantly, varying the additive disclosed that pivalic acid effectively facilitated the C–H arylation, contrary to 4-F-C_6_H_4_COOH, for which no improved yield was observed. Similarly, the presence of phenylphosphonic acid under otherwise identical reaction conditions proved unfruitful in increasing the reaction efficacy. It is worth noting that a silver salt and phosphine ligand were detrimental to the desired transformation (Table 1, entries 10–14). The increase of the reaction temperature led to an improved reaction outcome, providing the arylated pentafluoroarene in 82% yield (entry 15). Increasing the amount of 1a to 10 equivalents resulted in a 92% yield after 24 hours (entry 16). Other ruthenium catalysts, such as those bearing acetate, mesitylcarboxylate, or adamantane-1-carboxylate, as well as reported catalysts for a cycloruthenation-enabled oxidative addition, proved to be inefficient for the undirected ruthenium-catalyzed arylation (entries 17–19 and SI).

**Table 1:** Optimization of C–H arylation of pentafluorobenzene 1a

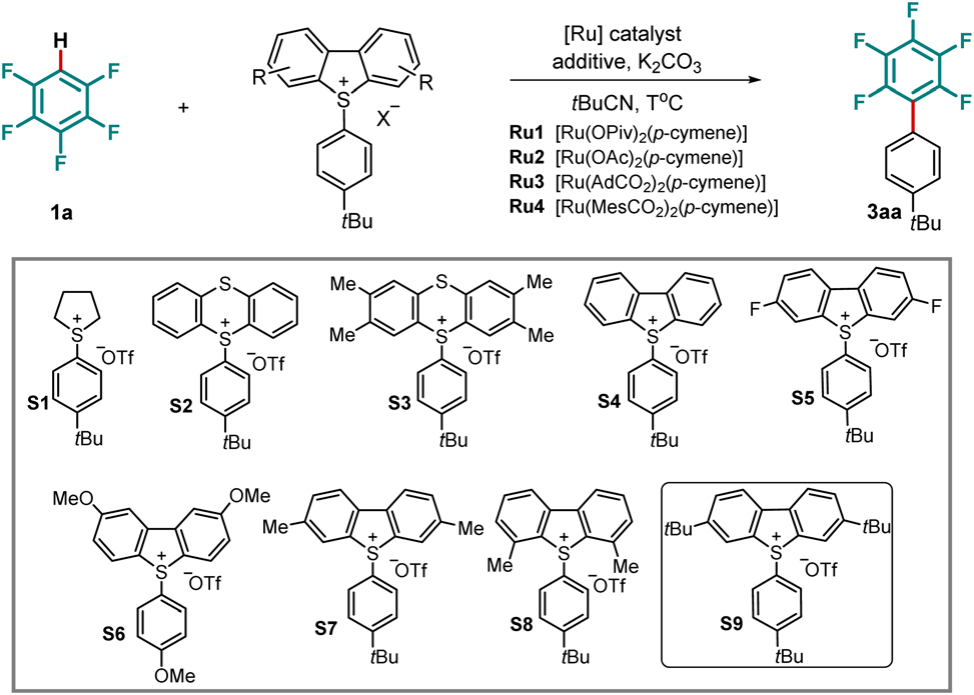						
Entry^a^	Sulfonium salt	[Ru]	Additive	T(°C)	Time (h)	Yield (%)
1	S1	Ru1	—	110	20	0
2	S2	Ru1	—	110	20	11
3	S3	Ru1	—	110	20	18
4	S4	Ru1	—	110	20	41
5	S5	Ru1	—	110	20	24
6	S6	Ru1	—	110	20	14
7	S7	Ru1	—	110	20	44
8	S8	Ru1	—	110	20	14
9	S9	Ru1	—	110	20	58
10	S9	Ru1	4-FC_6_H_4_COOH	110	20	58
11	S9	Ru1	PivOH	110	20	64
12	S9	Ru1	AgOPiv	110	20	Trace
13	S9	Ru1	PhPO(OH)_2_	110	20	55
14	S9	Ru1	(4CF_3_–C_6_H_4_)_3_P	110	20	0
15	S9	Ru1	PivOH	120	20	82
**16^b^**	**S9**	**Ru1**	**PivOH**	**120**	**24**	**92 (85)**
17	S9	Ru2	PivOH	120	20	62
18	S9	Ru3	PivOH	120	20	68
19	S9	Ru4	PivOH	120	20	57

a Unless otherwise specified, the reactions were performed under nitrogen with arylsulfonium salt (0.10 mmol, 1.0 equiv.), pentafluorobenzene 1a (0.50 mmol, 5.0 equiv.), Ru catalyst (10 mol%), additive (30 mol%), base (0.23 mmol, 2.3 equiv.), and *t*BuCN (55 µL).

b With pentafluorobenzene (1.0 mmol, 10.0 equiv.) and *t*BuCN (110 µL), the yield of the isolated product is given in parentheses.

### Investigation of substrate scope

**Scheme 2:**
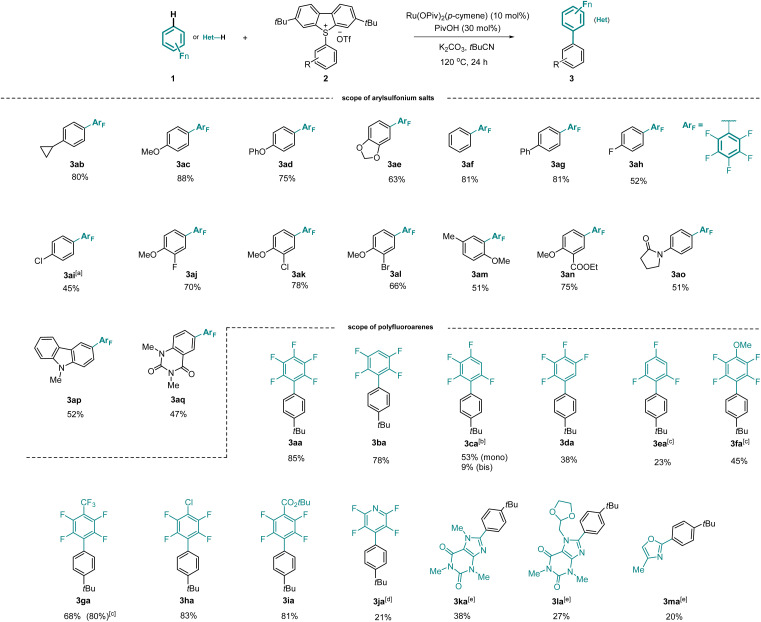
Investigation of substrate scope. Unless otherwise specified, reaction conditions: arylsulfonium salt 2 (0.2 mmol, 1 equiv.), polyfluoroarenes 1 (2.0 mmol, 10.0 equiv.), [Ru(OPiv)_2_(*p*-cymene)] (10 mol%), PivOH (30 mol%), K_2_CO_3_ (0.46 mmol, 2.3 equiv.), *t*BuCN (220 µL), under N_2_ at 120 °C for 24 h; isolated yields were reported. [a] 4-Chlorophenyl dibenzothiophenium salt was used. [b] 48 h. [c] 130 °C. [d] 130 °C, 42 h. [e] Heteroarenes 1 (1.0 mmol, 5 equiv.), *t*BuCN (300 µL).

With the optimized reaction conditions in hand, we investigated the substrate scope of the arylation employing various arylsulfonium salts. As depicted in Scheme 2, a series of sulfonium salts bearing electron-rich or neutral groups such as cyclopropyl-, methoxy-, phenoxy-, hydro-, and phenyl- in the C4 position of the aryl ring were fully tolerated, providing corresponding products 3ab–3ag in good yields. Importantly, halogens (F, Cl, Br) located in different positions of the aryl ring were all well tolerated, forming 3ah–3al in moderate to good yields. In contrast, *ortho*-substituted arylsulfonium salt 2m was less reactive. Notably, synthetically useful ester and amide groups proved to be compatible (3an, 3ao). Additionally, arylsulfonium salts were also selectively converted into the desired products 3ap and 3aq. It is worth mentioning that the *tert*-butyl substituted dibenzothiophene as a leaving group in the transformation could be recovered.

We further studied the generality of our strategy regarding the polyfluoroarenes. 1,2,4,5-Tetrafluorobenzene was efficiently arylated to deliver 3ba in 78% yield. Upon extending the reaction time, 1,3,4,5-tetrafluorobenzene afforded 3*ca* in 53% yield along with 9% of the bisarylated product. The arylation of 1,2,3,4-tetrafluorobenzene was revealed to be relatively sluggish, forming 3da in 38% yield. Less electron-deficient arenes, such as 1,3,5-trifluorobenzene and 2,3,5,6-tetrafluoroanisole, required higher reaction temperature to accomplish the desired arylation, forming 3ea and 3fa. Polyfluoroarenes carrying trifluoromethyl, chlorine, and ester functional groups were tolerated, furnishing the corresponding products in excellent yields (3ga–3ia). In contrast, tetrafluoropyridine was less reactive (3ja). Interestingly, heteroarenes such as caffeine, doxofylline and 4-methyloxazole were successfully arylated (3ka, 3la, 3ma).

**Scheme 3:**
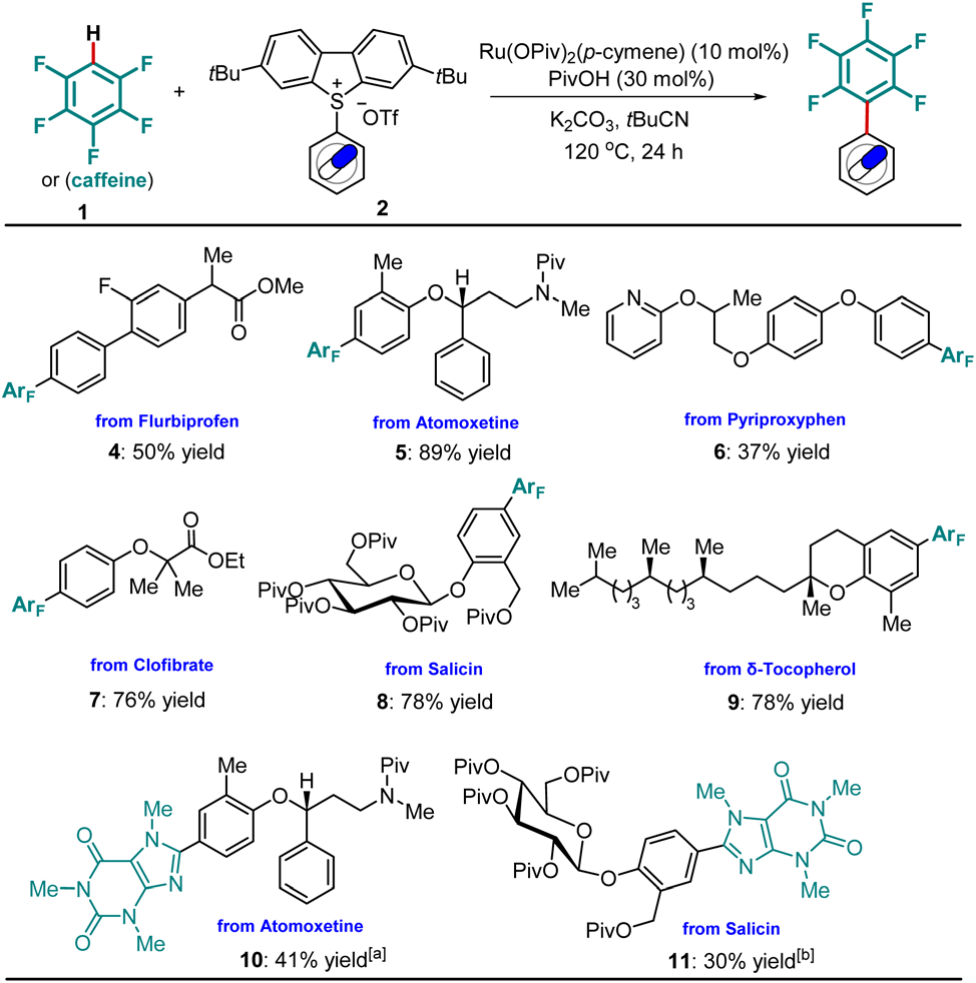
Late-stage incorporation of pentafluorobenzene or caffeine into drugs and natural products. Unless otherwise specified, reaction conditions: arylsulfonium salt 2 (0.20 mmol, 1.0 equiv.), polyfluoroarene or caffeine 1 (2.0 mmol, 10.0 equiv.), [Ru(OPiv)_2_(*p*-cymene)] (10 mol%), PivOH (30 mol%), K_2_CO_3_ (0.46 mmol, 2.3 equiv.), *t*BuCN (220 µL), under N_2_ at 120 °C for 24 h, isolated yields were reported. [a] Caffeine (1.0 mmol, 5.0 equiv.) was used, *t*BuCN (300 µL). [b] *t*BuCN (300 µL).

Subsequently, we investigated the incorporation of the pentafluorophenyl motif into more complex molecular frameworks including drugs and natural products. Hence, a Flurbiprofen-derived sulfonium salt was smoothly arylated to 4. The pentafluorophenyl unit was selectively installed on the electron-rich aromatic ring of Atomoxetine, forming 5 in 89% yield. Under otherwise identical conditions, nitrogen-containing drug Pyriproxyphen was functionalized, mirroring its utility in late-stage transformation. Likewise, the Clofibrate analogue 7 was prepared with high efficiency. Notably, modifications of bio-relevant natural products, such as d-salicin and δ-Tocopherol, were achieved in good yields (8, 9). Additionally, the incorporation of a heteroarene, such as caffeine, was coupled to complex drug molecules (10, 11, Scheme 3).

### Non-ruthenacycle intermediates for arylation

**Scheme 4:**
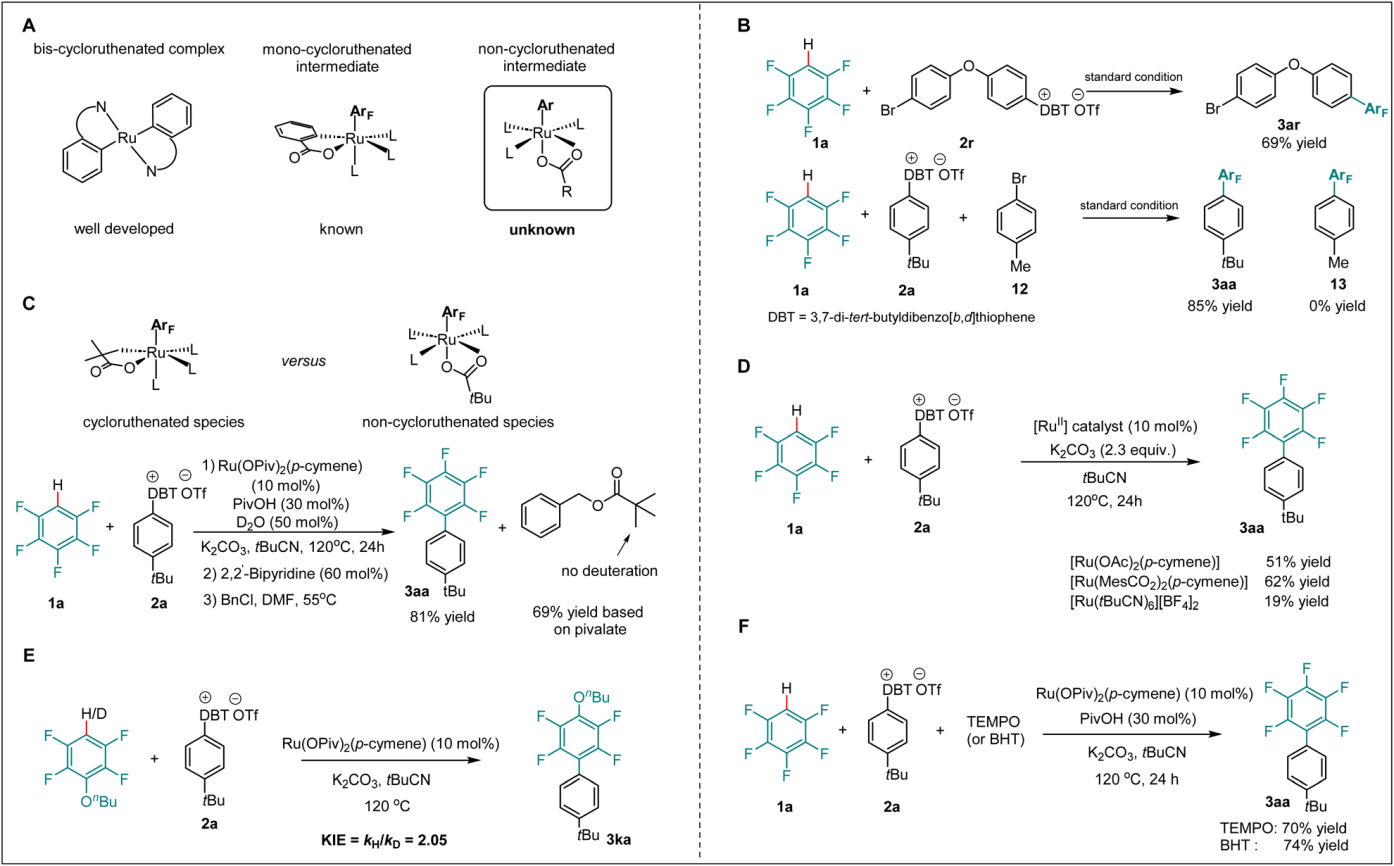
Non-ruthenacycle intermediates for arylation. (A) Proposed ruthenium(ii) intermediates for oxidative addition. (B) Competition experiment. (C) Examination of the non-cycloruthenated complex. (D) Control experiment without additive. (E) Kinetic isotope effect experiment. (F) Radical scavenger experiment.

Within ruthenium-catalyzed C–H arylation, oxidative addition was recognized as the key rate-determining step.^43,44,66^ Extensive studies revealed the crucial role of cycloruthenated intermediates for the oxidative addition of aryl halides onto ruthenium.^48–66^ Moreover, recent findings highlighted mono- and bis-cycloruthenated complexes as possible intermediates for the oxidative addition (Scheme 4A).^52–58,60–62^ Likewise, in ruthenium-catalyzed C–H arylation without directing groups, mono-cycloruthenated intermediates formed in the presence of benzoate proved critical for activating the aryl halide (Scheme 4A, middle).^19,59^ To shed light on the catalyst mode of action for the undirected C–H arylation with arylsulfonium salts, a set of mechanistic experiments was carried out. Intramolecular and intermolecular competition experiments between aryl bromides and arylsulfonium salts were performed. Although aryl bromides proved to be generally viable arylating reagents in ruthenium catalysis, we found that arylsulfonium salts outperformed aryl bromides under our standard conditions, providing a useful linchpin for further transformations of bromo-containing products (Scheme 4B). To verify the viability of a non-ruthenacycle intermediate, we carried out the desired C–H arylation in the presence of D_2_O under standard conditions.

The benzylation of the pivalate revealed no deuterium incorporation within the benzyl pivalate, which provides support for the oxidative addition of arylsulfonium salt 2a through a non-ruthenacycle intermediate, delivering the desired product in 81% yield (Scheme 4C). Furthermore, control experiments in the absence of additive, employing different ruthenium catalysts, afforded the desired product 3aa in moderate yields, further supporting that the non-ruthenacycle intermediates were responsible for the oxidative addition step (Scheme 4D). In addition, a kinetic isotope effect experiment showed a *k*_H_/*k*_D_ of 2.1, which indicated the C–H activation to be involved in or before the rate-determining step (Scheme 4E). Next, radical scavenger and radical clock experiments (see the SI) were conducted. Interestingly, neither TEMPO nor BHT substantially inhibited the formation of product 3aa, suggesting that an aryl radical is less likely involved in this reaction (Scheme 4F).

### DFT calculations

**Fig. 1:**
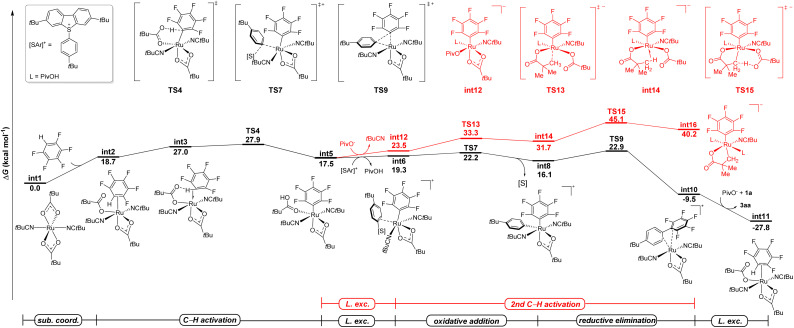
Computed relative Gibbs free energies (Δ*G*_393.15_) in kcal mol^−1^ for the ruthenium catalyzed C–H arylation of polyfluoroarenes with arylsulfonium salts at the PWPB95-D3/def2-TZVP-CPCM(*t*BuCN)//PBE0-D3(BJ)/def2-SVP level of theory.

The arylsulfonium salt enabled C–H activation prompted us to gain more insight into the mechanism of undirected C–H arylation *via* non-cycloruthenated species. Therefore, detailed studies were conducted by means of density functional theory (DFT) calculations to elaborate the C–H arylation of polyfluoroarenes at the PWPB95-D3/def2-TZVP-CPCM(*t*BuCN)//PBE0-D3(BJ)/def2-SVP level of theory (Fig. 1).^69^ Initiating from the *η*^6^-arene-free ruthenium complex [Ru(OPiv)_2_(*t*BuCN)_2_], C–H activation of the pentafluorobenzene occurred with a high barrier of 27.9 kcal mol^−1^, leading to the formation of a thermodynamically less stable aryl-ruthenium(ii) species int5 (Fig. 1, black line). This intermediate subsequently underwent ligand exchange with the dibenzothiophenium salt, accompanied by the dissociation of pivalic acid, to afford intermediate int6. The latter then underwent an oxidative addition (OA), generating the ruthenium(iv) species (int8), as confirmed by localized orbital bonding analysis (LOBA). The following reductive elimination step *via*TS9 required a barrier of 22.9 kcal mol^−1^ to afford the final polyfluoroarene product 3aa.

A viable radical pathway involving the homolytic cleavage of the S–Ar bond was also investigated (see the SI, Fig. S8). However, this mechanism was ultimately ruled out due to the prohibitively high energy barrier of 47.8 kcal mol^−1^ for the outer sphere dissociative electron transfer (DET) and 60.1 kcal mol^−1^ for the inner-sphere single electron transfer (ISET). These computational results, combined with radical scavenger and radical clock experiments, indicated that the radical pathway is likely not operative. In the process of C–H arylation, an alternative ruthenacycle pathway through a C(sp^3^)–H activation was also considered. Following the ligand exchange, an anionic species int12 was formed, which later evolved into an agostic complex int14 through the transition state TS13. Overall, the formation of a cycloruthenated intermediate features a prohibitively high energy barrier of 45.1 kcal mol^−1^ for the C(sp^3^)–H activation *via*TS15 (Fig. 1, red line). These results are in good agreement with the experimentally observed hydrogen isotope exchange (HIE) reaction depicted in Scheme 4C. Hence, a cyclometallation could indeed be ruled out by computational and experimental results. The emergence of a new model of arylation arguably implicates the potential of ruthenium(ii) catalysis for versatile C–C formation reactions that are rarely explored.

**Fig. 2:**
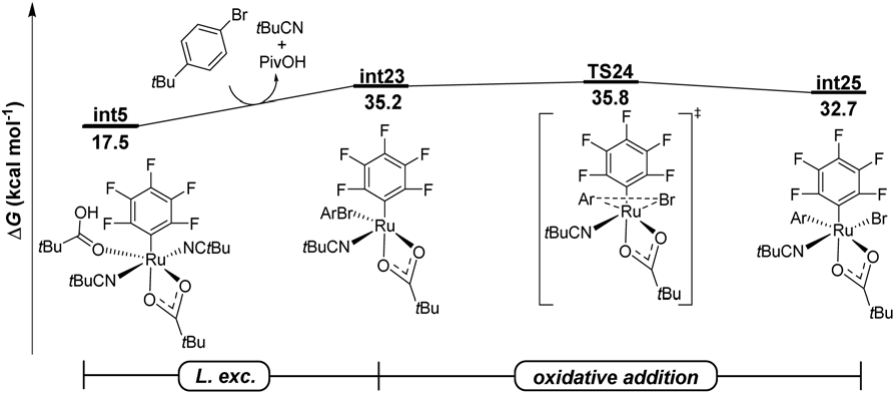
Free energy profile for the ruthenium catalyzed C–H arylation of polyfluoroarenes with *tert*-butylphenyl bromide at the PWPB95-D3/def2-TZVP-CPCM(*t*BuCN)//PBE0-D3(BJ)/def2-SVP level of theory.

To further compare the reactivity of arylsulfonium salts with more conventional aryl halides, DFT calculations were conducted for the oxidative addition elementary step in the presence of *tert*-butylphenyl bromide (Fig. 2). The commonly proposed two-electron oxidative addition pathway, which directly led to the formation of ruthenium(iv) species int25, through TS24, was found to be kinetically unfavorable with an overall energy barrier of 35.8 kcal mol^−1^. Furthermore, the formation of a *tert*-butylphenyl radical *via* either the DET or ISET pathways proved to be not feasible, as illustrated in Fig. S14 in the SI. Thus, these computational results corroborate the higher reactivity of arylsulfonium salts relative to aryl halides in the undirected C–H arylation catalytic system, which is consistent with the competition experiments where no product (13) was observed when dibenzothiophenium salt (2a) and 4-methylphenyl bromide (12) were simultaneously added to the catalytic system. This subtle reactivity of *tert*-butylphenyl bromide is likely attributed to the significantly higher bond dissociation energy (BDE) of the Br–Ar bond (82.4 kcal mol^−1^) compared to that in the dibenzothiophenium salt (69.8 kcal mol^−1^). Indeed, an effective combination of the non-cycloruthenated intermediate and highly reactive arylsulfonium salts enabled the arylation of polyfluoroarenes without directing groups.

**Scheme 5:**
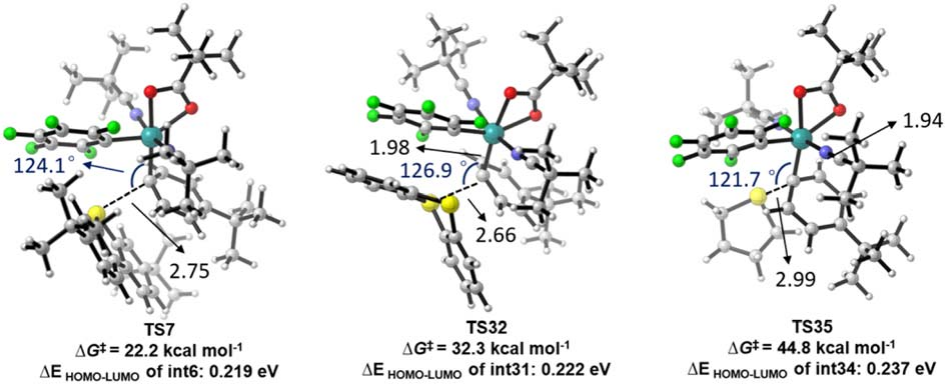
Transition states for the oxidative addition step with sulfonium salts S9, S2 and S1.

Furthermore, significant reactivity differences among arylsulfonium salts for the arylation also attracted our attention. Therefore, DFT calculations were also conducted for the oxidative addition steps with S1 and S2. As illustrated in Scheme 5, the oxidative addition step of S2 proceeded with a higher energy barrier (32.3 kcal mol^−1^ for TS32) compared to S9 (22.2 kcal mol^−1^ for TS7). Both were accessible at the reaction temperature. However, the oxidative addition of S1 exhibited a prohibitive energy barrier of 44.8 kcal mol^−1^, which aligns well with the experimental observation that no arylation product was generated (Table 1, entry 1). The increase in energy barriers from S9 and S2 to S1 correlates well with the corresponding enlargement in the HOMO–LUMO gap of the intermediates located before the oxidative addition step, suggesting that a larger HOMO–LUMO gap may hinder charge transfer during the oxidative addition.

### Data science

**Scheme 6:**
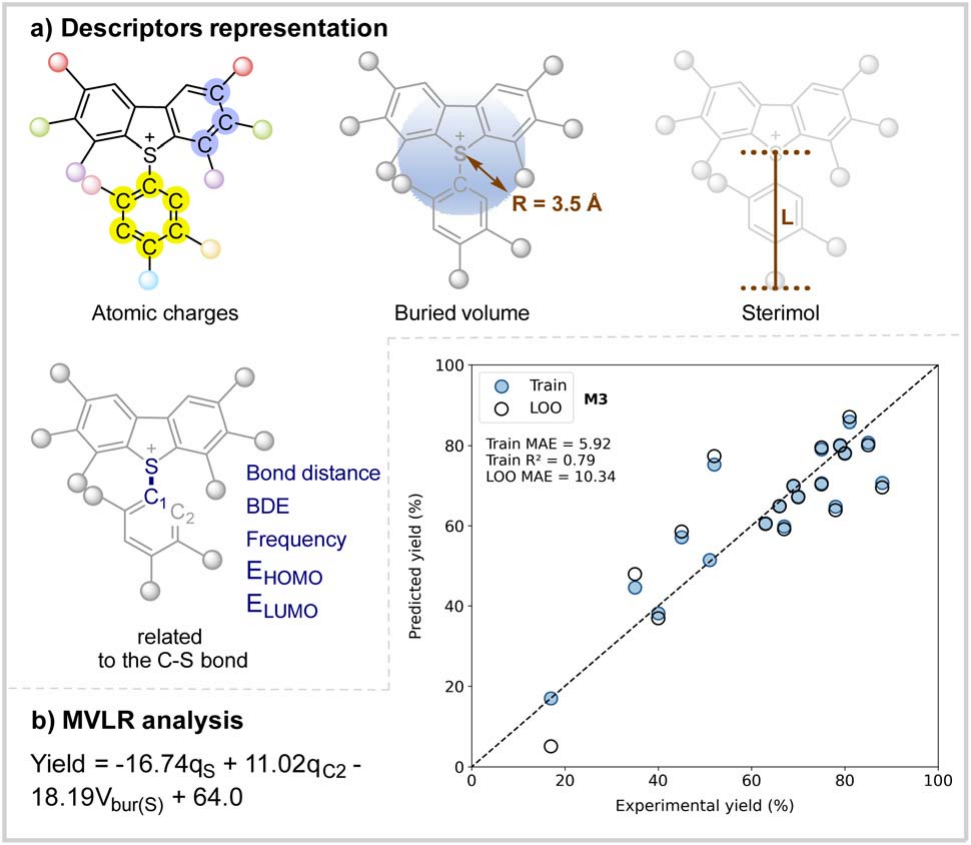
Multivariate linear regression analysis of the reactivity of the arylsulfonium salts.

To explore the features responsible for the activation of arylsulfonium salts in the reaction, multivariate linear regression (MVLR) analysis was conducted. Scheme 6a highlights the key physical organic molecular descriptors essential for the properties of arylsulfonium salts. Natural population analysis (NPA) charges for the arene moieties of the arylsulfonium salts were incorporated to represent their electronic features. To capture steric properties, the buried volume (%_Vbur_, radius = 3.5 Å) around the reactive sulfur and carbon atoms, along with the Sterimol parameters *B*_min_, *B*_max_ and L, was measured. Additional site-specific descriptors related to the C–S bond, such as bond distance, bond dissociation energy, vibrational modes, and frontier molecular orbital energies were also considered. With these parameters, the optimal regression model is showcased in Scheme 6b, exhibiting an *R*^2^ value of 0.79 and a robust cross-validation metric (LOO MAE = 10.34%). Notably, this persuasive model indicates that both the electron-donating substituents adjacent to the sulfur atom and electron-withdrawing substituents adjacent to the C2 atom contribute to the enhanced reactivity of the arylsulfonium salts. In addition to the electronic effects, arylsulfonium salts with lower buried volume also promote the reaction. Thus, the selected MVLR model offers a quantitative framework to understand the molecular properties of the arylsulfonium salts in this catalytic arylation, providing a theoretical basis for further exploration of the reactivity of arylsulfonium salts.

## Conclusion

We devised an undirected ruthenium-catalyzed C–H arylation of polyfluoroarenes utilizing arylsulfonium salts. The notable functional group tolerance was, among others, reflected by chemo-selective late-stage polyfluoroarylation. Notably, the use of sulfonium salt aryl transfer agents enabled an unprecedented pathway, being devoid of any ruthenacycle intermediates. The combination of mechanistic experiments and detailed DFT calculations demonstrated the higher reactivity of arylsulfonium salts as compared with conventional aryl halides, thereby enabling oxidative addition to occur without any metallacycle. A data science analysis was employed to identify a multivariate linear regression model for reactivity of arylsulfonium salts in the undirected ruthenium-catalyzed C–H arylation.

## Author contributions

L. A. designed and conceived the project. J. Z. and B. Y. prepared the manuscript and SI. J. Z., X. C., and H. C. G. conducted the experiments. B. Y. performed the theoretical calculations and data science analysis. L. A. revised the manuscript.

## Conflicts of interest

The authors declare no competing interests.

## Supplementary Material

SC-017-D5SC08962J-s001

SC-017-D5SC08962J-s002

## Data Availability

All data that support the findings of this study are available within the paper and its supplementary information (SI) files, and are also available from the corresponding author upon request. Supplementary information is available. See DOI: https://doi.org/10.1039/d5sc08962j.
