# Supplementary material for: Undirected ruthenium-catalyzed C–H activation using arylsulfonium salts: direct arylation without ruthenacycle intermediates revealed by computation and data science
Source: Chem Sci. 2026 Jan 23;17(12):6138–46. doi: 10.1039/d5sc08962j (PMC12865703; doi:10.1039/d5sc08962j)
Supplement: SC-017-D5SC08962J-s001 [file SC-017-D5SC08962J-s001.pdf]

Supplementary Information (SI) for Chemical Science.
This journal is © The Royal Society of Chemistry 2026

Supporting Information

# Undirected Ruthenium-Catalyzed C−H Activations Using Arylsulfonium Salts: Direct Arylations without Ruthenacycle Intermediates by Computation and Data Science

Jinbin Zhu[+], Binbin Yuan[+], Xuexue Chang, Hasret Can Gülen, and Lutz Ackermann[*]

# Table of Contents

1. **General Remark……………………………………………………………...S3**
2. **Synthesis of Arylsulfonium Salts…………………………….………………S4**
3. **Optimization of the Reaction Conditions……………………………………..S13**
4. **General Procedure for C–H Arylation and Product Characterization…………………………………………………………….…S15**
5. **Late-Stage Incorporation of Pentafluorophenyl Motif………………………S28**
6. **Mechanistic Experimental Studies …………………………………………...S37**
7. **Computational Studies………………………………………………………...S48**
8. **Data Science Analysis………………………………………………………S58**
9. **References……………………………………………………………………...S67**
10. **Cartesian Coordinates of the Optimized Structures……………………….…...S69**
11. **NMR Spectra…………………………………………………………….....S113**

## 1. General Remark

Catalytic reactions were performed in screw-cap pressure tubes under nitrogen atmosphere. Pivalonitrile was distilled under nitrogen atmosphere prior to use. All polyfluoroarenes were degassed with 3 freeze-pump-thaw cycles. Arylsulfonium salts were prepared following previously reported methods. Other chemicals were obtained from commercial sources and were used without further purification. Yields refer to isolated compounds, estimated to be >95% pure as determined by $^1$H-NMR. TLC: Macherey-Nagel, TLC plates Alugramm®Sil G/UV254. Detection under UV light at 254 nm. Chromatography: Separations were carried out on Merck Silica 60 (0.040-0.063 mm, 70-230 mesh ASTM). All IR spectra were recorded on a Bruker FT-IR Alpha-P spectrometer. EI-MS spectra were recorded on Jeol AccuTOF at 70Ev, ESI-MS spectra on Bruker MicrOTOF and maXis. $^1$H, $^{13}$C, and $^{19}$F NMR spectra were recorded at 300 ($^1$H), 400 ($^1$H), 75, 101 [$^{13}$C, APT (Attached Proton Test)], and 282, 377 ($^{19}$F) MHz respectively on an AVANCE NEO 300 MHz or Bruker Avance III 400 in the solvent indicated. Chemical shifts are given in ppm relative to the residual solvent signal.

## 2. Synthesis of Arylsulfonium salts.

**S1**[1] **S2**[2] **S4**[3] **S6**[4] were prepared according to reported literature.

**Reaction condition A** for syntheses of dibenzothiophenium salts.

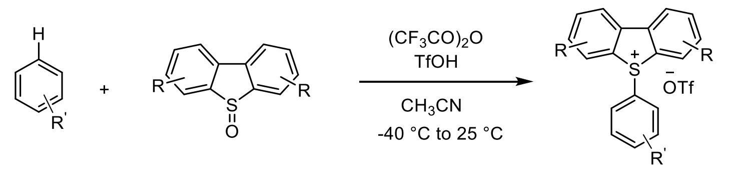


A dry 100 mL Schlenk flask equipped with a magnetic stir bar was charged with arene (1.92 mmol) and dry MeCN (8 mL) under nitrogen atmosphere at room temperature. After cooling to -40 °C, $(CF_3CO)_2O$ (0.66 mL, 4.80 mmol) and TfOH (0.35 mL, 3.84 mmol) were added to the stirred reaction mixture. Subsequently, dibenzothiophene S-oxide (1.92 mmol) was added to the stirred reaction mixture in small portions over 3 minutes. After addition, the reaction mixture was stirred at -40 °C for 1 h. Next, the Schlenk flask was taken out of the cold bath and warmed to 25 °C in air. After stirring at 25 °C for another 1 h, the reaction mixture was evaporated in vacuum to remove most of solvent, then the residue was diluted with DCM (50 mL) and poured onto saturated aqueous $NaHCO_3$ (20 mL). Then, the mixture was poured into a separatory funnel, and the layers were separated. The DCM layer was collected, and washed with aqueous NaOTf solution (15 mL x 2, 5 wt%), dried over anhydrous $Na_2SO_4$. After filtration, the mixture was concentrated to dryness under reduced pressure, the residue was purified by chromatography on silica gel or precipitation using diethyl ether.

**Reaction condition B** for syntheses of dibenzothiophenium salts.

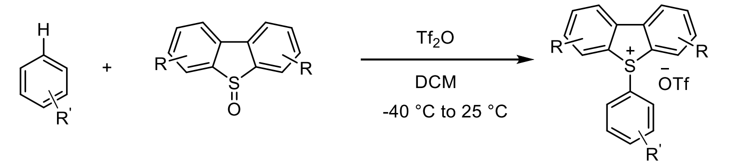


A dry 100 mL Schlenk flask equipped with a magnetic stir bar was charged with arene (1.92 mmol), dibenzothiophene S-oxide (1.92 mmol), and dry DCM (8 mL) under nitrogen atmosphere at room temperature. After cooling to -40 °C, $Tf_2O$ (2.50 mmol) was added dropwise over 3 minutes. After addition, the reaction mixture was stirred at -40 °C for 1 h. Next, the Schlenk flask was taken out of the cold bath and warmed to 25 °C in air. After stirring at 25 °C for another 1 h, the mixture was poured onto saturated aqueous $NaHCO_3$ (20 mL). Then, the mixture was poured into a separatory funnel, and the layers were separated. The DCM layer was collected, and washed with aqueous NaOTf solution (15 mL x 2 (5 wt%)), dried over anhydrous $Na_2SO_4$. After filtration, the mixture was concentrated to dryness under reduced pressure, the residue was purified by chromatography on silica gel or precipitation using diethyl ether.

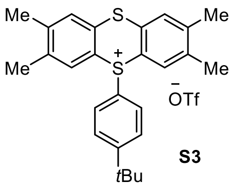

**S3**

**5-(4-(*tert*-butyl)phenyl)-2,3,7,8-tetramethyl-*5H*-thianthren-5-ium trifluoromethanesulfonate (S3)**

According to reported literature[5], **S3** was obtained as white solid by precipitation (636 mg, 73% yield). $^1$H NMR (400 MHz, $CDCl_3$): δ = 8.22 (s, 2H), 7.53 (s, 2H), 7.42-7.39 (m, 2H), 7.19-7.15 (m, 2H), 2.40 (s, 6H), 2.38 (s, 6H), 1.21 (s, 9H); $^{13}$C NMR (101 MHz, $CDCl_3$): δ = 156.9 ($C_q$), 145.3 ($C_q$), 139.8 ($C_q$), 135.0 (CH), 133.7 ($C_q$), 130.9 (CH), 127.9 (CH), 127.8 (CH), 121.8 ($C_q$), 121.0 (q, *J* = 320.9 Hz, $C_q$), 115.7 ($C_q$), 35.2 ($C_q$), 30.9 ($CH_3$), 20.3 ($CH_3$), 19.5 ($CH_3$); $^{19}$F NMR (377 MHz, $CDCl_3$): δ = -78.08 (s). HRMS-ESI (m/z) calculated for $C_{26}H_{29}S_2^+$ [M-OTf]$^+$, 405.1705; found: 405.1706.

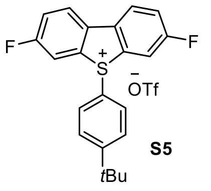

**S5**

**5-(4-(*tert*-butyl)phenyl)-3,7-difluoro-*5H*-dibenzo[*b,d*]thiophen-5-ium trifluoromethanesulfonate (S5)**

Following reaction condition A, **S5** was obtained as white solid by precipitation (1.13 g, 90% yield). $^1$H NMR (400 MHz, DMSO-$d_6$): δ = 8.57 (dd, *J* = 8.7, 4.8 Hz, 2H), 8.35 (dd, *J* = 8.0, 2.5 Hz, 2H), 7.88 (td, *J* = 8.7, 2.5 Hz, 2H), 7.65-7.62 (m, 2H), 7.58-7.54 (m, 2H), 1.24 (s, 9H); $^{13}$C NMR (101 MHz, DMSO-$d_6$): δ = 162.3 (d, *J* = 251.0 Hz, $C_q$) 157.7 ($C_q$), 135.5 (d, *J* = 11.2 Hz, $C_q$), 135.0 (d, *J* = 1.9 Hz, $C_q$), 129.8 (CH), 128.4 (CH), 126.1 (d, *J* = 9.2 Hz, CH), 124.6 ($C_q$), 121.6 (d, *J* = 23.1 Hz, CH), 115.7 (d, *J* = 28.3 Hz, CH), 120.7 (q, *J* = 322.2 Hz, $C_q$), 35.1 ($C_q$), 30.5 ($CH_3$); $^{19}$F NMR (377 MHz, DMSO-$d_6$): δ = -77.74 (s), -108.08 (td, *J* = 8.3, 4.8 Hz). HRMS-ESI (m/z) calculated for $C_{22}H_{19}F_2S^+$ [M-OTf]$^+$, 353.1170; found: 353.1169.

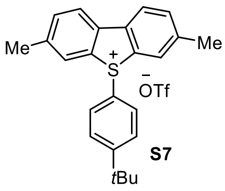

**S7**

**5-(4-(*tert*-butyl)phenyl)-3,7-dimethyl-*5H*-dibenzo[*b,d*]thiophen-5-ium trifluoromethanesulfonate (S7)**

Following reaction condition A, **S7** was obtained as colorless solid (563 mg, 45% yield), purification by column chromatography on silica gel (DCM/$CH_3OH$: 30/1). $^1$H NMR (400 MHz, $CDCl_3$): δ = 8.00 (d, *J* = 8.0 Hz, 2H), 7.84 (s, 2H), 7.59-7.54 (m, 4H), 7.53-7.49 (m, 2H), 2.42 (s, 6H), 1.27 (s, 9H); $^{13}$C NMR (101 MHz, $CDCl_3$): δ = 159.2 ($C_q$), 142.3 ($C_q$), 136.3 ($C_q$), 135.1 (CH), 132.0 ($C_q$), 130.5 (CH), 128.8 (CH), 128.4 (CH), 123.4 (CH), 122.7 ($C_q$), 120.9 (q, *J* = 322.2 Hz, $C_q$), 35.4 ($C_q$), 30.8 ($CH_3$), 21.6 ($CH_3$); $^{19}$F NMR (377 MHz, $CDCl_3$): δ = -78.14 (s). HRMS-ESI (m/z) calculated for $C_{24}H_{25}S^+$ [M-OTf]$^+$, 345.1671; found: 345.1671.

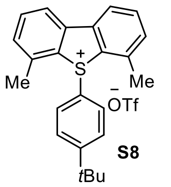

**5-(4-(*tert*-butyl)phenyl)-4,6-dimethyl-*5H*-dibenzo[*b,d*]thiophen-5-ium trifluoromethanesulfonate (S8)**

Following reaction condition A, **S8** was obtained as colorless solid (681 mg, 35% yield), purification by column chromatography on silica gel (DCM/$CH_3OH$: 40/1). $^1H$ NMR (400 MHz, $CDCl_3$): δ = 8.04 (d, $J$ = 7.8 Hz, 2H), 7.75 (t, $J$ = 7.7 Hz, 2H), 7.64-7.60 (m, 2H), 7.58-7.53 (m, 2H), 7.35 (d, $J$ = 7.6 Hz, 2H), 2.46 (s, 6H), 1.27 (s, 9H); $^{13}C$ NMR (101 MHz, $CDCl_3$): δ = 159.8 ($C_q$), 139.7 ($C_q$), 139.2 ($C_q$), 135.0 (CH), 132.6 (CH), 131.2 ($C_q$), 130.9 (CH), 129.2 (CH), 122.2 (CH), 120.8 (q, $J$ = 320.9 Hz, $C_q$), 118.6 ($C_q$), 35.7 ($C_q$), 31.0 ($CH_3$), 20.1 ($CH_3$); $^{19}F$ NMR (377 MHz, $CDCl_3$): δ = -78.14 (s). HRMS-ESI (m/z) calculated for $C_{24}H_{25}S^+$ [M-OTf]$^+$, 345.1671; found: 345.1670.

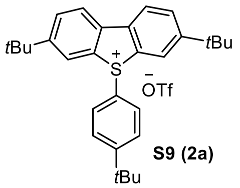

**3,7-di-*tert*-butyl-5-(4-(*tert*-butyl)phenyl)-*5H*-dibenzo[*b,d*]thiophen-5-ium trifluoromethanesulfonate (S9)**

Following reaction condition A, **S9 (2a)** was obtained as colorless solid (3.26 g, 88% yield), purification by column chromatography on silica gel (DCM/$CH_3OH$: 50/1). $^1H$ NMR (400 MHz, $CDCl_3$): δ = 8.07 - 8.05 (m, 4H), 7.85 (dd, $J$ = 8.3, 1.7 Hz, 2H), 7.64 – 7.60 (m, 2H), 7.56 – 7.52 (m, 2H), 1.33 (s, 18H), 1.28 (s, 9H); $^{13}C$ NMR (101 MHz, $CDCl_3$): δ = 159.2 ($C_q$), 156.0 ($C_q$), 136.4 ($C_q$), 132.5 ($C_q$), 131.8 (CH), 130.7 (CH), 129.0 (CH), 125.3 (CH), 123.3 (CH), 123.1 ($C_q$), 121.0 (q, $J$ = 322.2 Hz, $C_q$), 35.9 ($C_q$), 35.6 ($C_q$), 31.2 ($CH_3$), 31.0 ($CH_3$); $^{19}F$ NMR (377 MHz, $CDCl_3$): δ = -78.06 (s). HRMS-ESI (m/z) calculated for $C_{30}H_{37}S^+$ [M-OTf]$^+$, 429.2610; found: 429.2610.

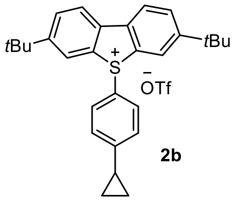

**3,7-di-*tert*-butyl-5-(4-cyclopropylphenyl)-*5H*-dibenzo[*b,d*]thiophen-5-ium trifluoromethanesulfonate (2b)**

Following reaction condition A, **2b** was obtained as white solid (1.29 g, 90% yield), purification by precipitation. $^1H$ NMR (300 MHz, $CDCl_3$): δ = 8.05 (d, $J$ = 3.4 Hz, 2H), 8.03 (d, $J$ = 3.0 Hz, 2H), 7.84 (dd, $J$ = 8.3, 1.7 Hz, 2H), 7.58-7.53 (m, 2H), 7.19-7.14 (m, 2H), 1.90 (tt, $J$ = 8.3, 5.0 Hz, 1H), 1.33 (s, 18H), 1.12-1.06 (m, 2H), 0.76 (dt, $J$ = 7.0, 4.8 Hz, 2H); $^{13}C$ NMR (75 MHz, $CDCl_3$): δ = 156.1 ($C_q$), 153.3 ($C_q$), 136.3 ($C_q$), 132.8 ($C_q$), 131.8 (CH), 131.0 (CH), 128.6 (CH), 125.2 (CH), 123.3 (CH), 122.1 ($C_q$), 121.0 (q, $J$ = 318.7 Hz, $C_q$), 35.9 ($C_q$), 31.2 ($CH_3$), 16.0 (CH), 11.4 ($CH_2$); $^{19}F$ NMR (282 MHz, $CDCl_3$): δ = -78.06 (s). HRMS-ESI (m/z) calculated for $C_{29}H_{33}S^+$ [M-OTf]$^+$, 413.2297; found: 413.2297.

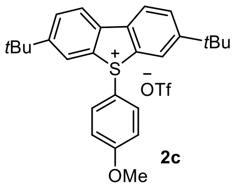

**3,7-di-*tert*-butyl-5-(4-methoxyphenyl)-*5H*-dibenzo[*b,d*]thiophen-5-ium trifluoromethanesulfonate (2c)**

Following reaction condition A, **2c** was obtained as white solid (1.64 g, 93% yield), purification by precipitation. $^{1}H$ NMR (400 MHz, $CDCl_3$): δ = 8.04 (d, $J$ = 8.3 Hz, 2H), 8.02 (d, $J$ = 1.7 Hz, 2H), 7.83 (dd, $J$ = 8.2, 1.7 Hz, 2H), 7.65-7.61 (m, 2H), 7.04-7.00 (m, 2H), 3.84 (s, 3H), 1.33 (s, 18H); $^{13}C$ NMR (101 MHz, $CDCl_3$): δ = 165.0 ($C_q$), 156.0 ($C_q$), 136.1 ($C_q$), 133.4 (CH), 133.1 ($C_q$), 131.8 (CH), 125.1 (CH), 123.3 (CH), 121.0 (q, $J$ = 322.2 Hz, $C_q$), 117.3 (CH), 115.2 ($C_q$), 56.1 ($CH_3$), 35.9 ($C_q$), 31.2 ($CH_3$); $^{19}F$ NMR (377 MHz, $CDCl_3$): δ = -78.03 (s). HRMS-ESI (m/z) calculated for $C_{27}H_{31}OS^+$ $[M-OTf]^+$, 403.2090; found: 403.2091.

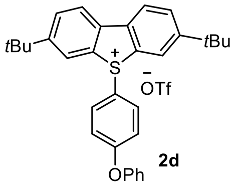

**3,7-di-*tert*-butyl-5-(4-phenoxyphenyl)-*5H*-dibenzo[*b,d*]thiophen-5-ium trifluoromethanesulfonate (2d)**

Following reaction condition A, **2d** was obtained as white solid (1.87 g, 95% yield), purification by column chromatography on silica gel (DCM/$CH_3OH$: 50/1). $^{1}H$ NMR (400 MHz, $CDCl_3$): δ = 8.07 (d, $J$ = 1.7 Hz, 2H), 8.04 (d, $J$ = 8.3 Hz, 2H), 7.84 (dd, $J$ = 8.3, 1.7 Hz, 2H), 7.66-7.62 (m, 2H), 7.41-7.36 (m, 2H), 7.24-7.20 (m, 1H), 7.06-6.99 (m, 4H), 1.33 (s, 18H); $^{13}C$ NMR (101 MHz, $CDCl_3$): δ = 163.9 ($C_q$), 156.1 ($C_q$), 154.0 ($C_q$), 136.2 ($C_q$), 133.4 (CH), 132.9 ($C_q$), 131.9 (CH), 130.5 (CH), 125.9 (CH), 125.2 (CH), 123.3 (CH), 121.0 (q, $J$ = 322.2 Hz, $C_q$), 120.9 (CH), 119.5 (CH), 117.6 ($C_q$), 35.9 ($C_q$), 31.2 ($CH_3$); $^{19}F$ NMR (377 MHz, $CDCl_3$): δ = -78.07 (s). HRMS-ESI (m/z) calculated for $C_{32}H_{33}OS^+$ $[M-OTf]^+$, 465.2247; found: 465.2246.

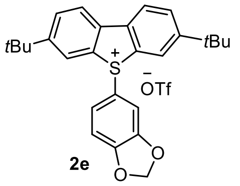

**5-(benzo[*d*][1,3]dioxol-5-yl)-3,7-di-*tert*-butyl-*5H*-dibenzo[*b,d*]thiophen-5-ium trifluoromethanesulfonate (2e)**

Following reaction condition A, **2e** was obtained as white solid (1.63 g, 90% yield), purification by precipitation. $^{1}H$ NMR (300 MHz, $CDCl_3$): δ = 8.06 (d, $J$ = 1.7 Hz, 2H), 8.03 (d, $J$ = 8.3 Hz, 2H), 7.92 (dd, $J$ = 8.3, 2.1 Hz, 1H), 7.84 (dd, $J$ = 8.2, 1.7 Hz, 2H), 7.00 (d, $J$ = 8.3 Hz, 1H), 6.38 (d, $J$ = 2.0 Hz, 1H), 6.04 (s, 2H), 1.34 (s, 18H); $^{13}C$ NMR (75 MHz, $CDCl_3$): δ = 156.1 ($C_q$), 154.0 ($C_q$), 150.3 ($C_q$), 136.1 ($C_q$), 132.7 ($C_q$), 131.9 (CH), 130.7 (CH), 125.3 (CH), 123.3 (CH), 121.0 (q, $J$ = 318.8 Hz, $C_q$), 116.8 ($C_q$), 110.6 (CH), 107.3 (CH), 103.3 ($CH_2$), 35.9 ($C_q$), 31.2 ($CH_3$); $^{19}F$ NMR (282 MHz, $CDCl_3$): δ = -78.09 (s). HRMS-ESI (m/z) calculated for $C_{27}H_{29}O_2S^+$ $[M-OTf]^+$, 417.1883; found: 417.1884.

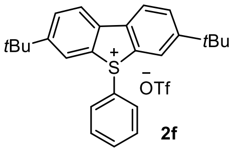

2f

**3,7-di-*tert*-butyl-5-phenyl-*5H*-dibenzo[*b,d*]thiophen-5-ium trifluoromethanesulfonate (2f)**

Following reaction condition A, **2f** was obtained as colorless solid (1.20 g, 72% yield), purification by column chromatography on silica gel (DCM/$CH_3OH$: 50/1). $^1H$ NMR (300 MHz, $CDCl_3$): δ = 8.09 (d, *J* = 1.7 Hz, 2H), 8.06 (d, *J* = 8.3 Hz, 2H), 7.85 (dd, *J* = 8.3, 1.7 Hz, 2H), 7.74 – 7.63 (m, 3H), 7.57 – 7.51 (m, 2H), 1.33 (s, 18H); $^{13}C$ NMR (75 MHz, $CDCl_3$): δ = 156.2 ($C_q$), 136.5 ($C_q$), 135.0 (CH), 132.4 ($C_q$), 131.9 (CH), 131.7 (CH), 130.9 (CH), 127.3 ($C_q$), 125.4 (CH), 123.4 (CH), 121.0 (q, *J* = 318.0 Hz, $C_q$), 35.9 ($C_q$), 31.2 ($CH_3$); $^{19}F$ NMR (282 MHz, $CDCl_3$): δ = -78.09 (s). HRMS-ESI (m/z) calculated for $C_{26}H_{29}S^+$ $[M-OTf]^+$, 373.1984; found: 373.1986.

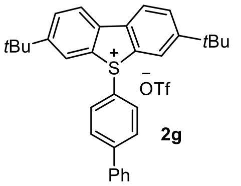

2g

**5-([1,1'-biphenyl]-4-yl)-3,7-di-*tert*-butyl-*5H*-dibenzo[*b,d*]thiophen-5-ium trifluoromethanesulfonate (2g)**

Following reaction condition A, **2g** was obtained as colorless solid (666 mg, 58% yield), purification by column chromatography on silica gel (DCM/$CH_3OH$: 50/1). $^1H$ NMR (300 MHz, $CDCl_3$): δ = 8.12 (d, *J* = 1.8 Hz, 2H), 8.10 (d, *J* = 4.8 Hz, 2H), 7.87 (dd, *J* = 8.3, 1.7 Hz, 2H), 7.80 – 7.69 (m, 4H), 7.57 – 7.49 (m, 2H), 7.47 – 7.33 (m, 3H), 1.33 (s, 18H); $^{13}C$ NMR (75 MHz, $CDCl_3$): δ = 156.1 ($C_q$), 147.9 ($C_q$), 138.2 ($C_q$), 136.4 ($C_q$), 132.4 ($C_q$), 132.0 (CH), 131.4 (CH), 130.1 (CH), 129.3 (CH), 129.2 (CH), 127.4 (CH), 125.3 (CH), 125.0 ($C_q$), 123.5 (CH), 121.0 (q, *J* = 318.8 Hz, $C_q$), 35.9 ($C_q$), 31.1 ($CH_3$); $^{19}F$ NMR (282 MHz, $CDCl_3$): δ = -78.00 (s). HRMS-ESI (m/z) calculated for $C_{32}H_{33}S^+$ $[M-OTf]^+$, 449.2297; found: 449.2300.

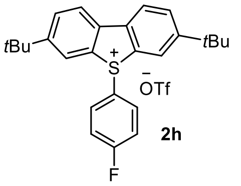

2h

**3,7-di-*tert*-butyl-5-(4-fluorophenyl)-*5H*-dibenzo[*b,d*]thiophen-5-ium trifluoromethanesulfonate (2h)**

Following reaction condition B, **2h** was obtained as colorless solid (346 mg, 40% yield), purification by column chromatography on silica gel (DCM/$CH_3OH$: 50/1). $^1H$ NMR (400 MHz, $CDCl_3$): δ = 8.14 (d, *J* = 1.7 Hz, 2H), 8.02 (d, *J* = 8.2 Hz, 2H), 7.86 (dd, *J* = 8.3, 1.7 Hz, 2H), 7.82 – 7.77 (m, 2H), 7.25 – 7.20 (m, 2H), 1.34 (s, 18H); $^{13}C$ NMR (101 MHz, $CDCl_3$): δ = 166.5 (d, *J* = 259.8 Hz, $C_q$ ), 156.4 ($C_q$), 136.3 ($C_q$), 134.0 (d, *J* = 9.8 Hz, CH), 132.7 ($C_q$), 132.0 (CH), 125.7 (CH), 123.2 (CH), 122.3 (d, *J* = 3.3 Hz, $C_q$), 121.0 (q, *J* = 321.2 Hz, $C_q$), 119.3 (d, *J* = 23.3 Hz, CH), 36.0 ($C_q$), 31.2 ($CH_3$); $^{19}F$ NMR (377 MHz, $CDCl_3$): δ = -78.19 (s), -100.24 (ddd, *J* = 12.5, 8.0, 4.6 Hz). HRMS-ESI (m/z) calculated for $C_{26}H_{28}FS^+$ $[M-OTf]^+$, 391.1890; found: 391.1893.

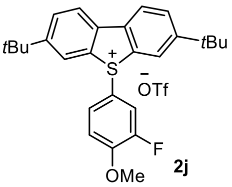

2j

**3,7-di-*tert*-butyl-5-(3-fluoro-4-methoxyphenyl)-*5H*-dibenzo[*b,d*]thiophen-5-ium trifluoromethanesulfonate (2j)**

Following reaction condition B, **2j** was obtained as white solid (1.16 g, 91% yield), purification by precipitation. $^1$H NMR (400 MHz, $CDCl_3$): δ = 8.25 (ddd, *J* = 8.8, 2.4, 1.3 Hz, 1H), 8.10 (d, *J* = 1.7 Hz, 2H), 8.02 (d, *J* = 8.3 Hz, 2H), 7.85 (dd, *J* = 8.3, 1.7 Hz, 2H), 7.28 – 7.22 (m, 1H), 6.75 (dd, *J* = 9.8, 2.5 Hz, 1H), 3.95 (s, 3H), 1.34 (s, 18H); $^{13}$C NMR (101 MHz, $CDCl_3$): δ = 156.4 ($C_q$), 153.9 (d, *J* = 10.3 Hz, $C_q$ ), 153.1 (d, *J* = 255.8 Hz, $C_q$), 136.1 ($C_q$), 132.7 ($C_q$), 132.2 (d, *J* = 3.6 Hz, CH), 132.0 (CH), 125.5 (CH), 123.3 (CH), 121.0 (q, *J* = 322.2 Hz, $C_q$), 115.7 (d, *J* = 21.3 Hz, CH), 115.7 ($C_q$), 115.2 (d, *J* = 2.3 Hz, CH), 56.9 ($CH_3$), 36.0 ($C_q$), 31.2 ($CH_3$); $^{19}$F NMR (377 MHz, $CDCl_3$): δ = -78.10 (s), -126.98 – -127.06 (m). HRMS-ESI (m/z) calculated for $C_{27}H_{30}FOS^+$ $[M\text{-}OTf]^+$, 421.1996; found: 421.1996.

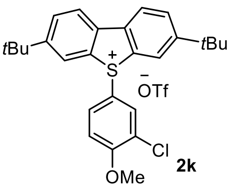

2k

**3,7-di-*tert*-butyl-5-(3-chloro-4-methoxyphenyl)-*5H*-dibenzo[*b,d*]thiophen-5-ium trifluoromethanesulfonate (2k)**

Following reaction condition B, **2k** was obtained as white solid (1.22 g, 93% yield), purification by precipitation. $^1$H NMR (400 MHz, $CDCl_3$): δ = 8.14 (dd, *J* = 8.9, 2.5 Hz, 1H), 8.06 (d, *J* = 4.8 Hz, 2H), 8.05 (d, *J* = 1.6 Hz, 2H), 7.86 (dd, *J* = 8.3, 1.6 Hz, 2H), 7.20 (d, *J* = 9.0 Hz, 1H), 7.17 (d, *J* = 2.5 Hz, 1H), 3.95 (s, 3H), 1.33 (s, 18H); $^{13}$C NMR (101 MHz, $CDCl_3$): δ= 160.6 ($C_q$), 156.3 ($C_q$), 136.1 ($C_q$), 134.2 (CH), 132.4 ($C_q$), 132.1 (CH), 130.3 (CH), 125.9 ($C_q$), 125.2 (CH), 123.5 (CH), 121.0 (q, *J* = 322.2 Hz, $C_q$), 116.2 ($C_q$), 114.3 (CH), 57.1 ($CH_3$), 35.9 ($C_q$), 31.1 ($CH_3$); $^{19}$F NMR (377 MHz, $CDCl_3$): δ = -78.09 (s). HRMS-ESI (m/z) calculated for $C_{27}H_{30}ClOS^+$ $[M\text{-}OTf]^+$, 437.1700; found: 437.1699.

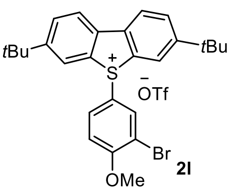

2l

**5-(3-bromo-4-methoxyphenyl)-3,7-di-*tert*-butyl-*5H*-dibenzo[*b,d*]thiophen-5-ium trifluoromethanesulfonate (2l)**

Following reaction condition B, **2l** was obtained as white solid (1.29 g, 91% yield), purification by precipitation. $^1$H NMR (400 MHz, $CDCl_3$): δ = 8.28 (dd, *J* = 8.9, 2.5 Hz, 1H), 8.07 (d, *J* = 1.7 Hz, 2H), 8.04 (d, *J* = 8.3 Hz, 2H), 7.86 (dd, *J* = 8.3, 1.7 Hz, 2H), 7.26 (d, *J* = 2.7 Hz, 1H), 7.17 (d, *J* = 8.9 Hz, 1H), 3.96 (s, 3H), 1.34 (s, 18H); $^{13}$C NMR (101 MHz, $CDCl_3$): δ= 161.4 ($C_q$), 156.3 ($C_q$), 136.1 ($C_q$), 135.2 (CH), 133.1 (CH), 132.5 ($C_q$), 132.1 (CH), 125.3 (CH), 123.4 (CH), 121.0 (q, *J* = 322.2 Hz, $C_q$), 116.6 ($C_q$), 114.8 ($C_q$), 114.0 (CH), 57.2 ($CH_3$), 36.0 ($C_q$), 31.2 ($CH_3$); $^{19}$F NMR (377 MHz,

$CDCl_3$): δ = -78.10 (s). HRMS-ESI (m/z) calculated for $C_{27}H_{30}BrOS^+$ [M-OTf]$^+$, 481.1195; found: 481.1198.

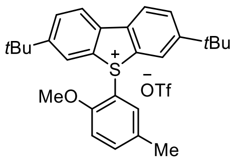

**2m**

**3,7-di-*tert*-butyl-5-(2-methoxy-5-methylphenyl)-*5H*-dibenzo[*b,d*]thiophen-5-ium trifluoromethanesulfonate (2m)**

Following reaction condition A, **2m** was obtained as white solid (1.14 g, 90% yield), purification by precipitation. $^1$H NMR (300 MHz, $CDCl_3$): δ = 8.09 (d, *J* = 8.3 Hz, 2H), 8.04 (d, *J* = 1.7 Hz, 2H), 7.86 (dd, *J* = 8.3, 1.8 Hz, 2H), 7.45 (ddd, *J* = 8.5, 2.1, 0.7 Hz, 1H), 7.11 (d, *J* = 8.6 Hz, 1H), 6.91 (d, *J* = 1.9 Hz, 1H), 3.93 (s, 3H), 2.21 (s, 3H), 1.36 (s, 18H); $^{13}$C NMR (75 MHz, $CDCl_3$): δ = 157.6 ($C_q$), 155.6 ($C_q$), 137.9 (CH), 137.0 ($C_q$), 133.5 ($C_q$), 131.7 (CH), 130.1 ($C_q$), 129.9 (CH), 125.0 (CH), 123.6 (CH), 121.1 (q, *J* = 319.5 Hz, $C_q$), 114.2 (CH), 113.1 ($C_q$), 57.5 ($CH_3$), 35.9 ($C_q$), 31.2 ($CH_3$), 20.6 ($CH_3$); $^{19}$F NMR (377 MHz, $CDCl_3$): δ = -78.12 (s). HRMS-ESI (m/z) calculated for $C_{28}H_{33}OS^+$ [M-OTf]$^+$, 417.2247; found: 417.2247.

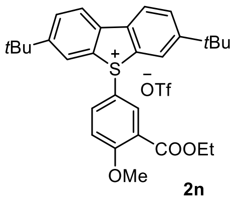

**2n**

**3,7-di-*tert*-butyl-5-(3-(ethoxycarbonyl)-4-methoxyphenyl)-*5H*-dibenzo[*b,d*]thiophen-5-ium trifluoromethanesulfonate (2n)**

Following reaction condition A, **2n** was obtained as white solid (1.31 g, 94% yield), purification by precipitation. $^1$H NMR (300 MHz, $CDCl_3$): δ = 8.12 (dd, *J* = 9.1, 2.6 Hz, 1H), 8.09 – 8.00 (m, 4H), 7.85 (dd, *J* = 8.2, 1.7 Hz, 2H), 7.75 (d, *J* = 2.7 Hz, 1H), 7.23 (d, *J* = 9.1 Hz, 1H), 4.26 (q, *J* = 7.1 Hz, 2H), 3.94 (s, 3H), 1.33 (s, 18H), 1.28 (t, *J* = 7.1 Hz, 3H); $^{13}$C NMR (75 MHz, $CDCl_3$): δ = 164.0 ($C_q$), 163.8 ($C_q$), 156.2 ($C_q$), 137.6 (CH), 136.2 ($C_q$), 133.5 (CH), 132.5 ($C_q$), 132.0 (CH), 125.2 (CH), 123.8 ($C_q$), 123.4 (CH), 121.0 (q, J = 318.0 Hz, $C_q$), 115.8 ($C_q$), 115.4 (CH), 61.9 ($CH_2$), 56.9 ($CH_3$), 35.9 ($C_q$), 31.1 ($CH_3$), 14.1 ($CH_3$); $^{19}$F NMR (282 MHz, $CDCl_3$): δ = -78.10 (s). HRMS-ESI (m/z) calculated for $C_{30}H_{35}O_3S^+$ [M-OTf]$^+$, 475.2301; found: 475.2302.

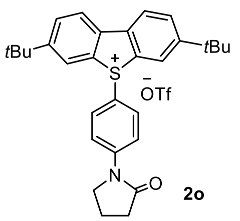

**2o**

**3,7-di-*tert*-butyl-5-(4-(2-oxopyrrolidin-1-yl)phenyl)-*5H*-dibenzo[*b,d*]thiophen-5-ium trifluoromethanesulfonate (2o)**

Following reaction condition A, **2o** was obtained as colorless solid (737 mg, 38% yield), purification by column chromatography on silica gel (DCM/$CH_3OH$: 40/1). $^1$H NMR (400 MHz, $CDCl_3$): δ = 8.07 (d, *J* = 1.7 Hz, 2H), 8.01 (d, *J* = 8.2 Hz, 2H), 7.91 – 7.86

(m, 2H), 7.84 (dd, $J$ = 8.3, 1.7 Hz, 2H), 7.72 – 7.67 (m, 2H), 3.90 (t, $J$ = 7.1 Hz, 2H), 2.60 (t, $J$ = 8.1 Hz, 2H), 2.24 – 2.10 (m, 2H), 1.34 (s, 18H); $^{13}$C NMR (101 MHz, $CDCl_3$): δ = 175.4 ($C_q$), 156.2 ($C_q$), 145.3 ($C_q$), 136.2 ($C_q$), 132.9 ($C_q$), 132.2 (CH), 131.8 (CH), 125.4 (CH), 123.2 (CH), 121.5 (CH), 121.0 (q, J = 321.2 Hz, $C_q$), 119.2 ($C_q$), 48.5 ($CH_2$), 36.0 ($C_q$), 32.9 ($CH_2$), 31.2 ($CH_3$), 17.9 ($CH_2$); $^{19}$F NMR (377 MHz, $CDCl_3$): δ = -78.08 (s). HRMS-ESI (m/z) calculated for $C_{30}H_{34}NOS^+$ [M-OTf]$^+$, 456.2356; found: 456.2356.

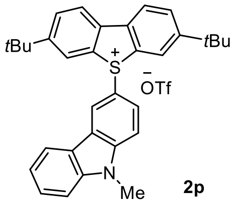


2p

**3,7-di-*tert*-butyl-5-(9-methyl-9H-carbazol-3-yl)-*5H*-dibenzo[*b,d*]thiophen-5-ium trifluoromethanesulfonate (2p)**

Following reaction condition A, **2p** was obtained as colorless solid (817 mg, 51% yield), purification by column chromatography on silica gel (DCM/$CH_3OH$: 40/1). $^1$H NMR (400 MHz, $CDCl_3$): δ = 8.89 (d, $J$ = 2.0 Hz, 1H), 8.22 – 8.12 (m, 3H), 8.03 (d, $J$ = 1.6 Hz, 2H), 7.85 (dd, $J$ = 8.3, 1.7 Hz, 2H), 7.46 (ddd, $J$ = 8.4, 7.2, 1.2 Hz, 1H), 7.31 (d, $J$ = 8.9 Hz, 1H), 7.28 – 7.20 (m, 2H), 6.99 (dd, $J$ = 8.8, 2.0 Hz, 1H), 3.71 (s, 3H), 1.27 (s, 18H); $^{13}$C NMR (101 MHz, $CDCl_3$): δ = 155.8 ($C_q$), 143.8 ($C_q$), 141.7 ($C_q$), 135.9 ($C_q$), 133.9 ($C_q$), 131.7 (CH), 127.8 (CH), 126.9 (CH), 125.7 (CH), 124.9 (CH), 124.4 ($C_q$), 123.6 (CH), 121.6 ($C_q$), 121.4 (CH), 121.2 (CH), 121.2 (q, J = 322.2 Hz, $C_q$), 112.1 (CH), 111.5 ($C_q$), 109.4 (CH), 35.9 ($C_q$), 31.2 ($CH_3$), 29.6 ($CH_3$); $^{19}$F NMR (377 MHz, $CDCl_3$): δ = -77.92 (s). HRMS-ESI (m/z) calculated for $C_{33}H_{34}NS^+$ [M-OTf]$^+$, 476.2406; found: 476.2408.

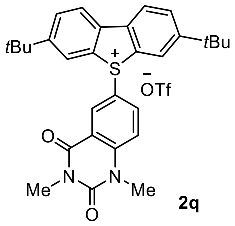


2q

**3,7-di-*tert*-butyl-5-(1,3-dimethyl-2,4-dioxo-1,2,3,4-tetrahydroquinazolin-6-yl)-*5H*-dibenzo[*b,d*]thiophen-5-ium trifluoromethanesulfonate (2q)**

Following reaction condition A, **2q** was obtained as colorless solid (1.15 g, 81% yield), purification by column chromatography on silica gel (DCM/$CH_3OH$: 30/1). $^1$H NMR (400 MHz, $CDCl_3$): δ = 8.98 (dd, $J$ = 9.0, 2.5 Hz, 1H), 8.14 (d, $J$ = 1.6 Hz, 2H), 8.02 (d, $J$ = 8.2 Hz, 2H), 7.87 (dd, $J$ = 8.3, 1.7 Hz, 2H), 7.70 (d, $J$ = 2.5 Hz, 1H), 7.63 (d, $J$ = 9.1 Hz, 1H), 3.65 (s, 3H), 3.37 (s, 3H), 1.34 (s, 18H); $^{13}$C NMR (101 MHz, $CDCl_3$): δ = 160.2 ($C_q$), 156.5 ($C_q$), 150.4 ($C_q$), 144.9 ($C_q$), 140.6 (CH), 136.3 ($C_q$), 132.3 ($C_q$), 132.2 (CH), 129.7 (CH), 125.5 (CH), 123.4 (CH), 120.9 (q, $J$ = 322.2 Hz, $C_q$), 120.4 ($C_q$), 117.8 ($C_q$), 117.4 (CH), 36.0 ($C_q$), 31.7 ($CH_3$), 31.2 ($CH_3$), 28.9 ($CH_3$); $^{19}$F NMR (377 MHz, $CDCl_3$): δ = -78.13 (s). HRMS-ESI (m/z) calculated for $C_{30}H_{33}N_2O_2S^+$ [M-OTf]$^+$, 485.2257; found: 485.2256.

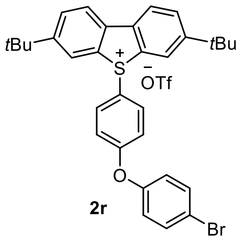

2r

**5-(4-(4-bromophenoxy)phenyl)-3,7-di-*tert*-butyl-*5H*-dibenzo[*b*,*d*]thiophen-5-ium trifluoromethanesulfonate (2r)**

Following reported procedure[4], **2r** was obtained as colorless solid (1.10 g, 83% yield), purification by column chromatography on silica gel (DCM/$CH_3OH$: 50/1). $^1H$ NMR (400 MHz, $CDCl_3$): δ = 8.12 (d, *J* = 1.6 Hz, 2H), 8.01 (d, *J* = 8.2 Hz, 2H), 7.84 (dd, *J* = 8.3, 1.7 Hz, 2H), 7.71 – 7.63 (m, 2H), 7.54 – 7.46 (m, 2H), 7.06 – 7.00 (m, 2H), 6.98 – 6.90 (m, 2H), 1.35 (s, 18H); $^{13}C$ NMR (101 MHz, $CDCl_3$): δ = 163.1 ($C_q$), 156.1 ($C_q$), 153.1 ($C_q$), 136.0 ($C_q$), 133.4 (CH), 133.4 (CH), 132.8 ($C_q$), 131.7 (CH), 125.3 (CH), 123.0 (CH), 122.5 (CH), 120.8 (q, J = 322.2 Hz, $C_q$), 119.5 (CH), 118.6 ($C_q$), 118.3 ($C_q$), 35.8 ($C_q$), 31.0 ($CH_3$); $^{19}F$ NMR (377 MHz, $CDCl_3$): δ = -78.13 (s). HRMS-ESI (m/z) calculated for $C_{32}H_{32}BrOS^+$ [M-OTf]$^+$, 543.1352; found: 543.1349.

## 3. Optimization of the Reaction Conditions

General procedure for the optimization of the reaction: to an oven-dried screw-cap pressure tube, ruthenium catalyst, arylsulfonium salt, and base were added. The tube was then moved to glovebox, and charged with pentafluorobenzene and pivalonitrile which were completely degassed and stored in glovebox. Next, the screw tube was taken out of the glovebox, and heated at oil bath for the corresponding time. After the reaction time point, the reaction mixture was taken out of the oil bath, and cooled to room temperature. The reaction mixture was added 0.1 mmol 1,3,5-trimethoxybenzene as internal standard, then the mixture was diluted with dichloromethane and filtered through celite gel. The homogenous solution was concentrated under reduced pressure, delivering the crude product that was tested using $^{1}$H NMR to assay the yield.

### 3.1 The screening of arylsulfonium salts

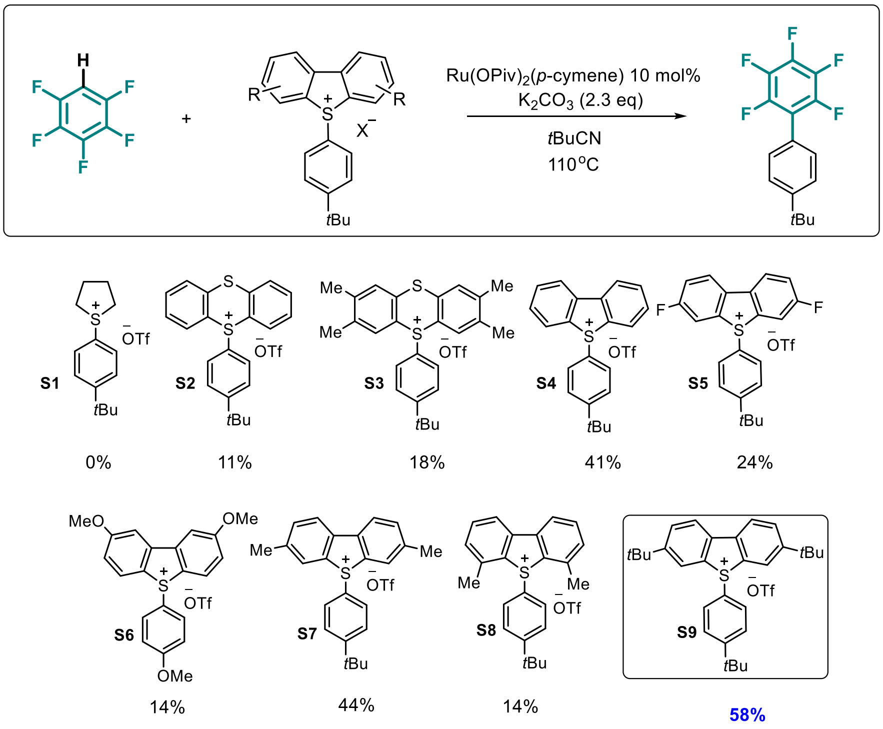


The reactions were performed under nitrogen at 110°C for 20 h with arylsulfonium salts (0.1 mmol, 1 equiv), pentafluorobenzene (0.5 mmol, 5 equiv), $Ru(OPiv)_2$(*p*-cymene) (10 mol%), $K_2CO_3$ (0.23 mmol, 2.3 equiv), tBuCN ( 55 uL).

**Table S1.** the screening of arylsulfonium salts

## 3.2 Examination of other parameters

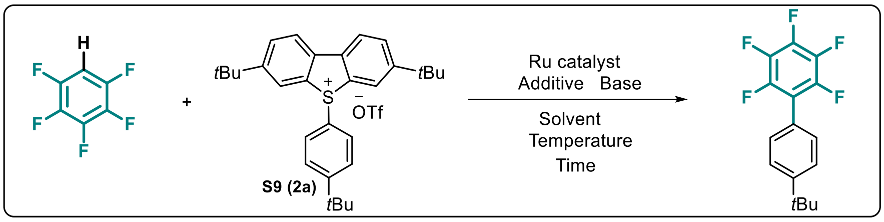


| Entry[a] | Ru catalyst | Base | Additive | Solvent | Temperature | Time | yield |
|---|---|---|---|---|---|---|---|
| 1 | $Ru(OPiv)_2$(*p*-cymene) | $K_2CO_3$ | 4F-$C_6H_4$COOH | *t*BuCN | 110°C | 20 h | 58% |
| 2 | $Ru(OPiv)_2$(*p*-cymene) | $K_2CO_3$ | KOPiv | *t*BuCN | 110°C | 20 h | 48% |
| 3 | $Ru(OPiv)_2$(*p*-cymene) | $K_2CO_3$ | AgOPiv | *t*BuCN | 110°C | 20 h | trace |
| 4 | $Ru(OPiv)_2$(*p*-cymene) | $K_2CO_3$ | PivOH | *t*BuCN | 110°C | 20 h | 64% |
| 5 | $Ru(OPiv)_2$(*p*-cymene) | $K_2CO_3$ | -- | *t*BuCN | 110°C | 20 h | 58% |
| 6 | $Ru(OPiv)_2$(*p*-cymene) | $K_2CO_3$ | KI | *t*BuCN | 110°C | 20 h | 50% |
| 7 | $Ru(OPiv)_2$(*p*-cymene) | $K_2CO_3$ | $PhPO(OH)_2$ | *t*BuCN | 110°C | 20 h | 55% |
| 8 | $Ru(OPiv)_2$(*p*-cymene) | $K_2CO_3$ | $(4CF_3\text{-}C_6H_4)_3P$ | *t*BuCN | 110°C | 20 h | 0 |
| 9 | $Ru(OPiv)_2$(*p*-cymene) | $K_2CO_3$ | PivOH | *t*BuCN | 120°C | 20 h | 82% |
| 10[b] | $Ru(OPiv)_2$(*p*-cymene) | $K_2CO_3$ | PivOH | *t*BuCN | 120°C | 24 h | 92% (85%) |
| 11 | $Ru(OAc)_2$(*p*-cymene) | $K_2CO_3$ | PivOH | *t*BuCN | 120°C | 20 h | 62% |
| 12 | $Ru(AdCOO)_2$(*p*-cymene) | $K_2CO_3$ | PivOH | *t*BuCN | 120°C | 20 h | 68% |
| 13 | $Ru(MesCOO)_2$(*p*-cymene) | $K_2CO_3$ | PivOH | *t*BuCN | 120°C | 20 h | 57% |
| 14 | $RuCl_3$ | $K_2CO_3$ | PivOH | *t*BuCN | 120°C | 20 h | no product |
| 15 | $Ru(OPiv)_2$(*p*-cymene) | KOAc | PivOH | *t*BuCN | 120°C | 24 h | 12% |
| 16 | $Ru(OPiv)_2$(*p*-cymene) | KOPiv | PivOH | *t*BuCN | 120°C | 24 h | 17% |
| 17 | $Ru(OPiv)_2$(*p*-cymene) | $Cs_2CO_3$ | PivOH | *t*BuCN | 120°C | 24 h | 16% |
| 18 | $Ru(OPiv)_2$(*p*-cymene) | $KO^tBu$ | PivOH | *t*BuCN | 120°C | 24 h | 10% |
| 19 | $Ru(OPiv)_2$(*p*-cymene) | $K_2CO_3$ | PivOH | toluene | 120°C | 24 h | 0 |
| 20 | $Ru(OPiv)_2$(*p*-cymene) | $K_2CO_3$ | PivOH | DMA | 120°C | 24 h | 11% |
| 21 | $Ru(OPiv)_2$(*p*-cymene) | $K_2CO_3$ | PivOH | NMP | 120°C | 24 h | trace |
| 22 | $Ru(OPiv)_2$(*p*-cymene) | $K_2CO_3$ | PivOH | dioxane | 120°C | 24 h | 6% |

[a] Unless otherwise specified, the reactions were performed under nitrogen with arylsulfonium salt (0.1 mmol, 1 equiv), pentafluorobenzene (0.5 mmol, 5 equiv), Ru catalyst (10 mol%), additive (30 mol%), base (0.23 mmol, 2.3 equiv), *t*BuCN ( 55 uL). [b] with pentafluorobenzene (1.0 mmol, 10 equiv), *t*BuCN (110 uL), the yield of isolated product given within parentheses.

**Table S2.** examination of other parameters

## 4. General Procedure for C–H Arylation and Product Characterization

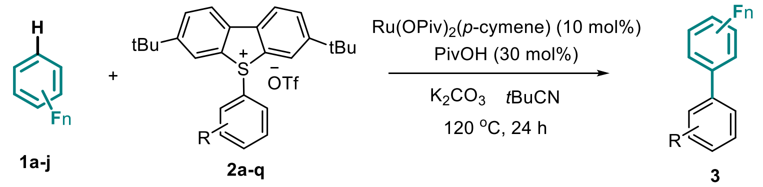


General procedure: to an oven-dried screw-cap pressure tube, $Ru(OPiv)_2$(*p*-cymene) (8.8 mg, 10 mol%), PivOH (6.12 mg, 30 mol%), arylsulfonium salt (0.2 mmol, 1 equiv), and $K_2CO_3$ (63.6 mg, 0.46 mmol, 2.3 equiv) were added. The screw-cap pressure tube was then moved to glovebox, and charged with polyfluoroarene or heteroarenes (2 mmol, 10 equiv) and pivalonitrile (220 µL) which were completely degassed and stored in glovebox. Next, the tube was taken out of the glovebox, and heated at oil bath for the corresponding time. After the reaction time point, the reaction mixture was taken out of the oil bath, and cooled to room temperature. The reaction mixture was diluted with dichloromethane and filtered through celite gel. The solution was concentrated under reduced pressure, then purified by column chromatography on silica gel.

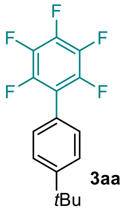


3aa

**4'-(*tert*-butyl)-2,3,4,5,6-pentafluoro-1,1'-biphenyl (3aa)**
The general procedure was followed using pentafluorobenzene (336.1 mg, 2 mmol) and 3,7-di-*tert*-butyl-5-(4-(*tert*-butyl)phenyl)-*5H*-dibenzo[*b,d*]thiophen-5-ium trifluoromethanesulfonate (**2a**) (115.8 mg, 0.2 mmol) at 120°C for 24 hours. Purification by column chromatography on silica gel (pentane) provided **3aa** (51.0 mg, 85%) as white solid. $^{1}$H NMR (400 MHz, $CDCl_3$): δ = 7.57 – 7.48 (m, 2H), 7.40 – 7.35 (m, 2H), 1.38 (s, 9H); $^{13}$C NMR (101 MHz, $CDCl_3$): δ = 152.6 ($C_q$), 144.34 (dm, $J$ = 247.2 Hz, $C_q$), 140.32 (dm, $J$ = 253.3 Hz, $C_q$), 138.0 (dm, $J$ = 253.5 Hz, $C_q$), 130.0 (CH), 125.9 (CH), 123.5 ($C_q$), 116.33 – 115.78 (m, $C_q$), 35.0 ($C_q$), 31.4 ($CH_3$); $^{19}$F NMR (377 MHz, $CDCl_3$): δ = -143.38 (dd, $J$ = 23.0, 8.2 Hz, 2F), -156.21 (t, $J$ = 20.9 Hz, 1F), -162.50 (ddd, $J$ = 22.9, 20.5, 8.1 Hz, 2F). IR (ATR): 2974, 1517, 1484, 1399, 1064, 982, 837 cm$^{-1}$. HRMS (EI): *m/z* calcd. for [M, $C_{16}H_{13}F_5$]$^{+}$ : 300.0932; found: 300.0932.

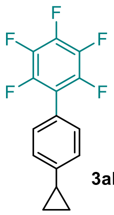


3ab

**4'-cyclopropyl-2,3,4,5,6-pentafluoro-1,1'-biphenyl (3ab)**
The general procedure was followed using pentafluorobenzene (336.1 mg, 2 mmol) and 3,7-di-*tert*-butyl-5-(4-cyclopropylphenyl)-*5H*-dibenzo[*b,d*]thiophen-5-ium trifluoro-

methanesulfonate (**2b**) (112.6 mg, 0.2 mmol) at 120°C for 24 hours. Purification by column chromatography on silica gel (pentane) provided **3ab** (45.5 mg, 80%) as white solid. $^{1}H$ NMR (400 MHz, $CDCl_3$): δ = 7.35 – 7.29 (m, 2H), 7.22 – 7.15 (m, 2H), 1.96 (tt, *J* = 8.4, 5.0 Hz, 1H), 1.07 – 1.01 (m, 2H), 0.80 – 0.75 (m, 2H); $^{13}C$ NMR (101 MHz, $CDCl_3$): δ= 145.8 ($C_q$), 144.3 (dm, *J* = 243.3 Hz, $C_q$), 140.3 (dm, *J* = 253.3 Hz, $C_q$ ), 138.0 (dm, *J* = 253.5 Hz, $C_q$), 130.2 (CH), 126.0 (CH), 123.3 ($C_q$), 116.45 - 115.64 (m, $C_q$), 15.5 (CH), 9.9 ($CH_2$); $^{19}F$ NMR (377 MHz, $CDCl_3$): δ = -143.42 (dd, *J* = 23.0, 8.1 Hz, 2F), -156.25 (t, *J* = 21.0 Hz, 1F), -162.38 – -162.78 (m, 2F). IR (ATR): 3016, 1515, 1485, 1409, 1041, 978, 821, 737 $cm^{-1}$. HRMS (EI): *m/z* calcd. for [M, $C_{15}H_9F_5$]$^+$ : 284.0619; found: 284.0617.

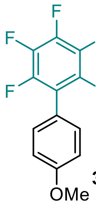

3ac

**2,3,4,5,6-pentafluoro-4'-methoxy-1,1'-biphenyl (3ac)**

The general procedure was followed using pentafluorobenzene (336.1 mg, 2 mmol) and 3,7-di-*tert*-butyl-5-(4-methoxyphenyl)-5*H*-dibenzo[*b*,*d*]thiophen-5-ium trifluoromethanesulfonate (**2c**) (110.5 mg, 0.2 mmol) at 120°C for 24 hours. Purification by column chromatography on silica gel (hexane/EtOAc: 100/1) provided **3ac** (48.2 mg, 88%) as white solid. $^{1}H$ NMR (300 MHz, $CDCl_3$): δ = 7.40 – 7.30 (m, 2H), 7.07 – 6.98 (m, 2H), 3.87 (s, 3H); $^{13}C$ NMR (75 MHz, $CDCl_3$): δ = 160.3 ($C_q$), 144.2 (dm, *J* = 242.5 Hz, $C_q$), 140.0 (dm, *J* = 252.9 Hz, $C_q$), 137.8 (dm, *J* = 251.3 Hz, $C_q$), 131.4 (CH), 118.4 ($C_q$), 116.20 – 115.15 (m, $C_q$), 114.2 (CH), 55.3 ($CH_3$); $^{19}F$ NMR (282 MHz, $CDCl_3$): δ = -143.65 (dd, *J* = 23.2, 8.1 Hz, 2F), -156.54 (t, *J* = 21.0 Hz, 1F), -162.23 - -162.94 (m, 2F). IR (ATR): 1608, 1516, 1486, 1259, 1065, 982, 737, 703 $cm^{-1}$. HRMS (EI): *m/z* calcd. for [M, $C_{13}H_7F_5O$]$^+$ : 274.0412; found: 274.0413.

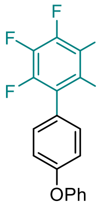

3ad

**2,3,4,5,6-pentafluoro-4'-phenoxy-1,1'-biphenyl (3ad)**

The general procedure was followed using pentafluorobenzene (336.1 mg, 2 mmol) and 3,7-di-*tert*-butyl-5-(4-phenoxyphenyl)-5*H*-dibenzo[*b*,*d*]thiophen-5-ium trifluoromethanesulfonate (**2d**) (123 mg, 0.2 mmol) at 120°C for 24 hours. Purification by column chromatography on silica gel (hexane) provided **3ad** (50.4 mg, 75%) as white solid. $^{1}H$ NMR (300 MHz, $CDCl_3$): δ = 7.45 – 7.34 (m, 4H), 7.22 – 7.15 (m, 1H), 7.13 – 7.06 (m, 4H); $^{13}C$ NMR (75 MHz, $CDCl_3$): δ= 158.7 ($C_q$), 156.3 ($C_q$), 144.3 (dm, *J* = 246.9 Hz, $C_q$), 140.4 (dm, *J* = 253.6 Hz, $C_q$), 138.0 (dm, *J* = 251.3 Hz, $C_q$), 131.8 (CH), 130.1 (CH), 124.3 (CH), 120.7 ($C_q$), 119.9 (CH), 118.4 (CH), 115.6 (td, *J* = 17.1, 3.9 Hz, $C_q$); $^{19}F$ NMR (282 MHz, $CDCl_3$): δ = -143.42 (dd, *J* = 23.1, 8.2 Hz, 2F), -155.91 (t, *J* = 21.0 Hz, 1F), -161.98 – -162.69 (m, 2F). IR (ATR): 2924, 1583, 1481, 1242, 1062, 978, 831, 747, 697 $cm^{-1}$. HRMS (EI): *m/z* calcd. for [M, $C_{18}H_9F_5O$]$^+$ : 336.0568; found: 336.0566.

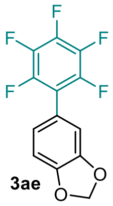

3ae

**5-(perfluorophenyl)benzo[*d*][1,3]dioxole (3ae)**

The general procedure was followed using pentafluorobenzene (336.1 mg, 2 mmol) and 5-(benzo[*d*][1,3]dioxol-5-yl)-3,7-di-*tert*-butyl-*5H*-dibenzo[*b,d*]thiophen-5-ium trifluoromethanesulfonate (**2e**) (113.3 mg, 0.2 mmol) at 120°C for 24 hours. Purification by column chromatography on silica gel (hexane) provided **3ae** (36.3 mg, 63%) as white solid. $^1$H NMR (300 MHz, $CDCl_3$): δ = 6.99 – 6.82 (m, 3H), 6.04 (s, 2H); $^{13}$C NMR (75 MHz, $CDCl_3$): δ= 148.6 ($C_q$), 148.1 ($C_q$), 144.4 (dm, $J$ = 247.2 Hz, $C_q$), 140.3 (dm, $J$ = 253.8 Hz, $C_q$), 138.0 (dm, $J$ = 249.8 Hz, $C_q$), 124.4 (CH), 119.6 ($C_q$), 116.17 – 115.36 (m, $C_q$ ), 110.5 (CH), 108.8 (CH), 101.7 ($CH_2$); $^{19}$F NMR (282 MHz, $CDCl_3$): δ = -143.19 (dd, $J$ = 23.2, 8.2 Hz, 2F), -156.02 (t, $J$ = 21.1 Hz, 1F), -162.05 – -162.87 (m, 2F). IR (ATR): 2917, 1530, 1490, 1444, 1244, 1045, 984, 929, 813 cm$^{-1}$. HRMS (EI): *m/z* calcd. for [M, $C_{13}H_5F_5O_2$]$^+$ : 288.0204; found: 288.0198.

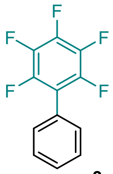

3af

**2,3,4,5,6-pentafluoro-1,1'-biphenyl (3af)**

The general procedure was followed using pentafluorobenzene (336.1 mg, 2 mmol) and 3,7-di-*tert*-butyl-5-phenyl-*5H*-dibenzo[*b,d*]thiophen-5-ium trifluoromethanesulfonate (**2f**) (104.5 mg, 0.2 mmol) at 120°C for 24 hours. Purification by column chromatography on silica gel (pentane) provided **3af** (39.6 mg, 81%) as white solid. $^1$H NMR (400 MHz, $CDCl_3$): δ = 7.53 – 7.45 (m, 3H), 7.45 – 7.40 (m, 2H); $^{13}$C NMR (101 MHz, $CDCl_3$): δ = 144.2 (dm, $J$ = 243.7 Hz, $C_q$), 140.5 (dm, $J$ = 240.3 Hz, $C_q$ ), 137.9 (dm, $J$ = 255.3 Hz, $C_q$), 130.2 (CH), 129.3 (CH), 128.8 (CH), 126.4 ($C_q$), 116.18 – 115.71 (m, $C_q$); $^{19}$F NMR (377 MHz, $CDCl_3$): δ = -143.24 (dd, $J$ = 22.9, 8.2 Hz, 2F), -155.62 (t, $J$ = 20.9 Hz, 1F), -161.91 – -162.60 (m, 2F). IR (ATR): 1651, 1527, 1494, 1438, 1067, 980, 745, 698 cm$^{-1}$. HRMS (EI): *m/z* calcd. for [M, $C_{12}H_5F_5$]$^+$ : 244.0306; found: 244.0312.

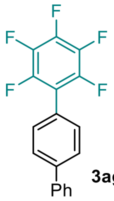

3ag

**2,3,4,5,6-pentafluoro-1,1':4',1''-terphenyl (3ag)**

The general procedure was followed using pentafluorobenzene (336.1 mg, 2 mmol) and 5-([1,1'-biphenyl]-4-yl)-3,7-di-*tert*-butyl-*5H*-dibenzo[*b,d*]thiophen-5-ium trifluoromethanesulfonate (**2g**) (120.0 mg, 0.2 mmol) at 120°C for 24 hours. Purification by column

chromatography on silica gel (pentane) provided **3ag** (51.8 mg, 81%) as white solid. $^{1}$H NMR (400 MHz, $CD_2Cl_2$): δ = 7.78 – 7.72 (m, 2H), 7.69 – 7.64 (m, 2H), 7.55 – 7.51 (m, 2H), 7.51 – 7.46 (m, 2H), 7.43 – 7.37 (m, 1H); $^{13}$C NMR (101 MHz, $CD_2Cl_2$): δ = 144.6 (dm, *J* = 246.7 Hz, $C_q$), 142.5 ($C_q$), 140.7 (dm, *J* = 267.6 Hz, $C_q$), 140.4 ($C_q$), 138.3 (d, *J* = 247.6 Hz, $C_q$), 130.9 (CH), 129.3 (CH), 128.2 (CH), 127.7 (CH), 127.5 (CH), 125.6 ($C_q$), 116.2 - 115.9 (m, $C_q$); $^{19}$F NMR (377 MHz, $CD_2Cl_2$): δ = -143.83 (dd, *J* = 23.0, 8.1 Hz, 2F), -156.52 (t, *J* = 20.8 Hz, 1F), -162.09 – -164.18 (m, 2F). IR (ATR): 1522, 1484, 1404, 1066, 976, 831, 757, 694 cm$^{-1}$. HRMS (EI): *m/z* calcd. for [M, $C_{18}H_9F_5$]$^+$ : 320.0619; found: 320.0616.

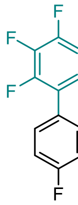
3ah

**2,3,4,4',5,6-hexafluoro-1,1'-biphenyl (3ah)**

The general procedure was followed using pentafluorobenzene (336.1 mg, 2 mmol) and 3,7-di-*tert*-butyl-5-(4-fluorophenyl)-*5H*-dibenzo[*b,d*]thiophen-5-ium trifluoromethan-esulfonate (**2h**) (108.0 mg, 0.2 mmol) at 120°C for 24 hours. Purification by column chromatography on silica gel (pentane) provided **3ah** (27.2 mg, 52%) as white solid. $^{1}$H NMR (400 MHz, $CDCl_3$): δ = 7.45 – 7.38 (m, 2H), 7.25 – 7.14 (m, 2H); $^{13}$C NMR (101 MHz, $CDCl_3$): δ = 163.3 (d, *J* = 249.9 Hz, $C_q$), 144.3 (dm, *J* = 247.7 Hz, $C_q$), 140.6 (dm, *J* = 254.2 Hz, $C_q$), 138.0 (dm, *J* = 253.2 Hz, $C_q$), 132.2 (d, *J* = 8.4 Hz, CH), 122.4 ($C_q$), 116.1 (d, *J* = 21.8 Hz, CH), 115.4 – 114.8 (m, $C_q$); $^{19}$F NMR (377 MHz, $CD_2Cl_2$): δ = -111.32 (ddd, *J* = 13.6, 8.5, 5.1 Hz, 1F), -143.35 (dd, *J* = 22.9, 8.2 Hz, 2F), -155.23 (t, *J* = 20.9 Hz, 1F), -160.59 – -163.32 (m, 2F). IR (ATR): 1601, 1497, 1231, 1066, 982, 841, 740 cm$^{-1}$. HRMS (EI): *m/z* calcd. for [M, $C_{12}H_4F_6$]$^+$ : 262.0212; found: 262.0211.

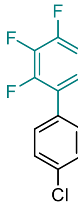
3ai

**4'-chloro-2,3,4,5,6-pentafluoro-1,1'-biphenyl (3ai)**

The general procedure was followed using pentafluorobenzene (336.1 mg, 2 mmol) and 5-(4-chlorophenyl)-*5H*-dibenzo[*b,d*]thiophen-5-ium trifluoromethanesulfonate (89.0 mg, 0.2 mmol) at 120°C for 24 hours. Purification by column chromatography on silica gel (pentane) provided **3ai** (25 mg, 45%) as white solid. $^{1}$H NMR (400 MHz, $CDCl_3$): δ = 7.51 - 7.45 (m, 2H), 7.39 - 7.34 (m, 2H); $^{13}$C NMR (101 MHz, $CDCl_3$): δ = 144.2 (dm, *J* = 248.0 Hz, $C_q$), 140.7 (dm, *J* = 254.7 Hz, $C_q$), 138.0 (dm, *J* = 253.3 Hz, $C_q$), 135.8 ($C_q$), 131.6 (CH), 129.2 (CH), 124.9 ($C_q$), 115.5 - 114.19 (m, $C_q$); $^{19}$F NMR (377 MHz, $CDCl_3$): δ = -143.15 (dd, *J* = 22.9, 8.2 Hz, 2F), -154.78 (t, *J* = 20.9 Hz, 1F), -161.36 – -162.74 (m, 2F). IR (ATR): 1531, 1486, 1394, 1066, 983, 832, 740 cm$^{-1}$. HRMS (EI): *m/z* calcd. for [M, $C_{12}H_4ClF_5$]$^+$ : 277.9916; found: 277.9919.

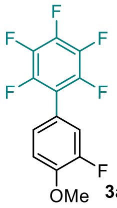
3aj

**2,3,3',4,5,6-hexafluoro-4'-methoxy-1,1'-biphenyl (3aj)**

The general procedure was followed using pentafluorobenzene (336.1 mg, 2 mmol) and 3,7-di-*tert*-butyl-5-(3-fluoro-4-methoxyphenyl)-*5H*-dibenzo[*b,d*]thiophen-5-ium trifluoromethanesulfonate (**2j**) (114.1 mg, 0.2 mmol) at 120°C for 24 hours. Purification by column chromatography on silica gel (Hexane/EtOAc : 50/1) provided **3aj** (40.9 mg, 70%) as white solid. $^{1}$H NMR (400 MHz, $CDCl_3$): δ = 7.23 - 7.13 (m, 2H), 7.09 - 7.05 (m, 1H), 3.95 (s, 3H); $^{13}$C NMR (101 MHz, $CDCl_3$): δ = 152.3 (d, *J* = 247.0 Hz, $C_q$), 148.7 (d, *J* = 10.4 Hz, $C_q$), 144.3 (dm, *J* = 247.8 Hz, $C_q$), 140.5 (dm, *J* = 251.5 Hz, $C_q$), 138.0 (dm, *J* = 249.6 Hz, $C_q$), 126.6 (d, *J* = 3.1 Hz, CH), 118.8 (d, *J* = 7.3 Hz, $C_q$), 118.1 (d, *J* = 20.0 Hz, CH), 115.1 – 114.4 (m, $C_q$), 113.5 (d, *J* = 2.5 Hz, CH), 56.4 ($CH_3$); $^{19}$F NMR (377 MHz, $CDCl_3$): δ = -134.02 – -134.71 (m, 1F), -143.35 (dd, *J* = 22.9, 8.2 Hz, 2F), -155.39 (t, *J* = 20.9 Hz, 1F), -161.65 – -162.42 (m, 2F). IR (ATR): 2957, 1517, 1488, 1272, 1060, 982, 820, 769 $cm^{-1}$. HRMS (EI): *m/z* calcd. for $[M, C_{13}H_6F_6O]^+$ : 292.0317; found: 292.0318.

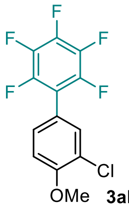
3ak

**3'-chloro-2,3,4,5,6-pentafluoro-4'-methoxy-1,1'-biphenyl (3ak)**

The general procedure was followed using pentafluorobenzene (336.1 mg, 2 mmol) and 3,7-di-*tert*-butyl-5-(3-chloro-4-methoxyphenyl)-*5H*-dibenzo[*b,d*]thiophen-5-ium trifluoromethanesulfonate (**2k**) (117.4 mg, 0.2 mmol) at 120°C for 24 hours. Purification by column chromatography on silica gel (Hexane/EtOAc : 50/1) provided **3ak** (48.1 mg, 78%) as white solid. $^{1}$H NMR (300 MHz, $CDCl_3$): δ = 7.46 (dt, *J* = 2.4, 1.4 Hz, 1H), 7.31 (ddt, *J* = 8.6, 2.5, 1.4 Hz, 1H), 7.04 (d, *J* = 8.6 Hz, 1H), 3.97 (s, 3H); $^{13}$C NMR (101 MHz, $CDCl_3$): δ = 155.8 ($C_q$), 144.2 (d, *J* = 247.4 Hz, $C_q$), 140.4 (d, *J* = 249.0 Hz, $C_q$), 137.9 (d, *J* = 252.9 Hz, $C_q$), 131.8 (CH), 129.8 (CH), 122.9 ($C_q$), 119.2 ($C_q$), 114.7 – 114.3 (m, $C_q$), 112.0 (CH), 56.3 ($CH_3$); $^{19}$F NMR (282 MHz, $CDCl_3$): δ = -142.55 – -143.93 (m, 2F), -155.32 (t, *J* = 21.0 Hz, 1F), -161.64 – -162.74 (m, 2F). IR (ATR): 2956, 1602, 1494, 1468, 1256, 1063, 985, 898, 695 $cm^{-1}$. HRMS (EI): *m/z* calcd. for $[M, C_{13}H_6ClF_5O]^+$ : 308.0022; found: 308.0023.

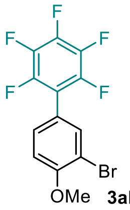
3al

**3'-bromo-2,3,4,5,6-pentafluoro-4'-methoxy-1,1'-biphenyl (3al)**

The general procedure was followed using pentafluorobenzene (336.1 mg, 2 mmol) and 5-(3-bromo-4-methoxyphenyl)-3,7-di-*tert*-butyl-*5H*-dibenzo[*b,d*]thiophen-5-ium trifluoromethanesulfonate (**2l**) (126.3 mg, 0.2 mmol) at 120°C for 24 hours. Purification by column chromatography on silica gel (Hexane/EtOAc : 50/1) provided **3al** (46.6 mg, 66%) as white solid. $^{1}$H NMR (300 MHz, $CDCl_3$): δ = 7.63 (dt, *J* = 2.5, 1.4 Hz, 1H), 7.36 (ddt, *J* = 8.6, 2.5, 1.4 Hz, 1H), 7.01 (d, *J* = 8.6 Hz, 1H), 3.96 (s, 3H); $^{13}$C NMR (101 MHz, $CDCl_3$): δ = 156.8 ($C_q$), 144.3 (dm, *J* = 247.6 Hz, $C_q$), 140.5 (dm, *J* = 253.2 Hz, $C_q$), 138.0 (dm, *J* = 254.1 Hz, $C_q$), 134.9 (CH), 130.6 (CH), 119.8 ($C_q$), 114.5 (td, *J* = 17.1, 4.2 Hz, $C_q$), 112.1 ($C_q$), 112.0 (CH), 56.5 ($CH_3$); $^{19}$F NMR (282 MHz, $CDCl_3$): δ = -143.22 (dd, *J* = 23.0, 8.2 Hz, 2F), -155.30 (t, *J* = 20.9 Hz, 1F), -161.33 – -163.04 (m, 2F). IR (ATR): 2920, 1598, 1492, 1466, 1454, 1258, 1060, 984, 890 $cm^{-1}$. HRMS (EI): *m/z* calcd. for $[M, C_{13}H_6BrF_5O]^+$ : 351.9517; found: 351.9519.

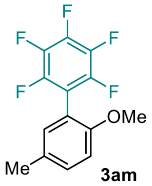
3am

**2,3,4,5,6-pentafluoro-2'-methoxy-5'-methyl-1,1'-biphenyl (3am)**

The general procedure was followed using pentafluorobenzene (336.1 mg, 2 mmol) and 3,7-di-*tert*-butyl-5-(2-methoxy-5-methylphenyl)-*5H*-dibenzo[*b,d*]thiophen-5-ium trifluoromethanesulfonate (**2m**) (113.3 mg, 0.2 mmol) at 120°C for 24 hours. Purification by column chromatography on silica gel (Hexane/EtOAc : 50/1) provided **3am** (29.4 mg, 51%) as white solid. $^{1}$H NMR (400 MHz, $CDCl_3$): δ = 7.27 – 7.23 (m, 1H), 7.03 (d, *J* = 2.2 Hz, 1H), 6.92 (d, *J* = 8.4 Hz, 1H), 3.78 (s, 3H), 2.34 (s, 3H); $^{13}$C NMR (101 MHz, $CDCl_3$): δ = 155.1 ($C_q$), 144.5 (dm, *J* = 246.9 Hz, $C_q$), 140.5 (dm, *J* = 252.5 Hz, $C_q$), 137.6 (dm, *J* = 251.9 Hz, $C_q$), 132.2 (CH), 131.5 (CH), 130.0 ($C_q$), 114.9 ($C_q$), 113.21 – 112.53 (m, $C_q$), 111.2 (CH), 55.8 ($CH_3$), 20.4 ($CH_3$); $^{19}$F NMR (377 MHz, $CDCl_3$): δ = -139.87 – -140.80 (m, 2F), -156.33 (t, *J* = 20.9 Hz, 1F), -162.73 – -163.88 (m, 2F). IR (ATR): 2924, 1516, 1497, 1255, 1065, 988, 807 $cm^{-1}$. HRMS (EI): *m/z* calcd. for $[M, C_{14}H_9F_5O]^+$ : 288.0568; found: 288.0565.

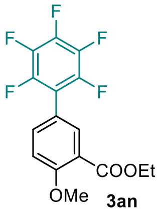
3an

**ethyl 2',3',4',5',6'-pentafluoro-4-methoxy-[1,1'-biphenyl]-3-carboxylate (3an)**

The general procedure was followed using pentafluorobenzene (336.1 mg, 2 mmol) and 3,7-di-*tert*-butyl-5-(3-(ethoxycarbonyl)-4-methoxyphenyl)-*5H*-dibenzo[*b,d*]thiophen-5-ium trifluoromethanesulfonate (**2n**) (125.0 mg, 0.2 mmol) at 120°C for 24 hours. Purification by column chromatography on silica gel (Hexane/EtOAc : 4/1) provided **3an** (51.9 mg, 75%) as white solid. $^{1}$H NMR (400 MHz, $CDCl_3$): δ = 7.86 (dt, *J* = 2.5, 1.3 Hz, 1H), 7.52 (ddt, *J* = 8.7, 2.6, 1.4 Hz, 1H), 7.09 (d, *J* = 8.7 Hz, 1H), 4.37 (q, *J* = 7.2 Hz, 2H), 3.96 (s, 3H), 1.38 (t, *J* = 7.1 Hz, 3H); $^{13}$C NMR (101 MHz, $CDCl_3$): δ = 165.5 ($C_q$), 159.8 ($C_q$), 144.3 (dm, *J* = 243.4 Hz, $C_q$), 140.5 (dm, *J* = 249.3 Hz, $C_q$), 138.0 (dm, *J* = 248.7 Hz, $C_q$), 135.1 (CH), 133.5 (CH), 121.0 ($C_q$), 118.2 ($C_q$), 114.9

(td, $J$ = 16.9, 3.8 Hz, $C_q$), 112.5 (CH), 61.3 ($CH_2$), 56.3 ($CH_3$), 14.4 ($CH_3$); $^{19}$F NMR (377 MHz, $CDCl_3$): δ = -143.33 (dd, $J$ = 23.0, 8.2 Hz, 2F), -155.54 (t, $J$ = 20.9 Hz, 1F), -162.00 – -162.22 (m, 2F). IR (ATR): 2983, 1730, 1495, 1240, 1190, 1072, 987, 828 $cm^{-1}$. HRMS (ESI): *m/z* calcd. for $[M+H, C_{16}H_{12}F_5O_3]^+$ : 347.0701; found: 347.0702.

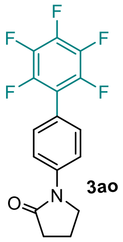

**1-(2',3',4',5',6'-pentafluoro-[1,1'-biphenyl]-4-yl)pyrrolidin-2-one (3ao)**

The general procedure was followed using pentafluorobenzene (336.1 mg, 2 mmol) and 3,7-di-*tert*-butyl-5-(4-(2-oxopyrrolidin-1-yl)phenyl)-*5H*-dibenzo[*b*,*d*]thiophen-5-ium trifluoromethanesulfonate (**2o**) (121.2 mg, 0.2 mmol) at 120°C for 24 hours. Purification by column chromatography on silica gel (Hexane/EtOAc : 3/1) provided **3ao** (33.3 mg, 51%) as white solid. $^1$H NMR (300 MHz, $CDCl_3$) δ 7.82 – 7.73 (m, 2H), 7.47 – 7.39 (m, 2H), 3.91 (t, $J$ = 7.0 Hz, 2H), 2.64 (t, $J$ = 8.1 Hz, 2H), 2.20 (p, $J$ = 7.5 Hz, 2H); $^{13}$C NMR (101 MHz, $CDCl_3$): δ = 174.6 ($C_q$), 144.3 (dm, $J$ = 243.5 Hz, $C_q$), 140.4 (dm, $J$ = 254.5 Hz, $C_q$), 140.4 ($C_q$), 137.9 (dm, $J$ = 248.0 Hz, $C_q$), 130.8 (CH), 122.1 ($C_q$), 119.7 (CH), 116.2 – 114.4 (m, $C_q$), 48.6 ($CH_2$), 32.9 ($CH_2$), 18.1 ($CH_2$); $^{19}$F NMR (282 MHz, $CDCl_3$): δ = -143.35 (dd, $J$ = 23.1, 8.1 Hz, 2F), -155.80 (t, $J$ = 21.0 Hz, 1F), -161.84 – -162.87 (m, 2F). IR (ATR): 2966, 1689, 1517, 1487, 1391, 1217, 980, 851 $cm^{-1}$. HRMS (ESI): *m/z* calcd. for $[M+H, C_{16}H_{11}F_5NO]^+$ : 328.0755; found: 328.0752.

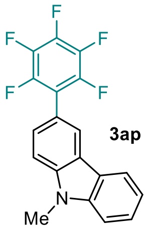

3ap

**9-methyl-3-(perfluorophenyl)-*9H*-carbazole (3ap)**

The general procedure was followed using pentafluorobenzene (336.1 mg, 2 mmol) and 3,7-di-*tert*-butyl-5-(9-methyl-*9H*-carbazol-3-yl)-*5H*-dibenzo[*b*,*d*]thiophen-5-ium trifluoromethanesulfonate (**2p**) (125.2 mg, 0.2 mmol) at 120°C for 24 hours. Purification by column chromatography on silica gel (Hexane/EtOAc : 20/1) provided **3ap** (36.1 mg, 52%) as white solid. $^1$H NMR (400 MHz, $CDCl_3$): δ = 8.18 – 8.14 (m, 1H), 8.11 (dt, $J$ = 7.7, 1.0 Hz, 1H), 7.57 – 7.47 (m, 3H), 7.44 (d, $J$ = 8.3 Hz, 1H), 7.29 (ddd, $J$ = 8.0, 7.1, 1.0 Hz, 1H), 3.88 (s, 3H); $^{13}$C NMR (101 MHz, $CDCl_3$): δ = 144.5 (dm, $J$ = 246.0 Hz, $C_q$), 141.5 ($C_q$), 141.2 ($C_q$), 140.0 (dm, $J$ = 253.5 Hz, $C_q$), 138.0 (dm, $J$ = 252.3 Hz, $C_q$), 127.6 (CH), 126.5 (CH), 123.2 ($C_q$), 122.6 ($C_q$), 122.5 (CH), 120.6 (CH), 119.6 (CH), 117.1 (td, $J$ = 17.4, 4.1 Hz, $C_q$), 116.5 ($C_q$), 108.9 (CH), 108.8 (CH), 29.3 ($CH_3$); $^{19}$F NMR (377 MHz, $CDCl_3$): δ = -143.51 (dd, $J$ = 23.4, 8.1 Hz, 2F), -156.87 (t, $J$ = 21.0 Hz, 1F), -162.58 – -162.77 (m, 2F). IR (ATR): 2932, 1600, 1526, 1498, 1252, 1059, 985, 745 $cm^{-1}$. HRMS (EI): *m/z* calcd. for $[M, C_{19}H_{10}F_5N]^+$ : 347.0728; found: 347.0723.

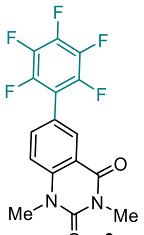

3aq

**1,3-dimethyl-6-(perfluorophenyl)quinazoline-2,4(*1H*,*3H*)-dione (3aq)**

The general procedure was followed using pentafluorobenzene (336.1 mg, 2 mmol) and 3,7-di-*tert*-butyl-5-(1,3-dimethyl-2,4-dioxo-1,2,3,4-tetrahydroquinazolin-6-yl)-*5H*-dibenzo[*b,d*]thiophen-5-ium trifluoromethanesulfonate (**2q**) (127.0 mg, 0.2 mmol) at 120°C for 24 hours. Purification by column chromatography on silica gel (Hexane/EtOAc : 4/1) provided **3aq** (33.5 mg, 47%) as white solid. $^{1}$H NMR (300 MHz, $CDCl_3$): δ = 8.32 (dt, *J* = 2.4, 1.3 Hz, 1H), 7.73 (ddt, *J* = 8.7, 2.5, 1.4 Hz, 1H), 7.34 (d, *J* = 8.7 Hz, 1H), 3.66 (s, 3H), 3.50 (s, 3H); $^{13}$C NMR (101 MHz, $CDCl_3$): δ = 161.5 ($C_q$), 151.2 ($C_q$), 144.4 (dm, *J* = 248.4 Hz, $C_q$), 140.9 (dm, *J* = 255.5 Hz, $C_q$), 141.0 ($C_q$), 138.1 (dm, *J* = 253.3 Hz, $C_q$), 136.5 (CH), 130.9 (CH), 121.3 (d, *J* = 2.0 Hz, $C_q$), 115.9 ($C_q$), 114.4 - 114.1 (m, $C_q$), 114.3 (CH), 31.1 ($CH_3$), 28.8 ($CH_3$); $^{19}$F NMR (282 MHz, $CDCl_3$): δ = -143.20 (dd, *J* = 22.8, 8.2 Hz, 2F), -154.29 (t, *J* = 20.9 Hz, 1F), -160.87 – -162.35 (m, 2F). IR (ATR): 1715, 1671, 1624, 1501, 1306, 1067, 986, 826 cm$^{-1}$. HRMS (EI): *m/z* calcd. for [M, $C_{16}H_9F_5N_2O_2$]$^+$ : 356.0579; found: 356.0581.

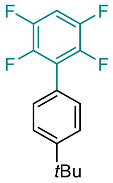

3ba

**4'-(*tert*-butyl)-2,3,5,6-tetrafluoro-1,1'-biphenyl (3ba)**

The general procedure was followed using 1,2,4,5-tetrafluorobenzene (300.1 mg, 2 mmol) and 3,7-di-*tert*-butyl-5-(4-(*tert*-butyl)phenyl)-*5H*-dibenzo[*b,d*]thiophen-5-ium trifluorome-thanesulfonate (**2a**) (115.8 mg, 0.2 mmol) at 120°C for 24 hours. Purification by column chromatography on silica gel (pentane) provided **3ba** (44.0 mg, 78%) as white solid. $^{1}$H NMR (400 MHz, $CDCl_3$): δ = 7.55 – 7.49 (m, 2H), 7.44 – 7.37 (m, 2H), 7.04 (tt, *J* = 9.7, 7.3 Hz, 1H), 1.38 (s, 9H); $^{13}$C NMR (101 MHz, $CDCl_3$): δ = 152.5 ($C_q$), 146.4 (dm, *J* = 251.8 Hz, $C_q$), 143.9 (dm, *J* = 247.5 Hz, $C_q$) 129.9 (t, *J* = 2.2 Hz, CH), 125.7 (CH), 124.8 – 124.5 (m, $C_q$), 121.6 (t, *J* = 16.5 Hz, $C_q$), 104.7 (t, *J* = 22.7 Hz, CH), 34.9 ($C_q$), 31.4 ($CH_3$); $^{19}$F NMR (377 MHz, $CDCl_3$): δ = -139.34 (ddd, *J* = 22.4, 12.8, 9.6 Hz, 2F), -143.97 (ddd, *J* = 22.2, 12.8, 7.3 Hz, 2F). IR (ATR): 2969, 1491, 1396, 1163, 925, 831, 699 cm$^{-1}$. HRMS (EI): *m/z* calcd. for [M, $C_{16}H_{14}F_4$]$^+$ : 282.1026; found: 282.1026.

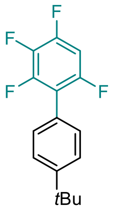

3ca

**4'-(*tert*-butyl)-2,3,4,6-tetrafluoro-1,1'-biphenyl (3ca)**

The general procedure was followed using 1,2,3,5-tetrafluorobenzene (300.1 mg, 2 mmol) and 3,7-di-*tert*-butyl-5-(4-(*tert*-butyl)phenyl)-5*H*-dibenzo[*b*,*d*]thiophen-5-ium trifluorome-thanesulfonate (**2a**) (115.8 mg, 0.2 mmol) at 120°C for 48 hours. Purification by column chromatography on silica gel (pentane) provided **3ca** (29.9 mg, 53%) as white solid. $^{1}$H NMR (400 MHz, $CDCl_3$): δ = 7.52 – 7.48 (m, 2H), 7.39 – 7.34 (m, 2H), 6.86 (dddd, *J* = 10.2, 9.5, 6.1, 2.4 Hz, 1H), 1.37 (s, 9H); $^{13}$C NMR (101 MHz, $CDCl_3$): δ = 154.3 (dm, *J* = 246.0 Hz, $C_q$), 151.9 ($C_q$), 149.6 (dm, *J* = 250.4 Hz, $C_q$), 149.0 (dm, *J* = 250.5 Hz, $C_q$), 137.5 (dm, *J* = 248.1 Hz, $C_q$ ), 129.8 (CH), 125.5 (CH), 124.5 ($C_q$), 116.48 – 115.12 (m, $C_q$), 100.9 (ddd, *J* = 29.3, 21.3, 4.0 Hz, CH), 34.8 ($C_q$), 31.3 ($CH_3$); $^{19}$F NMR (377 MHz, $CDCl_3$): δ = -118.13 (t, *J* = 10.2 Hz, 1F), -133.92 (ddd, *J* = 21.6, 10.2, 5.3 Hz, 1F), -135.51 (dd, *J* = 21.9, 5.0 Hz, 1F), -164.94 (tdd, *J* = 21.6, 10.9, 6.1 Hz, 1F). IR (ATR): 2963, 1639, 1503, 1457, 1145, 1057, 875, 835 $cm^{-1}$. HRMS (EI): *m/z* calcd. for $[M, C_{16}H_{14}F_4]^+$ : 282.1026; found: 282.1025.

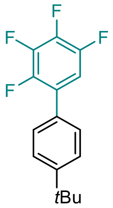

3da

**4'-(*tert*-butyl)-2,3,4,5-tetrafluoro-1,1'-biphenyl (3da)**

The general procedure was followed using 1,2,3,4-tetrafluorobenzene (300.1 mg, 2 mmol) and 3,7-di-*tert*-butyl-5-(4-(*tert*-butyl)phenyl)-5*H*-dibenzo[*b*,*d*]thiophen-5-ium trifluorome-thanesulfonate (**2a**) (115.8 mg, 0.2 mmol) at 120°C for 24 hours. Purification by column chromatography on silica gel (pentane) provided **3da** (21.4 mg, 38%) as white solid. $^{1}$H NMR (400 MHz, $CDCl_3$): δ = 7.52 – 7.47 (m, 2H), 7.45 – 7.41 (m, 2H), 7.06 (dddd, *J* = 10.7, 8.1, 6.5, 2.6 Hz, 1H), 1.37 (s, 9H); $^{13}$C NMR (101 MHz, $CDCl_3$): δ = 152.1 ($C_q$), 147.1 (dm, *J* = 246.5 Hz, $C_q$), 145.1 (dm, *J* = 247.5 Hz, $C_q$), 141.4 (dm, *J* = 253.5 Hz, $C_q$), 139.7 (dm, *J* = 253.5 Hz, $C_q$), 130.3 ($C_q$), 128.6 (d, *J* = 3.2 Hz, CH), 126.0 (CH), 125.68 – 125.20 (m, $C_q$), 111.3 (dt, *J* = 19.3, 3.2 Hz, CH), 34.9 ($C_q$), 31.4 ($CH_3$); $^{19}$F NMR (377 MHz, $CDCl_3$): δ = -139.29 – -140.34 (m, 1F), -143.61 – -144.08 (m, 1F), -155.38 (td, *J* = 20.1, 3.0 Hz, 1F), -157.24 – -158.11 (m, 1F). IR (ATR): 2964, 1619, 1507, 1475, 1265, 1078, 995, 835 $cm^{-1}$. HRMS (EI): *m/z* calcd. for $[M, C_{16}H_{14}F_4]^+$ : 282.1026; found: 282.1023.

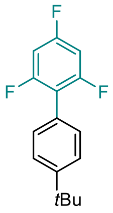

**3ea**

**4'-(*tert*-butyl)-2,4,6-trifluoro-1,1'-biphenyl (3ea)**

The general procedure was followed using 1,3,5-trifluorobenzene (264.2 mg, 2 mmol) and 3,7-di-*tert*-butyl-5-(4-(*tert*-butyl)phenyl)-5*H*-dibenzo[*b*,*d*]thiophen-5-ium trifluorome-thanesulfonate (**2a**) (115.8 mg, 0.2 mmol) at 130°C for 24 hours. Purification by column chromatography on silica gel (pentane) provided **3ea** (12.1 mg, 23%) as oil. $^1$H NMR (400 MHz, $CDCl_3$): δ = 7.51 – 7.45 (m, 2H), 7.39 – 7.33 (m, 2H), 6.80 – 6.70 (m, 2H), 1.36 (s, 9H); $^{13}$C NMR (101 MHz, $CDCl_3$): δ = 161.7 (dm, *J* = 250.5 Hz, $C_q$), 160.5 (dm, *J* = 249.5 Hz, $C_q$), 151.5 ($C_q$), 130.0 (CH), 125.5 (CH), 125.5 ($C_q$), 115.1 (dd, *J* = 23.9, 18.9 Hz, $C_q$), 100.6 (ddd, *J* = 29.7, 25.3, 1.9 Hz, CH), 34.8 ($C_q$), 31.4 ($CH_3$); $^{19}$F NMR (377 MHz, $CDCl_3$): δ = -109.63 (q, *J* = 8.5, 7.5 Hz, 1F), -111.32 (t, *J* = 6.9 Hz, 2F). IR (ATR): 2963, 2864, 1635, 1593, 1487, 1441, 1119, 1031, 839 cm$^{-1}$. HRMS (EI): *m/z* calcd. for [M, $C_{16}H_{15}F_3$]$^+$ : 264.1120; found: 264.1122.

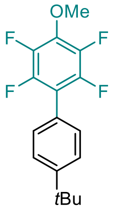

**3fa**

**4'-(*tert*-butyl)-2,3,5,6-tetrafluoro-4-methoxy-1,1'-biphenyl (3fa)**

The general procedure was followed using 1,2,4,5-tetrafluoro-3-methoxybenzene (360.2 mg, 2 mmol) and 3,7-di-*tert*-butyl-5-(4-(*tert*-butyl)phenyl)-5*H*-dibenzo[*b*,*d*]-thiophen-5-ium trifluorome-thanesulfonate (**2a**) (115.8 mg, 0.2 mmol) at 130°C for 24 hours. Purification by column chromatography on silica gel (hexane/EtOAc : 100/1) provided **3fa** (28.1 mg, 45%) as white solid. $^1$H NMR (400 MHz, $CDCl_3$): δ = 7.53 – 7.47 (m, 2H), 7.38 (dt, *J* = 8.4, 1.6 Hz, 2H), 4.12 (t, *J* = 1.3 Hz, 3H), 1.37 (s, 9H); $^{13}$C NMR (101 MHz, $CDCl_3$): δ = 152.1 ($C_q$), 144.5 (d, *J* = 245.8 Hz, $C_q$), 141.4 (d, *J* = 246.7 Hz, $C_q$), 137.7 – 137.0 (m, $C_q$), 130.0 (t, *J* = 2.2 Hz, CH), 125.7 (CH), 124.4 ($C_q$), 114.4 (t, *J* = 17.2 Hz, $C_q$), 62.3 ($CH_3$), 34.9 ($C_q$), 31.4 ($CH_3$); $^{19}$F NMR (377 MHz, $CDCl_3$): δ = -145.26 (dd, *J* = 22.2, 8.7 Hz, 2F), -158.40 (dd, *J* = 22.2, 8.7 Hz, 2F). IR (ATR): 2960, 1651, 1486, 1427, 1078, 977, 835 cm$^{-1}$. HRMS (EI): *m/z* calcd. for [M, $C_{17}H_{16}F_4O$]$^+$ : 312.1132; found: 312.1135.

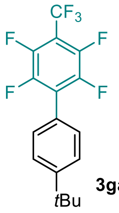

**3ga**

**4'-(*tert*-butyl)-2,3,5,6-tetrafluoro-4-(trifluoromethyl)-1,1'-biphenyl (3ga)**

The general procedure was followed using 1,2,4,5-tetrafluoro-3-(trifluoromethyl)benzene (436.1 mg, 2 mmol) and 3,7-di-*tert*-butyl-5-(4-(*tert*-butyl)phenyl)-*5H*-dibenzo[*b*,*d*]-thiophen-5-ium trifluorome-thanesulfonate (**2a**) (115.8 mg, 0.2 mmol) at 130°C for 24 hours. Purification by column chromatography on silica gel (pentane) provided **3ga** (56.0 mg, 80%) as white solid. $^{1}$H NMR (400 MHz, $CDCl_3$): δ = 7.59 – 7.51 (m, 2H), 7.45 – 7.37 (m, 2H), 1.38 (s, 9H); $^{13}$C NMR (101 MHz, $CDCl_3$): δ = 153.4 ($C_q$), 144.6 (dm, *J* = 258.5 Hz, $C_q$), 144.3 (dm, *J* = 243.3 Hz, $C_q$), 129.7 (t, *J* = 2.3 Hz, CH), 125.9 (CH), 124.9 (t, *J* = 16.1 Hz, $C_q$), 123.1 ($C_q$), 121.0 (dm, *J* = 274.5 Hz, $C_q$), 109.6 – 107.1 (m, $C_q$), 34.9 ($C_q$), 31.2 ($CH_3$); $^{19}$F NMR (377 MHz, $CDCl_3$): δ = -56.19 (t, *J* = 21.5 Hz, 3F), -140.78 – -141.20 (m, 2F), -141.58 – -141.81 (m, 2F). IR (ATR): 2966, 1655, 1484, 1337, 1147, 987, 837, 711 cm$^{-1}$. HRMS (EI): *m/z* calcd. for [M, $C_{17}H_{13}F_7$]$^+$ : 350.0900; found: 350.0899.

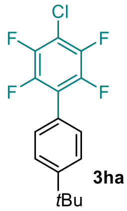


3ha

**4'-(*tert*-butyl)-4-chloro-2,3,5,6-tetrafluoro-1,1'-biphenyl (3ha)**

The general procedure was followed using 3-chloro-1,2,4,5-tetrafluorobenzene (369 mg, 2 mmol) and 3,7-di-*tert*-butyl-5-(4-(*tert*-butyl)phenyl)-*5H*-dibenzo[*b*,*d*]-thiophen-5-ium trifluorome-thanesulfonate (**2a**) (115.8 mg, 0.2 mmol) at 120°C for 24 hours. Purification by column chromatography on silica gel (pentane) provided **3ha** (52.5 mg, 83%) as white solid. $^{1}$H NMR (400 MHz, $CDCl_3$): δ = 7.58 – 7.48 (m, 2H), 7.40 (dt, *J* = 8.5, 1.7 Hz, 2H), 1.38 (s, 9H); $^{13}$C NMR (101 MHz, $CDCl_3$): δ = 152.8 ($C_q$), 144.6 (dm, *J* = 250.5 Hz, $C_q$), 144.2 (dm, *J* = 247.8 Hz, $C_q$), 129.9 (t, *J* = 2.3 Hz, CH), 125.9 (CH), 123.9 ($C_q$), 119.7 (t, *J* = 16.9 Hz, $C_q$), 111.70 – 109.52 (m, $C_q$), 35.0 ($C_q$), 31.4 ($CH_3$); $^{19}$F NMR (377 MHz, $CDCl_3$): δ = -141.40 – -141.61 (m, 2F), -142.57 – -142.79 (m, 2F). IR (ATR): 2964, 1484, 1405, 975, 832, 713 cm$^{-1}$. HRMS (EI): *m/z* calcd. for [M, $C_{16}H_{13}ClF_4$]$^+$ : 316.0636; found: 316.0634.

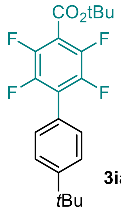


3ia

***tert*-butyl 4'-(*tert*-butyl)-2,3,5,6-tetrafluoro-[1,1'-biphenyl]-4-carboxylate (3ia)**

The general procedure was followed using *tert*-butyl 2,3,5,6-tetrafluorobenzoate (500.4 mg, 2 mmol) and 3,7-di-*tert*-butyl-5-(4-(*tert*-butyl)phenyl)-*5H*-dibenzo[*b*,*d*]-thiophen-5-ium trifluorome-thanesulfonate (**2a**) (115.8 mg, 0.2 mmol) at 120°C for 24 hours. Purification by column chromatography on silica gel (hexane/EtOAc : 10/1) provided **3ha** (61.9 mg, 81%) as white solid. $^{1}$H NMR (400 MHz, $CDCl_3$): δ = 7.56 – 7.49 (m, 2H), 7.41 (dt, *J* = 8.5, 1.7 Hz, 2H), 1.62 (s, 9H), 1.37 (s, 9H); $^{13}$C NMR (101 MHz, $CDCl_3$) δ 158.9 ($C_q$), 152.9 ($C_q$), 145.5 (dm, *J* = 74.5 Hz, $C_q$), 143.0 (dm, *J* = 69.0 Hz,

$C_q$), 129.9 (t, $J$ = 2.2 Hz, CH), 125.8 (CH), 124.0 ($C_q$), 123.0 (t, $J$ = 16.5 Hz, $C_q$), 113.2 (t, $J$ = 17.1 Hz, $C_q$), 84.7 ($C_q$), 35.0 ($C_q$), 31.4 ($CH_3$), 28.3 ($CH_3$); $^{19}$F NMR (377 MHz, $CDCl_3$): δ = -141.24 – -141.54 (m, 2F), -142.98 – -143.24 (m, 2F). IR (ATR): 2964, 1732, 1471, 1323, 1258, 1149, 984, 841 cm$^{-1}$. HRMS (EI): *m/z* calcd. for [M, $C_{21}H_{22}F_4O_2$]$^+$ : 382.1550; found: 382.1551.

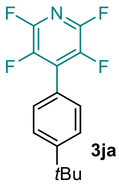


3ja

**4-(4-(*tert*-butyl)phenyl)-2,3,5,6-tetrafluoropyridine (3ja)**

The general procedure was followed using 2,3,5,6-tetrafluoropyridine (302.1 mg, 2 mmol) and 3,7-di-*tert*-butyl-5-(4-(*tert*-butyl)phenyl)-5*H*-dibenzo[*b*,*d*]-thiophen-5-ium trifluorome-thanesulfonate (**2a**) (115.8 mg, 0.2 mmol) at 130°C for 42 hours. Purification by column chromatography on silica gel (hexane) provided **3ja** (11.8 mg, 21%) as white solid. $^{1}$H NMR (400 MHz, $CDCl_3$): δ = 7.62 – 7.54 (m, 2H), 7.48 (dt, $J$ = 8.5, 1.7 Hz, 2H), 1.38 (s, 9H); $^{13}$C NMR (101 MHz, $CDCl_3$): δ = 154.1 ($C_q$), 144.1 (d, $J$ = 244.9 Hz, $C_q$), 139.4 (d, $J$ = 257.7 Hz, $C_q$), 134.4 – 132.6 (m, $C_q$), 129.6 (t, $J$ = 2.6 Hz, CH), 126.0 (CH), 123.0 ($C_q$), 35.0 ($C_q$), 31.2 ($CH_3$); $^{19}$F NMR (377 MHz, $CDCl_3$): δ = -89.90 – -93.06 (m, 2F), -144.84 – -146.39 (m, 2F). IR (ATR): 2972, 1638, 1450, 1402, 1152, 953, 828 cm$^{-1}$. HRMS (EI): *m/z* calcd. for [M, $C_{15}H_{13}F_4N$]$^+$ : 283.0979; found: 283.0980.

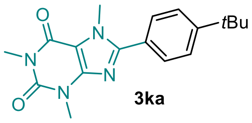


3ka

The general procedure was followed using caffeine (194 mg, 1.0 mmol), 3,7-di-*tert*-butyl-5-(4-(*tert*-butyl)phenyl)-5*H*-dibenzo[*b*,*d*]-thiophen-5-ium trifluoromethanesul-fonate (**2a**) (115.8 mg, 0.2 mmol) and *t*BuCN (0.3 mL) at 120°C for 24 hours. Purification by column chromatography on silica gel (hexane/EtOAc 1/1) provided **3ka** (24.8 mg, 38%) as gray solid. $^{1}$H NMR (400 MHz, $CDCl_3$) δ 7.66 – 7.58 (m, 2H), 7.56 – 7.50 (m, 2H), 4.06 (s, 3H), 3.63 (s, 3H), 3.44 (s, 3H), 1.36 (s, 9H); $^{13}$C NMR (101 MHz, $CDCl_3$) δ 155.8 ($C_q$), 154.0 ($C_q$), 152.5 ($C_q$), 151.9 ($C_q$), 148.5 ($C_q$), 129.0 (CH), 126.1 (CH), 125.6 ($C_q$), 108.6 ($C_q$), 35.1 ($C_q$), 34.0 ($CH_3$), 31.3 ($CH_3$), 29.9 ($CH_3$), 28.1 ($CH_3$). IR (ATR): 2959, 1702, 1660, 1541, 1434, 1230, 1032 cm$^{-1}$. HRMS (ESI): *m/z* calcd. for [M+H, $C_{18}H_{23}N_4O_2$]$^+$ : 327.1816; found: 327.1815.

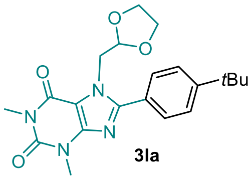


3la

The general procedure was followed using Doxofylline (266.3 mg, 1.0 mmol), 3,7-di-*tert*-butyl-5-(4-(*tert*-butyl)phenyl)-5*H*-dibenzo[*b*,*d*]-thiophen-5-ium trifluoromethane-sulfonate (**2a**) (115.8 mg, 0.2 mmol) and *t*BuCN (0.3 mL) at 120°C for 24 hours.

Purification by column chromatography on silica gel (hexane/EtOAc 1/1) provided **3la** (21.5 mg, 27%) as colorless solid. $^{1}$H NMR (300 MHz, $CDCl_3$) δ 7.73 – 7.63 (m, 2H), 7.57 – 7.47 (m, 2H), 5.35 (t, $J$ = 4.7 Hz, 1H), 4.52 (d, $J$ = 4.7 Hz, 2H), 3.94 – 3.78 (m, 4H), 3.63 (s, 3H), 3.44 (s, 3H), 1.36 (s, 9H); $^{13}$C NMR (75 MHz, $CDCl_3$) δ 155.5 ($C_q$), 153.9 ($C_q$), 153.5 ($C_q$), 151.9 ($C_q$), 148.6 ($C_q$), 129.5 (CH), 126.0 (CH), 125.9 ($C_q$), 108.2 ($C_q$), 102.0 (CH), 65.2 ($CH_2$), 48.4 ($CH_2$), 35.1 ($C_q$), 31.3 ($CH_3$), 30.0 ($CH_3$), 28.2 ($CH_3$). IR (ATR): 2957, 2922, 1702, 1661, 1540, 1460, 1434, 1033 cm$^{-1}$. HRMS (ESI): *m/z* calcd. for $[M+H, C_{21}H_{27}N_4O_4]^+$ : 399.2027; found: 399.2024.

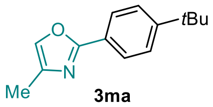


**3ma**

The general procedure was followed using 4-methyloxazole (83.1 mg, 1.0 mmol), 3,7-di-*tert*-butyl-5-(4-(*tert*-butyl)phenyl)-*5H*-dibenzo*[b,d]*-thiophen-5-ium trifluoromethanesulfonate (**2a**) (115.8 mg, 0.2 mmol) and *t*BuCN (0.3 mL) at 120°C for 24 hours. Purification by column chromatography on silica gel (hexane/EtOAc 5/1) provided **3ma** (21.5 mg, 20%) as viscous oil. $^{1}$H NMR (300 MHz, $CDCl_3$) δ 8.01 – 7.85 (m, 2H), 7.51 – 7.43 (m, 2H), 7.40 (q, $J$ = 1.3 Hz, 1H), 2.24 (d, $J$ = 1.3 Hz, 3H), 1.34 (s, 9H); $^{13}$C NMR (75 MHz, $CDCl_3$) δ 161.7 ($C_q$), 153.6 ($C_q$), 137.7 ($C_q$), 134.1 (CH), 126.2 (CH), 125.8 (CH), 125.1 ($C_q$), 35.0 ($C_q$), 31.3 ($CH_3$), 11.9 ($CH_3$). IR (ATR): 2963, 2926, 1703, 1666, 1495, 1269, 1112, 842 cm$^{-1}$. HRMS (ESI): *m/z* calcd. for $[M+H, C_{14}H_{18}NO]^+$ : 216.1383; found: 216.1383.

## 5. Late-Stage Incorporation of Pentafluorophenyl Motif

Flurbiprofen-derived 3,7-di-*tert*-butyldibenzothiophenium salt **LS4**

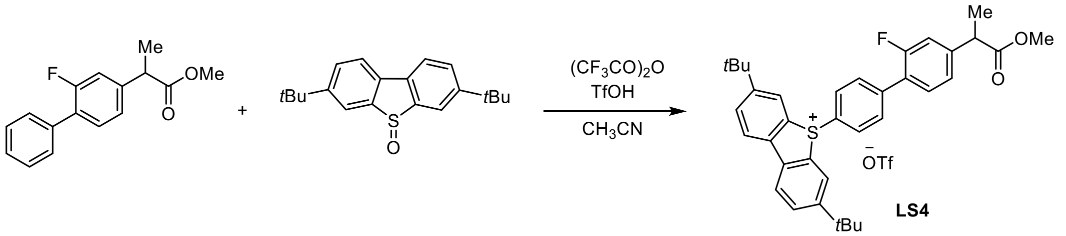


**LS4**

A dry 100 mL Schlenk flask equipped with a magnetic stir bar was charged with flurbiprofen methylester (440 mg, 1.70 mmol, 1 equiv.) and dry MeCN (8 mL) under nitrogen atmosphere at room temperature. After cooling to -40°C, $(CF_3CO)_2O$ (0.7 mL, 5.1 mmol, 3 equiv.) and TfOH (0.3 mL, 3.4 mmol, 2 equiv.) were added to the stirred reaction mixture. Subsequently, 3,7-di-*tert*-butyldibenzothiophene S-oxide (800 mg, 2.56 mmol, 1.5 equiv.) was added to the stirred reaction mixture in small portions over 3 minutes. After addition, the reaction mixture was stirred at -40°C for 1 h. Next, the Schlenk flask was taken out of the cold bath and warmed to 25°C in air. After stirring at 25°C for another 1 h, the reaction mixture was evaporated in vaccum to remove most of solvent, then the residue was diluted with DCM (50 mL) and poured onto saturated aqueous $NaHCO_3$ (20 mL). Then, the mixture was poured into a separatory funnel, and the layers were separated. The DCM layer was collected, and washed with aqueous NaOTf solution (15 mL x 2, 5 wt%), dried over anhydrous $Na_2SO_4$. After filtration, the mixture was concentrated to dryness under reduced pressure, the residue was purified by chromatography on silica gel ($DCM/CH_3OH$ : 50/1) to provide flurbiprofen-derived 3,7-di-*tert*-butyldibenzothiophenium salt **LS4** (954 mg, 1.36 mmol, 80% yield) as a colorless solid. $^1H$ NMR (400 MHz, $CDCl_3$): δ = 8.15 (d, *J* = 1.7 Hz, 2H), 8.05 (d, *J* = 8.3 Hz, 2H), 7.86 (dd, *J* = 8.3, 1.8 Hz, 2H), 7.81 – 7.75 (m, 2H), 7.73 – 7.66 (m, 2H), 7.38 (t, *J* = 8.1 Hz, 1H), 7.17 (dd, *J* = 8.1, 1.8 Hz, 1H), 7.12 (dd, *J* = 11.7, 1.8 Hz, 1H), 3.75 (q, *J* = 7.1 Hz, 1H), 3.68 (s, 3H), 1.51 (d, *J* = 7.2 Hz, 3H), 1.35 (s, 18H); $^{13}C$ NMR (101 MHz, $CDCl_3$): δ = 174.2 ($C_q$), 159.7 (d, *J* = 250.2 Hz, $C_q$), 156.3 ($C_q$), 144.1 (d, *J* = 7.8 Hz, $C_q$), 142.4 (d, *J* = 1.6 Hz, $C_q$), 136.5 ($C_q$), 132.4 ($C_q$), 132.0 (CH), 131.9 (CH), 131.1 (CH), 130.8 (d, *J* = 3.1 Hz, CH), 125.8 ($C_q$), 125.6 (CH), 125.1 (d, *J* = 12.7 Hz, $C_q$), 124.3 (d, *J* = 3.3 Hz, CH), 123.3 (CH), 121.0 (q, *J* = 322.2 Hz, Cq), 115.7 (d, *J* = 23.3 Hz, CH), 52.4 ($CH_3$), 45.1 (CH), 36.0 ($C_q$), 31.2 ($CH_3$), 18.5 ($CH_3$); $^{19}F$ NMR (377 MHz, $CDCl_3$): δ = -78.07 (s, 3F), -116.99 (dd, *J* = 11.7, 8.1 Hz, 1F). HRMS-ESI (m/z) calculated for $C_{36}H_{38}FO_2S^+$ $[M-OTf]^+$, 553.2571; found: 553.2571.

Atomoxetine-derived 3,7-di-*tert*-butyldibenzothiophenium salt **LS5**

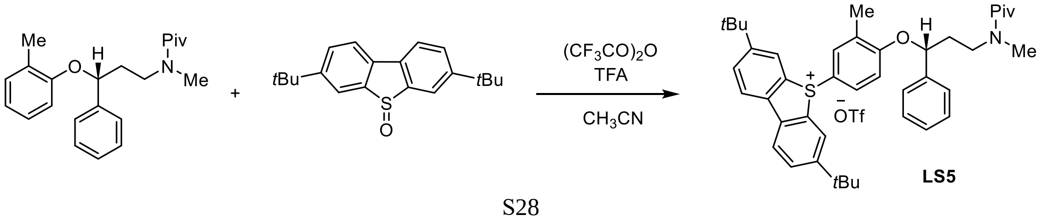


**LS5**

A dry 100 mL Schlenk flask equipped with a magnetic stir bar was charged with Atomoxetine-Piv (652 mg, 1.92 mmol, 1 equiv.) and dry MeCN (8 mL) under nitrogen atmosphere at room temperature. After cooling to -40°C, $(CF_3CO)_2O$ (0.8 mL, 5.76 mmol, 3 equiv.) and TFA (0.44 mL, 5.76 mmol, 3 equiv.) were added to the stirred reaction mixture. Subsequently, 3,7-di-*tert*-butyldibenzothiophene S-oxide (600 mg, 1.92 mmol, 1.0 equiv.) was added to the stirred reaction mixture in small portions over 3 minutes. After addition, the reaction mixture was stirred at -40°C for 1 h. Next, the Schlenk flask was taken out of the cold bath and warmed to 25°C in air. After stirring at 25°C for another 1 h, the reaction mixture was evaporated in vaccum to remove most of solvent, then the residue was diluted with DCM (50 mL) and poured onto saturated aqueous $NaHCO_3$ (20 mL). Then, the mixture was poured into a separatory funnel, and the layers were separated. The DCM layer was collected, and washed with aqueous NaOTf solution (15 mL x 2, 5 wt%), dried over anhydrous $Na_2SO_4$. After filtration, the mixture was concentrated to dryness under reduced pressure, the residue was purified by chromatography on silica gel ($DCM/CH_3OH$ : 40/1) to provide Atomoxetine-derived 3,7-di-*tert*-butyldibenzothiophenium salt **LS5** (1.35 g, 1.72 mmol, 90% yield) as a colorless solid. $^1H$ NMR (400 MHz, $CDCl_3$): δ = 8.06 – 7.97 (m, 3H), 7.88 (d, *J* = 1.6 Hz, 1H), 7.81 (td, *J* = 8.3, 1.8 Hz, 2H), 7.37 (dd, *J* = 2.6, 0.9 Hz, 1H), 7.34 – 7.16 (m, 6H), 6.76 (d, *J* = 8.9 Hz, 1H), 5.23 (dd, *J* = 8.3, 4.3 Hz, 1H), 3.63 – 3.38 (m, 2H), 3.02 (s, 3H), 2.30 (s, 3H), 2.28 – 2.18 (m, 1H), 2.15 – 2.04 (m, 1H), 1.31 (s, 9H), 1.30 (s, 9H), 1.20 (s, 9H); $^{13}C$ NMR (101 MHz, $CDCl_3$): δ = 177.5 ($C_q$), 161.5 ($C_q$), 155.9 ($C_q$), 155.8 ($C_q$), 139.9 ($C_q$), 136.1 ($C_q$), 135.9 ($C_q$), 132.9 ($C_q$), 132.8 ($C_q$), 132.6 (CH), 131.8 (CH), 131.7 (CH), 131.6 ($C_q$), 131.2 (CH), 129.1 (CH), 128.4 (CH), 125.7 (CH), 125.1 (CH), 124.7 (CH), 123.3 (CH), 123.2 (CH), 121.0 (q, *J* = 321.2 Hz, Cq), 115.1 (CH), 114.3 ($C_q$), 79.2 (CH), 47.3 ($CH_2$), 38.8 ($CH_2$), 36.7 ($CH_3$), 36.2 ($C_q$), 35.8 ($C_q$), 35.8 ($C_q$), 31.1 ($CH_3$), 31.1 ($CH_3$), 28.2 ($CH_3$), 16.8 ($CH_3$); $^{19}F$ NMR (377 MHz, $CDCl_3$): δ = -78.05 (s, 3F). HRMS-ESI (m/z) calculated for $C_{42}H_{52}NO_2S^+$ $[M-OTf]^+$, 634.3713; found: 634.3714.

Pyriproxyphen-derived 3,7-di-*tert*-butyldibenzothiophenium salt **LS6**

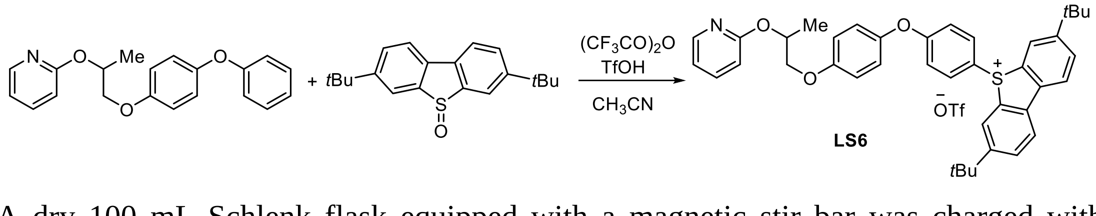


A dry 100 mL Schlenk flask equipped with a magnetic stir bar was charged with Pyriproxyphen (1.23 g, 3.84 mmol, 2 equiv.), 3,7-di-*tert*-butyldibenzothiophene S-oxide (600 mg, 1.92 mmol, 1.0 equiv.) and dry MeCN (8 mL) under nitrogen atmosphere at room temperature. After cooling to -40°C, $(CF_3CO)_2O$ (0.8 mL, 5.76 mmol, 3 equiv.) and TfOH (0.25 mL, 2.88 mmol, 1.5 equiv.) were added to the stirred reaction mixture. After addition, the reaction mixture was stirred at -40°C for 1 h. Next, the Schlenk flask was taken out of the cold bath and warmed to 25°C in air. After stirring at 25°C for another 4 h, the reaction mixture was evaporated in vaccum to remove most

of solvent, then the residue was diluted with DCM (50 mL) and poured onto saturated aqueous $NaHCO_3$ (20 mL). Then, the mixture was poured into a separatory funnel, and the layers were separated. The DCM layer was collected, and washed with aqueous NaOTf solution (15 mL x 2, 5 wt%), dried over anhydrous $Na_2SO_4$. After filtration, the mixture was concentrated to dryness under reduced pressure, the residue was purified by chromatography on silica gel (DCM/$CH_3OH$ : 30/1) to provide Pyriproxyphen-derived 3,7-di-*tert*-butyldibenzothiophenium salt **LS6** (735 mg, 0.96 mmol, 50% yield) as a colorless solid. $^1$H NMR (400 MHz, $CDCl_3$): δ = 8.18 – 8.10 (m, 3H), 7.97 (d, *J* = 8.2 Hz, 2H), 7.82 (dd, *J* = 8.2, 1.7 Hz, 2H), 7.68 – 7.61 (m, 2H), 7.57 (ddd, *J* = 8.4, 7.1, 2.0 Hz, 1H), 7.03 – 6.92 (m, 6H), 6.86 (ddd, *J* = 7.1, 5.1, 1.0 Hz, 1H), 6.74 (dt, *J* = 8.3, 0.9 Hz, 1H), 5.58 (dtd, *J* = 11.5, 6.4, 5.0 Hz, 1H), 4.20 (dd, *J* = 9.8, 5.3 Hz, 1H), 4.08 (dd, *J* = 9.8, 4.8 Hz, 1H), 1.48 (d, *J* = 6.4 Hz, 3H), 1.35 (s, 18H); $^{13}$C NMR (101 MHz, $CDCl_3$): δ = 164.6 ($C_q$), 163.2 ($C_q$), 156.9 ($C_q$), 156.2 ($C_q$), 147.4 ($C_q$), 146.9 (CH), 138.9 (CH), 136.1 ($C_q$), 133.5 (CH), 133.3 ($C_q$), 131.6 (CH), 125.6 (CH), 122.9 (CH), 122.1 (CH), 119.1 (CH), 117.1 ($C_q$), 117.0 (CH), 116.4 (CH), 111.8 (CH), 71.2 ($CH_2$), 69.3 (CH), 36.0 ($C_q$), 31.2 ($CH_3$), 17.1 ($CH_3$); $^{19}$F NMR (377 MHz, $CDCl_3$): δ = -78.17 (s, 3F). HRMS-ESI (m/z) calculated for $C_{40}H_{42}NO_3S^+$ $[M-OTf]^+$, 616.2880; found: 616.2887.

Clofibrate-derived 3,7-di-*tert*-butyldibenzothiophenium salt **LS7**

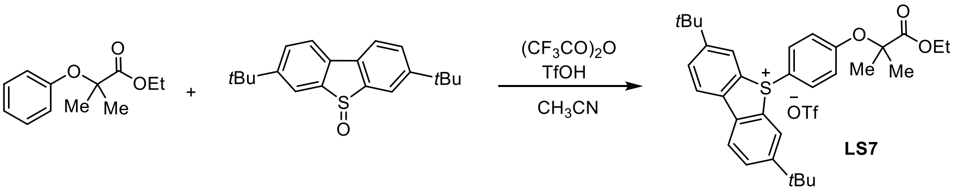


A dry 100 mL Schlenk flask equipped with a magnetic stir bar was charged with Clofibrate derivative (400 mg, 1.92 mmol, 1 equiv.) and dry MeCN (8 mL) under nitrogen atmosphere at room temperature. After cooling to -40°C, $(CF_3CO)_2O$ (0.66 mL, 4.8 mmol, 2.5 equiv.) and TfOH (0.35 mL, 3.84 mmol, 2 equiv.) were added to the stirred reaction mixture. Subsequently, 3,7-di-*tert*-butyldibenzothiophene S-oxide (600 mg, 1.92 mmol, 1.0 equiv.) was added to the stirred reaction mixture in small portions over 3 minutes. After addition, the reaction mixture was stirred at -40°C for 1 h. Next, the Schlenk flask was taken out of the cold bath and warmed to 25°C in air. After stirring at 25°C for another 1 h, the reaction mixture was evaporated in vaccum to remove most of solvent, then the residue was diluted with DCM (50 mL) and poured onto saturated aqueous $NaHCO_3$ (20 mL). Then, the mixture was poured into a separatory funnel, and the layers were separated. The DCM layer was collected, and washed with aqueous NaOTf solution (15 mL x 2, 5 wt%), dried over anhydrous $Na_2SO_4$. After filtration, the mixture was concentrated to dryness under reduced pressure, the residue was purified

by chromatography on silica gel (DCM/$CH_3OH$ : 50/1) to provide Clofibrate-derived 3,7-di-*tert*-butyldibenzothiophenium salt **LS7** (1.14 g, 1.74 mmol, 91% yield) as a colorless solid. $^1H$ NMR (400 MHz, $CDCl_3$): δ = 8.12 – 7.97 (m, 4H), 7.83 (dd, *J* = 8.3, 1.8 Hz, 2H), 7.63 – 7.55 (m, 2H), 7.00 – 6.79 (m, 2H), 4.20 (q, *J* = 7.1 Hz, 2H), 1.63 (s, 6H), 1.33 (s, 18H), 1.18 (t, *J* = 7.1 Hz, 3H); $^{13}C$ NMR (101 MHz, $CDCl_3$): δ = 172.8 ($C_q$), 161.3 ($C_q$), 156.0 ($C_q$), 136.1 ($C_q$), 133.0 ($C_q$), 132.9 (CH), 131.8 (CH), 125.3 (CH), 123.2 (CH), 121.0 (q, *J* = 321.2 Hz, $C_q$), 120.2 (CH), 116.2 ($C_q$), 80.2 ($C_q$), 62.1 ($CH_2$), 35.9 ($C_q$), 31.2 ($CH_3$), 25.5 ($CH_3$), 14.1 ($CH_3$); $^{19}F$ NMR (377 MHz, $CDCl_3$): δ = -78.12 (s, 3F). HRMS-ESI (m/z) calculated for $C_{32}H_{39}O_3S^+$ $[M-OTf]^+$, 503.2614; found: 503.2621.

Salicin-derived 3,7-di-*tert*-butyldibenzothiophenium salt **LS8**

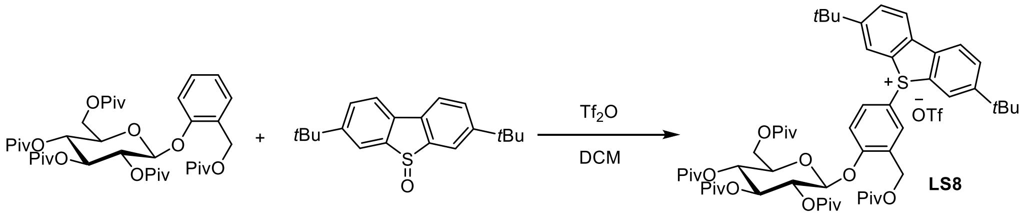


A dry 100 mL Schlenk flask equipped with a magnetic stir bar was charged with Salicin-Piv (1.36 g, 1.92 mmol, 1 equiv), 3,7-di-*tert*-butyldibenzothiophene S-oxide (660 mg, 2.11 mmol, 1.1 equiv), and dry DCM (8 mL) under nitrogen atmosphere at room temperature. After cooling to -40°C, $Tf_2O$ (0.39 mL, 2.30 mmol, 1.2 equiv) was added dropwise over 3 minutes. After addition, the reaction mixture was stirred at -40°C for 1 h. Next, the Schlenk flask was taken out of the cold bath and warmed to 25°C in air. After stirring at 25°C for another 1 h, the mixture was poured onto saturated aqueous $NaHCO_3$ (20 mL). Then, the mixture was poured into a separatory funnel, and the layers were separated. The DCM layer was collected, and washed with aqueous NaOTf solution (15 mL x 2 (5 wt%)), dried over anhydrous $Na_2SO_4$. After filtration, the mixture was concentrated to dryness under reduced pressure, the residue was purified by chromatography on silica gel (DCM/$CH_3OH$ : 50/1) to provide Salicin-derived 3,7-di-*tert*-butyldibenzothiophenium salt **LS8** (1.59 g, 1.38 mmol, 72% yield) as a colorless solid. $^1H$ NMR (400 MHz, $CDCl_3$): δ = 8.48 (dd, *J* = 8.9, 2.6 Hz, 1H), 8.19 (d, *J* = 1.7 Hz, 1H), 7.99 (t, *J* = 8.3 Hz, 2H), 7.92 (d, *J* = 1.7 Hz, 1H), 7.83 (td, *J* = 8.2, 1.7 Hz, 2H), 7.35 (d, *J* = 8.9 Hz, 1H), 6.71 (d, *J* = 2.5 Hz, 1H), 5.46 – 5.38 (m, 1H), 5.31 (q, *J* = 8.7, 8.1 Hz, 2H), 5.18 (dd, *J* = 10.1, 9.2 Hz, 1H), 4.92 – 4.80 (m, 2H), 4.24 (dd, *J* = 12.6, 1.7 Hz, 1H), 4.10 (dd, *J* = 12.6, 5.6 Hz, 1H), 4.01 (ddd, *J* = 10.1, 5.6, 1.7 Hz, 1H), 1.33 (s, 9H), 1.32 (s, 9H), 1.20 (s, 9H), 1.15 (s, 9H), 1.10 (s, 9H), 1.08 (s, 9H), 0.91 (s, 9H); $^{13}C$ NMR (101 MHz, $CDCl_3$): δ = 178.0 ($C_q$), 177.0 ($C_q$), 177.0 ($C_q$), 176.6 ($C_q$), 176.5 ($C_q$), 158.2 ($C_q$), 156.4 ($C_q$), 156.2 ($C_q$), 136.2 ($C_q$), 136.1 ($C_q$), 135.9 (CH), 132.7 ($C_q$), 132.6 ($C_q$), 132.0 (CH), 131.9 (CH), 130.9 ($C_q$), 126.4 (CH), 125.9 (CH), 124.9 (CH), 123.2 (CH), 123.0 (CH), 121.0 (q, *J* = 321.2 Hz, $C_q$), 118.9 ($C_q$), 116.1 (CH), 98.1 (CH), 73.1 (CH), 71.9 (CH), 70.7 (CH), 67.4 (CH), 61.7 ($CH_2$), 59.3 ($CH_2$),

39.0 ($C_q$), 38.9 ($C_q$), 38.8 ($C_q$), 38.7 ($C_q$), 36.0 ($C_q$), 35.9 ($C_q$), 31.1 ($CH_3$), 31.1 ($CH_3$), 27.2 ($CH_3$), 27.2 ($CH_3$), 27.1 ($CH_3$), 27.0 ($CH_3$) one $C_q$ resonance and one $CH_3$ resonance are missing due to overlap; $^{19}$F NMR (377 MHz, $CDCl_3$): δ = -78.09 (s, 3F). HRMS-ESI (m/z) calculated for $C_{58}H_{81}O_{12}S^+$ [M-OTf]$^+$, 1001.5443; found: 1001.5449.

Tocopherol-derived 3,7-di-*tert*-butyldibenzothiophenium salt **LS9**

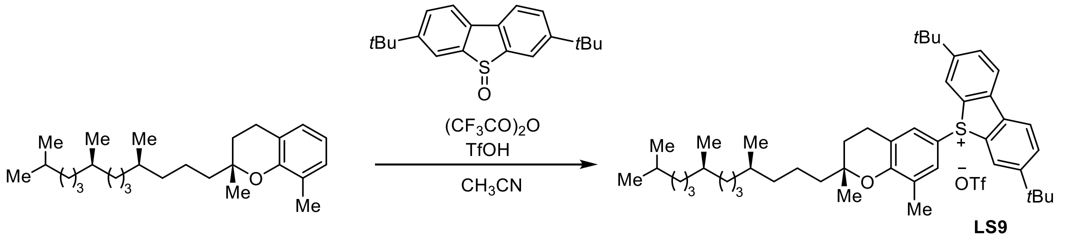


A dry 100 mL Schlenk flask equipped with a magnetic stir bar was charged with δ-Tocopherol derivative (743 mg, 1.92 mmol, 1 equiv.) and dry MeCN (8 mL) under nitrogen atmosphere at room temperature. After cooling to -40°C, $(CF_3CO)_2O$ (0.66 mL, 4.8 mmol, 2.5 equiv.) and TfOH (0.35 mL, 3.84 mmol, 2 equiv.) were added to the stirred reaction mixture. Subsequently, 3,7-di-*tert*-butyldibenzothiophene S-oxide (600 mg, 1.92 mmol, 1.0 equiv.) was added to the stirred reaction mixture in small portions over 3 minutes. After addition, the reaction mixture was stirred at -40°C for 1 h. Next, the Schlenk flask was taken out of the cold bath and warmed to 25°C in air. After stirring at 25°C for another 1 h, the reaction mixture was evaporated in vacuum to remove most of solvent, then the residue was diluted with DCM (50 mL) and poured onto saturated aqueous $NaHCO_3$ (20 mL). Then, the mixture was poured into a separatory funnel, and the layers were separated. The DCM layer was collected, and washed with aqueous NaOTf solution (15 mL x 2, 5 wt%), dried over anhydrous $Na_2SO_4$. After filtration, the mixture was concentrated to dryness under reduced pressure, the residue was purified by chromatography on silica gel ($DCM/CH_3OH$ : 50/1) to provide Tocopherol-derived 3,7-di-*tert*-butyldibenzothiophenium salt **LS9** (1.35 g, 1.63 mmol, 85% yield) as a colorless solid. $^1$H NMR (400 MHz, $CDCl_3$): δ = 8.04 (d, *J* = 8.3 Hz, 2H), 7.99 (dd, *J* = 4.1, 1.7 Hz, 2H), 7.83 (dd, *J* = 8.2, 1.8 Hz, 2H), 7.66 (d, *J* = 2.5 Hz, 1H), 6.79 (d, *J* = 2.6 Hz, 1H), 2.82 (t, *J* = 7.4 Hz, 2H), 2.04 (s, 3H), 1.88 – 1.71 (m, 2H), 1.57 (q, *J* = 8.4, 8.0 Hz, 2H), 1.48 (dt, *J* = 13.2, 6.6 Hz, 1H), 1.40 – 1.01 (m, 39 H), 0.85 – 0.81 (m, 12H);$^{13}$C NMR (101 MHz, $CDCl_3$): δ = 158.6 ($C_q$), 155.7 ($C_q$), 136.0 (d, *J* = 2.1 Hz, $C_q$), 133.2 (d, *J* = 3.2 Hz, $C_q$), 132.7 (CH), 131.6 (CH), 131.0 ($C_q$), 128.7 (CH), 125.0 (d, *J* = 3.6 Hz, CH), 124.1 ($C_q$), 123.2 (CH), 121.1 (q, *J* = 321.2 Hz, $C_q$), 111.8 ($C_q$), 79.0 ($C_q$), 40.7 ($CH_2$), 39.5 ($CH_2$), 37.5 ($CH_2$), 37.4 ($CH_2$), 37.4 ($CH_2$), 35.9 ($C_q$), 32.9 ($CH_3$), 32.8 ($CH_3$), 31.2 ($CH_3$), 30.2 ($CH_2$), 28.1 (CH), 24.9 ($CH_2$), 24.5 ($CH_2$), 24.4 ($CH_3$), 22.8 (CH), 22.7 (CH), 22.1 ($CH_2$), 21.0 ($CH_2$), 19.8 ($CH_3$), 19.7 ($CH_3$), 16.4 ($CH_3$) one $CH_2$ resonance is missing due to overlap; $^{19}$F NMR (377 MHz, $CDCl_3$): δ = -78.06 (s, 3F). HRMS-ESI (m/z) calculated for $C_{47}H_{69}OS^+$ [M-OTf]$^+$, 681.5064; found: 681.5064.

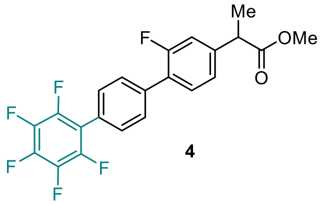
**4**

**methyl 2-(2,2'',3'',4'',5'',6''-hexafluoro-[1,1':4',1''-terphenyl]-4-yl)propanoate (4)**

The general procedure was followed using pentafluorobenzene (336.1 mg, 2 mmol) and **LS4** (140.5 mg, 0.2 mmol) at 120°C for 24 hours. Purification by column chromatography on silica gel (hexane) provided **4** (42.4 mg, 50%) as white solid. $^{1}$H NMR (400 MHz, $CDCl_3$): δ = 7.67 (dt, *J* = 8.4, 1.7 Hz, 2H), 7.53 – 7.49 (m, 2H), 7.45 (t, *J* = 8.1 Hz, 1H), 7.22 – 7.13 (m, 2H), 3.79 (q, *J* = 7.2 Hz, 1H), 3.72 (s, 3H), 1.56 (d, *J* = 7.2 Hz, 3H); $^{13}$C NMR (101 MHz, $CDCl_3$): δ = 174.5 ($C_q$), 159.9 (d, *J* = 249.1 Hz, $C_q$), 144.4 (dm, *J* = 248.1 Hz, $C_q$), 142.6 (d, *J* = 7.7 Hz, $C_q$), 140.6 (dm, *J* = 248.9 Hz, $C_q$), 138.1 (dm, *J* = 255.7 Hz, $C_q$), 136.7 ($C_q$), 130.9 (d, *J* = 3.7 Hz, CH), 130.4 (CH), 129.4 (d, *J* = 3.2 Hz, CH), 127.0 (d, *J* = 13.3 Hz, $C_q$), 125.8 ($C_q$), 123.9 (d, *J* = 3.3 Hz, CH), 115.9 - 115.4 (m, $C_q$), 115.6 (d, *J* = 23.5 Hz, CH), 52.4 ($CH_3$), 45.1 (d, *J* = 1.5 Hz, CH), 18.6 ($CH_3$); $^{19}$F NMR (377 MHz, $CDCl_3$): δ = -117.26 (dd, *J* = 11.5, 8.2 Hz, 1F), -143.06 (dd, *J* = 23.0, 8.2 Hz, 2F), -155.33 (t, *J* = 20.9 Hz, 1F), -161.21 – -163.13 (m, 2F). IR (ATR): 2958, 1738, 1487, 1399, 1172, 1065, 980, 822 $cm^{-1}$. HRMS (ESI): *m/z* calcd. for $[M+Na, C_{22}H_{14}F_6NaO_2]^+$ : 447.0790; found: 447.0782.

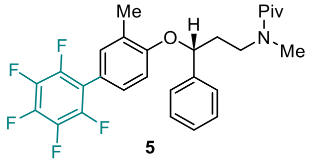
**5**

**(*R*)-*N*-methyl-*N*-(3-((2',3',4',5',6'-pentafluoro-3-methyl-[1,1'-biphenyl]-4-yl)oxy)-3-phenylpropyl)pivalamide (5)**

The general procedure was followed using pentafluorobenzene (336.1 mg, 2 mmol) and **LS5** (157 mg, 0.2 mmol) at 120°C for 24 hours. Purification by column chromatography on silica gel (hexane/EtOAc : 3/1) provided **5** (89.8 mg, 89%) as oil. $^{1}$H NMR (400 MHz, $CDCl_3$): δ = 7.38 – 7.27 (m, 5H), 7.19 (s, 1H), 7.02 (d, *J* = 8.5 Hz, 1H), 6.68 (d, *J* = 8.5 Hz, 1H), 5.24 (dd, *J* = 8.3, 4.3 Hz, 1H), 3.67 (ddt, *J* = 14.5, 10.1, 5.0 Hz, 1H), 3.54 (ddd, *J* = 13.5, 9.9, 5.9 Hz, 1H), 3.04 (s, 3H), 2.40 (s, 3H), 2.28 – 2.15 (m, 2H), 1.26 (s, 9H); $^{13}$C NMR (101 MHz, $CDCl_3$): δ = 177.5 ($C_q$), 156.7 ($C_q$), 144.3 (d, *J* = 246.6 Hz, $C_q$), 141.2 ($C_q$), 140.0 (dm, *J* = 248.5 Hz, $C_q$), 137.9 (d, *J* = 252.3 Hz, $C_q$), 132.4 (CH), 129.0 (CH), 128.8 (CH), 128.0 (CH), 127.6 ($C_q$), 125.7 (CH), 118.1 ($C_q$), 116.3 – 115.4 (m, $C_q$), 112.7 (CH), 78.1 (CH), 47.7 ($CH_2$), 38.9 ($CH_2$), 36.8 ($C_q$), 28.3 ($CH_3$), 27.3 ($CH_3$), 16.8 ($CH_3$); $^{19}$F NMR (377 MHz, $CDCl_3$): δ = -143.55 (dd, *J* = 23.3, 8.1 Hz, 2F), -156.76 (t, *J* = 21.0 Hz, 1F), -162.68 (td, *J* = 22.3, 8.1 Hz, 2F). IR (ATR): 2981, 2926, 1630, 1495, 1249, 1074, 989, 700 $cm^{-1}$. HRMS (ESI): *m/z* calcd. for $[M+H, C_{28}H_{29}F_5NO_2]^+$: 506.2113; found: 506.2112.

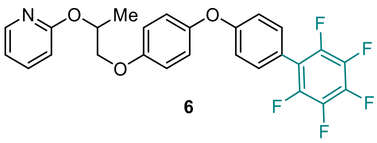

6

**2-((1-(4-((2',3',4',5',6'-pentafluoro-[1,1'-biphenyl]-4-yl)oxy)phenoxy)propan-2-yl)oxy)pyridine (6)**

The general procedure was followed using pentafluorobenzene (336.1 mg, 2 mmol) and **LS6** (154 mg, 0.2 mmol) at 120 °C for 24 hours. Purification by column chromatography on silica gel (hexane/EtOAc : 10/1) provided **6** (36.0 mg, 37%) as oil. $^1$H NMR (400 MHz, $CDCl_3$): δ = 8.16 (ddd, *J* = 5.0, 2.0, 0.8 Hz, 1H), 7.57 (ddd, *J* = 8.3, 7.1, 2.1 Hz, 1H), 7.38 – 7.30 (m, 2H), 7.06 – 6.96 (m, 6H), 6.87 (ddd, *J* = 7.1, 5.0, 0.9 Hz, 1H), 6.75 (dt, *J* = 8.4, 0.9 Hz, 1H), 5.60 (qt, *J* = 6.3, 5.0 Hz, 1H), 4.21 (dd, *J* = 9.9, 5.3 Hz, 1H), 4.09 (dd, *J* = 9.9, 4.8 Hz, 1H), 1.49 (d, *J* = 6.4 Hz, 3H); $^{13}$C NMR (101 MHz, $CDCl_3$): δ = 163.3 ($C_q$), 159.8 ($C_q$), 155.9 ($C_q$), 149.3 ($C_q$), 146.9 (CH), 144.3 (dm, *J* = 246.6 Hz, $C_q$), 140.3 (dm, *J* = 249.5 Hz, $C_q$), 138.9 (CH), 137.9 (dm, *J* = 235.3 Hz, $C_q$), 131.7 (CH), 121.6 (CH), 120.0 ($C_q$), 117.3 (CH), 116.9 (CH), 116.1 (CH), 115.9 – 115.3 (m, $C_q$), 111.8 (CH), 71.2 ($CH_2$), 69.4 (CH), 17.2 ($CH_3$); $^{19}$F NMR (377 MHz, $CDCl_3$): δ = -143.48 (dd, *J* = 23.1, 8.2 Hz, 2F), -156.10 (t, *J* = 20.9 Hz, 1F), -161.80 – -163.28 (m, 2F). IR (ATR): 2972, 1595, 1494, 1463, 1279, 1223, 1056, 986, 869, 835, 778 $cm^{-1}$. HRMS (ESI): *m/z* calcd. for $[M+H, C_{26}H_{19}F_5NO_3]^+$: 488.1280; found: 488.1273.

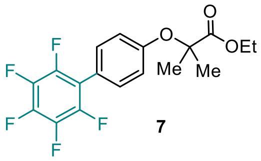

7

**ethyl 2-methyl-2-((2',3',4',5',6'-pentafluoro-[1,1'-biphenyl]-4-yl)oxy)propanoate (7)**

The general procedure was followed using pentafluorobenzene (336.1 mg, 2 mmol) and **LS7** (131 mg, 0.2 mmol) at 120°C for 24 hours. Purification by column chromatography on silica gel (hexane/EtOAc : 10/1) provided **7** (56.9 mg, 76%) as oil. $^1$H NMR (400 MHz, $CDCl_3$): δ = 7.33 – 7.28 (m, 2H), 6.95 – 6.90 (m, 2H), 4.25 (q, *J* = 7.1 Hz, 2H), 1.65 (s, 6H), 1.24 (t, *J* = 7.1 Hz, 3H); $^{13}$C NMR (101 MHz, $CDCl_3$): δ = 174.0 ($C_q$), 156.3 ($C_q$), 144.1 (dm, *J* = 243.1 Hz, $C_q$), 140.1 (dm, *J* = 253.1 Hz, $C_q$), 137.8 (dm, *J* = 251.5 Hz, $C_q$), 131.1 (CH), 119.5 ($C_q$), 118.5 (CH), 116.0 – 114.8 (m, $C_q$), 79.2 ($C_q$), 61.6 ($CH_2$), 25.4 ($CH_3$), 14.0 ($CH_3$); $^{19}$F NMR (377 MHz, $CDCl_3$): δ = -143.52 (dd, *J* = 23.0, 8.1 Hz, 2F), -156.31 (t, *J* = 21.0 Hz, 1F), -161.54 – -163.12 (m, 2F). IR (ATR): 2992, 1734, 1500, 1485, 1245, 1177, 1138, 1062, 984, 864, 837 $cm^{-1}$. HRMS (ESI): *m/z* calcd. for $[M+Na, C_{18}H_{15}F_5NaO_3]^+$: 397.0834; found: 397.0841.

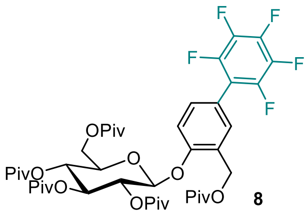

8

The general procedure was followed using pentafluorobenzene (336.1 mg, 2 mmol) and **LS8** (230.3 mg, 0.2 mmol) at 120°C for 24 hours. Purification by column

chromatography on silica gel (hexane/EtOAc : 9/1) provided **8** (136.1 mg, 78%) as oil. $^{1}$H NMR (400 MHz, $CDCl_3$): δ = 7.36 (s, 1H), 7.28 (d, *J* = 8.7 Hz, 1H), 7.10 (d, *J* = 8.6 Hz, 1H), 5.46 (t, *J* = 9.3 Hz, 1H), 5.41 – 5.34 (m, 1H), 5.24 (d, *J* = 7.8 Hz, 1H), 5.21 – 5.15 (m, 1H), 5.14 (s, 2H), 4.25 (dd, *J* = 12.3, 1.8 Hz, 1H), 4.05 (dd, *J* = 12.2, 6.8 Hz, 1H), 3.93 (ddd, *J* = 10.1, 6.8, 1.8 Hz, 1H), 1.22 (s, 9H), 1.19 (s, 9H), 1.17 (s, 9H), 1.16 (s, 9H), 1.14 (s, 9H); $^{13}$C NMR (101 MHz, $CDCl_3$): δ = 178.1 ($C_q$), 178.0 ($C_q$), 177.3 ($C_q$), 176.7 ($C_q$), 176.6 ($C_q$), 154.4 ($C_q$), 144.3 (dm, *J* = 247.4 Hz, $C_q$), 140.5 (dm, *J* = 245.4 Hz, $C_q$), 138.0 (dm, *J* = 249.2 Hz, $C_q$), 130.7 (CH), 130.2 (CH), 127.4 ($C_q$), 121.1 ($C_q$), 115.2 (td, *J* = 17.0, 4.0 Hz, $C_q$), 114.7 (CH), 98.7 (CH), 72.9 (CH), 72.1 (CH), 71.0 (CH), 68.1 (CH), 62.3 ($CH_2$), 60.1 ($CH_2$), 39.0 ($C_q$), 38.9 ($C_q$), 38.9 ($C_q$), 27.3 ($CH_3$), 27.3 ($CH_3$), 27.2 ($CH_3$), 27.2 ($CH_3$), 27.2 ($CH_3$) two $C_q$ resonances are missing due to overlap; $^{19}$F NMR (377 MHz, $CDCl_3$): δ = -143.65 (dd, *J* = 23.0, 8.1 Hz, 2F), -155.57 (t, *J* = 20.9 Hz, 1F), -162.07 (td, *J* = 22.5, 8.1 Hz, 2F). IR (ATR): 2968, 1739, 1490, 1278, 1127, 1073, 987, 767 $cm^{-1}$. HRMS (ESI): *m/z* calcd. for [M+Na, $C_{44}H_{57}F_5NaO_{12}$]$^+$: 895.3662; found: 895.3662.

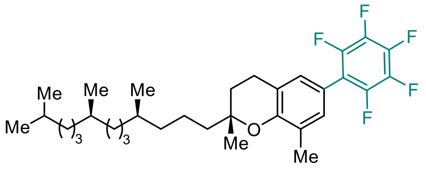


**9**

**(*R*)-2,8-dimethyl-6-(perfluorophenyl)-2-((*4R,8R*)-4,8,12-trimethyltridecyl)chromane (9)**

The general procedure was followed using pentafluorobenzene (336.1 mg, 2 mmol) and **LS9** (166 mg, 0.2 mmol) at 120 °C for 24 hours. Purification by column chromatography on silica gel (hexane) provided **9** (86.2 mg, 78%) as oil. $^{1}$H NMR (400 MHz, $CDCl_3$): δ = 7.02 (s, 1H), 6.97 (s, 1H), 2.80 (td, *J* = 6.6, 3.9 Hz, 2H), 2.21 (s, 3H), 1.95 – 1.73 (m, 2H), 1.67 – 1.00 (m, 24H), 0.92 – 0.77 (m, 12H); $^{13}$C NMR (101 MHz, $CDCl_3$): δ = 153.3 ($C_q$), 144.3 (dm, *J* = 242.1 Hz, $C_q$), 139.8 (dm, *J* = 252.3 Hz, $C_q$), 137.9 (dm, *J* = 251.5 Hz, $C_q$),130.0 (CH), 128.9 (CH), 126.9 ($C_q$), 120.9 ($C_q$), 116.7 – 116.2 (m, $C_q$), 116.4 ($C_q$), 76.9 ($C_q$), 40.5 ($CH_2$), 39.5 ($CH_2$), 37.6 ($CH_2$), 37.6 ($CH_2$), 37.4 ($CH_2$), 33.0 ($CH_3$), 32.9 ($CH_3$), 31.1 ($CH_2$), 28.1 (CH), 25.0 ($CH_2$), 24.6 ($CH_2$), 24.5 ($CH_3$), 22.9 (CH), 22.8 (CH), 22.4 ($CH_2$), 21.1 ($CH_2$), 19.9 ($CH_3$), 19.8 ($CH_3$), 16.3 ($CH_3$) one $CH_2$ resonance is missing due to overlap; $^{19}$F NMR (377 MHz, $CDCl_3$): δ = -143.51 (dd, *J* = 23.5, 8.1 Hz, 2F), -157.42 (t, *J* = 21.0 Hz, 1F), -162.97 (ddd, *J* = 23.2, 20.3, 8.0 Hz, 2F). IR (ATR): 2955, 2924, 2865, 1523, 1490, 1376, 1234, 1080, 983, 950, 700 $cm^{-1}$. HRMS (EI): *m/z* calcd. for [M, $C_{33}H_{45}F_5O$]$^+$ : 552.3385; found: 552.3384.

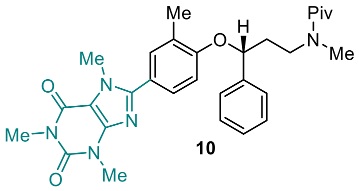


**10**

The general procedure was followed using Caffeine (194 mg, 1.0 mmol), **LS5** (157 mg, 0.2 mmol) and *t*BuCN (0.3 mL) at 120 °C for 24 hours. Purification by column chromatography on silica gel (hexane/EtOAc : 1/2) provided **10** (42.4 mg, 40%) as oil. $^1$H NMR (300 MHz, $CDCl_3$) δ 7.47 (d, $J$ = 2.2 Hz, 1H), 7.37 – 7.19 (m, 6H), 6.69 (d, $J$ = 8.6 Hz, 1H), 5.30 – 5.22 (m, 1H), 3.97 (s, 3H), 3.70 – 3.49 (m, 2H) 3.58 (s, 3H), 3.40 (s, 3H), 3.04 (s, 3H), 2.41 (s, 3H), 2.29 – 2.08 (m, 2H), 1.25 (s, 9H); $^{13}$C NMR (101 MHz, $CDCl_3$) δ 177.4 ($C_q$), 157.4 ($C_q$), 155.5 ($C_q$), 152.3 ($C_q$), 151.7 ($C_q$), 148.2 ($C_q$), 140.7 ($C_q$), 131.5 (CH), 128.8 (CH), 127.9 (CH), 127.9 ($C_q$), 127.8 (CH), 125.6 (CH), 120.2 ($C_q$), 112.7 (CH), 108.2 ($C_q$), 78.0 (CH), 47.5 ($CH_2$), 38.7 ($CH_2$), 36.7 ($CH_3$), 36.4 ($C_q$), 33.8 ($CH_3$), 29.7 ($CH_3$), 28.2 ($CH_3$), 27.9 ($CH_3$), 16.7 ($CH_3$). IR (ATR): 2955, 1701, 1659, 1625, 1541, 1479, 1431, 1250, 1230, 1134, 1035 cm$^{-1}$. HRMS (ESI): $m/z$ calcd. for [M+H, $C_{30}H_{38}N_5O_4$]$^+$ : 532.2918; found: 532.2913.

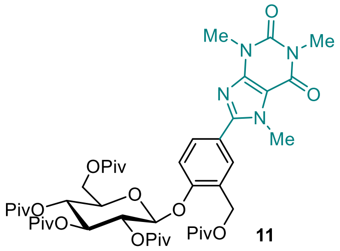


**11**

The general procedure was followed using Caffeine (388 mg, 2.0 mmol), **LS8** (230.3 mg, 0.2 mmol) and *t*BuCN (0.3 mL) at 120 °C for 48 hours. Purification by column chromatography on silica gel (hexane/EtOAc : 1/1) provided **11** (53.8 mg, 30%) as oil. $^1$H NMR (400 MHz, $CDCl_3$) δ 7.63 (d, $J$ = 2.2 Hz, 1H), 7.55 (dd, $J$ = 8.6, 2.3 Hz, 1H), 7.11 (d, $J$ = 8.6 Hz, 1H), 5.47 (t, $J$ = 9.3 Hz, 1H), 5.37 (dd, $J$ = 9.5, 7.8 Hz, 1H), 5.30 – 5.17 (m, 2H), 5.14 (s, 2H), 4.26 (dd, $J$ = 12.3, 1.9 Hz, 1H), 4.11 – 4.04 (m, 1H), 4.02 (s, 3H), 3.94 (ddd, $J$ = 10.2, 6.3, 1.8 Hz, 1H), 3.60 (s, 3H), 3.42 (s, 3H), 1.24 (s, 9H), 1.21 (s, 9H), 1.17 (s, 9H), 1.14 (s, 9H), 1.14 (s, 9H); $^{13}$C NMR (101 MHz, $CDCl_3$) δ 177.9 ($C_q$), 177.9 ($C_q$), 177.1 ($C_q$), 176.5 ($C_q$), 176.4 ($C_q$), 155.6 ($C_q$), 155.1 ($C_q$), 151.7 ($C_q$), 151.4 ($C_q$), 148.2 ($C_q$), 129.6 (CH), 129.0 (CH), 127.4 ($C_q$), 123.1 ($C_q$), 114.3 (CH), 108.5 ($C_q$), 98.6 (CH), 72.8 (CH), 71.9 (CH), 70.8 (CH), 67.7 (CH), 62.0 ($CH_2$), 60.1 ($CH_2$), 38.9 ($C_q$), 38.8 ($C_q$), 38.8 ($C_q$), 38.7 ($C_q$), 33.9 ($CH_3$), 29.7 ($CH_3$), 28.0 ($CH_3$), 27.2 ($CH_3$), 27.1 ($CH_3$), 27.1 ($CH_3$), 27.0 ($CH_3$), 27.0 ($CH_3$). one $C_q$ resonance is missing due to overlap. IR (ATR): 2972, 1742, 1704, 1663, 1480, 1279, 1135, 1070, 1036, 980 cm$^{-1}$. HRMS (ESI): $m/z$ calcd. for [M+Na, $C_{46}H_{66}N_4O_{14}Na$]$^+$ : 921.4468; found: 921.4468.

## 6. Mechanistic Experimental Studies

(1) Competition experiment

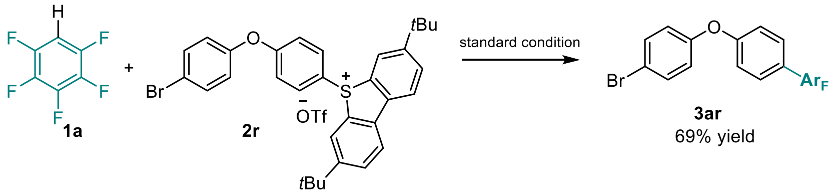


The general procedure was followed using pentafluorobenzene (336.1 mg, 2 mmol) and **2r** (139 mg, 0.2 mmol) at 120 °C for 24 hours. Purification by column chromatography on silica gel (hexane) provided **3ar** (57.3 mg, 69%) as colorless solid. $^{1}$H NMR (400 MHz, $CDCl_3$): δ = 7.52 – 7.46 (m, 2H), 7.43 – 7.37 (m, 2H), 7.12 – 7.07 (m, 2H), 7.00 – 6.95 (m, 2H); $^{13}$C NMR (101 MHz, $CDCl_3$): δ = 158.1 ($C_q$), 155.6 ($C_q$), 144.3 (dm, $J$ = 247.0 Hz, $C_q$), 140.5 (dm, $J$ = 249.5 Hz, $C_q$), 138.0 (dm, $J$ = 252.8 Hz, $C_q$), 133.1 (CH), 131.9 (CH), 121.5 (CH), 121.3 ($C_q$), 118.6 (CH), 116.8 ($C_q$), 115.4 (td, $J$ = 17.1, 4.1 Hz, $C_q$); $^{19}$F NMR (377 MHz, $CDCl_3$): δ = -143.40 (dd, $J$ = 23.0, 8.2 Hz, 2F), -155.62 (t, $J$ = 20.9 Hz, 1F), -162.14 (ddd, $J$ = 23.0, 20.7, 8.2 Hz, 2F). IR (ATR): 1502, 1483, 1240, 1064, 986, 880, 828 cm$^{-1}$. HRMS (EI): *m/z* calcd. for [M, $C_{18}H_8BrF_5O$]$^+$ : 413.9673; found: 413.9677.

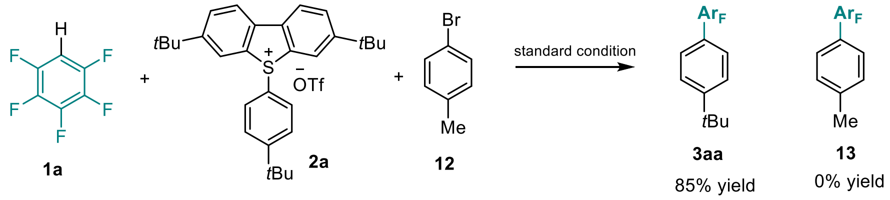


To an oven-dried screw tube, Ru(OPiv)$_2$(*p*-cymene) (8.8 mg, 10 mol%), PivOH (6.12 mg, 30 mol%), **2a** (115.8 mg, 0.2 mmol, 1 equiv), and $K_2CO_3$ (63.6 mg, 0.46 mmol, 2.3 equiv) were added. The screw tube was then moved to glovebox, and charged with polyfluoroarene (2 mmol, 10 equiv), 1-bromo-4-methylbenzene **12** (34.2 mg, 0.2 mmol, 1 equiv) and pivalonitrile (220 uL) which were completely degassed and stored in glovebox. Next, the screw tube was taken out of the glovebox, and heated at oil bath for the corresponding time. After the reaction time point, the reaction mixture was taken out of the oil bath, and cooled to room temperature. The reaction mixture was diluted with dichloromethane and filtered through celite gel. The solution was concentrated under reduced pressure, then purified by column chromatography on silica gel, providing **3aa** in 85% yield. Compound **13** was not detected.

(2) Examination of non-cycloruthenated intermediate

deuterium incorporation experiment

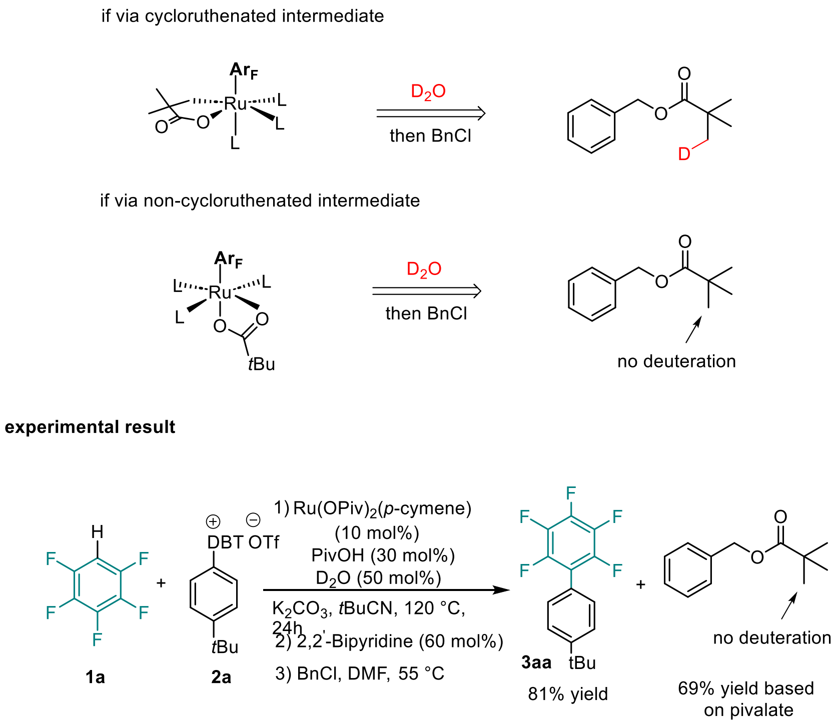


To an oven-dried screw tube, Ru(OPiv)$_2$(*p*-cymene) (26.4 mg, 10 mol%), PivOH (18.4 mg, 30 mol%), **2a** (347.4 mg, 0.6 mmol, 1 equiv), $K_2CO_3$ (207.3 mg, 1.50 mmol, 2.5 equiv) were added. The screw tube was then moved to glovebox, and charged with $D_2O$ (6.0 mg, 0.3 mmol, 50 mol%), pentafluorobenzene (6.0 mmol, 10 equiv), and pivalonitrile (660 uL) which were completely degassed and stored in glovebox. Next, the screw tube was taken out of the glovebox, and heated at oil bath for 24 hours.

After that time point, the reaction tube was taken out of the oil bath, and cooled to room temperature. Next, 2,2'-bipyridine (56.2 mg, 0.36 mmol) was added to the reaction mixture under nitrogen, the screw tube was heated at oil for another 2 hours, then the tube was taken out of the oil bath, and cooled to room temperature.

The reaction mixture was evacuated under reduced pressure to remove the pentafluorobenzene and pivalonitrile. Subsequently, 2.0 mL of N,N-dimethylformamide (DMF) and benzyl chloride (227 mg, 1.8 mmol) were added under nitrogen atmosphere, the reaction mixture was stirred at 55°C for 3 hours. After cooling to room temperature, the solvent was removed under vacuo, the residue was dissolved in 20 mL $CH_2Cl_2$ which was poured into a separatory funnel. Water (15 mL) was added to the funnel, the $CH_2Cl_2$ layer was collected, and the aqueous layer was further extracted with $CH_2Cl_2$ (2 × ca. 15 mL). The combined $CH_2Cl_2$ solution was dried over $Na_2SO_4$, filtered, and the solvent was removed under reduced pressure. The residue was purified by chromatography on silica gel eluting with hexane/ EtOAc (100:0 to 20:1), affording **3aa** (145.9 mg, 81%) as a colorless solid and benzyl pivalate **12** (39.7 mg, 69% yield) as colorless oil. HRMS (ESI) of compound **12**: *m/z* calcd. for [M+Na, $C_{12}H_{16}O_2Na]^+$ : 215.1043; found: 215.1042.

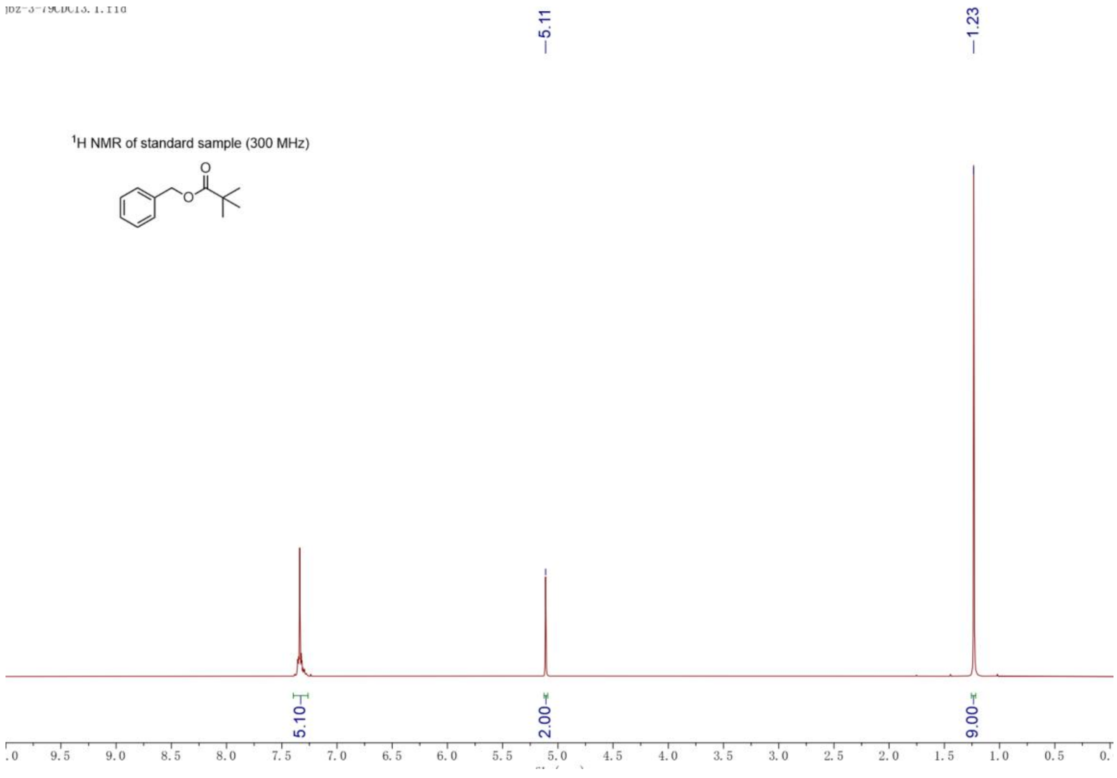


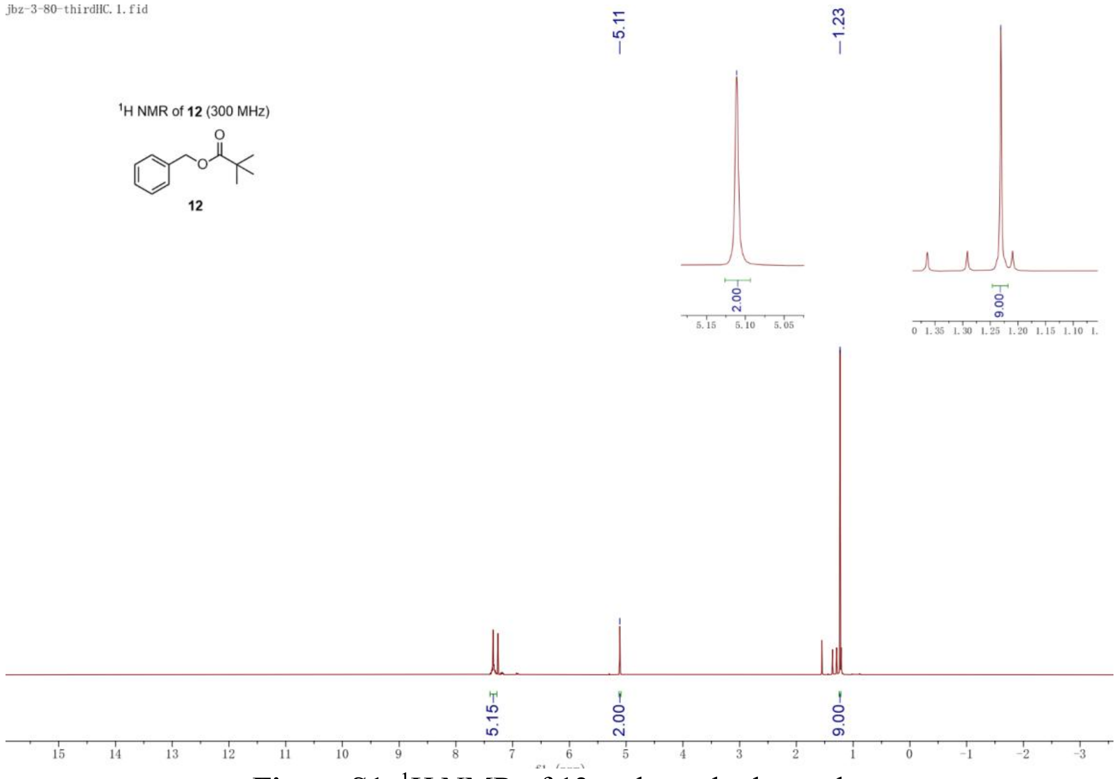


**Figure S1.** $^1$H NMR of **12** and standard sample.

(3) Comparative experiments using different Ru(II) catalysts **without additive**

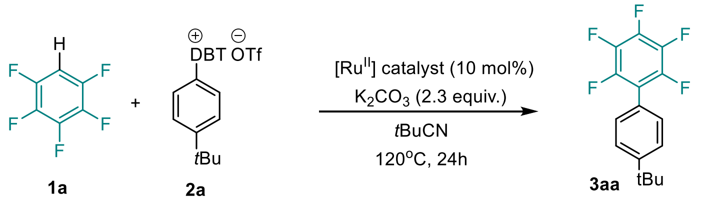


| | |
|---|---|
| [Ru(OAc)$_2$(*p*-cymene)] | 51% yield |
| [Ru(MesCO$_2$)$_2$(*p*-cymene)] | 62% yield |
| [Ru(*t*BuCN)$_6$][BF$_4$]$_2$ | 19% yield |

To an oven-dried screw tube, [Ru] catalyst (10 mol%), arylsulfonium salt **2a** (57.8 mg, 0.1 mmol, 1 equiv), and $K_2CO_3$ (31.8 mg, 0.23 mmol, 2.3 equiv) were added. The screw tube was then moved to glovebox, and charged with pentafluorobenzene (1 mmol, 10 equiv) and pivalonitrile (110 μL) which were completely degassed and stored in glovebox. Next, the screw tube was taken out of the glovebox, and heated at oil bath for the corresponding time. After the reaction time point, the reaction mixture was taken out of the oil bath, and cooled to room temperature. The reaction mixture was added 0.1 mmol 1,3,5-trimethoxybenzene as internal standard, then the mixture was diluted with dichloromethane and filtered through celite gel. The homogenous solution was concentrated under reduced pressure, delivering the crude product that was tested using $^1$H NMR to assay the yield. All of the [Ru] catalysts delivered product **3aa**, which demonstrated the non-cycloruthenated complex being responsible for the oxidative addition step.

(4) KIE experiment of fluoroarenes **d$_1$-1k** and **1k** with arylsulfonium salt **2a** in separate flasks.

Ru(OPiv)$_2$(*p*-cymene) (4.4 mg, 10 mol%), **2a** (57.8 mg, 0.1 mmol, 1.0 equiv), and $K_2CO_3$ (27.6 mg, 0.2 mmol, 2.0 equiv) were added to a dried screw tube. The screw tube was then moved to glovebox, and charged with **1k** or **d$_1$-1k** (1 mmol, 10 equiv) and pivalonitrile (110 μL) which were completely degassed and stored in glovebox. Next, the screw tube was taken out of the glovebox, and heated at oil bath for the corresponding time. After the reaction time point, the reaction tube was taken out of the oil bath, and cooled quickly to room temperature in cold water. The reaction mixture was diluted with dichloromethane and filtered through celite gel. The solution was concentrated under reduced pressure, then the residue was evacuated using pump to remove the **1k** or **d$_1$-1k**. A given amount of 1,3,5-trimethoxybenzene was added as internal standard to the above residue which was dissolved in $CDCl_3$. NMR yields were obtained by $^1$H-NMR analysis.

Kinetic isotope effect experiment

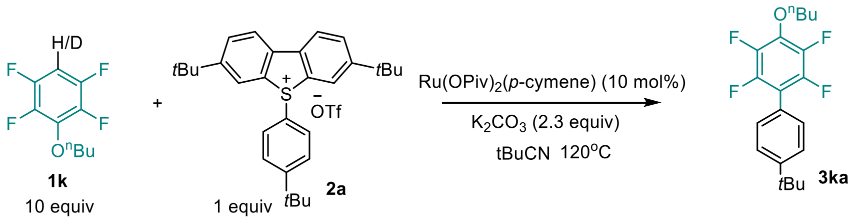


| entry | fluoroarene | time (min) | **3ak** (%) |
|---|---|---|---|
| 1 | **1k** | 20 | 4.2 |
| 2 | **1k** | 30 | 6.4 |
| 3 | **1k** | 40 | 8.6 |
| 4 | **1k** | 50 | 9.9 |
| 5 | **1k** | 60 | 12.4 |
| 6 | **d$_1$-1k** | 20 | 2.9 |
| 7 | **d$_1$-1k** | 30 | 4.2 |
| 8 | **d$_1$-1k** | 40 | 4.9 |
| 9 | **d$_1$-1k** | 50 | 5.9 |
| 10 | **d$_1$-1k** | 60 | 6.9 |

**Table S3.** KIE experiment for polyfluoroarenes **1k** and **d$_1$-1k** with **2a**

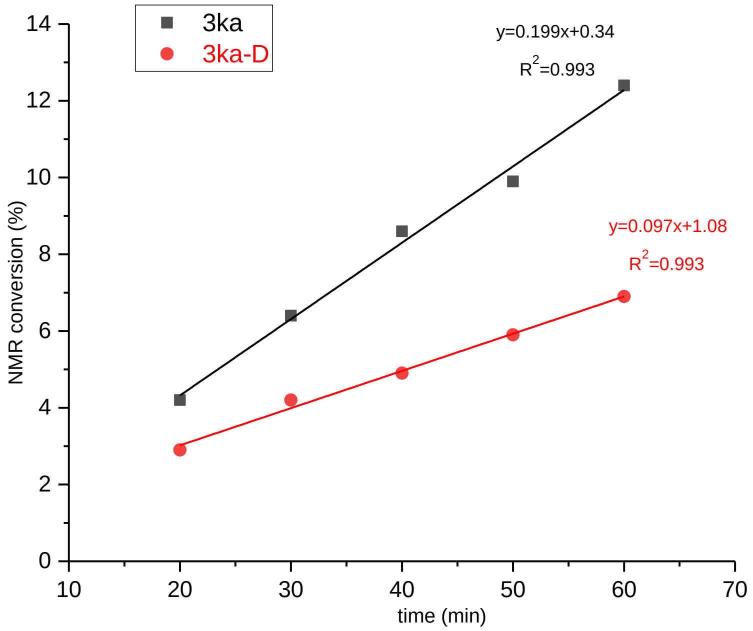


**Figure S2**.

Rate of formation of **3ka** using **1k** and **d$_1$-1k**

$$\textbf{KIE} = \frac{k\text{H}}{k\text{D}} = \frac{0.199}{0.097} = \textbf{2.05}$$

(5) Radical scavenger experiment

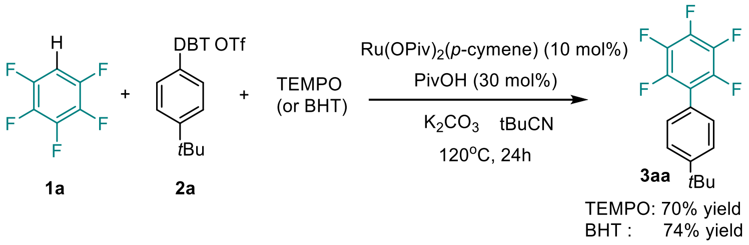


To an oven-dried screw tube, $Ru(OPiv)_2$(*p*-cymene) (8.8 mg, 10 mol%), PivOH (6.12 mg, 30 mol%), **2a** (115.8 mg, 0.2 mmol, 1 equiv), and $K_2CO_3$ (63.6 mg, 0.46 mmol, 2.3 equiv) were added. The screw tube was then moved to glovebox, and charged with TEMPO (2,2,6,6-Tetramethyl-1-piperidyloxy) (62.5 mg, 0.4 mmol, 2 equiv) or BHT (Butylated Hydroxytoluene) (88 mg, 0.4 mmol, 2 equiv), polyfluoroarene (2 mmol, 10 equiv), and pivalonitrile (220 uL) which were completely degassed and stored in glovebox. Next, the screw tube was taken out of the glovebox, and heated at oil bath for the corresponding time. After the reaction time point, the reaction mixture was taken out of the oil bath, and cooled to room temperature. The reaction mixture was diluted with dichloromethane and filtered through celite gel. The solution was concentrated under reduced pressure, then purified by column chromatography on silica gel. When TEMPO was added, the reaction provided **3aa** in 70% yield. When BHT was added, the reaction provided **3aa** in 74% yield.

(6) Radical trapping experiment

Synthesis of arylsulfonium salt **2t**

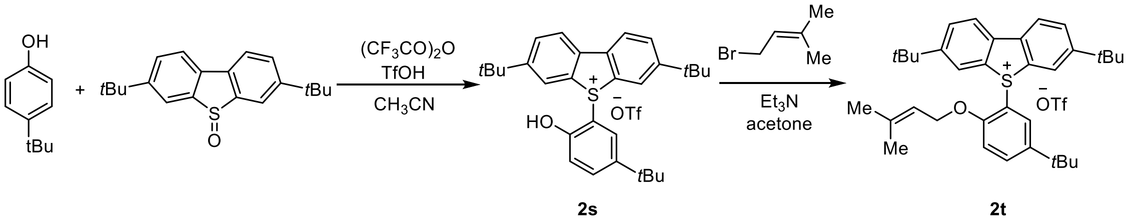


A dry 100 mL Schlenk flask equipped with a magnetic stir bar was charged with 4-*tert*-Butylphenol (288 mg, 1.92 mmol, 1 equiv.) and dry MeCN (8 mL) under nitrogen atmosphere at room temperature. After cooling to -30°C, $(CF_3CO)_2O$ (0.66 mL, 4.8 mmol, 2.5 equiv.) and TfOH (0.35 mL, 3.84 mmol, 2 equiv.) were added to the stirred reaction mixture. Subsequently, 3,7-di-*tert*-butyldibenzothiophene S-oxide (600 mg, 1.92 mmol, 1.0 equiv.) was added to the stirred reaction mixture in small portions over 3 minutes. After addition, the reaction mixture was stirred at -30°C for 1 h. Next, the Schlenk flask was taken out of the cold bath and warmed to 25°C in air. After stirring at 25°C for another 1 h, the reaction mixture was evaporated in vacuum to remove most of solvent, then the residue was diluted with DCM (50 mL) and poured onto saturated aqueous $NaHCO_3$ (20 mL). Then, the mixture was poured into a separatory funnel, and the layers were separated. The DCM layer was collected, and washed with aqueous NaOTf solution (15 mL x 2, 5 wt%), dried over anhydrous $Na_2SO_4$. After filtration, the mixture was concentrated to dryness under reduced pressure, the crude product was used for the next step without further purification.

Under ambient atmosphere, a 20 mL flask was charged with **2s** (363 mg, 0.61 mmol, 1 equiv.) and acetone (6.0 mL, c = 0.10 M). After addition of triethylamine (250 mg, 2.44 mmol, 4.00 equiv.) at 25°C, the reaction mixture turned into a clear solution. Prenylbromide (363 mg, 2.44 mmol, 4.00 equiv.) was subsequently added into the clear solution at 25 °C. After stirring at 25 °C for 30 min, colorless precipitates formed in the reaction mixture. After the resulting mixture was stirred at 25 °C for 12 h further, the reaction mixture was concentrated under reduced pressure, and the residue was dissolved in 30 mL $CH_2Cl_2$. The resulting mixture was poured into a separatory funnel, which was pre-charged with 20 mL of water. The $CH_2Cl_2$ layer was collected, and the aqueous layer was further extracted with $CH_2Cl_2$ (2 × ca. 20 mL). The combined $CH_2Cl_2$ solution was washed with aqueous NaOTf solution (2 × ca. 30 mL, 5 % w/w). The organic layer was dried over $Na_2SO_4$, filtered, and the solvent was removed under reduced pressure. The residue was purified by chromatography on silica gel eluting with DCM/MeOH (80:1 to 50:1), affording **2t** (230.5 mg, 57%) as a colorless oil. $^1$H NMR (300 MHz, $CDCl_3$): δ = 8.11 (d, $J$ = 8.3 Hz, 2H), 8.01 (d, $J$ = 1.7 Hz, 2H), 7.84 (dd, $J$ = 8.3, 1.8 Hz, 2H), 7.65 (dd, $J$ = 8.8, 2.3 Hz, 1H), 7.15 (s, 1H), 7.11 (d, $J$ = 8.8 Hz, 1H), 5.23 (t, $J$ = 7.2 Hz, 1H), 4.59 (d, $J$ = 7.0 Hz, 2H), 1.75 (s, 3H), 1.63 (s, 3H), 1.32 (s, 18H), 1.14 (s, 9H); $^{13}$C NMR (75 MHz, $CDCl_3$): δ = 156.2 ($C_q$), 155.1 ($C_q$), 146.2 ($C_q$), 140.5 ($C_q$), 137.0 ($C_q$), 134.3 (CH), 131.6 (CH), 129.2 ($C_q$), 126.5 (CH), 124.8 (CH), 123.7 (CH), 121.0 (q, $J$ = 318.8, $C_q$), 117.5 (CH), 114.2 (CH), 112.3 ($C_q$), 66.8 ($CH_2$), 35.6 ($C_q$), 34.7 ($C_q$), 31.1 ($CH_3$), 31.0 ($CH_3$), 25.8 ($CH_3$), 18.1 ($CH_3$); $^{19}$F NMR (282 MHz, $CDCl_3$): δ = -78.02 (s, 3F). HRMS-ESI (m/z) calculated for $C_{35}H_{45}OS^+$ [M-OTf]$^+$, 513.3186; found: 513.3184.

Radical trapping experiment using **2t**

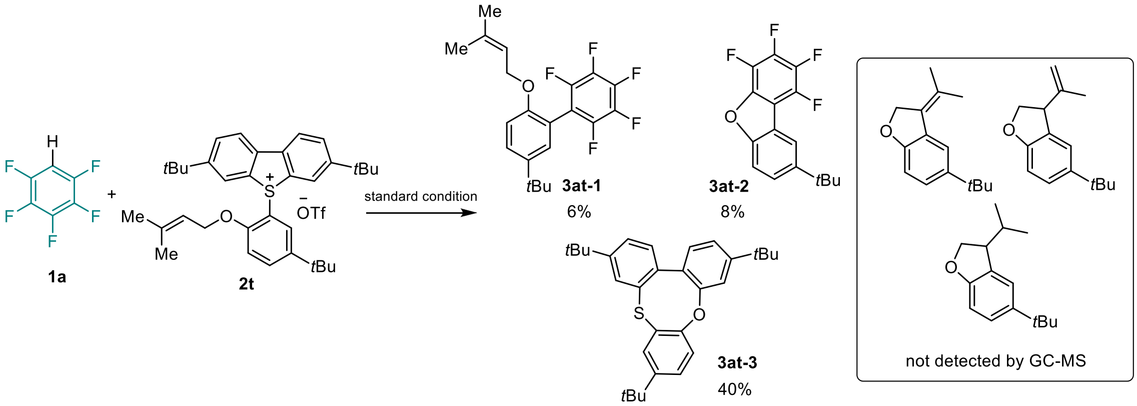


The general procedure was followed using pentafluorobenzene (336.1 mg, 2 mmol) and **2t** (132.5 mg, 0.2 mmol) at 120°C for 24 hours. **3at-1**, **3at-2**, **3at-3** were isolated by column chromatography on silica gel.

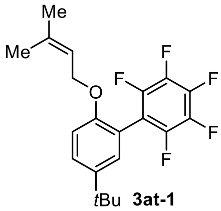

**3at-1**

**3at-1** (4.6 mg, ≤ 6% yield) was obtained as oil. $^1$H NMR (500 MHz, $CDCl_3$): δ = 7.43 (dd, *J* = 8.7, 2.5 Hz, 1H), 7.21 (d, *J* = 2.5 Hz, 1H), 6.95 (d, *J* = 8.8 Hz, 1H), 5.33 (tt, *J* = 6.5, 1.4 Hz, 1H), 4.52 (d, *J* = 6.6 Hz, 2H), 1.74 (s, 3H), 1.68 (s, 3H), 1.32 (s, 9H); $^{13}$C NMR (126 MHz, $CDCl_3$): δ = 154.4 ($C_q$), 144.6 (dm, *J* = 247.1 Hz, $C_q$), 143.4 ($C_q$), 140.5 (dm, *J* = 252.1 Hz, $C_q$), 137.7 ($C_q$), 137.7 (dm, *J* = 249.5 Hz, $C_q$), 129.0 (CH), 127.8 (CH), 119.9 (CH), 115.2 ($C_q$), 113.6 (td, *J* = 19.2, 4.2 Hz, $C_q$), 112.4 (CH), 65.7 ($CH_2$), 34.3 ($C_q$), 31.6 ($CH_3$), 25.9 ($CH_3$), 18.3 ($CH_3$); $^{19}$F NMR (471 MHz, $CDCl_3$): δ = -139.99 (dd, *J* = 23.2, 8.0 Hz, 2F), -156.73 (t, *J* = 20.9 Hz, 1F), -163.14 – -164.03 (m, 2F). HRMS (ESI): *m/z* calcd. for $[M+Na, C_{21}H_{21}F_5ONa]^+$ : 407.1405; found: 407.1403.

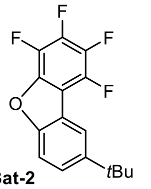

3at-2

**3at-2** (4.7 mg, 8% yield) was obtained as solid. $^1$H NMR (500 MHz, $CDCl_3$): δ = 8.01 (d, *J* = 2.0 Hz, 1H), 7.60 (dd, *J* = 8.8, 2.1 Hz, 1H), 7.54 (dd, *J* = 8.8, 0.6 Hz, 1H), 1.43 (s, 9H); $^{13}$C NMR (126 MHz, $CDCl_3$): δ = 154.9 ($C_q$), 147.9 ($C_q$), 141.2 (dm, *J* = 250.7 Hz, $C_q$), 140.2 (dm, *J* = 245.7 Hz, $C_q$), 139.5 – 138.8 (m, $C_q$), 137.1 (dm, *J* = 246.9 Hz, $C_q$), 134.5 (dm, *J* = 251.5 Hz, $C_q$), 126.5 (CH), 121.2 – 120.4 (m, $C_q$), 119.3 (d, *J* = 2.9 Hz, CH), 111.4 (CH), 110.9 (d, *J* = 18.6 Hz, $C_q$), 35.1 ($C_q$), 31.9 ($CH_3$); $^{19}$F NMR (471 MHz, $CDCl_3$): δ = -146.47 (dd, *J* = 21.0, 15.4 Hz, 1F), -157.91 (t, *J* = 19.7 Hz, 1F), -160.40 – -162.09 (m, 1F), -164.32 (t, *J* = 20.4 Hz, 1F). HRMS (EI): *m/z* calcd. for $[M, C_{16}H_{12}F_4O]^+$ : 296.0819; found: 296.0823.

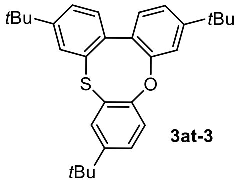

3at-3

**3at-3** (35.5 mg, 40% yield) was obtained as oil. $^1$H NMR (400 MHz, $CDCl_3$): δ = 7.85 (d, *J* = 2.0 Hz, 1H), 7.53 (dd, *J* = 8.0, 2.0 Hz, 1H), 7.47 – 7.42 (m, 2H), 7.34 – 7.28 (m, 2H), 7.24 – 7.22 (m, 2H), 7.13 (dd, *J* = 1.7, 0.6 Hz, 1H), 1.36 (s, 9H), 1.34 (s, 9H), 1.33 (s, 9H); $^{13}$C NMR (101 MHz, $CDCl_3$): δ = 153.9 ($C_q$), 153.5 ($C_q$), 152.0 ($C_q$), 151.7 ($C_q$), 147.2 ($C_q$), 142.3 ($C_q$), 133.9 ($C_q$), 133.4 (CH), 129.3 ($C_q$), 128.2 (CH), 127.9 (CH), 127.7 (CH), 127.5 (CH), 126.6 ($C_q$), 125.0 (CH), 122.5 (CH), 122.1 (CH), 118.4 (CH), 35.0 ($C_q$), 34.9 ($C_q$), 34.6 ($C_q$), 31.6 ($CH_3$), 31.5 ($CH_3$) one $CH_3$ resonance is missing due to overlap. HRMS (ESI): *m/z* calcd. for $[M+Na, C_{30}H_{36}OSNa]^+$ : 467.2379; found: 467.2379.

(7) Isolation of ruthenium intermediates

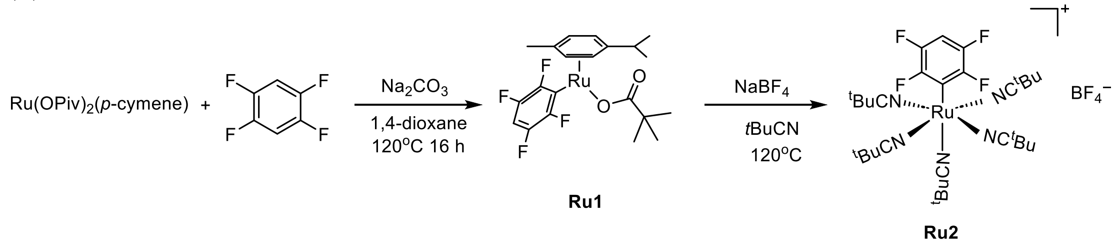


To an oven-dried screw-cap pressure tube, $Ru(OPiv)_2$(*p*-cymene) (160 mg, 0.365 mmol 1.0 equiv), $Na_2CO_3$ (77.5 mg, 0.731 mmol, 2.0 equiv) were added. The screw-cap pressure tube was then moved to glovebox, and charged with 1,2,4,5-Tetrafluorobenzene (7.31 mmol, 20 equiv) and 1,4-dioxane (750 μL) which were completely degassed and stored in glovebox. Next, the tube was taken out of the glovebox, and heated at 120°C for 16 hours. After the reaction time point, the reaction mixture was taken out of the oil bath, and cooled to room temperature. The reaction mixture was filtered through a cotton plug, then evaporated. The crude product was purified by chromatography on silica gel eluting with hexane/$Et_2O$ (100/0 to 100/20). **Ru1** was obtained as orange solid (122.2 mg, 69% yield). $^1$H NMR (300 MHz, $CDCl_3$) δ = 6.65 (tt, *J* = 9.5, 6.9 Hz, 1H), 5.56 (d, *J* = 5.9 Hz, 2H), 5.22 (d, *J* = 5.9 Hz, 2H), 2.86 (p, *J* = 6.9 Hz, 1H), 2.10 (s, 3H), 1.35 (d, *J* = 6.9 Hz, 6H), 0.76 (s, 9H); $^{19}$F NMR (282 MHz, $CDCl_3$) δ = -119.31 ~ -119.52 (m), -142.22 ~ -142.44 (m).

**Ru1** (97.1 mg, 0.2 mmol) and $NaBF_4$ (65.9 mg, 0.6 mmol) were place to an oven-dried screw-cap pressure tube, the tube was then moved to glovebox, and charged with pivalonitrile (4 mL). Next, the tube was taken out of the glovebox, and heated at 120°C for 75 min. After the reaction time point, the reaction mixture was taken out of the oil bath, and cooled to room temperature. The reaction mixture was diluted with 3 mL of pivalonitrile, filtered through a plug of Celite® and the remaining pale yellow/greenish solution was evaporated under reduced pressure. The crude product was then purified by crystallization from *t*BuCN/$Et_2O$ affording the **Ru2** as white solid (61.7 mg, 41% yield). $^1$H NMR (300 MHz, $CD_2Cl_2$) δ = 6.67 (tt, *J* = 9.8, 7.2 Hz, 1H), 1.58 (s, 9H), 1.43 (s, 36H); $^{13}$C NMR (101 MHz, $CDCl_2$) δ = 153.4 (dm, *J* = 223.5 Hz), 144.6 (dm, *J* = 245.6 Hz), 136.0 – 135.1 (m), 131.2, 128.8, 99.4 (t, *J* = 23.7 Hz), 30.1, 29.6, 28.3, 28.1; $^{19}$F NMR (282 MHz, $CD_2Cl_2$) δ = -121.39 – -121.57 (m), -144.89 – -145.07 (m), -153.27, -153.32. The obtained characterization data are in accordance with those previously reported.[31]

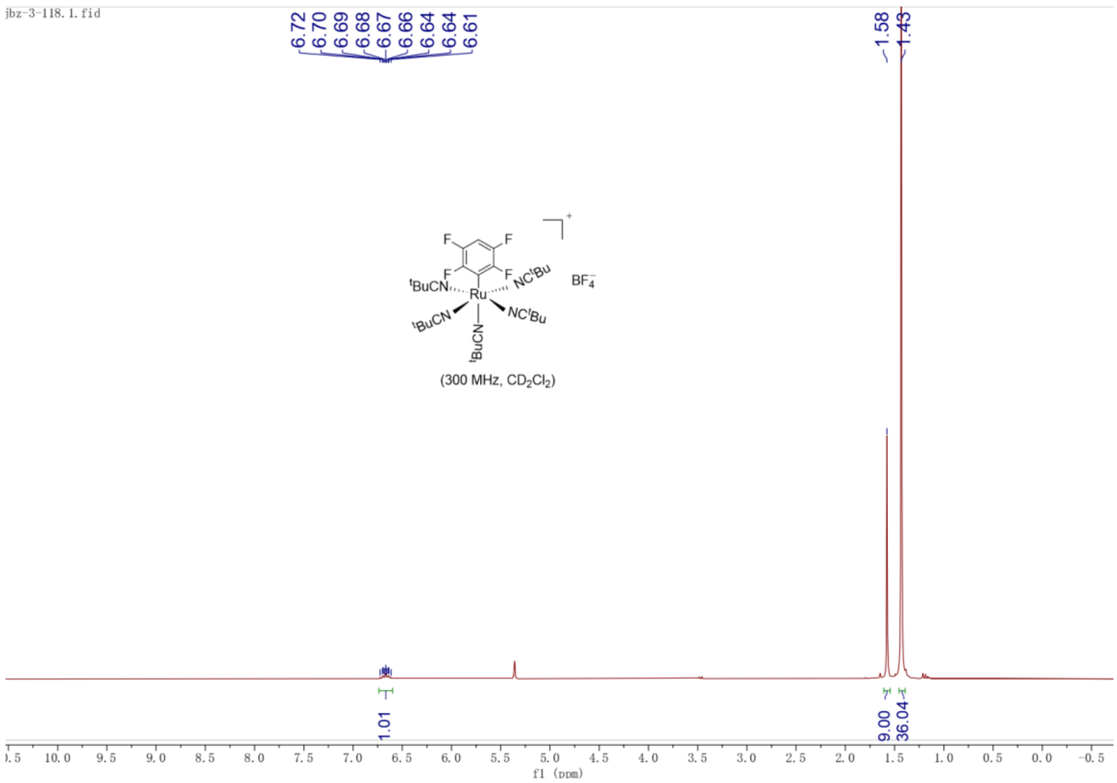

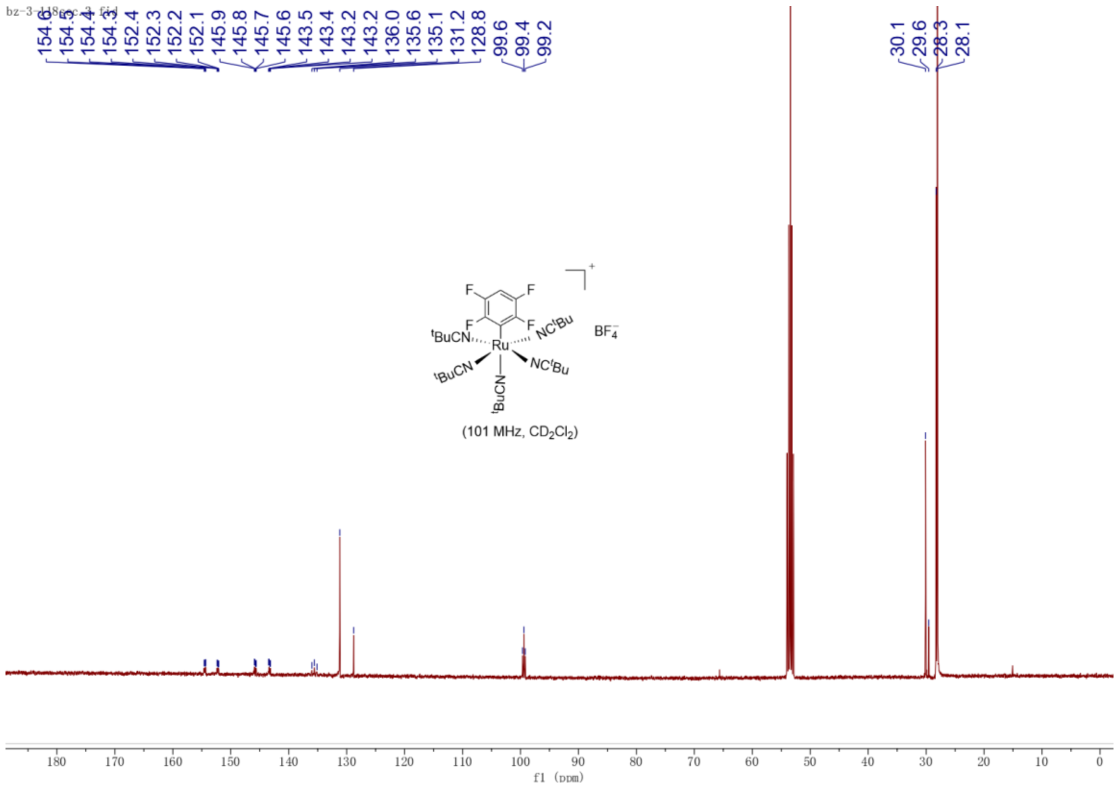

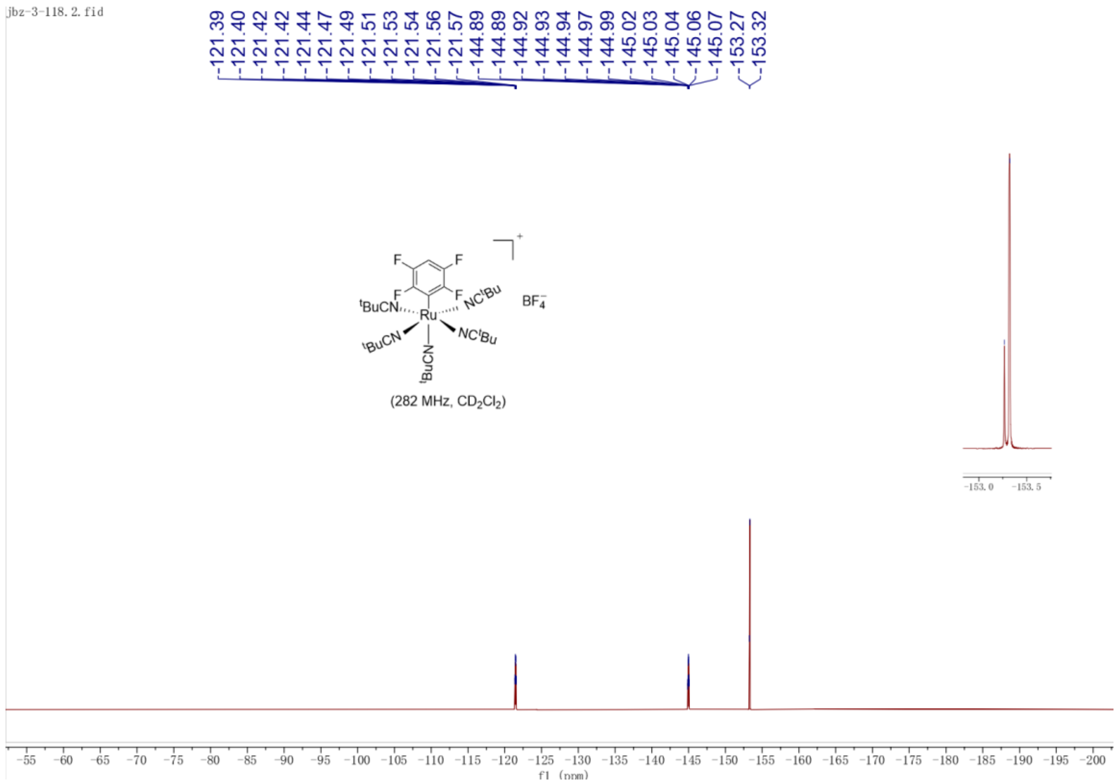
jbz-3-118.2.fid
-121.39
-121.40
-121.42
-121.42
-121.44
-121.47
-121.49
-121.51
-121.53
-121.54
-121.56
-121.57
-144.89
-144.89
-144.92
-144.93
-144.94
-144.97
-144.99
-145.02
-145.03
-145.04
-145.06
-145.07
-153.27
-153.32
(282 MHz, CD2Cl2)
BF4
f1 (ppm)

## 7. Computational Studies

### Computational methods

DFT optimizations were performed with Gaussian 16, Revision A.03 package.[6] Geometry optimizations were conducted at the PBE0[7,8] level of theory in combination with Grimme's D3 dispersion corrections with a Becke-Johnson damping scheme (D3BJ)[9,10] in gas phase. All atoms were described with a def2-SVP[11-14] basis set, while ruthenium was also described with a SDD pseudopotential.[15,16] Analytical frequency calculations were carried out at the same level of theory to confirm each optimized stationary point as an energy minimum (no imaginary frequencies) or a transition state (one imaginary frequency) and to further provide thermal and nonthermal corrections to the Gibbs free energy at 393.15 K and 1 atm. The electronic energies were further evaluated using the density functionals M06,[17] M06L,[18] and ωB97X-D[19] as implemented in Gaussian, and the double-hybrid functional PWPB95[20] as implemented in ORCA (Revision 5.0.1),[21] with the def2-TZVP[11-14] basis set. Solvent effects were taken into account using the implicit solvation model CPCM[22] in ORCA and the SMD model[23] in Gaussian for pivalonitrile ($\varepsilon$ = 20.09; $n_D$ = 1.377). When using the SMD model, the missing parameters for pivalonitrile were approximated by those of butanonitrile ($\varepsilon$ = 20.70) owing to their similar dielectric properties. All reported energies are based on gas phase Gibbs free energies with def2-SVP basis set for which the electronic energies were corrected at M06, M06L, ωB97X-D and PWPB95 functionals with a def2-TZVP level of theory including solvent effects. Transition states structures were visualized with the CYLview program.[24]

### Computational studies with dibenzothiophenium salt

Initially, we conducted a conformational analysis of the possible active species, as shown in Figure S3. The results indicate that the conformation with two coordinating acetonitrile ligands in *trans* positions exhibits the most stable geometry, which was identified as the active species used for subsequent mechanistic exploration. Calculations performed with various functionals reveal that the identity of the preferred active species is not significantly affected by the functional employed (Table S4).

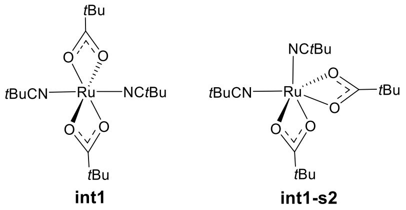


**Figure S3**. Conformational analysis of possible active species.

**Table S4.** Free energy (kcal mol$^{-1}$) of int1 isomers computed with different functionals.

| | **int1** | **int1-s2** |
|---|---|---|
| PWPB95-D3 | 0.0 | 2.5 |
| PBE0-D4 | 0.0 | 2.7 |
| PBE0-D3 | 0.0 | 2.7 |

| | | |
|---|---|---|
| M06-D3 | 0.0 | 4.0 |
| M06L-D3 | 0.0 | 3.1 |
| ωB97X-D | 0.0 | 3.6 |

Following coordination of substrate **1a** to the ruthenium center to form **int2**, multiple possible isomers were considered, as shown in Figure S4. The energy barriers for the subsequent C–H activation step associated with these isomers were also calculated. The first two correspond to the transformation of the acetate ligand from κ$^2$- to κ$^1$-coordination to accommodate the incorporation of the reacting arene **1a** (int2 and int2-s2). In the latter two, the arene **1a** occupies the position of the *t*BuCN ligand (int2-s3 and int2-s4). Among the different pathways, the first was the most favorable, exhibiting the lowest energy barrier. Furthermore, the energy spans obtained with different functionals indicate that the preferred pathway (path 1) is not sensitive to the choice of functional (Table S5).

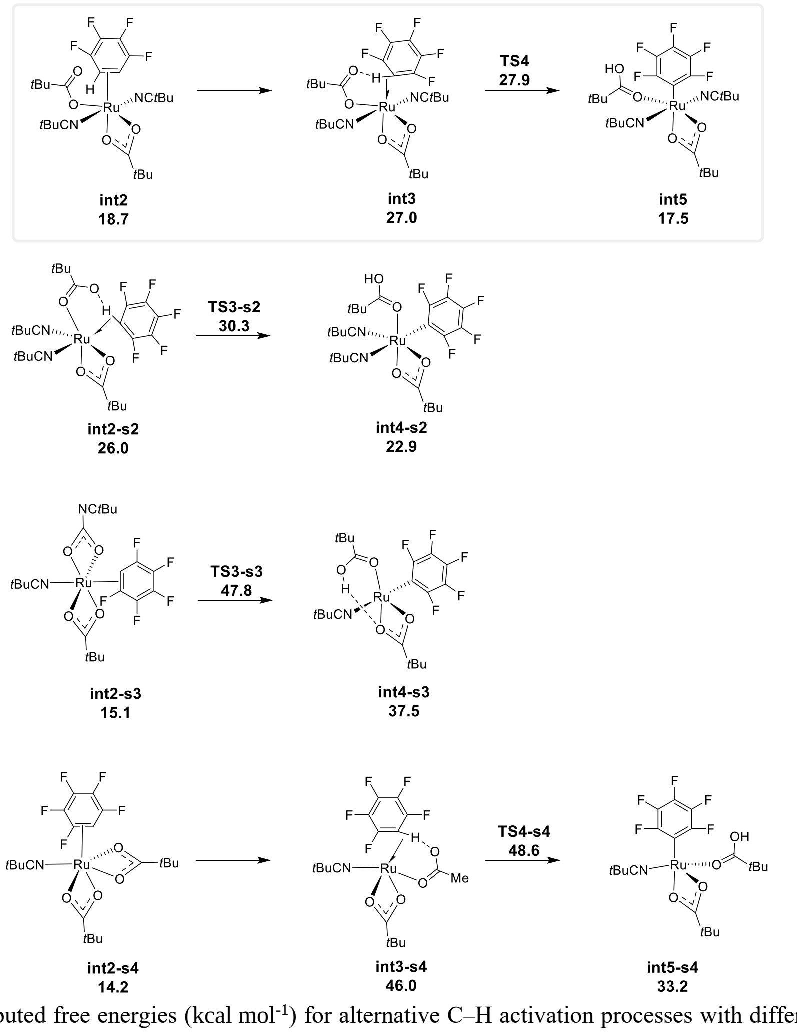


**Figure S4**. Computed free energies (kcal mol$^{-1}$) for alternative C–H activation processes with different isomers of int2 at the PWPB95/def2-TZVP-CPCM(*t*BuCN)//PBE0-D3(BJ)/def2-SVP level of theory.

**Table S5.** Comparison of free energies (kcal mol$^{-1}$) computed with different functionals for the energy barriers of C–H activation from int2 isomers.

| | **TS1** | **TS1-s2** | **TS1-s3** | **TS1-s4** |
|---|---|---|---|---|
| PWPB95-D3 | 27.9 | 30.3 | 47.8 | 48.6 |

| | | | | |
|---|---|---|---|---|
| PBE0-D4 | 26.5 | 27.2 | 41.9 | 41.3 |
| PBE0-D3 | 26.3 | 28.7 | 41.2 | 40.4 |
| M06-D3 | 29.4 | 30.7 | 44.4 | 43.9 |
| M06L-D3 | 30.4 | 33.7 | 45.0 | 42.6 |
| ωB97X-D | 31.6 | 33.2 | 41.9 | 42.0 |

In the two-electron oxidative addition step, two possible isomers were examined (Figure S5). Computational results demonstrate that the isomer in which the phenyl group is oriented upward is energetically preferred (black line), and this conclusion is also supported by calculations employing different functionals (Table S6).

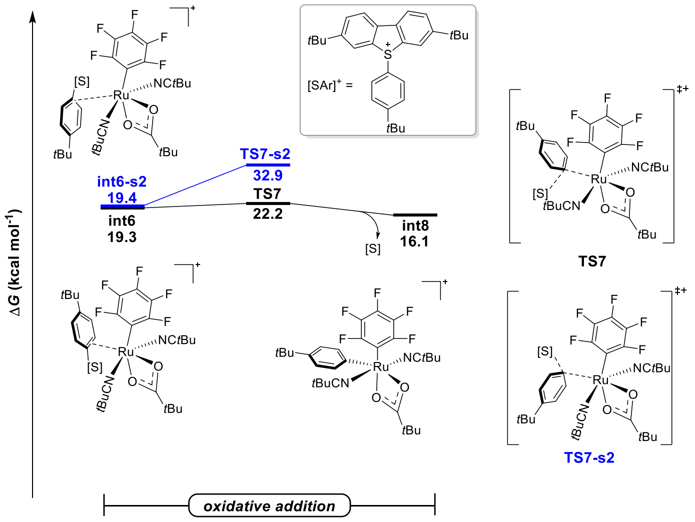


**Figure S5**. Computed free energies (kcal mol$^{-1}$) for alternative C–H activation processes with different isomers of int2 at the PWPB95/def2-TZVP-CPCM(*t*BuCN)//PBE0-D3(BJ)/def2-SVP level of theory.

**Table S6.** Comparison of free energies (kcal mol$^{-1}$) computed with different functionals for the energy barriers of C–H activation from int2 isomers.

| | int6 | TS7 | int6-s2 | TS7-s2 |
|---|---|---|---|---|
| PWPB95-D2 | 19.3 | 22.2 | 19.4 | 32.9 |
| PBE0-D4 | 25.4 | 29.7 | 26.9 | 42.2 |
| PBE0-D3 | 24.4 | 28.8 | 25.9 | 41.0 |
| M06-D3 | 18.5 | 19.7 | 19.6 | 30.7 |
| M06L-D3 | 28.2 | 24.2 | 29.8 | 34.8 |
| ωB97X-D | 30.0 | 35.5 | 30.8 | 45.9 |

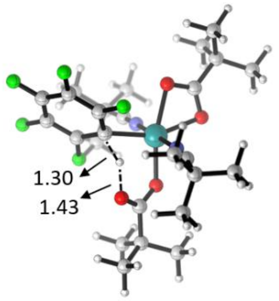


**Figure S6**. Computed transition state structure for the C–H activation step with dibenzothiophenium salt S9 (**TS4**). Relevant bond lengths in the transition states are given in Å.

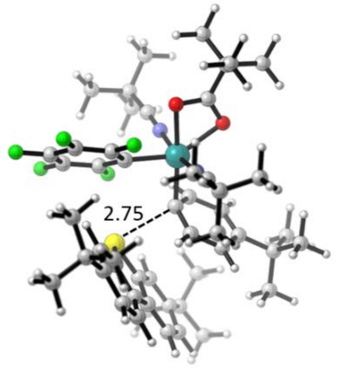


**Figure S7**. Computed transition state structure for the oxidative addition step with dibenzothiophenium salt S9 (**TS7**). The key distance is provided in Å.

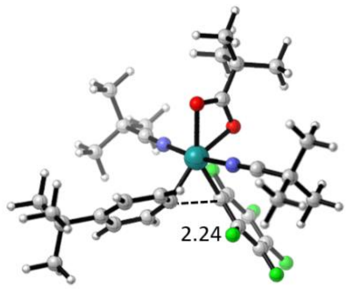


**Figure S8**. Computed transition state structure for the reductive elimination step with dibenzothiophenium salt S9 (**TS9**). The key distance is provided in Å.

Following the C–H activation step, coordination of the dibenzothiophenium salt may proceed *via* either a cationic pathway (in the absence of counterions, as illustrated in the manuscript) or a neutral pathway, as depicted in Figure S9. However, the neutral pathway, whether involving pivalate or triflate as counterions, exhibited higher energies compared to the **int6**. As a result, this pathway was not pursued further in our investigation.

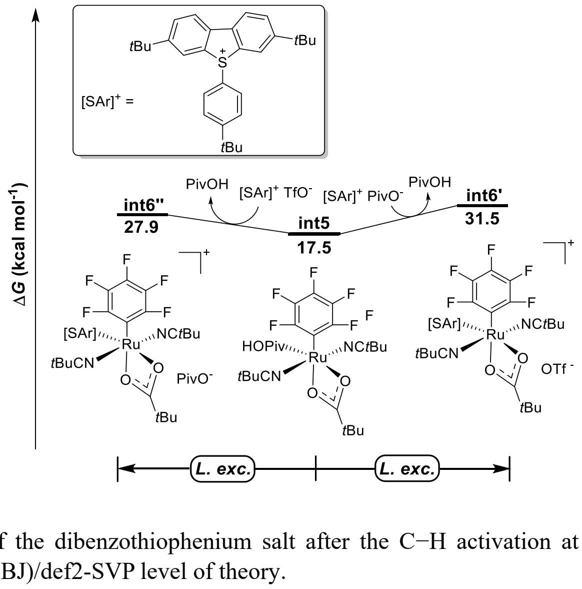


**Figure S9**. Coordianion of the dibenzothiophenium salt after the C−H activation at the PWPB95/def2-TZVP-CPCM(*t*BuCN)//PBE0-D3(BJ)/def2-SVP level of theory.

In addition to the oxidative addition pathway, the activation of dibenzothiophenium salt through the radical mechanism was also explored, which was shown in Figure S10. The dissociative electron transfer (DET) pathway was calculated to have a high energy

barrier of 47.8 kcal mol$^{-1}$ for the generation of *tert*-butylbenzyl radical and *tert*-butyl-substituted dibenzothiophene, which is not feasible under the optimal experimental conditions (120 °C). Alternative inner-sphere electron transfer (ISET) pathways, whether proceeding *via* an open-shell singlet or a triplet state, were found to involve even higher energy barriers and were therefore not considered further.

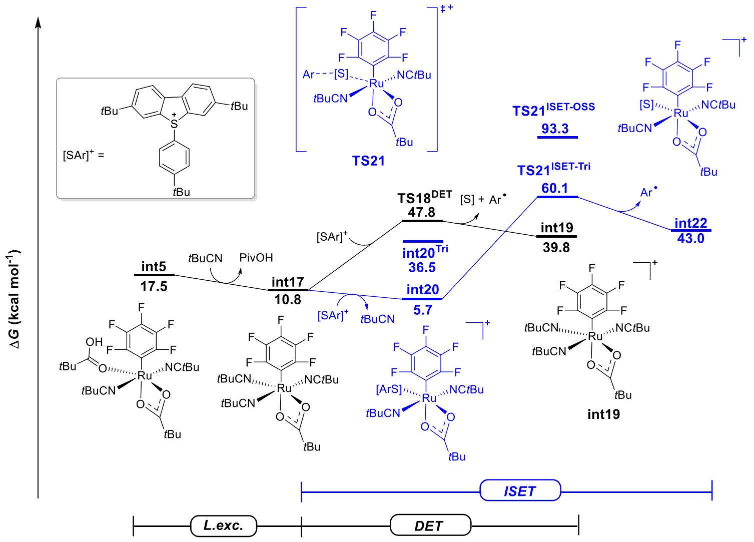

**Figure S10**. The activation of dibenzothiophenium salt following the radical mechanism at the PWPB95/def2-TZVP-CPCM(*t*BuCN)//PBE0-D3(BJ)/def2-SVP level of theory.

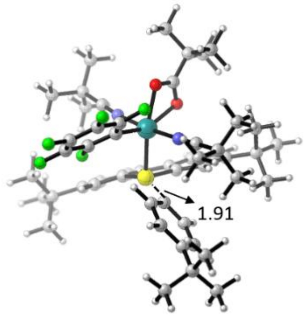


**Figure S11**. Computed transition state structure for the single-electron transfer step at the triplet state with dibenzothiophenium salt S9 (**TS21$^{ISET\text{-}Tri}$**). The key distance is provided in Å.

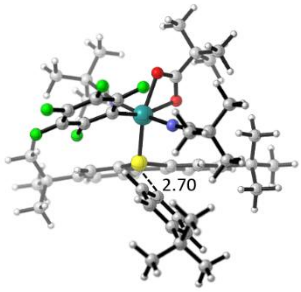


**Figure S12.** Computed transition state structure for the single-electron transfer step at the open-shell singlet state with dibenzothiophenium salt S9 (**TS21$^{ISET\text{-}OSS}$**). The key distance is provided in Å.

## Computational studies with *tert*-butylbenzyl bromide

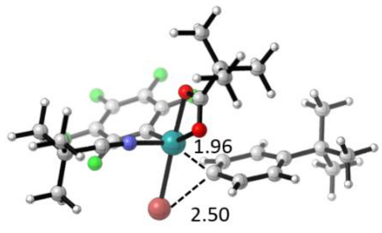


**Figure S13**. Computed transition state structure for the oxidative addition step with *tert*-butylbenzyl bromide (**TS24**). The key distances are provided in Å.

The activation of *tert*-butylbenzyl bromide following the radical pathway, either through dissociative or inner-sphere electron transfer, was proven to be unfeasible as shown below.

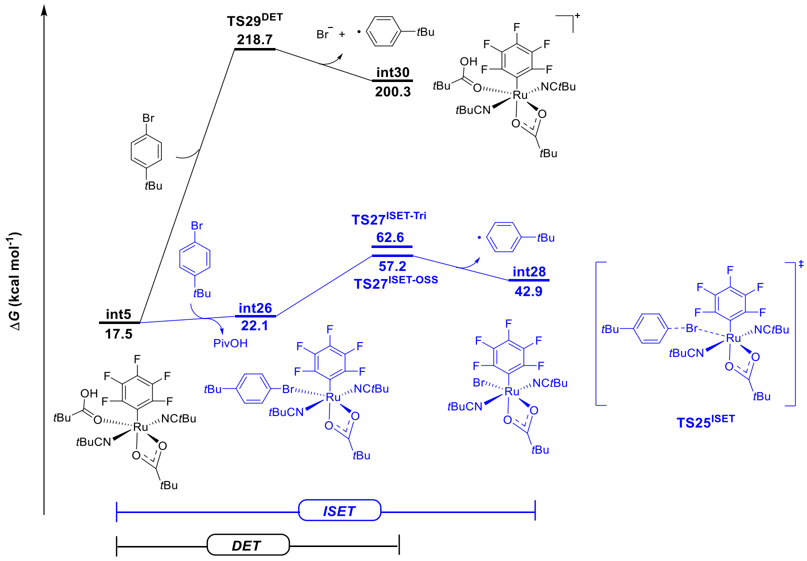


**Figure S14**. The activation of *tert*-butylbenzyl bromide following the radical mechanism at the PWPB95/def2-TZVP-CPCM(*t*BuCN)//PBE0-D3(BJ)/def2-SVP level of theory.

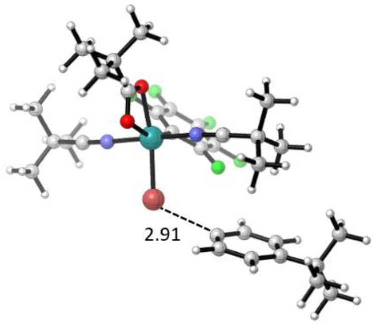


**Figure S15.** Computed transition state structure for the single-electron transfer step at the open-shell singlet state with *tert*-butylbenzyl bromide (**TS27$^{\text{ISET-OSS}}$**). The key distance is provided in Å.

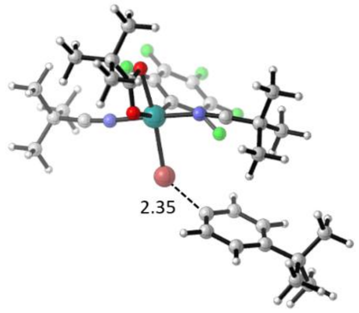


**Figure S16**. Computed transition state structure for the single-electron transfer step at the triplet state with *tert*-butylbenzyl bromide (**TS27$^{\mathbf{ISET\text{-}Tri}}$**). The key distance is provided in Å.

## Computational Studies with phenyl(tetrahydro)thiophenium triflate S1 and arylthianthrenium salt S2

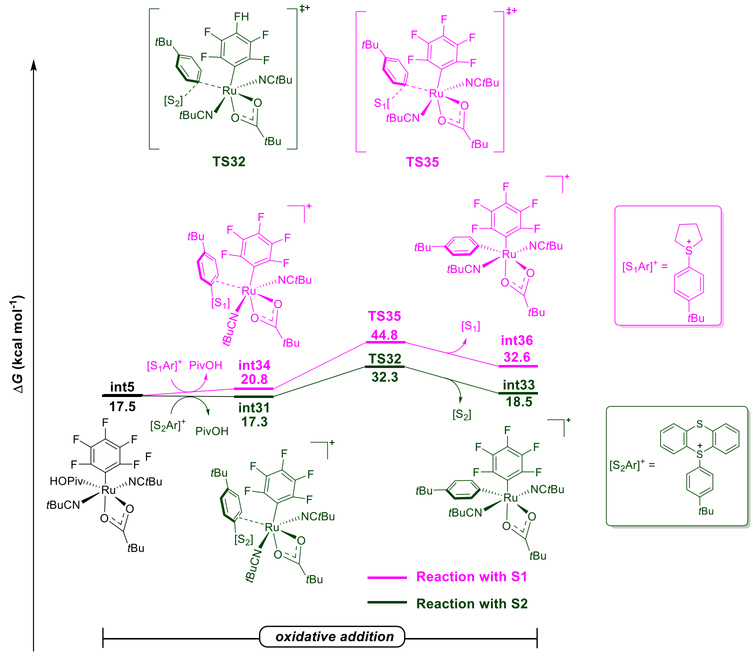


**Figure S17**. Oxidative addition pathway with phenyl(tetrahydro)thiophenium triflate **S1** and arylthianthrenium salt **S2** at the PWPB95/def2-TZVP-CPCM(*t*BuCN)//PBE0-D3(BJ)/def2-SVP level pf theory.

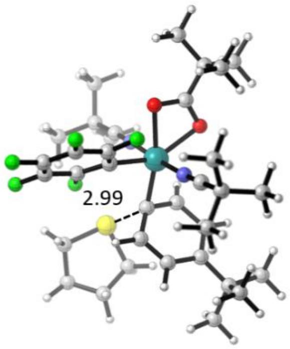


**Figure S18**. Computed transition state structure for the oxidative addition step with phenyl(tetrahydro)thiophenium triflate S1 (**TS32**). The key distance is provided in Å.

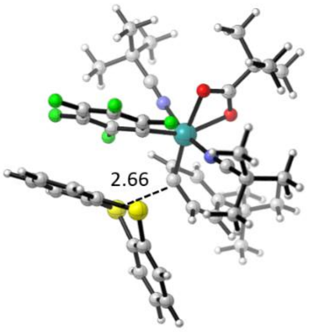


**Figure S19**. Computed transition state structure for the oxidative addition step with arylthianthrenium salt S2 (**TS35**). The key distance is provided in Å.

**Table S7.** Calculated electronic energies at the PWPB95-D3/def2-TZVP-CPCM(*t*BuCN) level of theory and Gibbs free energies for all structures (all in Hartree).

| Structure | Electronic Energy | Total Gibbs Free Energy |
|---|---|---|
| **int1** | -1288.723275 | -1288.297127 |
| **int1-s2** | -1288.721267 | -1288.293082 |
| **int2(int11)** | -2017.057226 | -2016.580957 |
| **int3** | -2017.038341 | -2016.567671 |
| **TS4** | -2017.034401 | -2016.566346 |
| **int5** | -2017.054291 | -2016.582872 |
| **int2-s2** | -2017.044422 | -2016.569254 |
| **TS3-s2** | -2017.032177 | -2016.562436 |
| **int4-s2** | -2017.049611 | -2016.574142 |
| **int2-s3** | -1766.443346 | -1766.081423 |
| **TS3-s3** | -1766.383978 | -1766.029541 |
| **int4-s3** | -1766.404925 | -1766.045900 |
| **int2-s4** | -1766.444743 | -1766.082821 |
| **int3-s4** | -1766.387825 | -1766.032365 |
| **TS4-s4** | -1766.384935 | -1766.028250 |
| **int5-s4** | -1766.407887 | -1766.052649 |
| **int6** | -3233.115597 | -3232.235335 |
| **int6’** | -4194.726825 | -4193.833986 |
| **int6”** | -3579.574753 | -3578.576782 |
| **TS7** | -3233.092344 | -3232.213873 |
| **int6-s2** | -3233.115597 | -3232.235335 |
| **TS7-s2** | -3233.092344 | -3232.213873 |
| **int8** | -2058.639125 | -2058.112278 |
| **TS9** | -2058.635334 | -2058.107617 |
| **int10** | -2058.694360 | -2058.159321 |
| **int12** | -2112.899823 | -2112.423032 |
| **TS13** | -2112.885691 | -2112.407517 |
| **int14** | -2112.889683 | -2112.410039 |
| **TS15** | -2112.862891 | -2112.388723 |
| **int16** | -2112.875448 | -2112.396546 |
| **int17** | -1920.738847 | -1920.280269 |
| **int19** | -1920.559839 | -1920.104793 |
| **int20** | -3233.135125 | -3232.257167 |
| **int20$^{\mathbf{Tri}}$** | -3233.081067 | -3232.208180 |
| **TS21$^{\mathbf{ISET\text{-}OSS}}$** | -3233.047177 | -3232.170496 |
| **TS21$^{\mathbf{ISET\text{-}Tri}}$** | -3233.054211 | -3232.117557 |
| **int22** | -2844.409552 | -2843.716681 |
| **int23** | -4382.338102 | -4381.933466 |
| **TS24** | -4382.340727 | -4381.932521 |
| **int25** | -4382.341171 | -4381.937427 |
| **int26** | -4632.981956 | -4632.459317 |
| **TS27$^{\mathbf{ISET\text{-}OSS}}$** | -4632.918740 | -4632.402660 |
| **TS27$^{\mathbf{ISET\text{-}Tri}}$** | -4632.908851 | -4632.394890 |
| **int28** | -4244.282104 | -4243.944795 |

| | | |
|---|---|---|
| **int30** | -2016.877150 | -2016.407632 |
| **int31** | -2614.010897 | -2613.376373 |
| **TS32** | -2613.966633 | -2613.338111 |
| **int33** | -2058.639126 | -2058.112276 |
| **int34** | -3316.905550 | -3316.231613 |
| **TS35** | -3316.878876 | -3316.207702 |
| **int36** | -2058.638474 | -2058.111349 |
| **Dibenzothiophenium salt S9** | -1562.970026 | -1562.474034 |
| **PivOH** | -346.919477 | -346.818315 |
| **Pentafluorobenzen** | -728.325830 | -728.313471 |
| ***t*BuCN** | -250.592636 | -250.505154 |
| ***tert*-Butylbenzyl radical** | -388.631207 | -388.481059 |
| ***tert*-Butylbenzyl bromide** | -2962.851332 | -2962.702425 |
| **Phenyl(tetrahydro)thiophenium triflate S1** | -943.873158 | -943.617262 |
| **Arylthianthrenium salt S2** | -1646.763521 | -1646.466949 |

**Table S8.** Calculated electronic energies at the PBE0-D4/def2-TZVP-SMD(*t*BuCN) level of theory and Gibbs free energies for all structures (all in Hartree).

| Structure | Electronic Energy | Total Gibbs Free Energy |
|---|---|---|
| **int1** | -1288.388170 | -1287.962022 |
| **int1-s2** | -1288.385956 | -1287.957771 |
| **TS4** | -2016.367499 | -2015.899444 |
| **TS3-s2** | -2016.368121 | -2015.898380 |
| **TS3-s3** | -1765.819489 | -1765.465052 |
| **TS4-s4** | -1765.822601 | -1765.465916 |
| **int6** | -3232.099690 | -3231.222957 |
| **TS7** | -3232.088570 | -3231.216168 |
| **int6-s2** | -3232.100809 | -3231.220547 |
| **TS7-s2** | -3232.074665 | -3231.196194 |

**Table S9.** Calculated electronic energies at the PBE0-D3/def2-TZVP-SMD(*t*BuCN) level of theory and Gibbs free energies for all structures (all in Hartree).

| Structure | Electronic Energy | Total Gibbs Free Energy |
|---|---|---|
| **int1** | -1288.387960 | -1287.961812 |
| **int1-s2** | -1288.385741 | -1287.957556 |
| **TS4** | -2016.366999 | -2015.898944 |
| **TS3-s2** | -2016.364715 | -2015.894974 |
| **TS3-s3** | -1765.818661 | -1765.464224 |
| **TS4-s4** | -1765.822124 | -1765.465439 |
| **int6** | -3232.099727 | -3231.222994 |
| **TS7** | -3232.088516 | -3231.216114 |
| **int6-s2** | -3232.100877 | -3231.220615 |
| **TS7-s2** | -3232.075038 | -3231.196567 |

**Table S10.** Calculated electronic energies at the M06-D3/def2-TZVP-SMD(*t*BuCN) level of theory and Gibbs free energies for all structures (all in Hartree).

| Structure | Electronic Energy | Total Gibbs Free Energy |
|---|---|---|
| **int1** | -1288.91737873 | -1288.49123073 |
| **int1-s2** | -1288.91310783 | -1288.48492283 |
| **TS4** | -2017.28929138 | -2016.82123638 |
| **TS3-s2** | -2017.28883312 | -2016.81909212 |
| **TS3-s3** | -1766.63294519 | -1766.27850819 |
| **TS4-s4** | -1766.63587945 | -1766.27919445 |
| **int6** | -3233.39958273 | -3232.52284973 |
| **TS7** | -3233.39333989 | -3232.52093789 |
| **int6-s2** | -3233.40134918 | -3232.52108718 |
| **TS7-s2** | -3233.38194303 | -3232.50347203 |

**Table S11.** Calculated electronic energies at the M06L-D3/def2-TZVP-SMD(*t*BuCN) level of theory and Gibbs free energies for all structures (all in Hartree).

| Structure | Electronic Energy | Total Gibbs Free Energy |
|---|---|---|
| **int1** | -1289.64922405 | -1289.22307605 |
| **int1-s2** | -1289.64631016 | -1289.21812516 |
| **TS4** | -2018.23787567 | -2017.76982067 |
| **TS3-s2** | -2018.23432809 | -2017.76458709 |
| **TS3-s3** | -1767.42717929 | -1767.07274229 |
| **TS4-s4** | -1767.43329908 | -1767.07661408 |
| **int6** | -3234.93760383 | -3234.06087083 |
| **TS7** | -3234.93965207 | -3234.06725007 |
| **int6-s2** | -3234.93854751 | -3234.05828551 |
| **TS7-s2** | -3234.92872318 | -3234.05025218 |

**Table S12.** Calculated electronic energies at the ωB97X-D/def2-TZVP-SMD(*t*BuCN) level of theory and Gibbs free energies for all structures (all in Hartree).

| Structure | Electronic Energy | Total Gibbs Free Energy |
|---|---|---|
| **int1** | -1289.40182293 | -1288.97567493 |
| **int1-s2** | -1289.39817735 | -1288.96999235 |
| **TS4** | -2017.87657517 | -2017.40852017 |
| **TS3-s2** | -2017.87570401 | -2017.40596301 |
| **TS3-s3** | -1767.11650909 | -1766.76207209 |
| **TS4-s4** | -1767.11871829 | -1766.76203329 |
| **int6** | -3234.42168662 | -3233.54495362 |
| **TS7** | -3234.40856723 | -3233.53616523 |
| **int6-s2** | -3234.42380938 | -3233.54354738 |
| **TS7-s2** | -3234.39804589 | -3233.51957489 |

## 8. Data Science Analysis

### Details of descriptors

Initially, four geometric descriptors, including bond lengths, dihedral angles and the vibrational stretching frequency around the C–S bond, were extracted from the DFT-optimized geometries at the PBE0-D3(BJ)/def2-SVP level of theory. A series of steric features, such as Sterimol parameters[25] ($B_{min}$, $B_{max}$, and L) and buried volume[26] for the reaction active space were calculated using DFT-based Steric Parameters (DBSTEP)[27] python package based on the optimized arylsulfonium salts. Further, the natural bond orbitals (NBO) atomic charges were obtained with the use of Gaussian NBO version 6.0[28] from the single point calculations at the PBE0-D3(BJ)/def2-TZVP level of theory. Frontier molecular orbital (HOMO, LUMO) energies and bond-dissociation energies were obtained from the single point calculations considering the solvent effect at the PBE0-D3(BJ)/def2-TZVP+SMD(*t*BuCN) level of theory based on the optimized structures. All cationic arylsulfonium salts were shown in Scheme S1. Full details of the used molecular descriptors are elaborated in Table S13.

The bond dissociation energy of C–S bond in dibenzothiophenium salt was calculated as follows:

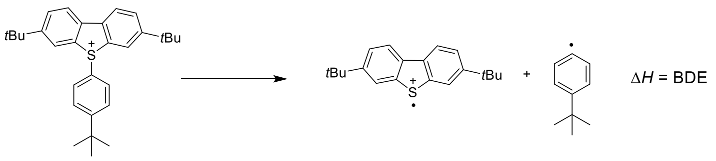


**Figure S20**. Bond dissociation energy (BDE) of the C–S bond in the dibenzothiophenium salt.

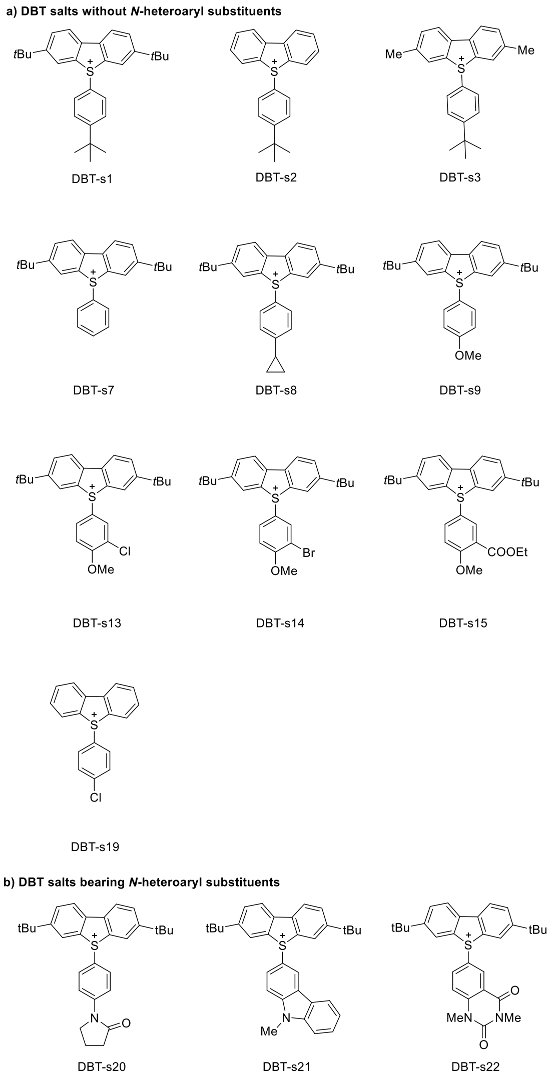


**Scheme S1**. Considered arylsulfonium salts used for MVLR studies.

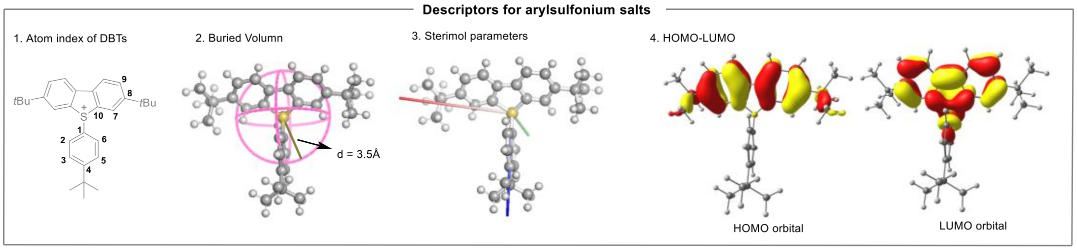


**Figure S21**. Arylsulfonium salts featurization. Here dibenzothiophenium salt was used for illustration.

**Table S13.** List of all the features considered in the MVLR studies.

| Features | Unit | Description |
|---|---|---|
| **bd**_$_{C1-S}$ | Å | Bond length of the indexed atoms |
| **da**_$_{C10_S_C1_C2}$ | ° | Dihedral angle between the indexed atoms |
| **da**_$_{C10_S_C1_C6}$ | ° | Dihedral angle between the indexed atoms |
| **VF**$_{C1-S}$ | $cm^{-1}$ | Vibrational stretching frequency of the indexed bond |

| | | |
|---|---|---|
| **$q_{C1}$, $q_{C2}$, $q_{C3}$, $q_{C4}$, $q_{C5}$, $q_{C6}$, $q_{C7}$, $q_{C8}$, $q_{C9}$, $q_S$** | e | NBO charges of indexed carbon atoms at arenes |
| **$B_{min}$, $B_{max}$, L** | Å | Sterimol parameter of sulfur atom toward carbon atom 1 |
| **$V_{bur(S)}$** | % | Percentage buried volume at the sulfur center (at default 3.5Å radius) |
| **$V_{bur(C1)}$** | % | Percentage buried volume at the C1 center (at default 3.5Å radius) |
| **$E_{\_HOMO}$** | a.u. | The energy of highest occupied molecular orbital of arylsulfonium salts |
| **$E_{\_LUMO}$** | a.u. | The energy of lowest unoccupied molecular orbital of arylsulfonium salts |
| **$BDE_{\_C1\text{-}S}$** | kcal mol$^{-1}$ | The homolytic bond dissociation energy of the reacting C1–S bond in arylsulfonium salts |

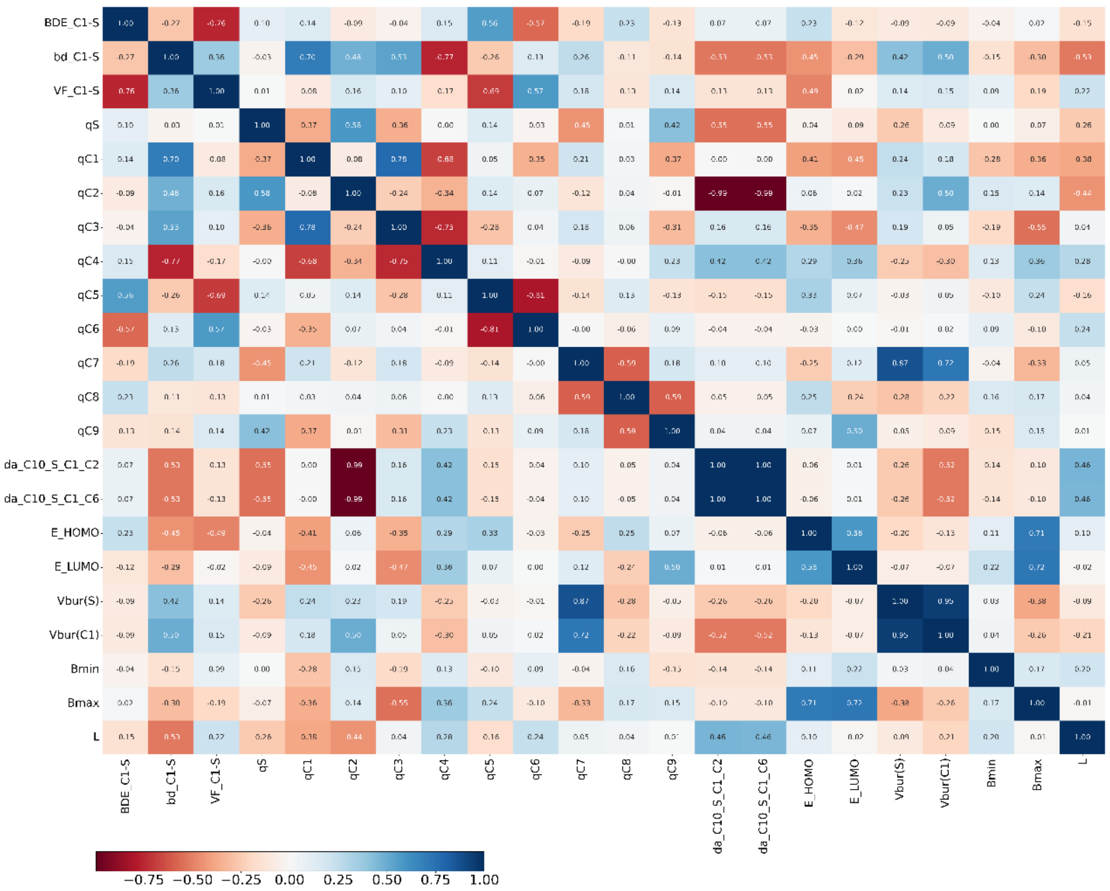


**Figure S22**. Pearson correlation heatmap of the training dataset. The color scale bar represents the magnitude and direction of the correlations between the features, with values ranging from -1 (dark red: strong negative correlation) to +1 (dark blue: strong positive correlation). Each cell displays the correlation coefficient for the respective feature pair.

Through the Pearson correlation analysis for the training data, the highly correlated features (Pearson correlation coefficient > 0.9) were eliminated for further feature analysis.

## Multivariate linear regression modeling

Multivariate linear regression (MVLR) analysis were performed using our previously reported Python scripts[29] and scikit-learn libraries.[30] With the reduced feature dimension, a best subset selection method was employed for model development. To assess the robustness of the models, leave-one-out (LOO) method was implemented.

Model performance was evaluated using mean absolute error (MAE) and the coefficient of determination ($R^2$) as key metrics. To avoid the risk of overfitting, particularly with smaller data samples, a maximum of four features was selected as the criterion. The top-performing models (M1, M2, M3, and M4) from each feature size (1 to 4) were presented in Tables S14 to S17. According to the $R^2$ value and the number of features, M4-1, M4-2 and M4-3 were identified as the best overall models, with MAE values of 8.35% and 15.43% for LOO cross-validation. However, the $R^2$ value (0.67) was not sufficiently high to demonstrate the robustness of this model. As shown in Table S18, a closer examination of the predicted versus experimental yields revealed that the model was not compatible with substrates bearing *N*-heteroaryl substituents, as exemplified by s20, s21, and s22. Accordingly, these three were regarded as a distinct class and were excluded from subsequent modeling. The revised models excluding these three data are summarized in Tables S19–S22.

**Table S14**. Summary of measured and predicted yields for the best five performing models based on a single feature (M1).

| | $R^2$ | MAE (%) | LOO MAE (%) | Features | Coefficients |
|---|---|---|---|---|---|
| M1-1 | 0.31 | 12.29 | 13.47 | $V_{bur(S)}$ | -6.99 |
| M1-2 | 0.28 | 13.30 | 14.83 | $B_{max}$ | 12.14 |
| M1-3 | 0.19 | 14.12 | 19.13 | $q_{C7}$ | -163.26 |
| M1-4 | 0.09 | 14.53 | 16.26 | $E_{\_LUMO}$ | 1790.74 |
| M1-5 | 0.07 | 14.18 | 16.44 | $q_{C9}$ | -43.64 |

**Table S15**. Summary of measured and predicted yields for the best five performing models based on two features (M2).

| | $R^2$ | MAE (%) | LOO MAE (%) | Features | Coefficients |
|---|---|---|---|---|---|
| M2-1 | 0.47 | 11.30 | 18.58 | $V_{bur(S)}$<br>$q_S$ | -8.17<br>-1164.06 |
| M2-2 | 0.43 | 11.64 | 13.54 | $B_{max}$<br>$V_{bur(S)}$ | 8.43<br>-5.21 |
| M2-3 | 0.42 | 11.50 | 17.08 | $q_{C7}$<br>$q_S$ | -246.50<br>-1519.31 |
| M2-4 | 0.40 | 10.94 | 18.30 | $q_{C9}$<br>$V_{bur(S)}$ | -48.37<br>-7.19 |
| M2-5 | 0.40 | 11.91 | 14.52 | $q_{C9}$<br>$B_{max}$ | -57.06<br>13.24 |

**Table S16**. Summary of measured and predicted yields for the best five performing models based on three features (M3).

| | $R^2$ | MAE (%) | LOO MAE (%) | Features | Coefficients |
|---|---|---|---|---|---|
| M3-1 | 0.61 | 8.94 | 15.24 | $V_{bur(S)}$<br>$q_{C9}$<br>$E_{\_LUMO}$ | -6.90<br>-92.28<br>3251.57 |
| M3-2 | 0.59 | 8.83 | 17.61 | $q_S$<br>$q_{C2}$<br>$V_{bur(S)}$ | -2090.64<br>86.52<br>-10.47 |
| M3-3 | 0.54 | 10.29 | 17.76 | $q_{C9}$<br>$V_{bur(S)}$<br>$B_{max}$ | -56.74<br>-5.19<br>9.55 |
| M3-4 | 0.54 | 10.35 | 17.44 | $q_S$<br>$V_{bur(S)}$<br>$B_{max}$ | -1004.21<br>-6.58<br>6.78 |
| M3-5 | 0.53 | 10.35 | 16.04 | $q_S$<br>$q_{C7}$<br>$E_{\_LUMO}$ | 1491.35<br>-260.96<br>2078.26 |

**Table S17**. Summary of measured and predicted yields for the best five performing models based on four features (M4).

| | $R^2$ | MAE (%) | LOO MAE (%) | Features | Coefficients |
|---|---|---|---|---|---|
| M4-1 | 0.67 | 8.35 | 15.43 | $q_{C9}$ | -107.70 |
| | | | | $E_{\_HOMO}$ | 3732.37 |
| | | | | $E_{\_HOMO\text{-}LUMO}$ | 4598.69 |
| | | | | $V_{bur(S)}$ | -7.58 |
| M4-2 | 0.67 | 8.35 | 15.43 | $q_{C9}$ | -107.70 |
| | | | | $E_{\_LUMO}$ | 3732.37 |
| | | | | $E_{\_HOMO\text{-}LUMO}$ | 866.32 |
| | | | | $V_{bur(S)}$ | -7.58 |
| M4-3 | 0.67 | 8.35 | 15.43 | $q_{C9}$ | -107.70 |
| | | | | $E_{\_HOMO}$ | 866.32 |
| | | | | $E_{\_LUMO}$ | 4598.69 |
| | | | | $V_{bur(S)}$ | -7.58 |
| M4-4 | 0.67 | 8.41 | 19.82 | $q_S$ | -2326.71 |
| | | | | $q_{C2}$ | 100.10 |
| | | | | $q_{C6}$ | -167.05 |
| | | | | $V_{bur(S)}$ | -11.10 |
| M4-5 | 0.66 | 8.24 | 11.73 | $q_{C8}$ | -41.70 |
| | | | | $q_{C9}$ | -124.40 |
| | | | | $E_{\_LUMO}$ | 3354.14 |
| | | | | $V_{bur(S)}$ | -8.11 |

**Table S18.** Comparison of the predicted yields and experimental yields using the best-performing model based on the four descriptors (M4-1) for the 22 samples.

| | Experimental yield | Predicted yield | Deviation |
|---|---|---|---|
| DBT-s1 | 85 | 76.3 | 8.7 |
| DBT-s2 | 67 | 63.2 | 3.8 |
| DBT-s3 | 69 | 64.0 | 5.0 |
| DBT-s4 | 17 | 17.4 | -0.4 |
| DBT-s5 | 40 | 39.8 | 0.2 |
| DBT-s6 | 35 | 38.1 | -3.1 |
| DBT-s7 | 81 | 71.3 | 9.7 |
| DBT-s8 | 80 | 77.4 | 2.6 |
| DBT-s9 | 88 | 79.7 | 8.3 |
| DBT-s10 | 75 | 77.3 | -2.3 |
| DBT-s11 | 63 | 64.6 | -1.6 |
| DBT-s12 | 70 | 74.0 | -4.0 |
| DBT-s13 | 78 | 62.0 | 16.0 |
| DBT-s14 | 66 | 60.7 | 5.3 |
| DBT-s15 | 75 | 65.7 | 9.3 |
| DBT-s16 | 52 | 69.4 | -17.4 |
| DBT-s17 | 51 | 48.7 | 2.3 |
| DBT-s18 | 79 | 59.6 | 19.4 |
| DBT-s19 | 45 | 54.4 | -9.4 |
| **DBT-s20** | 51 | 69.2 | **-18.2** |
| **DBT-s21** | 52 | 71.6 | **-19.6** |
| **DBT-s22** | 47 | 61.5 | **-14.5** |

With the reduced samples (19 samples in total), M3-1 was selected as the best overall model with $R^2$ value of 0.79, MAE value of 5.62% and 14.60% for LOO cross-validation (Table S21). The addition of a fourth descriptor provided only marginal improvement ($R^2$ = 0.86, MAE = 5.25%) but with a decreased LOO MAE value, which was listed in Table S22. Therefore, according to both the predictive accuracy and model simplicity, M3-1 was selected as the best overall model, with the most relevant features being $q_S$, $q_{C2}$, and $V_{Bur(S)}$.

To simplify interpretability, the selected model was further refined using scikit-learn's *StandardScaler* function, which enabled calculation of coefficient values that reflect the relative importance of each feature on a standardized scale.

**Table S19**. Summary of measured and predicted yields for the best five performing models based on a single feature

(M1).

| | $R^2$ | MAE (%) | LOO MAE (%) | Features | Coefficients |
|---|---|---|---|---|---|
| M1-1 | 0.38 | 11.16 | 12.30 | $V_{bur(S)}$ | -7.42 |
| M1-2 | 0.37 | 12.22 | 13.92 | $B_{max}$ | 13.92 |
| M1-3 | 0.25 | 13.34 | 18.70 | $q_{C7}$ | -181.15 |
| M1-4 | 0.21 | 13.85 | 15.68 | $E_{\_HOMO}$ | 1662.54 |
| M1-5 | 0.12 | 14.85 | 16.75 | $E_{\_HOMO\text{-}LUMO}$ | -1705.58 |

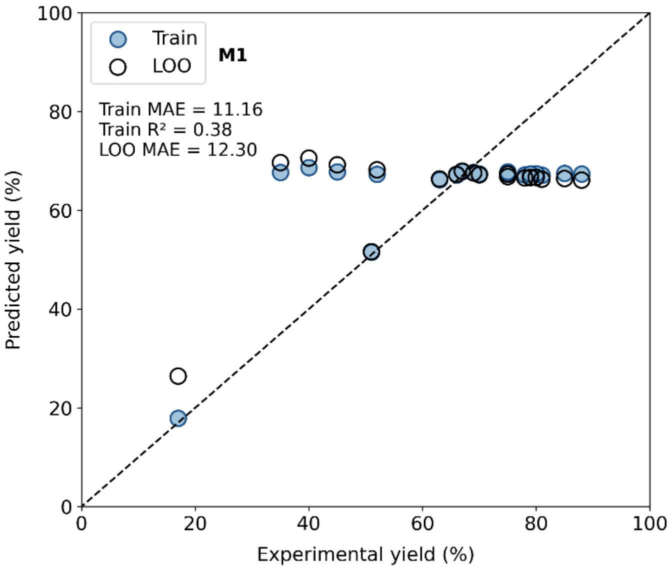


**Figure S23**. Measured vs. predicted yield plot for the best model with one feature contribution (M1-1 in Table S19).

**Table S20**. Summary of measured and predicted yields for the best five performing models based on two features (M2).

| | $R^2$ | MAE (%) | LOO MAE (%) | Features | Coefficients |
|---|---|---|---|---|---|
| M2-1 | 0.61 | 8.78 | 17.78 | $V_{bur(S)}$<br>$q_S$ | -8.95<br>-1397.01 |
| M2-2 | 0.60 | 8.70 | 14.40 | $q_S$<br>$q_{C7}$ | -1878.89<br>-291.14 |
| M2-3 | 0.54 | 10.24 | 12.80 | $V_{bur(S)}$<br>$B_{max}$ | -5.43<br>9.77 |
| M2-4 | 0.52 | 10.39 | 15.57 | $q_{C9}$<br>$B_{max}$ | -63.05<br>14.93 |
| M2-5 | 0.48 | 9.53 | 20.78 | $q_{C9}$<br>$V_{bur(S)}$ | -52.39<br>-7.64 |

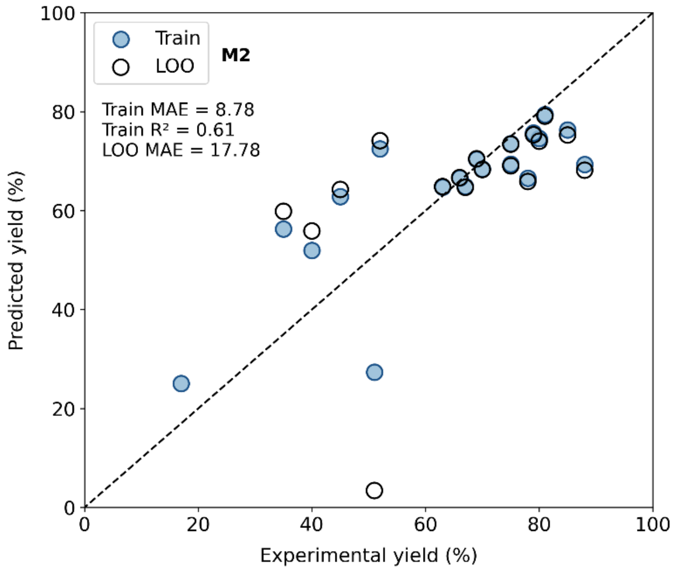


**Figure S24**. Measured vs. predicted yield plot for the best model with two features contribution (M2-1 in Table S20).

**Table S21**. Summary of measured and predicted yields for the best five performing models based on three features (M3).

| | $R^2$ | MAE (%) | LOO MAE (%) | Features | Coefficients |
|---|---|---|---|---|---|
| M3-1 | 0.79 | 5.92 | 10.34 | $q_S$<br>$q_{C2}$<br>$V_{bur(S)}$ | -2535.54<br>102.61<br>-11.79 |
| M3-2 | 0.75 | 7.08 | 17.39 | $q_{C9}$<br>$E_{\_LUMO}$<br>$V_{bur(S)}$ | -101.60<br>3593.48<br>-7.39 |
| M3-3 | 0.74 | 6.92 | 12.00 | $q_S$<br>$q_{C7}$<br>$E_{\_LUMO}$ | -1846.69<br>-307.95<br>2244.15 |
| M3-4 | 0.71 | 7.60 | 15.99 | $q_S$<br>$V_{bur(S)}$<br>$B_{max}$ | -1224.77<br>-7.14<br>7.96 |
| M3-5 | 0.71 | 7.15 | 12.41 | $q_S$<br>$q_{C7}$<br>$B_{max}$ | -1607.06<br>232.08<br>8.14 |

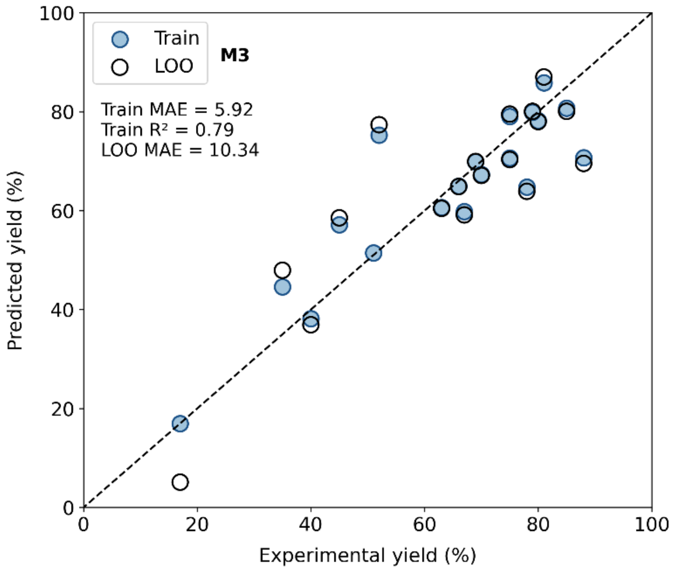


**Figure S25**. Measured vs. predicted yield plot for the best model with three features contribution (M3-1 in Table S21).

**Table S22**. Summary of measured and predicted yields for the best five performing models based on four features (M4).

| | $R^2$ | MAE (%) | LOO MAE (%) | Features | Coefficients |
|---|---|---|---|---|---|
| M4-1 | 0.86 | 5.25 | 17.43 | $bd_{\_C1-S}$<br>$q_{C9}$<br>$q_{C2}$<br>$V_{bur(S)}$ | -1224.24<br>-2905.51<br>142.89<br>-11.01 |
| M4-2 | 0.82 | 5.49 | 58.59 | $q_S$<br>$q_{C1}$<br>$q_{C2}$<br>$V_{bur(S)}$ | -2787.43<br>-259.02<br>106.85<br>-11.52 |
| M4-3 | 0.81 | 5.27 | 11.75 | $q_S$<br>$q_{C2}$<br>$E_{\_LUMO}$<br>$V_{bur(S)}$ | -2412.07<br>96.21<br>1016.18<br>-11.42 |
| M4-4 | 0.81 | 5.88 | 13.29 | $q_S$<br>$q_{C2}$<br>$V_{bur(S)}$<br>L | -2530.59<br>116.20<br>-11.80<br>3.35 |
| M4-5 | 0.80 | 6.09 | 17.01 | $q_{C8}$<br>$q_{C9}$<br>$E_{\_LUMO}$<br>$V_{bur(S)}$ | -40.43<br>-132.72<br>3694.37<br>-8.55 |

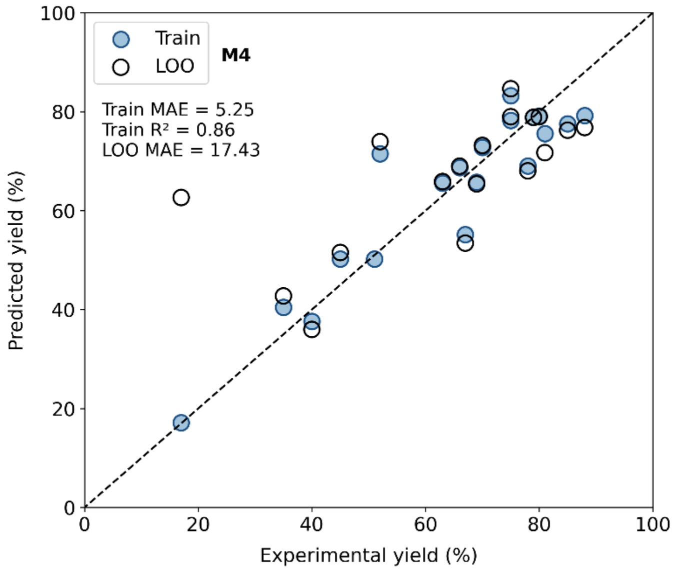


**Figure S26**. Measured vs. predicted yield plot for the best model with four features contribution (M4-1 in Table S22).

## 9. References


1 Gao, P., Zhang, Q. & Chen, F. Base-Promoted Synthesis of Vinyl Sulfides from Sulfonium Triflates. *Org. Lett.* **24**, 7769-7773 (2022).

2 Juliá, F. *et al.* High Site Selectivity in Electrophilic Aromatic Substitutions: Mechanism of C–H Thianthrenation. *J. Am. Chem. Soc.* **143**, 16041-16054 (2021).

3 Dewanji, A. *et al.* A general arene C–H functionalization strategy via electron donor–acceptor complex photoactivation. *Nat. Chem.* **15**, 43-52 (2023).

4 Xu, P. *et al.* Site-Selective Late-Stage Aromatic [18F]Fluorination via Aryl Sulfonium Salts. *Angew. Chem. Int. Ed.* **59**, 1956-1960 (2020).

5 Chen, X.-Y. *et al.* Cu-Mediated Thianthrenation and Phenoxathiination of Arylborons. *J. Am. Chem. Soc.* **145**, 10431-10440 (2023).

6 Gaussian 16, Revision A.03, Frisch, M. J. et al. (Gaussian, Inc., Wallingford CT, 2016).

7 Ernzerhof, M. & Scuseria, G. E. Assessment of the Perdew–Burke–Ernzerhof exchange-correlation functional. *J. Chem. Phy.s* **110**, 5029-5036 (1999).

8 Adamo, C. & Barone, V. Toward reliable density functional methods without adjustable parameters: The PBE0 model. *J. Chem. Phys.* **110**, 6158-6170 (1999).

9 Grimme, S., Ehrlich, S. & Goerigk, L. Effect of the damping function in dispersion corrected density functional theory. *J. Comput. Chem.* **32**, 1456-1465 (2011).

10 Grimme, S., Antony, J., Ehrlich, S. & Krieg, H. A consistent and accurate ab initio parametrization of density functional dispersion correction (DFT-D) for the 94 elements H-Pu. *J. Chem. Phys.* **132**, 154104 (2010).

11 Weigend, F. Accurate Coulomb-fitting basis sets for H to Rn. *Phys. Chem. Chem. Phys.* **8**, 1057-1065 (2006).

12 Weigend, F. & Ahlrichs, R. Balanced basis sets of split valence, triple zeta valence and quadruple zeta valence quality for H to Rn: Design and assessment of accuracy. *Phys. Chem. Chem. Phys.* **7**, 3297-3305 (2005).

13 Schäfer, A., Huber, C. & Ahlrichs, R. Fully optimized contracted Gaussian basis sets of triple zeta valence quality for atoms Li to Kr. *J. Chem. Phys.* **100**, 5829-5835 (1994).

14 Schäfer, A., Horn, H. & Ahlrichs, R. Fully optimized contracted Gaussian basis sets for atoms Li to Kr. *J. Chem. Phys.* **97**, 2571-2577 (1992).

15 Martin, J. M. L. & Sundermann, A. Correlation consistent valence basis sets for use with the Stuttgart–Dresden–Bonn relativistic effective core potentials: The atoms Ga–Kr and In–Xe. *J. Chem. Phys.* **114**, 3408-3420 (2001).

16 Dolg, M., Wedig, U., Stoll, H. & Preuss, H. Energy-adjustedabinitiopseudopotentials for the first row transition elements. *J. Chem. Phys.* **86**, 866-872 (1987).

17 Zhao, Y. & Truhlar, D. G. The M06 suite of density functionals for main group thermochemistry, thermochemical kinetics, noncovalent interactions, excited states, and transition elements: two new functionals and systematic testing of four M06-class functionals and 12 other functionals. *Theor. Chem. Acc.* **120**, 215-241 (2008).

18 Zhao, Y. & Truhlar, D. G. A new local density functional for main-group thermochemistry, transition metal bonding, thermochemical kinetics, and noncovalent interactions. *J. Chem. Phys.* **125**, 194101 (2006).

19 Chai, J.-D. & Head-Gordon, M. Long-range corrected hybrid density functionals with damped atom–atom dispersion corrections. *Phys. Chem. Chem. Phys.* **10**, 6615-6620 (2008).

20 Yu, F. Spin-Component-Scaled Double-Hybrid Density Functionals with Nonlocal van der Waals Correlations for Noncovalent Interactions. *J. Chem. Theory Comput.* **10**, 4400-4407 (2014).

21 Neese, F., Wennmohs, F., Becker, U. & Riplinger, C. The ORCA quantum chemistry program package. *J. Chem. Phys.* **152**, 224108 (2020).

22 Barone, V. & Cossi, M. Quantum Calculation of Molecular Energies and Energy Gradients in Solution by a Conductor Solvent Model. *J. Phys. Chem. A* **102**, 1995-2001 (1998).

23 Marenich, A. V., Cramer, C. J. & Truhlar, D. G. Universal solvation model based on solute electron density and on a continuum model of the solvent defined by the bulk dielectric constant and atomic surface tensions. *J. Phys. Chem. B* **113**, 6378-6396 (2009).

24 Legault, C. Y. CYLview, 1.0b ( Legault, C. Y., Université de Sherbrooke, 2009).

25 Verloop, A., Hoogenstraaten, W. & Tipker, J. in *Drug Design* Vol. 11 (ed E. J. Ariëns) 165-207 (Academic Press, 1976).

26 Falivene, L. *et al.* SambVca 2. A Web Tool for Analyzing Catalytic Pockets with Topographic Steric Maps. *Organometallics* **35**, 2286-2293 (2016).

27 Luchini, G., Patterson, T., Paton, R. S., DBSTEP: DFT Based Steric Parameters. (2022)

28 Glendening, E. D.; Reed, A. E.; Carpenter, J. E.; Weinhold, F. NBO Version 3.1 (1990).

29 Dana, S. *et al.* Parametrization of $\kappa^2$-N,O-Oxazoline Preligands for Enantioselective Cobaltaelectro-Catalyzed C–H Activations. *Acs. Catal.* **15**, 4450-4459 (2025).

30 FPedregosa, F. et al. Scikit-learn: machine learning in python. *J. Mach. Learn. Res.* **12**, 2825–2830 (2011).

31 Simonetti, M.; Perry, G. J. P.; Cambeiro, X. C.; Juliá-Hernández, F.; Arokianathar, J. N. & Larrosa, I. Ru-Catalyzed C–H Arylation of Fluoroarenes with Aryl Halides. *J. Am. Chem. Soc.***138**, 3596-3606 (2016).

## 10. Cartesian Coordinates of the Optimized Structure

**int1**

Lowest frequency = 5.8640 $cm^{-1}$

Charge = 0, Multiplicity = 1

63

| | | | |
|---|---|---|---|
| Ru | -0.014981000 | 0.011346000 | 0.001325000 |
| O | -0.704564000 | 1.706918000 | 1.076423000 |
| C | -0.971328000 | 2.302741000 | -0.005287000 |
| O | -0.700098000 | 1.703145000 | -1.083194000 |
| C | 0.960687000 | -2.271928000 | 0.007284000 |
| O | 0.682126000 | -1.681733000 | -1.074438000 |
| O | 0.678130000 | -1.678404000 | 1.085563000 |
| C | -2.936348000 | -1.059091000 | 0.000105000 |
| C | 2.921916000 | 1.037151000 | 0.001095000 |
| N | 1.815645000 | 0.696346000 | 0.001989000 |
| N | -1.840453000 | -0.686121000 | 0.000371000 |
| C | -4.332195000 | -1.512547000 | -0.001654000 |
| C | -4.477089000 | -2.645597000 | -1.027682000 |
| C | -4.686398000 | -2.012911000 | 1.406194000 |
| C | -5.221321000 | -0.320891000 | -0.387348000 |
| H | -4.210318000 | -2.302472000 | -2.037612000 |
| H | -3.830877000 | -3.497485000 | -0.770983000 |
| H | -5.521131000 | -2.993959000 | -1.042356000 |
| H | -4.570799000 | -1.212923000 | 2.151555000 |
| H | -5.732931000 | -2.353857000 | 1.420466000 |
| H | -4.042858000 | -2.854170000 | 1.701411000 |
| H | -6.275407000 | -0.637787000 | -0.391925000 |
| H | -5.109412000 | 0.505348000 | 0.329578000 |
| H | -4.966778000 | 0.055229000 | -1.388815000 |
| C | 4.333056000 | 1.440097000 | -0.001904000 |
| C | 4.963291000 | 1.007594000 | 1.330137000 |
| C | 5.031000000 | 0.739163000 | -1.176932000 |
| C | 4.406803000 | 2.965022000 | -0.162946000 |
| H | 4.460262000 | 1.487716000 | 2.181758000 |
| H | 4.899023000 | -0.082316000 | 1.461906000 |
| H | 6.025111000 | 1.297457000 | 1.343465000 |
| H | 4.578654000 | 1.028167000 | -2.136455000 |
| H | 6.094347000 | 1.023255000 | -1.192758000 |
| H | 4.964486000 | -0.354271000 | -1.080037000 |
| H | 5.461077000 | 3.281139000 | -0.168040000 |
| H | 3.943354000 | 3.286636000 | -1.106821000 |
| H | 3.895482000 | 3.476064000 | 0.665688000 |
| C | -1.664255000 | 3.654115000 | -0.002403000 |
| C | -1.169479000 | 4.475891000 | 1.187024000 |
| C | -1.400125000 | 4.377627000 | -1.320151000 |
| C | -3.165004000 | 3.360768000 | 0.149916000 |
| H | -1.316979000 | 3.927296000 | 2.127433000 |
| H | -0.095340000 | 4.698634000 | 1.093652000 |
| H | -1.713133000 | 5.432146000 | 1.242501000 |
| H | -1.717530000 | 3.764199000 | -2.174545000 |
| H | -1.946394000 | 5.333635000 | -1.345266000 |
| H | -0.327883000 | 4.591308000 | -1.446053000 |
| H | -3.737511000 | 4.301756000 | 0.167109000 |
| H | -3.530513000 | 2.749932000 | -0.689908000 |
| H | -3.361145000 | 2.816614000 | 1.086159000 |
| C | 1.689052000 | -3.604519000 | 0.004230000 |
| C | 1.440859000 | -4.337841000 | 1.319571000 |
| C | 1.223266000 | -4.437229000 | -1.189092000 |
| C | 3.181515000 | -3.267042000 | -0.141473000 |
| H | 1.736828000 | -3.716901000 | 2.176229000 |
| H | 0.374759000 | -4.582738000 | 1.440709000 |
| H | 2.014296000 | -5.277781000 | 1.345354000 |
| H | 1.358010000 | -3.881910000 | -2.127463000 |
| H | 1.794771000 | -5.377064000 | -1.245109000 |
| H | 0.155773000 | -4.691564000 | -1.100446000 |
| H | 3.782220000 | -4.190304000 | -0.155003000 |
| H | 3.365214000 | -2.717792000 | -1.077339000 |
| H | 3.523914000 | -2.644883000 | 0.700191000 |

**int1-s2**

Lowest frequency = 9.2370 $cm^{-1}$

Charge = 0, Multiplicity = 1

63

| | | | |
|---|---|---|---|
| Ru | -0.001581000 | -0.260929000 | 0.008557000 |
| O | 1.500805000 | -1.741597000 | -0.254241000 |
| C | 1.952009000 | -1.489196000 | 0.899569000 |
| O | 1.367036000 | -0.598268000 | 1.582232000 |
| C | -1.937808000 | -1.520555000 | -0.876406000 |
| O | -1.483119000 | -1.762327000 | 0.277283000 |
| O | -1.365282000 | -0.622914000 | -1.562603000 |
| C | -2.116281000 | 1.752638000 | 0.990939000 |
| C | 2.094930000 | 1.762151000 | -0.991256000 |
| C | -3.178696000 | -2.219346000 | -1.402444000 |
| C | -3.029272000 | -2.456645000 | -2.904924000 |
| C | -3.393156000 | -3.533100000 | -0.656183000 |
| C | -4.349907000 | -1.259995000 | -1.137490000 |
| H | -2.828131000 | -1.512806000 | -3.430177000 |
| H | -2.194626000 | -3.142695000 | -3.114750000 |
| H | -3.950153000 | -2.902263000 | -3.312912000 |
| H | -3.464767000 | -3.362657000 | 0.426825000 |
| H | -4.319088000 | -4.018800000 | -1.002148000 |
| H | -2.555519000 | -4.225515000 | -0.827257000 |
| H | -5.292886000 | -1.701818000 | -1.496575000 |
| H | -4.453561000 | -1.058430000 | -0.059617000 |
| H | -4.192964000 | -0.302963000 | -1.657641000 |
| C | 3.200953000 | -2.183787000 | 1.411566000 |
| C | 3.314648000 | -2.017356000 | 2.923896000 |
| C | 3.145912000 | -3.661426000 | 1.022190000 |
| C | 4.385703000 | -1.502231000 | 0.709305000 |
| H | 3.322121000 | -0.954827000 | 3.203972000 |
| H | 2.464082000 | -2.488557000 | 3.438304000 |
| H | 4.242034000 | -2.486837000 | 3.287854000 |
| H | 3.016325000 | -3.772075000 | -0.063200000 |
| H | 4.075337000 | -4.169410000 | 1.324184000 |
| H | 2.302486000 | -4.168067000 | 1.515528000 |
| H | 5.333725000 | -1.966040000 | 1.024673000 |
| H | 4.296117000 | -1.597835000 | -0.383128000 |

| | | | |
|---|---|---|---|
| H | 4.427235000 | -0.431020000 | 0.963247000 |
| C | -3.214019000 | 2.610324000 | 1.455183000 |
| C | -4.134020000 | 2.909793000 | 0.262291000 |
| C | -3.985732000 | 1.852692000 | 2.546047000 |
| C | -2.625346000 | 3.910683000 | 2.019576000 |
| H | -3.591464000 | 3.439720000 | -0.533918000 |
| H | -4.545385000 | 1.981908000 | -0.160707000 |
| H | -4.971274000 | 3.542897000 | 0.594047000 |
| H | -3.335600000 | 1.616547000 | 3.400658000 |
| H | -4.820538000 | 2.474803000 | 2.903682000 |
| H | -4.396482000 | 0.910178000 | 2.155921000 |
| H | -3.441517000 | 4.559474000 | 2.372431000 |
| H | -1.953791000 | 3.707180000 | 2.866205000 |
| H | -2.058523000 | 4.455678000 | 1.250704000 |
| C | 3.191787000 | 2.613994000 | -1.468054000 |
| C | 3.918766000 | 3.202629000 | -0.250134000 |
| C | 4.150603000 | 1.737553000 | -2.288414000 |
| C | 2.610177000 | 3.735105000 | -2.340264000 |
| H | 3.240657000 | 3.818975000 | 0.357696000 |
| H | 4.325639000 | 2.405879000 | 0.389366000 |
| H | 4.752683000 | 3.835450000 | -0.590633000 |
| H | 3.640038000 | 1.298024000 | -3.157274000 |
| H | 4.989621000 | 2.351669000 | -2.650012000 |
| H | 4.556018000 | 0.918125000 | -1.677401000 |
| H | 3.427083000 | 4.375178000 | -2.707091000 |
| H | 2.074801000 | 3.324384000 | -3.208436000 |
| H | 1.911167000 | 4.361501000 | -1.767104000 |
| N | -1.271086000 | 1.045434000 | 0.631657000 |
| N | 1.253320000 | 1.055028000 | -0.623538000 |

**int2**

Lowest frequency = 12.4776 cm$^{-1}$

Charge = 0, Multiplicity = 1

75

| | | | |
|---|---|---|---|
| O | -2.392060000 | -2.140487000 | -0.142006000 |
| C | -1.594073000 | -2.118409000 | 0.789629000 |
| O | -0.642377000 | -1.265692000 | 0.961157000 |
| C | -0.029844000 | 2.691633000 | 0.899877000 |
| O | -0.436461000 | 1.709338000 | 1.586228000 |
| O | 0.240296000 | 2.476707000 | -0.317436000 |
| C | 2.669267000 | -0.216136000 | 0.958436000 |
| N | 1.643435000 | 0.079725000 | 0.521312000 |
| C | 3.958552000 | -0.680584000 | 1.478701000 |
| C | 3.881597000 | -2.213543000 | 1.569591000 |
| C | 5.060508000 | -0.251151000 | 0.499502000 |
| C | 4.180369000 | -0.058194000 | 2.863526000 |
| H | 3.079346000 | -2.531420000 | 2.250902000 |
| H | 3.693371000 | -2.655703000 | 0.581344000 |
| H | 4.839041000 | -2.599694000 | 1.950425000 |
| H | 5.111510000 | 0.843866000 | 0.412096000 |
| H | 6.030716000 | -0.615322000 | 0.869084000 |
| H | 4.889238000 | -0.669614000 | -0.501409000 |
| H | 5.141720000 | -0.409671000 | 3.267006000 |
| H | 4.209503000 | 1.039853000 | 2.806988000 |
| H | 3.383163000 | -0.349472000 | 3.562536000 |
| C | -1.657305000 | -3.162493000 | 1.921582000 |
| C | -2.668001000 | -4.246445000 | 1.563754000 |
| C | -0.267372000 | -3.774890000 | 2.115487000 |
| C | -2.085601000 | -2.433885000 | 3.200240000 |
| H | -3.670088000 | -3.820437000 | 1.413073000 |
| H | -2.386352000 | -4.755714000 | 0.630662000 |
| H | -2.724139000 | -4.996584000 | 2.368675000 |
| H | 0.460211000 | -2.996478000 | 2.384993000 |
| H | -0.290372000 | -4.531999000 | 2.915862000 |
| H | 0.083506000 | -4.260717000 | 1.192130000 |
| H | -2.138915000 | -3.139327000 | 4.045335000 |
| H | -1.371668000 | -1.636266000 | 3.451095000 |
| H | -3.080483000 | -1.975487000 | 3.081078000 |
| C | 0.203628000 | 4.046677000 | 1.533660000 |
| C | 0.199414000 | 5.131902000 | 0.460036000 |
| C | -0.872752000 | 4.305097000 | 2.587657000 |
| C | 1.586300000 | 3.962083000 | 2.201199000 |
| H | 0.951399000 | 4.925151000 | -0.313774000 |
| H | -0.780206000 | 5.194425000 | -0.036957000 |
| H | 0.418121000 | 6.110708000 | 0.913998000 |
| H | -0.893541000 | 3.498234000 | 3.333135000 |
| H | -0.675571000 | 5.258136000 | 3.102266000 |
| H | -1.870757000 | 4.365965000 | 2.127290000 |
| H | 1.827639000 | 4.918739000 | 2.690177000 |
| H | 1.604838000 | 3.167663000 | 2.962378000 |
| H | 2.368155000 | 3.749709000 | 1.455775000 |
| C | 0.954760000 | -1.967163000 | -1.649307000 |
| C | 2.232218000 | -1.634516000 | -1.981629000 |
| C | 2.533099000 | -0.312321000 | -2.432003000 |
| C | 1.558767000 | 0.630042000 | -2.548770000 |
| C | 0.200064000 | 0.312453000 | -2.173765000 |
| C | -0.104022000 | -1.006292000 | -1.724629000 |
| F | 0.670264000 | -3.203029000 | -1.264328000 |
| F | 3.242931000 | -2.495219000 | -1.866875000 |
| F | 3.803683000 | -0.026106000 | -2.692151000 |
| F | 1.831907000 | 1.846608000 | -2.982679000 |
| Ru | -0.205254000 | 0.422036000 | -0.081572000 |
| N | -2.120131000 | 0.692715000 | -0.493458000 |
| C | -3.260571000 | 0.641833000 | -0.646920000 |
| C | -4.694534000 | 0.393189000 | -0.816498000 |
| C | -5.213933000 | -0.175877000 | 0.513030000 |
| C | -5.410937000 | 1.696537000 | -1.184526000 |
| C | -4.832011000 | -0.658594000 | -1.929001000 |
| H | -4.666951000 | -1.094627000 | 0.768059000 |
| H | -5.095151000 | 0.548612000 | 1.331981000 |
| H | -6.283705000 | -0.412567000 | 0.409345000 |
| H | -5.022692000 | 2.113689000 | -2.125130000 |
| H | -6.484629000 | 1.494243000 | -1.314188000 |
| H | -5.296336000 | 2.453195000 | -0.394459000 |
| H | -5.896560000 | -0.906456000 | -2.058243000 |
| H | -4.444582000 | -0.277119000 | -2.885098000 |
| H | -4.276975000 | -1.567073000 | -1.655808000 |
| F | -0.723678000 | 1.022514000 | -2.851886000 |
| H | -1.120415000 | -1.404675000 | -1.793851000 |

**int3**

Lowest frequency = 10.0097 cm$^{-1}$

Charge = 0, Multiplicity = 1

75

| | | | |
|---|---|---|---|
| O | 2.071464000 | -1.949929000 | -0.364021000 |
| C | 1.769336000 | -1.646408000 | -1.527347000 |
| O | 0.990803000 | -0.685790000 | -1.845903000 |
| C | -0.495792000 | 2.892511000 | -0.366266000 |
| O | 0.040962000 | 2.355245000 | -1.383220000 |
| O | -0.620321000 | 2.182950000 | 0.669632000 |
| C | -2.684168000 | -0.095521000 | -1.395749000 |
| N | -1.604375000 | 0.123801000 | -1.049928000 |
| C | -4.060071000 | -0.425761000 | -1.781247000 |
| C | -4.224313000 | -1.950274000 | -1.683725000 |
| C | -5.010672000 | 0.283413000 | -0.805368000 |
| C | -4.299129000 | 0.057469000 | -3.217976000 |
| H | -3.538462000 | -2.466626000 | -2.370197000 |
| H | -4.022609000 | -2.312488000 | -0.665971000 |
| H | -5.256938000 | -2.219138000 | -1.952898000 |
| H | -4.881303000 | 1.374824000 | -0.846507000 |
| H | -6.051038000 | 0.046250000 | -1.074314000 |
| H | -4.835897000 | -0.048107000 | 0.228395000 |
| H | -5.328853000 | -0.189318000 | -3.517468000 |
| H | -4.166144000 | 1.146235000 | -3.298239000 |
| H | -3.606916000 | -0.428534000 | -3.920344000 |
| C | 2.342662000 | -2.469455000 | -2.688990000 |
| C | 3.868206000 | -2.338003000 | -2.635185000 |
| C | 1.942160000 | -3.928398000 | -2.448518000 |
| C | 1.816160000 | -1.985090000 | -4.035456000 |
| H | 4.179989000 | -1.294968000 | -2.806047000 |
| H | 4.248111000 | -2.659474000 | -1.654500000 |
| H | 4.336327000 | -2.961006000 | -3.413911000 |
| H | 0.846776000 | -4.042869000 | -2.462917000 |
| H | 2.364787000 | -4.578199000 | -3.231418000 |
| H | 2.305044000 | -4.268103000 | -1.468350000 |
| H | 2.244142000 | -2.591143000 | -4.850291000 |
| H | 0.720533000 | -2.062510000 | -4.081655000 |
| H | 2.075627000 | -0.931104000 | -4.208156000 |
| C | -1.048624000 | 4.302302000 | -0.433910000 |
| C | -2.501436000 | 4.156329000 | -0.916407000 |
| C | -1.017481000 | 4.933849000 | 0.956238000 |
| C | -0.243333000 | 5.128378000 | -1.434792000 |
| H | -2.537486000 | 3.677399000 | -1.906895000 |
| H | -3.086699000 | 3.545757000 | -0.211714000 |
| H | -2.976004000 | 5.147170000 | -0.992901000 |
| H | 0.015933000 | 5.041244000 | 1.319096000 |
| H | -1.476316000 | 5.934310000 | 0.927430000 |
| H | -1.563118000 | 4.314433000 | 1.681197000 |
| H | -0.678506000 | 6.135290000 | -1.530211000 |
| H | 0.801969000 | 5.236899000 | -1.108099000 |
| H | -0.236449000 | 4.650238000 | -2.423882000 |
| C | -1.137872000 | -1.998292000 | 0.946076000 |
| C | -2.031732000 | -2.410269000 | 1.925748000 |
| C | -1.920222000 | -1.892131000 | 3.214827000 |
| C | -0.921458000 | -0.963239000 | 3.507050000 |
| C | -0.046722000 | -0.558170000 | 2.505610000 |
| C | -0.178279000 | -1.006438000 | 1.185461000 |
| F | -1.242997000 | -2.550705000 | -0.254564000 |
| F | -2.980021000 | -3.297005000 | 1.648577000 |
| F | -2.754891000 | -2.284329000 | 4.159562000 |
| F | -0.810775000 | -0.487682000 | 4.737111000 |
| Ru | 0.207826000 | 0.527008000 | -0.397509000 |
| N | 2.038094000 | 0.931229000 | 0.219835000 |
| C | 3.130281000 | 0.951892000 | 0.591855000 |
| C | 4.491854000 | 0.761942000 | 1.099874000 |
| C | 5.450064000 | 0.661705000 | -0.094725000 |
| C | 4.872260000 | 1.932241000 | 2.013570000 |
| C | 4.459410000 | -0.565034000 | 1.878821000 |
| H | 5.169694000 | -0.173004000 | -0.752659000 |
| H | 5.450673000 | 1.589001000 | -0.686055000 |
| H | 6.471549000 | 0.485830000 | 0.275060000 |
| H | 4.170342000 | 2.022388000 | 2.855175000 |
| H | 5.880393000 | 1.761748000 | 2.420302000 |
| H | 4.879368000 | 2.883979000 | 1.462265000 |
| H | 5.471766000 | -0.798169000 | 2.242586000 |
| H | 3.781905000 | -0.491479000 | 2.741819000 |
| H | 4.102482000 | -1.381895000 | 1.234445000 |
| F | 0.898238000 | 0.309414000 | 2.831067000 |
| H | 0.769160000 | -1.072793000 | 0.484410000 |

**TS4**

Lowest frequency = -666.7661 $cm^{-1}$

Charge = 0, Multiplicity = 1

75

| | | | |
|---|---|---|---|
| O | 1.540735000 | -2.193909000 | -0.344041000 |
| C | 1.361508000 | -1.885076000 | -1.545751000 |
| O | 0.792277000 | -0.814900000 | -1.906564000 |
| C | -0.147081000 | 2.968001000 | -0.388418000 |
| O | 0.263661000 | 2.390936000 | -1.437860000 |
| O | -0.315958000 | 2.259539000 | 0.644882000 |
| C | -2.760331000 | 0.215432000 | -1.298202000 |
| N | -1.650403000 | 0.352924000 | -1.011149000 |
| C | -4.166025000 | -0.072439000 | -1.600621000 |
| C | -4.819563000 | 1.181296000 | -2.195136000 |
| C | -4.201550000 | -1.238521000 | -2.599709000 |
| C | -4.849624000 | -0.474591000 | -0.283992000 |
| H | -4.773908000 | 2.025143000 | -1.491184000 |
| H | -4.324044000 | 1.482798000 | -3.129423000 |
| H | -5.876920000 | 0.971181000 | -2.415806000 |
| H | -3.703517000 | -2.124628000 | -2.181256000 |
| H | -5.248448000 | -1.495077000 | -2.821886000 |
| H | -3.700635000 | -0.970527000 | -3.541131000 |
| H | -5.904535000 | -0.717451000 | -0.482378000 |
| H | -4.367926000 | -1.357387000 | 0.159830000 |
| H | -4.814071000 | 0.345432000 | 0.447852000 |
| C | 1.791550000 | -2.840521000 | -2.657114000 |
| C | 2.645595000 | -3.968759000 | -2.087928000 |
| C | 0.500114000 | -3.404743000 | -3.265596000 |
| C | 2.567978000 | -2.054877000 | -3.715825000 |
| H | 3.569855000 | -3.578778000 | -1.635629000 |
| H | 2.103737000 | -4.520195000 | -1.307317000 |
| H | 2.924865000 | -4.671572000 | -2.888409000 |
| H | -0.116391000 | -2.595367000 | -3.682302000 |

| | | | |
|---|---|---|---|
| H | 0.738980000 | -4.117470000 | -4.070740000 |
| H | -0.095091000 | -3.932480000 | -2.504179000 |
| H | 2.844874000 | -2.715193000 | -4.552696000 |
| H | 1.964641000 | -1.222744000 | -4.103332000 |
| H | 3.495625000 | -1.634583000 | -3.295548000 |
| C | -0.510328000 | 4.440756000 | -0.396093000 |
| C | -0.302596000 | 5.029363000 | 0.997917000 |
| C | 0.330379000 | 5.176697000 | -1.437032000 |
| C | -1.998165000 | 4.501670000 | -0.778920000 |
| H | -0.877395000 | 4.470630000 | 1.749130000 |
| H | 0.757649000 | 4.989316000 | 1.290602000 |
| H | -0.623327000 | 6.082610000 | 1.015986000 |
| H | 0.208751000 | 4.724485000 | -2.430825000 |
| H | 0.028268000 | 6.234346000 | -1.488332000 |
| H | 1.400097000 | 5.138307000 | -1.180981000 |
| H | -2.339440000 | 5.548486000 | -0.806686000 |
| H | -2.164849000 | 4.056495000 | -1.771680000 |
| H | -2.611918000 | 3.956548000 | -0.045659000 |
| C | -1.327641000 | -1.791911000 | 0.996637000 |
| C | -2.122868000 | -2.188017000 | 2.063412000 |
| C | -1.823585000 | -1.729763000 | 3.344277000 |
| C | -0.724261000 | -0.894138000 | 3.534529000 |
| C | 0.045528000 | -0.509522000 | 2.441861000 |
| C | -0.259650000 | -0.891522000 | 1.127345000 |
| F | -1.623199000 | -2.307474000 | -0.196907000 |
| F | -3.163507000 | -2.992299000 | 1.872523000 |
| F | -2.565819000 | -2.098376000 | 4.373963000 |
| F | -0.425544000 | -0.475632000 | 4.755863000 |
| Ru | 0.224767000 | 0.522233000 | -0.443680000 |
| N | 2.114005000 | 0.688244000 | 0.080485000 |
| C | 3.225792000 | 0.637685000 | 0.388118000 |
| C | 4.602838000 | 0.404608000 | 0.833166000 |
| C | 5.507546000 | 1.542625000 | 0.347197000 |
| C | 4.590068000 | 0.333226000 | 2.367705000 |
| C | 5.041041000 | -0.941637000 | 0.233046000 |
| H | 5.501553000 | 1.615233000 | -0.750030000 |
| H | 5.187562000 | 2.509905000 | 0.761127000 |
| H | 6.540260000 | 1.351381000 | 0.675567000 |
| H | 3.914511000 | -0.458496000 | 2.720657000 |
| H | 5.606814000 | 0.117700000 | 2.729660000 |
| H | 4.257350000 | 1.284985000 | 2.806140000 |
| H | 6.048044000 | -1.193294000 | 0.598919000 |
| H | 4.344881000 | -1.744058000 | 0.516702000 |
| H | 5.072365000 | -0.891145000 | -0.865333000 |
| F | 1.085918000 | 0.276395000 | 2.692090000 |
| H | 0.700382000 | -1.259724000 | 0.328757000 |

**int5**

Lowest frequency = 10.8298 cm$^{-1}$

Charge = 0, Multiplicity = 1

75

| | | | |
|---|---|---|---|
| O | -0.306947000 | -2.706035000 | -0.972992000 |
| C | -0.591968000 | -2.006905000 | -2.041592000 |
| O | -0.438153000 | -0.789891000 | -2.088422000 |
| C | 1.832943000 | 2.414648000 | -0.419060000 |
| O | 1.592707000 | 1.946806000 | -1.559264000 |
| O | 1.316952000 | 1.850810000 | 0.597515000 |
| C | -2.170156000 | 2.224164000 | -0.581758000 |
| N | -1.214486000 | 1.577490000 | -0.644389000 |
| C | -3.432893000 | 2.950626000 | -0.409351000 |
| C | -4.578541000 | 1.940528000 | -0.580724000 |
| C | -3.444249000 | 3.536735000 | 1.010690000 |
| C | -3.528324000 | 4.066378000 | -1.457225000 |
| H | -4.581103000 | 1.515444000 | -1.594722000 |
| H | -4.489614000 | 1.110120000 | 0.133586000 |
| H | -5.539154000 | 2.451204000 | -0.413880000 |
| H | -2.610954000 | 4.239013000 | 1.157780000 |
| H | -4.389143000 | 4.076834000 | 1.174513000 |
| H | -3.362207000 | 2.740912000 | 1.764931000 |
| H | -4.477977000 | 4.607423000 | -1.328606000 |
| H | -2.703257000 | 4.785383000 | -1.348669000 |
| H | -3.498577000 | 3.657008000 | -2.477343000 |
| C | -1.148760000 | -2.800497000 | -3.202686000 |
| C | -0.158114000 | -3.916726000 | -3.555962000 |
| C | -2.481600000 | -3.406025000 | -2.736144000 |
| C | -1.368141000 | -1.875609000 | -4.395133000 |
| H | 0.808524000 | -3.502288000 | -3.881591000 |
| H | 0.019540000 | -4.577705000 | -2.696017000 |
| H | -0.562748000 | -4.522050000 | -4.381210000 |
| H | -3.180681000 | -2.620607000 | -2.412053000 |
| H | -2.944297000 | -3.962805000 | -3.565110000 |
| H | -2.330097000 | -4.095155000 | -1.893474000 |
| H | -1.775315000 | -2.450965000 | -5.239966000 |
| H | -2.073482000 | -1.070332000 | -4.147024000 |
| H | -0.427357000 | -1.404497000 | -4.713659000 |
| C | 2.728113000 | 3.626286000 | -0.208575000 |
| C | 1.910771000 | 4.689899000 | 0.533019000 |
| C | 3.908868000 | 3.180519000 | 0.662350000 |
| C | 3.217989000 | 4.158832000 | -1.550546000 |
| H | 1.047513000 | 5.013867000 | -0.069641000 |
| H | 1.533621000 | 4.293211000 | 1.486215000 |
| H | 2.533066000 | 5.575211000 | 0.739024000 |
| H | 4.505007000 | 2.408009000 | 0.151069000 |
| H | 4.570944000 | 4.034714000 | 0.875507000 |
| H | 3.551608000 | 2.763233000 | 1.614617000 |
| H | 3.869968000 | 5.033090000 | -1.396547000 |
| H | 3.783645000 | 3.391413000 | -2.097954000 |
| H | 2.374608000 | 4.459294000 | -2.188679000 |
| C | -2.003114000 | -0.879333000 | 0.935410000 |
| C | -2.744500000 | -1.301240000 | 2.034610000 |
| C | -2.106383000 | -1.481963000 | 3.258208000 |
| C | -0.738823000 | -1.234992000 | 3.345598000 |
| C | -0.042096000 | -0.791041000 | 2.223114000 |
| C | -0.635472000 | -0.576104000 | 0.971381000 |
| F | -2.687619000 | -0.779943000 | -0.218918000 |
| F | -4.050028000 | -1.530975000 | 1.925316000 |
| F | -2.786490000 | -1.892515000 | 4.319686000 |
| F | -0.113296000 | -1.417230000 | 4.503037000 |
| Ru | 0.352298000 | 0.397311000 | -0.538658000 |
| N | 1.949124000 | -0.720761000 | -0.325770000 |
| C | 2.911368000 | -1.303865000 | -0.055361000 |
| C | 4.082794000 | -2.020801000 | 0.458855000 |
| C | 4.763662000 | -2.789649000 | -0.679497000 |

| | | | |
|---|---|---|---|
| C | 5.036393000 | -0.977897000 | 1.061632000 |
| C | 3.581746000 | -2.982440000 | 1.548908000 |
| H | 4.078369000 | -3.522908000 | -1.129382000 |
| H | 5.109684000 | -2.107314000 | -1.469523000 |
| H | 5.636181000 | -3.331021000 | -0.283695000 |
| H | 4.537098000 | -0.403688000 | 1.855093000 |
| H | 5.910405000 | -1.489263000 | 1.492902000 |
| H | 5.389171000 | -0.272929000 | 0.294857000 |
| H | 4.440330000 | -3.501465000 | 2.001455000 |
| H | 3.041443000 | -2.434574000 | 2.333714000 |
| H | 2.903436000 | -3.737908000 | 1.125680000 |
| F | 1.263315000 | -0.563246000 | 2.408964000 |
| H | -0.100232000 | -2.046239000 | -0.258582000 |

**int2-s2**

Lowest frequency = 14.4959 $cm^{-1}$

Charge = 0, Multiplicity = 1

75

| | | | |
|---|---|---|---|
| O | -1.765210000 | 0.593808000 | -1.063288000 |
| C | -1.947563000 | 1.611512000 | -0.328967000 |
| O | -1.158816000 | 1.771528000 | 0.646480000 |
| C | 1.876326000 | 0.114969000 | 2.368398000 |
| O | 1.689814000 | -0.137780000 | 1.123624000 |
| C | 1.259666000 | -2.251659000 | -1.602052000 |
| C | 1.640817000 | 2.213611000 | -1.423521000 |
| N | 0.996039000 | 1.367736000 | -0.975239000 |
| N | 0.775142000 | -1.371119000 | -1.033984000 |
| C | 1.818789000 | -3.448526000 | -2.238765000 |
| C | 1.901375000 | -4.534696000 | -1.154120000 |
| C | 0.873244000 | -3.884233000 | -3.367161000 |
| C | 3.213066000 | -3.119600000 | -2.786815000 |
| H | 2.568235000 | -4.225797000 | -0.336502000 |
| H | 0.909110000 | -4.736616000 | -0.728172000 |
| H | 2.293846000 | -5.461834000 | -1.598666000 |
| H | 0.796062000 | -3.109368000 | -4.143772000 |
| H | 1.260390000 | -4.803633000 | -3.831796000 |
| H | -0.133952000 | -4.085893000 | -2.976214000 |
| H | 3.644044000 | -4.020804000 | -3.248298000 |
| H | 3.164934000 | -2.330398000 | -3.551445000 |
| H | 3.885781000 | -2.786221000 | -1.983293000 |
| C | 2.468666000 | 3.317347000 | -1.921124000 |
| C | 1.762615000 | 3.964333000 | -3.120371000 |
| C | 2.624595000 | 4.326216000 | -0.772355000 |
| C | 3.835476000 | 2.750455000 | -2.330099000 |
| H | 1.630604000 | 3.245053000 | -3.941661000 |
| H | 0.773606000 | 4.351918000 | -2.835828000 |
| H | 2.370476000 | 4.803899000 | -3.489659000 |
| H | 3.108014000 | 3.861005000 | 0.098463000 |
| H | 3.246764000 | 5.167791000 | -1.112443000 |
| H | 1.647958000 | 4.719228000 | -0.455202000 |
| H | 4.474491000 | 3.571199000 | -2.688902000 |
| H | 4.334417000 | 2.269150000 | -1.476622000 |
| H | 3.735184000 | 2.011404000 | -3.138260000 |
| C | -3.027378000 | 2.628204000 | -0.639483000 |
| C | -4.060598000 | 2.018390000 | -1.583749000 |
| C | -3.680124000 | 3.084667000 | 0.666387000 |
| C | -2.314994000 | 3.811646000 | -1.313216000 |
| H | -3.592758000 | 1.675813000 | -2.517128000 |
| H | -4.554587000 | 1.152821000 | -1.119200000 |
| H | -4.832229000 | 2.764521000 | -1.828421000 |
| H | -2.927110000 | 3.474760000 | 1.364862000 |
| H | -4.419313000 | 3.874192000 | 0.460566000 |
| H | -4.195943000 | 2.250072000 | 1.161908000 |
| H | -3.043840000 | 4.599178000 | -1.560495000 |
| H | -1.554459000 | 4.239627000 | -0.642796000 |
| H | -1.822663000 | 3.495302000 | -2.246448000 |
| C | 3.373076000 | 0.159722000 | 2.745231000 |
| C | 3.522323000 | 0.400332000 | 4.242758000 |
| C | 4.017413000 | -1.171834000 | 2.349714000 |
| C | 4.023546000 | 1.300037000 | 1.954561000 |
| H | 3.040550000 | 1.342730000 | 4.539113000 |
| H | 3.040049000 | -0.402515000 | 4.818474000 |
| H | 4.587572000 | 0.442581000 | 4.522226000 |
| H | 3.898259000 | -1.352262000 | 1.271755000 |
| H | 5.092460000 | -1.169227000 | 2.594077000 |
| H | 3.549553000 | -2.010854000 | 2.888593000 |
| H | 5.100590000 | 1.365362000 | 2.180267000 |
| H | 3.897414000 | 1.134296000 | 0.874247000 |
| H | 3.564292000 | 2.268217000 | 2.212394000 |
| O | 1.009746000 | 0.307832000 | 3.216125000 |
| H | -0.538480000 | -0.530642000 | 2.604151000 |
| Ru | -0.035333000 | 0.056362000 | 0.021730000 |
| C | -1.226889000 | -0.875419000 | 1.809003000 |
| C | -2.566671000 | -0.423023000 | 1.803440000 |
| C | -0.892097000 | -1.952876000 | 0.975860000 |
| C | -3.493902000 | -0.943529000 | 0.933604000 |
| C | -1.853490000 | -2.501185000 | 0.085259000 |
| C | -3.122273000 | -1.977434000 | 0.045582000 |
| F | 0.176266000 | -2.709230000 | 1.231011000 |
| F | -1.511164000 | -3.509501000 | -0.708855000 |
| F | -4.015159000 | -2.430030000 | -0.814725000 |
| F | -4.735547000 | -0.481109000 | 0.884435000 |
| F | -2.936321000 | 0.518982000 | 2.652251000 |

**TS3-s2**

Lowest frequency = -850.5057 $cm^{-1}$

Charge = 0, Multiplicity = 1

75

| | | | |
|---|---|---|---|
| O | -1.336308000 | 1.122655000 | -1.124537000 |
| C | -1.902386000 | 1.537676000 | -0.070478000 |
| O | -1.294053000 | 1.375215000 | 1.025684000 |
| C | 1.879050000 | -1.358322000 | 1.708307000 |
| O | 1.649463000 | -0.143540000 | 1.481796000 |
| C | 2.185648000 | -1.121453000 | -1.912965000 |
| C | 1.538702000 | 3.211844000 | -0.211581000 |
| N | 1.142324000 | 2.127105000 | -0.148946000 |
| N | 1.505665000 | -0.476789000 | -1.234399000 |
| C | 2.941639000 | -2.093029000 | -2.711014000 |
| C | 4.435343000 | -1.958916000 | -2.388497000 |
| C | 2.421999000 | -3.482250000 | -2.303234000 |

| | | | |
|---|---|---|---|
| C | 2.679092000 | -1.832528000 | -4.199937000 |
| H | 4.811382000 | -0.957970000 | -2.646382000 |
| H | 4.625639000 | -2.138255000 | -1.320298000 |
| H | 5.002763000 | -2.701324000 | -2.969929000 |
| H | 1.350042000 | -3.575981000 | -2.526576000 |
| H | 2.971314000 | -4.256471000 | -2.860485000 |
| H | 2.558165000 | -3.650807000 | -1.225275000 |
| H | 3.212618000 | -2.583729000 | -4.801947000 |
| H | 1.605668000 | -1.902771000 | -4.427156000 |
| H | 3.033050000 | -0.834985000 | -4.498887000 |
| C | 2.000564000 | 4.603760000 | -0.274215000 |
| C | 3.399843000 | 4.633415000 | -0.904229000 |
| C | 1.004949000 | 5.398446000 | -1.132622000 |
| C | 2.041090000 | 5.160712000 | 1.156458000 |
| H | 4.116981000 | 4.053398000 | -0.305503000 |
| H | 3.385263000 | 4.222099000 | -1.923959000 |
| H | 3.753877000 | 5.674154000 | -0.955890000 |
| H | -0.006012000 | 5.363044000 | -0.701553000 |
| H | 1.325382000 | 6.450158000 | -1.183340000 |
| H | 0.956362000 | 4.999689000 | -2.156189000 |
| H | 2.376304000 | 6.208727000 | 1.130013000 |
| H | 1.046269000 | 5.124677000 | 1.623214000 |
| H | 2.737332000 | 4.587926000 | 1.785734000 |
| C | -3.250668000 | 2.232135000 | -0.127445000 |
| C | -4.103824000 | 1.599173000 | -1.227326000 |
| C | -3.940817000 | 2.124979000 | 1.230954000 |
| C | -2.965618000 | 3.703278000 | -0.466534000 |
| H | -3.575151000 | 1.611880000 | -2.190304000 |
| H | -4.348311000 | 0.552484000 | -0.991823000 |
| H | -5.051162000 | 2.149929000 | -1.334292000 |
| H | -3.323860000 | 2.572026000 | 2.022446000 |
| H | -4.912164000 | 2.642612000 | 1.202636000 |
| H | -4.118862000 | 1.075491000 | 1.508297000 |
| H | -3.908813000 | 4.268983000 | -0.523693000 |
| H | -2.334550000 | 4.167268000 | 0.307630000 |
| H | -2.450574000 | 3.788940000 | -1.435363000 |
| C | 2.922387000 | -1.721106000 | 2.761080000 |
| C | 3.986195000 | -2.592981000 | 2.084665000 |
| C | 3.556129000 | -0.466030000 | 3.350936000 |
| C | 2.206257000 | -2.526784000 | 3.851515000 |
| H | 3.530201000 | -3.488943000 | 1.639896000 |
| H | 4.506472000 | -2.035901000 | 1.288801000 |
| H | 4.740122000 | -2.911863000 | 2.821212000 |
| H | 2.798134000 | 0.175452000 | 3.821693000 |
| H | 4.302697000 | -0.743822000 | 4.111365000 |
| H | 4.055225000 | 0.130198000 | 2.573415000 |
| H | 2.922651000 | -2.830745000 | 4.630709000 |
| H | 1.414210000 | -1.928363000 | 4.327482000 |
| H | 1.742782000 | -3.427782000 | 3.425914000 |
| O | 1.285574000 | -2.306106000 | 1.120801000 |
| H | 0.235092000 | -1.785129000 | 0.533145000 |
| Ru | 0.298500000 | 0.383211000 | 0.009466000 |
| C | -0.994294000 | -1.439866000 | 0.180465000 |
| C | -1.859711000 | -1.398476000 | 1.278602000 |
| C | -1.543998000 | -1.876422000 | -1.026873000 |
| C | -3.221238000 | -1.652509000 | 1.168085000 |
| C | -2.897746000 | -2.150924000 | -1.177328000 |
| C | -3.737637000 | -2.021247000 | -0.072813000 |

| | | | |
|---|---|---|---|
| F | -0.783326000 | -1.998247000 | -2.111788000 |
| F | -3.406657000 | -2.502524000 | -2.349748000 |
| F | -5.034489000 | -2.233798000 | -0.206958000 |
| F | -4.038063000 | -1.523554000 | 2.205207000 |
| F | -1.390754000 | -1.068434000 | 2.471991000 |

**int4-s2**

Lowest frequency = 11.3544 $cm^{-1}$

Charge = 0, Multiplicity = 1

75

| | | | |
|---|---|---|---|
| O | 1.387060000 | 1.303365000 | -1.525091000 |
| C | 1.597936000 | 2.104779000 | -0.581719000 |
| O | 1.232116000 | 1.753694000 | 0.591301000 |
| C | -0.513680000 | -0.417181000 | 2.521098000 |
| O | -0.141111000 | -1.062467000 | 1.542015000 |
| C | -0.439425000 | -2.522392000 | -1.835150000 |
| C | 3.532678000 | -1.049101000 | 0.195300000 |
| N | 2.413312000 | -0.784407000 | 0.067076000 |
| N | -0.022514000 | -1.633324000 | -1.222637000 |
| C | -1.108830000 | -3.597903000 | -2.577306000 |
| C | -2.188354000 | -4.193839000 | -1.659913000 |
| C | -1.754141000 | -2.982554000 | -3.828089000 |
| C | -0.082137000 | -4.666760000 | -2.971664000 |
| H | -1.738013000 | -4.645078000 | -0.763806000 |
| H | -2.894986000 | -3.419990000 | -1.329882000 |
| H | -2.740164000 | -4.975101000 | -2.204928000 |
| H | -0.997088000 | -2.529482000 | -4.484470000 |
| H | -2.279942000 | -3.767720000 | -4.392739000 |
| H | -2.481517000 | -2.205424000 | -3.552708000 |
| H | -0.587516000 | -5.475644000 | -3.520947000 |
| H | 0.700626000 | -4.246352000 | -3.620009000 |
| H | 0.398635000 | -5.101557000 | -2.083150000 |
| C | 4.961815000 | -1.349172000 | 0.350543000 |
| C | 5.662760000 | -1.054891000 | -0.983873000 |
| C | 5.523182000 | -0.447015000 | 1.459523000 |
| C | 5.118716000 | -2.828677000 | 0.727494000 |
| H | 5.257963000 | -1.680552000 | -1.792290000 |
| H | 5.540946000 | -0.000250000 | -1.270129000 |
| H | 6.738337000 | -1.266463000 | -0.885312000 |
| H | 5.019820000 | -0.635831000 | 2.418688000 |
| H | 6.597665000 | -0.649395000 | 1.585298000 |
| H | 5.397085000 | 0.615481000 | 1.206077000 |
| H | 6.187328000 | -3.063012000 | 0.847164000 |
| H | 4.606777000 | -3.054188000 | 1.674170000 |
| H | 4.706444000 | -3.483491000 | -0.053810000 |
| C | 2.332663000 | 3.412118000 | -0.793955000 |
| C | 2.150539000 | 3.871738000 | -2.238705000 |
| C | 1.803466000 | 4.460027000 | 0.184824000 |
| C | 3.814884000 | 3.122427000 | -0.508290000 |
| H | 2.510071000 | 3.108609000 | -2.942703000 |
| H | 1.088449000 | 4.053591000 | -2.456725000 |
| H | 2.708890000 | 4.804984000 | -2.410891000 |
| H | 1.923475000 | 4.123729000 | 1.224110000 |
| H | 2.351771000 | 5.406096000 | 0.055925000 |
| H | 0.734607000 | 4.649498000 | 0.011458000 |

| | | | |
|---|---|---|---|
| H | 4.412225000 | 4.037725000 | -0.643181000 |
| H | 3.952187000 | 2.769397000 | 0.525436000 |
| H | 4.202624000 | 2.354125000 | -1.195476000 |
| C | -1.269554000 | -1.075239000 | 3.657003000 |
| C | -0.673954000 | -0.638549000 | 4.998303000 |
| C | -2.726044000 | -0.590884000 | 3.541501000 |
| C | -1.210134000 | -2.591229000 | 3.497872000 |
| H | 0.373820000 | -0.963188000 | 5.094559000 |
| H | -0.707231000 | 0.453676000 | 5.112166000 |
| H | -1.245948000 | -1.091947000 | 5.821947000 |
| H | -3.165837000 | -0.889231000 | 2.578796000 |
| H | -3.325360000 | -1.040824000 | 4.347701000 |
| H | -2.789040000 | 0.503018000 | 3.630025000 |
| H | -1.791464000 | -3.070271000 | 4.299876000 |
| H | -1.624912000 | -2.896649000 | 2.527826000 |
| H | -0.174861000 | -2.959078000 | 3.552614000 |
| O | -0.341425000 | 0.871176000 | 2.647197000 |
| H | 0.128990000 | 1.250514000 | 1.861067000 |
| Ru | 0.524660000 | -0.095451000 | -0.189674000 |
| C | -1.409555000 | 0.659547000 | -0.393952000 |
| C | -1.756557000 | 1.976772000 | -0.716355000 |
| C | -2.528973000 | -0.146417000 | -0.145893000 |
| C | -3.062831000 | 2.463639000 | -0.772046000 |
| C | -3.851396000 | 0.290362000 | -0.182908000 |
| C | -4.128947000 | 1.614174000 | -0.499904000 |
| F | -2.398781000 | -1.447250000 | 0.180763000 |
| F | -4.844871000 | -0.548924000 | 0.101198000 |
| F | -5.378560000 | 2.056529000 | -0.541699000 |
| F | -3.298332000 | 3.733730000 | -1.081720000 |
| F | -0.820938000 | 2.898736000 | -0.996649000 |

**int2-s3**

Lowest frequency = 9.8785 cm$^{-1}$

Charge = 0, Multiplicity = 1

60

| | | | |
|---|---|---|---|
| Ru | -0.187487000 | -0.238139000 | -0.186155000 |
| O | 1.230979000 | 0.834067000 | 0.942678000 |
| C | 1.603443000 | 1.475174000 | -0.081940000 |
| O | 1.047295000 | 1.158229000 | -1.175484000 |
| C | -2.252890000 | -1.598569000 | -0.192669000 |
| O | -1.701891000 | -1.228567000 | -1.270880000 |
| O | -1.671491000 | -1.271391000 | 0.881443000 |
| C | -2.182051000 | 2.160714000 | 0.166304000 |
| N | -1.429553000 | 1.294757000 | 0.042408000 |
| C | -3.147547000 | 3.253696000 | 0.319220000 |
| C | -4.191684000 | 3.129580000 | -0.800277000 |
| C | -3.810482000 | 3.112754000 | 1.697402000 |
| C | -2.388348000 | 4.584105000 | 0.212865000 |
| H | -3.723888000 | 3.211492000 | -1.791816000 |
| H | -4.720429000 | 2.166983000 | -0.745020000 |
| H | -4.930212000 | 3.938542000 | -0.696223000 |
| H | -3.068385000 | 3.178835000 | 2.505786000 |
| H | -4.542203000 | 3.923804000 | 1.829040000 |
| H | -4.337311000 | 2.151891000 | 1.788663000 |
| H | -3.100173000 | 5.415330000 | 0.326159000 |
| H | -1.624828000 | 4.671192000 | 0.999250000 |
| H | -1.892660000 | 4.682768000 | -0.763645000 |
| C | 2.617248000 | 2.594788000 | -0.001123000 |
| C | 3.539873000 | 2.360195000 | 1.194179000 |
| C | 3.408841000 | 2.653022000 | -1.307663000 |
| C | 1.811724000 | 3.889433000 | 0.191925000 |
| H | 2.966353000 | 2.293440000 | 2.128945000 |
| H | 4.104876000 | 1.423870000 | 1.080821000 |
| H | 4.261186000 | 3.187252000 | 1.279346000 |
| H | 2.739947000 | 2.799621000 | -2.166863000 |
| H | 4.129779000 | 3.484118000 | -1.273153000 |
| H | 3.965998000 | 1.719766000 | -1.472428000 |
| H | 2.494713000 | 4.750932000 | 0.253171000 |
| H | 1.124555000 | 4.053200000 | -0.652260000 |
| H | 1.222695000 | 3.850697000 | 1.121324000 |
| C | -3.568468000 | -2.344978000 | -0.193587000 |
| C | -4.665690000 | -1.292683000 | -0.416520000 |
| C | -3.561507000 | -3.351869000 | -1.344803000 |
| C | -3.764170000 | -3.046007000 | 1.148074000 |
| H | -4.668810000 | -0.551494000 | 0.398552000 |
| H | -4.514176000 | -0.764393000 | -1.369866000 |
| H | -5.654283000 | -1.777047000 | -0.440360000 |
| H | -2.763778000 | -4.097203000 | -1.206973000 |
| H | -4.525150000 | -3.882355000 | -1.388911000 |
| H | -3.390681000 | -2.847610000 | -2.306016000 |
| H | -4.729504000 | -3.575093000 | 1.159547000 |
| H | -2.963407000 | -3.777850000 | 1.328749000 |
| H | -3.746218000 | -2.325739000 | 1.977741000 |
| C | 1.802916000 | -2.108996000 | 1.189231000 |
| C | 3.026841000 | -1.548538000 | 0.961959000 |
| C | 3.390739000 | -1.100077000 | -0.339967000 |
| C | 2.498126000 | -1.173054000 | -1.371155000 |
| C | 1.176428000 | -1.667433000 | -1.159679000 |
| C | 0.846758000 | -2.175187000 | 0.123883000 |
| H | 0.616870000 | -2.024288000 | -2.028165000 |
| F | 1.469940000 | -2.571014000 | 2.379840000 |
| F | 3.906192000 | -1.392621000 | 1.937037000 |
| F | 4.605089000 | -0.588442000 | -0.505378000 |
| F | 2.854608000 | -0.784753000 | -2.584181000 |
| F | -0.041865000 | -3.178835000 | 0.219633000 |

**TS3-s3**

Lowest frequency = -569.3645 cm$^{-1}$

Charge = 0, Multiplicity = 1

60

| | | | |
|---|---|---|---|
| Ru | -0.452879000 | 0.166525000 | 0.322869000 |
| O | -0.534714000 | -1.032309000 | -1.318950000 |
| C | -0.079049000 | -2.065990000 | -0.724746000 |
| O | 0.186346000 | -1.933429000 | 0.498347000 |
| C | -0.476314000 | 2.854350000 | -0.797039000 |
| O | 0.683850000 | 2.617293000 | -1.199534000 |
| O | -1.167488000 | 2.031800000 | -0.118596000 |
| C | -3.323229000 | -0.804185000 | 0.963192000 |
| N | -2.260676000 | -0.393674000 | 0.757252000 |
| C | -4.665487000 | -1.346947000 | 1.203301000 |

| | | | |
|---|---|---|---|
| C | -4.856947000 | -1.534400000 | 2.715161000 |
| C | -5.698814000 | -0.357428000 | 0.645731000 |
| C | -4.763282000 | -2.696935000 | 0.476129000 |
| H | -4.111550000 | -2.229725000 | 3.127312000 |
| H | -4.771414000 | -0.576792000 | 3.248733000 |
| H | -5.859011000 | -1.947567000 | 2.905323000 |
| H | -5.559967000 | -0.204964000 | -0.434202000 |
| H | -6.710653000 | -0.756400000 | 0.813354000 |
| H | -5.623969000 | 0.619140000 | 1.145360000 |
| H | -5.762583000 | -3.126968000 | 0.641902000 |
| H | -4.612873000 | -2.575700000 | -0.606409000 |
| H | -4.012492000 | -3.406626000 | 0.852756000 |
| C | 0.113523000 | -3.375239000 | -1.456274000 |
| C | 0.010763000 | -3.158710000 | -2.963327000 |
| C | 1.487368000 | -3.934915000 | -1.073964000 |
| C | -0.993525000 | -4.320598000 | -0.966909000 |
| H | -0.964306000 | -2.732679000 | -3.238718000 |
| H | 0.788204000 | -2.465301000 | -3.313628000 |
| H | 0.135426000 | -4.118255000 | -3.488184000 |
| H | 1.565893000 | -4.065947000 | 0.014287000 |
| H | 1.643366000 | -4.910335000 | -1.559681000 |
| H | 2.293294000 | -3.259152000 | -1.396122000 |
| H | -0.884411000 | -5.306246000 | -1.445295000 |
| H | -0.939158000 | -4.454156000 | 0.123591000 |
| H | -1.990114000 | -3.926636000 | -1.221076000 |
| C | -1.100828000 | 4.205757000 | -1.132401000 |
| C | -0.200368000 | 5.289218000 | -0.527181000 |
| C | -1.114999000 | 4.337763000 | -2.659917000 |
| C | -2.515767000 | 4.310388000 | -0.573369000 |
| H | -0.163032000 | 5.207793000 | 0.570429000 |
| H | 0.824764000 | 5.201430000 | -0.913372000 |
| H | -0.589072000 | 6.287694000 | -0.781028000 |
| H | -1.748122000 | 3.562929000 | -3.119467000 |
| H | -1.516263000 | 5.321726000 | -2.948603000 |
| H | -0.099512000 | 4.236863000 | -3.067594000 |
| H | -2.948547000 | 5.289431000 | -0.831715000 |
| H | -3.164074000 | 3.523183000 | -0.983910000 |
| H | -2.520555000 | 4.205911000 | 0.520961000 |
| C | 3.824493000 | -0.710120000 | -0.206879000 |
| C | 4.258554000 | -0.313146000 | 1.056777000 |
| C | 3.474550000 | 0.539887000 | 1.836057000 |
| C | 2.260629000 | 0.973925000 | 1.323337000 |
| C | 1.770204000 | 0.579540000 | 0.076371000 |
| C | 2.601247000 | -0.248065000 | -0.680468000 |
| H | 1.103771000 | 1.463346000 | -0.585704000 |
| F | 4.572324000 | -1.526701000 | -0.933932000 |
| F | 5.416482000 | -0.743002000 | 1.518872000 |
| F | 3.891779000 | 0.902631000 | 3.038316000 |
| F | 1.500250000 | 1.760555000 | 2.088400000 |
| F | 2.233396000 | -0.642273000 | -1.892539000 |

**int4-s3**

Lowest frequency = 10.7366 $cm^{-1}$

Charge = 0, Multiplicity = 1

60

| | | | |
|---|---|---|---|
| Ru | -0.509437000 | 0.165266000 | 0.642845000 |
| O | -1.011699000 | -0.966182000 | -0.979383000 |
| C | -1.325607000 | -2.006993000 | -0.290972000 |
| O | -1.248100000 | -1.895940000 | 0.952463000 |
| C | 0.749072000 | 1.963669000 | -1.281595000 |
| O | 0.699376000 | 0.965698000 | -2.119034000 |
| O | 0.199736000 | 1.943335000 | -0.176488000 |
| C | -3.578231000 | 1.046280000 | 0.725770000 |
| N | -2.445688000 | 0.804235000 | 0.712695000 |
| C | -5.021633000 | 1.315347000 | 0.739381000 |
| C | -5.492006000 | 1.379610000 | 2.199698000 |
| C | -5.281602000 | 2.651411000 | 0.029431000 |
| C | -5.722250000 | 0.165779000 | -0.000938000 |
| H | -5.296200000 | 0.432166000 | 2.722053000 |
| H | -4.984975000 | 2.188026000 | 2.745960000 |
| H | -6.575286000 | 1.572002000 | 2.224144000 |
| H | -4.935472000 | 2.621236000 | -1.013823000 |
| H | -6.362329000 | 2.858968000 | 0.029591000 |
| H | -4.770632000 | 3.479863000 | 0.540962000 |
| H | -6.808867000 | 0.339982000 | 0.002036000 |
| H | -5.384170000 | 0.101838000 | -1.045410000 |
| H | -5.523353000 | -0.799776000 | 0.486063000 |
| C | -1.810092000 | -3.266420000 | -0.972353000 |
| C | -1.441216000 | -3.239837000 | -2.453514000 |
| C | -1.172142000 | -4.468230000 | -0.271292000 |
| C | -3.336048000 | -3.300080000 | -0.796387000 |
| H | -1.889287000 | -2.373516000 | -2.960719000 |
| H | -0.351345000 | -3.181412000 | -2.584325000 |
| H | -1.802456000 | -4.156234000 | -2.944768000 |
| H | -1.412407000 | -4.466708000 | 0.800880000 |
| H | -1.543994000 | -5.403416000 | -0.717393000 |
| H | -0.077857000 | -4.441730000 | -0.374821000 |
| H | -3.746500000 | -4.212891000 | -1.255340000 |
| H | -3.605739000 | -3.295322000 | 0.270172000 |
| H | -3.809789000 | -2.431518000 | -1.280196000 |
| C | 1.602647000 | 3.140117000 | -1.698523000 |
| C | 3.002715000 | 2.825987000 | -1.135032000 |
| C | 1.659395000 | 3.269996000 | -3.219666000 |
| C | 1.058942000 | 4.416080000 | -1.057944000 |
| H | 2.975147000 | 2.733979000 | -0.039512000 |
| H | 3.398866000 | 1.887296000 | -1.550339000 |
| H | 3.693995000 | 3.640266000 | -1.400187000 |
| H | 0.661145000 | 3.456920000 | -3.643825000 |
| H | 2.307993000 | 4.116138000 | -3.491136000 |
| H | 2.062464000 | 2.361213000 | -3.686432000 |
| H | 1.721304000 | 5.260594000 | -1.299054000 |
| H | 0.051719000 | 4.653346000 | -1.432971000 |
| H | 1.001998000 | 4.314620000 | 0.033995000 |
| C | 3.465182000 | -1.738470000 | 0.089937000 |
| C | 4.259699000 | -0.914859000 | 0.883535000 |
| C | 3.666648000 | 0.135619000 | 1.580804000 |
| C | 2.294308000 | 0.320325000 | 1.464206000 |
| C | 1.451358000 | -0.458461000 | 0.670767000 |
| C | 2.094414000 | -1.496231000 | -0.001517000 |
| H | 0.199586000 | 0.206187000 | -1.729819000 |
| F | 4.024416000 | -2.741250000 | -0.576711000 |
| F | 5.565099000 | -1.128130000 | 0.974085000 |
| F | 4.411399000 | 0.941634000 | 2.328393000 |

| | | | |
|---|---|---|---|
| F | 1.754056000 | 1.344909000 | 2.156250000 |
| F | 1.410103000 | -2.326341000 | -0.806804000 |

**int2-s4**

Lowest frequency = 6.7883cm$^{-1}$

Charge = 0, Multiplicity = 1

60

| | | | |
|---|---|---|---|
| Ru | 0.606087000 | 0.087589000 | 0.139925000 |
| O | 2.617793000 | -0.044991000 | 0.718697000 |
| C | 2.889462000 | -0.627034000 | -0.370960000 |
| O | 1.908601000 | -0.879830000 | -1.141529000 |
| C | 0.405762000 | 2.541781000 | 0.298163000 |
| O | 1.039864000 | 1.987851000 | -0.648321000 |
| O | -0.102169000 | 1.781735000 | 1.175282000 |
| C | -2.122271000 | 0.182493000 | -1.337065000 |
| N | -1.111311000 | 0.126484000 | -0.779765000 |
| C | -3.418815000 | 0.185119000 | -2.022665000 |
| C | -3.508796000 | -1.104080000 | -2.854115000 |
| C | -4.520785000 | 0.217643000 | -0.952518000 |
| C | -3.496097000 | 1.426010000 | -2.922112000 |
| H | -2.722309000 | -1.136752000 | -3.621782000 |
| H | -3.409128000 | -1.991094000 | -2.213255000 |
| H | -4.487234000 | -1.142332000 | -3.356081000 |
| H | -4.452428000 | 1.128173000 | -0.339737000 |
| H | -5.503463000 | 0.204735000 | -1.447404000 |
| H | -4.455723000 | -0.652675000 | -0.285734000 |
| H | -4.466693000 | 1.436412000 | -3.440206000 |
| H | -3.408635000 | 2.350516000 | -2.332761000 |
| H | -2.699110000 | 1.419620000 | -3.679652000 |
| C | 4.295984000 | -1.007000000 | -0.769185000 |
| C | 5.271898000 | -0.629993000 | 0.340763000 |
| C | 4.314968000 | -2.519326000 | -1.023388000 |
| C | 4.624549000 | -0.252756000 | -2.064163000 |
| H | 5.248253000 | 0.450529000 | 0.541699000 |
| H | 5.025411000 | -1.147877000 | 1.278718000 |
| H | 6.295674000 | -0.908656000 | 0.047809000 |
| H | 3.589699000 | -2.793327000 | -1.802292000 |
| H | 5.319316000 | -2.829633000 | -1.350660000 |
| H | 4.060322000 | -3.077708000 | -0.110453000 |
| H | 5.636488000 | -0.520182000 | -2.405709000 |
| H | 3.907180000 | -0.507629000 | -2.856997000 |
| H | 4.590775000 | 0.836389000 | -1.907930000 |
| C | 0.185027000 | 4.037892000 | 0.337102000 |
| C | 0.101971000 | 4.505075000 | 1.789667000 |
| C | 1.312830000 | 4.746591000 | -0.409586000 |
| C | -1.157896000 | 4.278205000 | -0.373502000 |
| H | -0.685508000 | 3.964947000 | 2.333051000 |
| H | 1.052898000 | 4.332964000 | 2.315926000 |
| H | -0.119144000 | 5.582849000 | 1.825245000 |
| H | 1.391119000 | 4.384047000 | -1.443789000 |
| H | 1.126562000 | 5.831314000 | -0.428664000 |
| H | 2.282375000 | 4.573403000 | 0.080890000 |
| H | -1.390900000 | 5.354281000 | -0.381859000 |
| H | -1.119026000 | 3.925900000 | -1.415777000 |
| H | -1.973626000 | 3.751300000 | 0.144551000 |
| C | -0.812300000 | -2.651422000 | 0.233581000 |
| C | -2.076409000 | -2.399574000 | 0.666083000 |
| C | -2.325343000 | -1.479182000 | 1.732265000 |
| C | -1.291766000 | -0.821706000 | 2.325776000 |
| C | 0.057750000 | -1.011189000 | 1.874931000 |
| C | 0.300620000 | -1.943547000 | 0.818723000 |
| H | 0.855409000 | -0.827946000 | 2.602080000 |
| F | -0.582931000 | -3.503751000 | -0.749622000 |
| F | -3.132341000 | -2.953313000 | 0.073449000 |
| F | -3.588372000 | -1.279827000 | 2.089631000 |
| F | -1.512114000 | -0.009077000 | 3.345523000 |
| F | 1.452205000 | -2.646587000 | 0.820199000 |

**int3-s4**

Lowest frequency = 5.4078 cm$^{-1}$

Charge = 0, Multiplicity = 1

60

| | | | |
|---|---|---|---|
| Ru | -0.041529000 | 0.435430000 | -0.737428000 |
| O | -1.706157000 | -2.048158000 | 0.166711000 |
| C | -2.106101000 | -1.610905000 | -0.918911000 |
| O | -1.558607000 | -0.633229000 | -1.546312000 |
| C | 0.685990000 | 2.806065000 | -1.002207000 |
| O | -0.364108000 | 2.314401000 | -1.539076000 |
| O | 1.404467000 | 2.019398000 | -0.328585000 |
| C | -1.666273000 | 0.867692000 | 1.779201000 |
| N | -1.012561000 | 0.797494000 | 0.827977000 |
| C | -2.506069000 | 0.731615000 | 2.973343000 |
| C | -2.325119000 | -0.719778000 | 3.452236000 |
| C | -2.045941000 | 1.727509000 | 4.044199000 |
| C | -3.965581000 | 0.989671000 | 2.573407000 |
| H | -2.567423000 | -1.429772000 | 2.647970000 |
| H | -1.285645000 | -0.901518000 | 3.759704000 |
| H | -2.986235000 | -0.901939000 | 4.313129000 |
| H | -2.159161000 | 2.765916000 | 3.699610000 |
| H | -2.657778000 | 1.595718000 | 4.949195000 |
| H | -0.992409000 | 1.561425000 | 4.311663000 |
| H | -4.610187000 | 0.862168000 | 3.456145000 |
| H | -4.100831000 | 2.011822000 | 2.190790000 |
| H | -4.292509000 | 0.280552000 | 1.799402000 |
| C | -3.349483000 | -2.220752000 | -1.574931000 |
| C | -3.202937000 | -3.743523000 | -1.552891000 |
| C | -4.542968000 | -1.802736000 | -0.705090000 |
| C | -3.538051000 | -1.717187000 | -3.002174000 |
| H | -2.356155000 | -4.067658000 | -2.177918000 |
| H | -3.023923000 | -4.097737000 | -0.528381000 |
| H | -4.115292000 | -4.221746000 | -1.942627000 |
| H | -4.652112000 | -0.706517000 | -0.686790000 |
| H | -5.475849000 | -2.228967000 | -1.106709000 |
| H | -4.414614000 | -2.159301000 | 0.327763000 |
| H | -4.442094000 | -2.165979000 | -3.443656000 |
| H | -3.639447000 | -0.623575000 | -3.028630000 |
| H | -2.677886000 | -1.983414000 | -3.633677000 |
| C | 0.988925000 | 4.282629000 | -1.129241000 |
| C | 2.454623000 | 4.542853000 | -0.792803000 |
| C | 0.654491000 | 4.749859000 | -2.546764000 |

| | | | |
|---|---|---|---|
| C | 0.073768000 | 4.987930000 | -0.114386000 |
| H | 2.700405000 | 4.176772000 | 0.213515000 |
| H | 3.122507000 | 4.035406000 | -1.504754000 |
| H | 2.662955000 | 5.622786000 | -0.836497000 |
| H | -0.391005000 | 4.524579000 | -2.798340000 |
| H | 0.811887000 | 5.836164000 | -2.630336000 |
| H | 1.297332000 | 4.254874000 | -3.290648000 |
| H | 0.241606000 | 6.075438000 | -0.152956000 |
| H | -0.984665000 | 4.790046000 | -0.338536000 |
| H | 0.283371000 | 4.643854000 | 0.910066000 |
| C | 3.079613000 | -1.451203000 | 1.657411000 |
| C | 3.800340000 | -2.078231000 | 0.639349000 |
| C | 3.196975000 | -2.363217000 | -0.586004000 |
| C | 1.858607000 | -2.036418000 | -0.767583000 |
| C | 1.120642000 | -1.372552000 | 0.217227000 |
| C | 1.744867000 | -1.132073000 | 1.445049000 |
| H | -0.032384000 | -1.465026000 | 0.197680000 |
| F | 3.668273000 | -1.190624000 | 2.811290000 |
| F | 5.063973000 | -2.393974000 | 0.834310000 |
| F | 3.893532000 | -2.952693000 | -1.541161000 |
| F | 1.295275000 | -2.312603000 | -1.935351000 |
| F | 1.077402000 | -0.557722000 | 2.433389000 |

**TS4-s4**

Lowest frequency = -75.2999 $cm^{-1}$

Charge = 0, Multiplicity = 1

60

| | | | |
|---|---|---|---|
| Ru | -0.191499000 | 0.410209000 | -0.793481000 |
| O | -0.854645000 | -2.566128000 | -0.195597000 |
| C | -1.598445000 | -2.123097000 | -1.088288000 |
| O | -1.490089000 | -0.950405000 | -1.582656000 |
| C | -0.066103000 | 2.924230000 | -0.956439000 |
| O | -1.000281000 | 2.233928000 | -1.482478000 |
| O | 0.852900000 | 2.296900000 | -0.359214000 |
| C | -1.726182000 | 0.452651000 | 1.818805000 |
| N | -1.138047000 | 0.489714000 | 0.823434000 |
| C | -2.387594000 | 0.264533000 | 3.113781000 |
| C | -2.507367000 | -1.252711000 | 3.333474000 |
| C | -1.504569000 | 0.899064000 | 4.198477000 |
| C | -3.772066000 | 0.923460000 | 3.080324000 |
| H | -3.127738000 | -1.718415000 | 2.554284000 |
| H | -1.517956000 | -1.730549000 | 3.312204000 |
| H | -2.972702000 | -1.441458000 | 4.312759000 |
| H | -1.400341000 | 1.982645000 | 4.042009000 |
| H | -1.965733000 | 0.733926000 | 5.183894000 |
| H | -0.501615000 | 0.449689000 | 4.199959000 |
| H | -4.270336000 | 0.770219000 | 4.049339000 |
| H | -3.694623000 | 2.005397000 | 2.899003000 |
| H | -4.402674000 | 0.483717000 | 2.294075000 |
| C | -2.739109000 | -2.996002000 | -1.611736000 |
| C | -2.169798000 | -4.371998000 | -1.965161000 |
| C | -3.738571000 | -3.130443000 | -0.454082000 |
| C | -3.414547000 | -2.361992000 | -2.823104000 |
| H | -1.452086000 | -4.301433000 | -2.797257000 |
| H | -1.649400000 | -4.808977000 | -1.101779000 |
| H | -2.980291000 | -5.050708000 | -2.273463000 |
| H | -4.142197000 | -2.146397000 | -0.166166000 |
| H | -4.584099000 | -3.768794000 | -0.754658000 |
| H | -3.254465000 | -3.580391000 | 0.425040000 |
| H | -4.238436000 | -3.004119000 | -3.172244000 |
| H | -3.820268000 | -1.370332000 | -2.579991000 |
| H | -2.703405000 | -2.231905000 | -3.651677000 |
| C | -0.116648000 | 4.437076000 | -0.992250000 |
| C | 1.280013000 | 5.010051000 | -0.766130000 |
| C | -0.694554000 | 4.898760000 | -2.330244000 |
| C | -1.053926000 | 4.849388000 | 0.155316000 |
| H | 1.706423000 | 4.649090000 | 0.179837000 |
| H | 1.965746000 | 4.716515000 | -1.574980000 |
| H | 1.234583000 | 6.109547000 | -0.737277000 |
| H | -1.682487000 | 4.451684000 | -2.506423000 |
| H | -0.795282000 | 5.995008000 | -2.337707000 |
| H | -0.039023000 | 4.611392000 | -3.166407000 |
| H | -1.141554000 | 5.946421000 | 0.191484000 |
| H | -2.058368000 | 4.424329000 | 0.011632000 |
| H | -0.663559000 | 4.503232000 | 1.124699000 |
| C | 3.166276000 | -1.095184000 | 1.679967000 |
| C | 4.107629000 | -1.264720000 | 0.664825000 |
| C | 3.703227000 | -1.318256000 | -0.670570000 |
| C | 2.350855000 | -1.215486000 | -0.961123000 |
| C | 1.375963000 | -1.008325000 | 0.021880000 |
| C | 1.821094000 | -0.986840000 | 1.347791000 |
| H | 0.281824000 | -1.543460000 | -0.115931000 |
| F | 3.562000000 | -1.054759000 | 2.941472000 |
| F | 5.386244000 | -1.371032000 | 0.967086000 |
| F | 4.604710000 | -1.466829000 | -1.625653000 |
| F | 1.974282000 | -1.240687000 | -2.238931000 |
| F | 0.951186000 | -0.859181000 | 2.343952000 |

**int5-s4**

Lowest frequency = 10.3804 $cm^{-1}$

Charge = 0, Multiplicity = 1

60

| | | | |
|---|---|---|---|
| Ru | 0.485847000 | -0.168896000 | -0.797244000 |
| O | -2.005466000 | 1.774343000 | -0.106133000 |
| C | -1.389387000 | 2.142017000 | -1.195381000 |
| O | -0.484044000 | 1.470482000 | -1.691830000 |
| C | 2.796591000 | -1.141147000 | -0.982417000 |
| O | 2.562938000 | -0.103278000 | -1.651377000 |
| O | 1.845438000 | -1.650618000 | -0.299420000 |
| C | 1.323159000 | 1.447465000 | 1.619084000 |
| N | 1.006044000 | 0.846872000 | 0.680423000 |
| C | 1.566116000 | 2.128566000 | 2.895348000 |
| C | 0.269071000 | 2.860525000 | 3.275792000 |
| C | 1.898227000 | 1.050890000 | 3.939315000 |
| C | 2.728324000 | 3.116922000 | 2.743101000 |
| H | 0.011686000 | 3.625793000 | 2.528509000 |
| H | -0.568566000 | 2.152755000 | 3.350846000 |
| H | 0.401018000 | 3.356652000 | 4.249307000 |
| H | 2.815077000 | 0.507545000 | 3.668080000 |
| H | 2.052907000 | 1.528035000 | 4.919039000 |

| | | | |
|---|---|---|---|
| H | 1.077419000 | 0.325052000 | 4.024410000 |
| H | 2.905696000 | 3.623360000 | 3.703844000 |
| H | 3.653326000 | 2.599694000 | 2.449358000 |
| H | 2.504406000 | 3.882186000 | 1.985575000 |
| C | -1.838812000 | 3.459068000 | -1.786965000 |
| C | -3.353635000 | 3.403416000 | -2.020335000 |
| C | -1.505623000 | 4.549607000 | -0.756022000 |
| C | -1.096812000 | 3.714814000 | -3.094636000 |
| H | -3.616089000 | 2.613857000 | -2.740823000 |
| H | -3.895601000 | 3.210641000 | -1.084097000 |
| H | -3.697682000 | 4.364536000 | -2.430887000 |
| H | -0.423701000 | 4.586966000 | -0.554593000 |
| H | -1.811988000 | 5.531103000 | -1.147910000 |
| H | -2.032202000 | 4.374481000 | 0.193078000 |
| H | -1.412977000 | 4.680749000 | -3.515424000 |
| H | -0.009237000 | 3.738943000 | -2.939255000 |
| H | -1.308463000 | 2.928561000 | -3.833141000 |
| C | 4.179125000 | -1.762238000 | -0.904272000 |
| C | 4.054354000 | -3.285488000 | -0.969033000 |
| C | 5.055231000 | -1.233545000 | -2.035571000 |
| C | 4.753957000 | -1.343270000 | 0.458400000 |
| H | 3.389821000 | -3.656783000 | -0.176812000 |
| H | 3.641884000 | -3.608588000 | -1.937216000 |
| H | 5.044760000 | -3.751549000 | -0.848770000 |
| H | 5.118731000 | -0.137058000 | -2.006050000 |
| H | 6.071113000 | -1.650008000 | -1.952372000 |
| H | 4.647056000 | -1.514691000 | -3.017698000 |
| H | 5.762621000 | -1.765673000 | 0.589166000 |
| H | 4.829485000 | -0.246957000 | 0.532046000 |
| H | 4.114481000 | -1.703716000 | 1.277644000 |
| C | -2.601251000 | -1.916417000 | 1.829568000 |
| C | -3.265991000 | -2.701603000 | 0.892158000 |
| C | -2.880764000 | -2.648255000 | -0.450066000 |
| C | -1.842143000 | -1.800534000 | -0.800262000 |
| C | -1.135334000 | -0.993290000 | 0.095288000 |
| C | -1.555711000 | -1.085705000 | 1.422504000 |
| H | -1.648399000 | 0.875639000 | 0.129009000 |
| F | -2.969945000 | -1.963016000 | 3.102494000 |
| F | -4.258551000 | -3.493844000 | 1.267797000 |
| F | -3.504098000 | -3.398781000 | -1.346057000 |
| F | -1.440430000 | -1.764233000 | -2.089125000 |
| F | -0.975780000 | -0.343061000 | 2.380158000 |

**int6**

Lowest frequency = 7.4287 $cm^{-1}$

Charge = 1, Multiplicity = 1

126

| | | | |
|---|---|---|---|
| O | 2.425243000 | 1.730339000 | -1.308232000 |
| C | 1.501246000 | 2.504142000 | -1.694402000 |
| O | 0.310167000 | 2.192658000 | -1.395536000 |
| C | 1.556164000 | 1.923603000 | 2.160066000 |
| C | 0.882343000 | -0.613338000 | -3.472250000 |
| N | 0.940482000 | -0.368139000 | -2.345801000 |
| N | 1.357162000 | 1.302767000 | 1.209431000 |
| C | 1.854896000 | 2.590614000 | 3.430773000 |
| C | 0.758727000 | 2.195533000 | 4.431690000 |
| C | 3.226309000 | 2.075398000 | 3.898100000 |
| C | 1.881880000 | 4.108990000 | 3.215049000 |
| H | -0.232659000 | 2.531189000 | 4.092429000 |
| H | 0.731276000 | 1.105698000 | 4.574739000 |
| H | 0.969378000 | 2.668620000 | 5.402076000 |
| H | 4.017783000 | 2.359469000 | 3.189866000 |
| H | 3.461780000 | 2.517833000 | 4.877307000 |
| H | 3.224205000 | 0.980747000 | 3.994415000 |
| H | 2.134767000 | 4.603760000 | 4.164225000 |
| H | 2.637174000 | 4.392776000 | 2.467895000 |
| H | 0.903423000 | 4.485169000 | 2.882759000 |
| C | 0.896426000 | -0.929007000 | -4.903302000 |
| C | 0.130417000 | -2.242512000 | -5.117389000 |
| C | 0.224077000 | 0.225455000 | -5.660202000 |
| C | 2.367941000 | -1.078525000 | -5.322414000 |
| H | 0.600018000 | -3.071708000 | -4.568631000 |
| H | -0.918248000 | -2.152566000 | -4.796697000 |
| H | 0.139183000 | -2.493072000 | -6.188237000 |
| H | 0.756379000 | 1.173393000 | -5.494722000 |
| H | 0.240112000 | 0.006621000 | -6.737949000 |
| H | -0.822893000 | 0.354365000 | -5.349297000 |
| H | 2.410927000 | -1.332443000 | -6.391837000 |
| H | 2.922263000 | -0.142760000 | -5.162729000 |
| H | 2.868119000 | -1.871263000 | -4.749261000 |
| C | 1.840664000 | 3.758157000 | -2.474401000 |
| C | 2.943879000 | 3.424408000 | -3.482520000 |
| C | 0.599496000 | 4.284832000 | -3.189959000 |
| C | 2.359013000 | 4.787426000 | -1.457530000 |
| H | 3.830555000 | 3.017577000 | -2.978098000 |
| H | 2.599258000 | 2.678172000 | -4.215715000 |
| H | 3.235032000 | 4.330768000 | -4.034060000 |
| H | -0.207401000 | 4.516666000 | -2.481749000 |
| H | 0.847470000 | 5.202736000 | -3.743336000 |
| H | 0.209315000 | 3.547424000 | -3.907507000 |
| H | 2.637098000 | 5.717428000 | -1.976128000 |
| H | 1.589432000 | 5.032558000 | -0.710071000 |
| H | 3.247273000 | 4.405749000 | -0.933187000 |
| Ru | 1.122077000 | 0.320580000 | -0.486028000 |
| C | 2.633523000 | -0.953444000 | 0.080479000 |
| C | 3.532654000 | -1.476414000 | -0.854505000 |
| C | 2.930961000 | -1.296628000 | 1.403587000 |
| C | 4.603944000 | -2.307085000 | -0.523520000 |
| C | 3.985160000 | -2.127237000 | 1.775320000 |
| C | 4.835686000 | -2.644503000 | 0.805335000 |
| F | 2.197867000 | -0.842274000 | 2.430202000 |
| F | 4.174888000 | -2.426342000 | 3.055159000 |
| F | 5.840540000 | -3.432990000 | 1.138014000 |
| F | 5.402500000 | -2.772572000 | -1.470848000 |
| F | 3.419290000 | -1.203891000 | -2.158678000 |
| S | -1.995978000 | 0.250032000 | -1.026613000 |
| C | -2.840285000 | 1.604928000 | -0.234394000 |
| C | -3.364983000 | -0.843258000 | -0.706105000 |
| C | -0.784097000 | -0.319208000 | 0.237245000 |
| C | -2.298612000 | 2.866069000 | -0.033283000 |
| C | -4.108096000 | 1.215642000 | 0.222548000 |
| C | -3.381370000 | -2.188744000 | -1.018778000 |
| C | -4.413191000 | -0.185978000 | -0.047342000 |

| | | | |
|---|---|---|---|
| C | -0.096563000 | -1.527632000 | -0.083987000 |
| C | -1.184243000 | -0.109007000 | 1.608386000 |
| C | -3.047121000 | 3.807815000 | 0.684352000 |
| H | -1.300634000 | 3.078255000 | -0.416330000 |
| C | -4.865519000 | 2.173968000 | 0.897736000 |
| C | -4.497428000 | -2.959566000 | -0.656613000 |
| H | -2.529429000 | -2.648754000 | -1.521684000 |
| C | -5.534947000 | -0.943255000 | 0.281155000 |
| C | 0.147331000 | -2.492121000 | 0.950397000 |
| H | -0.125607000 | -1.930132000 | -1.097430000 |
| C | -0.894575000 | -1.044445000 | 2.551633000 |
| H | -1.716982000 | 0.804070000 | 1.879927000 |
| C | -4.331700000 | 3.437715000 | 1.122792000 |
| C | -2.495058000 | 5.191097000 | 1.024776000 |
| H | -5.864187000 | 1.928289000 | 1.264513000 |
| C | -5.563132000 | -2.305024000 | -0.020751000 |
| C | -4.485920000 | -4.461515000 | -0.937827000 |
| H | -6.386330000 | -0.481413000 | 0.785461000 |
| C | -0.233663000 | -2.284411000 | 2.246741000 |
| H | 0.619817000 | -3.427335000 | 0.650835000 |
| H | -1.199771000 | -0.846534000 | 3.581487000 |
| H | -4.937194000 | 4.162286000 | 1.671181000 |
| C | -1.103727000 | 5.404563000 | 0.430673000 |
| C | -2.399785000 | 5.314961000 | 2.553517000 |
| C | -3.435090000 | 6.272089000 | 0.472926000 |
| H | -6.449177000 | -2.873949000 | 0.260368000 |
| C | -4.247023000 | -4.701169000 | -2.435689000 |
| C | -3.348112000 | -5.095961000 | -0.121207000 |
| C | -5.803435000 | -5.128600000 | -0.542740000 |
| C | -0.045826000 | -3.316289000 | 3.353626000 |
| H | -1.119075000 | 5.358332000 | -0.668096000 |
| H | -0.725183000 | 6.397616000 | 0.713980000 |
| H | -0.381993000 | 4.655894000 | 0.791404000 |
| H | -1.993096000 | 6.299478000 | 2.831565000 |
| H | -3.381831000 | 5.212008000 | 3.038163000 |
| H | -1.736942000 | 4.540695000 | 2.971343000 |
| H | -3.527196000 | 6.193961000 | -0.620886000 |
| H | -4.444752000 | 6.202221000 | 0.903396000 |
| H | -3.043395000 | 7.272629000 | 0.711674000 |
| H | -3.281169000 | -4.295664000 | -2.773294000 |
| H | -4.240721000 | -5.780819000 | -2.648998000 |
| H | -5.040220000 | -4.238818000 | -3.042510000 |
| H | -3.307664000 | -6.180733000 | -0.303799000 |
| H | -2.365865000 | -4.674412000 | -0.384480000 |
| H | -3.501359000 | -4.936231000 | 0.957056000 |
| H | -6.007937000 | -5.030811000 | 0.534143000 |
| H | -6.657070000 | -4.712573000 | -1.099264000 |
| H | -5.754785000 | -6.203641000 | -0.769215000 |
| C | -1.436670000 | -3.786929000 | 3.808041000 |
| C | 0.755453000 | -4.525250000 | 2.871762000 |
| C | 0.701470000 | -2.689392000 | 4.540657000 |
| H | -2.045036000 | -2.956719000 | 4.198121000 |
| H | -1.988133000 | -4.245752000 | 2.972802000 |
| H | -1.342399000 | -4.535602000 | 4.609645000 |
| H | 1.760336000 | -4.231863000 | 2.531459000 |
| H | 0.882126000 | -5.239884000 | 3.698076000 |
| H | 0.248602000 | -5.058122000 | 2.052857000 |
| H | 0.793807000 | -3.427078000 | 5.352200000 |
| H | 1.711208000 | -2.372963000 | 4.246866000 |
| H | 0.172978000 | -1.816375000 | 4.952572000 |

**int6-s2**

Lowest frequency = 14.4136 $cm^{-1}$

Charge = 1, Multiplicity = 1

126

| | | | |
|---|---|---|---|
| O | -2.674558000 | -1.874420000 | -0.723958000 |
| C | -2.141604000 | -2.711234000 | -1.528487000 |
| O | -0.888161000 | -2.727389000 | -1.617089000 |
| C | -1.440617000 | -3.086119000 | 2.239141000 |
| C | -1.210901000 | 0.692449000 | -2.651418000 |
| N | -1.039789000 | 0.114844000 | -1.668754000 |
| N | -1.105906000 | -2.415577000 | 1.362798000 |
| C | -1.902811000 | -3.834771000 | 3.411577000 |
| C | -0.681801000 | -4.439932000 | 4.117749000 |
| C | -2.615477000 | -2.823006000 | 4.325893000 |
| C | -2.870054000 | -4.930878000 | 2.944122000 |
| H | -0.146463000 | -5.142264000 | 3.462416000 |
| H | 0.017361000 | -3.656126000 | 4.443409000 |
| H | -1.018931000 | -4.990561000 | 5.008128000 |
| H | -3.493184000 | -2.388241000 | 3.826330000 |
| H | -2.954333000 | -3.338664000 | 5.236513000 |
| H | -1.941717000 | -2.004608000 | 4.618086000 |
| H | -3.247858000 | -5.474864000 | 3.822235000 |
| H | -3.728334000 | -4.502717000 | 2.406668000 |
| H | -2.368163000 | -5.651668000 | 2.282388000 |
| C | -1.435828000 | 1.429719000 | -3.896625000 |
| C | -0.374078000 | 2.536076000 | -3.987866000 |
| C | -1.297758000 | 0.445511000 | -5.067358000 |
| C | -2.853645000 | 2.017491000 | -3.843964000 |
| H | -0.410034000 | 3.203060000 | -3.114811000 |
| H | 0.636985000 | 2.108622000 | -4.057695000 |
| H | -0.554261000 | 3.136395000 | -4.891730000 |
| H | -2.048010000 | -0.355557000 | -5.003320000 |
| H | -1.450343000 | 0.986537000 | -6.012821000 |
| H | -0.299241000 | -0.014564000 | -5.086894000 |
| H | -3.044595000 | 2.582878000 | -4.767970000 |
| H | -3.608514000 | 1.223024000 | -3.758817000 |
| H | -2.974748000 | 2.695728000 | -2.988315000 |
| C | -3.037011000 | -3.638409000 | -2.324877000 |
| C | -3.992592000 | -2.768822000 | -3.151388000 |
| C | -2.196614000 | -4.531720000 | -3.231421000 |
| C | -3.835924000 | -4.479848000 | -1.320563000 |
| H | -4.576904000 | -2.100995000 | -2.502860000 |
| H | -3.437300000 | -2.150886000 | -3.875029000 |
| H | -4.687907000 | -3.406183000 | -3.718282000 |
| H | -1.491222000 | -5.141347000 | -2.648662000 |
| H | -2.850116000 | -5.208734000 | -3.801740000 |
| H | -1.610006000 | -3.935948000 | -3.945759000 |
| H | -4.520084000 | -5.155852000 | -1.855444000 |
| H | -3.166681000 | -5.097147000 | -0.700664000 |
| H | -4.429132000 | -3.835702000 | -0.656066000 |
| Ru | -0.858693000 | -1.086378000 | -0.106977000 |
| C | -1.637872000 | 0.232710000 | 1.244681000 |

| | | | |
|---|---|---|---|
| C | -2.797864000 | 0.951401000 | 0.926371000 |
| C | -1.288536000 | 0.292197000 | 2.596650000 |
| C | -3.539596000 | 1.674387000 | 1.858467000 |
| C | -1.995195000 | 1.005410000 | 3.562504000 |
| C | -3.136160000 | 1.709030000 | 3.191234000 |
| F | -0.215935000 | -0.380166000 | 3.057689000 |
| F | -1.603851000 | 0.989484000 | 4.827900000 |
| F | -3.824964000 | 2.396794000 | 4.082618000 |
| F | -4.623835000 | 2.337260000 | 1.486517000 |
| F | -3.262004000 | 0.981619000 | -0.326323000 |
| S | 1.291901000 | 1.267106000 | 1.084122000 |
| C | 0.926363000 | 2.775316000 | 0.206681000 |
| C | 3.045020000 | 1.539751000 | 0.902259000 |
| C | 1.115511000 | -0.120178000 | -0.069213000 |
| C | -0.341951000 | 3.321112000 | 0.052769000 |
| C | 2.103972000 | 3.433786000 | -0.182532000 |
| C | 4.016798000 | 0.653640000 | 1.322769000 |
| C | 3.318429000 | 2.713003000 | 0.187672000 |
| C | 1.219773000 | -1.400586000 | 0.548392000 |
| C | 1.729785000 | 0.074925000 | -1.361370000 |
| C | -0.469254000 | 4.581202000 | -0.547312000 |
| H | -1.210377000 | 2.775153000 | 0.416518000 |
| C | 1.969510000 | 4.671743000 | -0.813110000 |
| C | 5.361636000 | 0.930367000 | 1.029152000 |
| H | 3.737987000 | -0.258055000 | 1.854096000 |
| C | 4.654698000 | 3.003831000 | -0.076423000 |
| C | 1.844652000 | -2.469511000 | -0.174632000 |
| H | 1.143586000 | -1.482667000 | 1.633359000 |
| C | 2.258326000 | -0.986402000 | -2.023464000 |
| H | 1.748055000 | 1.075122000 | -1.800131000 |
| C | 0.704717000 | 5.219605000 | -0.989215000 |
| C | -1.813165000 | 5.300430000 | -0.667600000 |
| H | 2.854467000 | 5.221723000 | -1.139704000 |
| C | 5.649096000 | 2.118964000 | 0.340676000 |
| C | 6.433971000 | -0.078678000 | 1.437016000 |
| H | 4.927871000 | 3.911200000 | -0.619232000 |
| C | 2.312290000 | -2.306091000 | -1.450434000 |
| H | 1.909939000 | -3.435512000 | 0.325048000 |
| H | 2.679166000 | -0.823042000 | -3.017595000 |
| H | 0.629597000 | 6.200386000 | -1.463099000 |
| C | -2.966662000 | 4.436607000 | -0.163148000 |
| C | -2.076928000 | 5.684324000 | -2.130129000 |
| C | -1.751138000 | 6.575230000 | 0.189943000 |
| H | 6.685001000 | 2.365832000 | 0.109109000 |
| C | 6.355188000 | -0.326292000 | 2.950355000 |
| C | 6.172259000 | -1.390741000 | 0.679349000 |
| C | 7.841429000 | 0.411229000 | 1.096464000 |
| C | 2.874785000 | -3.445675000 | -2.288730000 |
| H | -2.871391000 | 4.219311000 | 0.910568000 |
| H | -3.919102000 | 4.968812000 | -0.297634000 |
| H | -3.046789000 | 3.478494000 | -0.697507000 |
| H | -3.029346000 | 6.229980000 | -2.208030000 |
| H | -1.290868000 | 6.336077000 | -2.539149000 |
| H | -2.146774000 | 4.794977000 | -2.773062000 |
| H | -1.547909000 | 6.331967000 | 1.243752000 |
| H | -0.969129000 | 7.267714000 | -0.155322000 |
| H | -2.713271000 | 7.107795000 | 0.142863000 |
| H | 5.381009000 | -0.736944000 | 3.255197000 |
| H | 7.126131000 | -1.050440000 | 3.254501000 |
| H | 6.521363000 | 0.604441000 | 3.513497000 |
| H | 6.926284000 | -2.144474000 | 0.953734000 |
| H | 5.180100000 | -1.809362000 | 0.907643000 |
| H | 6.225518000 | -1.233422000 | -0.408732000 |
| H | 7.974726000 | 0.563922000 | 0.014720000 |
| H | 8.085064000 | 1.351391000 | 1.614375000 |
| H | 8.579519000 | -0.339898000 | 1.413365000 |
| C | 4.323146000 | -3.130128000 | -2.688793000 |
| C | 2.851237000 | -4.772967000 | -1.532636000 |
| C | 1.996686000 | -3.584285000 | -3.543867000 |
| H | 4.403261000 | -2.194446000 | -3.262190000 |
| H | 4.966468000 | -3.039019000 | -1.800218000 |
| H | 4.725727000 | -3.937292000 | -3.319680000 |
| H | 1.826467000 | -5.055837000 | -1.247937000 |
| H | 3.251324000 | -5.572318000 | -2.173476000 |
| H | 3.471512000 | -4.738567000 | -0.623605000 |
| H | 2.343900000 | -4.430938000 | -4.156049000 |
| H | 0.947023000 | -3.752368000 | -3.261620000 |
| H | 2.037141000 | -2.682724000 | -4.174014000 |

**int6'**

Lowest frequency = 10.0554 $cm^{-1}$

Charge = 0, Multiplicity = 1

134

| | | | |
|---|---|---|---|
| O | 1.077053000 | -2.765212000 | 1.683257000 |
| C | -0.105185000 | -2.965952000 | 1.269529000 |
| O | -0.573618000 | -2.150883000 | 0.425614000 |
| C | 2.164246000 | -2.691788000 | -1.771827000 |
| C | -0.399108000 | 0.319931000 | 2.978358000 |
| N | 0.307570000 | -0.109531000 | 2.173420000 |
| N | 1.933333000 | -2.002666000 | -0.877127000 |
| C | 2.479015000 | -3.525697000 | -2.934295000 |
| C | 1.662162000 | -3.002432000 | -4.124897000 |
| C | 3.984289000 | -3.403804000 | -3.211632000 |
| C | 2.092447000 | -4.975335000 | -2.609263000 |
| H | 0.582797000 | -3.053714000 | -3.919768000 |
| H | 1.923212000 | -1.959300000 | -4.353643000 |
| H | 1.878014000 | -3.617565000 | -5.011292000 |
| H | 4.575817000 | -3.751013000 | -2.352630000 |
| H | 4.243166000 | -4.018870000 | -4.086585000 |
| H | 4.264393000 | -2.361285000 | -3.419437000 |
| H | 2.326520000 | -5.616288000 | -3.472433000 |
| H | 2.648939000 | -5.349413000 | -1.737874000 |
| H | 1.016403000 | -5.059686000 | -2.396834000 |
| C | -1.297502000 | 0.894702000 | 3.981506000 |
| C | -1.959145000 | 2.132001000 | 3.353294000 |
| C | -2.350863000 | -0.166153000 | 4.333321000 |
| C | -0.459417000 | 1.275740000 | 5.209637000 |
| H | -1.205884000 | 2.894266000 | 3.098635000 |
| H | -2.534126000 | 1.874926000 | 2.450993000 |
| H | -2.652918000 | 2.570681000 | 4.085636000 |
| H | -1.882003000 | -1.033230000 | 4.821264000 |
| H | -3.078019000 | 0.274583000 | 5.030760000 |
| H | -2.885445000 | -0.497666000 | 3.431222000 |

| | | | |
|---|---|---|---|
| H | -1.125245000 | 1.697181000 | 5.977351000 |
| H | 0.051545000 | 0.399146000 | 5.633244000 |
| H | 0.301580000 | 2.029343000 | 4.958843000 |
| C | -0.907088000 | -4.130163000 | 1.813918000 |
| C | -1.022806000 | -3.924904000 | 3.330368000 |
| C | -2.293076000 | -4.163394000 | 1.179995000 |
| C | -0.128031000 | -5.416797000 | 1.517656000 |
| H | -0.029818000 | -3.846769000 | 3.795244000 |
| H | -1.589239000 | -3.008860000 | 3.554127000 |
| H | -1.559660000 | -4.773868000 | 3.780730000 |
| H | -2.231536000 | -4.372209000 | 0.102688000 |
| H | -2.893519000 | -4.963068000 | 1.640205000 |
| H | -2.817875000 | -3.205291000 | 1.312855000 |
| H | -0.667837000 | -6.284800000 | 1.926477000 |
| H | -0.014204000 | -5.569918000 | 0.432437000 |
| H | 0.874341000 | -5.381887000 | 1.967862000 |
| Ru | 1.263960000 | -0.957575000 | 0.643692000 |
| C | 3.172298000 | -0.606166000 | 1.346716000 |
| C | 3.435351000 | -0.400406000 | 2.705812000 |
| C | 4.322438000 | -0.629696000 | 0.549524000 |
| C | 4.711696000 | -0.183244000 | 3.227936000 |
| C | 5.610297000 | -0.401283000 | 1.028395000 |
| C | 5.815212000 | -0.174946000 | 2.383522000 |
| F | 4.261041000 | -0.859730000 | -0.768749000 |
| F | 6.642231000 | -0.385699000 | 0.188637000 |
| F | 7.032194000 | 0.040761000 | 2.858953000 |
| F | 4.878266000 | 0.016890000 | 4.528941000 |
| F | 2.457111000 | -0.392607000 | 3.614528000 |
| S | -1.199831000 | 0.861801000 | -0.257166000 |
| C | -2.156638000 | 0.178927000 | -1.592329000 |
| C | -1.363767000 | 2.502232000 | -0.931906000 |
| C | 0.535155000 | 0.521587000 | -0.748990000 |
| C | -2.497734000 | -1.150994000 | -1.708586000 |
| C | -2.603986000 | 1.181168000 | -2.468538000 |
| C | -0.781113000 | 3.630286000 | -0.393257000 |
| C | -2.132799000 | 2.510640000 | -2.101858000 |
| C | 1.536579000 | 1.168817000 | 0.041132000 |
| C | 0.786965000 | 0.365688000 | -2.161251000 |
| C | -3.316191000 | -1.541771000 | -2.780310000 |
| H | -2.147673000 | -1.856274000 | -0.955057000 |
| C | -3.416531000 | 0.786984000 | -3.524869000 |
| C | -0.941307000 | 4.861831000 | -1.046281000 |
| H | -0.195346000 | 3.559362000 | 0.523845000 |
| C | -2.315864000 | 3.737272000 | -2.734715000 |
| C | 2.718460000 | 1.669930000 | -0.603427000 |
| H | 1.265759000 | 1.662683000 | 0.976724000 |
| C | 1.951232000 | 0.810540000 | -2.705281000 |
| H | 0.026572000 | -0.107467000 | -2.785287000 |
| C | -3.757450000 | -0.557558000 | -3.671424000 |
| C | -3.747432000 | -3.001922000 | -2.870283000 |
| H | -3.802163000 | 1.527703000 | -4.228486000 |
| C | -1.722684000 | 4.885839000 | -2.209877000 |
| C | -0.242913000 | 6.096713000 | -0.477618000 |
| H | -2.916621000 | 3.802975000 | -3.644295000 |
| C | 2.955835000 | 1.493322000 | -1.937065000 |
| H | 3.424227000 | 2.218290000 | 0.020178000 |
| H | 2.106280000 | 0.670607000 | -3.777862000 |
| H | -4.408217000 | -0.834760000 | -4.500743000 |
| C | -2.501856000 | -3.901021000 | -2.856189000 |
| C | -4.537704000 | -3.295384000 | -4.145135000 |
| C | -4.640243000 | -3.299422000 | -1.654855000 |
| H | -1.877169000 | 5.828669000 | -2.734641000 |
| C | -0.693028000 | 6.315826000 | 0.974050000 |
| C | 1.275573000 | 5.861607000 | -0.520471000 |
| C | -0.563490000 | 7.357902000 | -1.279742000 |
| C | 4.197459000 | 2.025839000 | -2.646149000 |
| H | -1.872978000 | -3.720494000 | -1.972273000 |
| H | -2.796490000 | -4.962245000 | -2.849071000 |
| H | -1.884566000 | -3.725649000 | -3.752405000 |
| H | -4.801525000 | -4.363294000 | -4.178181000 |
| H | -5.478353000 | -2.725789000 | -4.181228000 |
| H | -3.956293000 | -3.066418000 | -5.052443000 |
| H | -4.120737000 | -3.100664000 | -0.708244000 |
| H | -5.526372000 | -2.649203000 | -1.657948000 |
| H | -4.961988000 | -4.353306000 | -1.666094000 |
| H | -0.444022000 | 5.458886000 | 1.617670000 |
| H | -0.196695000 | 7.202933000 | 1.397621000 |
| H | -1.781155000 | 6.470245000 | 1.029944000 |
| H | 1.811216000 | 6.734419000 | -0.114959000 |
| H | 1.571042000 | 4.981430000 | 0.070353000 |
| H | 1.618336000 | 5.696615000 | -1.553725000 |
| H | -0.226963000 | 7.275981000 | -2.324484000 |
| H | -1.641782000 | 7.578748000 | -1.279117000 |
| H | -0.047498000 | 8.221494000 | -0.834063000 |
| C | 3.761157000 | 3.120105000 | -3.632198000 |
| C | 5.206298000 | 2.617072000 | -1.661622000 |
| C | 4.892355000 | 0.886819000 | -3.408378000 |
| H | 3.050905000 | 2.737474000 | -4.380898000 |
| H | 3.271365000 | 3.950995000 | -3.101030000 |
| H | 4.633419000 | 3.521926000 | -4.171706000 |
| H | 5.555315000 | 1.859965000 | -0.943822000 |
| H | 6.086762000 | 2.986159000 | -2.208829000 |
| H | 4.786999000 | 3.467033000 | -1.102330000 |
| H | 5.776400000 | 1.274044000 | -3.938556000 |
| H | 5.222441000 | 0.102416000 | -2.713104000 |
| H | 4.233597000 | 0.428594000 | -4.161654000 |
| S | -4.419094000 | 0.195140000 | 0.682122000 |
| O | -5.221358000 | -0.188341000 | -0.478687000 |
| O | -3.607788000 | -0.874987000 | 1.298116000 |
| C | -5.682281000 | 0.591697000 | 1.975777000 |
| O | -3.686812000 | 1.481903000 | 0.545954000 |
| F | -6.503010000 | 1.544027000 | 1.555584000 |
| F | -6.399503000 | -0.484544000 | 2.272092000 |
| F | -5.095965000 | 1.017960000 | 3.098979000 |

**int6’’**

Lowest frequency = 10.1523 cm$^{-1}$

Charge = 0, Multiplicity = 1

142

| | | | |
|---|---|---|---|
| O | 0.942751000 | -2.789775000 | 1.727706000 |
| C | -0.300079000 | -2.913758000 | 1.496501000 |
| O | -0.823013000 | -2.126182000 | 0.661481000 |
| C | 1.619021000 | -2.825176000 | -1.854638000 |

| | | | |
|---|---|---|---|
| C | -0.185116000 | 0.403908000 | 3.106142000 |
| N | 0.390758000 | -0.097642000 | 2.241102000 |
| N | 1.518160000 | -2.117898000 | -0.949771000 |
| C | 1.773987000 | -3.679699000 | -3.034564000 |
| C | 0.749300000 | -3.226598000 | -4.084792000 |
| C | 3.205261000 | -3.504169000 | -3.562198000 |
| C | 1.517015000 | -5.134736000 | -2.619157000 |
| H | -0.278837000 | -3.322136000 | -3.705323000 |
| H | 0.915653000 | -2.178007000 | -4.370247000 |
| H | 0.847786000 | -3.852669000 | -4.984311000 |
| H | 3.945972000 | -3.791875000 | -2.802786000 |
| H | 3.346242000 | -4.139943000 | -4.449308000 |
| H | 3.396643000 | -2.459270000 | -3.843933000 |
| H | 1.628534000 | -5.789295000 | -3.496647000 |
| H | 2.232440000 | -5.459730000 | -1.850166000 |
| H | 0.499652000 | -5.259504000 | -2.220016000 |
| C | -0.891891000 | 1.108454000 | 4.178761000 |
| C | -1.472391000 | 2.396265000 | 3.570614000 |
| C | -2.017588000 | 0.204657000 | 4.700578000 |
| C | 0.123755000 | 1.427389000 | 5.284910000 |
| H | -0.667101000 | 3.059438000 | 3.217094000 |
| H | -2.159416000 | 2.180817000 | 2.734835000 |
| H | -2.029974000 | 2.934284000 | 4.352278000 |
| H | -1.605169000 | -0.683783000 | 5.200842000 |
| H | -2.609803000 | 0.768808000 | 5.436889000 |
| H | -2.667918000 | -0.114651000 | 3.871321000 |
| H | -0.390537000 | 1.958327000 | 6.099965000 |
| H | 0.570911000 | 0.509730000 | 5.693478000 |
| H | 0.936673000 | 2.067610000 | 4.911764000 |
| C | -1.098193000 | -3.945381000 | 2.269687000 |
| C | -0.968261000 | -3.578546000 | 3.754469000 |
| C | -2.562496000 | -3.911808000 | 1.849057000 |
| C | -0.473181000 | -5.321076000 | 2.015367000 |
| H | 0.086338000 | -3.542225000 | 4.061955000 |
| H | -1.425392000 | -2.597367000 | 3.948266000 |
| H | -1.493021000 | -4.325979000 | 4.369493000 |
| H | -2.678161000 | -4.264841000 | 0.815766000 |
| H | -3.151330000 | -4.577314000 | 2.499302000 |
| H | -2.970965000 | -2.890997000 | 1.912922000 |
| H | -1.009223000 | -6.090467000 | 2.592515000 |
| H | -0.535290000 | -5.593446000 | 0.949507000 |
| H | 0.585253000 | -5.332362000 | 2.312584000 |
| Ru | 1.095785000 | -1.024555000 | 0.619812000 |
| C | 3.092052000 | -0.766932000 | 1.073726000 |
| C | 3.533379000 | -0.558813000 | 2.385952000 |
| C | 4.133186000 | -0.857541000 | 0.142078000 |
| C | 4.873577000 | -0.397971000 | 2.742162000 |
| C | 5.480077000 | -0.687106000 | 0.453596000 |
| C | 5.862958000 | -0.454275000 | 1.768285000 |
| F | 3.900153000 | -1.101296000 | -1.154907000 |
| F | 6.399350000 | -0.733618000 | -0.507920000 |
| F | 7.139150000 | -0.293011000 | 2.085830000 |
| F | 5.210183000 | -0.190380000 | 4.009361000 |
| F | 2.678857000 | -0.492571000 | 3.410197000 |
| S | -1.365704000 | 0.947257000 | 0.071973000 |
| C | -2.500101000 | 0.375444000 | -1.179278000 |
| C | -1.436322000 | 2.604644000 | -0.582658000 |
| C | 0.264247000 | 0.490831000 | -0.692790000 |

| | | | |
|---|---|---|---|
| C | -2.945356000 | -0.930540000 | -1.302792000 |
| C | -2.891982000 | 1.416728000 | -2.031544000 |
| C | -0.718236000 | 3.674447000 | -0.091235000 |
| C | -2.282722000 | 2.696374000 | -1.693471000 |
| C | 1.403936000 | 1.078330000 | -0.062659000 |
| C | 0.320651000 | 0.291597000 | -2.119584000 |
| C | -3.822625000 | -1.236030000 | -2.354591000 |
| H | -2.598455000 | -1.671918000 | -0.583387000 |
| C | -3.774118000 | 1.108069000 | -3.065538000 |
| C | -0.814830000 | 4.920608000 | -0.729360000 |
| H | -0.074401000 | 3.545740000 | 0.778934000 |
| C | -2.401926000 | 3.937597000 | -2.312919000 |
| C | 2.515562000 | 1.499105000 | -0.867654000 |
| H | 1.295485000 | 1.595837000 | 0.893031000 |
| C | 1.424502000 | 0.657345000 | -2.827230000 |
| H | -0.537676000 | -0.149718000 | -2.628967000 |
| C | -4.218809000 | -0.199704000 | -3.217093000 |
| C | -4.362277000 | -2.647194000 | -2.586010000 |
| H | -4.115268000 | 1.887469000 | -3.750152000 |
| C | -1.672431000 | 5.025585000 | -1.832943000 |
| C | 0.033430000 | 6.082487000 | -0.212773000 |
| H | -3.056877000 | 4.062090000 | -3.178085000 |
| C | 2.558848000 | 1.293732000 | -2.217691000 |
| H | 3.329662000 | 2.012217000 | -0.356019000 |
| H | 1.425769000 | 0.488678000 | -3.907119000 |
| H | -4.907905000 | -0.420465000 | -4.035097000 |
| C | -3.847167000 | -3.622236000 | -1.531945000 |
| C | -3.915678000 | -3.136240000 | -3.971216000 |
| C | -5.896155000 | -2.621635000 | -2.512083000 |
| H | -1.777689000 | 5.982044000 | -2.345070000 |
| C | -0.289877000 | 6.333895000 | 1.267085000 |
| C | 1.516721000 | 5.708298000 | -0.363382000 |
| C | -0.224271000 | 7.372844000 | -0.990827000 |
| C | 3.719866000 | 1.747756000 | -3.097735000 |
| H | -4.164752000 | -3.323433000 | -0.523420000 |
| H | -4.243277000 | -4.630520000 | -1.726140000 |
| H | -2.748108000 | -3.684026000 | -1.532354000 |
| H | -4.296408000 | -4.152618000 | -4.159453000 |
| H | -4.284964000 | -2.485641000 | -4.777990000 |
| H | -2.816751000 | -3.165009000 | -4.040059000 |
| H | -6.233280000 | -2.259778000 | -1.528915000 |
| H | -6.335930000 | -1.967921000 | -3.279858000 |
| H | -6.302021000 | -3.634414000 | -2.663107000 |
| H | -0.078805000 | 5.452687000 | 1.891029000 |
| H | 0.316935000 | 7.167375000 | 1.654341000 |
| H | -1.351898000 | 6.589938000 | 1.399965000 |
| H | 2.158647000 | 6.526790000 | -0.001055000 |
| H | 1.770801000 | 4.803257000 | 0.208585000 |
| H | 1.767503000 | 5.514061000 | -1.417723000 |
| H | 0.028408000 | 7.265887000 | -2.056734000 |
| H | -1.274696000 | 7.692718000 | -0.914214000 |
| H | 0.399976000 | 8.182287000 | -0.583495000 |
| C | 3.212563000 | 2.848890000 | -4.041033000 |
| C | 4.881092000 | 2.298758000 | -2.270200000 |
| C | 4.246879000 | 0.561748000 | -3.919740000 |
| H | 2.387895000 | 2.494035000 | -4.677791000 |
| H | 2.842936000 | 3.713195000 | -3.467593000 |
| H | 4.023230000 | 3.194287000 | -4.702254000 |

| | | | |
|---|---|---|---|
| H | 5.284689000 | 1.534747000 | -1.589216000 |
| H | 5.697261000 | 2.613073000 | -2.938192000 |
| H | 4.583927000 | 3.177281000 | -1.677743000 |
| H | 5.074252000 | 0.889999000 | -4.568208000 |
| H | 4.620845000 | -0.230030000 | -3.255740000 |
| H | 3.470809000 | 0.131802000 | -4.571070000 |
| O | -3.319342000 | -0.449411000 | 1.808040000 |
| C | -5.537579000 | 0.454085000 | 1.481996000 |
| C | -6.070531000 | 0.912509000 | 0.123412000 |
| H | -5.726650000 | 0.244715000 | -0.682543000 |
| H | -7.173319000 | 0.916498000 | 0.115224000 |
| H | -5.705935000 | 1.923898000 | -0.103634000 |
| C | -6.021090000 | 1.413846000 | 2.573243000 |
| H | -7.122676000 | 1.435509000 | 2.619236000 |
| H | -5.647401000 | 1.106380000 | 3.563628000 |
| H | -5.656393000 | 2.431447000 | 2.370335000 |
| C | -5.994324000 | -0.968363000 | 1.781831000 |
| H | -7.094990000 | -1.025637000 | 1.816941000 |
| H | -5.635258000 | -1.666401000 | 1.009868000 |
| H | -5.592043000 | -1.318576000 | 2.742888000 |
| C | -3.992041000 | 0.535910000 | 1.453698000 |
| O | -3.512074000 | 1.638907000 | 1.054737000 |

**TS7**

Lowest frequency = -38.5926 $cm^{-1}$

Charge = 1, Multiplicity = 1

126

| | | | |
|---|---|---|---|
| O | 0.163250000 | -2.949140000 | 1.555151000 |
| C | -1.021903000 | -2.634794000 | 1.878652000 |
| O | -1.457319000 | -1.536160000 | 1.432361000 |
| C | -0.659092000 | -2.720542000 | -2.040940000 |
| C | 1.390684000 | -0.014443000 | 3.209716000 |
| N | 1.110265000 | -0.393773000 | 2.157370000 |
| N | -0.235922000 | -2.162141000 | -1.126775000 |
| C | -1.131161000 | -3.382154000 | -3.259273000 |
| C | -1.649230000 | -2.287770000 | -4.205317000 |
| C | 0.073174000 | -4.110364000 | -3.877676000 |
| C | -2.249595000 | -4.365971000 | -2.890826000 |
| H | -2.494278000 | -1.741644000 | -3.760510000 |
| H | -0.854416000 | -1.568614000 | -4.450132000 |
| H | -1.994568000 | -2.756412000 | -5.138459000 |
| H | 0.446455000 | -4.899864000 | -3.209812000 |
| H | -0.236508000 | -4.576073000 | -4.824726000 |
| H | 0.895229000 | -3.409651000 | -4.082521000 |
| H | -2.598471000 | -4.871239000 | -3.803259000 |
| H | -1.892899000 | -5.132917000 | -2.188304000 |
| H | -3.106149000 | -3.846026000 | -2.436761000 |
| C | 1.757717000 | 0.425213000 | 4.557774000 |
| C | 1.630723000 | 1.954310000 | 4.621915000 |
| C | 0.785437000 | -0.245137000 | 5.541310000 |
| C | 3.203469000 | -0.022848000 | 4.820976000 |
| H | 2.320393000 | 2.442530000 | 3.917273000 |
| H | 0.604253000 | 2.282040000 | 4.400609000 |
| H | 1.887523000 | 2.293490000 | 5.635981000 |
| H | 0.859012000 | -1.340919000 | 5.486728000 |
| H | 1.039979000 | 0.066869000 | 6.564747000 |
| H | -0.254433000 | 0.049450000 | 5.337892000 |
| H | 3.487728000 | 0.269756000 | 5.842380000 |
| H | 3.307423000 | -1.112698000 | 4.726197000 |
| H | 3.902758000 | 0.451843000 | 4.117844000 |
| C | -1.864800000 | -3.566529000 | 2.720151000 |
| C | -0.963865000 | -4.316333000 | 3.702675000 |
| C | -2.935521000 | -2.769556000 | 3.462887000 |
| C | -2.516816000 | -4.556816000 | 1.740315000 |
| H | -0.173381000 | -4.868150000 | 3.176384000 |
| H | -0.480348000 | -3.622355000 | 4.407355000 |
| H | -1.562431000 | -5.030569000 | 4.287166000 |
| H | -3.600516000 | -2.242882000 | 2.765656000 |
| H | -3.548859000 | -3.448915000 | 4.072942000 |
| H | -2.485063000 | -2.022538000 | 4.133901000 |
| H | -3.147803000 | -5.267172000 | 2.295474000 |
| H | -3.153775000 | -4.033793000 | 1.010694000 |
| H | -1.753294000 | -5.129877000 | 1.193491000 |
| Ru | 0.492412000 | -1.209702000 | 0.449106000 |
| C | 2.374352000 | -1.677845000 | -0.185619000 |
| C | 3.346858000 | -2.130933000 | 0.710261000 |
| C | 2.755492000 | -1.673137000 | -1.530937000 |
| C | 4.622065000 | -2.527929000 | 0.307855000 |
| C | 4.021793000 | -2.049358000 | -1.967191000 |
| C | 4.964813000 | -2.488418000 | -1.040897000 |
| F | 1.911070000 | -1.275821000 | -2.488803000 |
| F | 4.325025000 | -2.002122000 | -3.254980000 |
| F | 6.166109000 | -2.857632000 | -1.435701000 |
| F | 5.504175000 | -2.945862000 | 1.198588000 |
| F | 3.095897000 | -2.216465000 | 2.016297000 |
| S | -1.440488000 | 1.537728000 | 1.598320000 |
| C | -2.964260000 | 1.560060000 | 0.731330000 |
| C | -1.010928000 | 3.123755000 | 0.980183000 |
| C | -0.191106000 | 0.485570000 | -0.273185000 |
| C | -3.865860000 | 0.499569000 | 0.686760000 |
| C | -3.111745000 | 2.722341000 | -0.045770000 |
| C | 0.181887000 | 3.785281000 | 1.234267000 |
| C | -1.981973000 | 3.627621000 | 0.093724000 |
| C | 1.126431000 | 0.959831000 | -0.318279000 |
| C | -1.064206000 | 0.630534000 | -1.376769000 |
| C | -4.974764000 | 0.583738000 | -0.158224000 |
| H | -3.657157000 | -0.393065000 | 1.273376000 |
| C | -4.243777000 | 2.816615000 | -0.862390000 |
| C | 0.455648000 | 4.996492000 | 0.586636000 |
| H | 0.906199000 | 3.353405000 | 1.926798000 |
| C | -1.717694000 | 4.845323000 | -0.533582000 |
| C | 1.617470000 | 1.480354000 | -1.546855000 |
| H | 1.716482000 | 1.149442000 | 0.576221000 |
| C | -0.553074000 | 1.167255000 | -2.531166000 |
| H | -2.092084000 | 0.267830000 | -1.329950000 |
| C | -5.147142000 | 1.764234000 | -0.911190000 |
| C | -5.964239000 | -0.570344000 | -0.317690000 |
| H | -4.407595000 | 3.705971000 | -1.474927000 |
| C | -0.517104000 | 5.507696000 | -0.289019000 |
| C | 1.796550000 | 5.688550000 | 0.836491000 |
| H | -2.444496000 | 5.278172000 | -1.224552000 |
| C | 0.803956000 | 1.597438000 | -2.650914000 |

| | | | |
|---|---|---|---|
| H | 2.640791000 | 1.854005000 | -1.563573000 |
| H | -1.211968000 | 1.234925000 | -3.400082000 |
| H | -6.013479000 | 1.855700000 | -1.569573000 |
| C | -5.626800000 | -1.740549000 | 0.605835000 |
| C | -5.905271000 | -1.067837000 | -1.770563000 |
| C | -7.383187000 | -0.083339000 | 0.007525000 |
| H | -0.337249000 | 6.452868000 | -0.800785000 |
| C | 1.953296000 | 5.973879000 | 2.337047000 |
| C | 2.924994000 | 4.757085000 | 0.366696000 |
| C | 1.912244000 | 7.009992000 | 0.076460000 |
| C | 1.290101000 | 2.156134000 | -3.982538000 |
| H | -5.667585000 | -1.448082000 | 1.666073000 |
| H | -6.353789000 | -2.552926000 | 0.459191000 |
| H | -4.625040000 | -2.148076000 | 0.399068000 |
| H | -6.608537000 | -1.901993000 | -1.919352000 |
| H | -6.169377000 | -0.276745000 | -2.487743000 |
| H | -4.892993000 | -1.425571000 | -2.020509000 |
| H | -7.444411000 | 0.286634000 | 1.042078000 |
| H | -7.704848000 | 0.729003000 | -0.660317000 |
| H | -8.103866000 | -0.908106000 | -0.103613000 |
| H | 1.929187000 | 5.051920000 | 2.937811000 |
| H | 2.915713000 | 6.471472000 | 2.532273000 |
| H | 1.149125000 | 6.631611000 | 2.699683000 |
| H | 3.905857000 | 5.231413000 | 0.524830000 |
| H | 2.923960000 | 3.802751000 | 0.915973000 |
| H | 2.823927000 | 4.528086000 | -0.705497000 |
| H | 1.846912000 | 6.864761000 | -1.012563000 |
| H | 1.132395000 | 7.725260000 | 0.378373000 |
| H | 2.886324000 | 7.475094000 | 0.287902000 |
| C | 0.417076000 | 3.355210000 | -4.379681000 |
| C | 2.747332000 | 2.611303000 | -3.911631000 |
| C | 1.179549000 | 1.044067000 | -5.038570000 |
| H | -0.640735000 | 3.077683000 | -4.500231000 |
| H | 0.471814000 | 4.149145000 | -3.618832000 |
| H | 0.759579000 | 3.773283000 | -5.338552000 |
| H | 3.423194000 | 1.779748000 | -3.660317000 |
| H | 3.060526000 | 3.005870000 | -4.889191000 |
| H | 2.889885000 | 3.413530000 | -3.171158000 |
| H | 1.546488000 | 1.405813000 | -6.011293000 |
| H | 1.779948000 | 0.169592000 | -4.745529000 |
| H | 0.138728000 | 0.715015000 | -5.180898000 |

**TS7-s2**

Lowest frequency = 7.4287 $cm^{-1}$

Charge = 1, Multiplicity = 1

126

| | | | |
|---|---|---|---|
| O | 3.299895000 | -1.357580000 | 0.664010000 |
| C | 3.116002000 | -2.250652000 | 1.546516000 |
| O | 1.914659000 | -2.576822000 | 1.782876000 |
| C | 1.851487000 | -3.258239000 | -1.843910000 |
| C | 1.452084000 | 0.937238000 | 2.718974000 |
| N | 1.313236000 | 0.179562000 | 1.863137000 |
| N | 1.482186000 | -2.546337000 | -1.016305000 |
| C | 2.322264000 | -4.062725000 | -2.974193000 |
| C | 1.134467000 | -4.863710000 | -3.526237000 |
| C | 2.848942000 | -3.070135000 | -4.025955000 |
| C | 3.440383000 | -4.993656000 | -2.485697000 |
| H | 0.730711000 | -5.555251000 | -2.772707000 |
| H | 0.328677000 | -4.196548000 | -3.864748000 |
| H | 1.474625000 | -5.455920000 | -4.388207000 |
| H | 3.702267000 | -2.496132000 | -3.636916000 |
| H | 3.183845000 | -3.632647000 | -4.909743000 |
| H | 2.066116000 | -2.363876000 | -4.337325000 |
| H | 3.814695000 | -5.579486000 | -3.337814000 |
| H | 4.280577000 | -4.421924000 | -2.065811000 |
| H | 3.074061000 | -5.694247000 | -1.721396000 |
| C | 1.609969000 | 1.957289000 | 3.757421000 |
| C | 0.313162000 | 2.782129000 | 3.789798000 |
| C | 1.848696000 | 1.257056000 | 5.102322000 |
| C | 2.812463000 | 2.832179000 | 3.370771000 |
| H | 0.111621000 | 3.258489000 | 2.819002000 |
| H | -0.549892000 | 2.156491000 | 4.060498000 |
| H | 0.413711000 | 3.574340000 | 4.546045000 |
| H | 2.763896000 | 0.648045000 | 5.077800000 |
| H | 1.964881000 | 2.019362000 | 5.886428000 |
| H | 1.002913000 | 0.608695000 | 5.373149000 |
| H | 2.936630000 | 3.620570000 | 4.127484000 |
| H | 3.737900000 | 2.240576000 | 3.328838000 |
| H | 2.664686000 | 3.307119000 | 2.391209000 |
| C | 4.284421000 | -2.857111000 | 2.285526000 |
| C | 4.914813000 | -1.727853000 | 3.115061000 |
| C | 3.806866000 | -3.990356000 | 3.188298000 |
| C | 5.290452000 | -3.367128000 | 1.246946000 |
| H | 5.234351000 | -0.896316000 | 2.470635000 |
| H | 4.201852000 | -1.340789000 | 3.860262000 |
| H | 5.793712000 | -2.109140000 | 3.656074000 |
| H | 3.335011000 | -4.794834000 | 2.605756000 |
| H | 4.662047000 | -4.416465000 | 3.733490000 |
| H | 3.070104000 | -3.634548000 | 3.922205000 |
| H | 6.176863000 | -3.775053000 | 1.755071000 |
| H | 4.853670000 | -4.171373000 | 0.634344000 |
| H | 5.613178000 | -2.556423000 | 0.578698000 |
| Ru | 1.203808000 | -1.137109000 | 0.381552000 |
| C | 1.649442000 | 0.183005000 | -1.129082000 |
| C | 2.584983000 | 1.202302000 | -0.940535000 |
| C | 1.222784000 | 0.000332000 | -2.447176000 |
| C | 3.083979000 | 1.973131000 | -1.991269000 |
| C | 1.685810000 | 0.762855000 | -3.516498000 |
| C | 2.622485000 | 1.767159000 | -3.287543000 |
| F | 0.331049000 | -0.946744000 | -2.761769000 |
| F | 1.252673000 | 0.521470000 | -4.742394000 |
| F | 3.075752000 | 2.503011000 | -4.282318000 |
| F | 3.984274000 | 2.912962000 | -1.758341000 |
| F | 3.069146000 | 1.493299000 | 0.265663000 |
| S | -1.564671000 | 1.069759000 | -1.842021000 |
| C | -1.517800000 | 2.601404000 | -0.996037000 |
| C | -3.205952000 | 0.771950000 | -1.302063000 |
| C | -0.637331000 | -0.466798000 | 0.242066000 |
| C | -0.413399000 | 3.449474000 | -0.934399000 |
| C | -2.720677000 | 2.874281000 | -0.316705000 |
| C | -3.948144000 | -0.367628000 | -1.582154000 |
| C | -3.698497000 | 1.812216000 | -0.488886000 |
| C | -0.992584000 | -1.813700000 | 0.082901000 |

| | | | |
|---|---|---|---|
| C | -1.306238000 | 0.369566000 | 1.162636000 |
| C | -0.490164000 | 4.629530000 | -0.190066000 |
| H | 0.496316000 | 3.182069000 | -1.470718000 |
| C | -2.795322000 | 4.058465000 | 0.425126000 |
| C | -5.233521000 | -0.509146000 | -1.045796000 |
| H | -3.524224000 | -1.143750000 | -2.222280000 |
| C | -4.983844000 | 1.673323000 | 0.037974000 |
| C | -1.861917000 | -2.394763000 | 1.054632000 |
| H | -0.826164000 | -2.345409000 | -0.852736000 |
| C | -2.184124000 | -0.217480000 | 2.033913000 |
| H | -1.083015000 | 1.435341000 | 1.223014000 |
| C | -1.700669000 | 4.910778000 | 0.478549000 |
| C | 0.665170000 | 5.630436000 | -0.138730000 |
| H | -3.712801000 | 4.315064000 | 0.959716000 |
| C | -5.728136000 | 0.530131000 | -0.237458000 |
| C | -6.051027000 | -1.758460000 | -1.381634000 |
| H | -5.408266000 | 2.457931000 | 0.668662000 |
| C | -2.452958000 | -1.623178000 | 2.031766000 |
| H | -2.119435000 | -3.448096000 | 0.943059000 |
| H | -2.668204000 | 0.416434000 | 2.780311000 |
| H | -1.791002000 | 5.833912000 | 1.054135000 |
| C | 1.952274000 | 5.040936000 | -0.714190000 |
| C | 0.941647000 | 6.055526000 | 1.309622000 |
| C | 0.266806000 | 6.862608000 | -0.965974000 |
| H | -6.727440000 | 0.450738000 | 0.189967000 |
| C | -6.359340000 | -1.753168000 | -2.886698000 |
| C | -5.242055000 | -3.014903000 | -1.028712000 |
| C | -7.372160000 | -1.805754000 | -0.612863000 |
| C | -3.402321000 | -2.185350000 | 3.083621000 |
| H | 1.857889000 | 4.790179000 | -1.781138000 |
| H | 2.769902000 | 5.771471000 | -0.629303000 |
| H | 2.264042000 | 4.132552000 | -0.177031000 |
| H | 1.772554000 | 6.776662000 | 1.337849000 |
| H | 0.073845000 | 6.539676000 | 1.779958000 |
| H | 1.225699000 | 5.191458000 | 1.929352000 |
| H | 0.068616000 | 6.585547000 | -2.012275000 |
| H | -0.639957000 | 7.341579000 | -0.567188000 |
| H | 1.076472000 | 7.608927000 | -0.956489000 |
| H | -5.439845000 | -1.761137000 | -3.491081000 |
| H | -6.949854000 | -2.641494000 | -3.160276000 |
| H | -6.936608000 | -0.859178000 | -3.166554000 |
| H | -5.824487000 | -3.919923000 | -1.259954000 |
| H | -4.300709000 | -3.074885000 | -1.594909000 |
| H | -4.991155000 | -3.034634000 | 0.042557000 |
| H | -7.214593000 | -1.803304000 | 0.476866000 |
| H | -8.027417000 | -0.959863000 | -0.868606000 |
| H | -7.915482000 | -2.727856000 | -0.866807000 |
| C | -4.764253000 | -1.485260000 | 2.960806000 |
| C | -3.604946000 | -3.691930000 | 2.927464000 |
| C | -2.800294000 | -1.915772000 | 4.472246000 |
| H | -4.690988000 | -0.399367000 | 3.120262000 |
| H | -5.199840000 | -1.642020000 | 1.963105000 |
| H | -5.463281000 | -1.885036000 | 3.711090000 |
| H | -2.657091000 | -4.243628000 | 3.019616000 |
| H | -4.278815000 | -4.058392000 | 3.715491000 |
| H | -4.062880000 | -3.947534000 | 1.959991000 |
| H | -3.463168000 | -2.315094000 | 5.255042000 |
| H | -1.817628000 | -2.400955000 | 4.576093000 |
| H | -2.672017000 | -0.840031000 | 4.665488000 |

**int8**

Lowest frequency = 10.9171 $cm^{-1}$

Charge = 1, Multiplicity = 1

81

| | | | |
|---|---|---|---|
| O | 2.117284000 | 1.024809000 | 1.253439000 |
| C | 1.900412000 | 2.282798000 | 1.164947000 |
| O | 0.875372000 | 2.608706000 | 0.523876000 |
| C | -1.490890000 | 0.376354000 | 2.630074000 |
| C | 2.614269000 | 0.268001000 | -2.109999000 |
| N | 1.757666000 | 0.355749000 | -1.343750000 |
| N | -0.710230000 | 0.469762000 | 1.787018000 |
| C | -2.544912000 | 0.168901000 | 3.625203000 |
| C | -3.865038000 | 0.630367000 | 2.985355000 |
| C | -2.584379000 | -1.338805000 | 3.928935000 |
| C | -2.225232000 | 0.982131000 | 4.886055000 |
| H | -3.839642000 | 1.702708000 | 2.743714000 |
| H | -4.079655000 | 0.067029000 | 2.065748000 |
| H | -4.683591000 | 0.456865000 | 3.699043000 |
| H | -1.644916000 | -1.677566000 | 4.388779000 |
| H | -3.404019000 | -1.536583000 | 4.635008000 |
| H | -2.760795000 | -1.924171000 | 3.015328000 |
| H | -3.020431000 | 0.815059000 | 5.626972000 |
| H | -1.270101000 | 0.671271000 | 5.332980000 |
| H | -2.179379000 | 2.059059000 | 4.669248000 |
| C | 3.745983000 | 0.080060000 | -3.021105000 |
| C | 3.459862000 | 0.809447000 | -4.340226000 |
| C | 4.990534000 | 0.657348000 | -2.327498000 |
| C | 3.900411000 | -1.434175000 | -3.242642000 |
| H | 2.557232000 | 0.416707000 | -4.830041000 |
| H | 3.332603000 | 1.890079000 | -4.182831000 |
| H | 4.310839000 | 0.659286000 | -5.020323000 |
| H | 5.182661000 | 0.151355000 | -1.370594000 |
| H | 5.861774000 | 0.506322000 | -2.981378000 |
| H | 4.882100000 | 1.735363000 | -2.140913000 |
| H | 4.778477000 | -1.611848000 | -3.880470000 |
| H | 4.047055000 | -1.962005000 | -2.289696000 |
| H | 3.019145000 | -1.855976000 | -3.747335000 |
| C | 2.877751000 | 3.264256000 | 1.762856000 |
| C | 4.121235000 | 3.236479000 | 0.857529000 |
| C | 2.258501000 | 4.659004000 | 1.781494000 |
| C | 3.247112000 | 2.802041000 | 3.175840000 |
| H | 4.565410000 | 2.230947000 | 0.825070000 |
| H | 3.871761000 | 3.547457000 | -0.168792000 |
| H | 4.875335000 | 3.935353000 | 1.248974000 |
| H | 1.358597000 | 4.688503000 | 2.413176000 |
| H | 2.983917000 | 5.379366000 | 2.186959000 |
| H | 1.972289000 | 4.984467000 | 0.771754000 |
| H | 3.989068000 | 3.489852000 | 3.607365000 |
| H | 2.366907000 | 2.797138000 | 3.836676000 |
| H | 3.675850000 | 1.790163000 | 3.165224000 |
| Ru | 0.487855000 | 0.426022000 | 0.194727000 |
| C | 0.517897000 | -1.586335000 | 0.294663000 |
| C | 1.709880000 | -2.317907000 | 0.252836000 |

| | | | |
|---|---|---|---|
| C | -0.656476000 | -2.326065000 | 0.474938000 |
| C | 1.739641000 | -3.705282000 | 0.371533000 |
| C | -0.662229000 | -3.711605000 | 0.590268000 |
| C | 0.546891000 | -4.406501000 | 0.545112000 |
| F | -1.844614000 | -1.720014000 | 0.501486000 |
| F | -1.796303000 | -4.367163000 | 0.752932000 |
| F | 0.560206000 | -5.714696000 | 0.658541000 |
| F | 2.885463000 | -4.356620000 | 0.324092000 |
| F | 2.884146000 | -1.707759000 | 0.120051000 |
| C | -0.878481000 | 1.134274000 | -0.975144000 |
| C | -1.130872000 | 0.219508000 | -2.002533000 |
| C | -1.823941000 | 2.103076000 | -0.605884000 |
| C | -2.431451000 | 0.144449000 | -2.518276000 |
| H | -0.359192000 | -0.452225000 | -2.379684000 |
| C | -3.077578000 | 2.042084000 | -1.181111000 |
| H | -1.572977000 | 2.859493000 | 0.138925000 |
| C | -3.422110000 | 1.044235000 | -2.126048000 |
| H | -2.641831000 | -0.614743000 | -3.271648000 |
| H | -3.826651000 | 2.777045000 | -0.878242000 |
| C | -4.842262000 | 0.998820000 | -2.679040000 |
| C | -5.165727000 | 2.339824000 | -3.355844000 |
| C | -5.028682000 | -0.122833000 | -3.700605000 |
| C | -5.808449000 | 0.760289000 | -1.506475000 |
| H | -5.102560000 | 3.186258000 | -2.656389000 |
| H | -4.477067000 | 2.537799000 | -4.191020000 |
| H | -6.190206000 | 2.318976000 | -3.757250000 |
| H | -4.835952000 | -1.114457000 | -3.263434000 |
| H | -6.066625000 | -0.121116000 | -4.063683000 |
| H | -4.375209000 | 0.006286000 | -4.576774000 |
| H | -6.844177000 | 0.717130000 | -1.876410000 |
| H | -5.587978000 | -0.192449000 | -1.000683000 |
| H | -5.757065000 | 1.564953000 | -0.757905000 |

**TS9**

Lowest frequency = -56.1452 cm$^{-1}$

Charge = 1, Multiplicity = 1

81

| | | | |
|---|---|---|---|
| O | -1.533252000 | 1.738697000 | -0.261991000 |
| C | -1.258456000 | 2.305660000 | -1.369777000 |
| O | -0.381790000 | 1.726906000 | -2.077632000 |
| C | -2.594707000 | -1.263782000 | -1.806734000 |
| C | 2.035651000 | 2.220571000 | 0.562481000 |
| N | 1.257246000 | 1.474401000 | 0.157135000 |
| N | -1.628156000 | -0.752823000 | -1.440326000 |
| C | -3.829899000 | -1.962769000 | -2.168880000 |
| C | -3.445796000 | -3.286499000 | -2.847129000 |
| C | -4.590223000 | -2.222831000 | -0.856214000 |
| C | -4.645932000 | -1.069851000 | -3.113613000 |
| H | -2.882757000 | -3.114527000 | -3.775768000 |
| H | -2.843571000 | -3.915711000 | -2.176208000 |
| H | -4.365230000 | -3.834195000 | -3.100047000 |
| H | -4.865307000 | -1.280277000 | -0.361369000 |
| H | -5.513429000 | -2.774534000 | -1.085965000 |
| H | -3.990151000 | -2.824626000 | -0.159086000 |
| H | -5.582642000 | -1.586100000 | -3.369162000 |
| H | -4.901797000 | -0.111902000 | -2.637866000 |
| H | -4.100480000 | -0.866562000 | -4.046362000 |
| C | 3.008493000 | 3.153807000 | 1.134821000 |
| C | 4.234158000 | 2.338605000 | 1.575827000 |
| C | 3.390465000 | 4.182400000 | 0.060873000 |
| C | 2.337372000 | 3.835415000 | 2.338181000 |
| H | 3.965811000 | 1.597314000 | 2.342397000 |
| H | 4.696036000 | 1.815294000 | 0.725848000 |
| H | 4.979531000 | 3.022731000 | 2.006802000 |
| H | 2.512632000 | 4.751170000 | -0.278172000 |
| H | 4.113445000 | 4.891640000 | 0.489392000 |
| H | 3.855358000 | 3.700512000 | -0.811082000 |
| H | 3.058593000 | 4.520901000 | 2.806619000 |
| H | 1.458357000 | 4.417517000 | 2.026526000 |
| H | 2.015477000 | 3.099520000 | 3.088434000 |
| C | -1.903931000 | 3.609288000 | -1.756116000 |
| C | -1.227208000 | 4.685945000 | -0.889134000 |
| C | -1.669127000 | 3.885638000 | -3.238653000 |
| C | -3.398978000 | 3.536360000 | -1.432295000 |
| H | -1.366769000 | 4.477538000 | 0.181765000 |
| H | -0.148165000 | 4.742882000 | -1.101640000 |
| H | -1.668288000 | 5.668714000 | -1.112345000 |
| H | -2.135333000 | 3.114102000 | -3.868876000 |
| H | -2.109060000 | 4.857330000 | -3.506631000 |
| H | -0.596972000 | 3.912088000 | -3.477726000 |
| H | -3.875652000 | 4.499737000 | -1.665297000 |
| H | -3.897790000 | 2.759405000 | -2.032162000 |
| H | -3.566402000 | 3.313207000 | -0.369466000 |
| Ru | -0.129247000 | 0.260679000 | -0.588213000 |
| C | -0.817779000 | -0.566020000 | 1.205907000 |
| C | -0.898966000 | 0.268763000 | 2.323814000 |
| C | -1.425680000 | -1.818525000 | 1.330983000 |
| C | -1.599823000 | -0.092524000 | 3.474278000 |
| C | -2.141705000 | -2.199634000 | 2.462791000 |
| C | -2.230258000 | -1.332141000 | 3.547402000 |
| F | -1.338791000 | -2.728459000 | 0.359195000 |
| F | -2.728975000 | -3.382195000 | 2.504060000 |
| F | -2.898156000 | -1.677826000 | 4.626302000 |
| F | -1.672792000 | 0.742942000 | 4.492281000 |
| F | -0.337814000 | 1.473811000 | 2.334730000 |
| C | 1.023717000 | -1.165765000 | 0.071096000 |
| C | 1.171752000 | -1.693003000 | -1.217308000 |
| C | 2.046191000 | -1.223062000 | 1.021562000 |
| C | 2.474347000 | -2.079322000 | -1.617569000 |
| H | 0.334982000 | -1.944583000 | -1.869521000 |
| C | 3.285973000 | -1.666212000 | 0.604520000 |
| H | 1.886198000 | -0.859909000 | 2.038220000 |
| C | 3.542988000 | -2.063558000 | -0.733526000 |
| H | 2.595599000 | -2.482331000 | -2.623447000 |
| H | 4.102494000 | -1.673197000 | 1.330473000 |
| C | 4.956554000 | -2.482662000 | -1.128287000 |
| C | 5.399338000 | -3.667066000 | -0.256422000 |
| C | 5.041094000 | -2.895880000 | -2.597386000 |
| C | 5.896505000 | -1.287620000 | -0.900710000 |
| H | 5.410381000 | -3.415476000 | 0.814536000 |
| H | 4.731819000 | -4.530985000 | -0.394307000 |
| H | 6.418606000 | -3.977342000 | -0.531985000 |

| | | | |
|---|---|---|---|
| H | 4.746518000 | -2.077827000 | -3.272593000 |
| H | 6.077122000 | -3.169043000 | -2.844738000 |
| H | 4.411837000 | -3.772352000 | -2.814664000 |
| H | 6.926005000 | -1.558130000 | -1.180393000 |
| H | 5.593820000 | -0.424734000 | -1.514150000 |
| H | 5.914755000 | -0.969815000 | 0.152878000 |

**int10**

Lowest frequency = 11.5861 $cm^{-1}$

Charge = 1, Multiplicity = 1

81

| | | | |
|---|---|---|---|
| O | -0.804666000 | 2.077912000 | 0.005297000 |
| C | -0.233206000 | 2.300732000 | -1.108237000 |
| O | 0.368656000 | 1.310493000 | -1.622930000 |
| C | -2.944201000 | -0.307618000 | -1.401379000 |
| C | 2.535168000 | 1.266494000 | 1.095625000 |
| N | 1.575451000 | 0.752951000 | 0.717436000 |
| N | -1.906349000 | -0.232939000 | -0.901182000 |
| C | -4.289941000 | -0.420288000 | -1.969721000 |
| C | -4.600861000 | -1.916306000 | -2.138425000 |
| C | -5.262125000 | 0.235222000 | -0.974125000 |
| C | -4.314006000 | 0.307830000 | -3.321049000 |
| H | -3.913687000 | -2.389432000 | -2.854536000 |
| H | -4.533049000 | -2.448163000 | -1.179409000 |
| H | -5.625499000 | -2.024318000 | -2.522684000 |
| H | -5.031924000 | 1.301034000 | -0.833471000 |
| H | -6.284389000 | 0.151693000 | -1.370813000 |
| H | -5.232865000 | -0.263550000 | 0.004438000 |
| H | -5.324113000 | 0.227713000 | -3.748242000 |
| H | -4.074590000 | 1.374967000 | -3.205546000 |
| H | -3.603857000 | -0.138423000 | -4.031805000 |
| C | 3.724656000 | 1.933174000 | 1.633458000 |
| C | 4.520351000 | 0.901165000 | 2.446115000 |
| C | 4.559003000 | 2.472239000 | 0.463227000 |
| C | 3.237714000 | 3.079997000 | 2.534007000 |
| H | 3.918774000 | 0.490536000 | 3.269687000 |
| H | 4.862089000 | 0.071977000 | 1.810657000 |
| H | 5.405094000 | 1.392177000 | 2.876743000 |
| H | 3.988928000 | 3.198013000 | -0.134545000 |
| H | 5.447299000 | 2.981470000 | 0.864400000 |
| H | 4.895223000 | 1.660529000 | -0.196808000 |
| H | 4.111782000 | 3.595367000 | 2.958285000 |
| H | 2.647766000 | 3.812083000 | 1.963961000 |
| H | 2.620991000 | 2.703276000 | 3.362412000 |
| C | -0.250282000 | 3.668794000 | -1.739314000 |
| C | 0.618745000 | 4.570661000 | -0.847621000 |
| C | 0.317560000 | 3.601177000 | -3.154119000 |
| C | -1.696615000 | 4.176093000 | -1.746921000 |
| H | 0.229394000 | 4.605470000 | 0.180150000 |
| H | 1.659593000 | 4.211644000 | -0.818885000 |
| H | 0.626447000 | 5.593723000 | -1.251923000 |
| H | -0.284675000 | 2.942580000 | -3.796731000 |
| H | 0.320553000 | 4.606397000 | -3.600360000 |
| H | 1.347650000 | 3.218118000 | -3.155168000 |
| H | -1.731318000 | 5.193519000 | -2.163421000 |
| H | -2.338081000 | 3.534838000 | -2.371615000 |
| H | -2.114342000 | 4.200362000 | -0.730830000 |
| Ru | -0.161718000 | 0.106421000 | 0.000157000 |
| C | -0.697065000 | -1.573151000 | 1.323353000 |
| C | -0.675249000 | -0.383338000 | 2.128372000 |
| C | -1.952910000 | -2.212527000 | 1.103943000 |
| C | -1.907888000 | 0.139220000 | 2.638518000 |
| C | -3.109542000 | -1.690619000 | 1.615062000 |
| C | -3.094998000 | -0.476332000 | 2.350739000 |
| F | -2.002095000 | -3.297243000 | 0.348997000 |
| F | -4.276992000 | -2.255591000 | 1.367222000 |
| F | -4.243603000 | 0.014329000 | 2.764608000 |
| F | -1.861521000 | 1.212135000 | 3.393887000 |
| F | 0.390335000 | -0.081625000 | 2.884635000 |
| C | 0.536892000 | -1.968007000 | 0.629293000 |
| C | 0.486084000 | -2.018027000 | -0.792019000 |
| C | 1.791934000 | -2.139281000 | 1.273505000 |
| C | 1.689827000 | -2.167392000 | -1.524912000 |
| H | -0.457273000 | -2.186209000 | -1.311627000 |
| C | 2.928725000 | -2.300894000 | 0.524255000 |
| H | 1.836896000 | -2.143619000 | 2.362887000 |
| C | 2.914762000 | -2.285510000 | -0.899925000 |
| H | 1.615620000 | -2.222994000 | -2.610452000 |
| H | 3.876395000 | -2.452335000 | 1.045823000 |
| C | 4.228126000 | -2.426876000 | -1.663458000 |
| C | 4.921190000 | -3.734092000 | -1.251946000 |
| C | 4.011761000 | -2.441818000 | -3.176300000 |
| C | 5.123664000 | -1.228964000 | -1.311274000 |
| H | 5.156138000 | -3.763300000 | -0.177706000 |
| H | 4.290367000 | -4.605247000 | -1.484152000 |
| H | 5.869718000 | -3.845668000 | -1.798767000 |
| H | 3.537077000 | -1.514862000 | -3.532876000 |
| H | 4.981145000 | -2.533231000 | -3.687454000 |
| H | 3.392728000 | -3.294953000 | -3.492656000 |
| H | 6.082187000 | -1.299565000 | -1.847536000 |
| H | 4.640011000 | -0.282862000 | -1.600510000 |
| H | 5.350986000 | -1.187176000 | -0.235037000 |

**int12**

Lowest frequency = 10.9053 $cm^{-1}$

Charge = -1, Multiplicity = 1

76

| | | | |
|---|---|---|---|
| O | 1.446149000 | 1.582982000 | 0.822095000 |
| C | 1.609153000 | 2.492274000 | -0.047458000 |
| O | 1.177521000 | 2.333684000 | -1.212917000 |
| C | -1.184470000 | -1.462509000 | -2.262003000 |
| O | -0.718596000 | -0.343255000 | -2.108704000 |
| C | 2.982560000 | -1.031438000 | -1.176393000 |
| N | 1.950333000 | -0.535794000 | -0.963216000 |
| C | -3.310251000 | 2.498159000 | 0.690413000 |
| C | -3.421985000 | 1.712880000 | 2.000794000 |
| C | -2.628281000 | 3.841011000 | 0.959693000 |
| C | -4.689900000 | 2.712605000 | 0.080140000 |

| | | | |
|---|---|---|---|
| H | -3.863797000 | 0.718715000 | 1.831318000 |
| H | -2.423607000 | 1.562934000 | 2.436207000 |
| H | -4.052069000 | 2.252630000 | 2.729227000 |
| H | -2.527073000 | 4.426883000 | 0.031499000 |
| H | -3.209949000 | 4.441374000 | 1.681048000 |
| H | -1.621151000 | 3.671951000 | 1.365868000 |
| H | -5.328086000 | 3.306501000 | 0.757232000 |
| H | -4.613310000 | 3.234912000 | -0.884768000 |
| H | -5.183199000 | 1.749274000 | -0.114640000 |
| C | 4.299193000 | -1.675477000 | -1.073766000 |
| C | 4.105939000 | -3.034744000 | -0.382615000 |
| C | 4.921697000 | -1.860260000 | -2.461930000 |
| C | 5.183214000 | -0.768119000 | -0.202883000 |
| H | 3.629663000 | -2.898331000 | 0.598315000 |
| H | 3.466722000 | -3.694828000 | -0.987765000 |
| H | 5.082217000 | -3.526772000 | -0.243612000 |
| H | 5.049689000 | -0.891909000 | -2.968010000 |
| H | 5.910137000 | -2.337983000 | -2.369710000 |
| H | 4.287428000 | -2.496886000 | -3.096650000 |
| H | 6.171703000 | -1.233655000 | -0.058349000 |
| H | 5.323863000 | 0.214437000 | -0.676987000 |
| H | 4.715265000 | -0.607821000 | 0.778765000 |
| C | 2.350251000 | 3.761537000 | 0.367986000 |
| C | 3.745704000 | 3.347869000 | 0.847073000 |
| C | 2.446984000 | 4.723242000 | -0.810756000 |
| C | 1.574196000 | 4.399541000 | 1.524109000 |
| H | 3.667410000 | 2.618893000 | 1.666296000 |
| H | 4.319516000 | 2.880673000 | 0.030344000 |
| H | 4.309981000 | 4.226255000 | 1.202605000 |
| H | 1.446125000 | 4.995972000 | -1.174604000 |
| H | 2.979320000 | 5.641968000 | -0.513051000 |
| H | 2.982786000 | 4.261422000 | -1.652794000 |
| H | 2.095711000 | 5.300451000 | 1.888636000 |
| H | 0.562706000 | 4.691064000 | 1.202604000 |
| H | 1.468990000 | 3.686127000 | 2.353604000 |
| C | -2.241364000 | -1.740126000 | -3.306317000 |
| C | -3.569021000 | -1.807476000 | -2.529106000 |
| C | -2.288417000 | -0.567872000 | -4.282228000 |
| C | -1.960098000 | -3.064453000 | -4.016566000 |
| H | -3.555103000 | -2.620094000 | -1.788213000 |
| H | -3.738694000 | -0.854465000 | -2.005097000 |
| H | -4.394627000 | -1.986959000 | -3.236676000 |
| H | -1.339690000 | -0.464876000 | -4.831077000 |
| H | -3.098962000 | -0.725009000 | -5.011398000 |
| H | -2.468023000 | 0.363099000 | -3.725730000 |
| H | -2.765096000 | -3.277929000 | -4.737378000 |
| H | -1.008864000 | -3.027402000 | -4.571284000 |
| H | -1.906739000 | -3.896156000 | -3.299405000 |
| O | -0.853634000 | -2.467195000 | -1.484713000 |
| H | -0.317464000 | -2.058181000 | -0.749760000 |
| Ru | 0.348296000 | 0.366766000 | -0.477155000 |
| C | -0.146176000 | -1.016342000 | 0.936722000 |
| C | 0.796983000 | -1.488823000 | 1.864325000 |
| C | -1.468334000 | -1.349249000 | 1.276617000 |
| C | 0.473528000 | -2.187451000 | 3.024606000 |
| C | -1.838509000 | -2.029913000 | 2.433146000 |
| C | -0.858693000 | -2.453425000 | 3.324452000 |
| F | -2.475082000 | -1.043944000 | 0.450508000 |
| F | -3.120492000 | -2.289678000 | 2.691810000 |
| F | -1.186117000 | -3.115303000 | 4.434417000 |
| F | 1.429009000 | -2.600241000 | 3.861217000 |
| F | 2.111114000 | -1.273302000 | 1.688655000 |
| C | -2.420789000 | 1.678990000 | -0.284726000 |
| O | -1.246031000 | 1.491018000 | 0.184391000 |
| O | -2.884865000 | 1.288538000 | -1.352493000 |

**TS13**

Lowest frequency = -52.4887 cm$^{-1}$

Charge = -1, Multiplicity = 1

76

| | | | |
|---|---|---|---|
| O | 1.635422000 | -0.525598000 | -1.041926000 |
| C | 2.420962000 | -1.498296000 | -0.780656000 |
| O | 2.241679000 | -2.348611000 | 0.095530000 |
| C | -2.583970000 | 0.253735000 | 1.560238000 |
| O | -1.877075000 | -0.640339000 | 1.113095000 |
| C | 1.774887000 | 0.031193000 | 2.498693000 |
| N | 1.016033000 | -0.076559000 | 1.623336000 |
| C | -1.682485000 | -3.439999000 | -1.306202000 |
| C | -0.718239000 | -3.785447000 | -2.442146000 |
| C | -0.963735000 | -3.611963000 | 0.037026000 |
| C | -2.915281000 | -4.335240000 | -1.356037000 |
| H | -1.210908000 | -3.691096000 | -3.423597000 |
| H | 0.140078000 | -3.099864000 | -2.425170000 |
| H | -0.351236000 | -4.821230000 | -2.341444000 |
| H | -1.599716000 | -3.286903000 | 0.874786000 |
| H | -0.699633000 | -4.671265000 | 0.197698000 |
| H | -0.008894000 | -3.055151000 | 0.070566000 |
| H | -2.626759000 | -5.398839000 | -1.297013000 |
| H | -3.598136000 | -4.111573000 | -0.523417000 |
| H | -3.479096000 | -4.174091000 | -2.286949000 |
| C | 2.965582000 | 0.078101000 | 3.355733000 |
| C | 3.290479000 | 1.544064000 | 3.674159000 |
| C | 2.710735000 | -0.712017000 | 4.644984000 |
| C | 4.109700000 | -0.562298000 | 2.551609000 |
| H | 3.410316000 | 2.121048000 | 2.745778000 |
| H | 2.486276000 | 2.007916000 | 4.265093000 |
| H | 4.226256000 | 1.603676000 | 4.252701000 |
| H | 2.480552000 | -1.763350000 | 4.418694000 |
| H | 3.607333000 | -0.681720000 | 5.284368000 |
| H | 1.867488000 | -0.287235000 | 5.210137000 |
| H | 5.018268000 | -0.621445000 | 3.172778000 |
| H | 3.829766000 | -1.567849000 | 2.205423000 |
| H | 4.328854000 | 0.040712000 | 1.658932000 |
| C | 3.657432000 | -1.553466000 | -1.703042000 |
| C | 4.372898000 | -0.201633000 | -1.660181000 |
| C | 4.592495000 | -2.670214000 | -1.254214000 |
| C | 3.151856000 | -1.820404000 | -3.124750000 |
| H | 3.679513000 | 0.608354000 | -1.923841000 |
| H | 4.761951000 | 0.008193000 | -0.650929000 |
| H | 5.225921000 | -0.190115000 | -2.360019000 |
| H | 4.064578000 | -3.634034000 | -1.232921000 |
| H | 5.456234000 | -2.748874000 | -1.935907000 |
| H | 4.969012000 | -2.485105000 | -0.236710000 |

| | | | |
|---|---|---|---|
| H | 3.993380000 | -1.855725000 | -3.837669000 |
| H | 2.617328000 | -2.782240000 | -3.177350000 |
| H | 2.452835000 | -1.030510000 | -3.433739000 |
| C | -4.043047000 | 0.030132000 | 1.881495000 |
| C | -4.811230000 | 0.578068000 | 0.663298000 |
| C | -4.305173000 | -1.468692000 | 2.006421000 |
| C | -4.433345000 | 0.779503000 | 3.155649000 |
| H | -4.625618000 | 1.653264000 | 0.526975000 |
| H | -4.494349000 | 0.046470000 | -0.248076000 |
| H | -5.890453000 | 0.421424000 | 0.820833000 |
| H | -3.710650000 | -1.917111000 | 2.817302000 |
| H | -5.371451000 | -1.637964000 | 2.222505000 |
| H | -4.049596000 | -1.971911000 | 1.063143000 |
| H | -5.510124000 | 0.649061000 | 3.345077000 |
| H | -3.885373000 | 0.396763000 | 4.031479000 |
| H | -4.225163000 | 1.854840000 | 3.063965000 |
| O | -2.132033000 | 1.473823000 | 1.723487000 |
| H | -1.236766000 | 1.487877000 | 1.295606000 |
| Ru | -0.051103000 | -0.425671000 | 0.095293000 |
| C | -0.116545000 | 1.486784000 | -0.454650000 |
| C | 1.013875000 | 2.324086000 | -0.423310000 |
| C | -1.228112000 | 2.049082000 | -1.107997000 |
| C | 1.045962000 | 3.594458000 | -0.993548000 |
| C | -1.227799000 | 3.317362000 | -1.684410000 |
| C | -0.077281000 | 4.096725000 | -1.641470000 |
| F | -2.385353000 | 1.393117000 | -1.158983000 |
| F | -2.321221000 | 3.795638000 | -2.276837000 |
| F | -0.058127000 | 5.312001000 | -2.187944000 |
| F | 2.152416000 | 4.337573000 | -0.930585000 |
| F | 2.149707000 | 1.949011000 | 0.175729000 |
| C | -2.097027000 | -1.947451000 | -1.425557000 |
| O | -1.104204000 | -1.136975000 | -1.540602000 |
| O | -3.284295000 | -1.643809000 | -1.397657000 |

**int14**

Lowest frequency = 11.88112 $cm^{-1}$

Charge = -1, Multiplicity = 1

76

| | | | |
|---|---|---|---|
| O | 1.511262000 | -0.312277000 | -1.114768000 |
| C | 2.581835000 | -0.978186000 | -0.917914000 |
| O | 2.718868000 | -1.951408000 | -0.173465000 |
| C | -2.839103000 | -0.111521000 | 1.368767000 |
| O | -2.128401000 | -0.902087000 | 0.760962000 |
| C | 1.439731000 | -0.880920000 | 2.517123000 |
| N | 0.751248000 | -0.688225000 | 1.601088000 |
| C | -1.150242000 | -2.929703000 | -1.965438000 |
| C | -0.020078000 | -2.869405000 | -2.998192000 |
| C | -0.546154000 | -3.068990000 | -0.566718000 |
| C | -2.067011000 | -4.116551000 | -2.235672000 |
| H | -0.429225000 | -2.803980000 | -4.018912000 |
| H | 0.602324000 | -1.981475000 | -2.817372000 |
| H | 0.616571000 | -3.768546000 | -2.938104000 |
| H | -1.307088000 | -3.043585000 | 0.223509000 |
| H | -0.003647000 | -4.026191000 | -0.482230000 |
| H | 0.318241000 | -2.372514000 | -0.349768000 |
| H | -1.498372000 | -5.062262000 | -2.223090000 |
| H | -2.864314000 | -4.178461000 | -1.479288000 |
| H | -2.559746000 | -4.004531000 | -3.211350000 |
| C | 2.578411000 | -1.207851000 | 3.386431000 |
| C | 2.195140000 | -1.070420000 | 4.862948000 |
| C | 2.999710000 | -2.647738000 | 3.052116000 |
| C | 3.715465000 | -0.239211000 | 3.026841000 |
| H | 1.889319000 | -0.039940000 | 5.098899000 |
| H | 1.363972000 | -1.744052000 | 5.120578000 |
| H | 3.057679000 | -1.328617000 | 5.497500000 |
| H | 3.192000000 | -2.733127000 | 1.971991000 |
| H | 3.909876000 | -2.906984000 | 3.617263000 |
| H | 2.206452000 | -3.360830000 | 3.323485000 |
| H | 4.596458000 | -0.455322000 | 3.652552000 |
| H | 3.989099000 | -0.360152000 | 1.969975000 |
| H | 3.414051000 | 0.806196000 | 3.185035000 |
| C | 3.782462000 | -0.503249000 | -1.773276000 |
| C | 3.578450000 | 0.923600000 | -2.275043000 |
| C | 5.051948000 | -0.591625000 | -0.927729000 |
| C | 3.884501000 | -1.474018000 | -2.955088000 |
| H | 2.663938000 | 0.998346000 | -2.878331000 |
| H | 3.470139000 | 1.622432000 | -1.432261000 |
| H | 4.440009000 | 1.243764000 | -2.886022000 |
| H | 5.154251000 | -1.598869000 | -0.500204000 |
| H | 5.943146000 | -0.364102000 | -1.536495000 |
| H | 5.018461000 | 0.130545000 | -0.096200000 |
| H | 4.750396000 | -1.224727000 | -3.591937000 |
| H | 4.000576000 | -2.507211000 | -2.593543000 |
| H | 2.977396000 | -1.430498000 | -3.577590000 |
| C | -4.329389000 | -0.338578000 | 1.485788000 |
| C | -4.934108000 | 0.330000000 | 0.235229000 |
| C | -4.610956000 | -1.839634000 | 1.438764000 |
| C | -4.883790000 | 0.293941000 | 2.760670000 |
| H | -4.718298000 | 1.407552000 | 0.218026000 |
| H | -4.521889000 | -0.116438000 | -0.683946000 |
| H | -6.026478000 | 0.186613000 | 0.245951000 |
| H | -4.144832000 | -2.365212000 | 2.286828000 |
| H | -5.697404000 | -2.012279000 | 1.479841000 |
| H | -4.222005000 | -2.270440000 | 0.505910000 |
| H | -5.975129000 | 0.153630000 | 2.798703000 |
| H | -4.449112000 | -0.167377000 | 3.661508000 |
| H | -4.671155000 | 1.371483000 | 2.793920000 |
| O | -2.362758000 | 0.993930000 | 1.881783000 |
| H | -1.435289000 | 1.075289000 | 1.530075000 |
| Ru | -0.220556000 | -0.533560000 | -0.046133000 |
| C | -0.177348000 | 1.491228000 | -0.046136000 |
| C | 1.005110000 | 2.194236000 | 0.237535000 |
| C | -1.195648000 | 2.290665000 | -0.590118000 |
| C | 1.190357000 | 3.544686000 | -0.044733000 |
| C | -1.052256000 | 3.649036000 | -0.868815000 |
| C | 0.156666000 | 4.283706000 | -0.609233000 |
| F | -2.404761000 | 1.784824000 | -0.837231000 |
| F | -2.063659000 | 4.350068000 | -1.380508000 |
| F | 0.315363000 | 5.579353000 | -0.875272000 |
| F | 2.357858000 | 4.136718000 | 0.213800000 |
| F | 2.062401000 | 1.593966000 | 0.798096000 |
| C | -1.955460000 | -1.600844000 | -2.087889000 |
| O | -1.295211000 | -0.534037000 | -1.806835000 |

| | | | |
|---|---|---|---|
| O | -3.118628000 | -1.628395000 | -2.471922000 |

**TS15**

Lowest frequency = -1291.6796 $cm^{-1}$

Charge = -1, Multiplicity = 1

76

| | | | |
|---|---|---|---|
| O | -2.969260000 | -1.008849000 | 0.576033000 |
| C | -3.208287000 | -0.203193000 | -0.376488000 |
| O | -2.325382000 | 0.210452000 | -1.149107000 |
| C | 0.967882000 | 2.420783000 | -1.110211000 |
| O | 0.198297000 | 1.564184000 | -1.509588000 |
| C | -1.083179000 | 1.119933000 | 2.214888000 |
| N | -0.749079000 | 0.660728000 | 1.195645000 |
| C | -0.816851000 | -3.167347000 | -1.068111000 |
| C | -0.661695000 | -2.321924000 | 0.204317000 |
| C | -2.278068000 | -3.273080000 | -1.525332000 |
| C | -0.267794000 | -4.573246000 | -0.838650000 |
| H | 0.321242000 | -2.522424000 | 0.646320000 |
| H | -1.745900000 | -1.303892000 | 0.414371000 |
| H | -1.353611000 | -2.725274000 | 0.973770000 |
| H | -2.343996000 | -3.942354000 | -2.396738000 |
| H | -2.919575000 | -3.668192000 | -0.719587000 |
| H | -2.667090000 | -2.291859000 | -1.831887000 |
| H | -0.867514000 | -5.113484000 | -0.085691000 |
| H | -0.273782000 | -5.133069000 | -1.785576000 |
| H | 0.773045000 | -4.531415000 | -0.479899000 |
| C | -1.478306000 | 1.303896000 | 3.617901000 |
| C | -0.204901000 | 1.476735000 | 4.459271000 |
| C | -2.390430000 | 2.527632000 | 3.759675000 |
| C | -2.223531000 | 0.026106000 | 4.038489000 |
| H | 0.466195000 | 0.617476000 | 4.317808000 |
| H | 0.337537000 | 2.389037000 | 4.168685000 |
| H | -0.467748000 | 1.553143000 | 5.526688000 |
| H | -3.301771000 | 2.408912000 | 3.154573000 |
| H | -2.687756000 | 2.655643000 | 4.812697000 |
| H | -1.876852000 | 3.443301000 | 3.430372000 |
| H | -2.571861000 | 0.118685000 | 5.080156000 |
| H | -3.086406000 | -0.157105000 | 3.381985000 |
| H | -1.557797000 | -0.845141000 | 3.960541000 |
| C | -4.643481000 | 0.286648000 | -0.579349000 |
| C | -4.625575000 | 1.811448000 | -0.429462000 |
| C | -5.065174000 | -0.088688000 | -2.003066000 |
| C | -5.585975000 | -0.340557000 | 0.442295000 |
| H | -4.297605000 | 2.104138000 | 0.581145000 |
| H | -3.928241000 | 2.257024000 | -1.152787000 |
| H | -5.632494000 | 2.227327000 | -0.598021000 |
| H | -5.074881000 | -1.181785000 | -2.135746000 |
| H | -6.076838000 | 0.293187000 | -2.217536000 |
| H | -4.359971000 | 0.332770000 | -2.732997000 |
| H | -6.617713000 | 0.009016000 | 0.273063000 |
| H | -5.570319000 | -1.437490000 | 0.371784000 |
| H | -5.292672000 | -0.074851000 | 1.468671000 |
| C | 1.400969000 | 3.595933000 | -1.964539000 |
| C | 2.916772000 | 3.463031000 | -2.175514000 |
| C | 0.667807000 | 3.537332000 | -3.300216000 |
| C | 1.083701000 | 4.898840000 | -1.222784000 |
| H | 3.455332000 | 3.547760000 | -1.220806000 |
| H | 3.166981000 | 2.487216000 | -2.616880000 |
| H | 3.269708000 | 4.258378000 | -2.850814000 |
| H | -0.420917000 | 3.592222000 | -3.157094000 |
| H | 0.984647000 | 4.377811000 | -3.937377000 |
| H | 0.879189000 | 2.593069000 | -3.820639000 |
| H | 1.426688000 | 5.760129000 | -1.817614000 |
| H | 0.000222000 | 5.007985000 | -1.058701000 |
| H | 1.585024000 | 4.929359000 | -0.244782000 |
| O | 1.515820000 | 2.381606000 | 0.084882000 |
| H | 1.261593000 | 1.502176000 | 0.464150000 |
| Ru | -0.296637000 | -0.245745000 | -0.429981000 |
| C | 1.613463000 | -0.555436000 | 0.234663000 |
| C | 1.942792000 | -0.952212000 | 1.540005000 |
| C | 2.736312000 | -0.395341000 | -0.594614000 |
| C | 3.240503000 | -1.185142000 | 1.992151000 |
| C | 4.049136000 | -0.621374000 | -0.187874000 |
| C | 4.308934000 | -1.027628000 | 1.117632000 |
| F | 2.601835000 | 0.063117000 | -1.842604000 |
| F | 5.067793000 | -0.434432000 | -1.027292000 |
| F | 5.559322000 | -1.241300000 | 1.526764000 |
| F | 3.470231000 | -1.551666000 | 3.255023000 |
| F | 0.986820000 | -1.126664000 | 2.472647000 |
| C | -0.068459000 | -2.512725000 | -2.254149000 |
| O | 0.110520000 | -1.240347000 | -2.155749000 |
| O | 0.248516000 | -3.186270000 | -3.224269000 |

**int16**

Lowest frequency = 11.8394 $cm^{-1}$

Charge = -1, Multiplicity = 1

76

| | | | |
|---|---|---|---|
| O | -3.197625000 | -1.052969000 | 0.313143000 |
| C | -3.313921000 | 0.049759000 | -0.383329000 |
| O | -2.375071000 | 0.549927000 | -0.982759000 |
| C | 0.901038000 | 2.678323000 | -0.197606000 |
| O | 0.116120000 | 2.039042000 | -0.872694000 |
| C | -1.022369000 | 0.155424000 | 2.467348000 |
| N | -0.723060000 | 0.073196000 | 1.335355000 |
| C | -0.856054000 | -2.624715000 | -2.054240000 |
| C | -0.619850000 | -2.220767000 | -0.587169000 |
| C | -2.338075000 | -2.565254000 | -2.448280000 |
| C | -0.335407000 | -4.032749000 | -2.332111000 |
| H | 0.307297000 | -2.708905000 | -0.249229000 |
| H | -2.239797000 | -1.345773000 | 0.201692000 |
| H | -1.385801000 | -2.727271000 | 0.047331000 |
| H | -2.460461000 | -2.893514000 | -3.492094000 |
| H | -2.948506000 | -3.215900000 | -1.798040000 |
| H | -2.725884000 | -1.538415000 | -2.381953000 |
| H | -0.904920000 | -4.783983000 | -1.757300000 |
| H | -0.410993000 | -4.257179000 | -3.406931000 |
| H | 0.724628000 | -4.116950000 | -2.044884000 |
| C | -1.307643000 | -0.249133000 | 3.854064000 |
| C | 0.024888000 | -0.319927000 | 4.615976000 |
| C | -2.253875000 | 0.754664000 | 4.522406000 |

| | | | |
|---|---|---|---|
| C | -1.957530000 | -1.641849000 | 3.802047000 |
| H | 0.712521000 | -1.018150000 | 4.118259000 |
| H | 0.506064000 | 0.668920000 | 4.655161000 |
| H | -0.149539000 | -0.663707000 | 5.648664000 |
| H | -3.208087000 | 0.815707000 | 3.977525000 |
| H | -2.465455000 | 0.444969000 | 5.558523000 |
| H | -1.807488000 | 1.760159000 | 4.545045000 |
| H | -2.187492000 | -1.992993000 | 4.821649000 |
| H | -2.888937000 | -1.614775000 | 3.217480000 |
| H | -1.277124000 | -2.359926000 | 3.322568000 |
| C | -4.707504000 | 0.654971000 | -0.378572000 |
| C | -5.064528000 | 0.997263000 | 1.073903000 |
| C | -4.713653000 | 1.911745000 | -1.241762000 |
| C | -5.689226000 | -0.385702000 | -0.929301000 |
| H | -5.057752000 | 0.096129000 | 1.703861000 |
| H | -4.345642000 | 1.716096000 | 1.497107000 |
| H | -6.068668000 | 1.448283000 | 1.118340000 |
| H | -4.428462000 | 1.679248000 | -2.277425000 |
| H | -5.718596000 | 2.362460000 | -1.242816000 |
| H | -3.992736000 | 2.651578000 | -0.866188000 |
| H | -6.711472000 | 0.024962000 | -0.930046000 |
| H | -5.429995000 | -0.664353000 | -1.962162000 |
| H | -5.676959000 | -1.299601000 | -0.318857000 |
| C | 1.374944000 | 4.071542000 | -0.570241000 |
| C | 2.892745000 | 3.987645000 | -0.790984000 |
| C | 0.672099000 | 4.509226000 | -1.850487000 |
| C | 1.062710000 | 5.033131000 | 0.581237000 |
| H | 3.410378000 | 3.722267000 | 0.141851000 |
| H | 3.138587000 | 3.220308000 | -1.539536000 |
| H | 3.273439000 | 4.960319000 | -1.140956000 |
| H | -0.418776000 | 4.534340000 | -1.714656000 |
| H | 1.015900000 | 5.514092000 | -2.142196000 |
| H | 0.881195000 | 3.807231000 | -2.669506000 |
| H | 1.436588000 | 6.040890000 | 0.339789000 |
| H | -0.022494000 | 5.105279000 | 0.755013000 |
| H | 1.539050000 | 4.699157000 | 1.514142000 |
| O | 1.440623000 | 2.202070000 | 0.905721000 |
| H | 1.161000000 | 1.251755000 | 0.944658000 |
| Ru | -0.288224000 | -0.141417000 | -0.497299000 |
| C | 1.624149000 | -0.604859000 | -0.026759000 |
| C | 2.012840000 | -1.429097000 | 1.041906000 |
| C | 2.713272000 | -0.110390000 | -0.766149000 |
| C | 3.333606000 | -1.743107000 | 1.356873000 |
| C | 4.046271000 | -0.402669000 | -0.490096000 |
| C | 4.366428000 | -1.235535000 | 0.577933000 |
| F | 2.519899000 | 0.756691000 | -1.765517000 |
| F | 5.027600000 | 0.122671000 | -1.225375000 |
| F | 5.637903000 | -1.522008000 | 0.861454000 |
| F | 3.618691000 | -2.522964000 | 2.403151000 |
| F | 1.100789000 | -1.964982000 | 1.876324000 |
| C | -0.148313000 | -1.618559000 | -2.994315000 |
| O | 0.067042000 | -0.456102000 | -2.478305000 |
| O | 0.104222000 | -1.926184000 | -4.150639000 |

**int17**

Lowest frequency = 13.2862 cm$^{-1}$

Charge = 0, Multiplicity = 1

73

| | | | |
|---|---|---|---|
| O | -1.801968000 | -1.432501000 | 0.880145000 |
| C | -2.255978000 | -2.196805000 | -0.025906000 |
| O | -1.742362000 | -2.162135000 | -1.172437000 |
| C | 1.800125000 | -2.516103000 | 0.566296000 |
| C | -2.611328000 | 1.578758000 | -0.961955000 |
| N | -1.771342000 | 0.789405000 | -0.885554000 |
| N | 0.947138000 | -1.848008000 | 0.165187000 |
| C | 2.962327000 | -3.232106000 | 1.103766000 |
| C | 3.904710000 | -3.563452000 | -0.062285000 |
| C | 3.653688000 | -2.286188000 | 2.099777000 |
| C | 2.492181000 | -4.513676000 | 1.802768000 |
| H | 3.413749000 | -4.217033000 | -0.797929000 |
| H | 4.232095000 | -2.647059000 | -0.574590000 |
| H | 4.795050000 | -4.082453000 | 0.324048000 |
| H | 2.989522000 | -2.051234000 | 2.943982000 |
| H | 4.561190000 | -2.769143000 | 2.493216000 |
| H | 3.933721000 | -1.341410000 | 1.612820000 |
| H | 3.362260000 | -5.040717000 | 2.222724000 |
| H | 1.796841000 | -4.284867000 | 2.623237000 |
| H | 1.985567000 | -5.188771000 | 1.097709000 |
| C | -3.653603000 | 2.605424000 | -0.851112000 |
| C | -3.011163000 | 3.842567000 | -0.202501000 |
| C | -4.212769000 | 2.939572000 | -2.238311000 |
| C | -4.751946000 | 2.037480000 | 0.061409000 |
| H | -2.576102000 | 3.587928000 | 0.774012000 |
| H | -2.216477000 | 4.256372000 | -0.840330000 |
| H | -3.779331000 | 4.617575000 | -0.058415000 |
| H | -4.658083000 | 2.052752000 | -2.712403000 |
| H | -4.994082000 | 3.708408000 | -2.141212000 |
| H | -3.426721000 | 3.329544000 | -2.901410000 |
| H | -5.536753000 | 2.796286000 | 0.201970000 |
| H | -5.209066000 | 1.139083000 | -0.378002000 |
| H | -4.338324000 | 1.769234000 | 1.043790000 |
| C | -3.443338000 | -3.094995000 | 0.294033000 |
| C | -4.659522000 | -2.172609000 | 0.457289000 |
| C | -3.675556000 | -4.087662000 | -0.840005000 |
| C | -3.166899000 | -3.823506000 | 1.611252000 |
| H | -4.480850000 | -1.433840000 | 1.252116000 |
| H | -4.867434000 | -1.630254000 | -0.478794000 |
| H | -5.554274000 | -2.760790000 | 0.716555000 |
| H | -2.803395000 | -4.744140000 | -0.975851000 |
| H | -4.552959000 | -4.716789000 | -0.621427000 |
| H | -3.842917000 | -3.565552000 | -1.792360000 |
| H | -4.034395000 | -4.439185000 | 1.897605000 |
| H | -2.293730000 | -4.488387000 | 1.518745000 |
| H | -2.961147000 | -3.103299000 | 2.415225000 |
| Ru | -0.394547000 | -0.539732000 | -0.420811000 |
| C | 0.509212000 | 0.836041000 | 0.783831000 |
| C | -0.227999000 | 1.642188000 | 1.659581000 |
| C | 1.886558000 | 1.072415000 | 0.838495000 |
| C | 0.328098000 | 2.632684000 | 2.466647000 |
| C | 2.489415000 | 2.040427000 | 1.638542000 |
| C | 1.704272000 | 2.839222000 | 2.462042000 |
| F | 2.752275000 | 0.355460000 | 0.089090000 |

| | | | |
|---|---|---|---|
| F | 3.810361000 | 2.206855000 | 1.610638000 |
| F | 2.253515000 | 3.776035000 | 3.225240000 |
| F | -0.449422000 | 3.386638000 | 3.238944000 |
| F | -1.558069000 | 1.508896000 | 1.764786000 |
| C | 1.568830000 | 0.432969000 | -2.603580000 |
| C | 2.691079000 | 0.932193000 | -3.408294000 |
| C | 2.218077000 | 1.238420000 | -4.833932000 |
| C | 3.222802000 | 2.201814000 | -2.723088000 |
| C | 3.776148000 | -0.155398000 | -3.417848000 |
| H | 1.823935000 | 0.336692000 | -5.324928000 |
| H | 1.429078000 | 2.004570000 | -4.833699000 |
| H | 3.063909000 | 1.615167000 | -5.428906000 |
| H | 3.529258000 | 1.986514000 | -1.689816000 |
| H | 4.092680000 | 2.582618000 | -3.280147000 |
| H | 2.454716000 | 2.988423000 | -2.699008000 |
| H | 4.647068000 | 0.199988000 | -3.989565000 |
| H | 4.100626000 | -0.385489000 | -2.392762000 |
| H | 3.407699000 | -1.080776000 | -3.884117000 |
| N | 0.784104000 | 0.061791000 | -1.837895000 |

**int19**

Lowest frequency = 11.0221 $cm^{-1}$

Charge = 1, Multiplicity = 2

73

| | | | |
|---|---|---|---|
| O | -1.495714000 | 1.496876000 | -0.820778000 |
| C | -1.703440000 | 2.419433000 | 0.034043000 |
| O | -1.144527000 | 2.278240000 | 1.157801000 |
| C | 2.164428000 | 2.165068000 | -0.464395000 |
| C | -2.921355000 | -1.162198000 | 1.058180000 |
| N | -1.911823000 | -0.616623000 | 0.945873000 |
| N | 1.271153000 | 1.537950000 | -0.095305000 |
| C | 3.328819000 | 2.902572000 | -0.960877000 |
| C | 4.289077000 | 3.129527000 | 0.216296000 |
| C | 3.985447000 | 2.028954000 | -2.042995000 |
| C | 2.847862000 | 4.238841000 | -1.544558000 |
| H | 3.817913000 | 3.727420000 | 1.009562000 |
| H | 4.627004000 | 2.175207000 | 0.645899000 |
| H | 5.172863000 | 3.674441000 | -0.146215000 |
| H | 3.307587000 | 1.873190000 | -2.894494000 |
| H | 4.889651000 | 2.535874000 | -2.410333000 |
| H | 4.273644000 | 1.046737000 | -1.641600000 |
| H | 3.715624000 | 4.788478000 | -1.937419000 |
| H | 2.137031000 | 4.083484000 | -2.368797000 |
| H | 2.365686000 | 4.861023000 | -0.776814000 |
| C | -4.210405000 | -1.857029000 | 1.081415000 |
| C | -4.042689000 | -3.145838000 | 0.258308000 |
| C | -4.595788000 | -2.171154000 | 2.532721000 |
| C | -5.239392000 | -0.922275000 | 0.424251000 |
| H | -3.711714000 | -2.923155000 | -0.765810000 |
| H | -3.314152000 | -3.824915000 | 0.724319000 |
| H | -5.012103000 | -3.662843000 | 0.209281000 |
| H | -4.698147000 | -1.253462000 | 3.129489000 |
| H | -5.563933000 | -2.692650000 | 2.537020000 |
| H | -3.853486000 | -2.824027000 | 3.013919000 |
| H | -6.212840000 | -1.433079000 | 0.392803000 |
| H | -5.357229000 | 0.008802000 | 0.997168000 |
| H | -4.945615000 | -0.668161000 | -0.604158000 |
| C | -2.556472000 | 3.612296000 | -0.321903000 |
| C | -3.891094000 | 3.095557000 | -0.872464000 |
| C | -2.772916000 | 4.487564000 | 0.908816000 |
| C | -1.804531000 | 4.387777000 | -1.414807000 |
| H | -3.734065000 | 2.443449000 | -1.743055000 |
| H | -4.442416000 | 2.526774000 | -0.107475000 |
| H | -4.520521000 | 3.943961000 | -1.179552000 |
| H | -1.818018000 | 4.844713000 | 1.319291000 |
| H | -3.384028000 | 5.361696000 | 0.639839000 |
| H | -3.292683000 | 3.936707000 | 1.706042000 |
| H | -2.393605000 | 5.266093000 | -1.718313000 |
| H | -0.829670000 | 4.745600000 | -1.047847000 |
| H | -1.636208000 | 3.758690000 | -2.300628000 |
| Ru | -0.283159000 | 0.416962000 | 0.450393000 |
| C | 0.291595000 | -0.975136000 | -0.875502000 |
| C | -0.634008000 | -1.571561000 | -1.748981000 |
| C | 1.621144000 | -1.403635000 | -1.023181000 |
| C | -0.280692000 | -2.552908000 | -2.669968000 |
| C | 2.007970000 | -2.384274000 | -1.927413000 |
| C | 1.050450000 | -2.959132000 | -2.765236000 |
| F | 2.595753000 | -0.902162000 | -0.259079000 |
| F | 3.268561000 | -2.770261000 | -2.003502000 |
| F | 1.400101000 | -3.882282000 | -3.629537000 |
| F | -1.190940000 | -3.097337000 | -3.453799000 |
| F | -1.914451000 | -1.224445000 | -1.729049000 |
| C | 1.619044000 | -0.861715000 | 2.594970000 |
| C | 2.656254000 | -1.477393000 | 3.425446000 |
| C | 2.077678000 | -1.786375000 | 4.812826000 |
| C | 3.093871000 | -2.765529000 | 2.705670000 |
| C | 3.825889000 | -0.485037000 | 3.521633000 |
| H | 1.746246000 | -0.871061000 | 5.323681000 |
| H | 1.228434000 | -2.481454000 | 4.748242000 |
| H | 2.860137000 | -2.257292000 | 5.425441000 |
| H | 3.457177000 | -2.550047000 | 1.690811000 |
| H | 3.909128000 | -3.230653000 | 3.278848000 |
| H | 2.266179000 | -3.485982000 | 2.637790000 |
| H | 4.628656000 | -0.939398000 | 4.120427000 |
| H | 4.228792000 | -0.250354000 | 2.525714000 |
| H | 3.519390000 | 0.450906000 | 4.010291000 |
| N | 0.851885000 | -0.390182000 | 1.872875000 |

**int20**

Lowest frequency = 11.8581 $cm^{-1}$

Charge = 1, Multiplicity = 1

126

| | | | |
|---|---|---|---|
| O | -0.255647000 | 1.910599000 | 2.887768000 |
| C | -0.169290000 | 3.022934000 | 2.278572000 |
| O | -0.131454000 | 2.998518000 | 1.016590000 |
| C | -3.383624000 | 0.969001000 | 1.018946000 |
| C | 2.876706000 | 0.714744000 | 1.262321000 |
| N | 1.724107000 | 0.753903000 | 1.223331000 |

| | | | |
|---|---|---|---|
| N | -2.233705000 | 0.930005000 | 1.098432000 |
| C | -4.839628000 | 0.917721000 | 0.861586000 |
| C | -5.130423000 | 0.862906000 | -0.646870000 |
| C | -5.336362000 | -0.358829000 | 1.558711000 |
| C | -5.459693000 | 2.169526000 | 1.495538000 |
| H | -4.765458000 | 1.766380000 | -1.156632000 |
| H | -4.656846000 | -0.013245000 | -1.112996000 |
| H | -6.217643000 | 0.794799000 | -0.800210000 |
| H | -5.133334000 | -0.325547000 | 2.638687000 |
| H | -6.423362000 | -0.446511000 | 1.414334000 |
| H | -4.850820000 | -1.252777000 | 1.143373000 |
| H | -6.552248000 | 2.129521000 | 1.374985000 |
| H | -5.234737000 | 2.225565000 | 2.570254000 |
| H | -5.092704000 | 3.086715000 | 1.012509000 |
| C | 4.333660000 | 0.583085000 | 1.329569000 |
| C | 4.637542000 | -0.745167000 | 2.041401000 |
| C | 4.876784000 | 0.571068000 | -0.106958000 |
| C | 4.891969000 | 1.773396000 | 2.121970000 |
| H | 4.169956000 | -0.780926000 | 3.035300000 |
| H | 4.267865000 | -1.597287000 | 1.453063000 |
| H | 5.726294000 | -0.850503000 | 2.157501000 |
| H | 4.668857000 | 1.521491000 | -0.620022000 |
| H | 5.967222000 | 0.428916000 | -0.077889000 |
| H | 4.434803000 | -0.247930000 | -0.693019000 |
| H | 5.987093000 | 1.687229000 | 2.177018000 |
| H | 4.644976000 | 2.728275000 | 1.635016000 |
| H | 4.494751000 | 1.789421000 | 3.146963000 |
| C | -0.084700000 | 4.325707000 | 3.046566000 |
| C | 1.330627000 | 4.880894000 | 2.829743000 |
| C | -1.119209000 | 5.294092000 | 2.464285000 |
| C | -0.339562000 | 4.084637000 | 4.531719000 |
| H | 2.094017000 | 4.178678000 | 3.200071000 |
| H | 1.520904000 | 5.074036000 | 1.764204000 |
| H | 1.448706000 | 5.828183000 | 3.377398000 |
| H | -2.142598000 | 4.914479000 | 2.608418000 |
| H | -1.046276000 | 6.269688000 | 2.968123000 |
| H | -0.958139000 | 5.442342000 | 1.387164000 |
| H | -0.265950000 | 5.034391000 | 5.082439000 |
| H | -1.341393000 | 3.664411000 | 4.701134000 |
| H | 0.389959000 | 3.379011000 | 4.952841000 |
| Ru | -0.256829000 | 0.794943000 | 1.144956000 |
| C | -0.389558000 | -1.046213000 | 2.049617000 |
| C | 0.450879000 | -1.382410000 | 3.119176000 |
| C | -1.373506000 | -1.999372000 | 1.791540000 |
| C | 0.362075000 | -2.583112000 | 3.824516000 |
| C | -1.505980000 | -3.209214000 | 2.467220000 |
| C | -0.630374000 | -3.506455000 | 3.505923000 |
| F | -2.265835000 | -1.802131000 | 0.802098000 |
| F | -2.462574000 | -4.064271000 | 2.129417000 |
| F | -0.730208000 | -4.643742000 | 4.168292000 |
| F | 1.207131000 | -2.842780000 | 4.808980000 |
| F | 1.417910000 | -0.555910000 | 3.525863000 |
| S | -0.126678000 | 0.167837000 | -0.974790000 |
| C | -1.477124000 | -0.375623000 | -2.044524000 |
| C | 0.087686000 | 1.648445000 | -1.950075000 |
| C | 1.227005000 | -0.906181000 | -1.450195000 |
| C | -2.109216000 | -1.599476000 | -1.981812000 |
| C | -1.783832000 | 0.608795000 | -2.996455000 |
| C | 1.031233000 | 2.618388000 | -1.666318000 |
| C | -0.899102000 | 1.772402000 | -2.930087000 |
| C | 1.885149000 | -0.744402000 | -2.664921000 |
| C | 1.589594000 | -1.910508000 | -0.554418000 |
| C | -3.157283000 | -1.875123000 | -2.876349000 |
| H | -1.806480000 | -2.338158000 | -1.243435000 |
| C | -2.824778000 | 0.336933000 | -3.879897000 |
| C | 1.026879000 | 3.798429000 | -2.415804000 |
| H | 1.731690000 | 2.467928000 | -0.848547000 |
| C | -0.900881000 | 2.948531000 | -3.681778000 |
| C | 2.935727000 | -1.603585000 | -2.977108000 |
| H | 1.595325000 | 0.047959000 | -3.358132000 |
| C | 2.633684000 | -2.760806000 | -0.895114000 |
| H | 1.072935000 | -2.007039000 | 0.403147000 |
| C | -3.499955000 | -0.882994000 | -3.805664000 |
| C | -3.863744000 | -3.227414000 | -2.790551000 |
| H | -3.106703000 | 1.068398000 | -4.640236000 |
| C | 0.049479000 | 3.931734000 | -3.419361000 |
| C | 1.988387000 | 4.943127000 | -2.105523000 |
| H | -1.645845000 | 3.104976000 | -4.464667000 |
| C | 3.335549000 | -2.625323000 | -2.104053000 |
| H | 3.451613000 | -1.462220000 | -3.926559000 |
| H | 2.910962000 | -3.546166000 | -0.188996000 |
| H | -4.310827000 | -1.064616000 | -4.511013000 |
| C | -2.833389000 | -4.340625000 | -3.034146000 |
| C | -4.978518000 | -3.354901000 | -3.828413000 |
| C | -4.474397000 | -3.388670000 | -1.389071000 |
| H | 0.017257000 | 4.848304000 | -4.011673000 |
| C | 1.167949000 | 6.084313000 | -1.482977000 |
| C | 3.067675000 | 4.516442000 | -1.108190000 |
| C | 2.673262000 | 5.425288000 | -3.390418000 |
| C | 4.494218000 | -3.570195000 | -2.415558000 |
| H | -2.028268000 | -4.327726000 | -2.284228000 |
| H | -3.321087000 | -5.325625000 | -2.977318000 |
| H | -2.373360000 | -4.244026000 | -4.029333000 |
| H | -5.460046000 | -4.338929000 | -3.733070000 |
| H | -5.760283000 | -2.592639000 | -3.687868000 |
| H | -4.595345000 | -3.275338000 | -4.857203000 |
| H | -3.717057000 | -3.354140000 | -0.592518000 |
| H | -5.214793000 | -2.598450000 | -1.189029000 |
| H | -4.990120000 | -4.358077000 | -1.313757000 |
| H | 0.652037000 | 5.737931000 | -0.574711000 |
| H | 1.825087000 | 6.925321000 | -1.212583000 |
| H | 0.406587000 | 6.463426000 | -2.180971000 |
| H | 3.750555000 | 5.357542000 | -0.919644000 |
| H | 2.636104000 | 4.218873000 | -0.140545000 |
| H | 3.670525000 | 3.680136000 | -1.496939000 |
| H | 3.250962000 | 4.615235000 | -3.861557000 |
| H | 1.953311000 | 5.803422000 | -4.130582000 |
| H | 3.366509000 | 6.248693000 | -3.161306000 |
| C | 3.968080000 | -5.013536000 | -2.432996000 |
| C | 5.136226000 | -3.267231000 | -3.769462000 |
| C | 5.563864000 | -3.421064000 | -1.322266000 |
| H | 3.537252000 | -5.308842000 | -1.464983000 |
| H | 3.192313000 | -5.142323000 | -3.202893000 |
| H | 4.789216000 | -5.711214000 | -2.657156000 |
| H | 5.556952000 | -2.250684000 | -3.808412000 |
| H | 5.962046000 | -3.970300000 | -3.951056000 |

| | | | |
|---|---|---|---|
| H | 4.421323000 | -3.380773000 | -4.598547000 |
| H | 6.409295000 | -4.095373000 | -1.526672000 |
| H | 5.953099000 | -2.391782000 | -1.286203000 |
| H | 5.174327000 | -3.673191000 | -0.324680000 |

**int20$^{Tri}$**

Lowest frequency = 12.5833 cm$^{-1}$

Charge = 1, Multiplicity = 3

126

| | | | |
|---|---|---|---|
| O | 2.246825000 | 2.157479000 | -1.827269000 |
| C | 2.617954000 | 2.023696000 | -0.618939000 |
| O | 2.219903000 | 1.042991000 | 0.053137000 |
| C | 4.055781000 | -1.188636000 | -2.003842000 |
| C | -1.601582000 | 1.517233000 | -2.340357000 |
| N | -0.517871000 | 1.144142000 | -2.175788000 |
| N | 3.050660000 | -0.636974000 | -2.135725000 |
| C | 5.296003000 | -1.927165000 | -1.747469000 |
| C | 5.321522000 | -2.243106000 | -0.242462000 |
| C | 5.268294000 | -3.221220000 | -2.573544000 |
| C | 6.491280000 | -1.049866000 | -2.143290000 |
| H | 5.326973000 | -1.320751000 | 0.355997000 |
| H | 4.445946000 | -2.839281000 | 0.051950000 |
| H | 6.232052000 | -2.815615000 | -0.011469000 |
| H | 5.225590000 | -3.007596000 | -3.651160000 |
| H | 6.184993000 | -3.794918000 | -2.372263000 |
| H | 4.404079000 | -3.846122000 | -2.305888000 |
| H | 7.422238000 | -1.597382000 | -1.934891000 |
| H | 6.469674000 | -0.803737000 | -3.214632000 |
| H | 6.505656000 | -0.112494000 | -1.569276000 |
| C | -2.979877000 | 1.930906000 | -2.635074000 |
| C | -3.680393000 | 0.743307000 | -3.315820000 |
| C | -3.695828000 | 2.295972000 | -1.327336000 |
| C | -2.920863000 | 3.139463000 | -3.581583000 |
| H | -3.165960000 | 0.448487000 | -4.240066000 |
| H | -3.717023000 | -0.130011000 | -2.649858000 |
| H | -4.712309000 | 1.034066000 | -3.562156000 |
| H | -3.206531000 | 3.138206000 | -0.817426000 |
| H | -4.728139000 | 2.595135000 | -1.561171000 |
| H | -3.733589000 | 1.442513000 | -0.637139000 |
| H | -3.946763000 | 3.452076000 | -3.825975000 |
| H | -2.402045000 | 3.989848000 | -3.116281000 |
| H | -2.404662000 | 2.886811000 | -4.518508000 |
| C | 3.624028000 | 3.008885000 | -0.038036000 |
| C | 3.536513000 | 4.351685000 | -0.758078000 |
| C | 3.385323000 | 3.171387000 | 1.461159000 |
| C | 5.003124000 | 2.373181000 | -0.279436000 |
| H | 3.705571000 | 4.234589000 | -1.836781000 |
| H | 2.543619000 | 4.807240000 | -0.625931000 |
| H | 4.287426000 | 5.048880000 | -0.355876000 |
| H | 3.396551000 | 2.195481000 | 1.966325000 |
| H | 4.166083000 | 3.808414000 | 1.904189000 |
| H | 2.411516000 | 3.642404000 | 1.658173000 |
| H | 5.795689000 | 3.029500000 | 0.111757000 |
| H | 5.076874000 | 1.400940000 | 0.231599000 |
| H | 5.182588000 | 2.220768000 | -1.354661000 |
| Ru | 1.280502000 | 0.275074000 | -2.106056000 |
| C | 0.353807000 | -1.568714000 | -2.145654000 |
| C | -0.702302000 | -1.879587000 | -3.002756000 |
| C | 0.626897000 | -2.523161000 | -1.167343000 |
| C | -1.493862000 | -3.017357000 | -2.858837000 |
| C | -0.141640000 | -3.669866000 | -0.978987000 |
| C | -1.213581000 | -3.920876000 | -1.832575000 |
| F | 1.618660000 | -2.327296000 | -0.284620000 |
| F | 0.120594000 | -4.503999000 | 0.021975000 |
| F | -1.967070000 | -4.990498000 | -1.664577000 |
| F | -2.524413000 | -3.238118000 | -3.659779000 |
| F | -1.024467000 | -1.046516000 | -3.995970000 |
| S | -0.241181000 | 0.242217000 | 0.925625000 |
| C | 0.373261000 | -0.473831000 | 2.435735000 |
| C | -0.164470000 | 1.879350000 | 1.614987000 |
| C | -1.984775000 | -0.145250000 | 0.976012000 |
| C | 0.631975000 | -1.816461000 | 2.611604000 |
| C | 0.591886000 | 0.506251000 | 3.414637000 |
| C | -0.450820000 | 3.039356000 | 0.915384000 |
| C | 0.262833000 | 1.851864000 | 2.947691000 |
| C | -2.865432000 | 0.384722000 | 1.914948000 |
| C | -2.424979000 | -1.034391000 | -0.002709000 |
| C | 1.150082000 | -2.248752000 | 3.843143000 |
| H | 0.458931000 | -2.525610000 | 1.803396000 |
| C | 1.098583000 | 0.073818000 | 4.637217000 |
| C | -0.360176000 | 4.267177000 | 1.582511000 |
| H | -0.715766000 | 2.986543000 | -0.138648000 |
| C | 0.335078000 | 3.076046000 | 3.614994000 |
| C | -4.207364000 | 0.020367000 | 1.853176000 |
| H | -2.516358000 | 1.082258000 | 2.679170000 |
| C | -3.765758000 | -1.395419000 | -0.036254000 |
| H | -1.726462000 | -1.428211000 | -0.739407000 |
| C | 1.368526000 | -1.281977000 | 4.835582000 |
| C | 1.470690000 | -3.731189000 | 4.024038000 |
| H | 1.296722000 | 0.789554000 | 5.437895000 |
| C | 0.013609000 | 4.248583000 | 2.939379000 |
| C | -0.661473000 | 5.591506000 | 0.882417000 |
| H | 0.654219000 | 3.117117000 | 4.658375000 |
| C | -4.689109000 | -0.869940000 | 0.880063000 |
| H | -4.891374000 | 0.448033000 | 2.585962000 |
| H | -4.091677000 | -2.098402000 | -0.806140000 |
| H | 1.772531000 | -1.587426000 | 5.800718000 |
| C | 0.186860000 | -4.550546000 | 3.820553000 |
| C | 2.031388000 | -4.028702000 | 5.414116000 |
| C | 2.511969000 | -4.141138000 | 2.969168000 |
| H | 0.080398000 | 5.191290000 | 3.485352000 |
| C | -0.806368000 | 5.409058000 | -0.629688000 |
| C | -1.976949000 | 6.148904000 | 1.447592000 |
| C | 0.475628000 | 6.590199000 | 1.141415000 |
| C | -6.161247000 | -1.260025000 | 0.774447000 |
| H | -0.219463000 | -4.433076000 | 2.805033000 |
| H | 0.396909000 | -5.621054000 | 3.967098000 |
| H | -0.591574000 | -4.254665000 | 4.540187000 |
| H | 2.248754000 | -5.103162000 | 5.501747000 |
| H | 2.970843000 | -3.487203000 | 5.603466000 |
| H | 1.315165000 | -3.772691000 | 6.209873000 |
| H | 2.135562000 | -4.002842000 | 1.944894000 |

| | | | |
|---|---|---|---|
| H | 3.436338000 | -3.552918000 | 3.077066000 |
| H | 2.767913000 | -5.205147000 | 3.088202000 |
| H | -1.673993000 | 4.781829000 | -0.884795000 |
| H | -0.964688000 | 6.386542000 | -1.107648000 |
| H | 0.090908000 | 4.956271000 | -1.079613000 |
| H | -2.223313000 | 7.108044000 | 0.966459000 |
| H | -2.811281000 | 5.452635000 | 1.269866000 |
| H | -1.910257000 | 6.324663000 | 2.531778000 |
| H | 0.585490000 | 6.830278000 | 2.208617000 |
| H | 1.438981000 | 6.200511000 | 0.780485000 |
| H | 0.271731000 | 7.534685000 | 0.614948000 |
| C | -6.286122000 | -2.786183000 | 0.896476000 |
| C | -7.009968000 | -0.610292000 | 1.867196000 |
| C | -6.688307000 | -0.802824000 | -0.595176000 |
| H | -5.735252000 | -3.312363000 | 0.103109000 |
| H | -5.902805000 | -3.138233000 | 1.866127000 |
| H | -7.342618000 | -3.084577000 | 0.819544000 |
| H | -6.988463000 | 0.488743000 | 1.808406000 |
| H | -8.058124000 | -0.923038000 | 1.754037000 |
| H | -6.684469000 | -0.912234000 | 2.874350000 |
| H | -7.751539000 | -1.067453000 | -0.699194000 |
| H | -6.597097000 | 0.288620000 | -0.709285000 |
| H | -6.145081000 | -1.278222000 | -1.425500000 |

**TS21$^{ISET-OSS}$**

Lowest frequency = -47.3212 cm$^{-1}$

Charge = 1, Multiplicity = 1

126

| | | | |
|---|---|---|---|
| O | -0.474693000 | 1.673968000 | 2.638803000 |
| C | -0.564613000 | 2.736460000 | 1.938007000 |
| O | -0.638258000 | 2.587052000 | 0.690164000 |
| C | -3.562119000 | 0.515877000 | 1.195339000 |
| C | 2.682712000 | 0.646130000 | 1.168991000 |
| N | 1.543384000 | 0.585889000 | 1.002146000 |
| N | -2.421601000 | 0.393574000 | 1.085641000 |
| C | -5.007610000 | 0.693376000 | 1.350492000 |
| C | -5.606847000 | 0.943461000 | -0.041104000 |
| C | -5.578838000 | -0.585009000 | 1.981482000 |
| C | -5.232603000 | 1.905212000 | 2.268248000 |
| H | -5.192445000 | 1.855074000 | -0.495014000 |
| H | -5.408350000 | 0.103359000 | -0.720387000 |
| H | -6.695298000 | 1.067935000 | 0.056621000 |
| H | -5.154613000 | -0.760142000 | 2.980521000 |
| H | -6.668554000 | -0.476968000 | 2.083921000 |
| H | -5.374058000 | -1.464513000 | 1.355755000 |
| H | -6.314070000 | 2.054408000 | 2.401921000 |
| H | -4.780734000 | 1.748030000 | 3.258242000 |
| H | -4.810111000 | 2.821065000 | 1.829934000 |
| C | 4.115768000 | 0.682248000 | 1.470083000 |
| C | 4.464726000 | -0.643132000 | 2.168093000 |
| C | 4.900419000 | 0.832806000 | 0.160668000 |
| C | 4.360805000 | 1.875131000 | 2.406914000 |
| H | 3.855618000 | -0.793194000 | 3.070074000 |
| H | 4.309645000 | -1.492262000 | 1.488571000 |
| H | 5.525612000 | -0.620750000 | 2.457222000 |
| H | 4.677822000 | 1.789568000 | -0.332443000 |
| H | 5.976045000 | 0.810736000 | 0.389187000 |
| H | 4.677548000 | 0.013237000 | -0.537070000 |
| H | 5.429983000 | 1.911602000 | 2.661947000 |
| H | 4.088600000 | 2.825758000 | 1.925725000 |
| H | 3.785001000 | 1.774532000 | 3.337975000 |
| C | -0.524985000 | 4.092600000 | 2.600861000 |
| C | 0.934717000 | 4.317239000 | 3.029578000 |
| C | -0.961270000 | 5.165671000 | 1.607024000 |
| C | -1.436706000 | 4.072942000 | 3.830623000 |
| H | 1.264049000 | 3.537068000 | 3.731281000 |
| H | 1.607278000 | 4.312549000 | 2.157762000 |
| H | 1.029662000 | 5.294156000 | 3.527019000 |
| H | -2.001044000 | 5.010962000 | 1.282850000 |
| H | -0.891952000 | 6.158485000 | 2.075584000 |
| H | -0.329407000 | 5.156235000 | 0.707927000 |
| H | -1.385473000 | 5.041707000 | 4.349444000 |
| H | -2.485635000 | 3.897836000 | 3.544829000 |
| H | -1.136466000 | 3.283357000 | 4.533285000 |
| Ru | -0.443840000 | 0.425723000 | 0.988176000 |
| C | -0.315454000 | -1.332038000 | 1.965171000 |
| C | 0.581894000 | -1.522038000 | 3.027651000 |
| C | -1.123723000 | -2.434691000 | 1.666593000 |
| C | 0.727228000 | -2.738979000 | 3.686739000 |
| C | -1.003615000 | -3.669182000 | 2.294745000 |
| C | -0.077648000 | -3.819691000 | 3.326745000 |
| F | -2.030839000 | -2.358572000 | 0.689073000 |
| F | -1.770045000 | -4.684620000 | 1.940202000 |
| F | 0.044648000 | -4.972838000 | 3.945007000 |
| F | 1.607955000 | -2.871159000 | 4.661771000 |
| F | 1.353825000 | -0.530484000 | 3.462541000 |
| S | -0.415485000 | -0.288183000 | -1.253530000 |
| C | -2.024646000 | -0.369107000 | -2.010669000 |
| C | -0.073337000 | 1.263923000 | -2.057327000 |
| C | 2.033916000 | -1.317045000 | -1.740181000 |
| C | -2.878118000 | -1.459624000 | -1.969221000 |
| C | -2.288244000 | 0.773836000 | -2.786396000 |
| C | 1.102914000 | 1.981091000 | -1.950493000 |
| C | -1.180543000 | 1.720223000 | -2.790349000 |
| C | 2.886781000 | -0.944187000 | -2.749741000 |
| C | 2.315592000 | -2.256484000 | -0.773130000 |
| C | -4.084040000 | -1.422813000 | -2.684478000 |
| H | -2.600534000 | -2.345161000 | -1.401264000 |
| C | -3.496894000 | 0.818010000 | -3.480168000 |
| C | 1.209008000 | 3.229367000 | -2.578242000 |
| H | 1.932492000 | 1.575850000 | -1.374088000 |
| C | -1.068076000 | 2.955346000 | -3.426824000 |
| C | 4.148327000 | -1.559194000 | -2.773328000 |
| H | 2.613369000 | -0.203473000 | -3.506112000 |
| C | 3.577723000 | -2.854497000 | -0.826808000 |
| H | 1.598560000 | -2.523890000 | 0.008275000 |
| C | -4.375644000 | -0.262483000 | -3.419171000 |
| C | -4.990335000 | -2.655440000 | -2.674366000 |
| H | -3.745842000 | 1.685603000 | -4.095126000 |
| C | 0.107537000 | 3.693979000 | -3.311556000 |
| C | 2.505969000 | 4.021899000 | -2.418905000 |
| H | -1.900780000 | 3.348372000 | -4.014021000 |
| C | 4.515755000 | -2.516204000 | -1.818128000 |

| | | | |
|---|---|---|---|
| H | 4.845576000 | -1.276557000 | -3.563199000 |
| H | 3.829598000 | -3.609028000 | -0.076603000 |
| H | -5.304785000 | -0.202614000 | -3.985927000 |
| C | -4.232119000 | -3.819217000 | -3.332192000 |
| C | -6.288737000 | -2.418420000 | -3.445806000 |
| C | -5.346275000 | -3.030258000 | -1.228362000 |
| H | 0.160956000 | 4.660274000 | -3.812747000 |
| C | 2.727484000 | 4.301208000 | -0.923855000 |
| C | 3.672203000 | 3.191992000 | -2.975981000 |
| C | 2.461680000 | 5.357839000 | -3.159994000 |
| C | 5.885002000 | -3.200679000 | -1.823123000 |
| H | -3.313503000 | -4.072230000 | -2.781971000 |
| H | -4.864801000 | -4.719799000 | -3.357121000 |
| H | -3.949226000 | -3.571112000 | -4.366268000 |
| H | -6.918036000 | -3.319080000 | -3.396289000 |
| H | -6.871366000 | -1.584954000 | -3.023388000 |
| H | -6.103254000 | -2.207611000 | -4.509732000 |
| H | -4.454364000 | -3.204612000 | -0.607922000 |
| H | -5.945598000 | -2.238203000 | -0.753934000 |
| H | -5.945399000 | -3.953382000 | -1.213420000 |
| H | 2.764582000 | 3.372617000 | -0.334661000 |
| H | 3.673962000 | 4.842510000 | -0.769537000 |
| H | 1.910183000 | 4.914845000 | -0.515747000 |
| H | 4.623105000 | 3.731832000 | -2.846413000 |
| H | 3.767022000 | 2.218572000 | -2.471880000 |
| H | 3.536168000 | 2.997859000 | -4.050783000 |
| H | 2.321404000 | 5.220728000 | -4.242827000 |
| H | 1.656816000 | 6.008089000 | -2.785133000 |
| H | 3.411636000 | 5.893597000 | -3.016521000 |
| C | 5.688595000 | -4.713804000 | -1.997768000 |
| C | 6.777341000 | -2.693265000 | -2.956359000 |
| C | 6.593815000 | -2.923081000 | -0.488998000 |
| H | 5.091555000 | -5.148166000 | -1.182223000 |
| H | 5.176363000 | -4.937095000 | -2.945817000 |
| H | 6.662816000 | -5.226589000 | -2.006709000 |
| H | 6.972041000 | -1.612690000 | -2.874589000 |
| H | 7.749529000 | -3.206492000 | -2.919517000 |
| H | 6.338422000 | -2.892734000 | -3.945630000 |
| H | 7.582408000 | -3.407171000 | -0.474049000 |
| H | 6.743882000 | -1.842565000 | -0.338821000 |
| H | 6.024732000 | -3.309760000 | 0.369721000 |

**TS21$^{ISET-Tri}$**

Lowest frequency = -19.5233 cm$^{-1}$

Charge = 1, Multiplicity = 3

126

| | | | |
|---|---|---|---|
| O | -0.208377000 | 1.986767000 | 2.470491000 |
| C | -0.303644000 | 2.997618000 | 1.700109000 |
| O | -0.383046000 | 2.761249000 | 0.465522000 |
| C | -3.363945000 | 0.903053000 | 1.031822000 |
| C | 2.883036000 | 0.648368000 | 1.116558000 |
| N | 1.738346000 | 0.652891000 | 0.981755000 |
| N | -2.229428000 | 0.726893000 | 0.938092000 |
| C | -4.805187000 | 1.114835000 | 1.175874000 |
| C | -5.407749000 | 1.277646000 | -0.226400000 |
| C | -5.383992000 | -0.117273000 | 1.888249000 |
| C | -5.014865000 | 2.384506000 | 2.014861000 |
| H | -4.984080000 | 2.150469000 | -0.743359000 |
| H | -5.223958000 | 0.390270000 | -0.847006000 |
| H | -6.493845000 | 1.423527000 | -0.131647000 |
| H | -4.960871000 | -0.230352000 | 2.896771000 |
| H | -6.473056000 | 0.003796000 | 1.982925000 |
| H | -5.184017000 | -1.035355000 | 1.319036000 |
| H | -6.094293000 | 2.552526000 | 2.142292000 |
| H | -4.560745000 | 2.287352000 | 3.011580000 |
| H | -4.586132000 | 3.266396000 | 1.516996000 |
| C | 4.329421000 | 0.582086000 | 1.329977000 |
| C | 4.599676000 | -0.683997000 | 2.160636000 |
| C | 5.017552000 | 0.495773000 | -0.039721000 |
| C | 4.759924000 | 1.842799000 | 2.093566000 |
| H | 4.043689000 | -0.665892000 | 3.108498000 |
| H | 4.315710000 | -1.585550000 | 1.600255000 |
| H | 5.675269000 | -0.738578000 | 2.383384000 |
| H | 4.855530000 | 1.410837000 | -0.626673000 |
| H | 6.100118000 | 0.376740000 | 0.113960000 |
| H | 4.651097000 | -0.365284000 | -0.617252000 |
| H | 5.844671000 | 1.799080000 | 2.269262000 |
| H | 4.540499000 | 2.753911000 | 1.518365000 |
| H | 4.253644000 | 1.911126000 | 3.067023000 |
| C | -0.282689000 | 4.395185000 | 2.272464000 |
| C | 1.146005000 | 4.637681000 | 2.785126000 |
| C | -0.645214000 | 5.405171000 | 1.187403000 |
| C | -1.275207000 | 4.460491000 | 3.437767000 |
| H | 1.416247000 | 3.904267000 | 3.558539000 |
| H | 1.878721000 | 4.568322000 | 1.966164000 |
| H | 1.218395000 | 5.645448000 | 3.220964000 |
| H | -1.660730000 | 5.230884000 | 0.802643000 |
| H | -0.602200000 | 6.424791000 | 1.597918000 |
| H | 0.045206000 | 5.342353000 | 0.334706000 |
| H | -1.249264000 | 5.460815000 | 3.894954000 |
| H | -2.304407000 | 4.272835000 | 3.093949000 |
| H | -1.029000000 | 3.717027000 | 4.208530000 |
| Ru | -0.247623000 | 0.618735000 | 0.916757000 |
| C | -0.299376000 | -0.996963000 | 2.102005000 |
| C | 0.559903000 | -1.124669000 | 3.208632000 |
| C | -1.215302000 | -2.047363000 | 1.933306000 |
| C | 0.567207000 | -2.244927000 | 4.033791000 |
| C | -1.235600000 | -3.181267000 | 2.736134000 |
| C | -0.345990000 | -3.274763000 | 3.807144000 |
| F | -2.091916000 | -2.025970000 | 0.931719000 |
| F | -2.098143000 | -4.154307000 | 2.508647000 |
| F | -0.356852000 | -4.332176000 | 4.584742000 |
| F | 1.414582000 | -2.329694000 | 5.041997000 |
| F | 1.432893000 | -0.174847000 | 3.517347000 |
| S | -0.254207000 | -0.548034000 | -1.202579000 |
| C | -2.093911000 | -0.521806000 | -1.908516000 |
| C | 0.010132000 | 0.993806000 | -2.101098000 |
| C | 1.488337000 | -1.313103000 | -1.399865000 |
| C | -2.989289000 | -1.566763000 | -1.822056000 |
| C | -2.271454000 | 0.578094000 | -2.745223000 |
| C | 1.191421000 | 1.717597000 | -2.040304000 |
| C | -1.122510000 | 1.475898000 | -2.777363000 |

| | | | |
|---|---|---|---|
| C | 2.194890000 | -1.229495000 | -2.594397000 |
| C | 1.884835000 | -2.212533000 | -0.410470000 |
| C | -4.183694000 | -1.508391000 | -2.562403000 |
| H | -2.767278000 | -2.430457000 | -1.194533000 |
| C | -3.461270000 | 0.647892000 | -3.473629000 |
| C | 1.290573000 | 2.964840000 | -2.666960000 |
| H | 2.042842000 | 1.306140000 | -1.500948000 |
| C | -1.020984000 | 2.714679000 | -3.408406000 |
| C | 3.354155000 | -1.990302000 | -2.759974000 |
| H | 1.864168000 | -0.564465000 | -3.395898000 |
| C | 3.038333000 | -2.965065000 | -0.594569000 |
| H | 1.306576000 | -2.311662000 | 0.511681000 |
| C | -4.396344000 | -0.382147000 | -3.373719000 |
| C | -5.169408000 | -2.675299000 | -2.480381000 |
| H | -3.654569000 | 1.490761000 | -4.141802000 |
| C | 0.163967000 | 3.443972000 | -3.348139000 |
| C | 2.604987000 | 3.737835000 | -2.565358000 |
| H | -1.878420000 | 3.120327000 | -3.949425000 |
| C | 3.807342000 | -2.867751000 | -1.767233000 |
| H | 3.904688000 | -1.891785000 | -3.696029000 |
| H | 3.342290000 | -3.653981000 | 0.197428000 |
| H | -5.307990000 | -0.308591000 | -3.966541000 |
| C | -4.476458000 | -3.941129000 | -3.007672000 |
| C | -6.427791000 | -2.424590000 | -3.311609000 |
| C | -5.588199000 | -2.891899000 | -1.018807000 |
| H | 0.201625000 | 4.412674000 | -3.846401000 |
| C | 2.900325000 | 4.013415000 | -1.083169000 |
| C | 3.731475000 | 2.891358000 | -3.176273000 |
| C | 2.545840000 | 5.074931000 | -3.303384000 |
| C | 5.066442000 | -3.720128000 | -1.923177000 |
| H | -3.589961000 | -4.201515000 | -2.410418000 |
| H | -5.167331000 | -4.797561000 | -2.970431000 |
| H | -4.153484000 | -3.807297000 | -4.051090000 |
| H | -7.113420000 | -3.279022000 | -3.212511000 |
| H | -6.969042000 | -1.526254000 | -2.976491000 |
| H | -6.197768000 | -2.311253000 | -4.381676000 |
| H | -4.725178000 | -3.083852000 | -0.363359000 |
| H | -6.126116000 | -2.013335000 | -0.630369000 |
| H | -6.263173000 | -3.757952000 | -0.940646000 |
| H | 2.945932000 | 3.084850000 | -0.495323000 |
| H | 3.862527000 | 4.537668000 | -0.972690000 |
| H | 2.112792000 | 4.640137000 | -0.639375000 |
| H | 4.696748000 | 3.413621000 | -3.086495000 |
| H | 3.829897000 | 1.913618000 | -2.680826000 |
| H | 3.545177000 | 2.703548000 | -4.244529000 |
| H | 2.352380000 | 4.940873000 | -4.378354000 |
| H | 1.768903000 | 5.736309000 | -2.890838000 |
| H | 3.509179000 | 5.596873000 | -3.204881000 |
| C | 4.671436000 | -5.203978000 | -1.876056000 |
| C | 5.783763000 | -3.447788000 | -3.245580000 |
| C | 6.035623000 | -3.408902000 | -0.772695000 |
| H | 4.191136000 | -5.471584000 | -0.923148000 |
| H | 3.970627000 | -5.450726000 | -2.687826000 |
| H | 5.563343000 | -5.839302000 | -1.989735000 |
| H | 6.100761000 | -2.396982000 | -3.332186000 |
| H | 6.687403000 | -4.071350000 | -3.311828000 |
| H | 5.152783000 | -3.692246000 | -4.113369000 |
| H | 6.944751000 | -4.022638000 | -0.864952000 |
| H | 6.339959000 | -2.350884000 | -0.786419000 |
| H | 5.592496000 | -3.623377000 | 0.211410000 |

**int22**

Lowest frequency = 12.8842 $cm^{-1}$

Charge = 1, Multiplicity = 2

103

| | | | |
|---|---|---|---|
| O | -1.404895000 | 1.140809000 | 1.919452000 |
| C | -1.796318000 | -0.033600000 | 2.222161000 |
| O | -1.443295000 | -0.963542000 | 1.448579000 |
| C | 1.993304000 | -0.185746000 | 2.074530000 |
| C | -3.068969000 | 1.381051000 | -1.236055000 |
| N | -2.083279000 | 0.979553000 | -0.790922000 |
| N | 1.154415000 | 0.066370000 | 1.326170000 |
| C | 3.058215000 | -0.471234000 | 3.038263000 |
| C | 3.694474000 | -1.818268000 | 2.668679000 |
| C | 4.083424000 | 0.669679000 | 2.945940000 |
| C | 2.424208000 | -0.529001000 | 4.436582000 |
| H | 2.958901000 | -2.633877000 | 2.717231000 |
| H | 4.115142000 | -1.797265000 | 1.654246000 |
| H | 4.503181000 | -2.036958000 | 3.381310000 |
| H | 3.632884000 | 1.633861000 | 3.221991000 |
| H | 4.910219000 | 0.463918000 | 3.641417000 |
| H | 4.494846000 | 0.753932000 | 1.930560000 |
| H | 3.212414000 | -0.731774000 | 5.176329000 |
| H | 1.942539000 | 0.424125000 | 4.698608000 |
| H | 1.675663000 | -1.331927000 | 4.502453000 |
| C | -4.303883000 | 1.984236000 | -1.743501000 |
| C | -3.924077000 | 3.341996000 | -2.358832000 |
| C | -4.930769000 | 1.057527000 | -2.793424000 |
| C | -5.243574000 | 2.173417000 | -0.541597000 |
| H | -3.424993000 | 3.986166000 | -1.621719000 |
| H | -3.257936000 | 3.214967000 | -3.224371000 |
| H | -4.840910000 | 3.843846000 | -2.700900000 |
| H | -5.207329000 | 0.086029000 | -2.359549000 |
| H | -5.844762000 | 1.529285000 | -3.182259000 |
| H | -4.249261000 | 0.886630000 | -3.639416000 |
| H | -6.171526000 | 2.650706000 | -0.888860000 |
| H | -5.502359000 | 1.209587000 | -0.079889000 |
| H | -4.785190000 | 2.817430000 | 0.222210000 |
| C | -2.670849000 | -0.258314000 | 3.432888000 |
| C | -4.052679000 | 0.319838000 | 3.087082000 |
| C | -2.771879000 | -1.752220000 | 3.728354000 |
| C | -2.068801000 | 0.498304000 | 4.620920000 |
| H | -3.984309000 | 1.392845000 | 2.856176000 |
| H | -4.493554000 | -0.198813000 | 2.221653000 |
| H | -4.733402000 | 0.191655000 | 3.942006000 |
| H | -1.786732000 | -2.179839000 | 3.965952000 |
| H | -3.435598000 | -1.917803000 | 4.589744000 |
| H | -3.172162000 | -2.304245000 | 2.866612000 |
| H | -2.711954000 | 0.377236000 | 5.505196000 |
| H | -1.069976000 | 0.109868000 | 4.874164000 |
| H | -1.974923000 | 1.570717000 | 4.400758000 |
| Ru | -0.416185000 | 0.516033000 | 0.203795000 |

| | | | |
|---|---|---|---|
| C | 0.370043000 | 2.313827000 | -0.212953000 |
| C | -0.397303000 | 3.489902000 | -0.133725000 |
| C | 1.725237000 | 2.509440000 | -0.516949000 |
| C | 0.124068000 | 4.752584000 | -0.396769000 |
| C | 2.281156000 | 3.752793000 | -0.788862000 |
| C | 1.474814000 | 4.889368000 | -0.717150000 |
| F | 2.553319000 | 1.467930000 | -0.602035000 |
| F | 3.559680000 | 3.866778000 | -1.097901000 |
| F | 1.980266000 | 6.075361000 | -0.961937000 |
| F | -0.646898000 | 5.821504000 | -0.330771000 |
| F | -1.681021000 | 3.455719000 | 0.205048000 |
| S | 0.400981000 | -0.733508000 | -1.683590000 |
| C | 1.875090000 | -1.643715000 | -1.279179000 |
| C | -0.615731000 | -2.152665000 | -1.315633000 |
| C | 3.171537000 | -1.198335000 | -1.474429000 |
| C | 1.577508000 | -2.941218000 | -0.825757000 |
| C | -1.992063000 | -2.211827000 | -1.443728000 |
| C | 0.148942000 | -3.222107000 | -0.819481000 |
| C | 4.248282000 | -2.046250000 | -1.176205000 |
| H | 3.350666000 | -0.199672000 | -1.868559000 |
| C | 2.649005000 | -3.781492000 | -0.523411000 |
| C | -2.675424000 | -3.363487000 | -1.032209000 |
| H | -2.539012000 | -1.359697000 | -1.844655000 |
| C | -0.530658000 | -4.376340000 | -0.433640000 |
| C | 3.956970000 | -3.332014000 | -0.693431000 |
| C | 5.673636000 | -1.546787000 | -1.419465000 |
| H | 2.466107000 | -4.801199000 | -0.177549000 |
| C | -1.918833000 | -4.433347000 | -0.531820000 |
| C | -4.201571000 | -3.386942000 | -1.119114000 |
| H | 0.022032000 | -5.236833000 | -0.050535000 |
| H | 4.771624000 | -4.017163000 | -0.458947000 |
| C | 5.839431000 | -1.240166000 | -2.915871000 |
| C | 6.720832000 | -2.584184000 | -1.014356000 |
| C | 5.915037000 | -0.267368000 | -0.603960000 |
| H | -2.421236000 | -5.345089000 | -0.209408000 |
| C | -4.755031000 | -2.289942000 | -0.195524000 |
| C | -4.636116000 | -3.119467000 | -2.567141000 |
| C | -4.783859000 | -4.730045000 | -0.679137000 |
| H | 5.147977000 | -0.453710000 | -3.253247000 |
| H | 6.863456000 | -0.892407000 | -3.121286000 |
| H | 5.654483000 | -2.137525000 | -3.525308000 |
| H | 7.728617000 | -2.181369000 | -1.192450000 |
| H | 6.653301000 | -2.841422000 | 0.054162000 |
| H | 6.630101000 | -3.510994000 | -1.600593000 |
| H | 5.189259000 | 0.525096000 | -0.840794000 |
| H | 5.855488000 | -0.473888000 | 0.475841000 |
| H | 6.920304000 | 0.130104000 | -0.811132000 |
| H | -4.359964000 | -1.296724000 | -0.457857000 |
| H | -5.853814000 | -2.251178000 | -0.258961000 |
| H | -4.477518000 | -2.484536000 | 0.851111000 |
| H | -5.734560000 | -3.119658000 | -2.641806000 |
| H | -4.277385000 | -2.147129000 | -2.936594000 |
| H | -4.249286000 | -3.895475000 | -3.244500000 |
| H | -4.425651000 | -5.557042000 | -1.310597000 |
| H | -4.538503000 | -4.961088000 | 0.368436000 |
| H | -5.880458000 | -4.703572000 | -0.761119000 |

**int23**

Lowest frequency = 10.2299 cm$^{-1}$

Charge = 0, Multiplicity = 1

67

| | | | |
|---|---|---|---|
| O | -1.596945000 | 2.440832000 | 0.179455000 |
| C | -0.561999000 | 3.067583000 | -0.222601000 |
| O | 0.294117000 | 2.439983000 | -0.896238000 |
| C | 0.615682000 | -0.022033000 | 1.917808000 |
| N | -0.030013000 | 0.220063000 | 0.988949000 |
| C | 1.419807000 | -0.420514000 | 3.076741000 |
| C | 0.878351000 | -1.767134000 | 3.580117000 |
| C | 2.879685000 | -0.558948000 | 2.620655000 |
| C | 1.289125000 | 0.657467000 | 4.161313000 |
| H | -0.179561000 | -1.687414000 | 3.865730000 |
| H | 0.960950000 | -2.539058000 | 2.801717000 |
| H | 1.461342000 | -2.088119000 | 4.456725000 |
| H | 3.274065000 | 0.399075000 | 2.252883000 |
| H | 3.497534000 | -0.886032000 | 3.470974000 |
| H | 2.977033000 | -1.297556000 | 1.811485000 |
| H | 1.894217000 | 0.370775000 | 5.034638000 |
| H | 1.645670000 | 1.632425000 | 3.797651000 |
| H | 0.244253000 | 0.768621000 | 4.485074000 |
| C | -0.347940000 | 4.511529000 | 0.191768000 |
| C | 0.442711000 | 4.445436000 | 1.509609000 |
| C | 0.471422000 | 5.235269000 | -0.874763000 |
| C | -1.691123000 | 5.201884000 | 0.418581000 |
| H | -0.130188000 | 3.913165000 | 2.284164000 |
| H | 1.400542000 | 3.922767000 | 1.363776000 |
| H | 0.654592000 | 5.462881000 | 1.873775000 |
| H | -0.077424000 | 5.290037000 | -1.827101000 |
| H | 0.692438000 | 6.263229000 | -0.548342000 |
| H | 1.418825000 | 4.712061000 | -1.063567000 |
| H | -1.530453000 | 6.231610000 | 0.773742000 |
| H | -2.275315000 | 5.248589000 | -0.512780000 |
| H | -2.291429000 | 4.660574000 | 1.162365000 |
| Ru | -1.011044000 | 0.606676000 | -0.559182000 |
| C | -2.603779000 | -0.521996000 | -0.097635000 |
| C | -2.876614000 | -1.414364000 | 0.933268000 |
| C | -3.639519000 | -0.312165000 | -1.003354000 |
| C | -4.120158000 | -2.035663000 | 1.064690000 |
| C | -4.891720000 | -0.902318000 | -0.919331000 |
| C | -5.128749000 | -1.783303000 | 0.137548000 |
| F | -3.373172000 | 0.533125000 | -2.027413000 |
| F | -5.843796000 | -0.660739000 | -1.809663000 |
| F | -6.308847000 | -2.375735000 | 0.258684000 |
| F | -4.350935000 | -2.877591000 | 2.065060000 |
| F | -1.951601000 | -1.718380000 | 1.852904000 |
| C | 1.875300000 | -1.265569000 | -1.316732000 |
| C | 2.630618000 | -2.419435000 | -1.169368000 |
| C | 2.440223000 | 0.000833000 | -1.217766000 |
| C | 3.998118000 | -2.298044000 | -0.916090000 |
| H | 2.166360000 | -3.404861000 | -1.243071000 |
| C | 3.804780000 | 0.089199000 | -0.963209000 |
| H | 1.828457000 | 0.902702000 | -1.305672000 |
| C | 4.614511000 | -1.046840000 | -0.806704000 |
| H | 4.581255000 | -3.211966000 | -0.801114000 |

| | | | |
|---|---|---|---|
| H | 4.243686000 | 1.086423000 | -0.882862000 |
| C | 6.107961000 | -0.881567000 | -0.522000000 |
| C | 6.287329000 | -0.110794000 | 0.794310000 |
| C | 6.821379000 | -2.227777000 | -0.393886000 |
| C | 6.754934000 | -0.095901000 | -1.672033000 |
| H | 5.821629000 | 0.885050000 | 0.752879000 |
| H | 5.836987000 | -0.659242000 | 1.635886000 |
| H | 7.357662000 | 0.030028000 | 1.012403000 |
| H | 6.746670000 | -2.820373000 | -1.318398000 |
| H | 7.890538000 | -2.063047000 | -0.192666000 |
| H | 6.418234000 | -2.828577000 | 0.435801000 |
| H | 7.832720000 | 0.034654000 | -1.486661000 |
| H | 6.633554000 | -0.627282000 | -2.628204000 |
| H | 6.312139000 | 0.904491000 | -1.785759000 |
| Br | -0.015861000 | -1.449757000 | -1.633760000 |

**TS24**

Lowest frequency = -33.8247 $cm^{-1}$

Charge = 0, Multiplicity = 1

67

| | | | |
|---|---|---|---|
| O | -0.416872000 | -0.726564000 | -1.733493000 |
| C | -0.989350000 | -1.851495000 | -1.507611000 |
| O | -0.917666000 | -2.325966000 | -0.353639000 |
| C | 2.769719000 | -2.047812000 | 0.109422000 |
| N | 1.767497000 | -1.481972000 | 0.058249000 |
| C | 4.101353000 | -2.637290000 | 0.259170000 |
| C | 5.080071000 | -1.762207000 | -0.541153000 |
| C | 4.436505000 | -2.588136000 | 1.759412000 |
| C | 4.091786000 | -4.079287000 | -0.260712000 |
| H | 4.844029000 | -1.779877000 | -1.615076000 |
| H | 5.048523000 | -0.721023000 | -0.191357000 |
| H | 6.099747000 | -2.151850000 | -0.403952000 |
| H | 3.725645000 | -3.187106000 | 2.346025000 |
| H | 5.448225000 | -2.993327000 | 1.911020000 |
| H | 4.405565000 | -1.555267000 | 2.132833000 |
| H | 5.095205000 | -4.512321000 | -0.135296000 |
| H | 3.375818000 | -4.700001000 | 0.297023000 |
| H | 3.830948000 | -4.118253000 | -1.328301000 |
| C | -1.810192000 | -2.496100000 | -2.606267000 |
| C | -1.968989000 | -3.987634000 | -2.323915000 |
| C | -3.179421000 | -1.795863000 | -2.552226000 |
| C | -1.150160000 | -2.255944000 | -3.963549000 |
| H | -0.996904000 | -4.503032000 | -2.348696000 |
| H | -2.410711000 | -4.155113000 | -1.332066000 |
| H | -2.619842000 | -4.446902000 | -3.083605000 |
| H | -3.076540000 | -0.717222000 | -2.742244000 |
| H | -3.848785000 | -2.223091000 | -3.315050000 |
| H | -3.648649000 | -1.927639000 | -1.564984000 |
| H | -1.778811000 | -2.671033000 | -4.766325000 |
| H | -1.008080000 | -1.182162000 | -4.147030000 |
| H | -0.164205000 | -2.742698000 | -4.017827000 |
| Ru | 0.164771000 | -0.381461000 | 0.183516000 |
| C | 1.234476000 | 1.301116000 | -0.206694000 |
| C | 2.570043000 | 1.532171000 | 0.137438000 |
| C | 0.665753000 | 2.267081000 | -1.045551000 |
| C | 3.289519000 | 2.639516000 | -0.309057000 |
| C | 1.348237000 | 3.394828000 | -1.495948000 |
| C | 2.678215000 | 3.580013000 | -1.130988000 |
| F | -0.601144000 | 2.149079000 | -1.446070000 |
| F | 0.749702000 | 4.281041000 | -2.279202000 |
| F | 3.351084000 | 4.635461000 | -1.559323000 |
| F | 4.562534000 | 2.791071000 | 0.033902000 |
| F | 3.261202000 | 0.683687000 | 0.903569000 |
| C | -1.332571000 | -0.143127000 | 1.419144000 |
| C | -2.314977000 | -1.090964000 | 1.720794000 |
| C | -1.644530000 | 1.176496000 | 1.081086000 |
| C | -3.635290000 | -0.734347000 | 1.508709000 |
| H | -2.038471000 | -2.096228000 | 2.037411000 |
| C | -2.994813000 | 1.479876000 | 0.833305000 |
| H | -0.891837000 | 1.962926000 | 1.084173000 |
| C | -4.004500000 | 0.541065000 | 1.022142000 |
| H | -4.402075000 | -1.486369000 | 1.698052000 |
| H | -3.237586000 | 2.501265000 | 0.531827000 |
| C | -5.457871000 | 0.915074000 | 0.723370000 |
| C | -5.569492000 | 1.311768000 | -0.756522000 |
| C | -6.417864000 | -0.246545000 | 0.984021000 |
| C | -5.875155000 | 2.097781000 | 1.609285000 |
| H | -4.929009000 | 2.172804000 | -0.997756000 |
| H | -5.268462000 | 0.478577000 | -1.410072000 |
| H | -6.607753000 | 1.584639000 | -1.003476000 |
| H | -6.406304000 | -0.561562000 | 2.038668000 |
| H | -7.446098000 | 0.062884000 | 0.743471000 |
| H | -6.181221000 | -1.121209000 | 0.358667000 |
| H | -6.920013000 | 2.379644000 | 1.403907000 |
| H | -5.793060000 | 1.838526000 | 2.675868000 |
| H | -5.249269000 | 2.984256000 | 1.430113000 |
| Br | 0.819157000 | -0.194776000 | 2.692862000 |

**int25**

Lowest frequency = 7.6460 $cm^{-1}$

Charge = 0, Multiplicity = 1

67

| | | | |
|---|---|---|---|
| O | 0.113390000 | -0.893597000 | 1.837786000 |
| C | 0.577620000 | -2.067488000 | 1.605366000 |
| O | 0.618829000 | -2.456133000 | 0.419425000 |
| C | -2.842741000 | -1.914220000 | -0.521829000 |
| N | -1.844175000 | -1.383500000 | -0.303981000 |
| C | -4.153296000 | -2.452045000 | -0.888719000 |
| C | -5.203576000 | -1.720101000 | -0.037757000 |
| C | -4.349939000 | -2.136516000 | -2.381318000 |
| C | -4.182920000 | -3.962841000 | -0.630622000 |
| H | -5.067763000 | -1.931213000 | 1.032946000 |
| H | -5.143574000 | -0.633819000 | -0.192192000 |
| H | -6.204973000 | -2.065432000 | -0.334769000 |
| H | -3.583426000 | -2.628648000 | -2.996230000 |
| H | -5.341164000 | -2.498902000 | -2.692708000 |
| H | -4.289027000 | -1.054787000 | -2.563967000 |
| H | -5.168560000 | -4.356199000 | -0.920191000 |

| | | | |
|---|---|---|---|
| H | -3.416187000 | -4.483625000 | -1.222009000 |
| H | -4.019606000 | -4.191136000 | 0.432665000 |
| C | 1.122911000 | -2.882775000 | 2.759908000 |
| C | 1.298719000 | -4.336206000 | 2.330487000 |
| C | 2.485081000 | -2.257380000 | 3.104201000 |
| C | 0.177132000 | -2.770717000 | 3.956974000 |
| H | 0.334094000 | -4.791585000 | 2.060685000 |
| H | 1.958717000 | -4.412944000 | 1.455444000 |
| H | 1.736736000 | -4.920176000 | 3.154297000 |
| H | 2.368509000 | -1.208077000 | 3.411786000 |
| H | 2.954180000 | -2.814663000 | 3.929835000 |
| H | 3.163322000 | -2.288596000 | 2.237351000 |
| H | 0.595988000 | -3.312074000 | 4.819155000 |
| H | 0.026511000 | -1.720067000 | 4.240726000 |
| H | -0.807515000 | -3.207176000 | 3.728079000 |
| Ru | -0.203625000 | -0.369172000 | -0.103523000 |
| C | -1.152063000 | 1.363386000 | 0.316670000 |
| C | -2.454501000 | 1.725687000 | -0.040898000 |
| C | -0.504971000 | 2.238323000 | 1.196532000 |
| C | -3.060678000 | 2.894208000 | 0.418802000 |
| C | -1.073509000 | 3.421001000 | 1.660047000 |
| C | -2.369934000 | 3.748300000 | 1.272399000 |
| F | 0.731950000 | 1.965872000 | 1.614921000 |
| F | -0.405614000 | 4.222592000 | 2.476429000 |
| F | -2.938466000 | 4.858445000 | 1.710615000 |
| F | -4.302375000 | 3.188835000 | 0.059053000 |
| F | -3.214910000 | 0.960992000 | -0.823827000 |
| C | 1.484582000 | -0.201340000 | -1.089678000 |
| C | 2.403970000 | -1.225657000 | -1.321942000 |
| C | 1.893065000 | 1.127826000 | -0.945653000 |
| C | 3.756915000 | -0.922240000 | -1.254257000 |
| H | 2.065018000 | -2.245336000 | -1.501956000 |
| C | 3.262378000 | 1.388648000 | -0.830867000 |
| H | 1.173353000 | 1.945846000 | -0.952716000 |
| C | 4.217156000 | 0.379925000 | -0.976598000 |
| H | 4.470749000 | -1.732225000 | -1.407759000 |
| H | 3.573682000 | 2.424011000 | -0.674869000 |
| C | 5.703963000 | 0.719074000 | -0.860035000 |
| C | 5.971912000 | 1.287813000 | 0.541535000 |
| C | 6.593986000 | -0.507997000 | -1.061972000 |
| C | 6.073165000 | 1.765208000 | -1.922067000 |
| H | 5.387512000 | 2.200275000 | 0.730337000 |
| H | 5.708658000 | 0.555166000 | 1.319925000 |
| H | 7.037941000 | 1.541262000 | 0.654820000 |
| H | 6.466438000 | -0.947386000 | -2.063083000 |
| H | 7.650828000 | -0.218633000 | -0.960411000 |
| H | 6.391490000 | -1.288917000 | -0.313020000 |
| H | 7.142383000 | 2.020268000 | -1.850963000 |
| H | 5.878164000 | 1.382396000 | -2.935412000 |
| H | 5.499443000 | 2.695353000 | -1.798724000 |
| Br | -0.726079000 | 0.112316000 | -2.597968000 |

**int26**

Lowest frequency = 13.1238 cm$^{-1}$

Charge = 0, Multiplicity = 1

82

| | | | |
|---|---|---|---|
| O | 1.490833000 | 1.338920000 | 1.721906000 |
| C | 0.779054000 | 2.382827000 | 1.587754000 |
| O | -0.039342000 | 2.441699000 | 0.635265000 |
| C | -1.469401000 | -1.066813000 | 1.663765000 |
| C | 3.096931000 | 1.739997000 | -1.499630000 |
| N | 2.158926000 | 1.272697000 | -1.012883000 |
| N | -0.652027000 | -0.458488000 | 1.119397000 |
| C | -2.482205000 | -1.972380000 | 2.214634000 |
| C | -3.386014000 | -2.397986000 | 1.046895000 |
| C | -1.747374000 | -3.191486000 | 2.793096000 |
| C | -3.294106000 | -1.246168000 | 3.292864000 |
| H | -3.888421000 | -1.530707000 | 0.595580000 |
| H | -2.798567000 | -2.899307000 | 0.265150000 |
| H | -4.151738000 | -3.096792000 | 1.417432000 |
| H | -1.083377000 | -2.900994000 | 3.620102000 |
| H | -2.484687000 | -3.913947000 | 3.175038000 |
| H | -1.142926000 | -3.681442000 | 2.016323000 |
| H | -4.052614000 | -1.930782000 | 3.701469000 |
| H | -2.651119000 | -0.910353000 | 4.119503000 |
| H | -3.810382000 | -0.370000000 | 2.873468000 |
| C | 4.393304000 | 2.218918000 | -1.991277000 |
| C | 5.248052000 | 0.978841000 | -2.300971000 |
| C | 4.193437000 | 3.077457000 | -3.245138000 |
| C | 5.035315000 | 3.038977000 | -0.861524000 |
| H | 5.343514000 | 0.340850000 | -1.411216000 |
| H | 4.800508000 | 0.382150000 | -3.109128000 |
| H | 6.251318000 | 1.299307000 | -2.620572000 |
| H | 3.570071000 | 3.957669000 | -3.030228000 |
| H | 5.172015000 | 3.427128000 | -3.607137000 |
| H | 3.714183000 | 2.501386000 | -4.050051000 |
| H | 6.028570000 | 3.386691000 | -1.183795000 |
| H | 4.423188000 | 3.917141000 | -0.609581000 |
| H | 5.152734000 | 2.426919000 | 0.044047000 |
| C | 0.990122000 | 3.549026000 | 2.540343000 |
| C | 2.282878000 | 4.244749000 | 2.087668000 |
| C | -0.189598000 | 4.512391000 | 2.452933000 |
| C | 1.158699000 | 3.015396000 | 3.963730000 |
| H | 3.132772000 | 3.547518000 | 2.125367000 |
| H | 2.186078000 | 4.618644000 | 1.056298000 |
| H | 2.503126000 | 5.101719000 | 2.743690000 |
| H | -1.124674000 | 4.021865000 | 2.762940000 |
| H | -0.021115000 | 5.378984000 | 3.111187000 |
| H | -0.329556000 | 4.871496000 | 1.424009000 |
| H | 1.369296000 | 3.844150000 | 4.657841000 |
| H | 0.243888000 | 2.507681000 | 4.306889000 |
| H | 1.984466000 | 2.292475000 | 4.013239000 |
| Ru | 0.732303000 | 0.409379000 | 0.032498000 |
| C | 1.768894000 | -1.343014000 | 0.063825000 |
| C | 3.142588000 | -1.368464000 | 0.336118000 |
| C | 1.200038000 | -2.616122000 | -0.060621000 |
| C | 3.898049000 | -2.534219000 | 0.441013000 |
| C | 1.911034000 | -3.808093000 | 0.066204000 |
| C | 3.278696000 | -3.773226000 | 0.312829000 |
| F | -0.114174000 | -2.772141000 | -0.304245000 |
| F | 1.286341000 | -4.978160000 | -0.038098000 |
| F | 3.977358000 | -4.895984000 | 0.424360000 |

| | | | |
|---|---|---|---|
| F | 5.205927000 | -2.470731000 | 0.673625000 |
| F | 3.831263000 | -0.232533000 | 0.526711000 |
| C | -2.303816000 | 0.071687000 | -1.724555000 |
| C | -3.259945000 | -0.725101000 | -2.336437000 |
| C | -2.652099000 | 1.072135000 | -0.823017000 |
| C | -4.605719000 | -0.519852000 | -2.027445000 |
| H | -2.965767000 | -1.510307000 | -3.035552000 |
| C | -3.999833000 | 1.248158000 | -0.528215000 |
| H | -1.885541000 | 1.683206000 | -0.337866000 |
| C | -5.004860000 | 0.460458000 | -1.112709000 |
| H | -5.346161000 | -1.158100000 | -2.510097000 |
| H | -4.266903000 | 2.029255000 | 0.187558000 |
| C | -6.466483000 | 0.685639000 | -0.723064000 |
| C | -6.864034000 | 2.130007000 | -1.059769000 |
| C | -7.411508000 | -0.261768000 | -1.462567000 |
| C | -6.623039000 | 0.445651000 | 0.786294000 |
| H | -6.244179000 | 2.861998000 | -0.521499000 |
| H | -6.754690000 | 2.325892000 | -2.137315000 |
| H | -7.914326000 | 2.312746000 | -0.782667000 |
| H | -7.193323000 | -1.316902000 | -1.237035000 |
| H | -8.449647000 | -0.065769000 | -1.154642000 |
| H | -7.358130000 | -0.123542000 | -2.553226000 |
| H | -7.668730000 | 0.603696000 | 1.094240000 |
| H | -6.341327000 | -0.585192000 | 1.051821000 |
| H | -5.994449000 | 1.129172000 | 1.376387000 |
| Br | -0.449078000 | -0.253501000 | -2.108005000 |

**TS27$^{ISET-OSS}$**

Lowest frequency = -27.4119 $cm^{-1}$

Charge = 0, Multiplicity = 1

82

| | | | |
|---|---|---|---|
| O | -0.093950000 | 2.088331000 | 2.351421000 |
| C | 0.133978000 | 3.294986000 | 2.014502000 |
| O | 0.979522000 | 3.502413000 | 1.111274000 |
| C | -0.760466000 | 1.137861000 | -1.336311000 |
| C | 3.411152000 | 1.381832000 | 3.315815000 |
| N | 2.666587000 | 1.397471000 | 2.437041000 |
| N | -0.045898000 | 1.222347000 | -0.436147000 |
| C | -1.643194000 | 0.976053000 | -2.495611000 |
| C | -1.046654000 | 1.771101000 | -3.665361000 |
| C | -1.696559000 | -0.521144000 | -2.835462000 |
| C | -3.035465000 | 1.504743000 | -2.125015000 |
| H | -0.975160000 | 2.841717000 | -3.424572000 |
| H | -0.044938000 | 1.401412000 | -3.925081000 |
| H | -1.699228000 | 1.655376000 | -4.544010000 |
| H | -2.096590000 | -1.107475000 | -1.995727000 |
| H | -2.352440000 | -0.665040000 | -3.707340000 |
| H | -0.696799000 | -0.901957000 | -3.084573000 |
| H | -3.707122000 | 1.382550000 | -2.987870000 |
| H | -3.460289000 | 0.950934000 | -1.275193000 |
| H | -3.000553000 | 2.572411000 | -1.863288000 |
| C | 4.272543000 | 1.260408000 | 4.495163000 |
| C | 3.637129000 | 2.084905000 | 5.624454000 |
| C | 4.307924000 | -0.230772000 | 4.869839000 |
| C | 5.675844000 | 1.777409000 | 4.157065000 |
| H | 3.580912000 | 3.150132000 | 5.356801000 |
| H | 2.621521000 | 1.727785000 | 5.847672000 |
| H | 4.250403000 | 1.987279000 | 6.532797000 |
| H | 4.766979000 | -0.827538000 | 4.068456000 |
| H | 4.904760000 | -0.359423000 | 5.785292000 |
| H | 3.293977000 | -0.615172000 | 5.050237000 |
| H | 6.324019000 | 1.666685000 | 5.039231000 |
| H | 6.119215000 | 1.209649000 | 3.326533000 |
| H | 5.652075000 | 2.840133000 | 3.875739000 |
| C | -0.586714000 | 4.416221000 | 2.738057000 |
| C | -0.079843000 | 4.405987000 | 4.187395000 |
| C | -0.278601000 | 5.751935000 | 2.069526000 |
| C | -2.089880000 | 4.122916000 | 2.711175000 |
| H | -0.276985000 | 3.434037000 | 4.662253000 |
| H | 1.004178000 | 4.596927000 | 4.227013000 |
| H | -0.585464000 | 5.191828000 | 4.769986000 |
| H | -0.620543000 | 5.761050000 | 1.024346000 |
| H | -0.784878000 | 6.567880000 | 2.608092000 |
| H | 0.801965000 | 5.952209000 | 2.062826000 |
| H | -2.636647000 | 4.899448000 | 3.268374000 |
| H | -2.472650000 | 4.112503000 | 1.678660000 |
| H | -2.305183000 | 3.145569000 | 3.165315000 |
| Ru | 1.302308000 | 1.300808000 | 1.013290000 |
| C | 1.014679000 | -0.648583000 | 1.481289000 |
| C | 0.937929000 | -1.082349000 | 2.810521000 |
| C | 0.865301000 | -1.654712000 | 0.521558000 |
| C | 0.788556000 | -2.420211000 | 3.168061000 |
| C | 0.719115000 | -3.002175000 | 0.842479000 |
| C | 0.674600000 | -3.391340000 | 2.177653000 |
| F | 0.888324000 | -1.373112000 | -0.778342000 |
| F | 0.611885000 | -3.913654000 | -0.115978000 |
| F | 0.530434000 | -4.665307000 | 2.501332000 |
| F | 0.753101000 | -2.773091000 | 4.447040000 |
| F | 1.011268000 | -0.224259000 | 3.829986000 |
| C | 2.621202000 | -0.118098000 | -3.260754000 |
| C | 2.673300000 | 0.912857000 | -4.169219000 |
| C | 2.052624000 | -1.346899000 | -3.520191000 |
| C | 2.110241000 | 0.693263000 | -5.434905000 |
| H | 3.123709000 | 1.876728000 | -3.916591000 |
| C | 1.492279000 | -1.537496000 | -4.786695000 |
| H | 2.015353000 | -2.137408000 | -2.767553000 |
| C | 1.502703000 | -0.525868000 | -5.761731000 |
| H | 2.143795000 | 1.503559000 | -6.165195000 |
| H | 1.029890000 | -2.503094000 | -5.011576000 |
| C | 0.842852000 | -0.782233000 | -7.119728000 |
| C | -0.648988000 | -1.078014000 | -6.904030000 |
| C | 0.961551000 | 0.421231000 | -8.055611000 |
| C | 1.513214000 | -1.986602000 | -7.795869000 |
| H | -0.801838000 | -1.960033000 | -6.264472000 |
| H | -1.149833000 | -0.223797000 | -6.422175000 |
| H | -1.149527000 | -1.269869000 | -7.866710000 |
| H | 2.012213000 | 0.674391000 | -8.263823000 |
| H | 0.477593000 | 0.193578000 | -9.017704000 |
| H | 0.469091000 | 1.313642000 | -7.639710000 |
| H | 1.048745000 | -2.187602000 | -8.774606000 |
| H | 2.585484000 | -1.797420000 | -7.956902000 |
| H | 1.422526000 | -2.899360000 | -7.188880000 |

| | | | |
|---|---|---|---|
| Br | 3.091598000 | 1.079074000 | -0.645955000 |

**TS27^ISET-Tri^**

Lowest frequency = -186.0493 cm$^{-1}$

Charge = 0, Multiplicity = 3

82

| | | | |
|---|---|---|---|
| O | 2.504438000 | 1.467427000 | 1.443153000 |
| C | 2.185769000 | 2.605285000 | 0.993106000 |
| O | 1.297259000 | 2.670934000 | 0.100270000 |
| C | -1.360741000 | 0.659763000 | 1.934014000 |
| C | 3.511776000 | 0.322890000 | -1.971767000 |
| N | 2.598017000 | 0.409955000 | -1.274555000 |
| N | -0.373821000 | 0.598630000 | 1.341568000 |
| C | -2.659132000 | 0.695569000 | 2.613964000 |
| C | -3.590311000 | 1.599478000 | 1.791645000 |
| C | -3.206068000 | -0.739240000 | 2.655081000 |
| C | -2.463762000 | 1.249795000 | 4.030915000 |
| H | -3.197932000 | 2.624869000 | 1.730759000 |
| H | -3.720453000 | 1.213922000 | 0.770634000 |
| H | -4.576109000 | 1.632360000 | 2.279658000 |
| H | -2.532623000 | -1.409993000 | 3.207451000 |
| H | -4.185434000 | -0.732369000 | 3.157010000 |
| H | -3.337098000 | -1.135227000 | 1.639100000 |
| H | -3.437337000 | 1.279722000 | 4.542560000 |
| H | -1.784191000 | 0.615353000 | 4.618323000 |
| H | -2.054872000 | 2.270435000 | 4.007430000 |
| C | 4.735261000 | 0.123025000 | -2.753537000 |
| C | 5.088822000 | -1.370448000 | -2.656770000 |
| C | 4.487526000 | 0.538102000 | -4.208568000 |
| C | 5.838977000 | 0.980413000 | -2.116083000 |
| H | 5.200412000 | -1.680563000 | -1.608092000 |
| H | 4.308271000 | -1.991288000 | -3.119849000 |
| H | 6.037018000 | -1.552545000 | -3.184592000 |
| H | 4.220929000 | 1.602541000 | -4.279655000 |
| H | 5.404493000 | 0.370988000 | -4.793242000 |
| H | 3.676936000 | -0.052886000 | -4.658585000 |
| H | 6.778356000 | 0.830339000 | -2.669110000 |
| H | 5.582716000 | 2.049313000 | -2.149556000 |
| H | 6.000832000 | 0.694829000 | -1.066769000 |
| C | 2.921959000 | 3.846984000 | 1.466491000 |
| C | 4.233926000 | 3.885443000 | 0.666345000 |
| C | 2.089150000 | 5.092508000 | 1.176199000 |
| C | 3.225558000 | 3.722324000 | 2.959398000 |
| H | 4.829789000 | 2.979128000 | 0.851290000 |
| H | 4.031554000 | 3.955116000 | -0.413819000 |
| H | 4.831687000 | 4.762166000 | 0.961185000 |
| H | 1.141041000 | 5.074242000 | 1.734523000 |
| H | 2.644496000 | 5.996113000 | 1.472163000 |
| H | 1.843125000 | 5.161856000 | 0.107742000 |
| H | 3.817042000 | 4.586611000 | 3.299546000 |
| H | 2.297605000 | 3.690596000 | 3.550933000 |
| H | 3.789273000 | 2.802635000 | 3.167844000 |
| Ru | 1.142768000 | 0.483985000 | 0.053324000 |
| C | 1.473717000 | -1.466668000 | 0.544330000 |
| C | 2.775217000 | -1.952301000 | 0.712548000 |
| C | 0.457221000 | -2.402514000 | 0.767604000 |
| C | 3.062727000 | -3.276611000 | 1.032960000 |
| C | 0.707713000 | -3.736293000 | 1.083905000 |
| C | 2.020095000 | -4.177928000 | 1.224207000 |
| F | -0.829820000 | -2.072021000 | 0.661872000 |
| F | -0.293897000 | -4.586473000 | 1.266544000 |
| F | 2.272651000 | -5.439100000 | 1.531606000 |
| F | 4.319787000 | -3.682777000 | 1.158504000 |
| F | 3.836775000 | -1.153999000 | 0.571284000 |
| C | -2.920254000 | 0.032471000 | -1.824856000 |
| C | -3.628067000 | 1.182639000 | -2.107677000 |
| C | -3.507711000 | -1.080970000 | -1.248465000 |
| C | -4.998435000 | 1.208397000 | -1.816111000 |
| H | -3.138711000 | 2.058860000 | -2.541786000 |
| C | -4.873635000 | -1.034753000 | -0.968597000 |
| H | -2.914112000 | -1.966680000 | -1.006098000 |
| C | -5.644618000 | 0.108554000 | -1.238422000 |
| H | -5.559206000 | 2.116711000 | -2.043127000 |
| H | -5.344989000 | -1.913138000 | -0.518148000 |
| C | -7.133060000 | 0.115631000 | -0.880175000 |
| C | -7.286964000 | -0.097347000 | 0.633249000 |
| C | -7.809626000 | 1.436182000 | -1.249566000 |
| C | -7.843801000 | -1.018088000 | -1.633246000 |
| H | -6.848073000 | -1.052594000 | 0.957817000 |
| H | -6.787801000 | 0.708790000 | 1.193058000 |
| H | -8.351526000 | -0.103095000 | 0.917334000 |
| H | -7.749795000 | 1.637072000 | -2.330043000 |
| H | -8.875502000 | 1.397101000 | -0.977466000 |
| H | -7.362118000 | 2.288791000 | -0.716100000 |
| H | -8.917362000 | -1.031975000 | -1.385629000 |
| H | -7.743989000 | -0.887220000 | -2.721554000 |
| H | -7.428206000 | -2.003414000 | -1.375737000 |
| Br | -0.569649000 | 0.049879000 | -1.718604000 |

**int28**

Lowest frequency = 12.3185 cm$^{-1}$

Charge = 0, Multiplicity = 2

59

| | | | |
|---|---|---|---|
| O | 0.740118000 | -1.305053000 | -1.413158000 |
| C | 1.085461000 | -2.428516000 | -0.928267000 |
| O | 0.876784000 | -2.628320000 | 0.294380000 |
| C | -2.852707000 | -1.492605000 | 0.015677000 |
| C | 2.981352000 | 0.528263000 | 0.946940000 |
| N | 1.922901000 | 0.099285000 | 0.797563000 |
| N | -1.736622000 | -1.224970000 | 0.115343000 |
| C | -4.304476000 | -1.688007000 | -0.033276000 |
| C | -4.814757000 | -1.618469000 | 1.414768000 |
| C | -4.892000000 | -0.537907000 | -0.867233000 |
| C | -4.622737000 | -3.047300000 | -0.666235000 |
| H | -4.384997000 | -2.424307000 | 2.026667000 |
| H | -4.548319000 | -0.657709000 | 1.877217000 |
| H | -5.910314000 | -1.722229000 | 1.415787000 |
| H | -4.528990000 | -0.573744000 | -1.904731000 |

| | | | |
|---|---|---|---|
| H | -5.988710000 | -0.626318000 | -0.880000000 |
| H | -4.620814000 | 0.435945000 | -0.436081000 |
| H | -5.713899000 | -3.184822000 | -0.696029000 |
| H | -4.239619000 | -3.108462000 | -1.695297000 |
| H | -4.187170000 | -3.870860000 | -0.082104000 |
| C | 4.305750000 | 1.148559000 | 1.037820000 |
| C | 4.145503000 | 2.610420000 | 0.588677000 |
| C | 4.802630000 | 1.068298000 | 2.486468000 |
| C | 5.247487000 | 0.391546000 | 0.089948000 |
| H | 3.719772000 | 2.667436000 | -0.423211000 |
| H | 3.483747000 | 3.161660000 | 1.272251000 |
| H | 5.131317000 | 3.099247000 | 0.588626000 |
| H | 4.907007000 | 0.024080000 | 2.814653000 |
| H | 5.786889000 | 1.554866000 | 2.558140000 |
| H | 4.111288000 | 1.578336000 | 3.172410000 |
| H | 6.244487000 | 0.855741000 | 0.124288000 |
| H | 5.346169000 | -0.663196000 | 0.385281000 |
| H | 4.880275000 | 0.430091000 | -0.945757000 |
| C | 1.782661000 | -3.443218000 | -1.813369000 |
| C | 3.192396000 | -2.889893000 | -2.072984000 |
| C | 1.858974000 | -4.791284000 | -1.103689000 |
| C | 1.017956000 | -3.558227000 | -3.134176000 |
| H | 3.140650000 | -1.905019000 | -2.559815000 |
| H | 3.752386000 | -2.782359000 | -1.130771000 |
| H | 3.752829000 | -3.576270000 | -2.726723000 |
| H | 0.854375000 | -5.189121000 | -0.897705000 |
| H | 2.396513000 | -5.517679000 | -1.732573000 |
| H | 2.381765000 | -4.703132000 | -0.141232000 |
| H | 1.539507000 | -4.250152000 | -3.813528000 |
| H | 0.000384000 | -3.945387000 | -2.970123000 |
| H | 0.934901000 | -2.578219000 | -3.624314000 |
| Ru | 0.097063000 | -0.560063000 | 0.441854000 |
| C | -0.448672000 | 1.256855000 | -0.274226000 |
| C | 0.384736000 | 1.959129000 | -1.153157000 |
| C | -1.681112000 | 1.865885000 | -0.008928000 |
| C | 0.036787000 | 3.183250000 | -1.720344000 |
| C | -2.061927000 | 3.085397000 | -0.563856000 |
| C | -1.198860000 | 3.751762000 | -1.428335000 |
| F | -2.571771000 | 1.312630000 | 0.812484000 |
| F | -3.248308000 | 3.608342000 | -0.283353000 |
| F | -1.547880000 | 4.909182000 | -1.963921000 |
| F | 0.873599000 | 3.806951000 | -2.539102000 |
| F | 1.578053000 | 1.482424000 | -1.511921000 |
| Br | -0.465466000 | -0.192522000 | 2.801199000 |

**int30**

Lowest frequency = 7.7052 $cm^{-1}$

Charge = 1, Multiplicity = 2

75

| | | | |
|---|---|---|---|
| O | 0.871633000 | 1.962610000 | 0.743013000 |
| C | 1.201126000 | 2.704918000 | -0.244404000 |
| O | 1.013963000 | 2.212437000 | -1.389610000 |
| C | -0.202537000 | -2.069437000 | -2.030073000 |
| O | -0.283056000 | -0.833781000 | -1.973671000 |
| C | -2.615130000 | 1.761627000 | -0.592360000 |
| C | 3.136785000 | -0.703443000 | -0.117130000 |
| N | 2.054014000 | -0.345896000 | -0.292155000 |
| N | -1.561475000 | 1.295299000 | -0.586595000 |
| C | -3.986313000 | 2.275425000 | -0.552135000 |
| C | -4.686147000 | 1.867483000 | -1.857329000 |
| C | -4.668473000 | 1.621552000 | 0.662261000 |
| C | -3.937825000 | 3.802598000 | -0.404708000 |
| H | -4.186572000 | 2.303547000 | -2.734155000 |
| H | -4.710663000 | 0.774280000 | -1.971560000 |
| H | -5.722418000 | 2.234903000 | -1.833034000 |
| H | -4.182083000 | 1.921670000 | 1.601654000 |
| H | -5.718313000 | 1.947061000 | 0.698891000 |
| H | -4.646567000 | 0.524449000 | 0.590005000 |
| H | -4.966680000 | 4.187399000 | -0.352310000 |
| H | -3.412443000 | 4.100892000 | 0.514032000 |
| H | -3.439041000 | 4.272405000 | -1.264588000 |
| C | 4.500186000 | -1.146269000 | 0.182235000 |
| C | 4.412019000 | -2.117332000 | 1.371530000 |
| C | 5.081258000 | -1.840082000 | -1.057735000 |
| C | 5.319165000 | 0.100660000 | 0.553405000 |
| H | 3.941579000 | -1.640308000 | 2.242839000 |
| H | 3.836946000 | -3.017482000 | 1.110039000 |
| H | 5.429000000 | -2.429478000 | 1.650200000 |
| H | 5.133153000 | -1.153983000 | -1.915270000 |
| H | 6.101643000 | -2.179672000 | -0.828046000 |
| H | 4.484861000 | -2.718826000 | -1.343108000 |
| H | 6.343866000 | -0.210888000 | 0.803086000 |
| H | 5.369950000 | 0.810605000 | -0.284672000 |
| H | 4.891095000 | 0.614327000 | 1.426092000 |
| C | 1.769753000 | 4.080505000 | -0.007144000 |
| C | 3.005424000 | 3.925478000 | 0.889861000 |
| C | 2.138136000 | 4.728404000 | -1.338323000 |
| C | 0.693343000 | 4.899502000 | 0.720512000 |
| H | 2.746627000 | 3.437812000 | 1.840334000 |
| H | 3.785215000 | 3.328219000 | 0.391832000 |
| H | 3.429919000 | 4.916145000 | 1.110101000 |
| H | 1.261089000 | 4.825245000 | -1.993701000 |
| H | 2.549387000 | 5.732703000 | -1.159657000 |
| H | 2.892931000 | 4.137688000 | -1.876934000 |
| H | 1.075156000 | 5.910092000 | 0.928337000 |
| H | -0.212833000 | 5.001548000 | 0.103139000 |
| H | 0.415925000 | 4.429551000 | 1.674789000 |
| C | -0.499382000 | -2.829254000 | -3.296703000 |
| C | -1.748303000 | -3.684309000 | -3.018224000 |
| C | -0.758591000 | -1.851591000 | -4.438594000 |
| C | 0.701035000 | -3.733699000 | -3.611875000 |
| H | -1.566915000 | -4.411374000 | -2.214452000 |
| H | -2.607619000 | -3.057520000 | -2.735939000 |
| H | -2.014655000 | -4.236320000 | -3.931159000 |
| H | 0.114697000 | -1.211347000 | -4.628600000 |
| H | -0.977829000 | -2.415017000 | -5.356487000 |
| H | -1.615138000 | -1.198285000 | -4.221740000 |
| H | 0.487654000 | -4.307456000 | -4.525169000 |
| H | 1.611967000 | -3.142404000 | -3.791860000 |
| H | 0.896402000 | -4.444568000 | -2.797173000 |
| O | 0.119618000 | -2.790266000 | -0.995345000 |
| H | 0.187349000 | -2.208900000 | -0.202301000 |

| | | | |
|---|---|---|---|
| Ru | 0.226311000 | 0.433628000 | -0.443193000 |
| C | -0.489611000 | -0.733906000 | 1.054835000 |
| C | 0.207526000 | -0.867621000 | 2.267930000 |
| C | -1.790002000 | -1.268815000 | 1.040736000 |
| C | -0.347613000 | -1.475410000 | 3.391616000 |
| C | -2.373871000 | -1.882298000 | 2.140856000 |
| C | -1.647863000 | -1.977020000 | 3.330429000 |
| F | -2.514489000 | -1.240440000 | -0.078565000 |
| F | -3.599320000 | -2.364454000 | 2.074776000 |
| F | -2.182336000 | -2.549749000 | 4.381333000 |
| F | 0.339548000 | -1.573695000 | 4.511062000 |
| F | 1.437271000 | -0.392345000 | 2.402100000 |

**int31**

Lowest frequency = 14.1985 $cm^{-1}$

Charge = 1, Multiplicity = 1

94

| | | | |
|---|---|---|---|
| O | 0.264815000 | 1.158873000 | 1.917678000 |
| C | 1.500301000 | 1.463634000 | 1.797386000 |
| O | 2.085298000 | 1.134699000 | 0.735249000 |
| C | -0.941368000 | 3.131564000 | -0.597981000 |
| C | 1.169654000 | -2.144322000 | 1.758512000 |
| N | 0.760177000 | -1.355316000 | 1.022514000 |
| N | -0.466436000 | 2.089771000 | -0.461812000 |
| C | -1.656448000 | 4.399350000 | -0.771256000 |
| C | -1.463622000 | 4.871521000 | -2.219158000 |
| C | -3.140394000 | 4.109915000 | -0.485402000 |
| C | -1.093633000 | 5.423498000 | 0.223462000 |
| H | -0.402702000 | 5.053424000 | -2.444294000 |
| H | -1.855861000 | 4.132872000 | -2.933055000 |
| H | -2.011437000 | 5.814071000 | -2.364383000 |
| H | -3.285833000 | 3.776353000 | 0.552141000 |
| H | -3.721075000 | 5.031535000 | -0.638102000 |
| H | -3.532718000 | 3.334032000 | -1.158419000 |
| H | -1.649013000 | 6.367104000 | 0.119537000 |
| H | -1.199181000 | 5.072855000 | 1.260148000 |
| H | -0.030498000 | 5.627320000 | 0.029652000 |
| C | 1.626951000 | -3.131759000 | 2.739904000 |
| C | 1.285645000 | -4.531745000 | 2.209256000 |
| C | 3.142379000 | -2.971069000 | 2.924793000 |
| C | 0.876861000 | -2.847422000 | 4.051357000 |
| H | 0.200893000 | -4.651256000 | 2.070078000 |
| H | 1.794820000 | -4.734770000 | 1.255031000 |
| H | 1.618544000 | -5.285357000 | 2.937823000 |
| H | 3.393764000 | -1.962928000 | 3.284031000 |
| H | 3.494167000 | -3.700020000 | 3.669481000 |
| H | 3.683078000 | -3.148701000 | 1.983831000 |
| H | 1.188290000 | -3.581923000 | 4.808617000 |
| H | 1.105604000 | -1.840105000 | 4.426846000 |
| H | -0.210696000 | -2.921305000 | 3.912190000 |
| C | 2.189315000 | 2.203658000 | 2.925399000 |
| C | 2.068410000 | 1.345270000 | 4.190815000 |
| C | 3.653586000 | 2.449496000 | 2.576696000 |
| C | 1.447064000 | 3.532016000 | 3.124347000 |
| H | 1.015203000 | 1.134799000 | 4.424879000 |
| H | 2.597183000 | 0.386367000 | 4.068506000 |
| H | 2.520238000 | 1.869013000 | 5.046605000 |
| H | 3.750974000 | 3.043190000 | 1.656497000 |
| H | 4.147330000 | 2.995372000 | 3.394450000 |
| H | 4.191515000 | 1.503148000 | 2.420511000 |
| H | 1.901716000 | 4.096829000 | 3.952157000 |
| H | 1.503663000 | 4.155935000 | 2.218531000 |
| H | 0.387822000 | 3.357072000 | 3.360456000 |
| Ru | 0.145507000 | 0.263789000 | 0.059035000 |
| C | -1.858979000 | -0.165236000 | 0.163384000 |
| C | -2.409269000 | -0.747459000 | 1.312380000 |
| C | -2.815590000 | 0.308568000 | -0.741052000 |
| C | -3.779775000 | -0.872563000 | 1.540492000 |
| C | -4.192325000 | 0.218344000 | -0.551523000 |
| C | -4.685493000 | -0.373570000 | 0.607244000 |
| F | -2.441302000 | 0.881073000 | -1.900626000 |
| F | -5.026551000 | 0.712119000 | -1.454456000 |
| F | -5.984836000 | -0.470112000 | 0.813521000 |
| F | -4.225733000 | -1.442762000 | 2.647640000 |
| F | -1.624540000 | -1.234839000 | 2.278269000 |
| S | -1.105507000 | -1.842148000 | -2.158994000 |
| C | 0.443584000 | -1.089318000 | -1.702149000 |
| C | 0.604810000 | 0.265327000 | -2.114054000 |
| C | 1.617962000 | -1.920886000 | -1.607268000 |
| C | 1.924867000 | 0.768861000 | -2.341682000 |
| H | -0.240738000 | 0.797416000 | -2.551403000 |
| C | 2.852090000 | -1.374602000 | -1.782250000 |
| H | 1.522355000 | -2.977503000 | -1.350617000 |
| C | 3.043101000 | 0.004729000 | -2.135974000 |
| H | 2.010686000 | 1.805918000 | -2.664208000 |
| H | 3.726820000 | -2.016028000 | -1.657658000 |
| C | 4.461966000 | 0.545173000 | -2.249128000 |
| C | 5.231370000 | -0.239272000 | -3.321446000 |
| C | 4.475789000 | 2.028403000 | -2.615087000 |
| C | 5.141816000 | 0.375556000 | -0.879782000 |
| H | 5.284007000 | -1.314712000 | -3.094240000 |
| H | 4.761851000 | -0.123774000 | -4.310319000 |
| H | 6.265447000 | 0.131281000 | -3.390783000 |
| H | 3.956660000 | 2.634362000 | -1.856969000 |
| H | 5.514214000 | 2.385972000 | -2.672578000 |
| H | 4.009477000 | 2.215012000 | -3.594724000 |
| H | 6.158745000 | 0.796114000 | -0.910031000 |
| H | 4.564272000 | 0.889644000 | -0.097675000 |
| H | 5.232133000 | -0.683668000 | -0.594085000 |
| C | -0.681113000 | -2.997284000 | -3.526505000 |
| H | 0.220952000 | -2.608947000 | -4.019336000 |
| H | -1.526446000 | -2.930194000 | -4.227232000 |
| C | -0.543566000 | -4.383049000 | -2.912570000 |
| H | 0.478485000 | -4.528941000 | -2.530665000 |
| H | -0.716543000 | -5.160011000 | -3.671669000 |
| C | -1.555153000 | -4.457225000 | -1.774102000 |
| H | -1.402305000 | -5.338652000 | -1.133826000 |
| H | -2.577471000 | -4.528654000 | -2.178745000 |
| C | -1.411374000 | -3.183852000 | -0.952912000 |
| H | -2.305372000 | -2.894827000 | -0.386179000 |
| H | -0.554178000 | -3.191691000 | -0.266785000 |

**TS32**

Lowest frequency = -37.2395 $cm^{-1}$

Charge = 1, Multiplicity = 1

94

| | | | |
|---|---|---|---|
| O | 1.499558000 | 2.305344000 | -0.761828000 |
| C | 2.021540000 | 1.926864000 | -1.851956000 |
| O | 1.655161000 | 0.789628000 | -2.286104000 |
| C | -1.991213000 | 2.046551000 | -1.777809000 |
| C | 2.984568000 | -1.012275000 | 0.547987000 |
| N | 2.030310000 | -0.502116000 | 0.153817000 |
| N | -1.081186000 | 1.469876000 | -1.369409000 |
| C | -3.170064000 | 2.776035000 | -2.251437000 |
| C | -3.842668000 | 1.938674000 | -3.349861000 |
| C | -4.114973000 | 2.955804000 | -1.052566000 |
| C | -2.704770000 | 4.135218000 | -2.794301000 |
| H | -3.171867000 | 1.789822000 | -4.208449000 |
| H | -4.151667000 | 0.957037000 | -2.961823000 |
| H | -4.737266000 | 2.472485000 | -3.702541000 |
| H | -3.631355000 | 3.509643000 | -0.235687000 |
| H | -4.998150000 | 3.522106000 | -1.382504000 |
| H | -4.449075000 | 1.983512000 | -0.665507000 |
| H | -3.583055000 | 4.695957000 | -3.145872000 |
| H | -2.206082000 | 4.729013000 | -2.014842000 |
| H | -2.013587000 | 4.013866000 | -3.640817000 |
| C | 4.213451000 | -1.580363000 | 1.107781000 |
| C | 3.822145000 | -2.510106000 | 2.266036000 |
| C | 4.949773000 | -2.351150000 | 0.003685000 |
| C | 5.061661000 | -0.402794000 | 1.616900000 |
| H | 3.285572000 | -1.960954000 | 3.052955000 |
| H | 3.189340000 | -3.338721000 | 1.918462000 |
| H | 4.736301000 | -2.934997000 | 2.705561000 |
| H | 5.211873000 | -1.693121000 | -0.837197000 |
| H | 5.879648000 | -2.767090000 | 0.417937000 |
| H | 4.341950000 | -3.183070000 | -0.380124000 |
| H | 5.983003000 | -0.799314000 | 2.067976000 |
| H | 5.340553000 | 0.271300000 | 0.794319000 |
| H | 4.519944000 | 0.181013000 | 2.373638000 |
| C | 3.084381000 | 2.744498000 | -2.539963000 |
| C | 2.775017000 | 4.231767000 | -2.363217000 |
| C | 4.398681000 | 2.388866000 | -1.820423000 |
| C | 3.164395000 | 2.363461000 | -4.016449000 |
| H | 1.830689000 | 4.503420000 | -2.859068000 |
| H | 2.691533000 | 4.496798000 | -1.300320000 |
| H | 3.578683000 | 4.834140000 | -2.811721000 |
| H | 4.632496000 | 1.318709000 | -1.933149000 |
| H | 5.226984000 | 2.966396000 | -2.257434000 |
| H | 4.337224000 | 2.627002000 | -0.747981000 |
| H | 3.977543000 | 2.924365000 | -4.500335000 |
| H | 3.359356000 | 1.289559000 | -4.143001000 |
| H | 2.227690000 | 2.601888000 | -4.542097000 |
| Ru | 0.450207000 | 0.473320000 | -0.566015000 |
| C | 0.018870000 | 1.163469000 | 1.329067000 |
| C | 1.054497000 | 1.601346000 | 2.162770000 |
| C | -1.266471000 | 1.396693000 | 1.818346000 |
| C | 0.829130000 | 2.177417000 | 3.413324000 |
| C | -1.529505000 | 1.959787000 | 3.064932000 |
| C | -0.471179000 | 2.363825000 | 3.871955000 |
| F | -2.342217000 | 1.057537000 | 1.106024000 |
| F | -2.778785000 | 2.121685000 | 3.469416000 |
| F | -0.695070000 | 2.906550000 | 5.050299000 |
| F | 1.851288000 | 2.560117000 | 4.157714000 |
| F | 2.334962000 | 1.509452000 | 1.808570000 |
| S | -3.643895000 | -0.975675000 | -0.620136000 |
| C | -0.702119000 | -1.009634000 | -0.063182000 |
| C | -0.451082000 | -1.402875000 | -1.385743000 |
| C | -0.724305000 | -1.911033000 | 1.006982000 |
| C | 0.042205000 | -2.726665000 | -1.593418000 |
| H | -0.831347000 | -0.859993000 | -2.255314000 |
| C | -0.297082000 | -3.194495000 | 0.747189000 |
| H | -0.962957000 | -1.580294000 | 2.019996000 |
| C | 0.119330000 | -3.628331000 | -0.551711000 |
| H | 0.244360000 | -3.039457000 | -2.618114000 |
| H | -0.233120000 | -3.895088000 | 1.583703000 |
| C | 0.549150000 | -5.081289000 | -0.738582000 |
| C | -0.629402000 | -5.994912000 | -0.366445000 |
| C | 0.960654000 | -5.374117000 | -2.181084000 |
| C | 1.744552000 | -5.379645000 | 0.177927000 |
| H | -0.942712000 | -5.864230000 | 0.680288000 |
| H | -1.500887000 | -5.794594000 | -1.008588000 |
| H | -0.347505000 | -7.050656000 | -0.497702000 |
| H | 1.798296000 | -4.737744000 | -2.505699000 |
| H | 1.287160000 | -6.420587000 | -2.267783000 |
| H | 0.125724000 | -5.233980000 | -2.884044000 |
| H | 2.052635000 | -6.430250000 | 0.067929000 |
| H | 2.606655000 | -4.747518000 | -0.084340000 |
| H | 1.509596000 | -5.217121000 | 1.240563000 |
| C | -3.740278000 | -2.801157000 | -0.700883000 |
| H | -2.740158000 | -3.198361000 | -0.926517000 |
| H | -4.420401000 | -3.085176000 | -1.518174000 |
| C | -4.265995000 | -3.252989000 | 0.655187000 |
| H | -3.437549000 | -3.272892000 | 1.382488000 |
| H | -4.681363000 | -4.271002000 | 0.600046000 |
| C | -5.303600000 | -2.224404000 | 1.084659000 |
| H | -5.628881000 | -2.366699000 | 2.126599000 |
| H | -6.198124000 | -2.308229000 | 0.446424000 |
| C | -4.661222000 | -0.856924000 | 0.897715000 |
| H | -5.395583000 | -0.048440000 | 0.774354000 |
| H | -4.000564000 | -0.586624000 | 1.734264000 |

**int33**

Lowest frequency = 15.0759 $cm^{-1}$

Charge = 1, Multiplicity = 1

81

| | | | |
|---|---|---|---|
| O | -1.490345000 | -1.648336000 | 0.817396000 |
| C | -0.935782000 | -2.020008000 | 1.895607000 |
| O | 0.008623000 | -1.278099000 | 2.313026000 |
| C | -2.599872000 | 1.591660000 | 1.687172000 |
| C | 2.144478000 | -1.846340000 | -0.567261000 |
| N | 1.312430000 | -1.177190000 | -0.135665000 |
| N | -1.637487000 | 1.024416000 | 1.402754000 |

| | | | |
|---|---|---|---|
| C | -3.838342000 | 2.333112000 | 1.936240000 |
| C | -3.466281000 | 3.796707000 | 2.218040000 |
| C | -4.681456000 | 2.222075000 | 0.653108000 |
| C | -4.565407000 | 1.703593000 | 3.132156000 |
| H | -2.845427000 | 3.887841000 | 3.120916000 |
| H | -2.926838000 | 4.240112000 | 1.368929000 |
| H | -4.390148000 | 4.371323000 | 2.377947000 |
| H | -4.948638000 | 1.176865000 | 0.440970000 |
| H | -5.610043000 | 2.795068000 | 0.790616000 |
| H | -4.144819000 | 2.631609000 | -0.214331000 |
| H | -5.504507000 | 2.249523000 | 3.303285000 |
| H | -4.811837000 | 0.649073000 | 2.941665000 |
| H | -3.961135000 | 1.765237000 | 4.048639000 |
| C | 3.159483000 | -2.715842000 | -1.165697000 |
| C | 3.874694000 | -1.914133000 | -2.263934000 |
| C | 4.145151000 | -3.145851000 | -0.070219000 |
| C | 2.425258000 | -3.929210000 | -1.759370000 |
| H | 3.171575000 | -1.590839000 | -3.044936000 |
| H | 4.375952000 | -1.027817000 | -1.848830000 |
| H | 4.637065000 | -2.554081000 | -2.731479000 |
| H | 3.636068000 | -3.695383000 | 0.734523000 |
| H | 4.902579000 | -3.808654000 | -0.513287000 |
| H | 4.660503000 | -2.278953000 | 0.368207000 |
| H | 3.160357000 | -4.585684000 | -2.247332000 |
| H | 1.912387000 | -4.505880000 | -0.976207000 |
| H | 1.679880000 | -3.619763000 | -2.505324000 |
| C | -1.317012000 | -3.307064000 | 2.575543000 |
| C | -2.825654000 | -3.522356000 | 2.442476000 |
| C | -0.555616000 | -4.407680000 | 1.812743000 |
| C | -0.884257000 | -3.272142000 | 4.039425000 |
| H | -3.387937000 | -2.735841000 | 2.968571000 |
| H | -3.137040000 | -3.521656000 | 1.388795000 |
| H | -3.101945000 | -4.489846000 | 2.886550000 |
| H | 0.532895000 | -4.264034000 | 1.895608000 |
| H | -0.803052000 | -5.390293000 | 2.241235000 |
| H | -0.832028000 | -4.413143000 | 0.747850000 |
| H | -1.114548000 | -4.236549000 | 4.515419000 |
| H | 0.194626000 | -3.087043000 | 4.134591000 |
| H | -1.413866000 | -2.483921000 | 4.594920000 |
| Ru | -0.135181000 | -0.044589000 | 0.618259000 |
| C | -1.035868000 | 0.147967000 | -1.224957000 |
| C | -1.180940000 | -0.973134000 | -2.045930000 |
| C | -1.697808000 | 1.302526000 | -1.647018000 |
| C | -1.958022000 | -0.953408000 | -3.205189000 |
| C | -2.488321000 | 1.350175000 | -2.792049000 |
| C | -2.624167000 | 0.209914000 | -3.579431000 |
| F | -1.586857000 | 2.447266000 | -0.965706000 |
| F | -3.113309000 | 2.466858000 | -3.121255000 |
| F | -3.366562000 | 0.233862000 | -4.665403000 |
| F | -2.069949000 | -2.041722000 | -3.943321000 |
| F | -0.601628000 | -2.135260000 | -1.758496000 |
| C | 0.801141000 | 1.495878000 | -0.072889000 |
| C | 1.211204000 | 1.757301000 | 1.239878000 |
| C | 1.610921000 | 1.744770000 | -1.180143000 |
| C | 2.588633000 | 2.030980000 | 1.448096000 |
| H | 0.524581000 | 1.905414000 | 2.077573000 |
| C | 2.929428000 | 2.075378000 | -0.933810000 |
| H | 1.244409000 | 1.577729000 | -2.195153000 |
| C | 3.460904000 | 2.180891000 | 0.381985000 |
| H | 2.921776000 | 2.221613000 | 2.468605000 |
| H | 3.596711000 | 2.208078000 | -1.789014000 |
| C | 4.946692000 | 2.481035000 | 0.560508000 |
| C | 5.287107000 | 3.807507000 | -0.135090000 |
| C | 5.335854000 | 2.585353000 | 2.034865000 |
| C | 5.750939000 | 1.336873000 | -0.077990000 |
| H | 5.078682000 | 3.778621000 | -1.214911000 |
| H | 4.712554000 | 4.639767000 | 0.298541000 |
| H | 6.357203000 | 4.033043000 | -0.012539000 |
| H | 5.126956000 | 1.653744000 | 2.582993000 |
| H | 6.414571000 | 2.781284000 | 2.118720000 |
| H | 4.813964000 | 3.412183000 | 2.539981000 |
| H | 6.829396000 | 1.520681000 | 0.041011000 |
| H | 5.516723000 | 0.374034000 | 0.402964000 |
| H | 5.551798000 | 1.241137000 | -1.156216000 |

**int34**

Lowest frequency = 15.5971 cm$^{-1}$

Charge = 1, Multiplicity = 1

103

| | | | |
|---|---|---|---|
| O | -2.732554000 | -0.040186000 | 1.192916000 |
| C | -3.063298000 | 1.179603000 | 1.001596000 |
| O | -2.285131000 | 1.901836000 | 0.330031000 |
| C | -2.914717000 | -1.097886000 | -2.066028000 |
| C | 0.179910000 | 1.009290000 | 2.850570000 |
| N | -0.151744000 | 0.660049000 | 1.802822000 |
| N | -2.177109000 | -0.562814000 | -1.358941000 |
| C | -3.793731000 | -1.884488000 | -2.936673000 |
| C | -3.494323000 | -1.514420000 | -4.395899000 |
| C | -3.466344000 | -3.364241000 | -2.671163000 |
| C | -5.252355000 | -1.570860000 | -2.577046000 |
| H | -3.695160000 | -0.450675000 | -4.589401000 |
| H | -2.446996000 | -1.729177000 | -4.653354000 |
| H | -4.139477000 | -2.111135000 | -5.057237000 |
| H | -3.684266000 | -3.636565000 | -1.628473000 |
| H | -4.083157000 | -3.992852000 | -3.330246000 |
| H | -2.407168000 | -3.581443000 | -2.871807000 |
| H | -5.916622000 | -2.183756000 | -3.203822000 |
| H | -5.461773000 | -1.801195000 | -1.522640000 |
| H | -5.489782000 | -0.512255000 | -2.756620000 |
| C | 0.589324000 | 1.424604000 | 4.194624000 |
| C | 1.963107000 | 2.101551000 | 4.086853000 |
| C | -0.462687000 | 2.405609000 | 4.732221000 |
| C | 0.661387000 | 0.168083000 | 5.074810000 |
| H | 2.714476000 | 1.413205000 | 3.673708000 |
| H | 1.916821000 | 2.997398000 | 3.450339000 |
| H | 2.293022000 | 2.410730000 | 5.089608000 |
| H | -1.449028000 | 1.925871000 | 4.805404000 |
| H | -0.164925000 | 2.737453000 | 5.737768000 |
| H | -0.551288000 | 3.291157000 | 4.086410000 |
| H | 0.947752000 | 0.460094000 | 6.095953000 |
| H | -0.309147000 | -0.345663000 | 5.115629000 |
| H | 1.408698000 | -0.540288000 | 4.691448000 |
| C | -4.375890000 | 1.682540000 | 1.567406000 |

| | | | |
|---|---|---|---|
| C | -4.372285000 | 1.426383000 | 3.078890000 |
| C | -4.542054000 | 3.169093000 | 1.269694000 |
| C | -5.495685000 | 0.868490000 | 0.904061000 |
| H | -4.211810000 | 0.361205000 | 3.297053000 |
| H | -3.578274000 | 2.006618000 | 3.574603000 |
| H | -5.334688000 | 1.733435000 | 3.515418000 |
| H | -4.531532000 | 3.363520000 | 0.187657000 |
| H | -5.499129000 | 3.528428000 | 1.676541000 |
| H | -3.732134000 | 3.759610000 | 1.722118000 |
| H | -6.474292000 | 1.187035000 | 1.293980000 |
| H | -5.500919000 | 1.019099000 | -0.187030000 |
| H | -5.371076000 | -0.204850000 | 1.106493000 |
| Ru | -0.987450000 | 0.100909000 | 0.097960000 |
| C | -0.462887000 | -1.867041000 | 0.287044000 |
| C | -0.595699000 | -2.522905000 | 1.518409000 |
| C | -0.196248000 | -2.728904000 | -0.780048000 |
| C | -0.452413000 | -3.901273000 | 1.682468000 |
| C | -0.053714000 | -4.108966000 | -0.669874000 |
| C | -0.189544000 | -4.709053000 | 0.578120000 |
| F | -0.032948000 | -2.241878000 | -2.030714000 |
| F | 0.185601000 | -4.846837000 | -1.744681000 |
| F | -0.063762000 | -6.015621000 | 0.715207000 |
| F | -0.578306000 | -4.450398000 | 2.879953000 |
| F | -0.858378000 | -1.839117000 | 2.634701000 |
| S | 2.139662000 | -0.681648000 | -0.727765000 |
| C | 3.109322000 | -0.639673000 | 0.750544000 |
| C | 3.314439000 | -0.293260000 | -1.994256000 |
| C | 1.033884000 | 0.737218000 | -0.666615000 |
| C | 2.738508000 | -1.546281000 | 1.747893000 |
| C | 3.065328000 | -0.901782000 | -3.228582000 |
| C | 4.382480000 | 0.593513000 | -1.820490000 |
| C | 0.094325000 | 0.797072000 | -1.732129000 |
| C | 1.492660000 | 1.966659000 | -0.076647000 |
| H | 1.886924000 | -2.207507000 | 1.577957000 |
| C | 4.922162000 | 0.151682000 | 2.120249000 |
| H | 2.239882000 | -1.612768000 | -3.317379000 |
| C | 5.177309000 | 0.902625000 | -2.930374000 |
| C | -0.397489000 | 2.073207000 | -2.149484000 |
| H | -0.003876000 | -0.044545000 | -2.417597000 |
| C | 0.941817000 | 3.147559000 | -0.469745000 |
| H | 2.237349000 | 1.941903000 | 0.717886000 |
| C | 4.530745000 | -0.723066000 | 3.127925000 |
| H | 5.799728000 | 0.788357000 | 2.254226000 |
| C | 4.906239000 | 0.330851000 | -4.169056000 |
| H | 6.024634000 | 1.581767000 | -2.810758000 |
| C | -0.033754000 | 3.234309000 | -1.519240000 |
| H | -1.102916000 | 2.085598000 | -2.979493000 |
| H | 1.268515000 | 4.062104000 | 0.028830000 |
| H | 5.099409000 | -0.752131000 | 4.059958000 |
| H | 5.538114000 | 0.584703000 | -5.022806000 |
| C | -0.618715000 | 4.596003000 | -1.868639000 |
| C | 0.507229000 | 5.536609000 | -2.321730000 |
| C | -1.659856000 | 4.498009000 | -2.982470000 |
| C | -1.302912000 | 5.158716000 | -0.611543000 |
| H | 1.275560000 | 5.668760000 | -1.545191000 |
| H | 1.002874000 | 5.153347000 | -3.226819000 |
| H | 0.098216000 | 6.532183000 | -2.552499000 |
| H | -2.500754000 | 3.850443000 | -2.690799000 |
| H | -2.067843000 | 5.496520000 | -3.197388000 |
| H | -1.226667000 | 4.111611000 | -3.917916000 |
| H | -1.779612000 | 6.123755000 | -0.842811000 |
| H | -2.067405000 | 4.459420000 | -0.243218000 |
| H | -0.584544000 | 5.333318000 | 0.203684000 |
| C | 3.860864000 | -0.581639000 | -4.321666000 |
| H | 3.668991000 | -1.046608000 | -5.290179000 |
| C | 3.446210000 | -1.582478000 | 2.942496000 |
| H | 3.158312000 | -2.291431000 | 3.720936000 |
| C | 4.221092000 | 0.202764000 | 0.907517000 |
| S | 4.807709000 | 1.348154000 | -0.291189000 |

**TS35**

Lowest frequency = -20.6760 $cm^{-1}$

Charge = 1, Multiplicity = 1

103

| | | | |
|---|---|---|---|
| O | -2.020710000 | -1.641508000 | 1.442964000 |
| C | -3.172939000 | -1.150248000 | 1.225351000 |
| O | -3.260413000 | -0.257478000 | 0.334964000 |
| C | -1.702530000 | -2.962192000 | -1.695196000 |
| C | -0.849794000 | 1.478460000 | 2.499563000 |
| N | -0.930591000 | 0.870867000 | 1.525101000 |
| N | -1.532371000 | -1.929752000 | -1.212584000 |
| C | -1.804641000 | -4.309741000 | -2.260162000 |
| C | -0.664432000 | -4.471013000 | -3.278727000 |
| C | -1.635181000 | -5.294704000 | -1.091429000 |
| C | -3.174955000 | -4.478463000 | -2.928350000 |
| H | -0.777707000 | -3.771092000 | -4.118979000 |
| H | 0.317931000 | -4.300078000 | -2.816190000 |
| H | -0.688619000 | -5.495482000 | -3.678037000 |
| H | -2.424761000 | -5.161600000 | -0.337982000 |
| H | -1.694241000 | -6.321806000 | -1.480306000 |
| H | -0.660024000 | -5.164220000 | -0.600654000 |
| H | -3.245625000 | -5.493434000 | -3.345643000 |
| H | -3.992386000 | -4.347365000 | -2.204536000 |
| H | -3.313640000 | -3.759892000 | -3.748845000 |
| C | -0.702008000 | 2.214079000 | 3.757047000 |
| C | 0.422823000 | 3.243693000 | 3.572567000 |
| C | -2.034277000 | 2.905702000 | 4.077934000 |
| C | -0.332955000 | 1.190862000 | 4.843053000 |
| H | 1.375412000 | 2.748519000 | 3.335623000 |
| H | 0.186905000 | 3.960264000 | 2.771921000 |
| H | 0.549031000 | 3.805547000 | 4.509547000 |
| H | -2.846683000 | 2.173328000 | 4.190154000 |
| H | -1.933542000 | 3.454432000 | 5.025687000 |
| H | -2.315122000 | 3.621753000 | 3.292073000 |
| H | -0.198251000 | 1.719412000 | 5.798139000 |
| H | -1.127270000 | 0.441249000 | 4.968344000 |
| H | 0.598794000 | 0.665132000 | 4.593465000 |
| C | -4.365862000 | -1.631027000 | 2.018797000 |
| C | -4.077575000 | -1.336264000 | 3.497390000 |
| C | -5.630573000 | -0.913589000 | 1.557772000 |
| C | -4.484866000 | -3.145362000 | 1.800997000 |
| H | -3.150015000 | -1.827879000 | 3.823747000 |
| H | -3.978936000 | -0.252858000 | 3.671377000 |

| | | | |
|---|---|---|---|
| H | -4.906822000 | -1.702352000 | 4.121002000 |
| H | -5.832539000 | -1.102487000 | 0.493716000 |
| H | -6.493504000 | -1.266875000 | 2.141578000 |
| H | -5.544357000 | 0.174226000 | 1.691389000 |
| H | -5.326390000 | -3.543212000 | 2.387579000 |
| H | -4.673628000 | -3.378431000 | 0.740921000 |
| H | -3.565924000 | -3.661189000 | 2.113937000 |
| Ru | -1.130965000 | -0.376522000 | -0.007831000 |
| C | 0.658539000 | -1.330078000 | 0.256493000 |
| C | 1.143625000 | -1.616916000 | 1.533784000 |
| C | 1.333982000 | -1.959105000 | -0.787799000 |
| C | 2.218112000 | -2.476894000 | 1.759622000 |
| C | 2.372895000 | -2.863563000 | -0.598886000 |
| C | 2.832161000 | -3.116897000 | 0.688369000 |
| F | 1.016826000 | -1.706475000 | -2.067954000 |
| F | 2.939756000 | -3.456444000 | -1.640729000 |
| F | 3.857636000 | -3.923136000 | 0.886667000 |
| F | 2.660743000 | -2.684264000 | 2.988968000 |
| F | 0.591127000 | -1.088032000 | 2.624515000 |
| S | 2.295163000 | 1.112095000 | -1.823146000 |
| C | 3.589820000 | 0.161501000 | -1.081651000 |
| C | 2.659988000 | 2.703472000 | -1.152369000 |
| C | -0.219309000 | 1.088888000 | -0.968748000 |
| C | 4.321390000 | -0.723245000 | -1.876510000 |
| C | 2.560271000 | 3.828921000 | -1.972459000 |
| C | 2.949455000 | 2.845441000 | 0.214144000 |
| C | -1.016105000 | 0.612929000 | -2.023223000 |
| C | -0.240786000 | 2.451636000 | -0.581970000 |
| H | 4.094117000 | -0.806474000 | -2.940980000 |
| C | 4.893764000 | -0.480344000 | 0.850835000 |
| H | 2.345505000 | 3.701202000 | -3.035529000 |
| C | 3.135762000 | 4.123486000 | 0.748007000 |
| C | -2.068321000 | 1.457835000 | -2.499307000 |
| H | -0.691823000 | -0.224014000 | -2.639628000 |
| C | -1.242570000 | 3.232108000 | -1.095161000 |
| H | 0.442419000 | 2.841828000 | 0.170763000 |
| C | 5.597139000 | -1.389056000 | 0.063314000 |
| H | 5.127877000 | -0.365618000 | 1.911293000 |
| C | 3.005391000 | 5.245129000 | -0.068407000 |
| H | 3.390540000 | 4.231926000 | 1.804615000 |
| C | -2.213881000 | 2.742269000 | -2.029546000 |
| H | -2.705442000 | 1.077197000 | -3.297722000 |
| H | -1.323650000 | 4.260562000 | -0.735631000 |
| H | 6.377625000 | -2.002902000 | 0.516706000 |
| H | 3.148304000 | 6.240582000 | 0.357470000 |
| C | -3.343807000 | 3.667903000 | -2.465293000 |
| C | -2.757752000 | 4.950151000 | -3.073732000 |
| C | -4.256699000 | 3.007517000 | -3.497519000 |
| C | -4.179595000 | 4.010790000 | -1.220896000 |
| H | -2.126105000 | 5.500969000 | -2.360946000 |
| H | -2.148485000 | 4.723132000 | -3.961845000 |
| H | -3.569684000 | 5.625974000 | -3.382437000 |
| H | -4.723493000 | 2.092284000 | -3.102802000 |
| H | -5.066535000 | 3.699341000 | -3.771468000 |
| H | -3.714878000 | 2.753482000 | -4.421370000 |
| H | -5.018705000 | 4.667251000 | -1.498062000 |
| H | -4.591574000 | 3.098682000 | -0.762493000 |
| H | -3.586178000 | 4.536288000 | -0.457407000 |
| C | 2.720661000 | 5.099644000 | -1.426250000 |
| H | 2.634626000 | 5.979303000 | -2.067258000 |
| C | 5.316769000 | -1.505712000 | -1.297652000 |
| H | 5.875260000 | -2.211615000 | -1.914926000 |
| C | 3.882248000 | 0.295834000 | 0.282117000 |
| S | 2.985415000 | 1.440418000 | 1.285987000 |

**int36**

Lowest frequency = 14.8435 $cm^{-1}$

Charge = 1, Multiplicity = 1

81

| | | | |
|---|---|---|---|
| O | 1.552150000 | 1.555425000 | 0.886938000 |
| C | 1.038086000 | 1.891000000 | 1.996932000 |
| O | 0.059847000 | 1.179067000 | 2.387138000 |
| C | 2.521837000 | -1.763031000 | 1.581057000 |
| C | -2.043348000 | 2.016159000 | -0.477248000 |
| N | -1.272778000 | 1.269879000 | -0.058079000 |
| N | 1.580656000 | -1.142882000 | 1.339270000 |
| C | 3.731429000 | -2.565767000 | 1.775989000 |
| C | 3.302469000 | -4.023013000 | 2.004548000 |
| C | 4.556604000 | -2.436400000 | 0.482933000 |
| C | 4.504721000 | -2.017417000 | 2.982882000 |
| H | 2.693537000 | -4.125727000 | 2.914273000 |
| H | 2.730524000 | -4.407864000 | 1.148179000 |
| H | 4.203681000 | -4.641805000 | 2.123692000 |
| H | 4.864161000 | -1.395241000 | 0.308969000 |
| H | 5.462295000 | -3.052782000 | 0.579381000 |
| H | 3.988446000 | -2.786536000 | -0.390428000 |
| H | 5.422905000 | -2.608241000 | 3.113676000 |
| H | 4.791863000 | -0.966858000 | 2.831306000 |
| H | 3.914205000 | -2.092496000 | 3.907298000 |
| C | -2.960706000 | 2.990980000 | -1.071530000 |
| C | -4.402788000 | 2.520930000 | -0.837392000 |
| C | -2.704610000 | 4.347479000 | -0.396766000 |
| C | -2.634812000 | 3.056991000 | -2.573007000 |
| H | -4.582478000 | 1.535945000 | -1.291550000 |
| H | -4.635926000 | 2.459974000 | 0.235456000 |
| H | -5.092077000 | 3.243243000 | -1.298279000 |
| H | -1.666477000 | 4.677094000 | -0.546216000 |
| H | -3.372838000 | 5.097950000 | -0.843616000 |
| H | -2.909430000 | 4.303510000 | 0.682655000 |
| H | -3.276672000 | 3.815792000 | -3.043819000 |
| H | -1.584016000 | 3.332252000 | -2.740382000 |
| H | -2.825591000 | 2.091696000 | -3.063956000 |
| C | 1.514883000 | 3.103461000 | 2.749861000 |
| C | 0.889189000 | 4.309560000 | 2.025375000 |
| C | 1.037663000 | 3.039994000 | 4.198570000 |
| C | 3.041554000 | 3.174769000 | 2.666798000 |
| H | 1.199817000 | 4.340960000 | 0.970599000 |
| H | -0.210165000 | 4.270371000 | 2.068794000 |
| H | 1.215704000 | 5.240547000 | 2.512179000 |
| H | 1.468533000 | 2.175649000 | 4.725267000 |
| H | 1.348660000 | 3.951665000 | 4.729413000 |
| H | -0.056667000 | 2.960605000 | 4.257863000 |
| H | 3.396962000 | 4.087805000 | 3.166456000 |

| | | | |
|---|---|---|---|
| H | 3.508095000 | 2.312251000 | 3.167022000 |
| H | 3.382956000 | 3.194418000 | 1.622586000 |
| Ru | 0.118669000 | 0.030606000 | 0.629967000 |
| C | 0.990690000 | -0.112389000 | -1.232779000 |
| C | 1.182403000 | 1.038196000 | -2.001914000 |
| C | 1.589442000 | -1.276044000 | -1.719989000 |
| C | 1.937553000 | 1.035406000 | -3.175590000 |
| C | 2.358892000 | -1.307787000 | -2.879899000 |
| C | 2.539372000 | -0.139678000 | -3.615775000 |
| F | 1.432891000 | -2.445342000 | -1.091815000 |
| F | 2.922518000 | -2.436820000 | -3.271555000 |
| F | 3.262263000 | -0.148529000 | -4.714991000 |
| F | 2.090587000 | 2.151352000 | -3.863536000 |
| F | 0.670041000 | 2.214763000 | -1.650533000 |
| C | -0.893399000 | -1.431249000 | -0.124769000 |
| C | -1.290738000 | -1.767476000 | 1.174297000 |
| C | -1.725519000 | -1.594930000 | -1.231818000 |
| C | -2.667077000 | -2.039807000 | 1.386227000 |
| H | -0.595508000 | -1.967498000 | 1.993915000 |
| C | -3.043039000 | -1.931090000 | -0.988076000 |
| H | -1.371168000 | -1.369620000 | -2.239889000 |
| C | -3.555392000 | -2.121041000 | 0.325648000 |
| H | -2.987240000 | -2.288828000 | 2.398236000 |
| H | -3.723629000 | -2.005733000 | -1.839790000 |
| C | -5.037253000 | -2.438799000 | 0.506349000 |
| C | -5.379786000 | -3.721177000 | -0.266891000 |
| C | -5.403953000 | -2.639403000 | 1.976530000 |
| C | -5.857487000 | -1.262101000 | -0.046203000 |
| H | -5.188706000 | -3.622119000 | -1.345780000 |
| H | -4.793287000 | -4.575224000 | 0.103934000 |
| H | -6.446421000 | -3.961679000 | -0.142548000 |
| H | -5.191392000 | -1.743543000 | 2.579995000 |
| H | -6.480497000 | -2.845863000 | 2.062995000 |
| H | -4.871206000 | -3.494148000 | 2.420199000 |
| H | -6.932876000 | -1.464430000 | 0.070931000 |
| H | -5.626355000 | -0.331498000 | 0.494697000 |
| H | -5.670282000 | -1.092328000 | -1.117430000 |

**Dibenzothiophenium salt S9**

Lowest frequency = 16.0758 cm$^{-1}$

Charge = 1, Multiplicity = 1

68

| | | | |
|---|---|---|---|
| C | 2.998138000 | 2.854326000 | 1.038485000 |
| C | 3.534922000 | 1.940759000 | 0.118246000 |
| C | 2.634267000 | 1.196925000 | -0.662756000 |
| C | 1.278680000 | 1.385895000 | -0.474776000 |
| C | 0.729386000 | 2.293689000 | 0.443465000 |
| C | 1.625578000 | 3.038907000 | 1.205423000 |
| H | 3.672466000 | 3.451097000 | 1.652267000 |
| H | 2.997097000 | 0.476993000 | -1.398792000 |
| H | 1.259588000 | 3.766215000 | 1.933038000 |
| C | -1.280568000 | 1.384473000 | -0.474819000 |
| C | -2.635948000 | 1.194013000 | -0.662842000 |
| C | -3.537450000 | 1.936852000 | 0.118122000 |
| C | -3.001700000 | 2.851003000 | 1.038388000 |
| C | -1.629351000 | 3.037099000 | 1.205371000 |
| C | -0.732307000 | 2.292879000 | 0.443439000 |
| H | -2.997949000 | 0.473682000 | -1.398896000 |
| H | -3.676707000 | 3.447018000 | 1.652158000 |
| H | -1.264189000 | 3.764803000 | 1.933006000 |
| C | 0.000449000 | -1.124036000 | -0.775585000 |
| C | -0.000958000 | -1.399167000 | 0.590193000 |
| C | 0.002807000 | -2.144008000 | -1.727240000 |
| C | 0.000058000 | -2.725958000 | 1.001257000 |
| H | -0.002834000 | -0.590351000 | 1.324702000 |
| C | 0.003805000 | -3.462640000 | -1.288321000 |
| H | 0.003810000 | -1.914350000 | -2.795451000 |
| C | 0.002470000 | -3.784746000 | 0.077871000 |
| H | -0.001068000 | -2.935415000 | 2.070609000 |
| H | 0.005628000 | -4.258525000 | -2.035312000 |
| C | 0.003611000 | -5.249035000 | 0.509699000 |
| C | 1.262515000 | -5.929472000 | -0.050576000 |
| C | 0.001909000 | -5.400998000 | 2.031031000 |
| C | -1.252353000 | -5.932334000 | -0.053683000 |
| H | 1.295916000 | -5.900196000 | -1.149631000 |
| H | 2.176551000 | -5.449393000 | 0.330734000 |
| H | 1.282601000 | -6.987112000 | 0.252649000 |
| H | -0.892956000 | -4.953466000 | 2.489977000 |
| H | 0.002834000 | -6.468956000 | 2.292571000 |
| H | 0.894586000 | -4.951370000 | 2.492185000 |
| H | -1.270756000 | -6.990033000 | 0.249446000 |
| H | -2.168418000 | -5.454370000 | 0.325406000 |
| H | -1.283127000 | -5.903073000 | -1.152816000 |
| S | -0.000492000 | 0.543767000 | -1.388921000 |
| C | 5.036954000 | 1.730067000 | -0.067570000 |
| C | -5.039245000 | 1.724564000 | -0.067795000 |
| C | 5.373885000 | 0.263529000 | 0.243043000 |
| H | 4.848568000 | -0.433863000 | -0.426994000 |
| H | 6.453373000 | 0.090438000 | 0.117259000 |
| H | 5.106483000 | 0.005840000 | 1.279097000 |
| C | 5.858335000 | 2.627245000 | 0.858529000 |
| H | 6.929460000 | 2.442652000 | 0.692457000 |
| H | 5.676889000 | 3.695417000 | 0.665165000 |
| H | 5.652033000 | 2.423448000 | 1.920301000 |
| C | 5.410595000 | 2.052849000 | -1.522361000 |
| H | 4.890148000 | 1.400740000 | -2.239947000 |
| H | 5.166514000 | 3.096217000 | -1.772915000 |
| H | 6.491284000 | 1.910573000 | -1.673631000 |
| C | -5.413141000 | 2.047236000 | -1.522550000 |
| H | -4.891924000 | 1.395839000 | -2.240221000 |
| H | -6.493665000 | 1.903820000 | -1.673923000 |
| H | -5.170169000 | 3.090919000 | -1.772869000 |
| C | -5.374641000 | 0.257614000 | 0.242510000 |
| H | -6.453944000 | 0.083419000 | 0.116666000 |
| H | -4.848597000 | -0.439106000 | -0.427657000 |
| H | -5.107001000 | -0.000012000 | 1.278519000 |
| C | -5.861635000 | 2.620677000 | 0.858440000 |
| H | -5.655133000 | 2.416912000 | 1.920181000 |
| H | -5.681366000 | 3.689084000 | 0.665278000 |
| H | -6.932552000 | 2.434942000 | 0.692301000 |

**PivOH**
Lowest frequency = 43.2569 cm$^{-1}$
Charge = 0, Multiplicity = 1

17

| | | | |
|---|---|---|---|
| C | 0.963879000 | -0.799927000 | 1.255402000 |
| C | 0.571268000 | -0.010252000 | -0.000006000 |
| H | 0.678922000 | -0.259430000 | 2.171313000 |
| H | 0.481292000 | -1.787230000 | 1.272851000 |
| H | 2.054888000 | -0.946234000 | 1.275514000 |
| C | 0.963755000 | -0.800406000 | -1.255144000 |
| C | 1.240103000 | 1.360029000 | -0.000294000 |
| H | 0.678619000 | -0.260311000 | -2.171238000 |
| H | 2.054773000 | -0.946629000 | -1.275361000 |
| H | 0.481246000 | -1.787755000 | -1.272123000 |
| H | 2.334433000 | 1.243421000 | -0.000255000 |
| H | 0.950378000 | 1.942682000 | -0.886303000 |
| H | 0.950342000 | 1.943067000 | 0.885448000 |
| C | -0.938687000 | 0.182024000 | 0.000076000 |
| O | -1.513260000 | 1.239142000 | -0.000004000 |
| O | -1.601974000 | -0.985371000 | 0.000000000 |
| H | -2.544924000 | -0.760556000 | -0.000011000 |

**Pentafluorobenzen**
Lowest frequency = 133.9300 cm$^{-1}$
Charge = 0, Multiplicity = 1

12

| | | | |
|---|---|---|---|
| C | 0.000028000 | -1.670536000 | -0.000028000 |
| C | -1.197104000 | -0.967296000 | -0.000055000 |
| C | -1.210774000 | 0.426459000 | -0.000030000 |
| C | -0.000002000 | 1.118364000 | -0.000015000 |
| C | 1.210749000 | 0.426517000 | -0.000034000 |
| C | 1.197106000 | -0.967283000 | 0.000011000 |
| H | 0.000000000 | -2.760633000 | 0.000006000 |
| F | -2.351322000 | -1.613515000 | 0.000044000 |
| F | -2.350662000 | 1.093082000 | 0.000002000 |
| F | -0.000082000 | 2.436675000 | 0.000030000 |
| F | 2.350680000 | 1.093068000 | 0.000002000 |
| F | 2.351384000 | -1.613389000 | 0.000022000 |

***t*BuCN**
Lowest frequency = 183.9196 cm$^{-1}$
Charge = 0, Multiplicity = 1

15

| | | | |
|---|---|---|---|
| N | 2.349620000 | 0.000059000 | 0.000174000 |
| C | 1.191970000 | 0.000068000 | 0.000172000 |
| C | -0.280201000 | 0.000003000 | -0.000003000 |
| C | -0.768225000 | 1.289918000 | -0.673278000 |
| C | -0.768040000 | -1.228028000 | -0.780579000 |
| C | -0.768523000 | -0.061982000 | 1.453630000 |
| H | -0.413916000 | 2.179178000 | -0.132058000 |
| H | -0.413465000 | 1.354633000 | -1.712119000 |
| H | -1.868860000 | 1.305250000 | -0.681604000 |
| H | -0.413397000 | -2.160039000 | -0.317095000 |
| H | -1.868671000 | -1.242875000 | -0.789923000 |
| H | -0.413493000 | -1.203976000 | -1.821229000 |
| H | -1.869158000 | -0.062730000 | 1.470750000 |
| H | -0.414097000 | -0.975198000 | 1.953283000 |
| H | -0.414169000 | 0.805465000 | 2.029126000 |

***tert*-Butylbenzyl radical**
Lowest frequency = 49.3249 cm$^{-1}$
Charge = 0, Multiplicity = 2

23

| | | | |
|---|---|---|---|
| C | 2.891528000 | -0.006619000 | 0.000001000 |
| C | 2.270933000 | 1.218370000 | -0.000010000 |
| C | 2.239553000 | -1.219408000 | 0.000010000 |
| C | 0.868108000 | 1.221535000 | -0.000003000 |
| H | 2.828062000 | 2.159086000 | -0.000031000 |
| C | 0.842167000 | -1.183329000 | 0.000025000 |
| H | 2.773521000 | -2.173399000 | -0.000004000 |
| C | 0.133296000 | 0.029530000 | 0.000045000 |
| H | 0.353824000 | 2.183760000 | -0.000029000 |
| H | 0.296887000 | -2.131169000 | 0.000003000 |
| C | -1.398218000 | 0.005755000 | -0.000001000 |
| C | -1.893066000 | -0.727658000 | 1.255182000 |
| C | -1.996997000 | 1.412935000 | -0.000750000 |
| C | -1.892946000 | -0.728953000 | -1.254467000 |
| H | -1.530863000 | -1.765688000 | 1.292235000 |
| H | -1.547381000 | -0.219510000 | 2.168412000 |
| H | -2.994228000 | -0.756840000 | 1.274385000 |
| H | -1.699103000 | 1.984315000 | -0.893180000 |
| H | -3.095696000 | 1.348204000 | -0.000753000 |
| H | -1.699154000 | 1.985242000 | 0.891105000 |
| H | -2.994105000 | -0.758245000 | -1.273696000 |
| H | -1.547256000 | -0.221698000 | -2.168191000 |
| H | -1.530649000 | -1.766992000 | -1.290455000 |

***tert*-Butylbenzyl bromide**
Lowest frequency = 47.8315 cm$^{-1}$
Charge = 0, Multiplicity = 1

24

| | | | |
|---|---|---|---|
| C | 1.367126000 | 0.010128000 | -0.000002000 |
| C | 0.688072000 | 1.221779000 | -0.000001000 |
| C | 0.669484000 | -1.195436000 | -0.000002000 |
| C | -0.707065000 | 1.221561000 | -0.000002000 |
| H | 1.240900000 | 2.162699000 | 0.000000000 |
| C | -0.720548000 | -1.170984000 | -0.000004000 |
| H | 1.209489000 | -2.143761000 | -0.000001000 |
| C | -1.442858000 | 0.032131000 | -0.000006000 |

| | | | |
|---|---|---|---|
| H | -1.218483000 | 2.184470000 | -0.000001000 |
| H | -1.252454000 | -2.125333000 | -0.000002000 |
| C | -2.972057000 | 0.001474000 | 0.000000000 |
| C | -3.461489000 | -0.735808000 | -1.255186000 |
| C | -3.461471000 | -0.735834000 | 1.255178000 |
| C | -3.574505000 | 1.406756000 | 0.000018000 |
| H | -3.118395000 | -0.226849000 | -2.168888000 |
| H | -3.094583000 | -1.772220000 | -1.290785000 |
| H | -4.562326000 | -0.770253000 | -1.274343000 |
| H | -3.118375000 | -0.226886000 | 2.168885000 |
| H | -4.562307000 | -0.770292000 | 1.274344000 |
| H | -3.094551000 | -1.772242000 | 1.290756000 |
| H | -4.672887000 | 1.339187000 | 0.000012000 |
| H | -3.278361000 | 1.979297000 | 0.892281000 |
| H | -3.278353000 | 1.979322000 | -0.892227000 |
| Br | 3.271216000 | -0.004907000 | 0.000001000 |

**Phenyl(tetrahydro)thiophenium triflate S1**

Lowest frequency = 23.7900 cm$^{-1}$

Charge = 1, Multiplicity = 1

36

| | | | |
|---|---|---|---|
| C | -0.726123000 | -0.533510000 | -0.025322000 |
| C | -0.278469000 | 0.787052000 | 0.046581000 |
| C | 0.180466000 | -1.595733000 | -0.084036000 |
| C | 1.088622000 | 1.035378000 | 0.059993000 |
| H | -0.976432000 | 1.625673000 | 0.092132000 |
| C | 1.541396000 | -1.320844000 | -0.066399000 |
| H | -0.173500000 | -2.627686000 | -0.142395000 |
| C | 2.028883000 | -0.006213000 | 0.005490000 |
| H | 1.424901000 | 2.070409000 | 0.115326000 |
| H | 2.239759000 | -2.158434000 | -0.110532000 |
| C | 3.533582000 | 0.245042000 | 0.022167000 |
| C | 4.143324000 | -0.326423000 | -1.267917000 |
| C | 3.867559000 | 1.734635000 | 0.104704000 |
| C | 4.138780000 | -0.464577000 | 1.243973000 |
| H | 3.982099000 | -1.410629000 | -1.359363000 |
| H | 3.716557000 | 0.158324000 | -2.159046000 |
| H | 5.229785000 | -0.152707000 | -1.274951000 |
| H | 3.477090000 | 2.195834000 | 1.024855000 |
| H | 4.959141000 | 1.864771000 | 0.114119000 |
| H | 3.480834000 | 2.294001000 | -0.760963000 |
| H | 5.225131000 | -0.292663000 | 1.274115000 |
| H | 3.708425000 | -0.080639000 | 2.181306000 |
| H | 3.977688000 | -1.552279000 | 1.215200000 |
| S | -2.435665000 | -0.976474000 | -0.063568000 |
| C | -3.230661000 | -0.080674000 | 1.334490000 |
| H | -2.453653000 | 0.563154000 | 1.770497000 |
| H | -3.533443000 | -0.833839000 | 2.074326000 |
| C | -4.377908000 | 0.699903000 | 0.709565000 |
| H | -4.700461000 | 1.499787000 | 1.392476000 |
| H | -5.246254000 | 0.039865000 | 0.553709000 |
| C | -3.875400000 | 1.241470000 | -0.623897000 |
| H | -3.146785000 | 2.051207000 | -0.462358000 |
| H | -4.689527000 | 1.659618000 | -1.233977000 |
| C | -3.222519000 | 0.076532000 | -1.353617000 |
| H | -3.956207000 | -0.583125000 | -1.840193000 |
| H | -2.443694000 | 0.340706000 | -2.081843000 |

**Arylthianthrenium salt S2**

Lowest frequency = 22.8679 cm$^{-1}$

Charge = 1, Multiplicity = 1

45

| | | | |
|---|---|---|---|
| C | -2.958892000 | 3.556952000 | 0.910193000 |
| C | -3.706315000 | 2.460269000 | 0.487868000 |
| C | -3.065202000 | 1.360675000 | -0.086989000 |
| C | -1.671932000 | 1.387617000 | -0.206127000 |
| C | -0.911006000 | 2.470387000 | 0.224879000 |
| C | -1.569929000 | 3.562567000 | 0.784667000 |
| C | -1.672331000 | -1.387597000 | -0.206154000 |
| C | -3.065592000 | -1.360199000 | -0.086977000 |
| C | -3.707051000 | -2.459612000 | 0.487844000 |
| H | -4.792510000 | -2.445032000 | 0.606262000 |
| C | -2.959983000 | -3.556574000 | 0.910073000 |
| C | -1.571030000 | -3.562668000 | 0.784457000 |
| C | -0.911761000 | -2.470677000 | 0.224707000 |
| H | -3.469222000 | 4.414942000 | 1.352581000 |
| H | -4.791783000 | 2.446044000 | 0.606255000 |
| H | 0.174452000 | 2.471774000 | 0.112842000 |
| H | -0.990439000 | 4.424719000 | 1.119963000 |
| H | -3.470585000 | -4.414413000 | 1.352438000 |
| H | -0.991821000 | -4.425050000 | 1.119647000 |
| H | 0.173688000 | -2.472445000 | 0.112591000 |
| C | 0.754456000 | -0.000248000 | -0.625950000 |
| C | 1.171341000 | -0.000589000 | 0.707376000 |
| C | 1.677449000 | 0.000133000 | -1.674143000 |
| C | 2.532978000 | -0.000579000 | 0.981672000 |
| H | 0.444095000 | -0.000842000 | 1.522558000 |
| C | 3.032891000 | 0.000151000 | -1.371459000 |
| H | 1.341389000 | 0.000406000 | -2.713675000 |
| C | 3.492805000 | -0.000219000 | -0.044583000 |
| H | 2.849827000 | -0.000850000 | 2.024299000 |
| H | 3.748484000 | 0.000461000 | -2.195484000 |
| C | 4.992572000 | -0.000130000 | 0.236338000 |
| C | 5.612816000 | -1.257630000 | -0.393421000 |
| C | 5.612500000 | 1.257830000 | -0.392819000 |
| C | 5.298293000 | -0.000456000 | 1.734277000 |
| H | 5.176326000 | -2.173308000 | 0.033577000 |
| H | 5.472260000 | -1.289369000 | -1.483848000 |
| H | 6.695730000 | -1.276139000 | -0.199443000 |
| H | 5.175796000 | 2.173192000 | 0.034638000 |
| H | 6.695414000 | 1.276511000 | -0.198860000 |
| H | 5.471896000 | 1.290061000 | -1.483226000 |
| H | 6.387239000 | -0.000411000 | 1.885369000 |
| H | 4.899665000 | 0.893380000 | 2.238139000 |
| H | 4.899790000 | -0.894583000 | 2.237722000 |
| S | -4.020877000 | 0.000377000 | -0.697492000 |
| S | -0.950508000 | -0.000093000 | -1.07742700 |

## 11. NMR Spectra

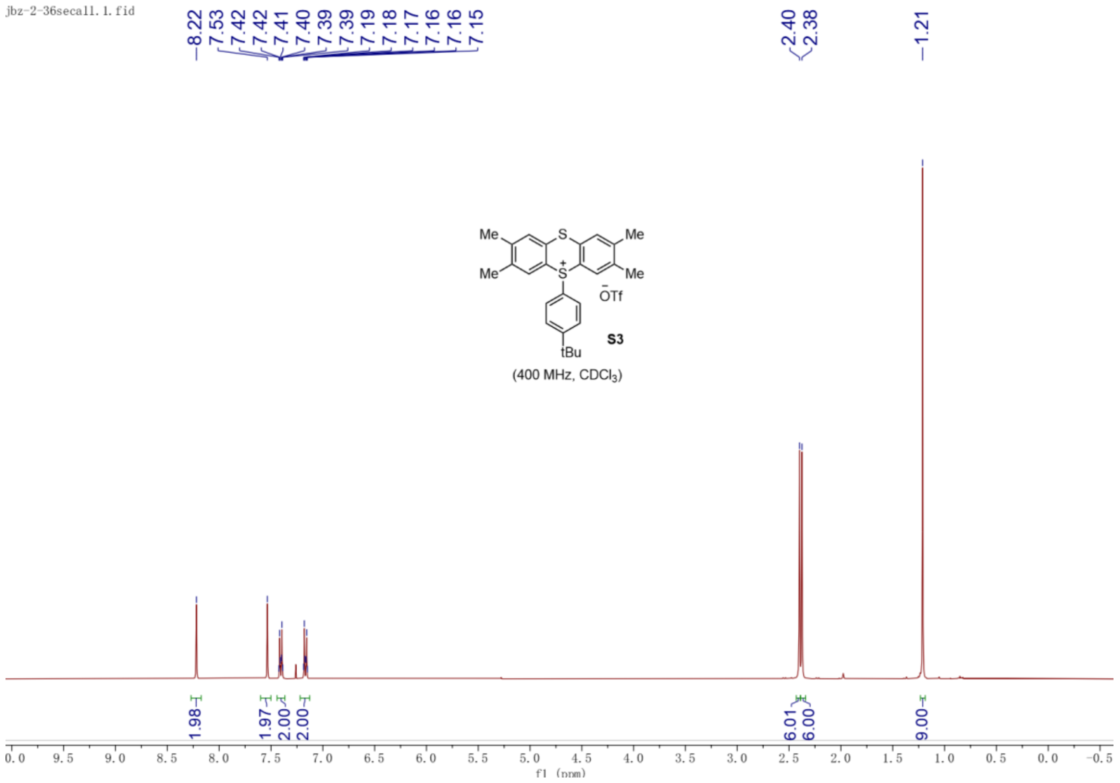


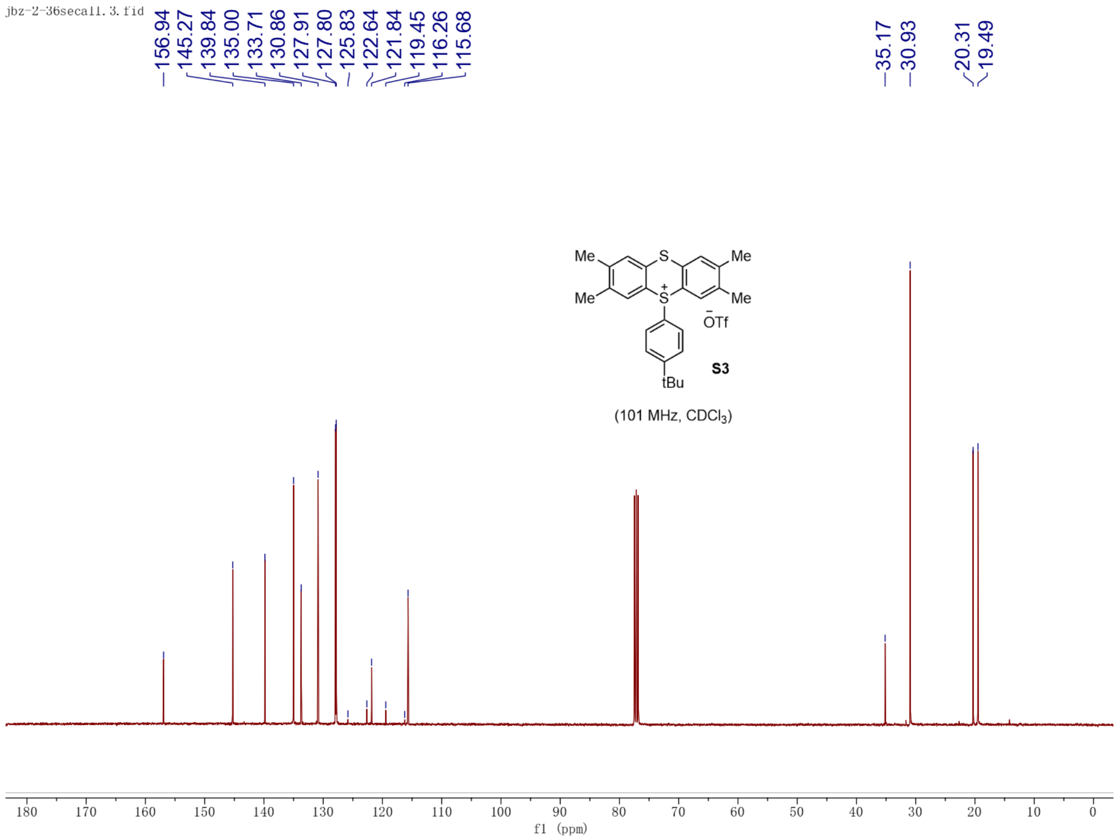

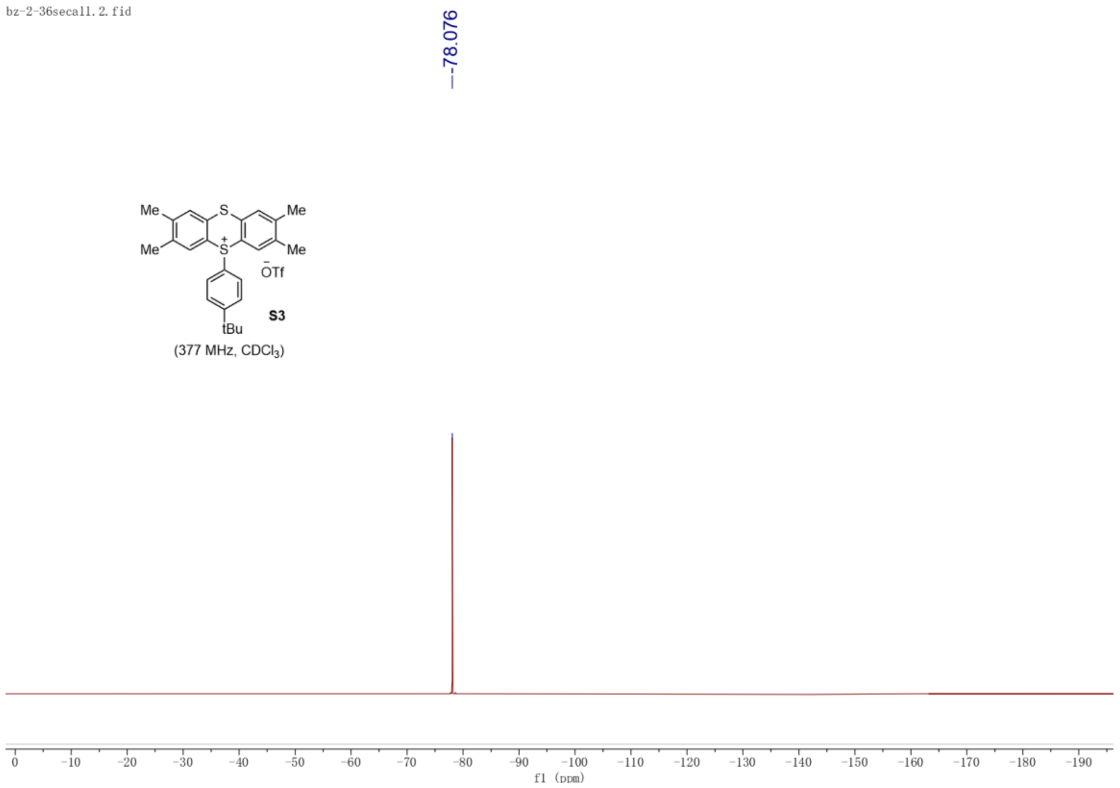
bz-2-36secal1.2.fid
-78.076
Me
Me
S
S
Me
Me
OTf
tBu
S3
(377 MHz, CDCl3)
0 −10 −20 −30 −40 −50 −60 −70 −80 −90 −100 −110 −120 −130 −140 −150 −160 −170 −180 −190
f1 (ppm)

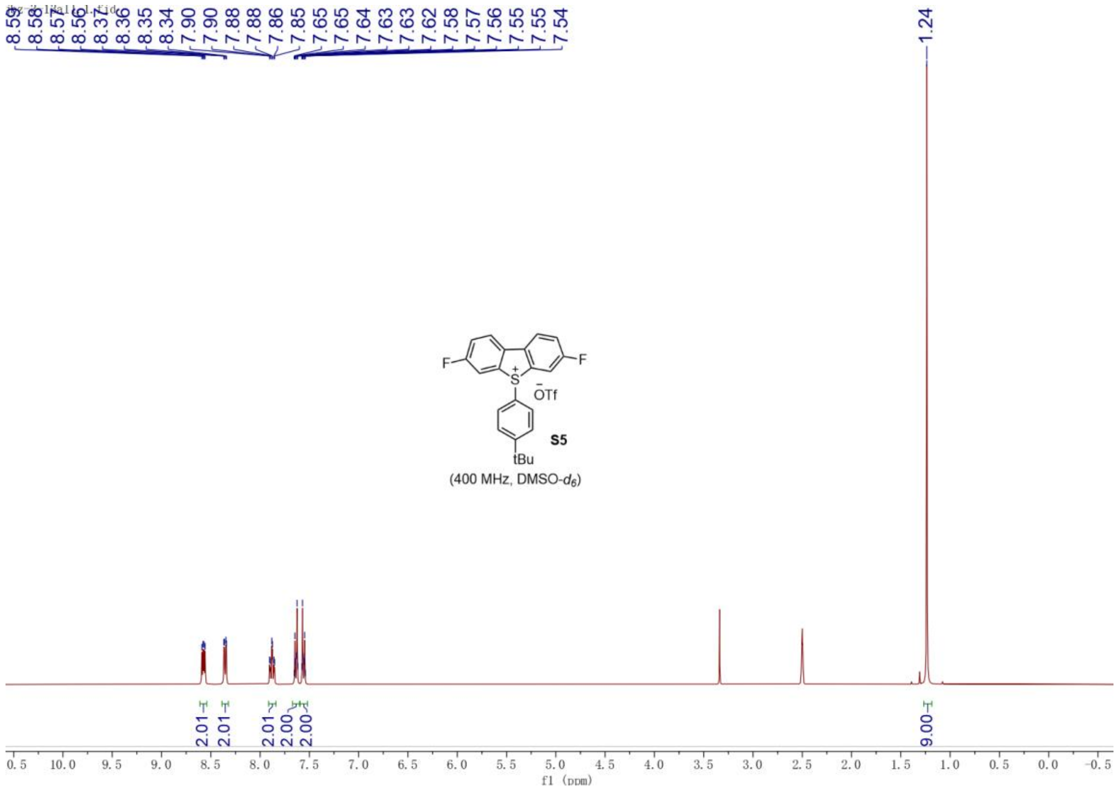
8.59 8.58 8.57 8.56 8.37 8.36 8.35 8.34 7.90 7.90 7.88 7.88 7.86 7.85 7.65 7.65 7.64 7.63 7.63 7.62 7.58 7.57 7.56 7.55 7.55 7.54
1.24
F
S
F
OTf
S5
tBu
(400 MHz, DMSO-d6)
2.01 2.01 2.01 2.00 2.00
9.00
0.5 10.0 9.5 9.0 8.5 8.0 7.5 7.0 6.5 6.0 5.5 5.0 4.5 4.0 3.5 3.0 2.5 2.0 1.5 1.0 0.5 0.0 −0.5
f1 (ppm)

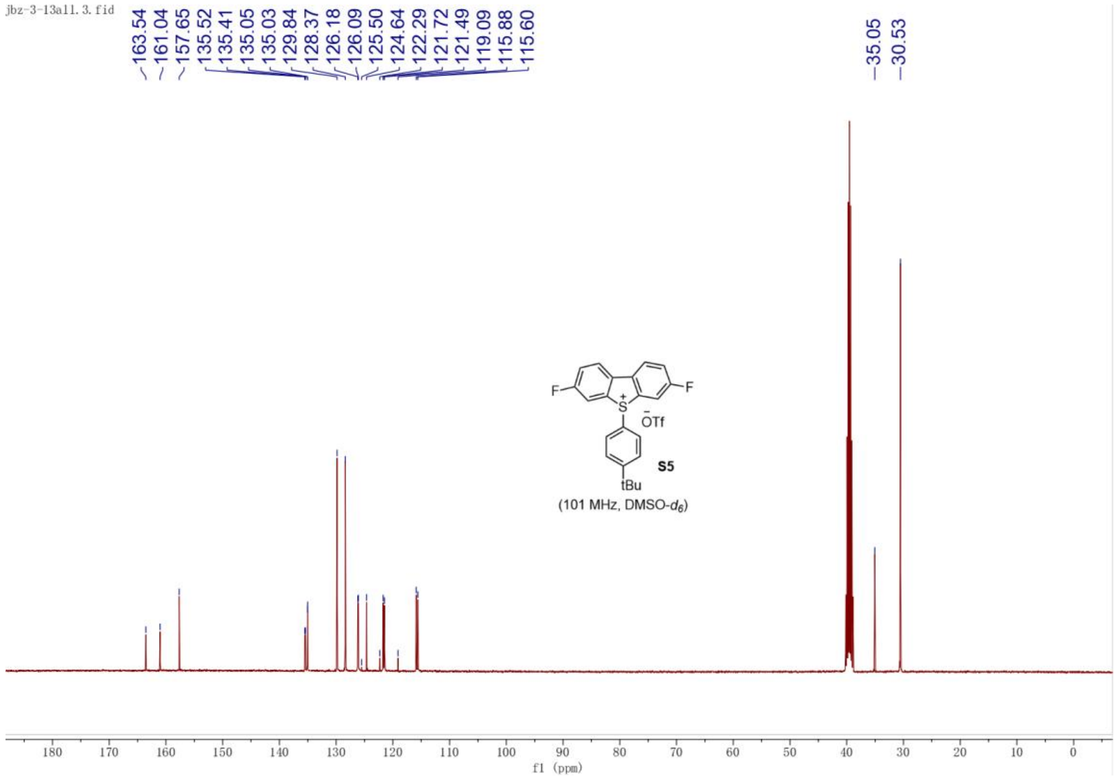
jbz-3-13all.3.fid
163.54
161.04
157.65
135.52
135.41
135.05
135.03
129.84
128.37
126.18
126.09
125.50
124.64
122.29
121.72
121.49
119.09
115.88
115.60
35.05
30.53
F
F
S
OTf
tBu
S5
(101 MHz, DMSO-$d_6$)
f1 (ppm)

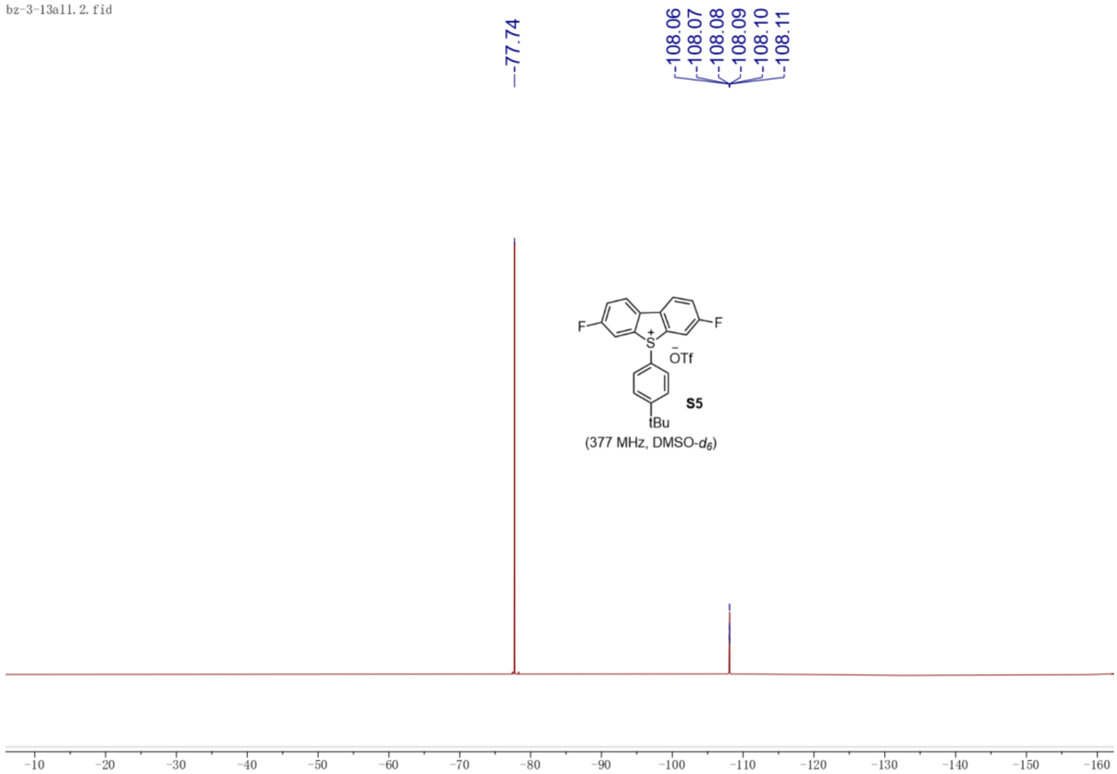
bz-3-13all.2.fid
-77.74
-108.06
-108.07
-108.08
-108.09
-108.10
-108.11
F
F
S
OTf
tBu
S5
(377 MHz, DMSO-$d_6$)
f1 (ppm)

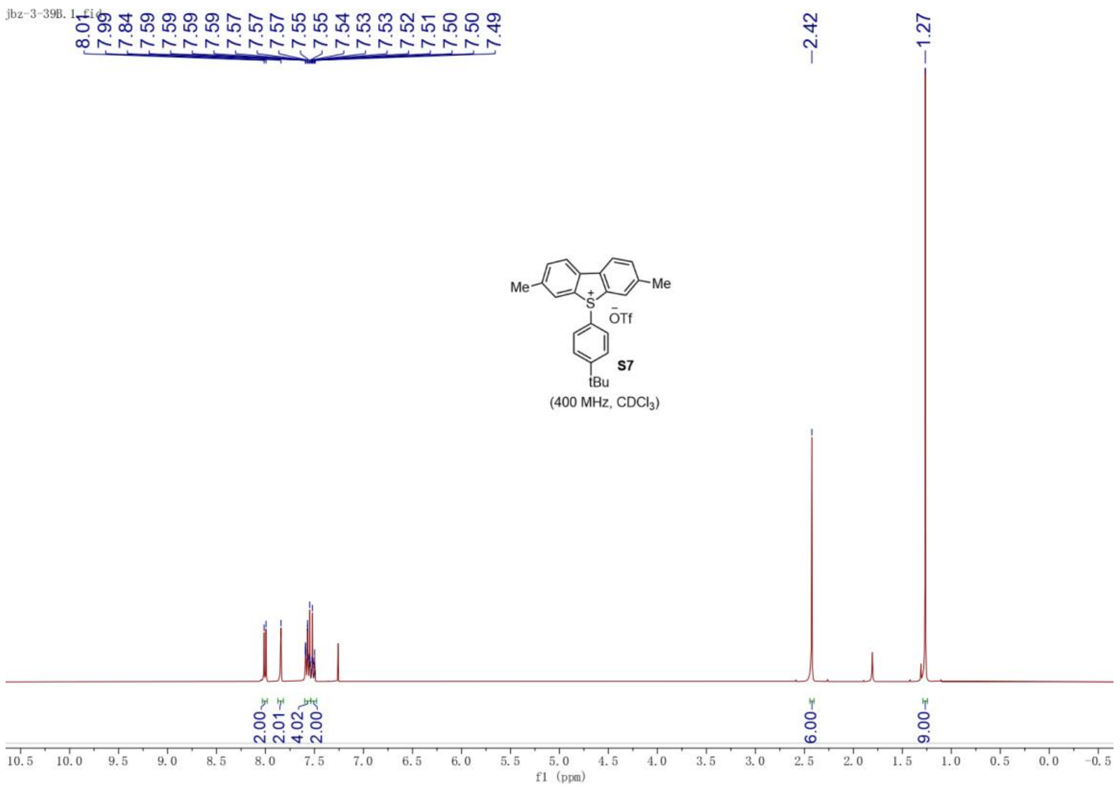
jbz-3-39B.1.fid
8.01
7.99
7.84
7.59
7.59
7.59
7.59
7.57
7.57
7.57
7.55
7.55
7.54
7.53
7.53
7.52
7.51
7.50
7.50
7.49
2.42
1.27
Me
Me
S
OTf
tBu
S7
(400 MHz, $CDCl_3$)
2.00
2.01
4.02
2.00
6.00
9.00
f1 (ppm)

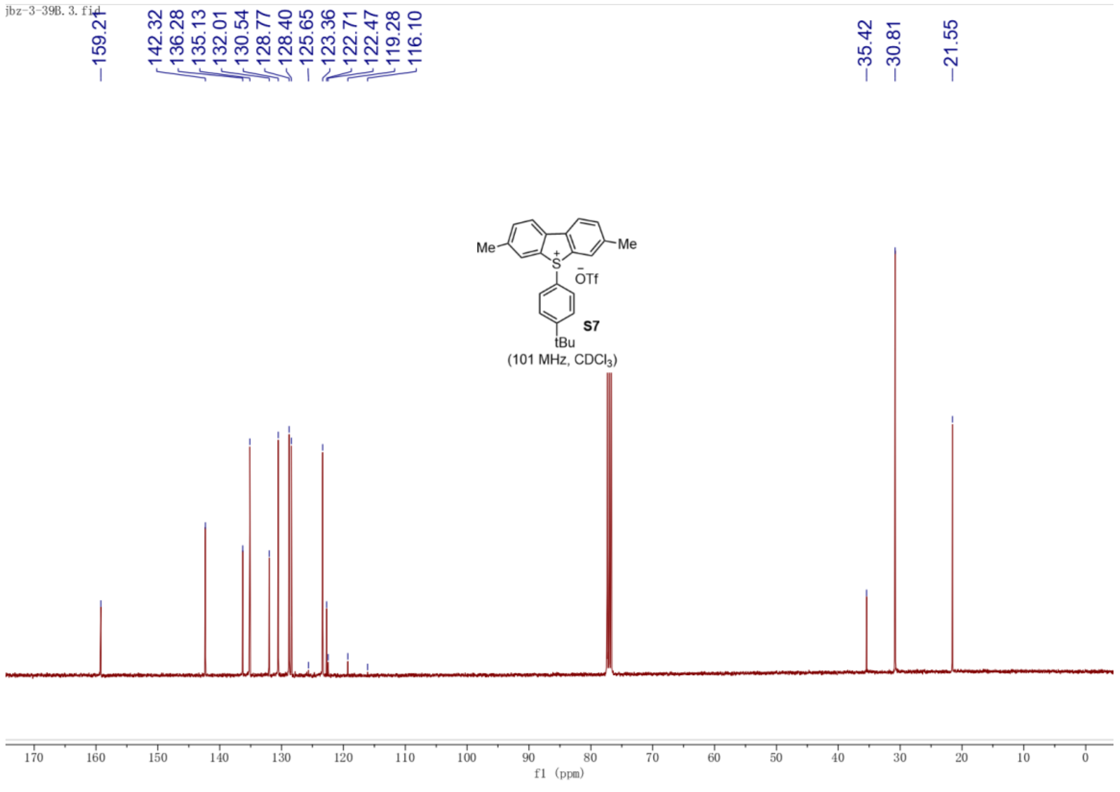
jbz-3-39B.3.fid
159.21
142.32
136.28
135.13
132.01
130.54
128.77
128.40
125.65
123.36
122.71
122.47
119.28
116.10
35.42
30.81
21.55
Me
Me
S
OTf
tBu
S7
(101 MHz, $CDCl_3$)
f1 (ppm)

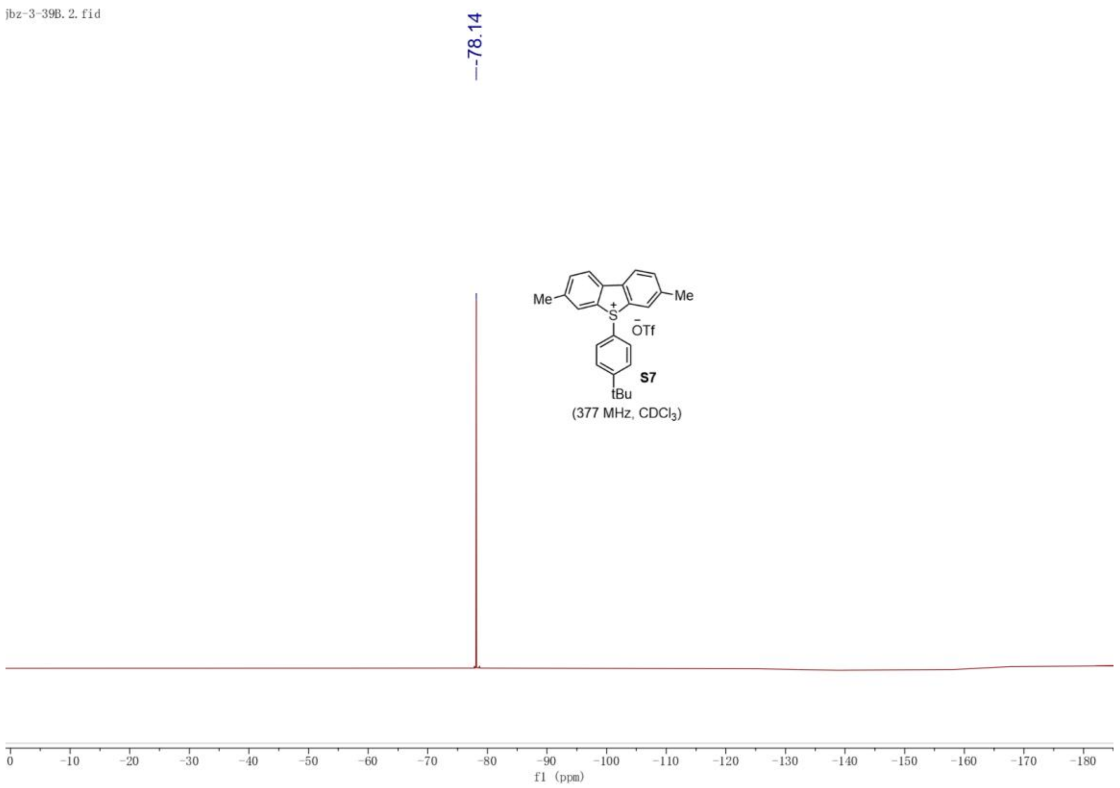
jbz-3-39B.2.fid
-78.14
Me
Me
S
OTf
tBu
S7
(377 MHz, CDCl3)
0 -10 -20 -30 -40 -50 -60 -70 -80 -90 -100 -110 -120 -130 -140 -150 -160 -170 -180
f1 (ppm)

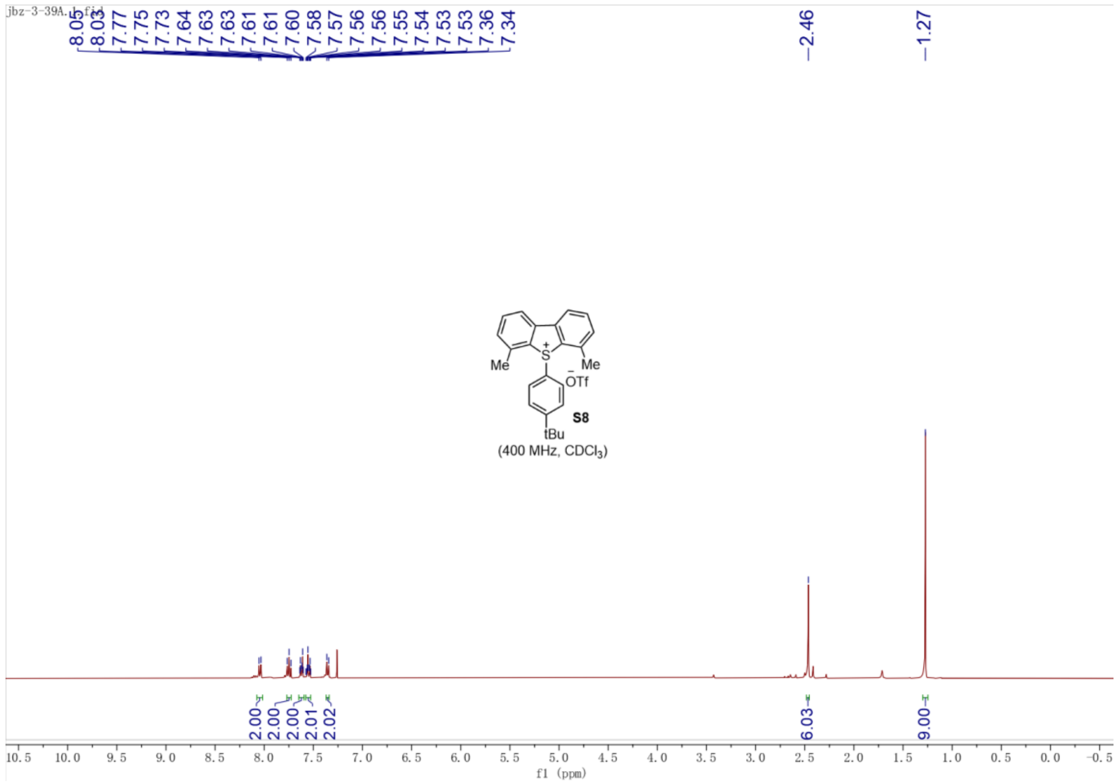
jbz-3-39A.1.fid
8.05 8.03 7.77 7.75 7.73 7.64 7.63 7.63 7.61 7.61 7.60 7.58 7.57 7.56 7.56 7.55 7.54 7.53 7.53 7.36 7.34
2.46
1.27
Me
Me
S
OTf
tBu
S8
(400 MHz, CDCl3)
2.00 2.00 2.00 2.01 2.02
6.03
9.00
10.5 10.0 9.5 9.0 8.5 8.0 7.5 7.0 6.5 6.0 5.5 5.0 4.5 4.0 3.5 3.0 2.5 2.0 1.5 1.0 0.5 0.0 -0.5
f1 (ppm)

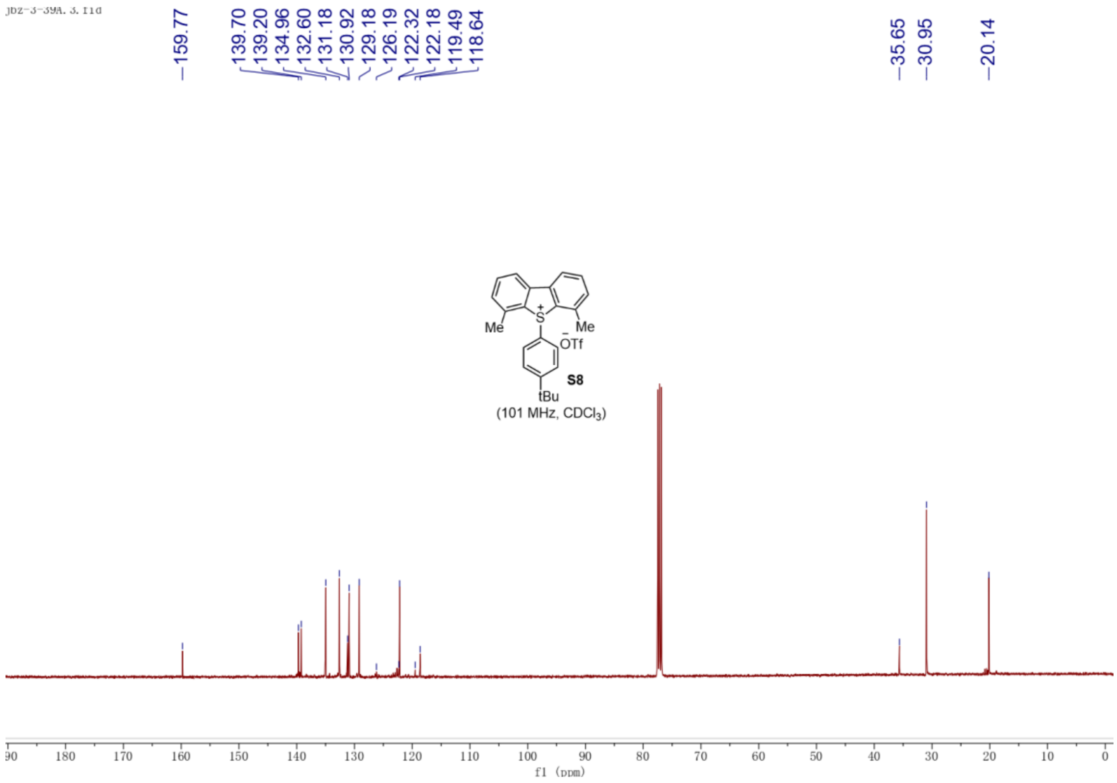
jbz-3-39A.3.fid
159.77
139.70
139.20
134.96
132.60
131.18
130.92
129.18
126.19
122.32
122.18
119.49
118.64
35.65
30.95
20.14
Me
S
Me
OTf
tBu
S8
(101 MHz, CDCl$_3$)
f1 (ppm)

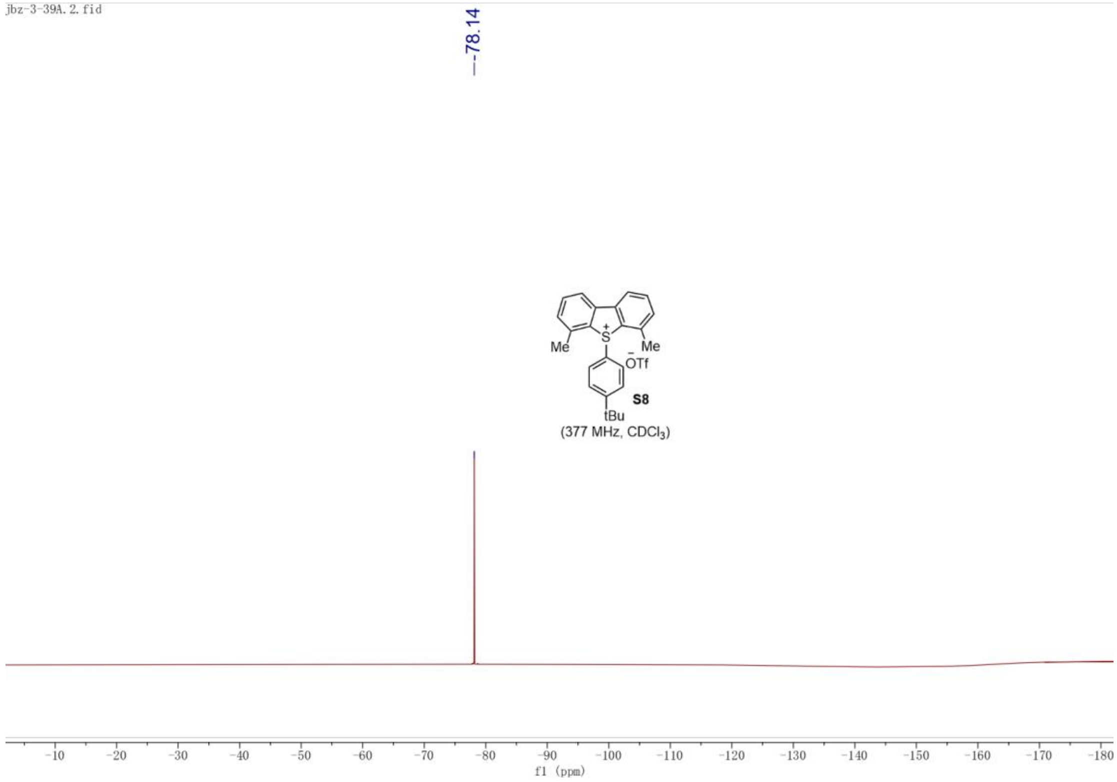
jbz-3-39A.2.fid
-78.14
Me
S
Me
OTf
tBu
S8
(377 MHz, CDCl$_3$)
f1 (ppm)

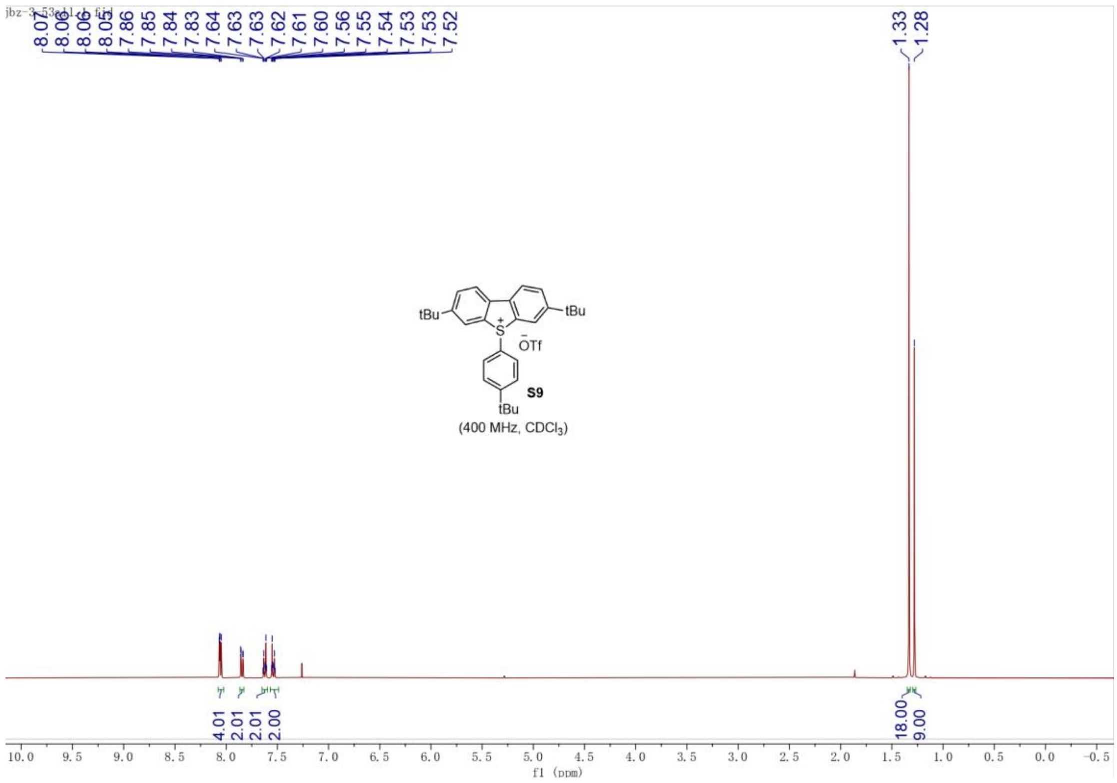
jbz-3-53all.1.fid
8.07
8.06
8.06
8.05
7.86
7.85
7.84
7.83
7.64
7.63
7.63
7.62
7.61
7.60
7.56
7.55
7.54
7.53
7.53
7.52
1.33
1.28
tBu
tBu
S
OTf
tBu
S9
(400 MHz, CDCl3)
4.01
2.01
2.01
2.00
18.00
9.00
f1 (ppm)

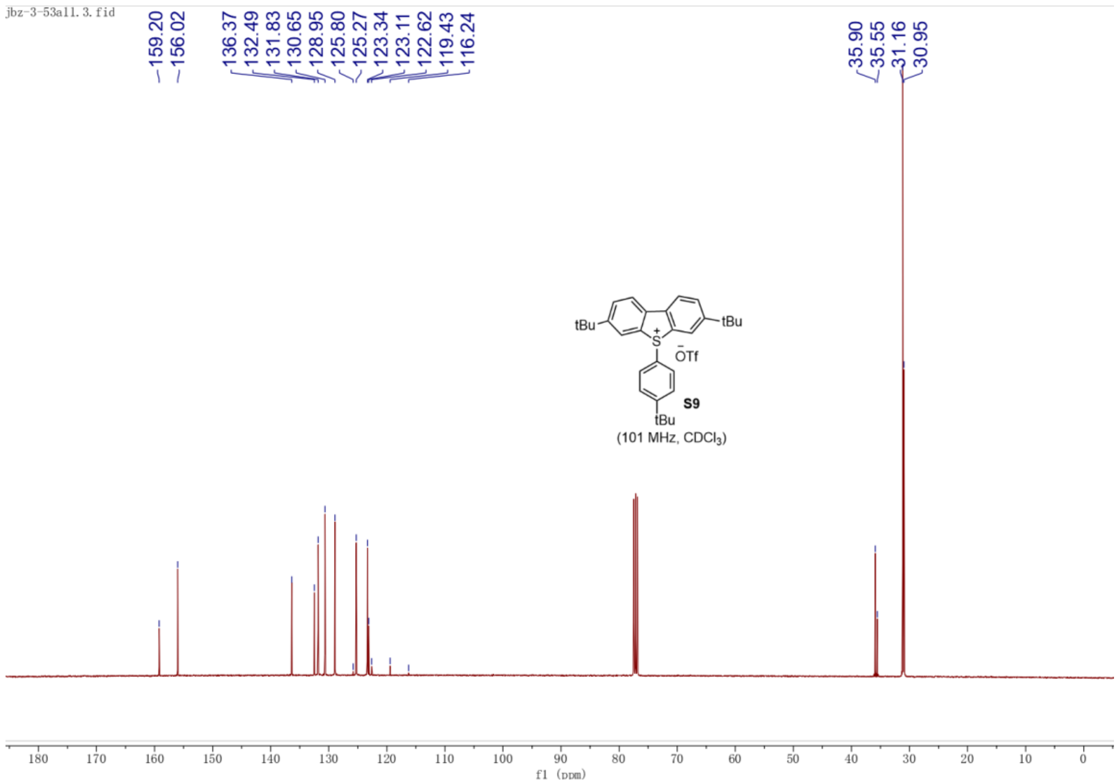
jbz-3-53all.3.fid
159.20
156.02
136.37
132.49
131.83
130.65
128.95
125.80
125.27
123.34
123.11
122.62
119.43
116.24
35.90
35.55
31.16
30.95
tBu
tBu
S
OTf
tBu
S9
(101 MHz, CDCl3)
f1 (ppm)

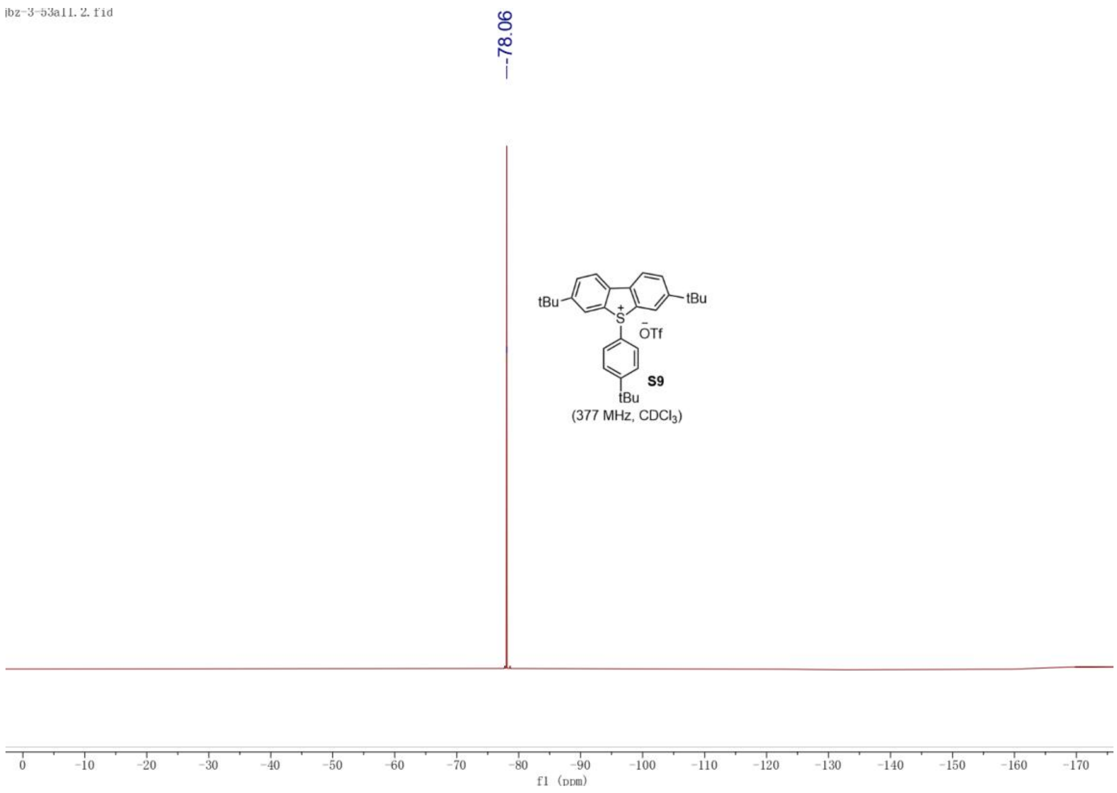
jbz-3-53a11.2.fid
-78.06
tBu
tBu
OTf
tBu
S9
(377 MHz, CDCl3)
f1 (ppm)

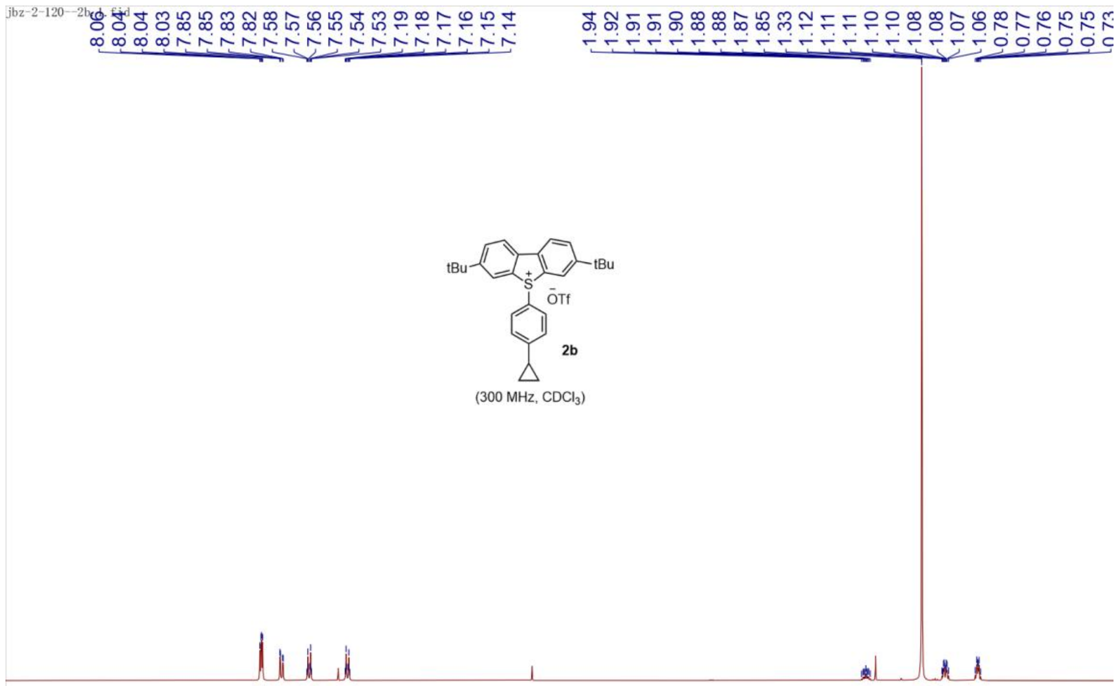
jbz-2-120--2b
8.04
8.03
7.85
7.85
7.83
7.82
7.58
7.57
7.56
7.55
7.54
7.53
7.19
7.18
7.17
7.16
7.15
7.14
1.94
1.92
1.91
1.90
1.88
1.87
1.85
1.33
1.12
1.11
1.11
1.10
1.10
1.08
1.07
1.06
0.78
0.76
0.75
0.75
tBu
tBu
OTf
2b
(300 MHz, CDCl3)
2.00
2.00
2.00
2.00
1.03
18.00
2.01
2.00

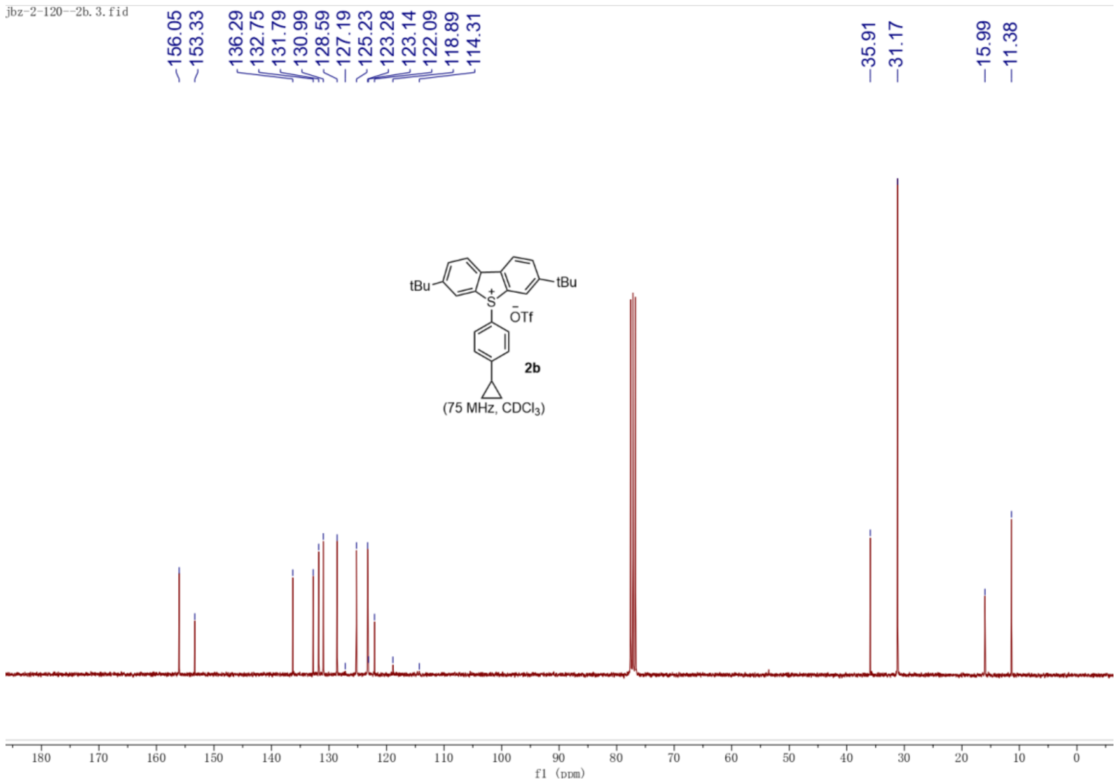
jbz-2-120--2b.3.fid
156.05
153.33
136.29
132.75
131.79
130.99
128.59
127.19
125.23
123.28
123.14
122.09
118.89
114.31
35.91
31.17
15.99
11.38
tBu
tBu
OTf
2b
(75 MHz, CDCl3)
f1 (ppm)

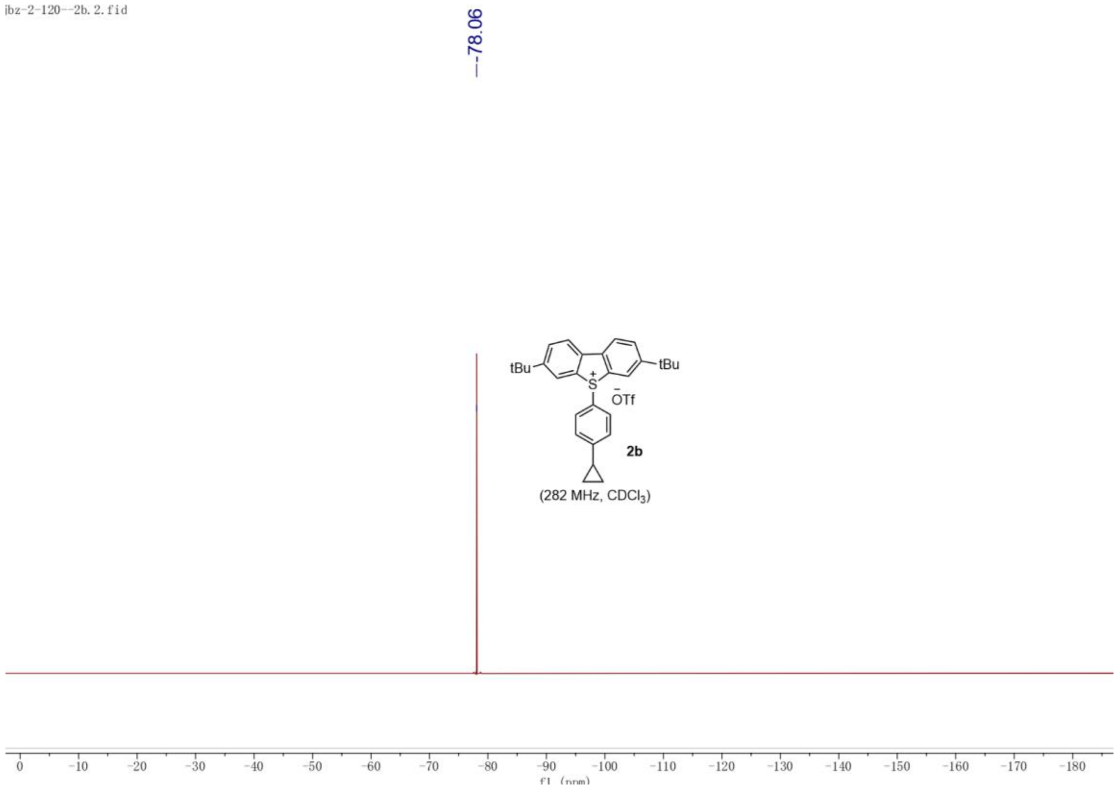
jbz-2-120--2b.2.fid
-78.06
tBu
tBu
OTf
2b
(282 MHz, CDCl3)
f1 (ppm)

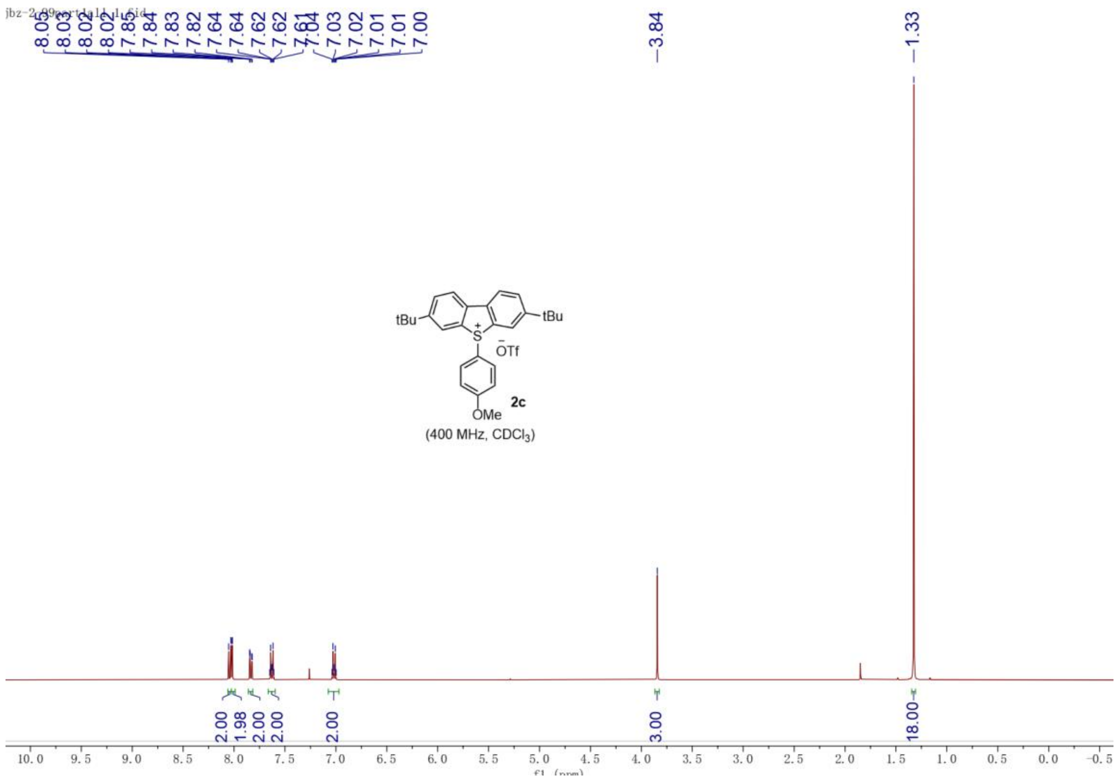
jbz-2-99part1all.1.fid
8.05
8.03
8.02
8.02
7.85
7.84
7.83
7.82
7.64
7.64
7.62
7.62
7.61
7.04
7.03
7.02
7.01
7.01
7.00
3.84
1.33
tBu
tBu
S
OTf
OMe
2c
(400 MHz, CDCl3)
2.00
1.98
2.00
2.00
2.00
3.00
18.00
10.0
9.5
9.0
8.5
8.0
7.5
7.0
6.5
6.0
5.5
5.0
4.5
4.0
3.5
3.0
2.5
2.0
1.5
1.0
0.5
0.0
-0.5
f1 (ppm)

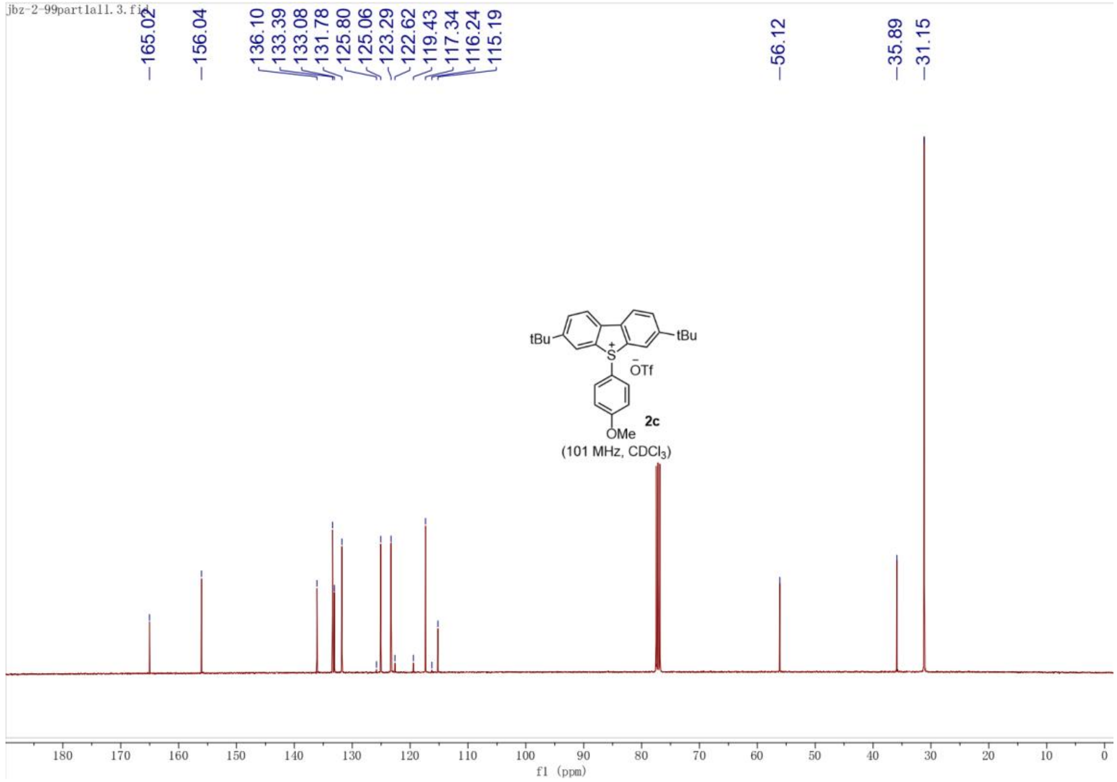
jbz-2-99part1all.3.fid
165.02
156.04
136.10
133.39
133.08
131.78
125.80
125.06
123.29
122.62
119.43
117.34
116.24
115.19
56.12
35.89
31.15
tBu
tBu
S
OTf
OMe
2c
(101 MHz, CDCl3)
180
170
160
150
140
130
120
110
100
90
80
70
60
50
40
30
20
10
0
f1 (ppm)

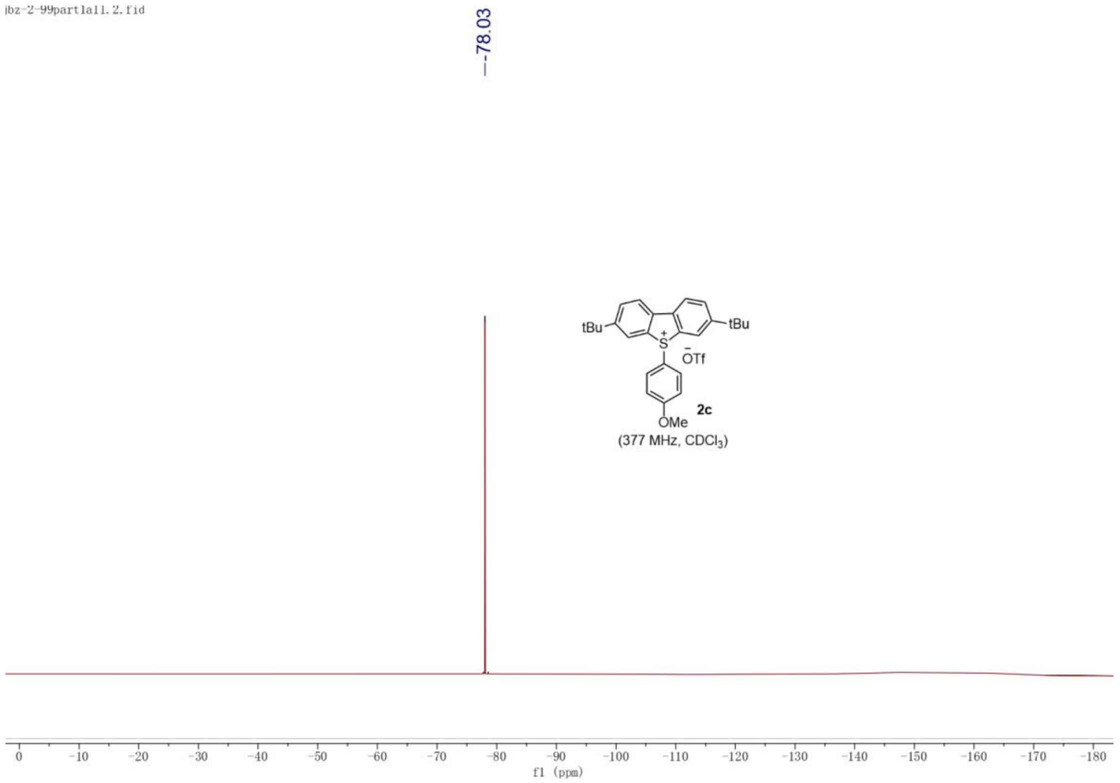
jbz-2-99partlall.2.fid
-78.03
tBu
tBu
S
OTf
OMe
2c
(377 MHz, CDCl3)
f1 (ppm)

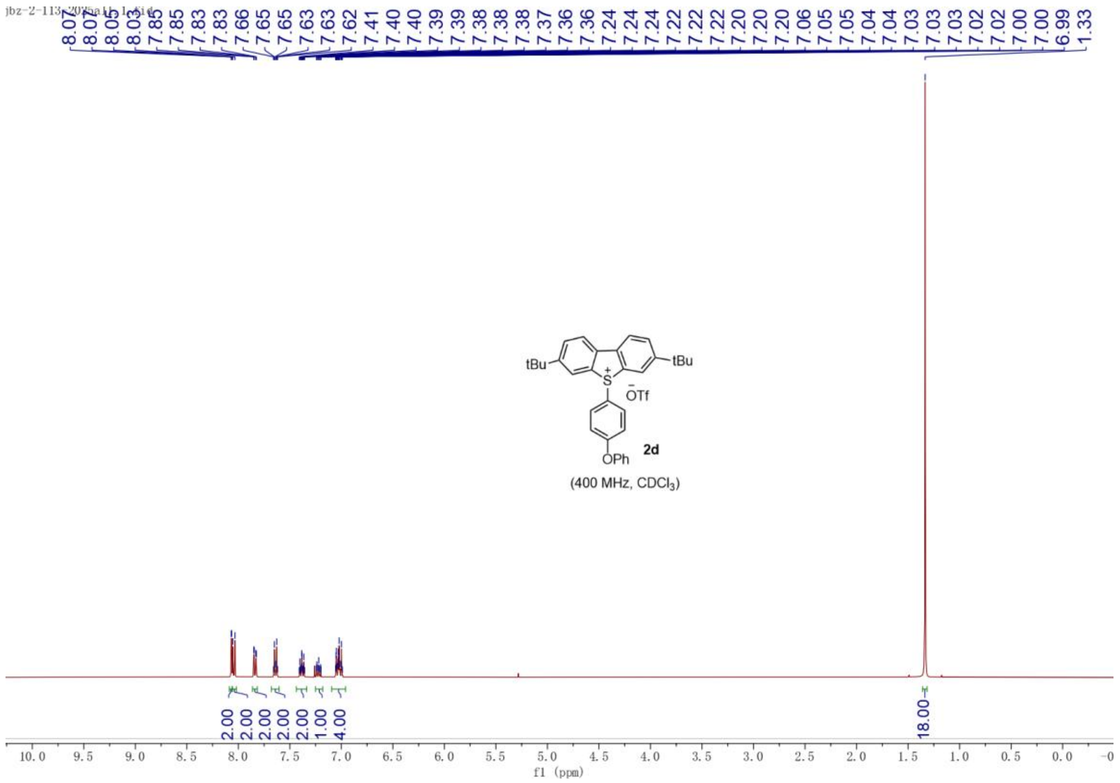
8.07
8.07
8.05
8.03
7.85
7.85
7.83
7.66
7.65
7.65
7.63
7.63
7.62
7.41
7.40
7.39
7.39
7.38
7.38
7.38
7.37
7.36
7.36
7.24
7.24
7.24
7.22
7.22
7.22
7.20
7.20
7.20
7.06
7.05
7.05
7.04
7.04
7.03
7.03
7.03
7.02
7.02
7.00
7.00
6.99
1.33
tBu
tBu
S
OTf
OPh
2d
(400 MHz, CDCl3)
2.00
2.00
2.00
2.00
2.00
1.00
4.00
18.00
f1 (ppm)

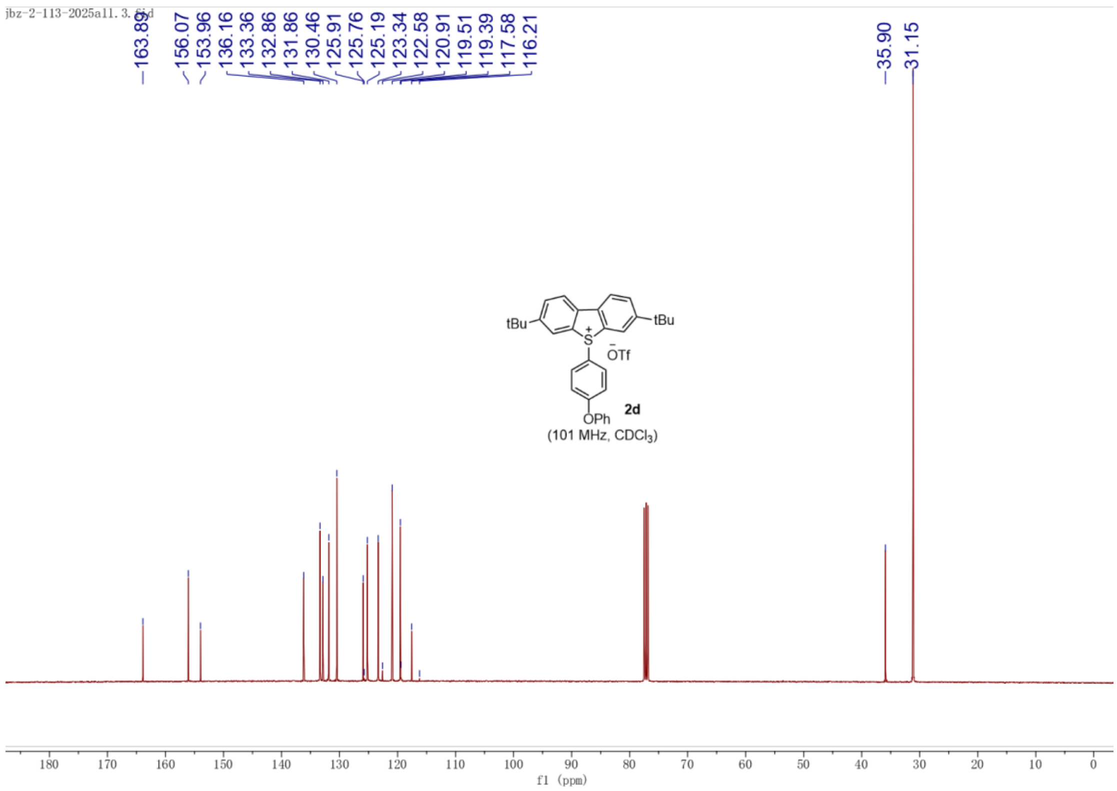
jbz-2-113-2025all.3.fid
163.89
156.07
153.96
136.16
133.36
132.86
131.86
130.46
125.91
125.76
125.19
123.34
122.58
120.91
119.51
119.39
117.58
116.21
35.90
31.15
tBu
tBu
S
OTf
OPh
2d
(101 MHz, CDCl3)
f1 (ppm)

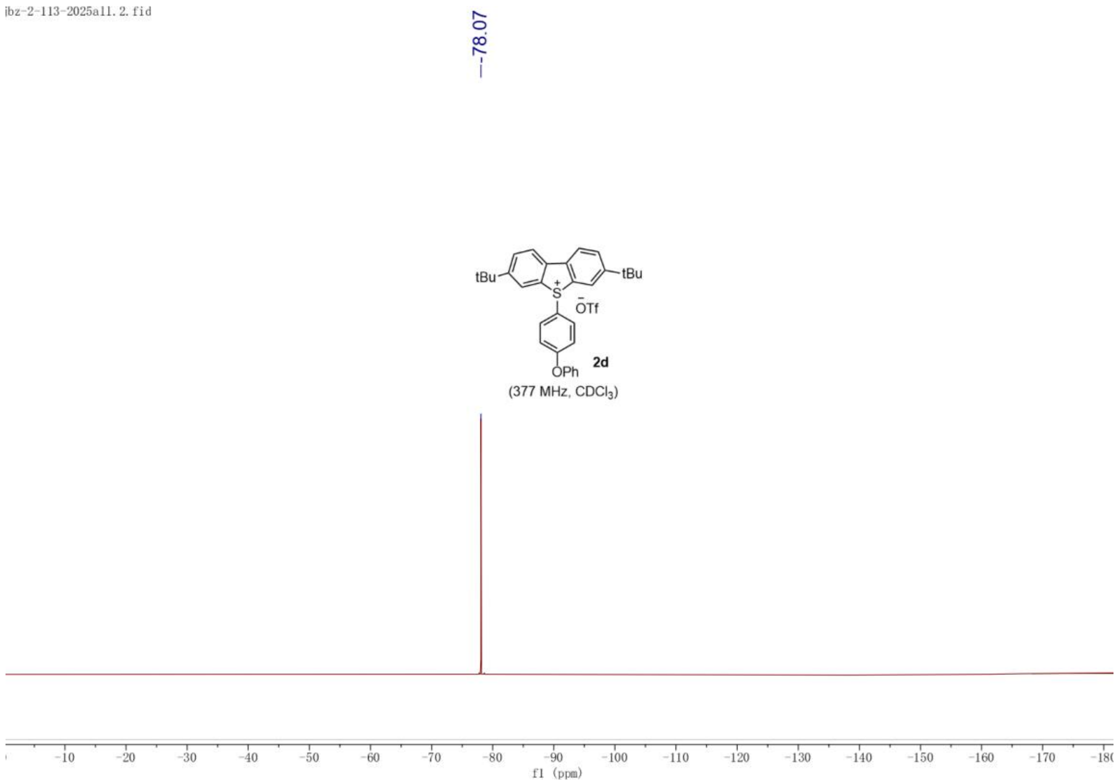
jbz-2-113-2025all.2.fid
-78.07
tBu
tBu
S
OTf
OPh
2d
(377 MHz, CDCl3)
f1 (ppm)

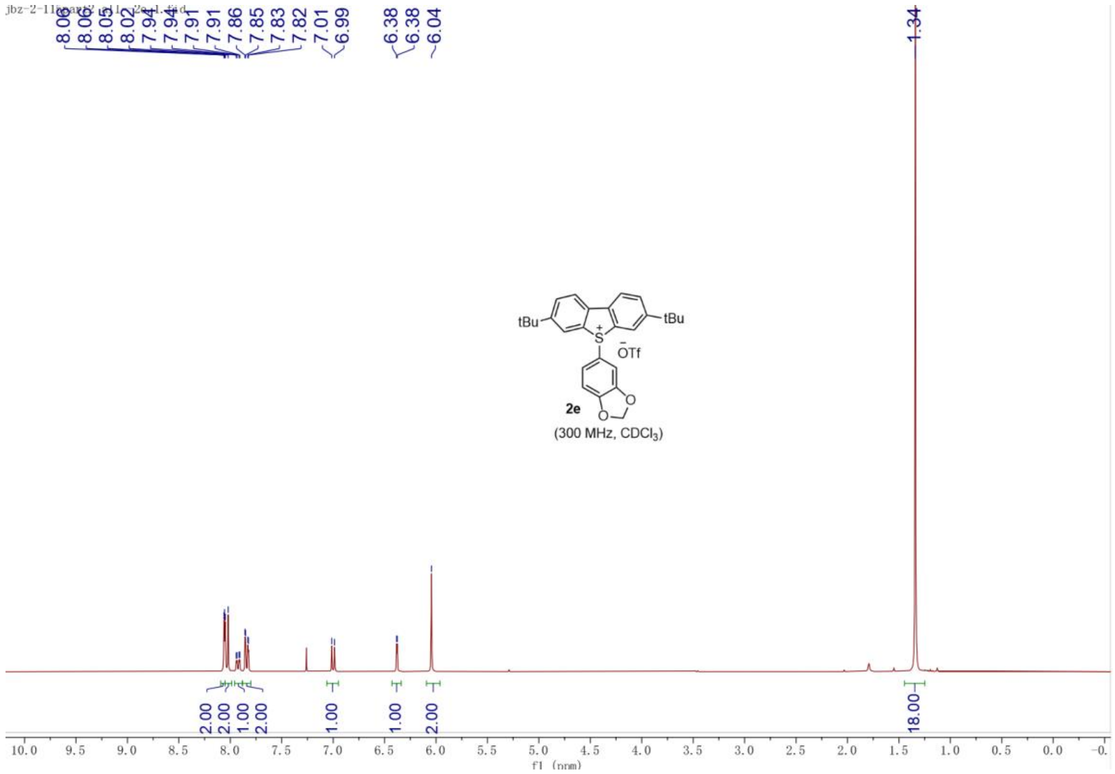
8.06
8.06
8.05
8.02
7.94
7.91
7.91
7.86
7.85
7.83
7.82
7.01
6.99
6.38
6.38
6.04
1.34
tBu
tBu
S
OTf
O
O
2e
(300 MHz, CDCl3)
2.00
2.00
1.00
2.00
1.00
1.00
2.00
18.00
10.0
9.5
9.0
8.5
8.0
7.5
7.0
6.5
6.0
5.5
5.0
4.5
4.0
3.5
3.0
2.5
2.0
1.5
1.0
0.5
0.0
f1 (ppm)

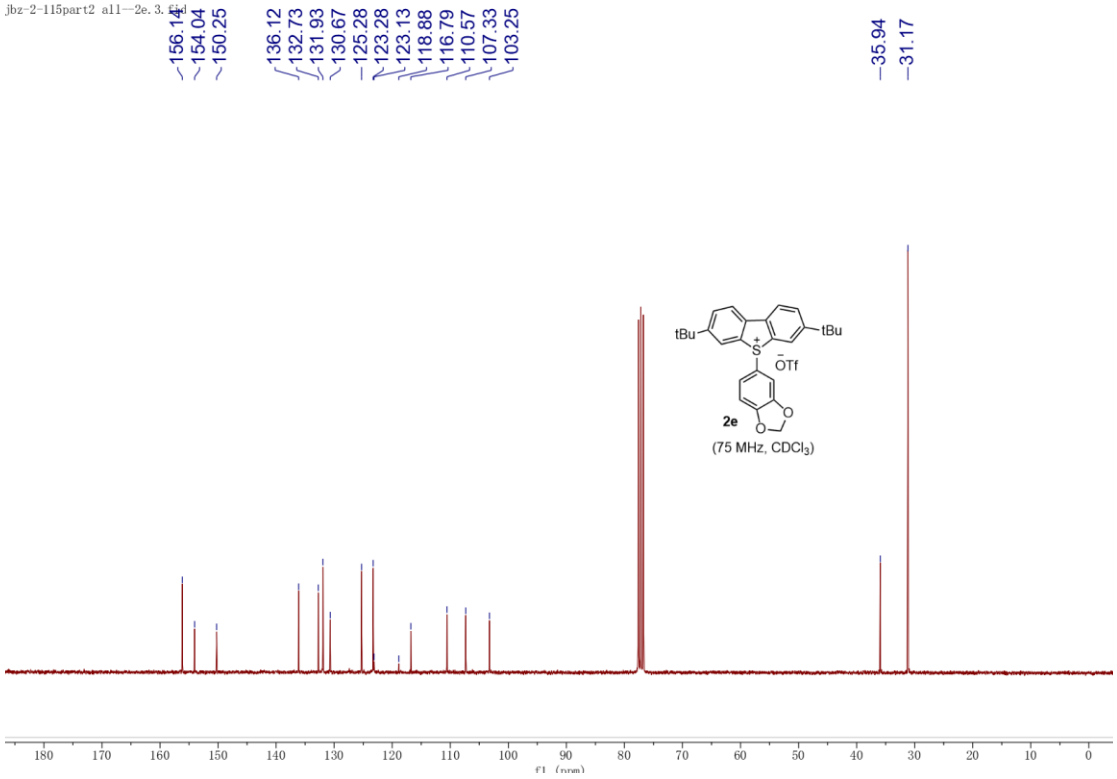
jbz-2-115part2 all—2e.3.fid
156.14
154.04
150.25
136.12
132.73
131.93
130.67
125.28
123.28
123.13
118.88
116.79
110.57
107.33
103.25
35.94
31.17
tBu
tBu
S
OTf
O
O
2e
(75 MHz, CDCl3)
180
170
160
150
140
130
120
110
100
90
80
70
60
50
40
30
20
10
0
f1 (ppm)

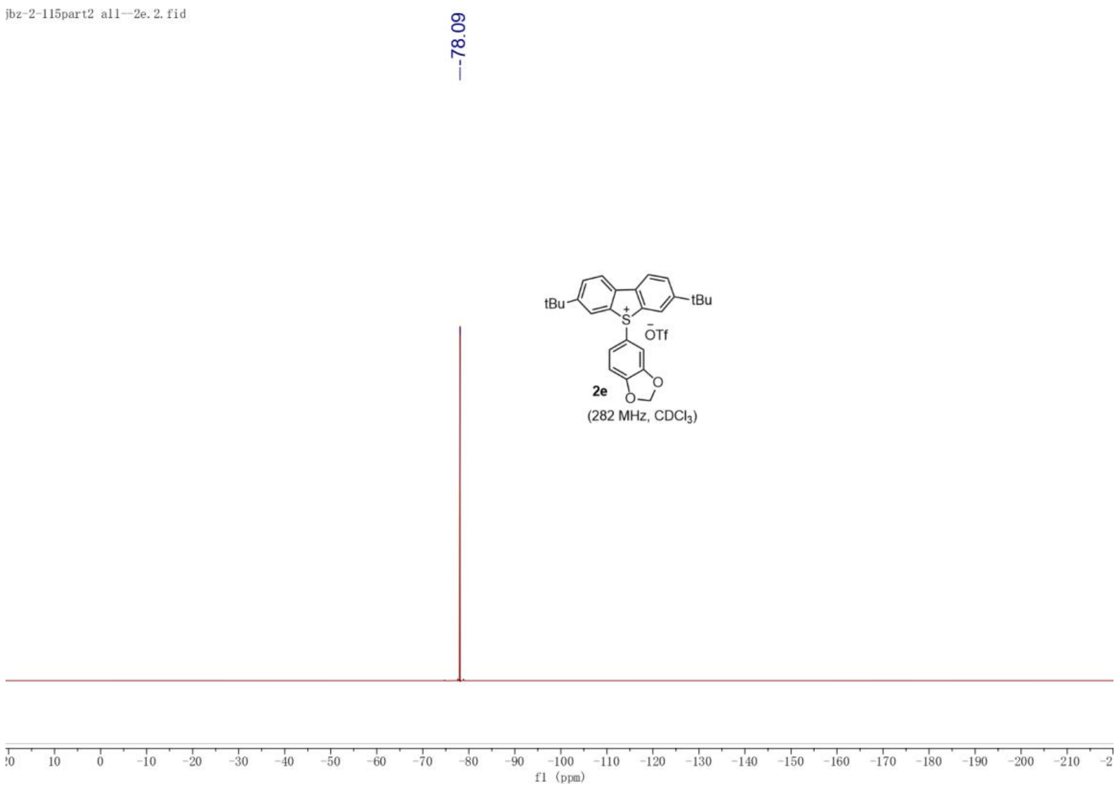
jbz-2-115part2 all—2e.2.fid
-78.09
tBu
tBu
OTf
2e
(282 MHz, CDCl3)
f1 (ppm)

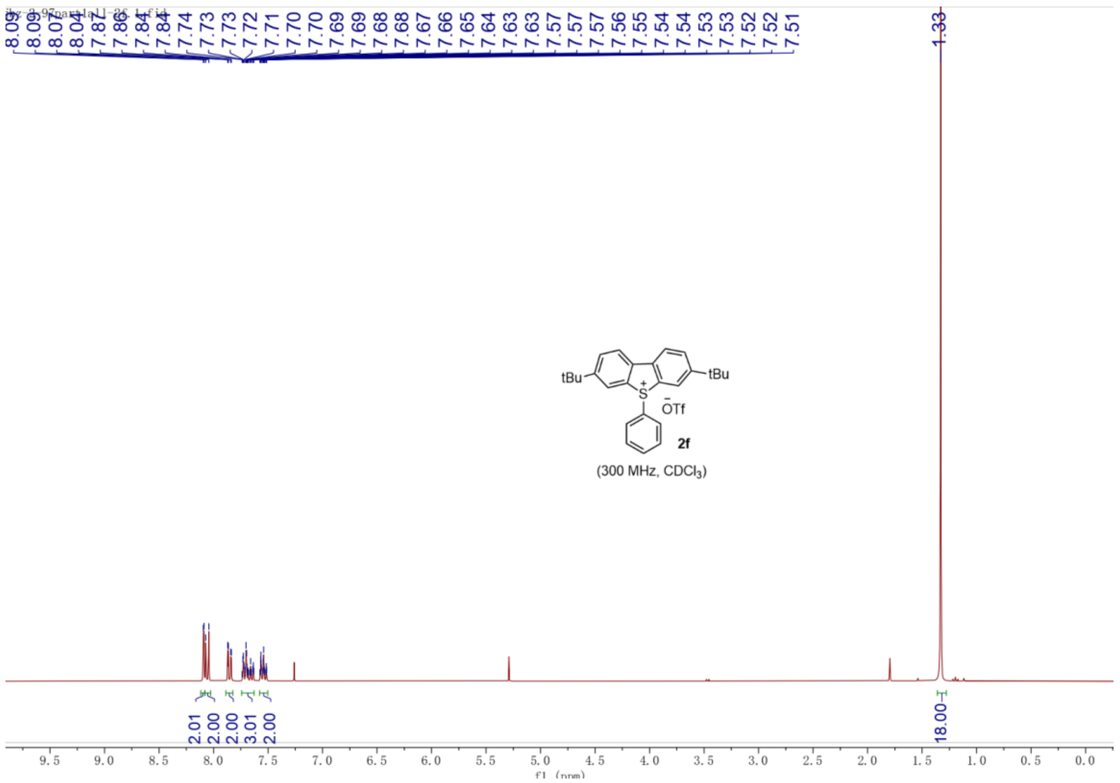
tBu
tBu
OTf
2f
(300 MHz, CDCl3)
1.33
2.01
2.00
2.00
3.01
2.00
18.00
f1 (ppm)

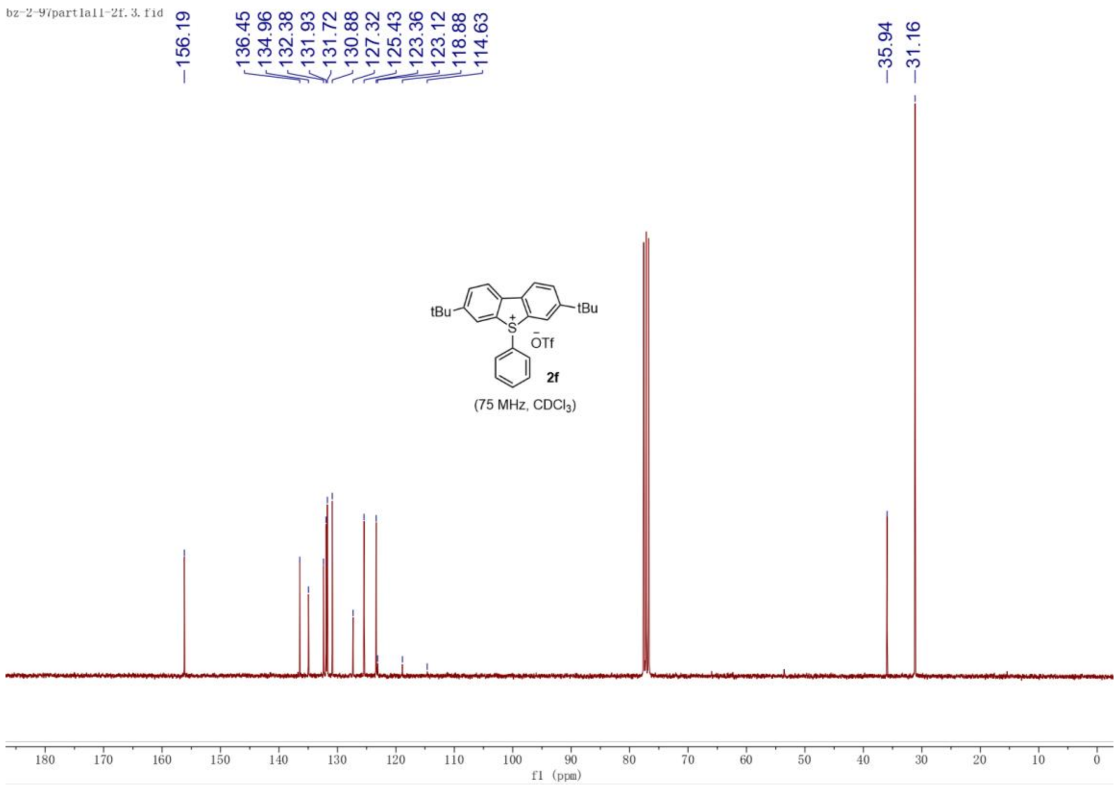
bz-2-97part1all-2f.3.fid
156.19
136.45
134.96
132.38
131.93
131.72
130.88
127.32
125.43
123.36
123.12
118.88
114.63
35.94
31.16
tBu
tBu
S
OTf
2f
(75 MHz, $CDCl_3$)
f1 (ppm)

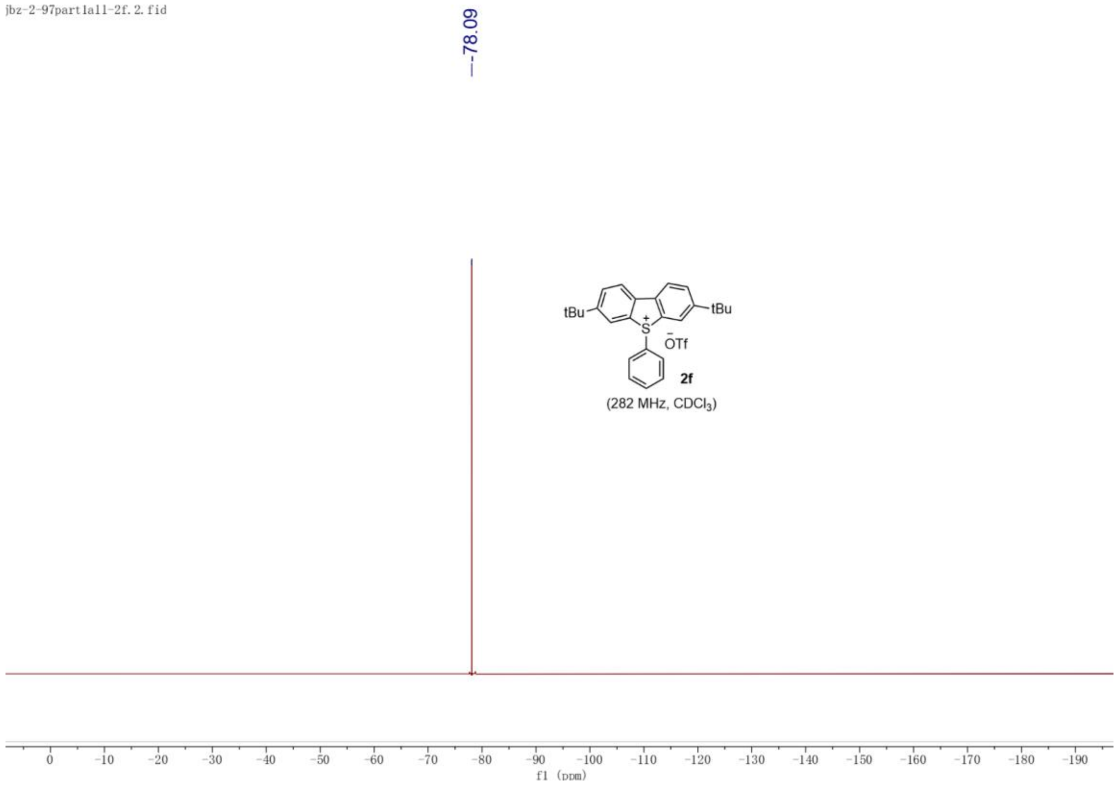
jbz-2-97part1all-2f.2.fid
-78.09
tBu
tBu
S
OTf
2f
(282 MHz, $CDCl_3$)
f1 (ppm)

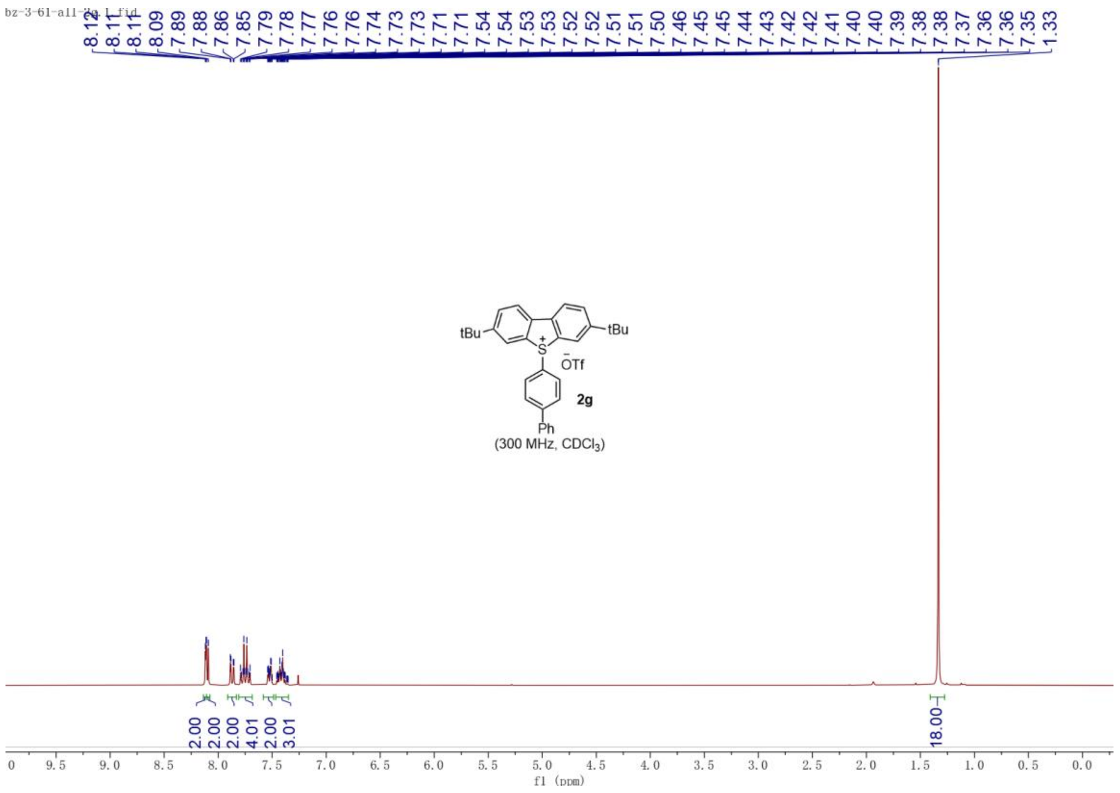

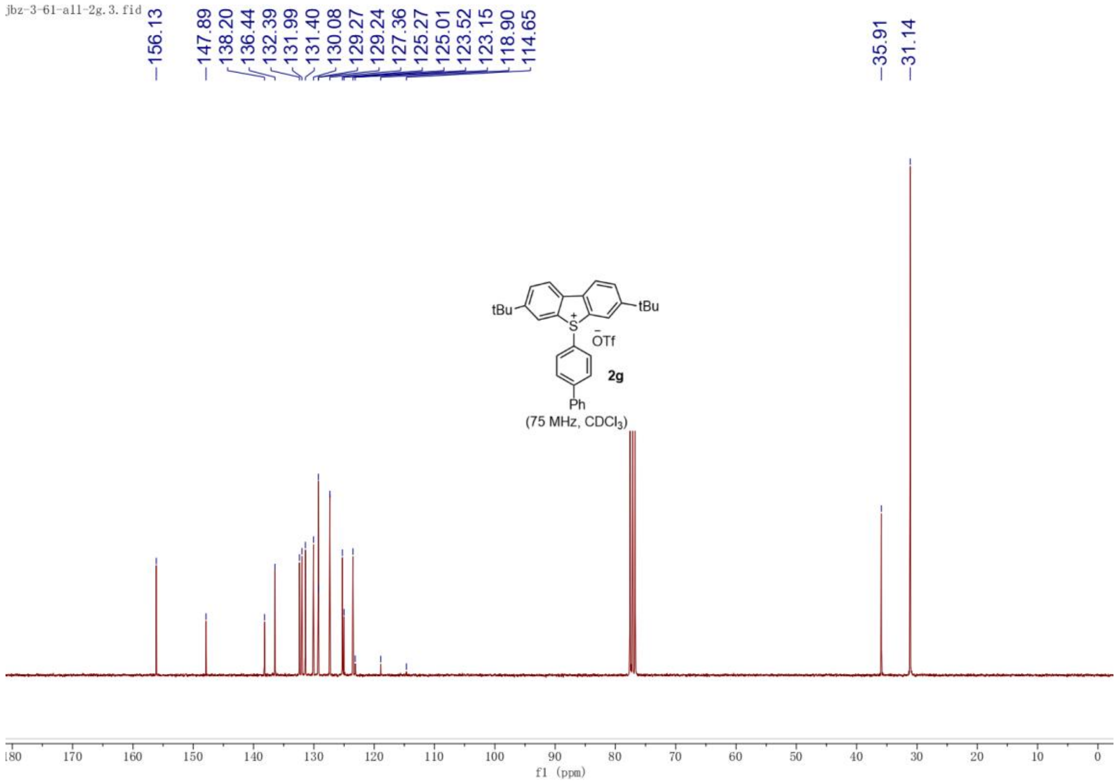

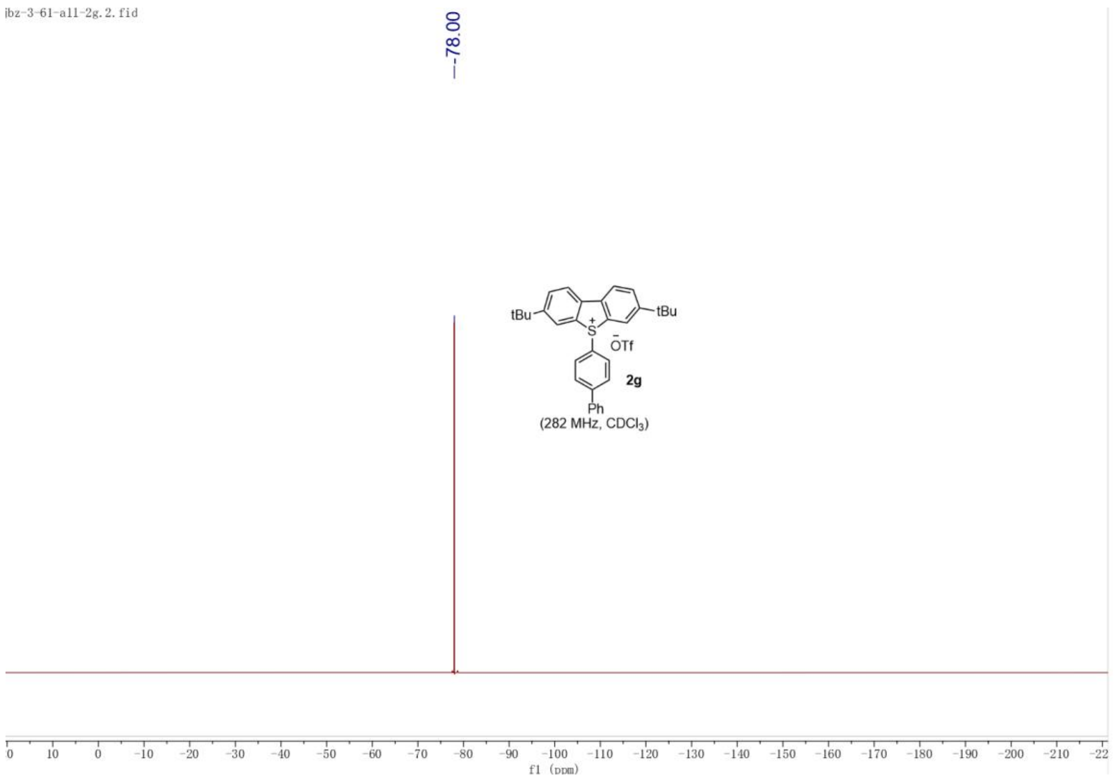
jbz-3-61-all-2g.2.fid
-78.00
tBu
tBu
S
OTf
2g
Ph
(282 MHz, CDCl3)
f1 (ppm)

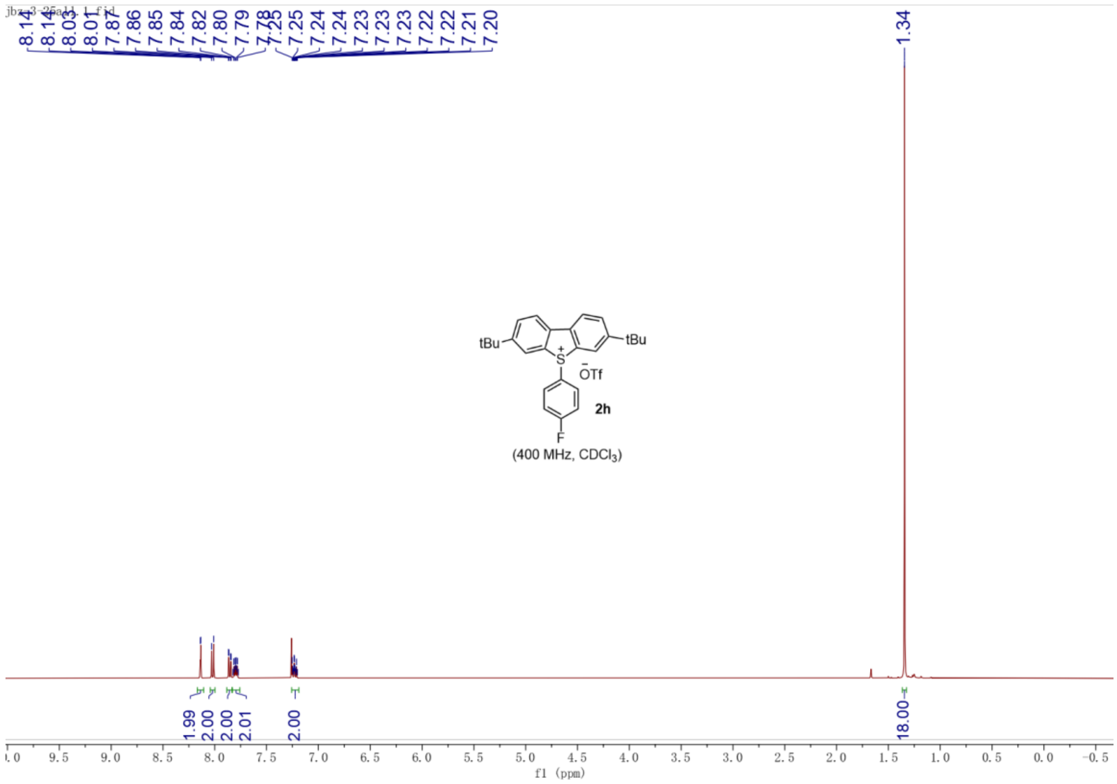
1.34
tBu
tBu
S
OTf
2h
F
(400 MHz, CDCl3)
1.99
2.00
2.00
2.01
2.00
18.00
f1 (ppm)

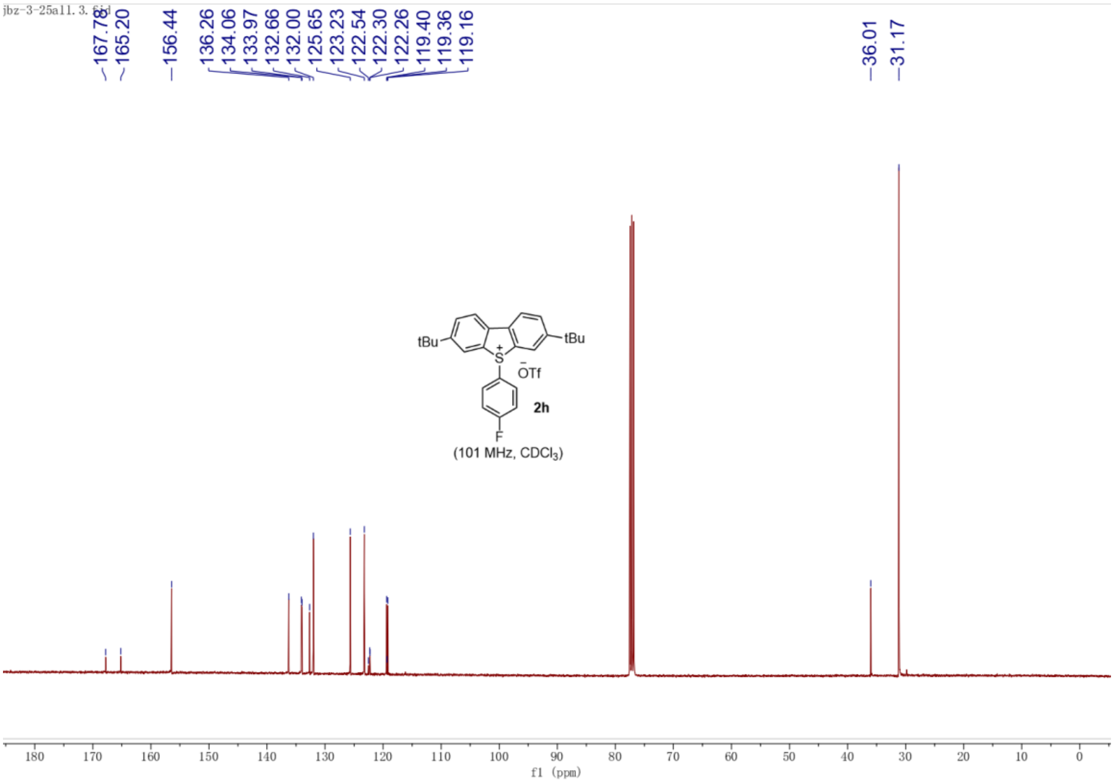
jbz-3-25al1.3.fid
167.78
165.20
156.44
136.26
134.06
133.97
132.66
132.00
125.65
123.23
122.54
122.30
122.26
119.40
119.36
119.16
36.01
31.17
tBu
tBu
S
OTf
F
2h
(101 MHz, CDCl3)
180
170
160
150
140
130
120
110
100
90
80
70
60
50
40
30
20
10
0
f1 (ppm)

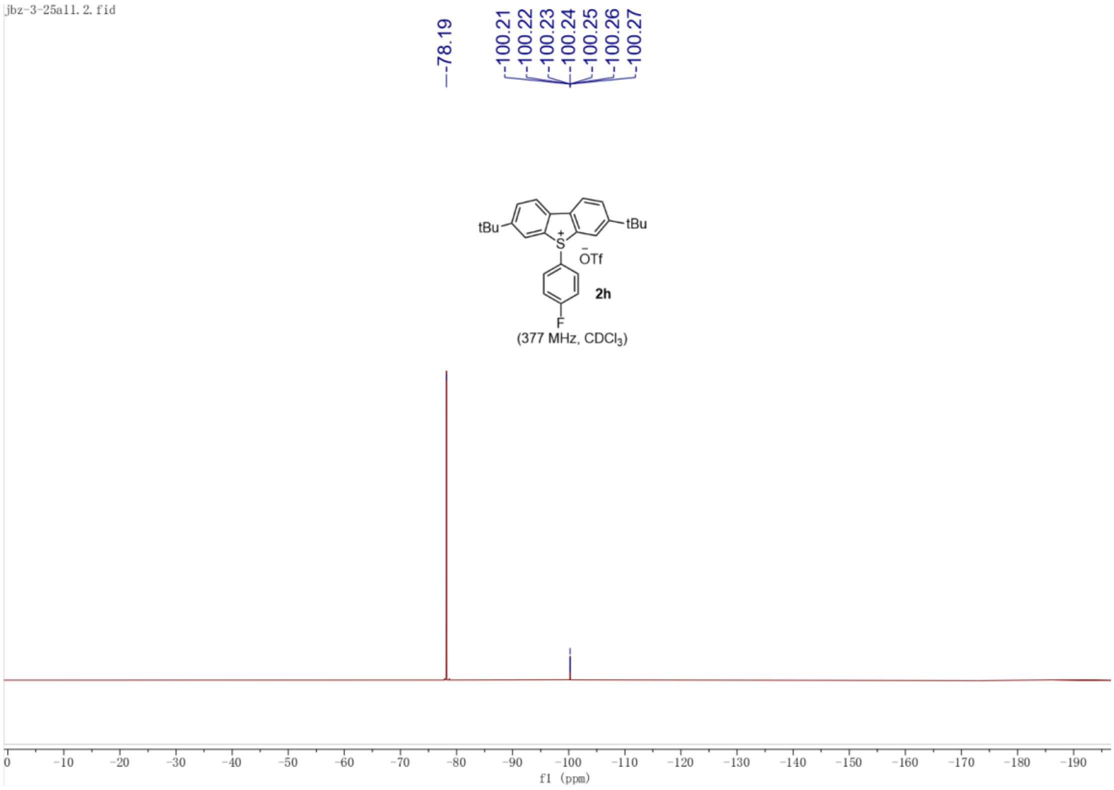
jbz-3-25al1.2.fid
-78.19
-100.21
-100.22
-100.23
-100.24
-100.25
-100.26
-100.27
tBu
tBu
S
OTf
F
2h
(377 MHz, CDCl3)
0
-10
-20
-30
-40
-50
-60
-70
-80
-90
-100
-110
-120
-130
-140
-150
-160
-170
-180
-190
f1 (ppm)

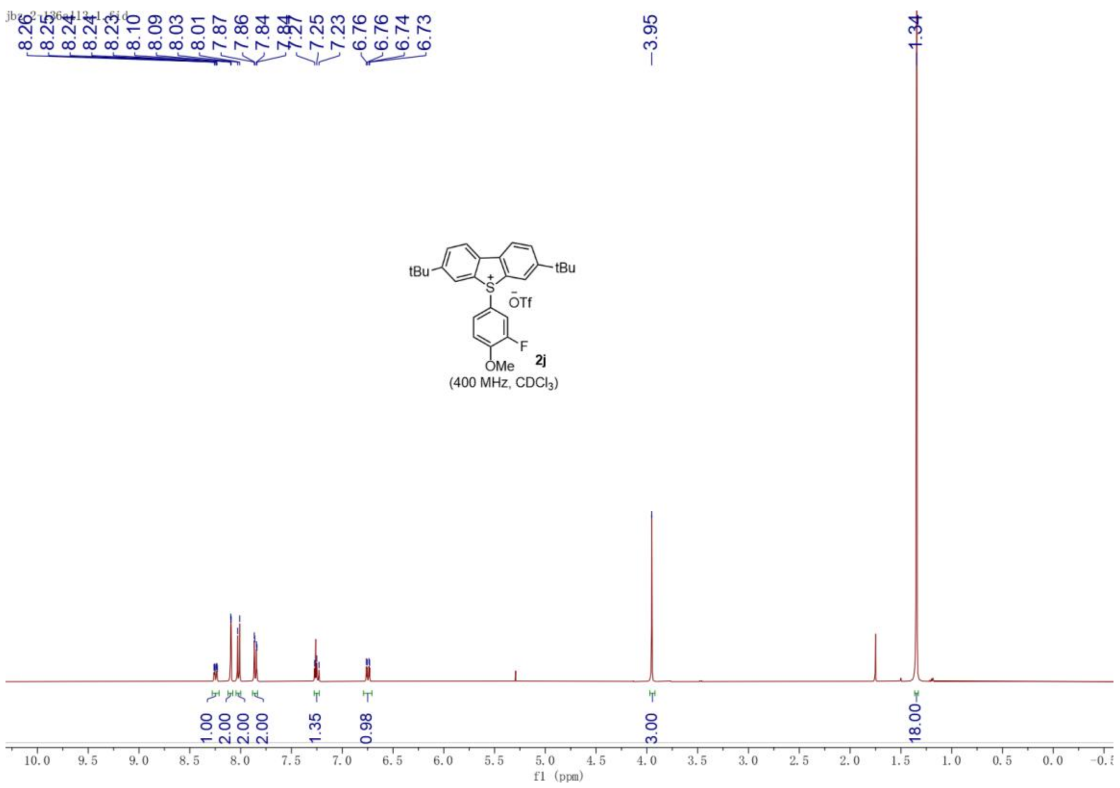
jbz-2-136a113.1.fid
8.26
8.25
8.24
8.24
8.23
8.10
8.09
8.03
8.01
7.87
7.86
7.84
7.84
7.27
7.25
7.23
6.76
6.76
6.74
6.73
3.95
1.34
tBu
tBu
S
OTf
F
OMe
2j
(400 MHz, CDCl3)
1.00
2.00
2.00
2.00
1.35
0.98
3.00
18.00
f1 (ppm)

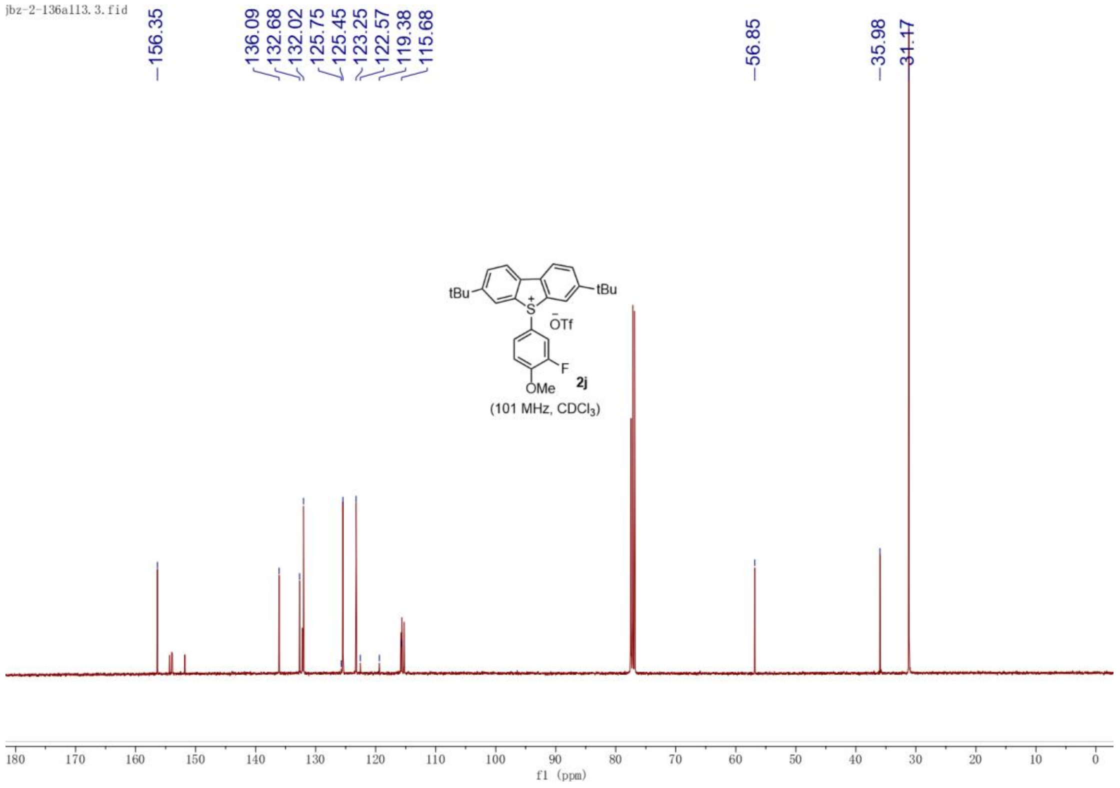
jbz-2-136a113.3.fid
156.35
136.09
132.68
132.02
125.75
125.45
123.25
122.57
119.38
115.68
56.85
35.98
31.17
tBu
tBu
S
OTf
F
OMe
2j
(101 MHz, CDCl3)
f1 (ppm)

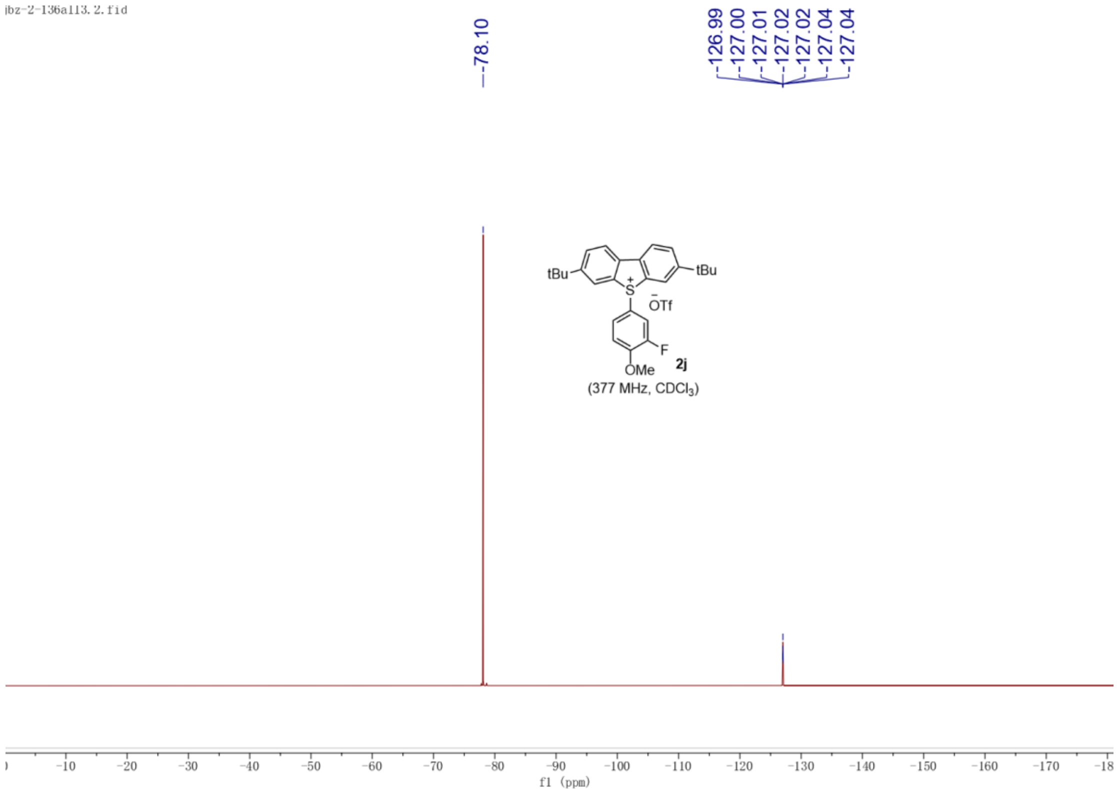
jbz-2-136a113.2.fid
-78.10
-126.99
-127.00
-127.01
-127.02
-127.02
-127.04
-127.04
tBu
tBu
S
OTf
F
OMe
2j
(377 MHz, CDCl3)
f1 (ppm)

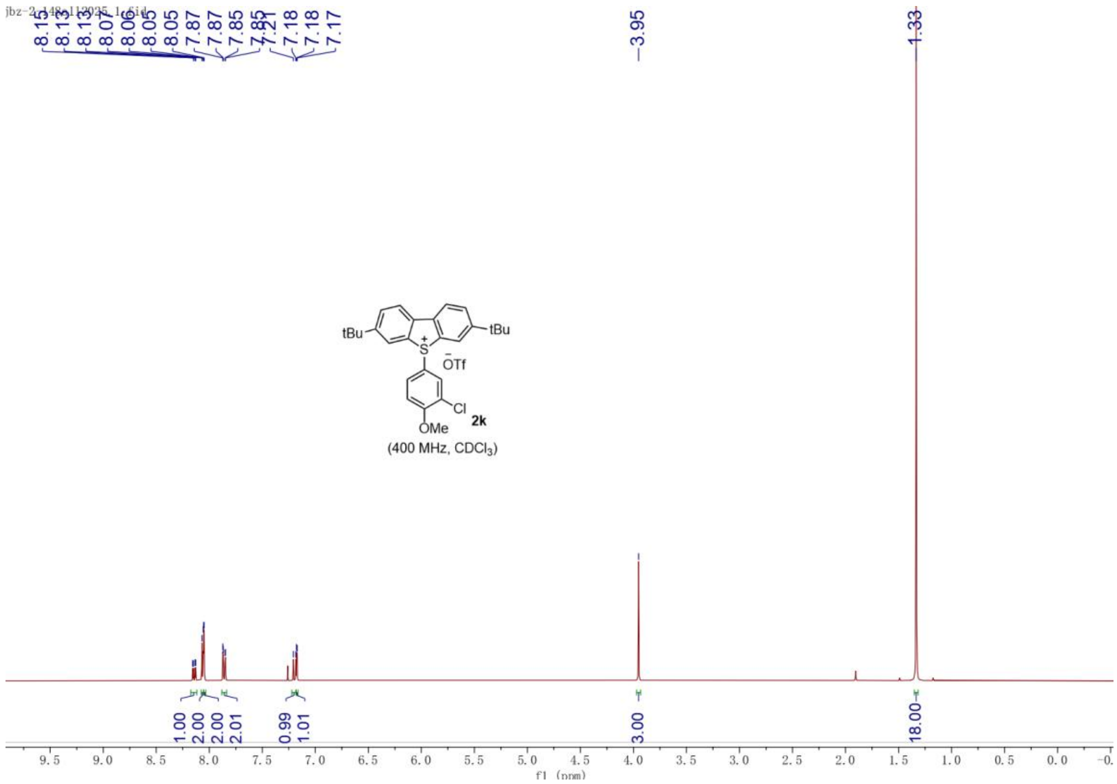
8.13
8.13
8.07
8.06
8.05
7.87
7.87
7.85
7.85
7.21
7.18
7.18
7.17
-3.95
-1.33
tBu
tBu
S
OTf
Cl
OMe
2k
(400 MHz, CDCl3)
1.00
2.00
2.00
2.01
0.99
1.01
3.00
18.00
f1 (ppm)

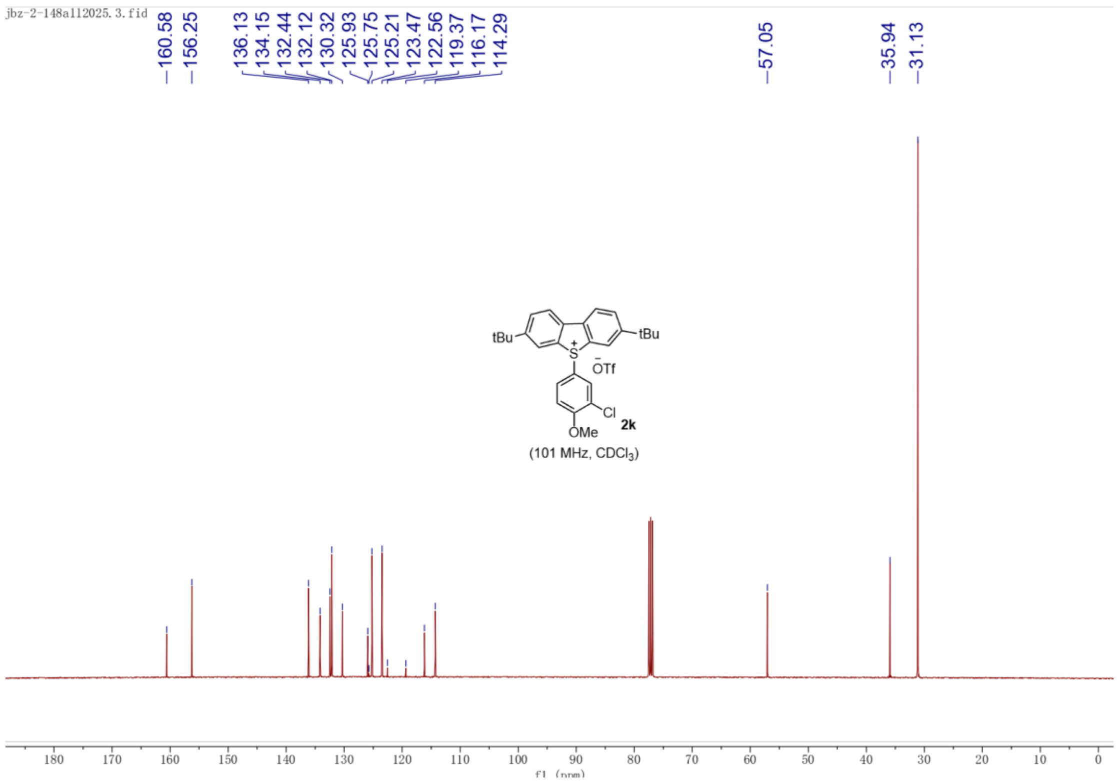

jbz-2-148a112025.3.fid
160.58
156.25
136.13
134.15
132.44
132.12
130.32
125.93
125.75
125.21
123.47
122.56
119.37
116.17
114.29
57.05
35.94
31.13
tBu
tBu
S
OTf
Cl
OMe
2k
(101 MHz, CDCl3)
f1 (ppm)


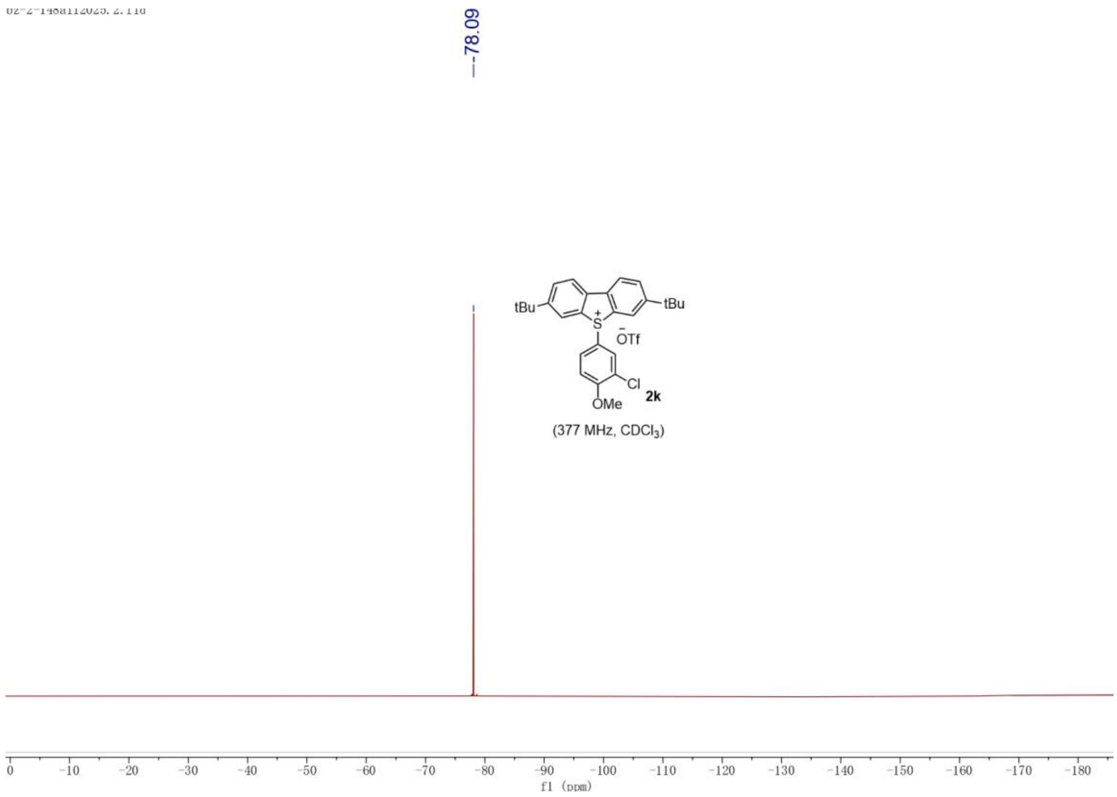

-78.09
tBu
tBu
S
OTf
Cl
OMe
2k
(377 MHz, CDCl3)
f1 (ppm)

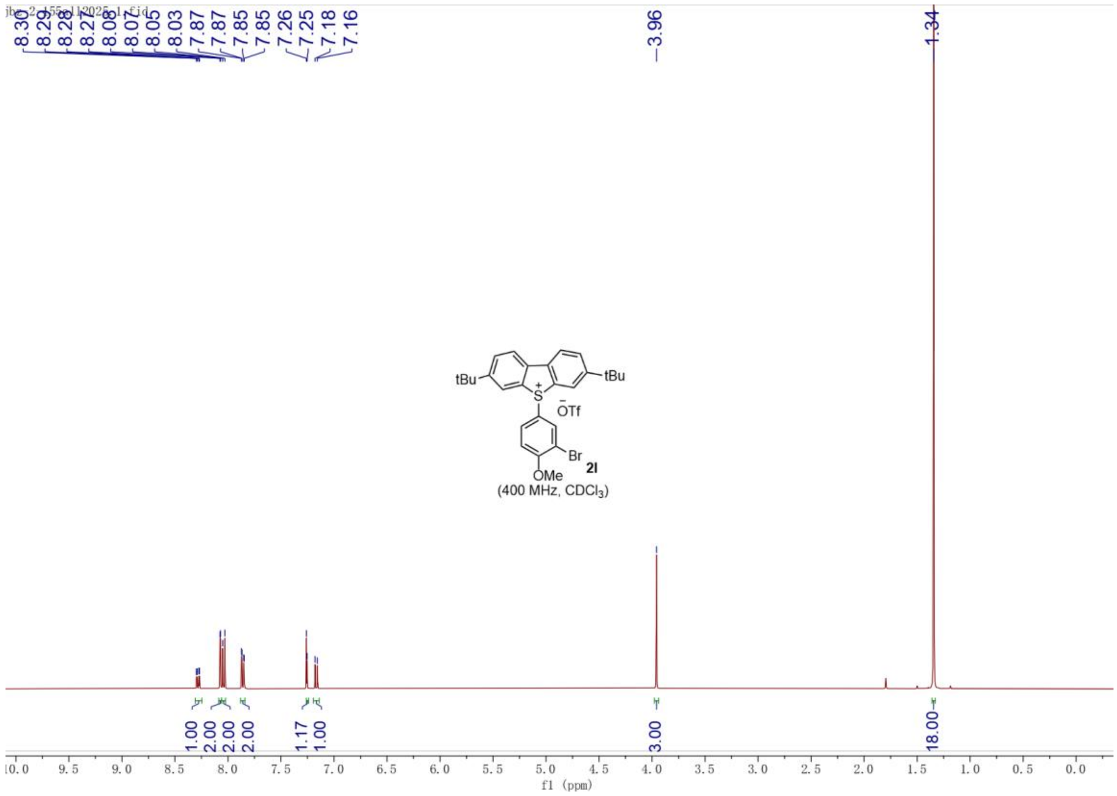

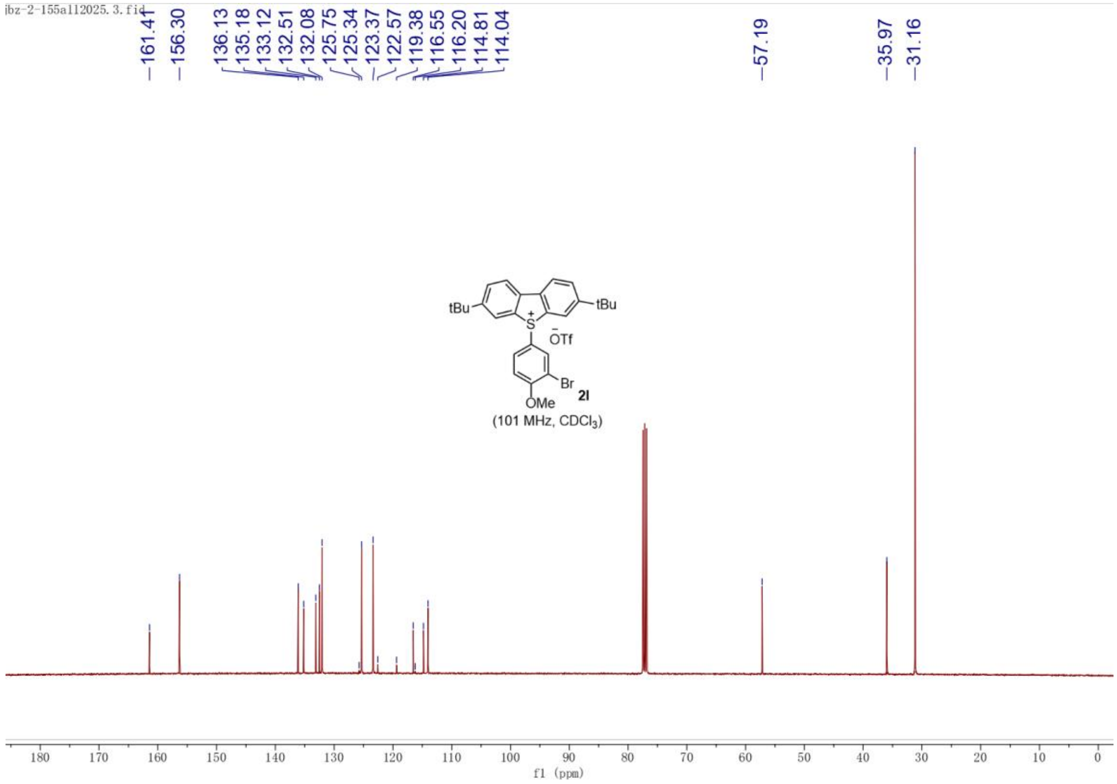

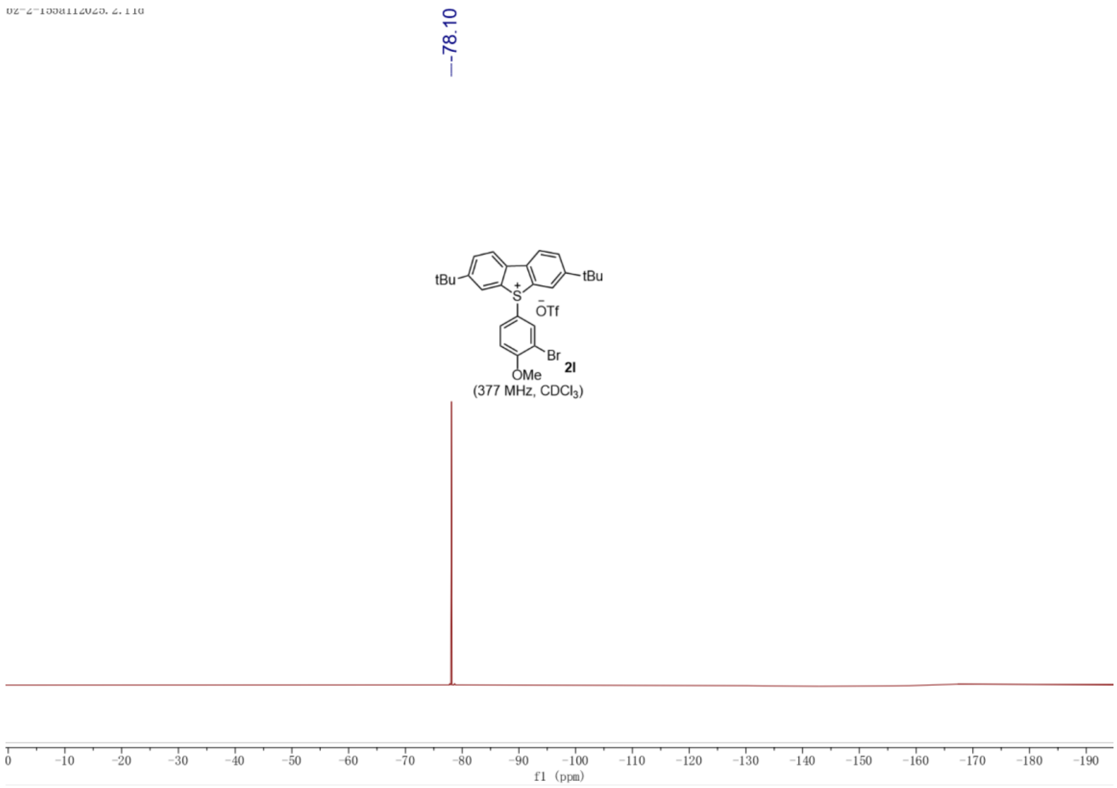

-78.10
tBu
tBu
S
OTf
Br
OMe
2l
(377 MHz, CDCl3)
f1 (ppm)


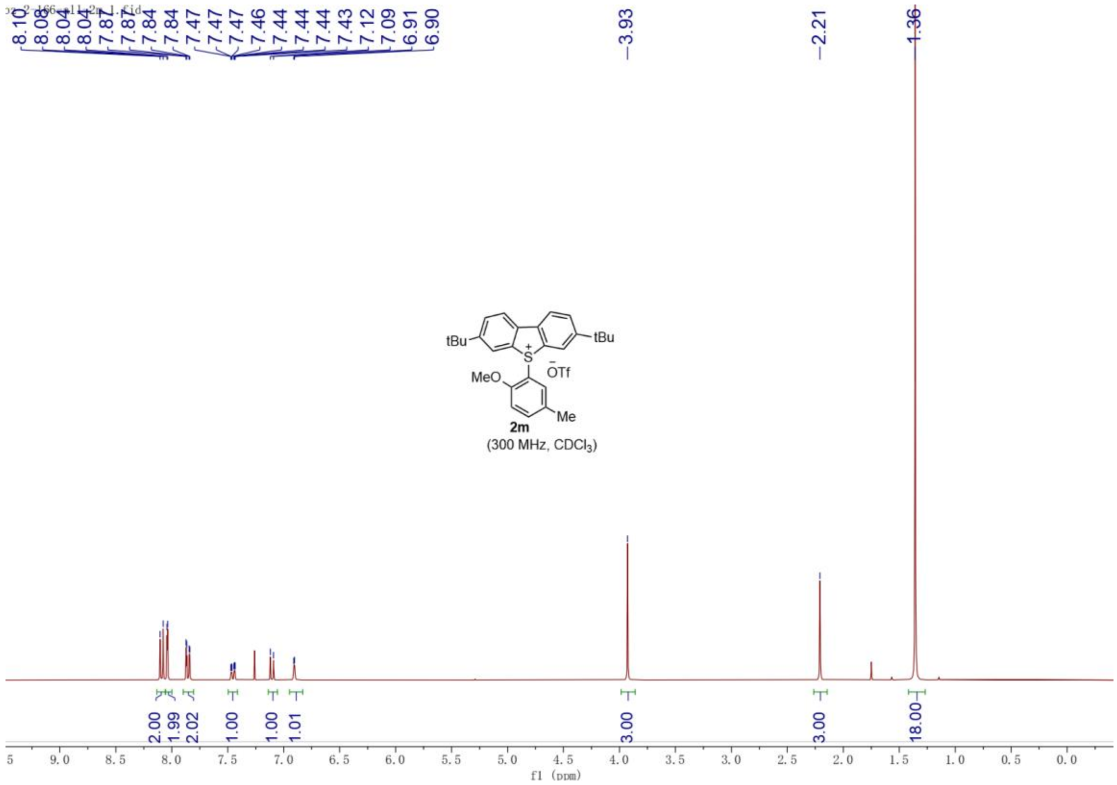

8.10
8.08
8.04
8.04
7.87
7.87
7.84
7.84
7.47
7.47
7.47
7.46
7.44
7.44
7.44
7.43
7.12
7.09
6.91
6.90
3.93
2.21
1.36
tBu
tBu
S
OTf
MeO
Me
2m
(300 MHz, CDCl3)
2.00
1.99
2.02
1.00
1.00
1.01
3.00
3.00
18.00
f1 (ppm)

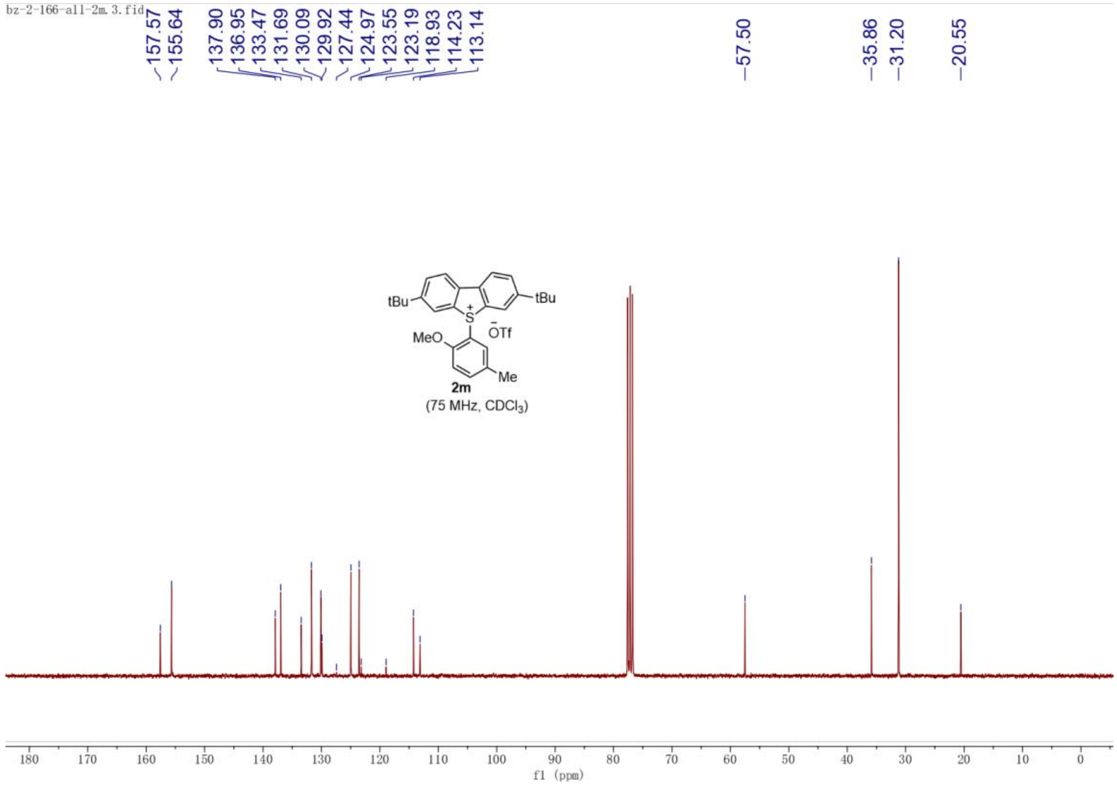
bz-2-166-all-2m.3.fid
157.57
155.64
137.90
136.95
133.47
131.69
130.09
129.92
127.44
124.97
123.55
123.19
118.93
114.23
113.14
57.50
35.86
31.20
20.55
tBu
tBu
MeO
OTf
Me
2m
(75 MHz, CDCl3)
f1 (ppm)

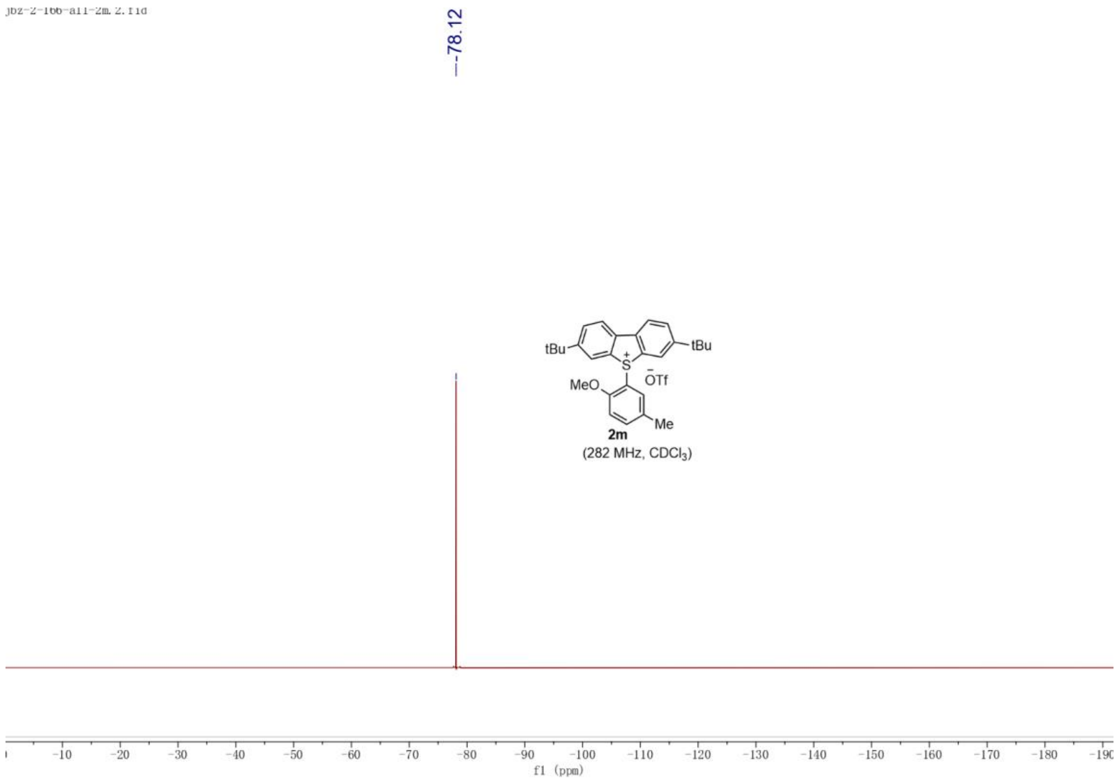
jbz-2-166-all-2m.2.fid
-78.12
tBu
tBu
MeO
OTf
Me
2m
(282 MHz, CDCl3)
f1 (ppm)

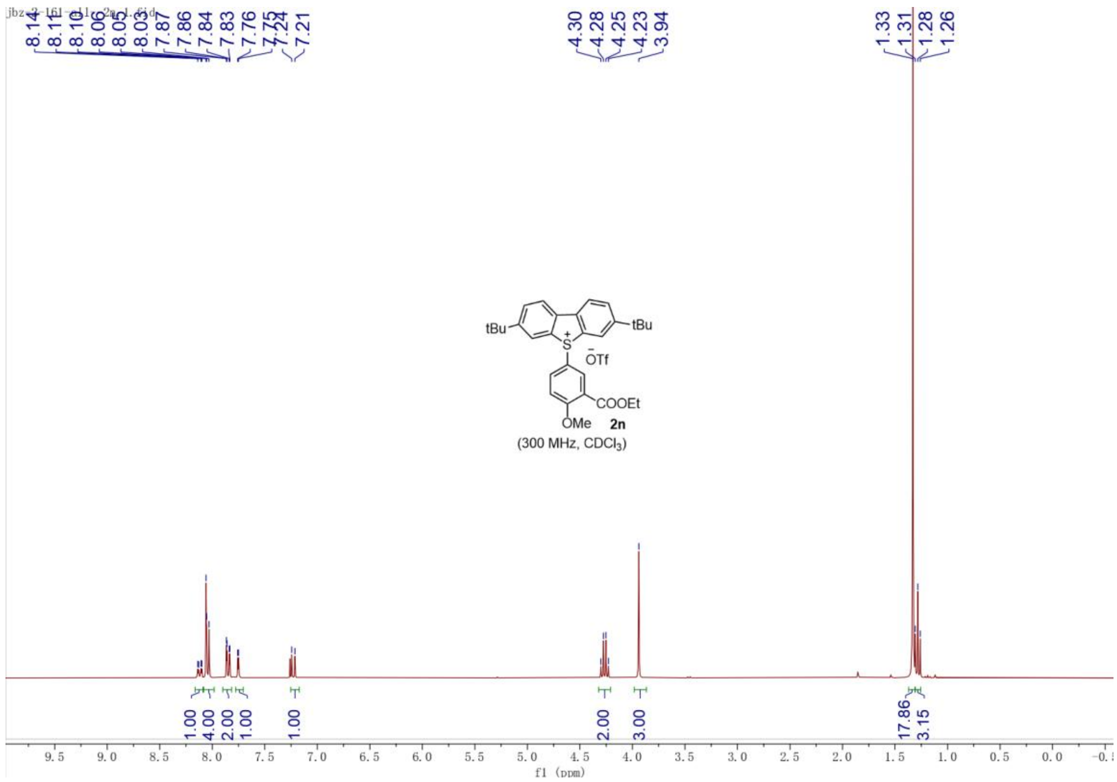
jbz-2-161-all--2n-1.fid
8.14
8.11
8.10
8.06
8.05
8.03
7.87
7.86
7.84
7.83
7.76
7.75
7.24
7.21
4.30
4.28
4.25
4.23
3.94
1.33
1.31
1.28
1.26
tBu
tBu
S
OTf
COOEt
OMe
2n
(300 MHz, CDCl3)
1.00
4.00
2.00
1.00
1.00
2.00
3.00
17.86
3.15
f1 (ppm)

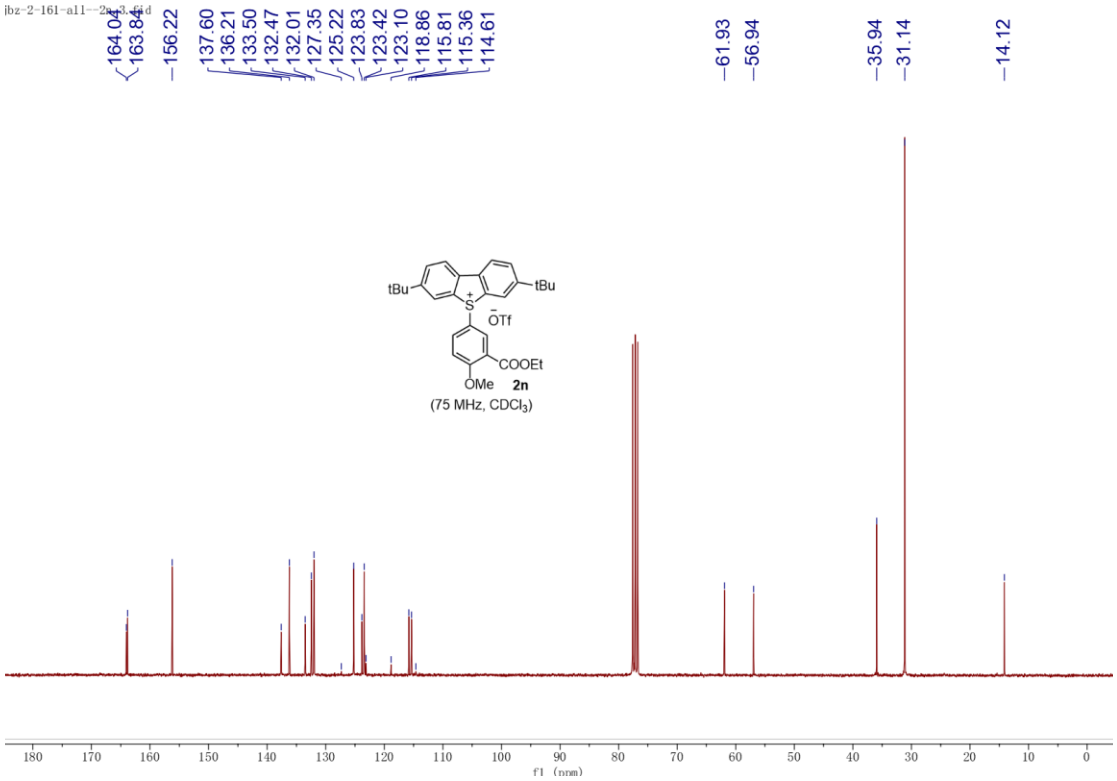
jbz-2-161-all--2n-3.fid
164.04
163.84
156.22
137.60
136.21
133.50
132.47
132.01
127.35
125.22
123.83
123.42
123.10
118.86
115.81
115.36
114.61
61.93
56.94
35.94
31.14
14.12
tBu
tBu
S
OTf
COOEt
OMe
2n
(75 MHz, CDCl3)
f1 (ppm)

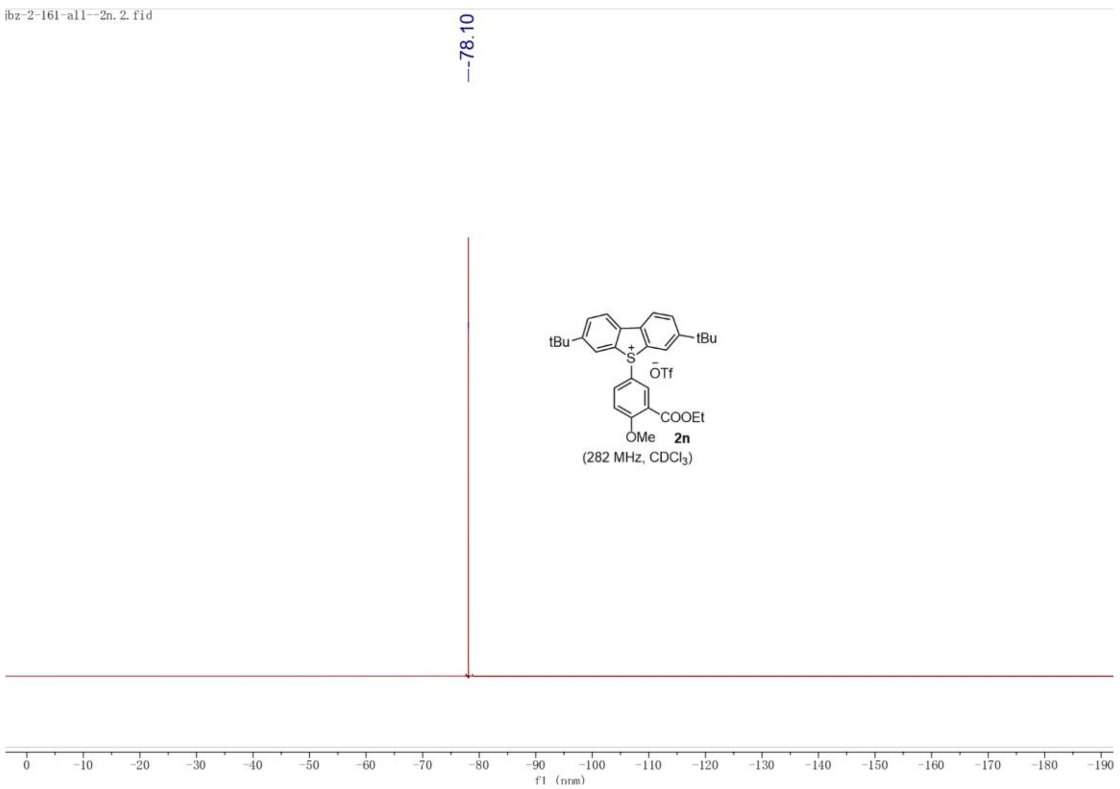
jbz-2-161-all--2n.2.fid
-78.10
tBu
tBu
S+
OTf
COOEt
OMe
2n
(282 MHz, CDCl3)
0 -10 -20 -30 -40 -50 -60 -70 -80 -90 -100 -110 -120 -130 -140 -150 -160 -170 -180 -190
f1 (ppm)

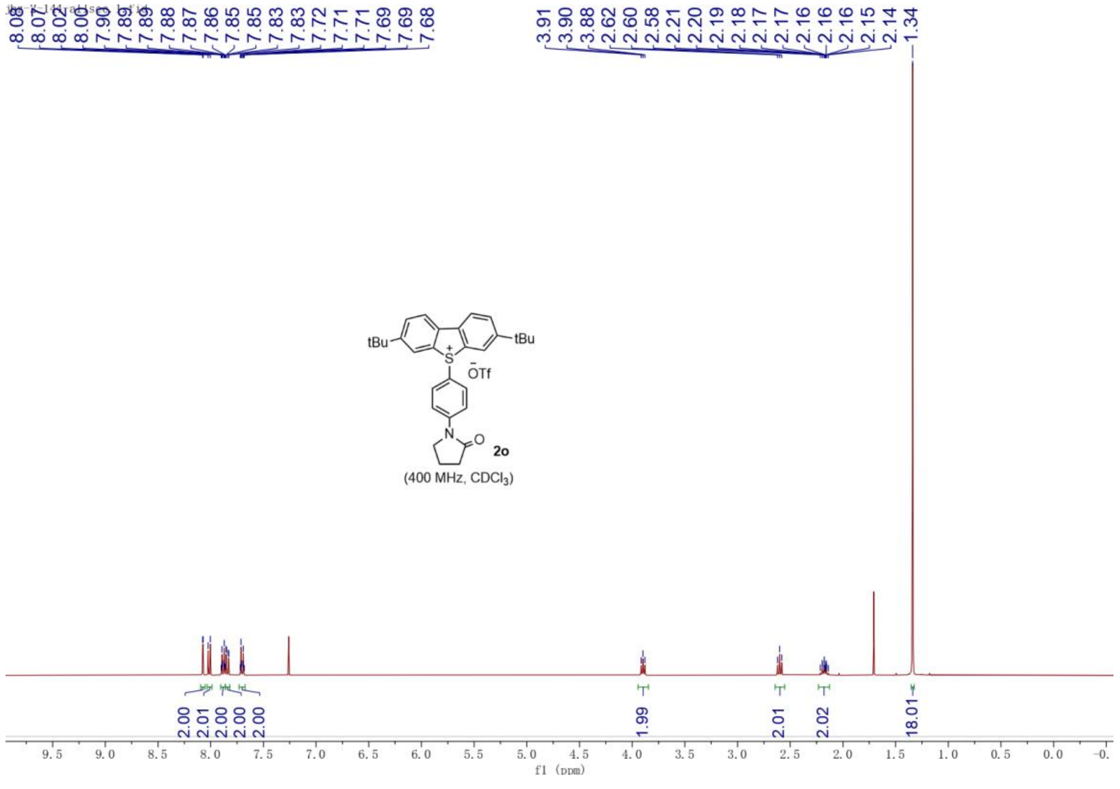
8.08 8.07 8.02 8.00 7.90 7.89 7.89 7.88 7.87 7.86 7.85 7.85 7.83 7.83 7.72 7.71 7.69 7.69 7.68
3.91 3.90 3.88 2.62 2.60 2.58 2.21 2.20 2.19 2.18 2.17 2.17 2.16 2.16 2.16 2.15 2.14 1.34
tBu
tBu
S+
OTf
N
O
2o
(400 MHz, CDCl3)
2.00 2.01 2.00 2.00 2.00 1.99 2.01 2.02 18.01
9.5 9.0 8.5 8.0 7.5 7.0 6.5 6.0 5.5 5.0 4.5 4.0 3.5 3.0 2.5 2.0 1.5 1.0 0.5 0.0 -0.
f1 (ppm)

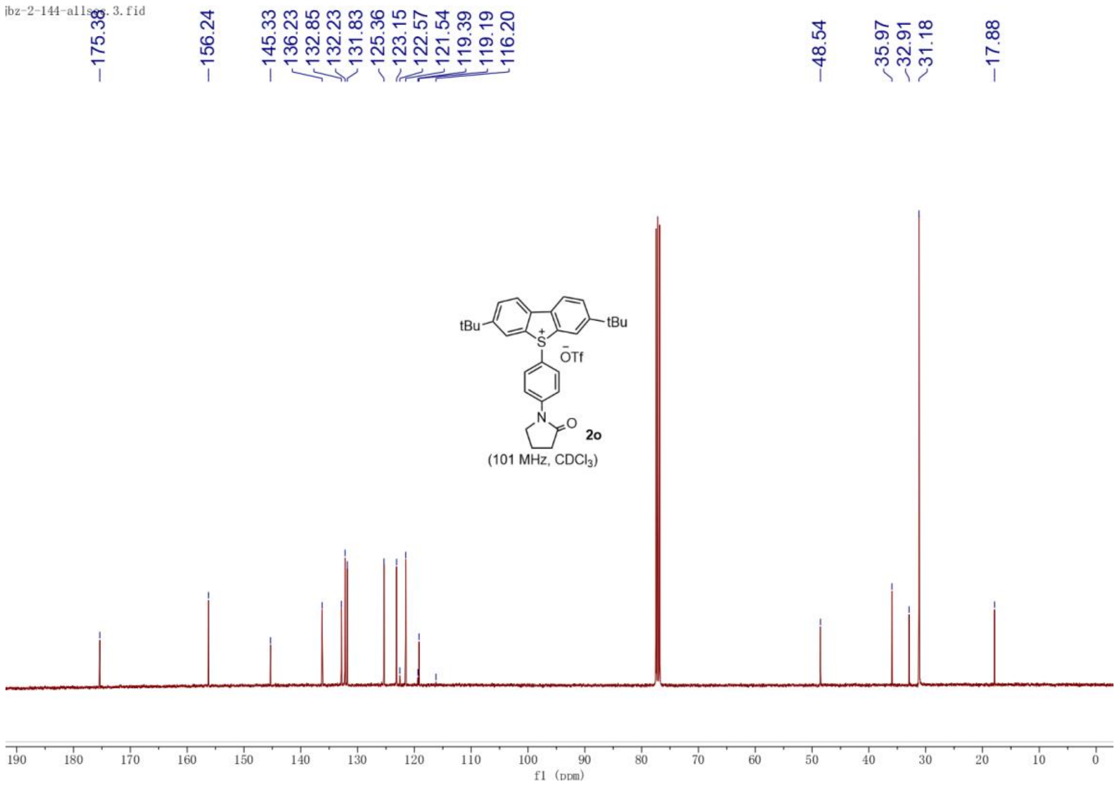
jbz-2-144-allsec.3.fid
175.38
156.24
145.33
136.23
132.85
132.23
131.83
125.36
123.15
122.57
121.54
119.39
119.19
116.20
48.54
35.97
32.91
31.18
17.88
tBu
tBu
S
OTf
N
O
2o
(101 MHz, CDCl3)
f1 (ppm)

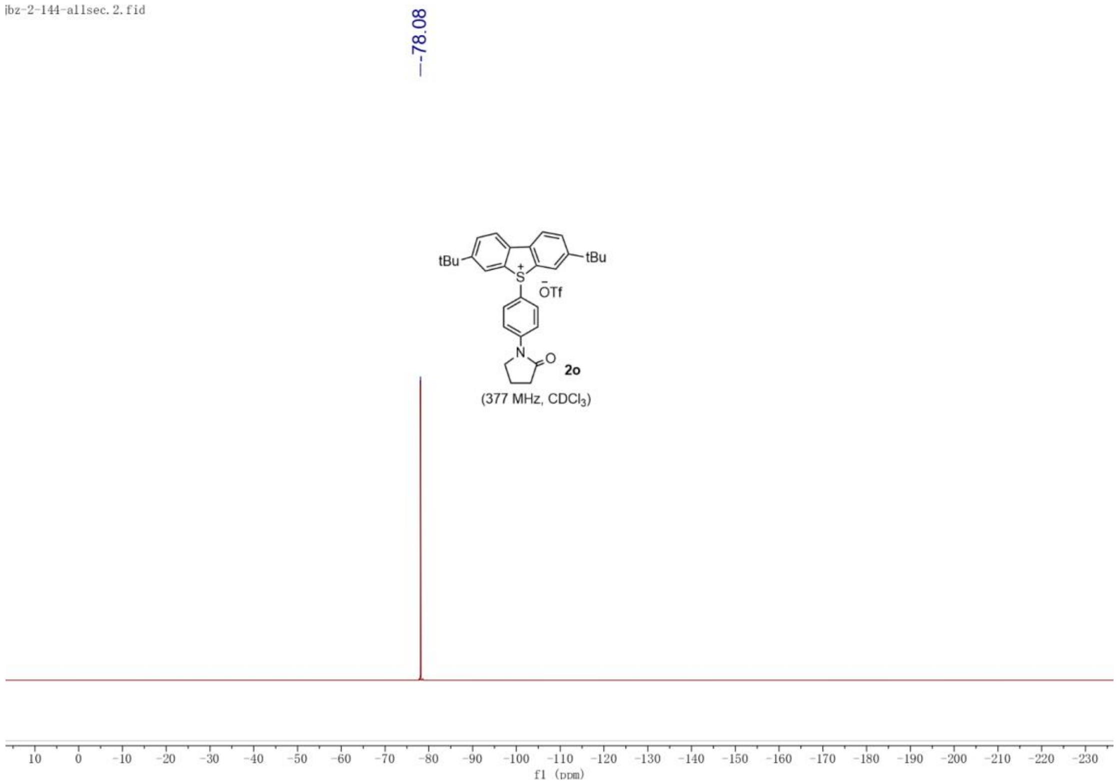
jbz-2-144-allsec.2.fid
-78.08
tBu
tBu
S
OTf
N
O
2o
(377 MHz, CDCl3)
f1 (ppm)

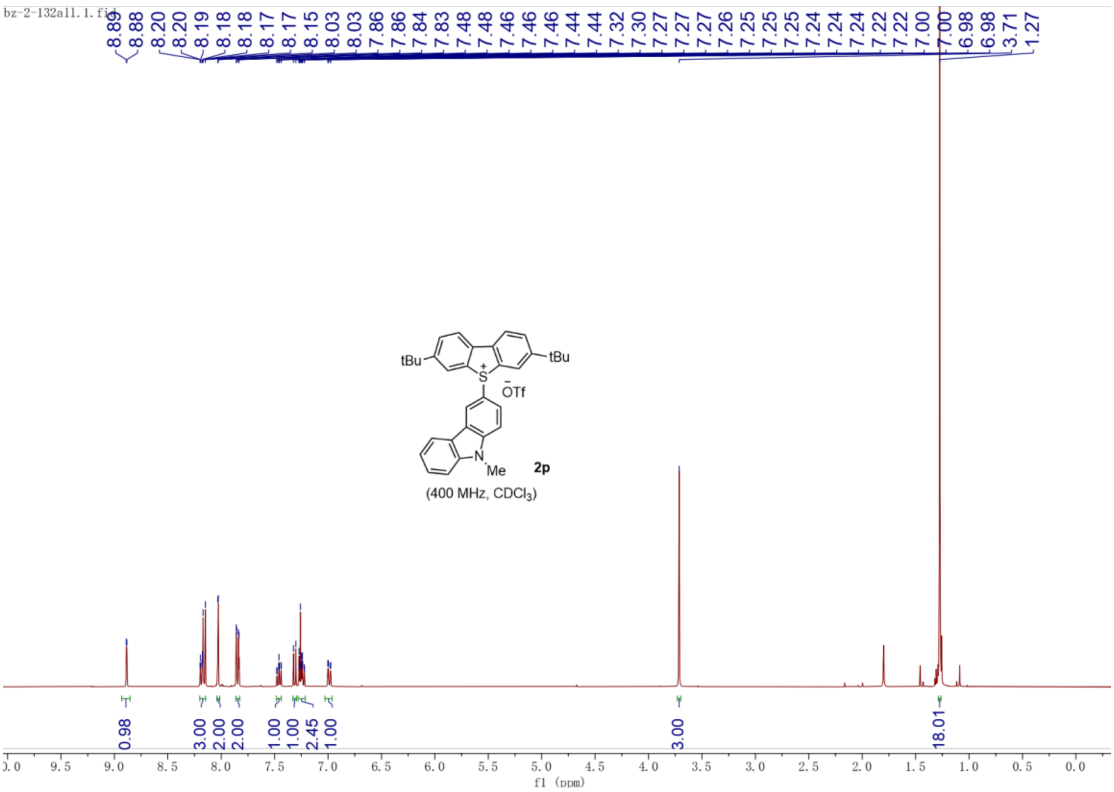

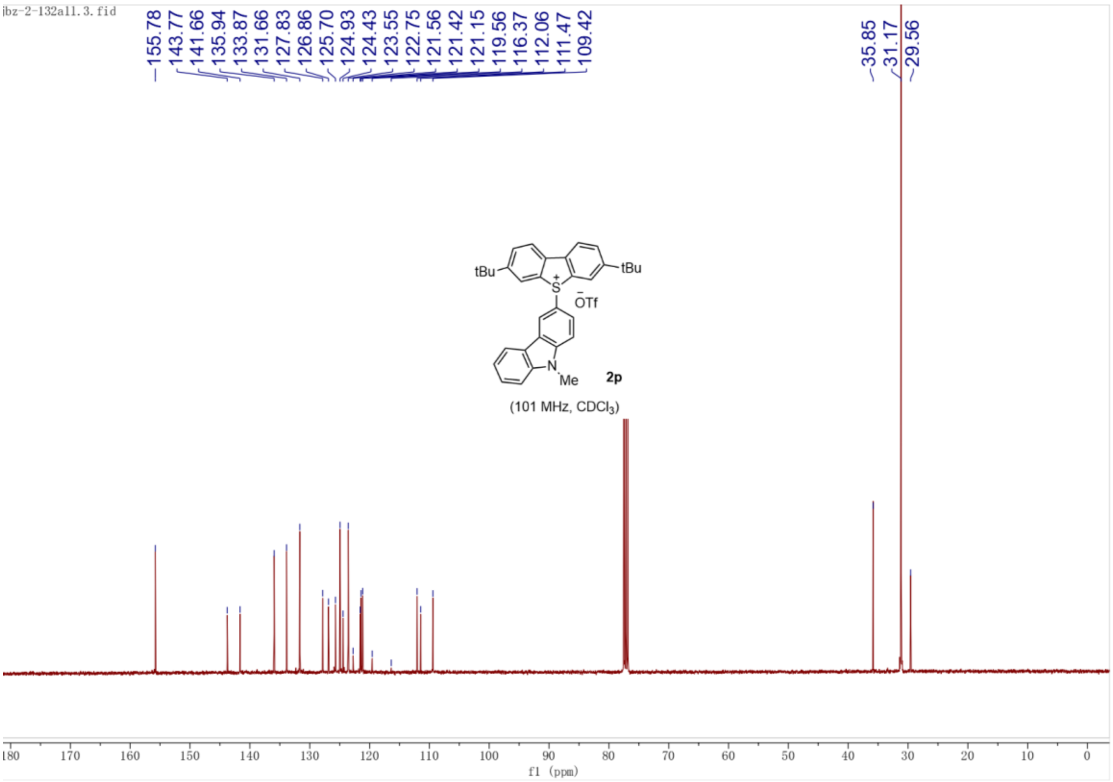

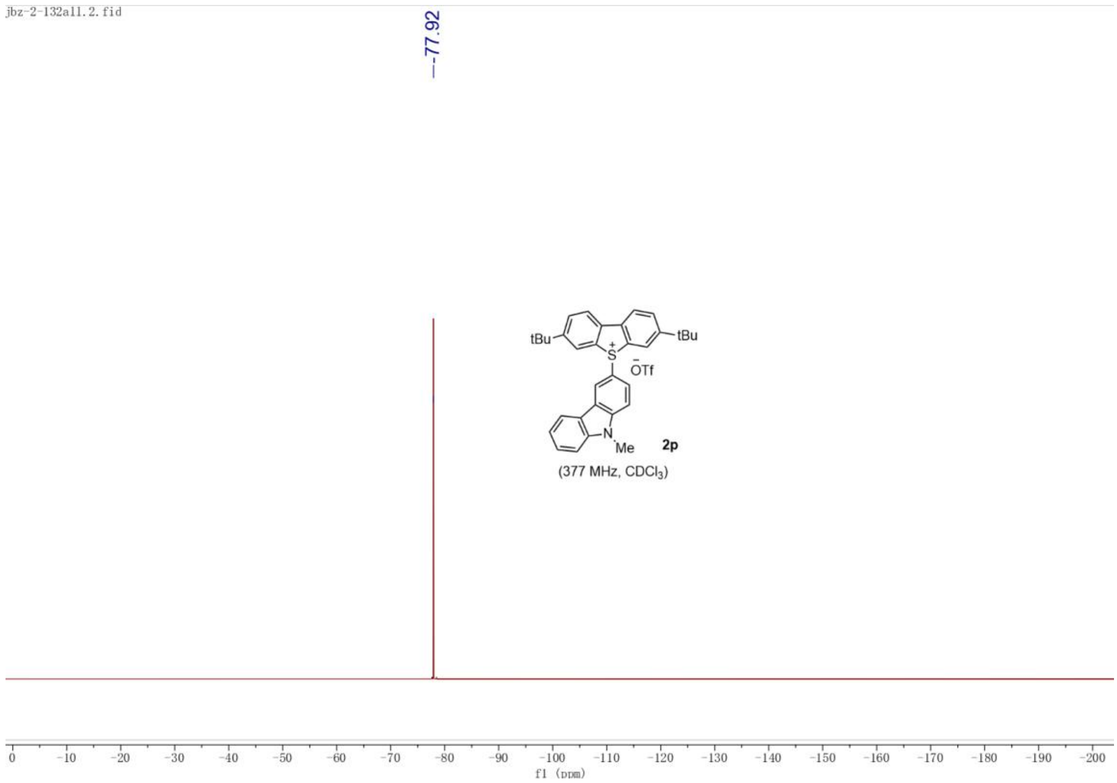
jbz-2-132all.2.fid
-77.92
tBu
tBu
S
OTf
N
Me
2p
(377 MHz, CDCl3)
0 −10 −20 −30 −40 −50 −60 −70 −80 −90 −100 −110 −120 −130 −140 −150 −160 −170 −180 −190 −200
f1 (ppm)

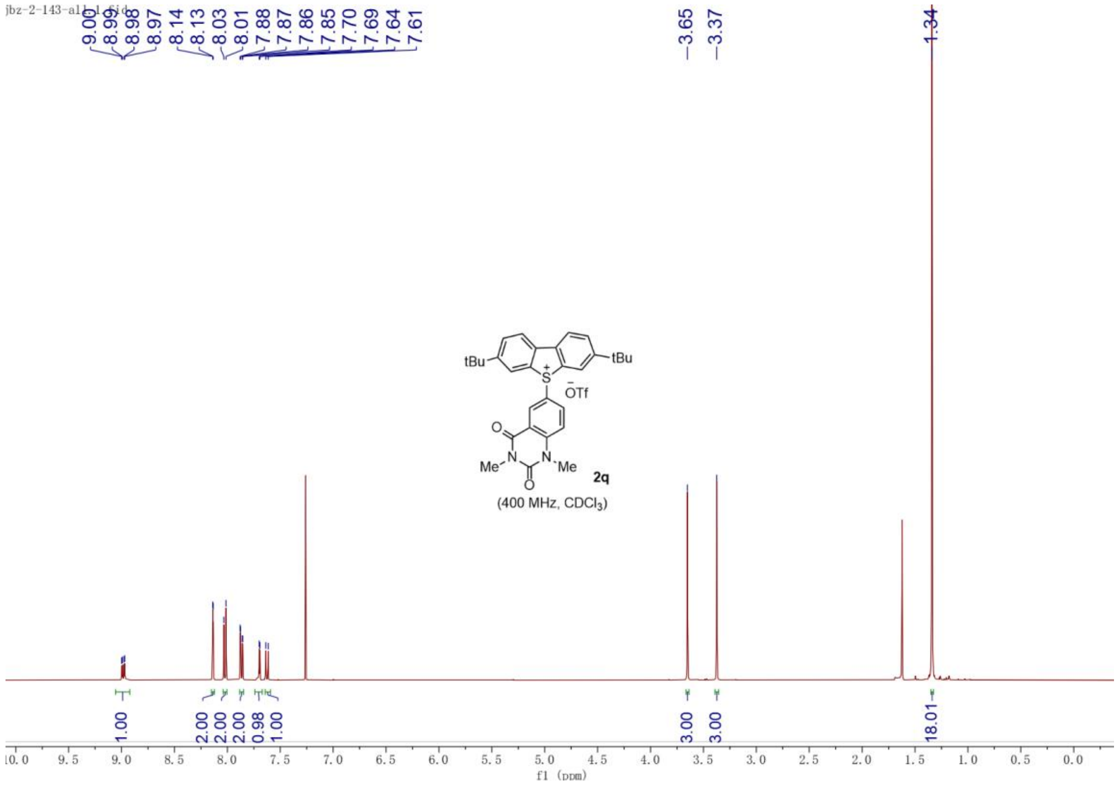
jbz-2-143-all.1.fid
9.00 8.99 8.98 8.97 8.14 8.13 8.03 8.01 7.88 7.87 7.86 7.85 7.70 7.69 7.64 7.61
3.65
3.37
1.34
tBu
tBu
S
OTf
O
Me
N
N
Me
O
2q
(400 MHz, CDCl3)
1.00
2.00 2.00 2.00 0.98 1.00
3.00
3.00
18.01
10.0 9.5 9.0 8.5 8.0 7.5 7.0 6.5 6.0 5.5 5.0 4.5 4.0 3.5 3.0 2.5 2.0 1.5 1.0 0.5 0.0
f1 (ppm)

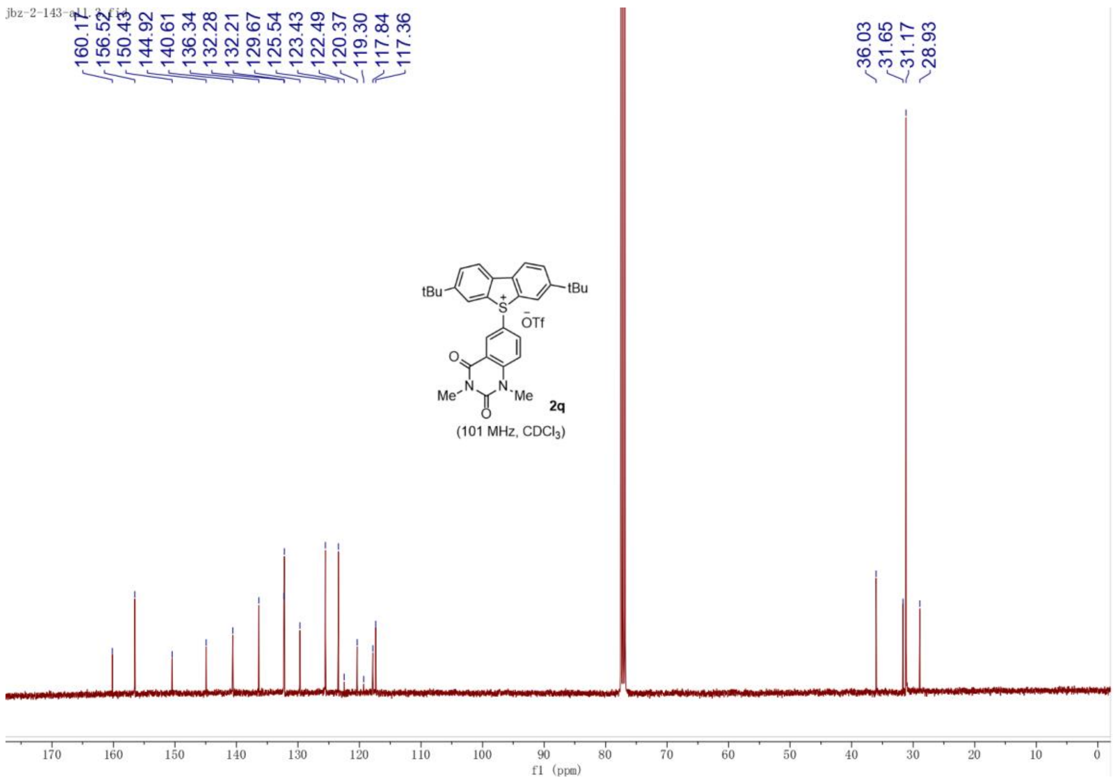

jbz-2-143-all.3.fid
160.17
156.52
150.43
144.92
140.61
136.34
132.28
132.21
129.67
125.54
123.43
122.49
120.37
119.30
117.84
117.36
36.03
31.65
31.17
28.93
tBu
tBu
S
OTf
O
N
N
Me
Me
O
2q
(101 MHz, $CDCl_3$)
170 160 150 140 130 120 110 100 90 80 70 60 50 40 30 20 10 0
f1 (ppm)


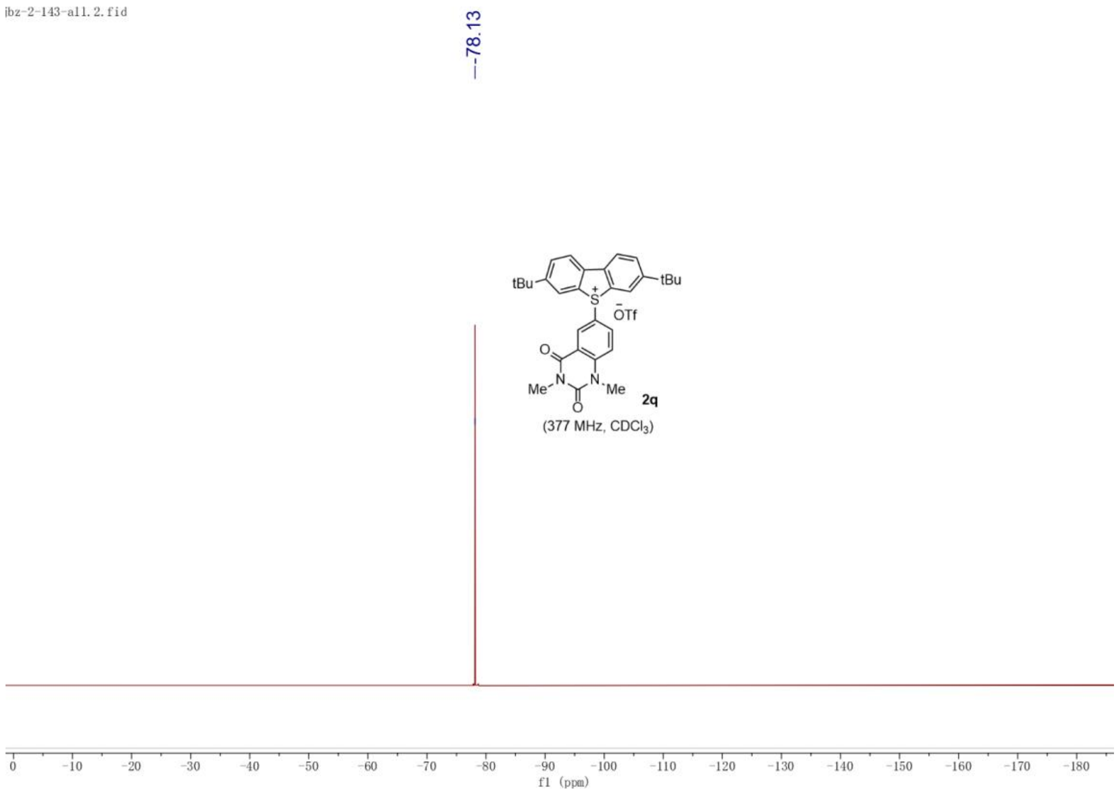

jbz-2-143-all.2.fid
-78.13
tBu
tBu
S
OTf
O
N
N
Me
Me
O
2q
(377 MHz, $CDCl_3$)
0 -10 -20 -30 -40 -50 -60 -70 -80 -90 -100 -110 -120 -130 -140 -150 -160 -170 -180
f1 (ppm)

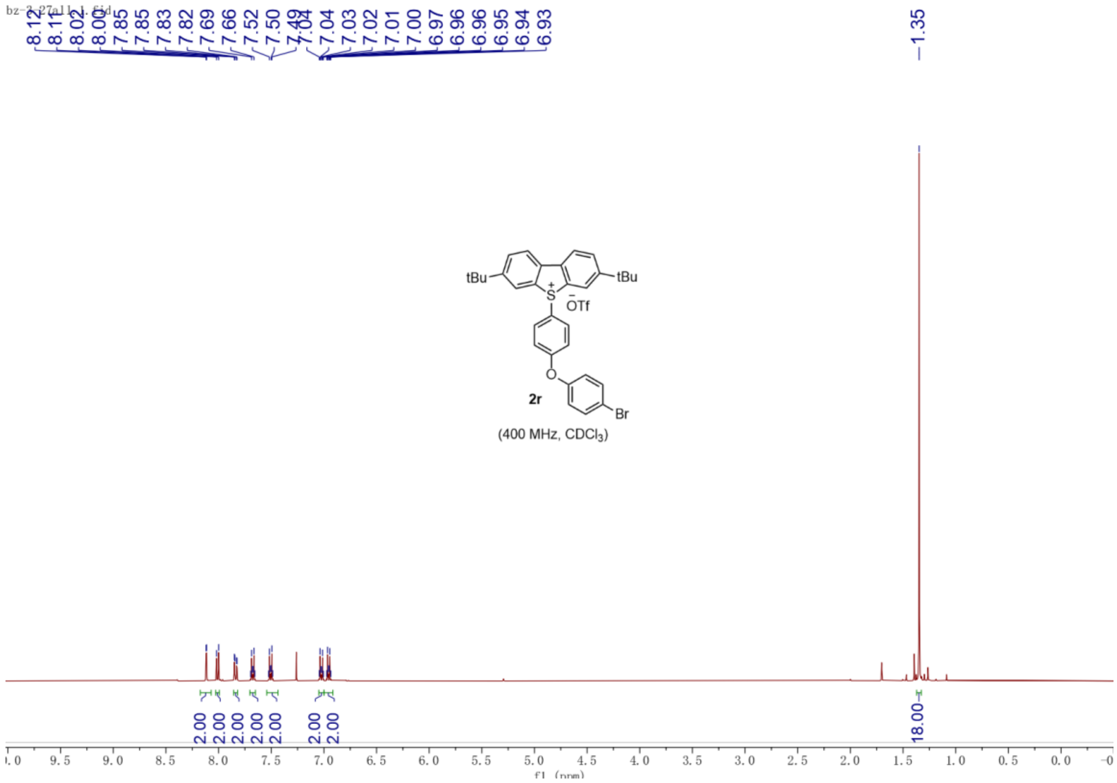
bz-3-27a11.1.fid
8.12
8.11
8.02
8.00
7.85
7.85
7.83
7.82
7.69
7.66
7.52
7.50
7.49
7.04
7.04
7.03
7.02
7.01
7.00
6.97
6.96
6.96
6.95
6.94
6.93
1.35
tBu
tBu
S
OTf
O
2r
Br
(400 MHz, CDCl3)
2.00
2.00
2.00
2.00
2.00
2.00
2.00
18.00
f1 (ppm)

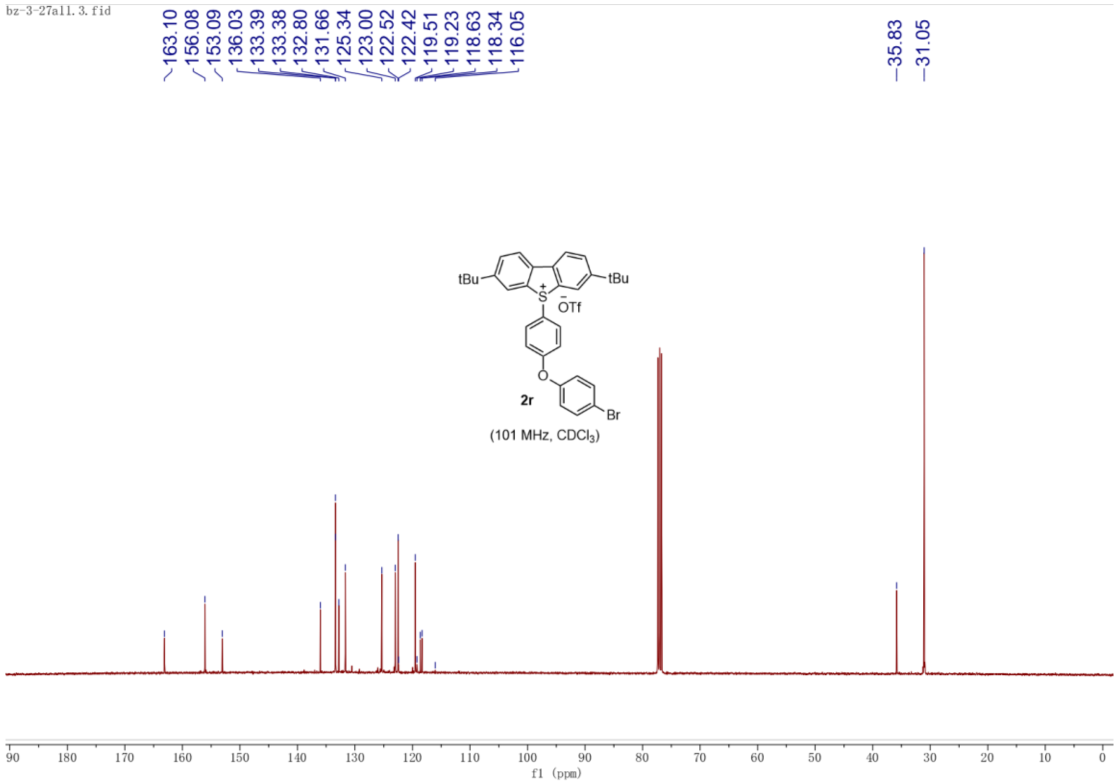
bz-3-27a11.3.fid
163.10
156.08
153.09
136.03
133.39
133.38
132.80
131.66
125.34
123.00
122.52
122.42
119.51
119.23
118.63
118.34
116.05
35.83
31.05
tBu
tBu
S
OTf
O
2r
Br
(101 MHz, CDCl3)
f1 (ppm)

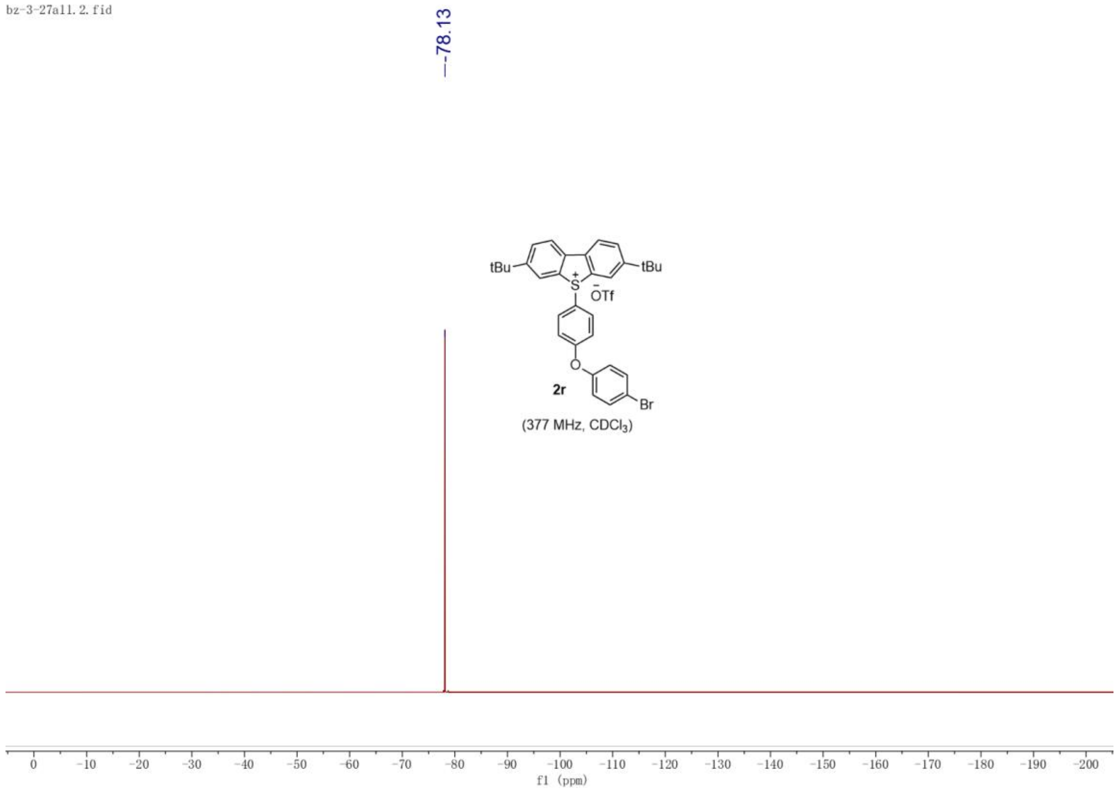
bz-3-27all.2.fid
-78.13
tBu
tBu
S
OTf
O
Br
2r
(377 MHz, CDCl$_3$)
f1 (ppm)

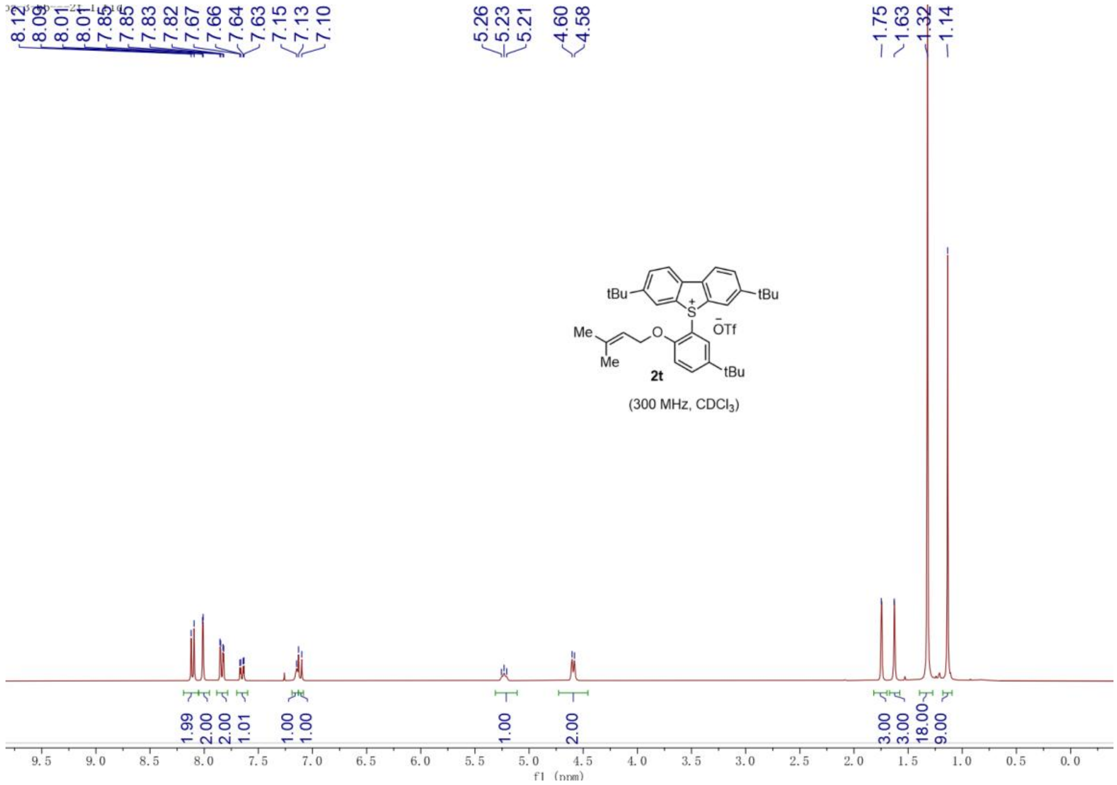
8.12 8.09 8.01 8.01 7.85 7.85 7.83 7.82 7.67 7.66 7.64 7.63 7.15 7.13 7.10
5.26 5.23 5.21 4.60 4.58
1.75 1.63 1.32 1.14
tBu
tBu
S
OTf
Me
Me
O
tBu
2t
(300 MHz, CDCl$_3$)
1.99 2.00 2.00 1.01 1.00 1.00 1.00 2.00 3.00 3.00 18.00 9.00
f1 (ppm)

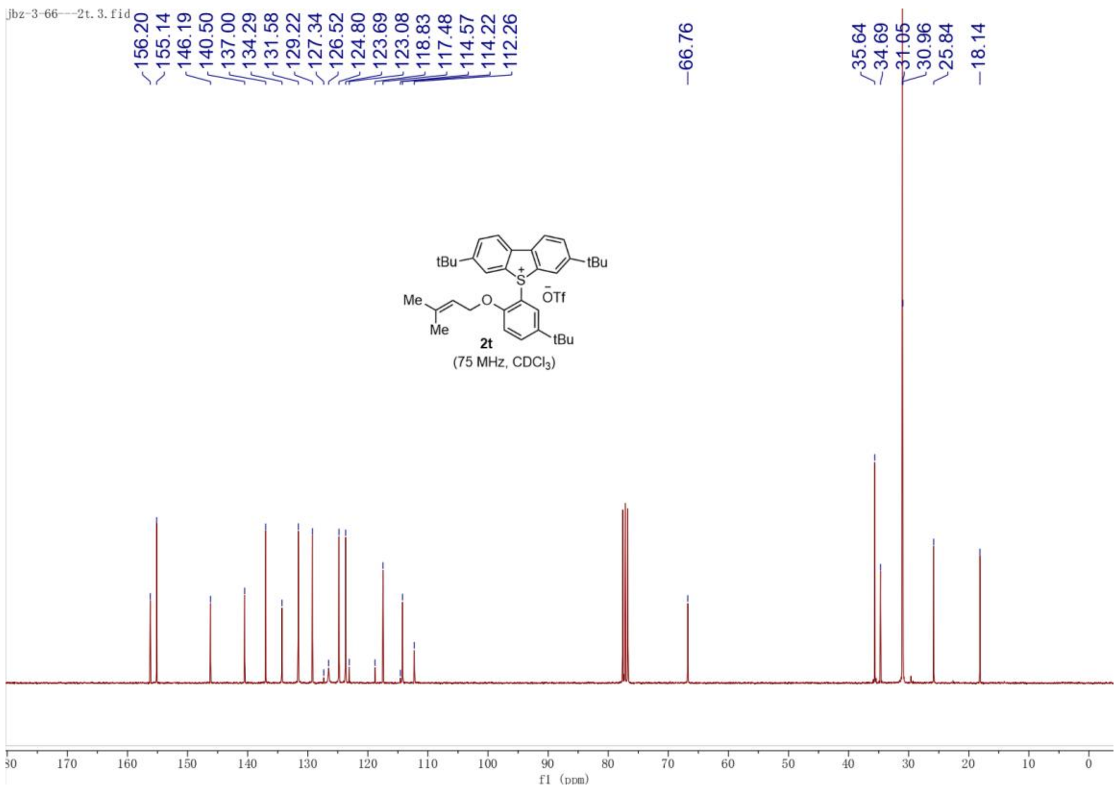
jbz-3-66---2t.3.fid
156.20
155.14
146.19
140.50
137.00
134.29
131.58
129.22
127.34
126.52
124.80
123.69
123.08
118.83
117.48
114.57
114.22
112.26
66.76
35.64
34.69
31.05
30.96
25.84
18.14
tBu
tBu
OTf
Me
Me
O
S
tBu
2t
(75 MHz, CDCl3)
f1 (ppm)

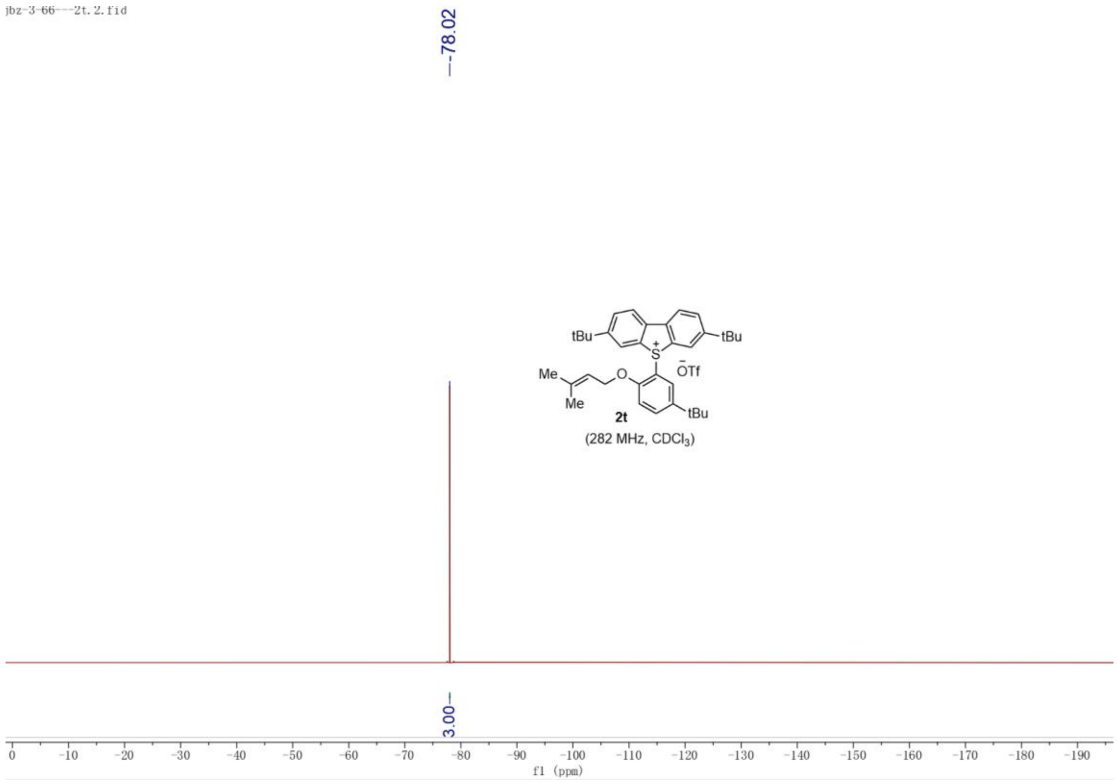
jbz-3-66---2t.2.fid
-78.02
tBu
tBu
OTf
Me
Me
O
S
tBu
2t
(282 MHz, CDCl3)
3.00
f1 (ppm)

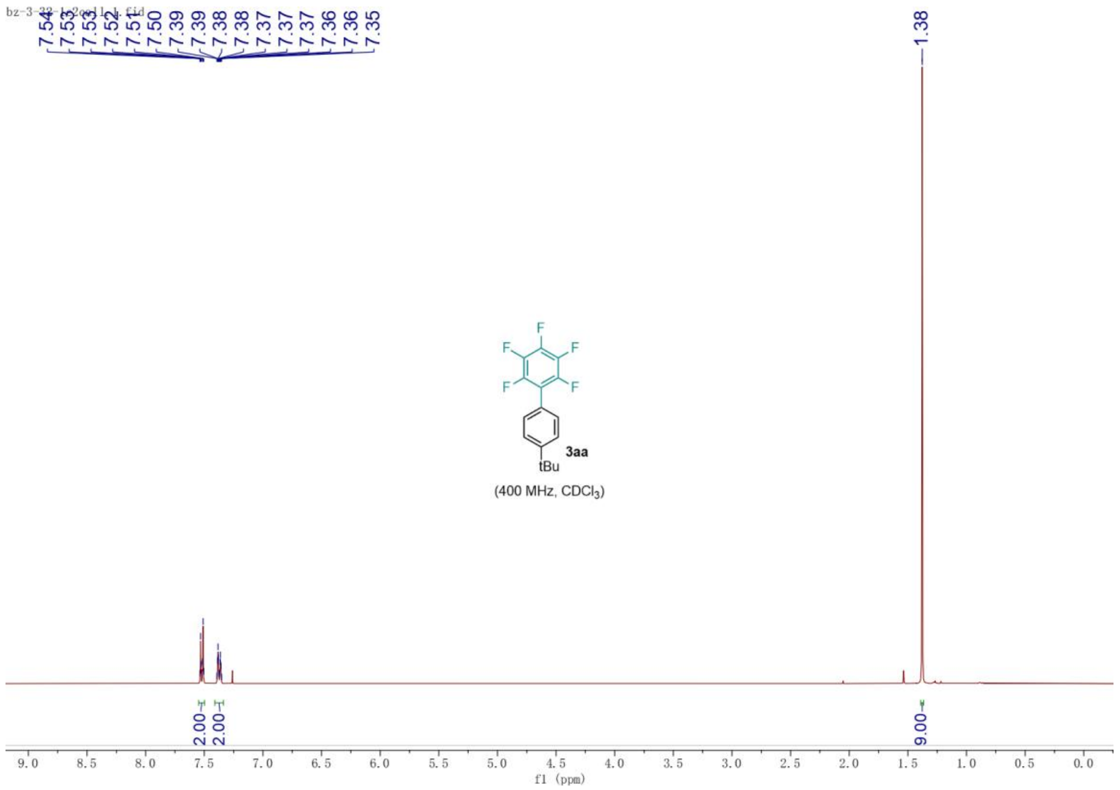
bz-3-32-1c2call.1.fid
7.54
7.53
7.53
7.52
7.51
7.50
7.39
7.39
7.38
7.38
7.37
7.37
7.37
7.36
7.36
7.35
1.38
F
F
F
F
F
tBu
3aa
(400 MHz, CDCl3)
2.00
2.00
9.00
f1 (ppm)

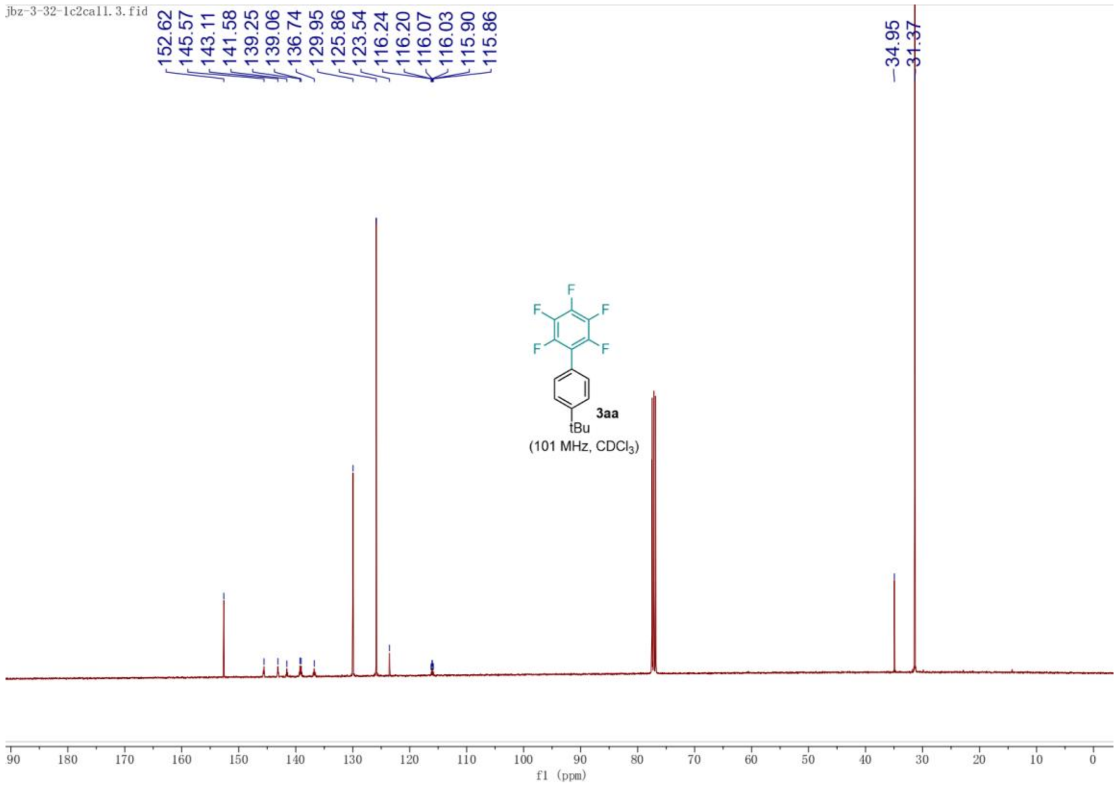
jbz-3-32-1c2call.3.fid
152.62
145.57
143.11
141.58
139.25
139.06
136.74
129.95
125.86
123.54
116.24
116.20
116.07
116.03
115.90
115.86
34.95
31.37
F
F
F
F
F
tBu
3aa
(101 MHz, CDCl3)
f1 (ppm)

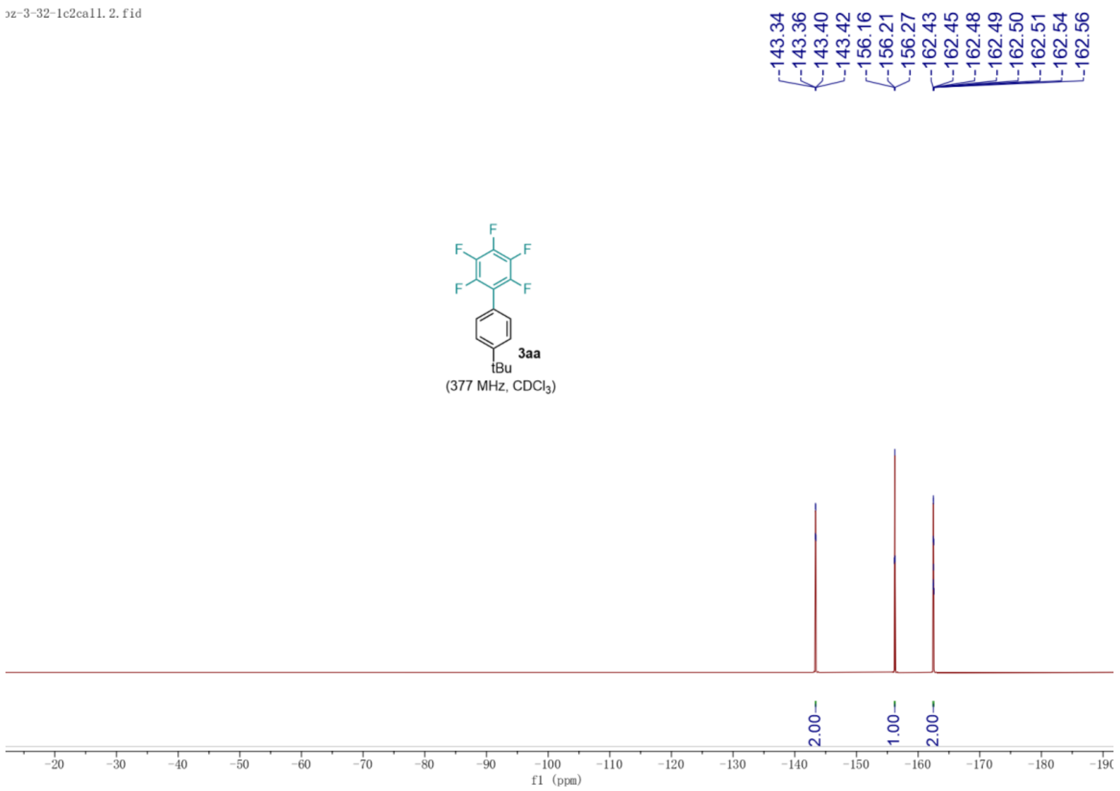

ɔz-3-32-1c2ca11.2.fid
143.34 143.36 143.40 143.42 156.16 156.21 156.27 162.43 162.45 162.48 162.49 162.50 162.51 162.54 162.56
F F F F F
tBu
3aa
(377 MHz, CDCl3)
2.00 1.00 2.00
-20 -30 -40 -50 -60 -70 -80 -90 -100 -110 -120 -130 -140 -150 -160 -170 -180 -190
f1 (ppm)


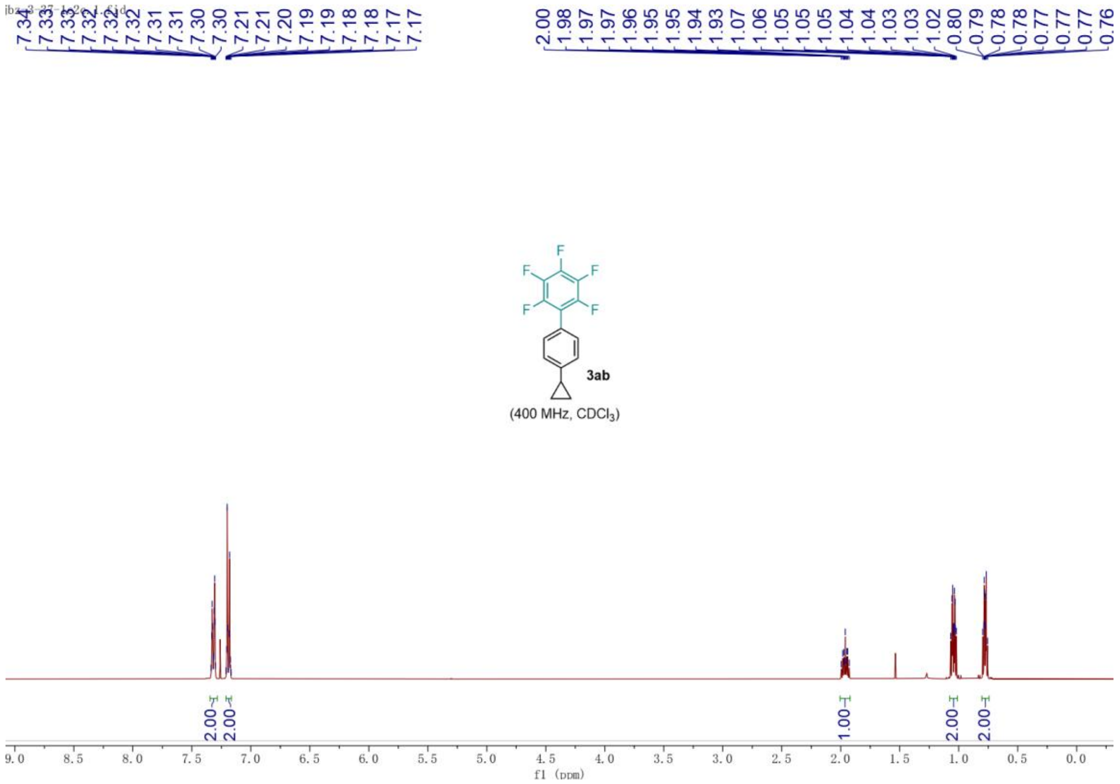

7.34 7.33 7.33 7.32 7.32 7.32 7.31 7.31 7.30 7.30 7.21 7.21 7.20 7.19 7.19 7.18 7.18 7.17 7.17
2.00 1.98 1.97 1.97 1.96 1.95 1.95 1.94 1.93 1.07 1.06 1.05 1.05 1.05 1.04 1.04 1.03 1.03 1.02 0.80 0.79 0.78 0.78 0.77 0.77 0.77 0.76
F F F F F
3ab
(400 MHz, CDCl3)
2.00 2.00 1.00 2.00 2.00
9.0 8.5 8.0 7.5 7.0 6.5 6.0 5.5 5.0 4.5 4.0 3.5 3.0 2.5 2.0 1.5 1.0 0.5 0.0
f1 (ppm)

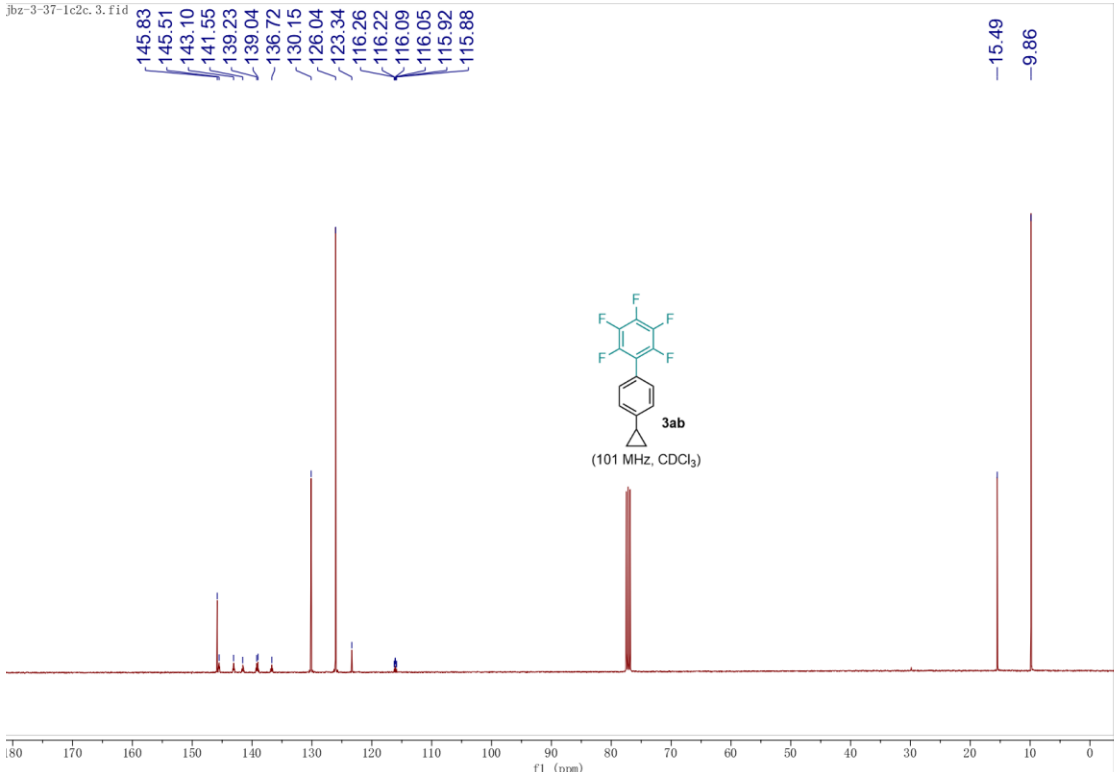

jbz-3-37-1c2c.3.fid
145.83
145.51
143.10
141.55
139.23
139.04
136.72
130.15
126.04
123.34
116.26
116.22
116.09
116.05
115.92
115.88
15.49
9.86
F
3ab
(101 MHz, CDCl3)
180
170
160
150
140
130
120
110
100
90
80
70
60
50
40
30
20
10
0
f1 (ppm)


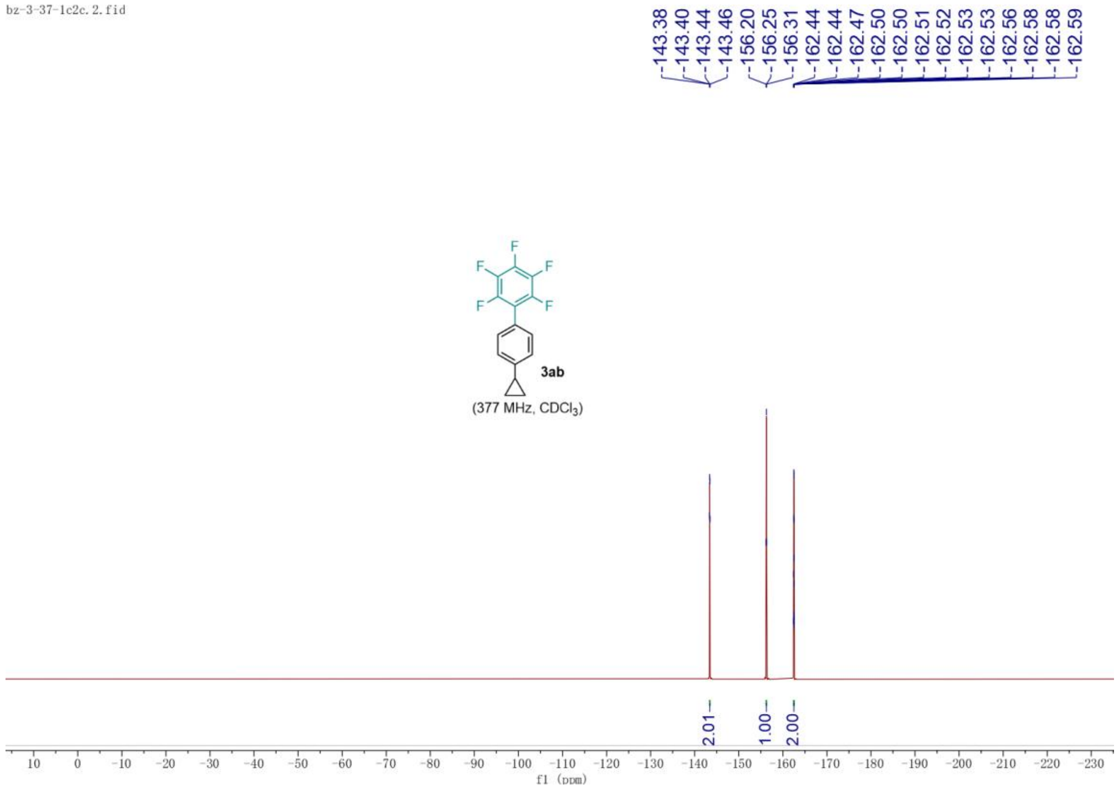

bz-3-37-1c2c.2.fid
-143.38
-143.40
-143.44
-143.46
-156.20
-156.25
-156.31
-162.44
-162.44
-162.47
-162.50
-162.50
-162.51
-162.52
-162.53
-162.53
-162.56
-162.58
-162.58
-162.59
F
3ab
(377 MHz, CDCl3)
2.01
1.00
2.00
10
0
-10
-20
-30
-40
-50
-60
-70
-80
-90
-100
-110
-120
-130
-140
-150
-160
-170
-180
-190
-200
-210
-220
-230
f1 (ppm)

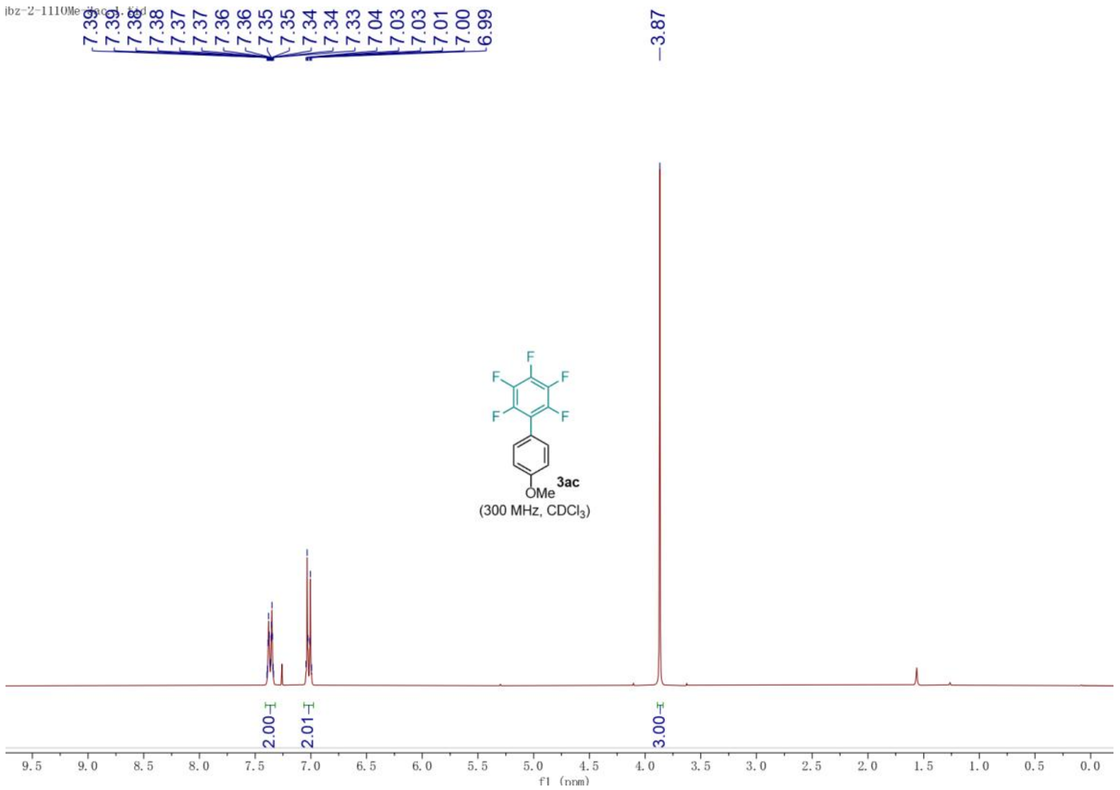
7.39
7.39
7.38
7.38
7.37
7.37
7.36
7.36
7.35
7.35
7.34
7.34
7.33
7.04
7.03
7.03
7.01
7.00
6.99
3.87
3ac
(300 MHz, CDCl$_3$)
2.00
2.01
3.00
f1 (ppm)

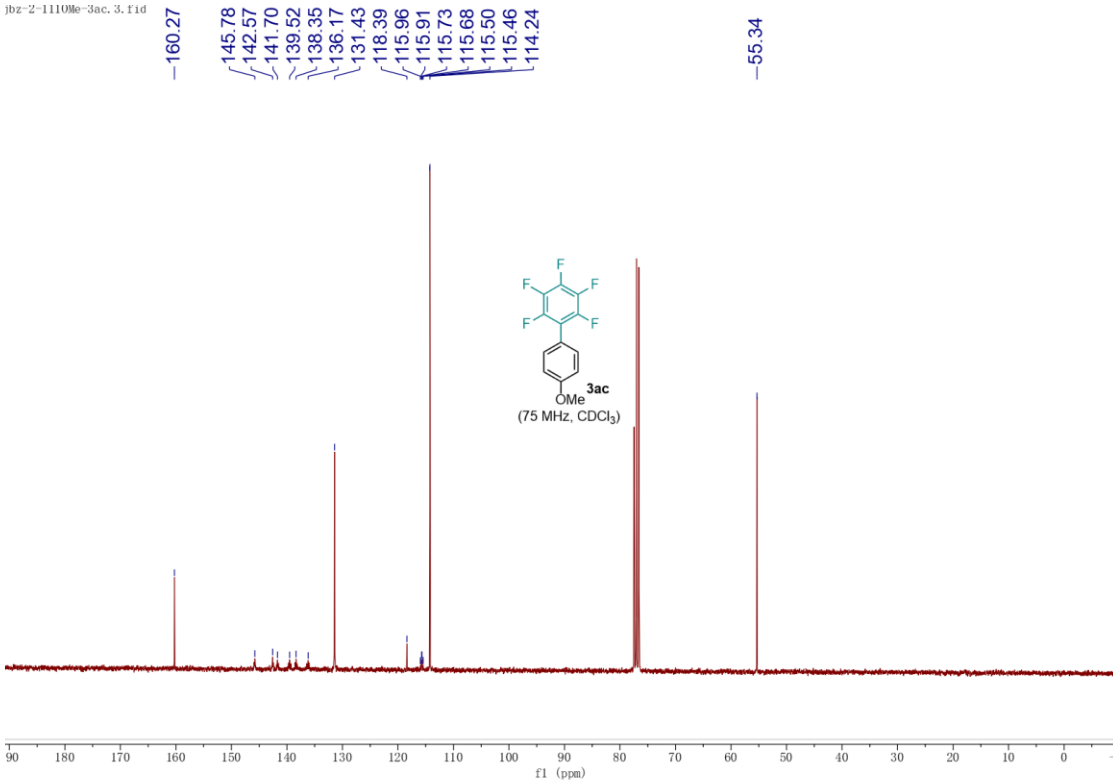
jbz-2-1110Me-3ac.3.fid
160.27
145.78
142.57
141.70
139.52
138.35
136.17
131.43
118.39
115.96
115.91
115.73
115.68
115.50
115.46
114.24
55.34
3ac
(75 MHz, CDCl$_3$)
f1 (ppm)

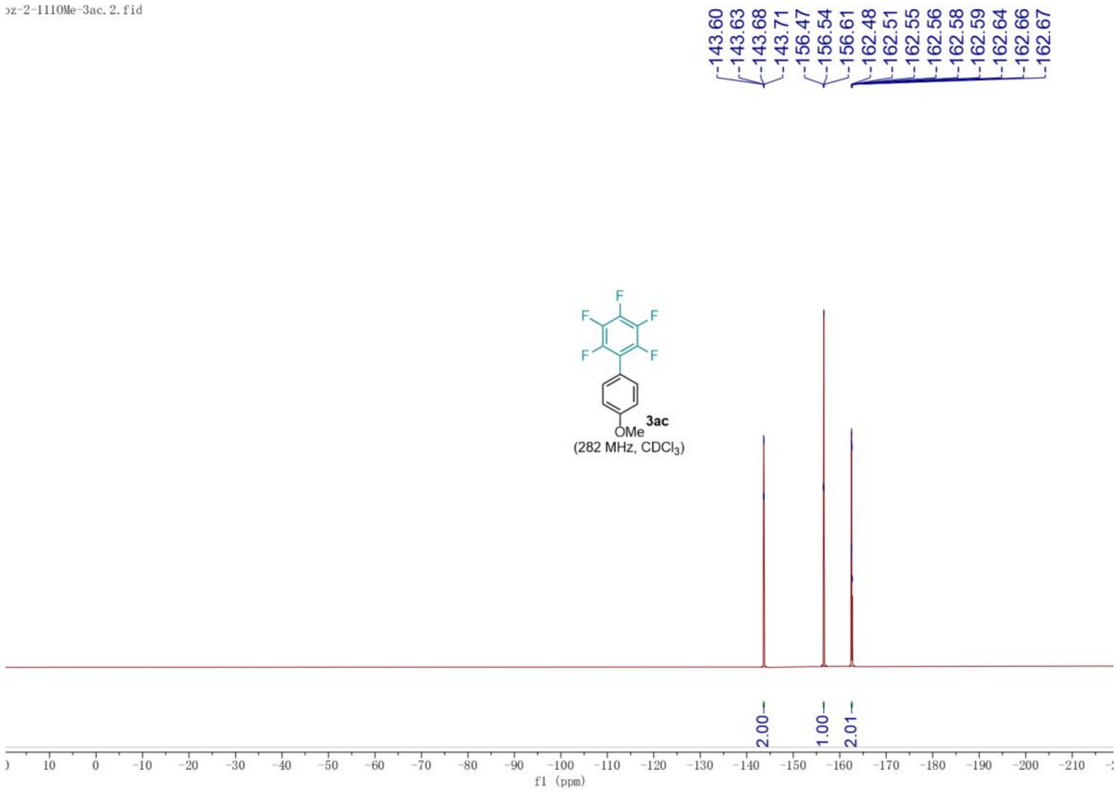
bz-2-1110Me-3ac.2.fid
-143.60
-143.63
-143.68
-143.71
-156.47
-156.54
-156.61
-162.48
-162.51
-162.55
-162.56
-162.58
-162.59
-162.64
-162.66
-162.67
3ac
(282 MHz, CDCl3)
2.00
1.00
2.01
f1 (ppm)

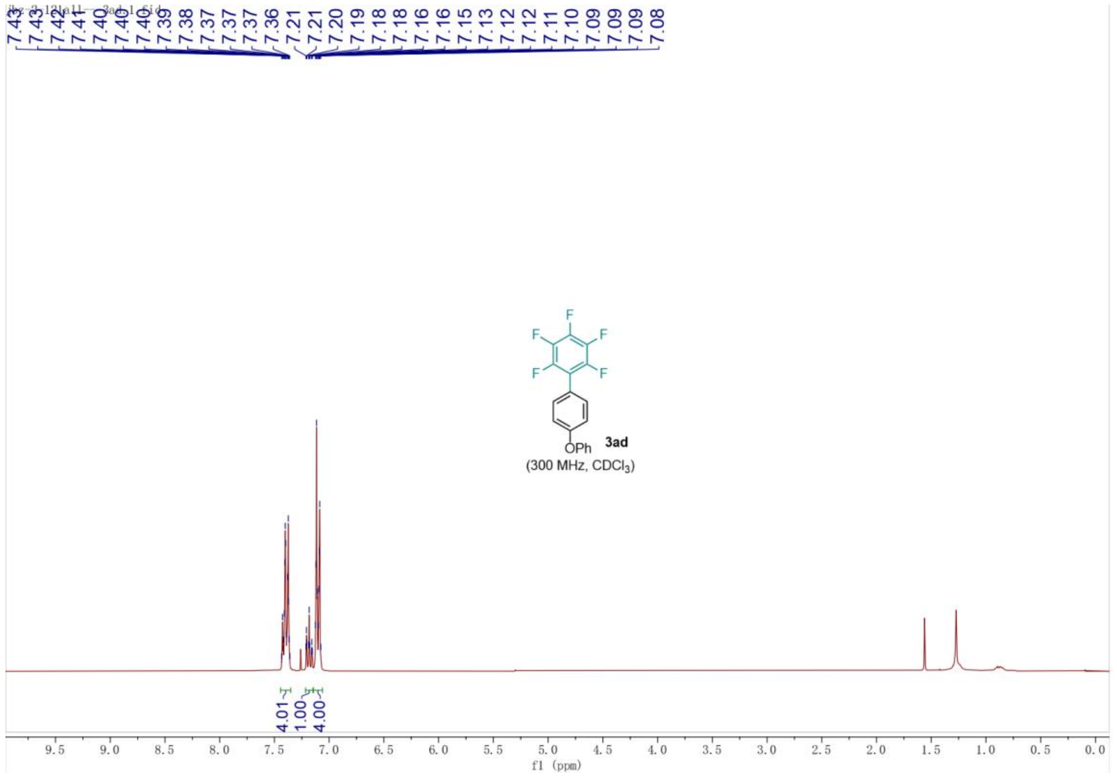
7.43
7.43
7.42
7.41
7.40
7.40
7.39
7.38
7.37
7.37
7.36
7.21
7.21
7.20
7.19
7.18
7.18
7.16
7.15
7.13
7.12
7.12
7.11
7.10
7.09
7.09
7.08
3ad
(300 MHz, CDCl3)
4.01
1.00
4.00
f1 (ppm)

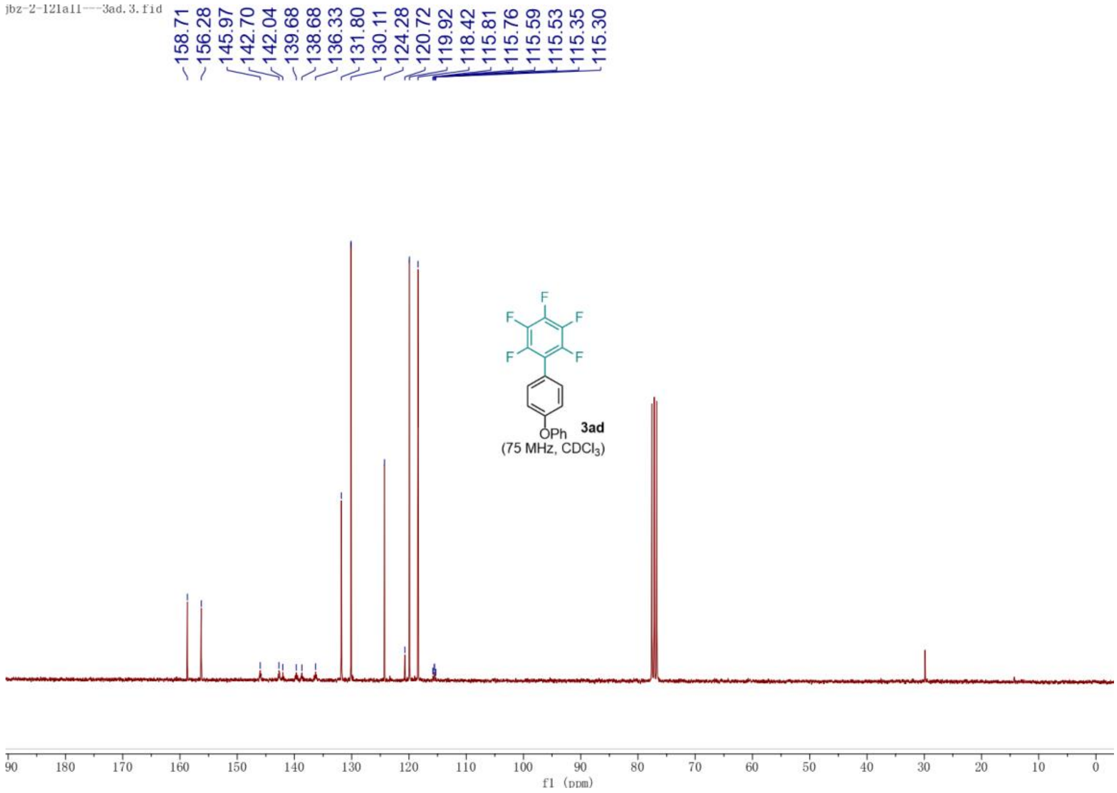
jbz-2-121all---3ad.3.fid
158.71
156.28
145.97
142.70
142.04
139.68
138.68
136.33
131.80
130.11
124.28
120.72
119.92
118.42
115.81
115.76
115.59
115.53
115.35
115.30
3ad
(75 MHz, CDCl3)
f1 (ppm)

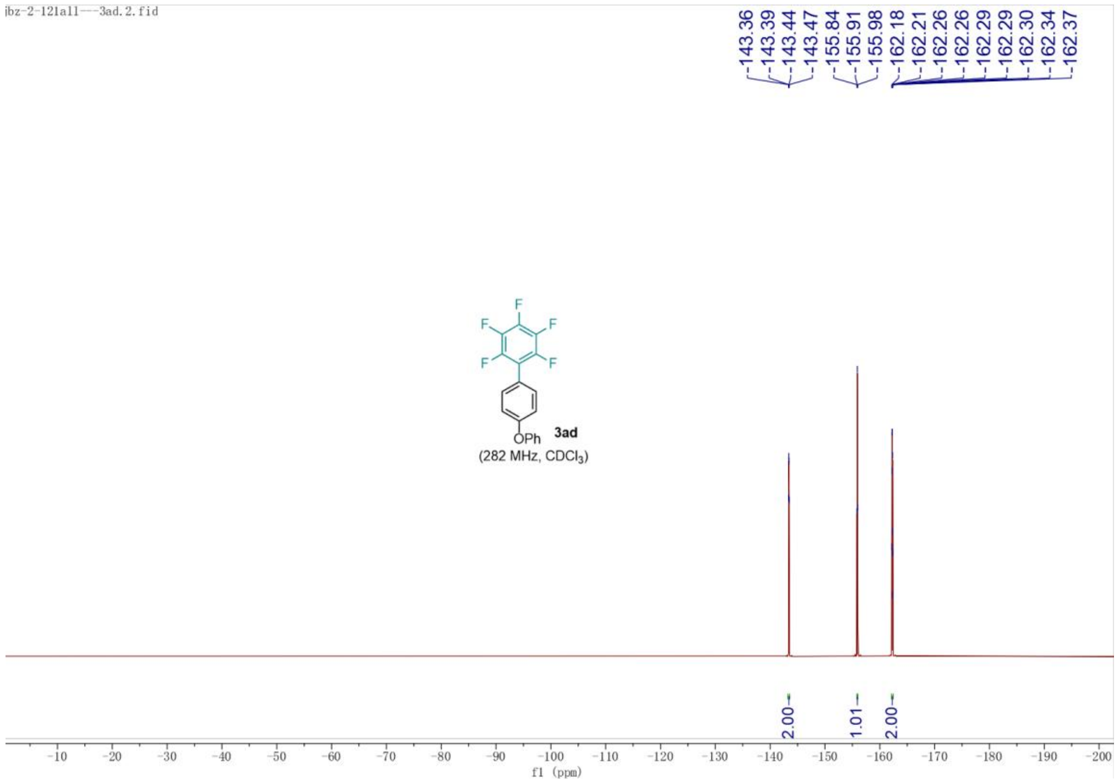
jbz-2-121all---3ad.2.fid
-143.36
-143.39
-143.44
-143.47
-155.84
-155.91
-155.98
-162.18
-162.21
-162.26
-162.26
-162.29
-162.29
-162.30
-162.34
-162.37
3ad
(282 MHz, CDCl3)
2.00
1.01
2.00
f1 (ppm)

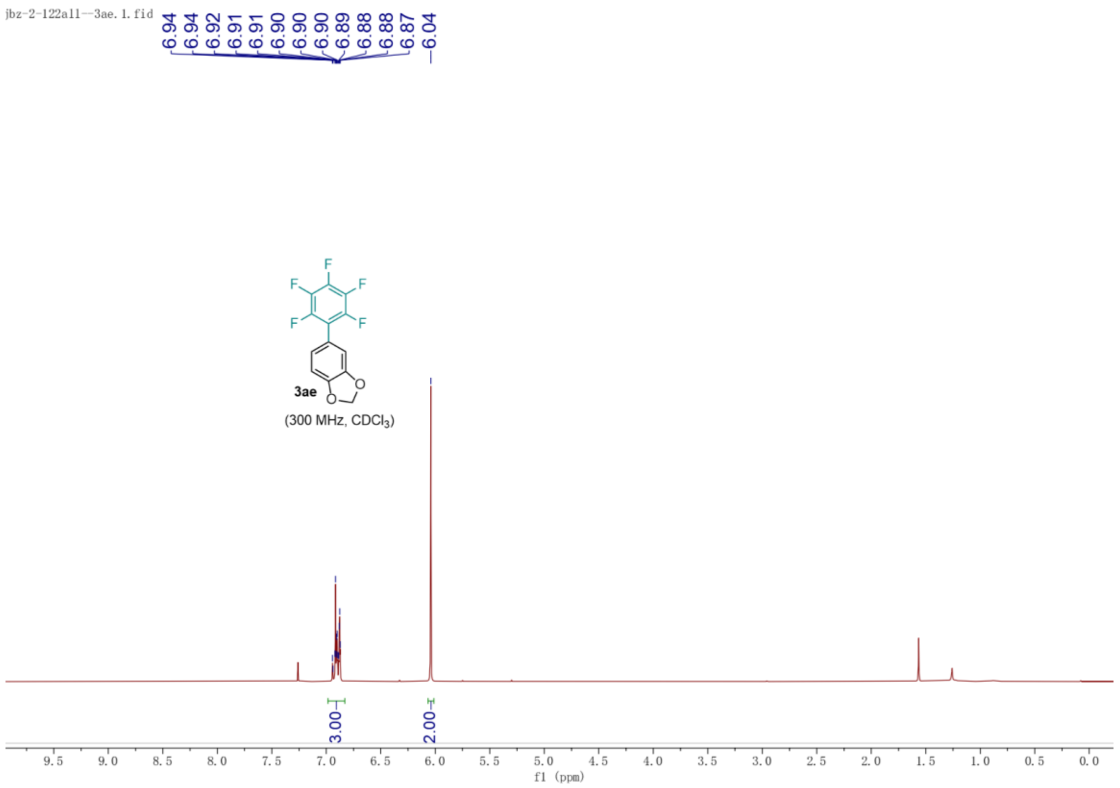
jbz-2-122all--3ae.1.fid
6.94
6.94
6.92
6.91
6.91
6.90
6.90
6.90
6.89
6.88
6.88
6.87
6.04
3ae
(300 MHz, CDCl3)
3.00
2.00
f1 (ppm)

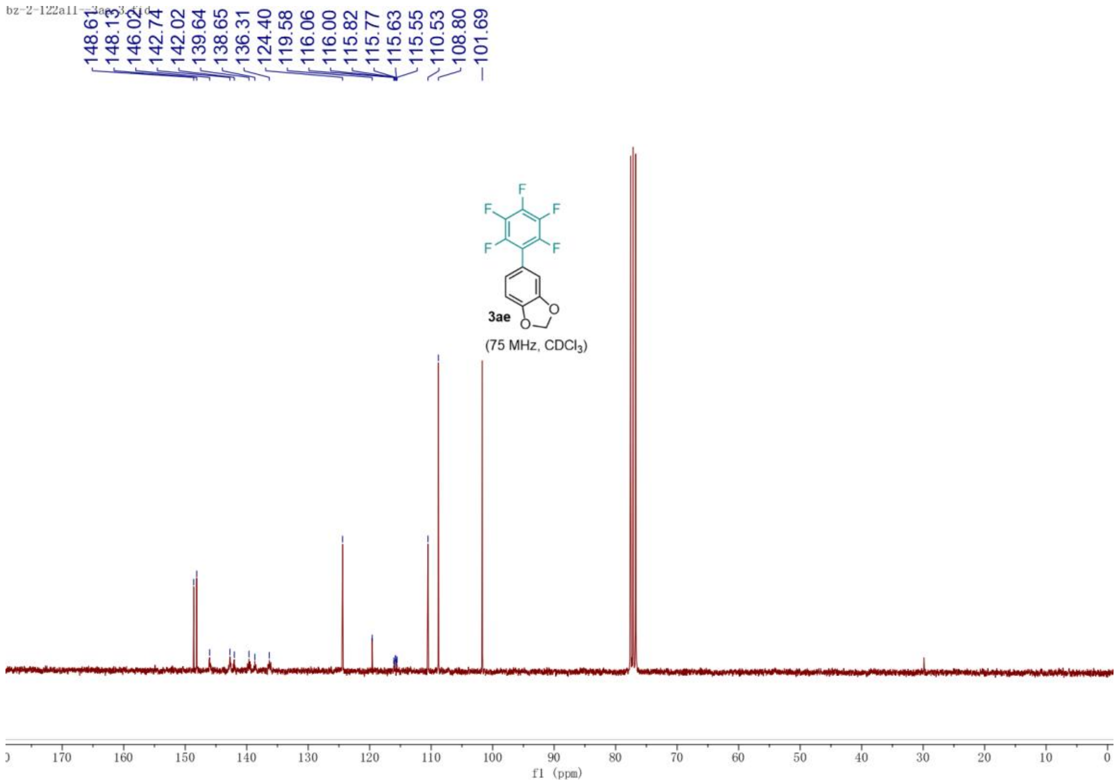
148.61
148.13
146.02
142.74
142.02
139.64
138.65
136.31
124.40
119.58
116.06
116.00
115.82
115.77
115.63
115.55
110.53
108.80
101.69
3ae
(75 MHz, CDCl3)
f1 (ppm)

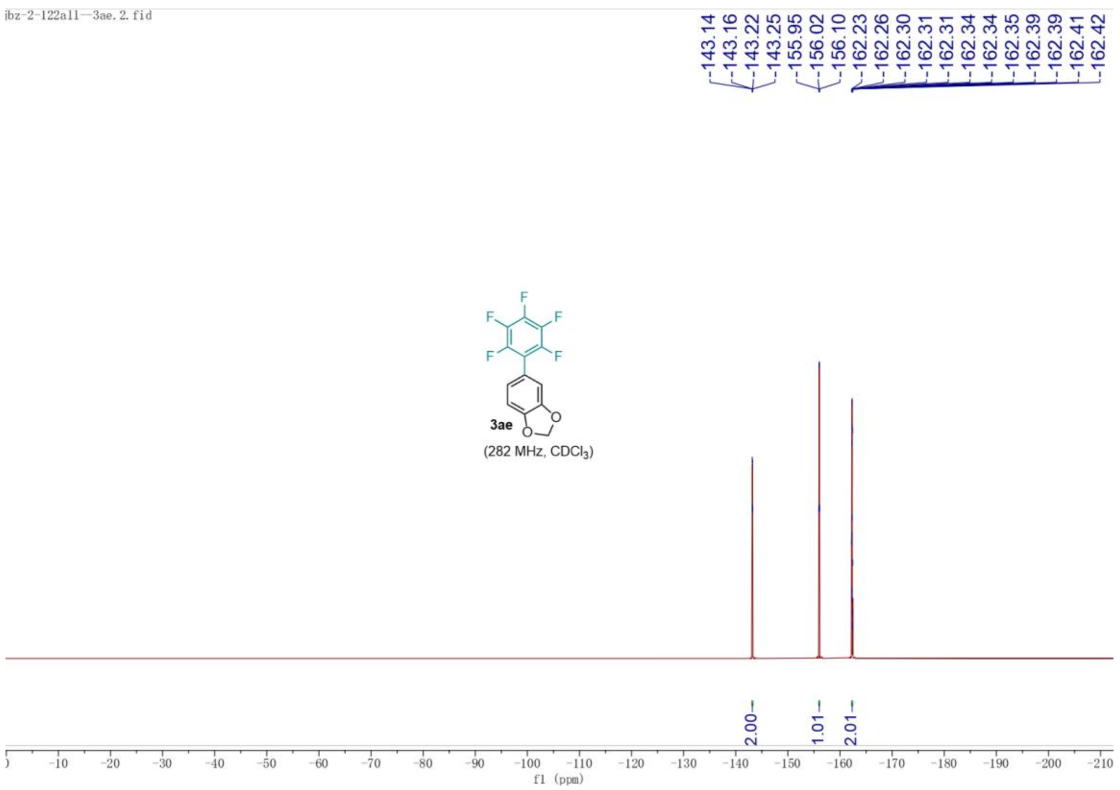
jbz-2-122all—3ae.2.fid
-143.14
-143.16
-143.22
-143.25
-155.95
-156.02
-156.10
-162.23
-162.26
-162.30
-162.31
-162.31
-162.34
-162.35
-162.39
-162.39
-162.41
-162.42
F
F
F
F
F
O
O
3ae
(282 MHz, CDCl3)
2.00
1.01
2.01
0
-10
-20
-30
-40
-50
-60
-70
-80
-90
-100
-110
-120
-130
-140
-150
-160
-170
-180
-190
-200
-210
f1 (ppm)

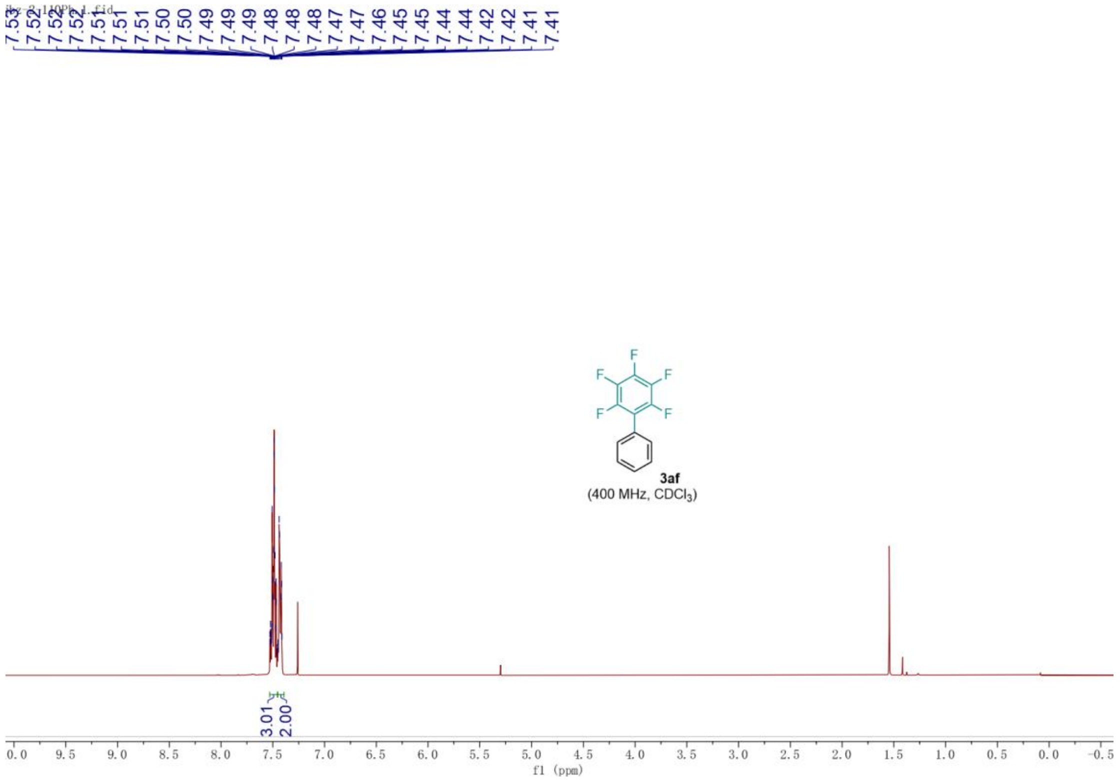
7.53
7.52
7.52
7.52
7.51
7.51
7.51
7.50
7.50
7.49
7.49
7.49
7.48
7.48
7.48
7.47
7.47
7.46
7.45
7.45
7.44
7.44
7.42
7.42
7.41
7.41
F
F
F
F
F
3af
(400 MHz, CDCl3)
3.01
2.00
10.0
9.5
9.0
8.5
8.0
7.5
7.0
6.5
6.0
5.5
5.0
4.5
4.0
3.5
3.0
2.5
2.0
1.5
1.0
0.5
0.0
-0.5
f1 (ppm)

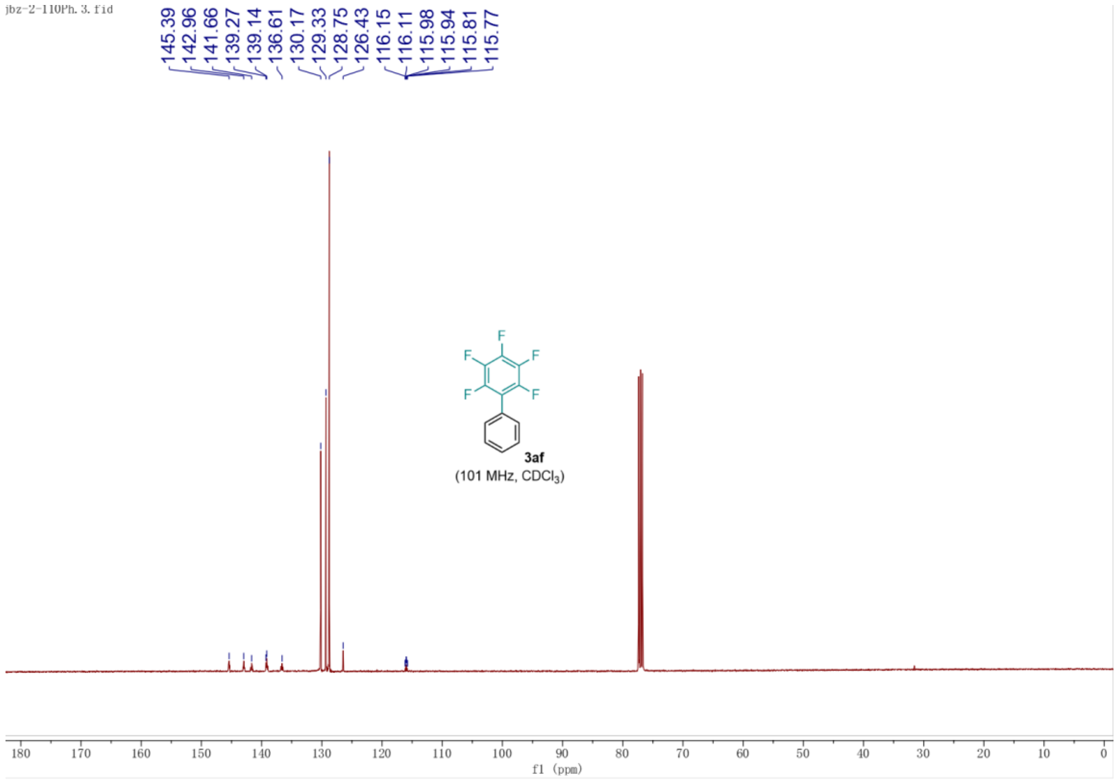
jbz-2-110Ph. 3. fid
145.39
142.96
141.66
139.27
139.14
136.61
130.17
129.33
128.75
126.43
116.15
116.11
115.98
115.94
115.81
115.77
3af
(101 MHz, CDCl3)
f1 (ppm)

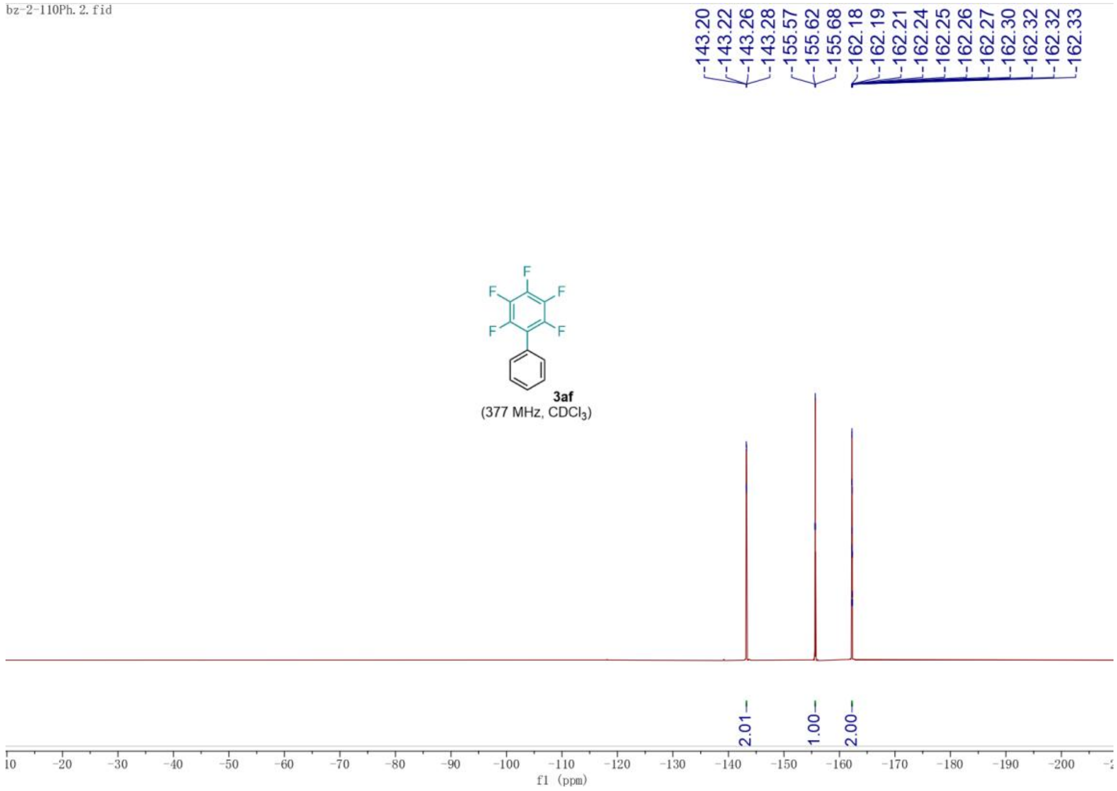
bz-2-110Ph. 2. fid
-143.20
-143.22
-143.26
-143.28
-155.57
-155.62
-155.68
-162.18
-162.19
-162.21
-162.24
-162.25
-162.26
-162.27
-162.30
-162.32
-162.32
-162.33
3af
(377 MHz, CDCl3)
2.01
1.00
2.00
f1 (ppm)

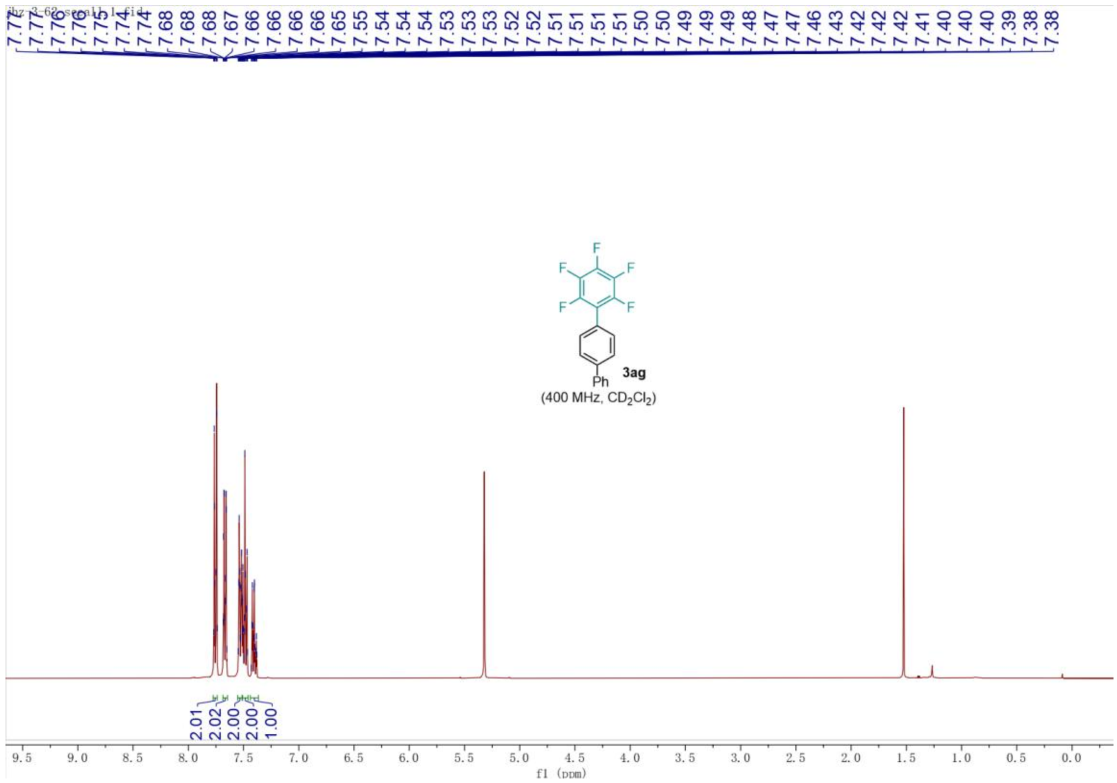
3ag
(400 MHz, $CD_2Cl_2$)

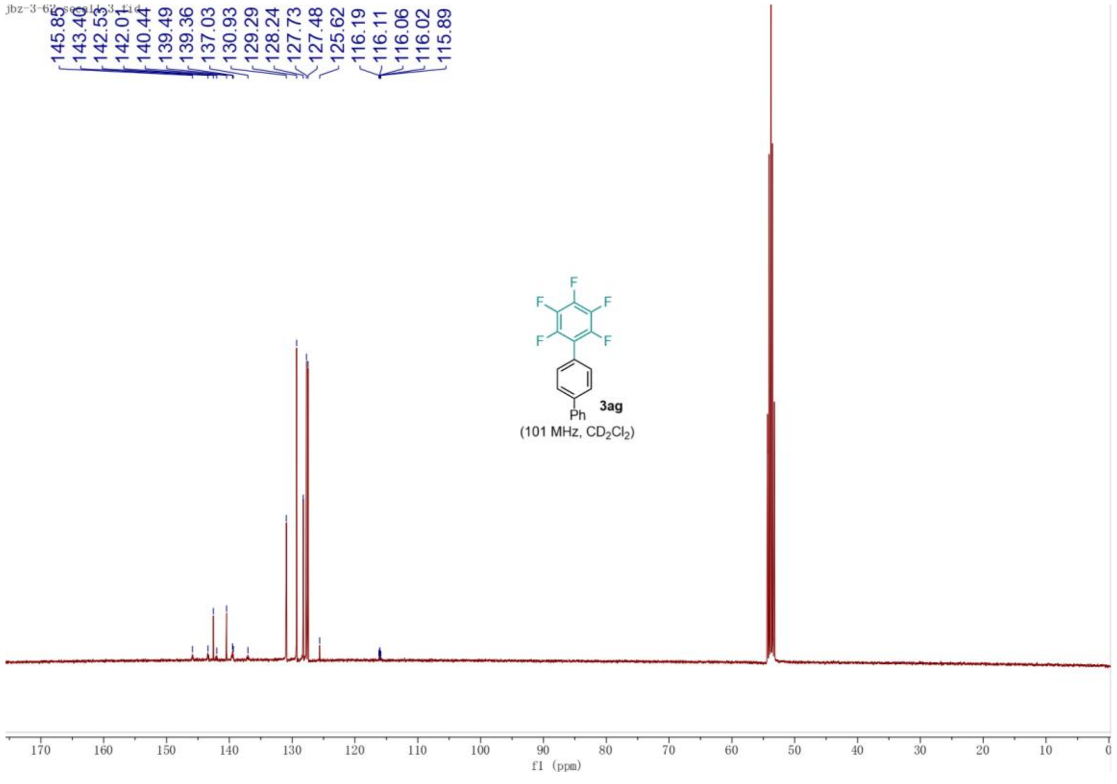
3ag
(101 MHz, $CD_2Cl_2$)

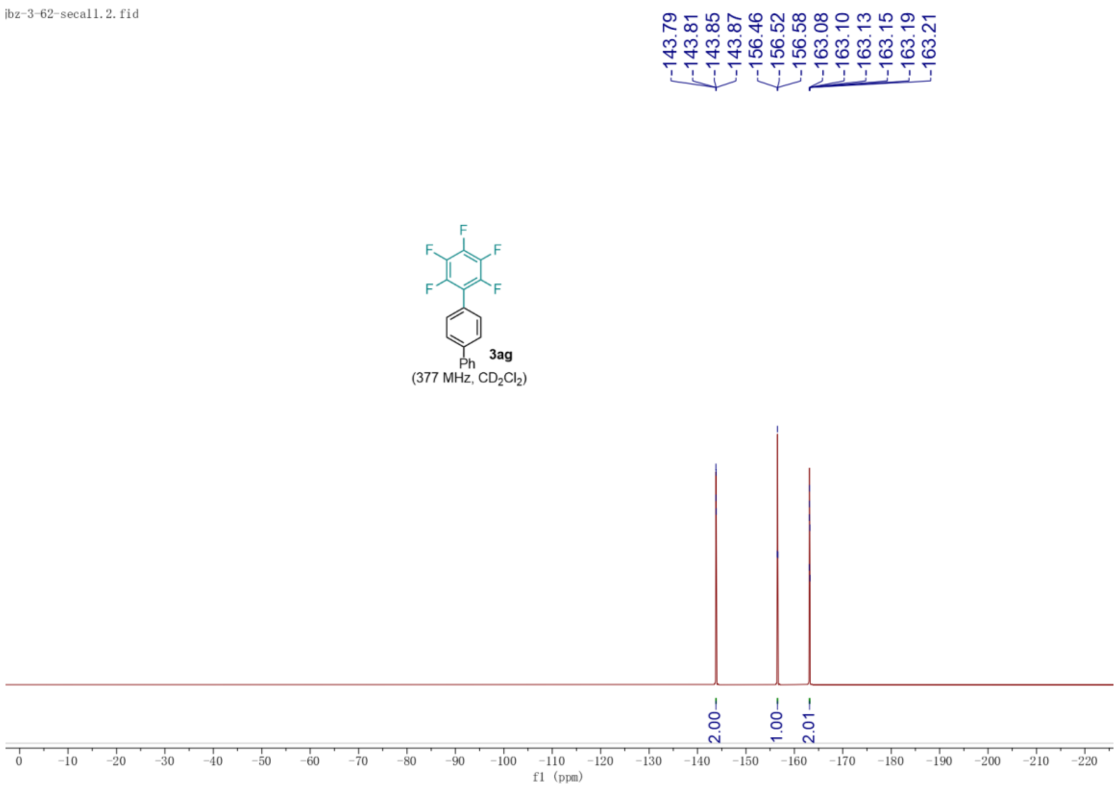
jbz-3-62-secal1.2.fid
143.79
143.81
143.85
143.87
156.46
156.52
156.58
163.08
163.10
163.13
163.15
163.19
163.21
F
F
F
F
F
Ph
3ag
(377 MHz, $CD_2Cl_2$)
2.00
1.00
2.01
0
−10
−20
−30
−40
−50
−60
−70
−80
−90
−100
−110
−120
−130
−140
−150
−160
−170
−180
−190
−200
−210
−220
f1 (ppm)

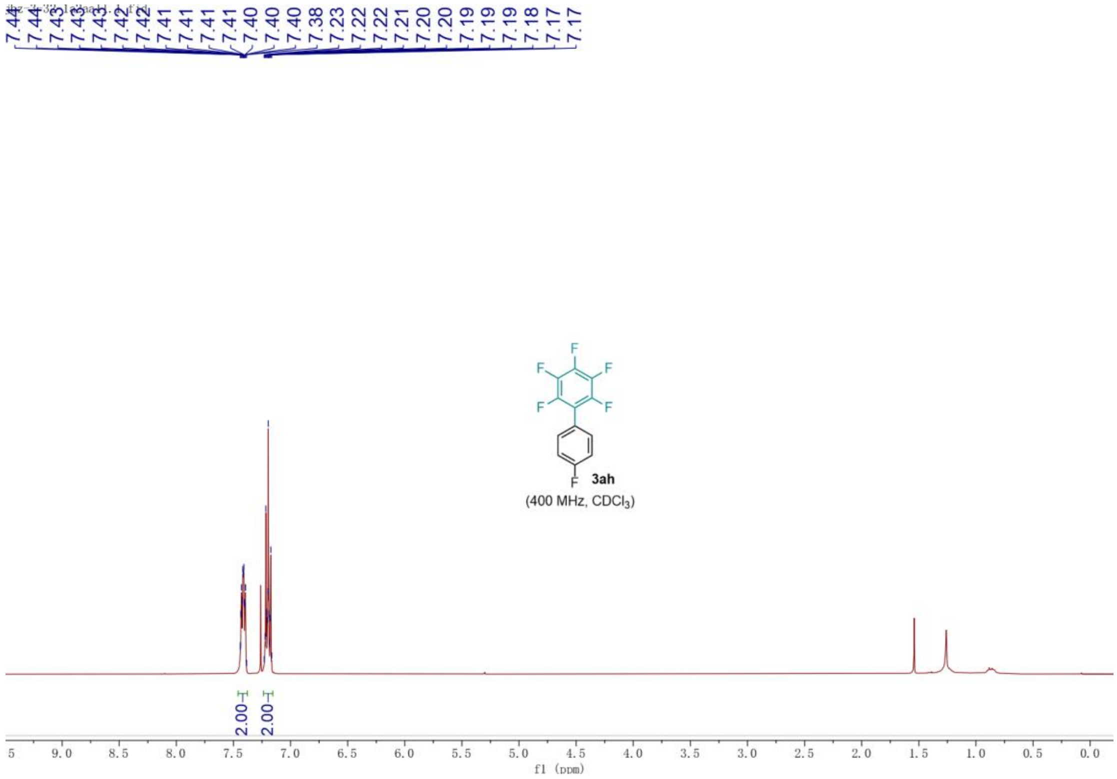
7.44
7.44
7.43
7.43
7.43
7.42
7.42
7.41
7.41
7.41
7.41
7.40
7.40
7.40
7.38
7.23
7.22
7.22
7.21
7.20
7.19
7.19
7.19
7.18
7.17
7.17
F
F
F
F
F
F
3ah
(400 MHz, $CDCl_3$)
2.00
2.00
9.0
8.5
8.0
7.5
7.0
6.5
6.0
5.5
5.0
4.5
4.0
3.5
3.0
2.5
2.0
1.5
1.0
0.5
0.0
f1 (ppm)

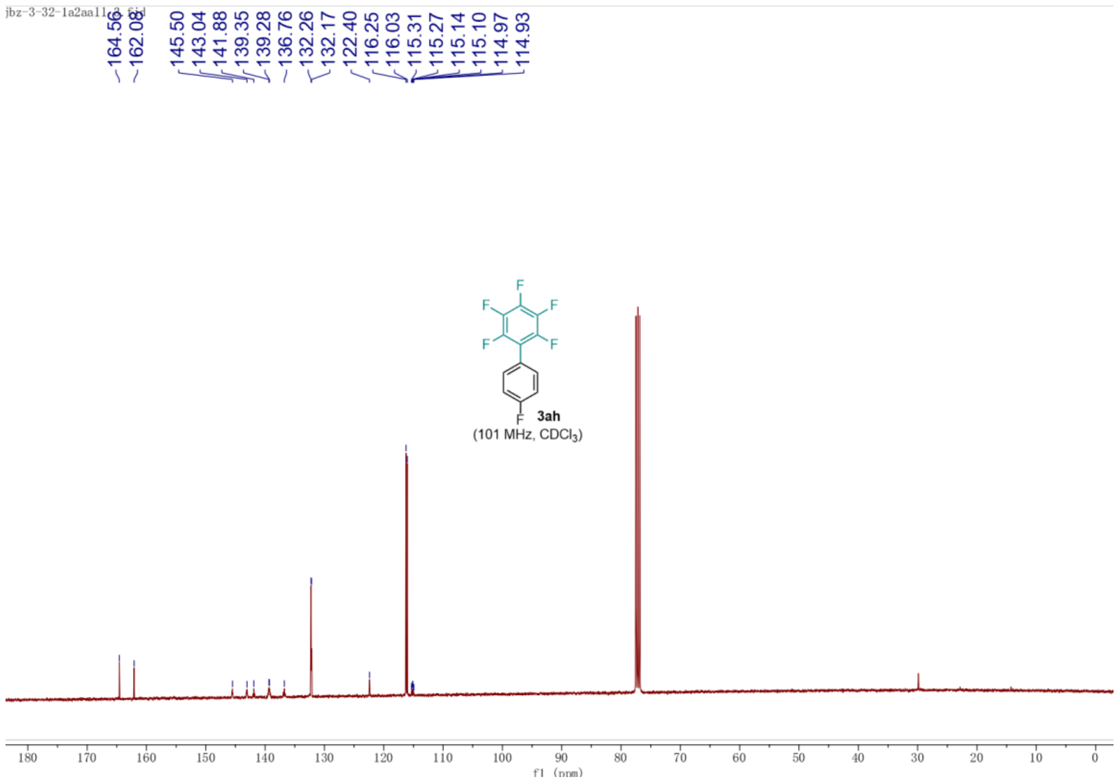
jbz-3-32-1a2aa11.2.fid
164.56
162.08
145.50
143.04
141.88
139.35
139.28
136.76
132.26
132.17
122.40
116.25
116.03
115.31
115.27
115.14
115.10
114.97
114.93
3ah
(101 MHz, CDCl3)
f1 (ppm)

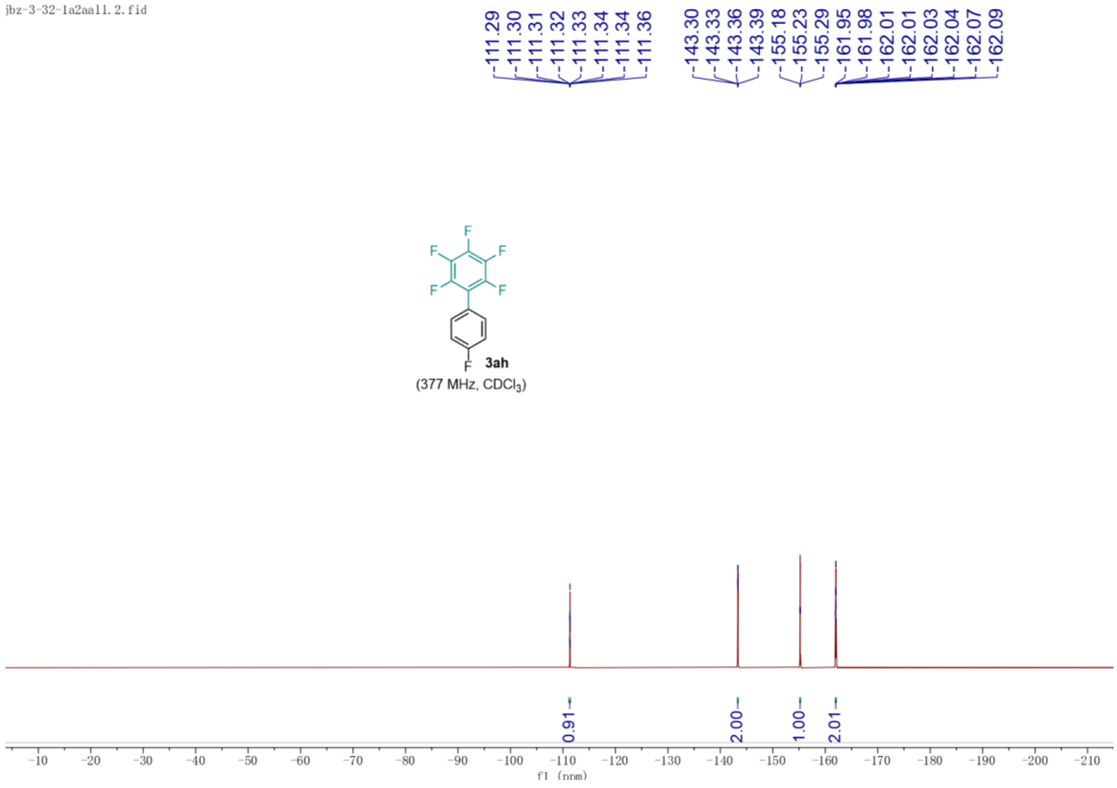
jbz-3-32-1a2aa11.2.fid
-111.29
-111.30
-111.31
-111.32
-111.33
-111.34
-111.34
-111.36
-143.30
-143.33
-143.36
-143.39
-155.18
-155.23
-155.29
-161.95
-161.98
-162.01
-162.03
-162.04
-162.07
-162.09
3ah
(377 MHz, CDCl3)
0.91
2.00
1.00
2.01
f1 (ppm)

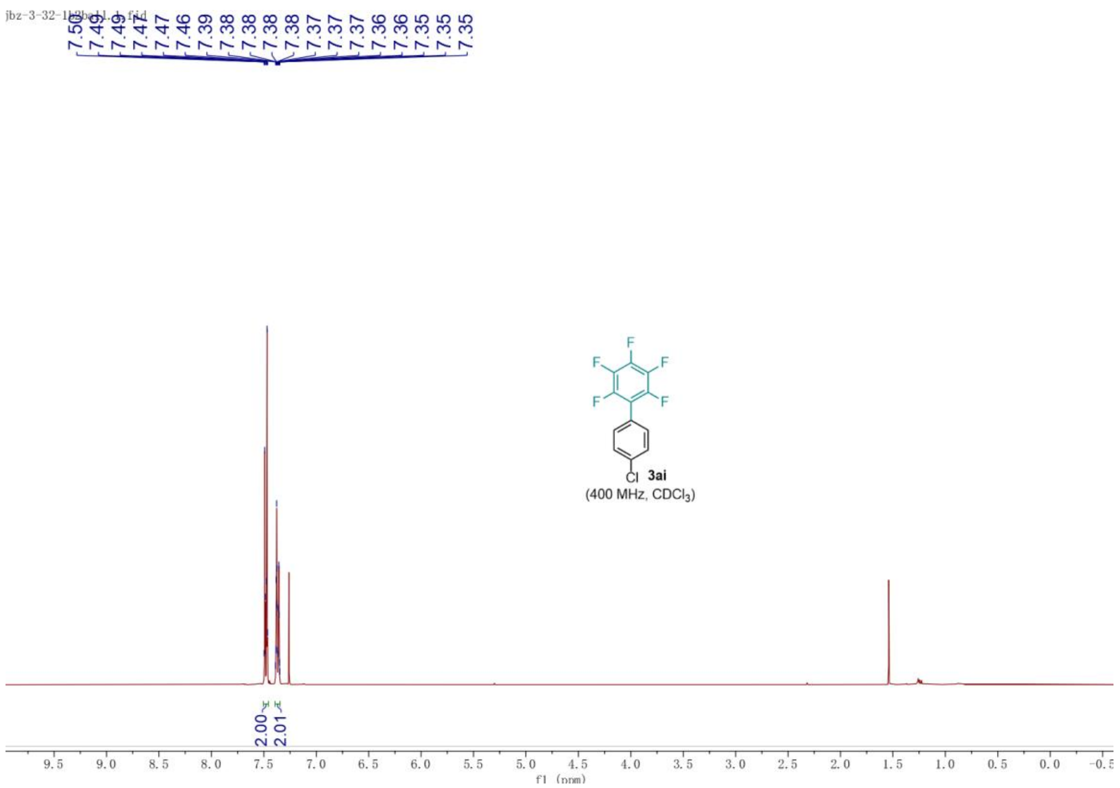
jbz-3-32-1b2ba11.1.fid
7.50
7.49
7.49
7.47
7.47
7.46
7.39
7.38
7.38
7.38
7.38
7.37
7.37
7.37
7.36
7.36
7.35
7.35
7.35
3ai
(400 MHz, CDCl3)
2.00
2.01
f1 (ppm)

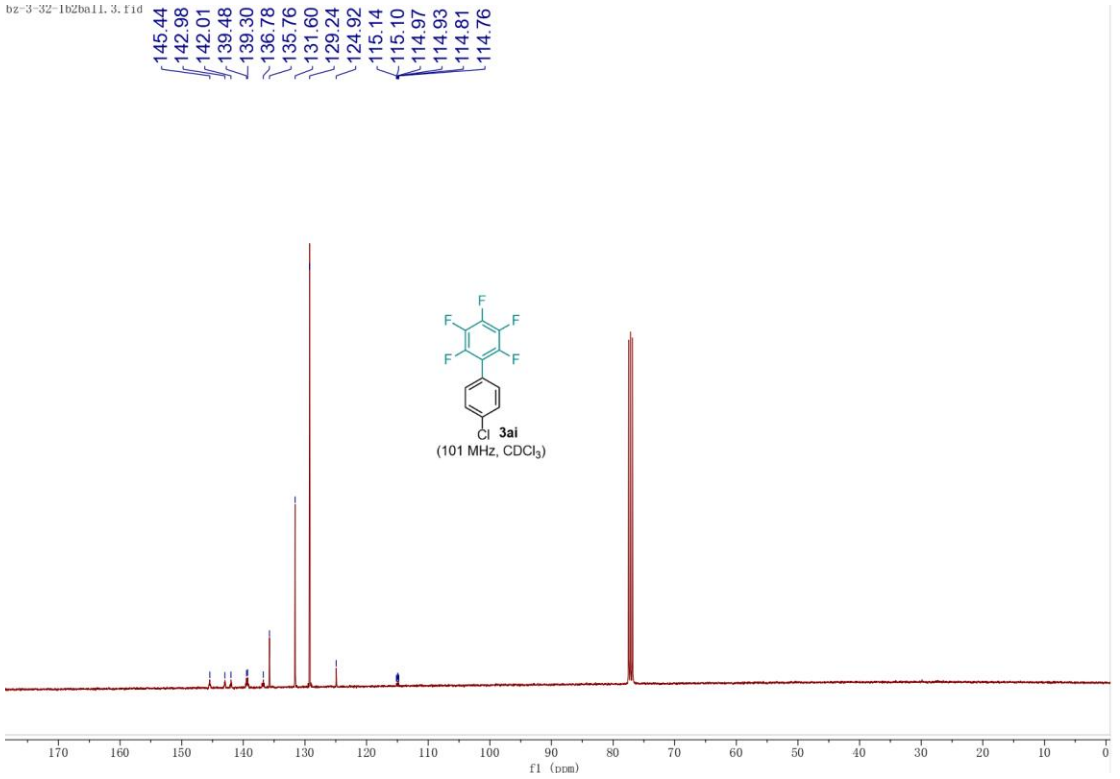
bz-3-32-1b2ba11.3.fid
145.44
142.98
142.01
139.48
139.30
136.78
135.76
131.60
129.24
124.92
115.14
115.10
114.97
114.93
114.81
114.76
3ai
(101 MHz, CDCl3)
f1 (ppm)

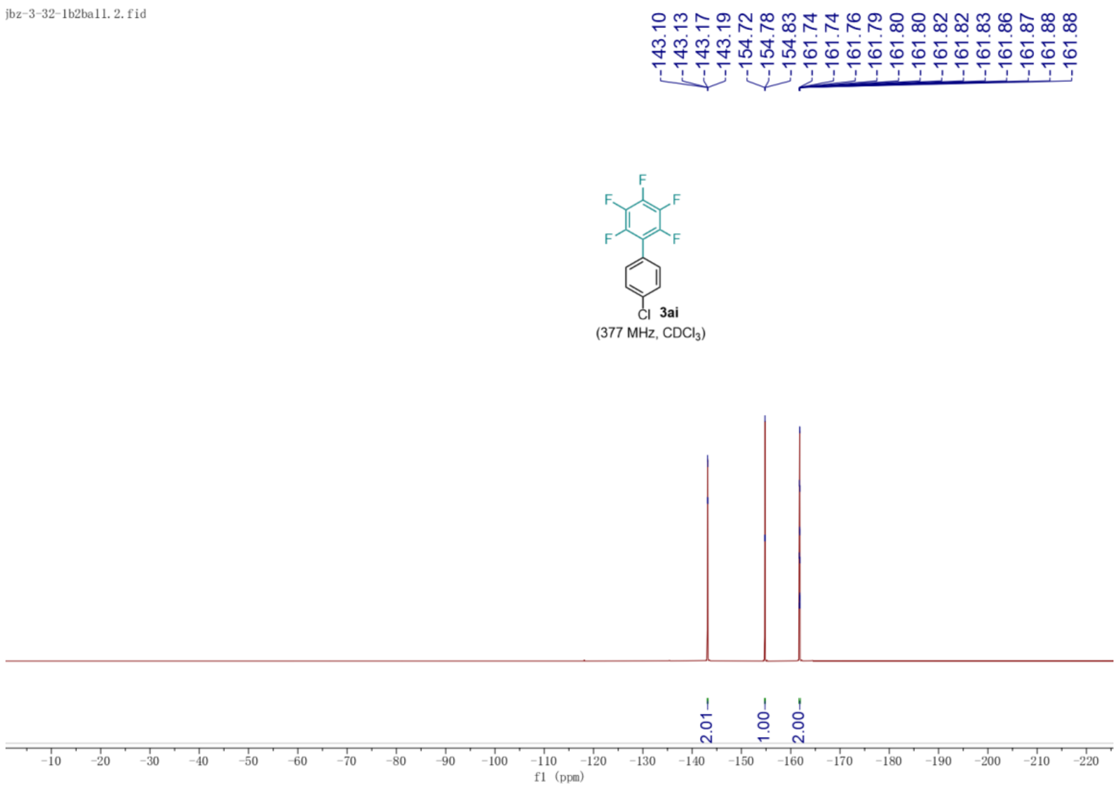
jbz-3-32-1b2ba11.2.fid
143.10
143.13
143.17
143.19
154.72
154.78
154.83
161.74
161.74
161.76
161.79
161.80
161.80
161.82
161.82
161.83
161.86
161.87
161.88
161.88
Cl 3ai
(377 MHz, CDCl3)
2.01
1.00
2.00
−10 −20 −30 −40 −50 −60 −70 −80 −90 −100 −110 −120 −130 −140 −150 −160 −170 −180 −190 −200 −210 −220
f1 (ppm)

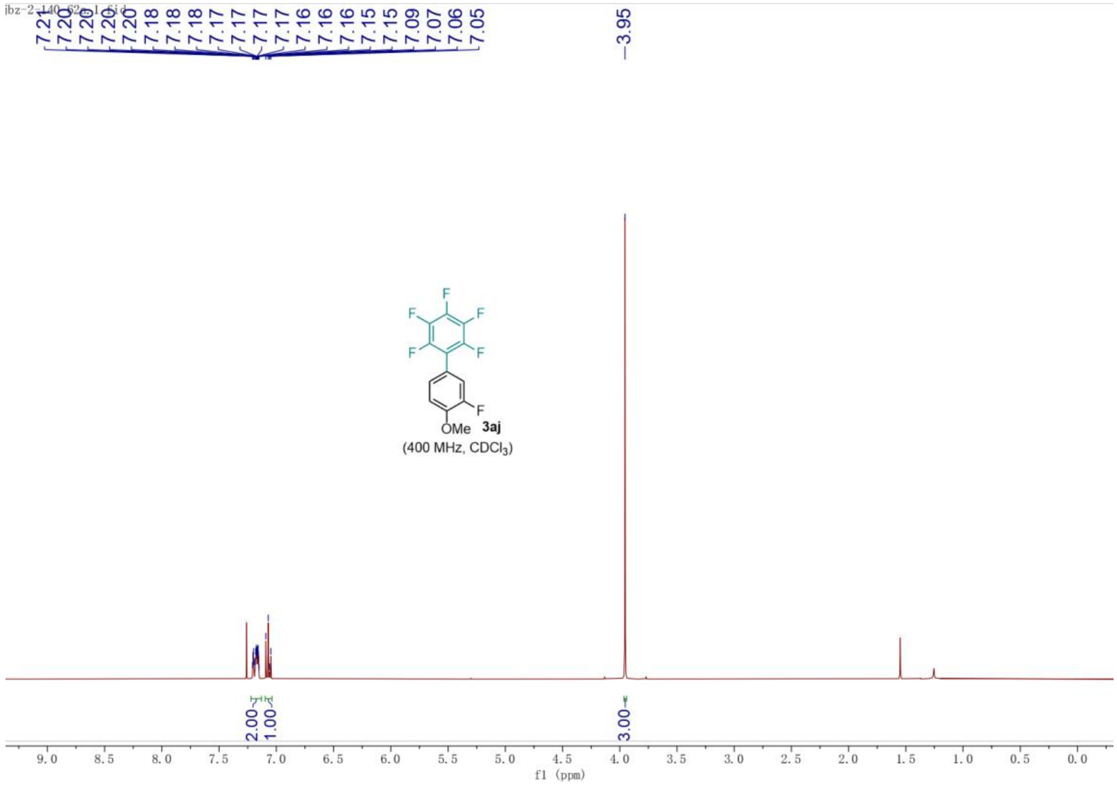
7.21
7.20
7.20
7.20
7.18
7.18
7.18
7.17
7.17
7.17
7.16
7.16
7.16
7.15
7.09
7.07
7.06
7.05
3.95
OMe 3aj
(400 MHz, CDCl3)
2.00
1.00
3.00
9.0 8.5 8.0 7.5 7.0 6.5 6.0 5.5 5.0 4.5 4.0 3.5 3.0 2.5 2.0 1.5 1.0 0.5 0.0
f1 (ppm)

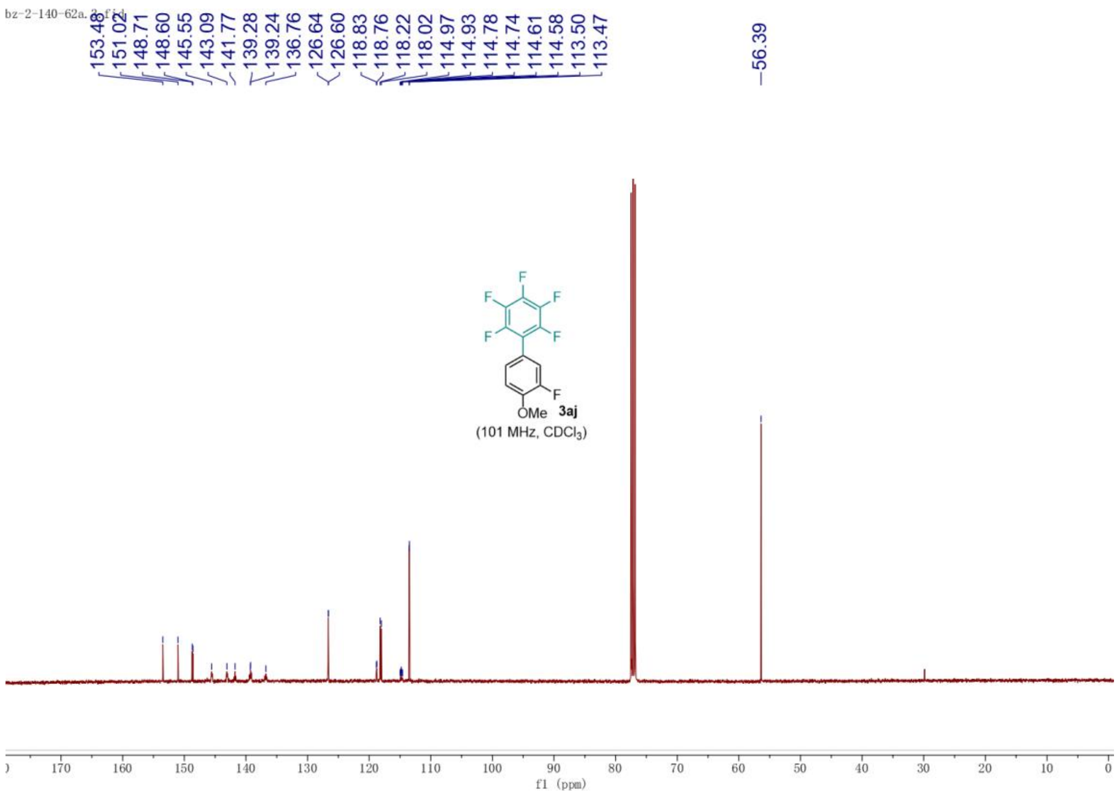
153.48
151.02
148.71
148.60
145.55
143.09
141.77
139.28
139.24
136.76
126.64
126.60
118.83
118.76
118.22
118.02
114.97
114.93
114.78
114.74
114.61
114.58
113.50
113.47
56.39
OMe 3aj
(101 MHz, CDCl3)
f1 (ppm)

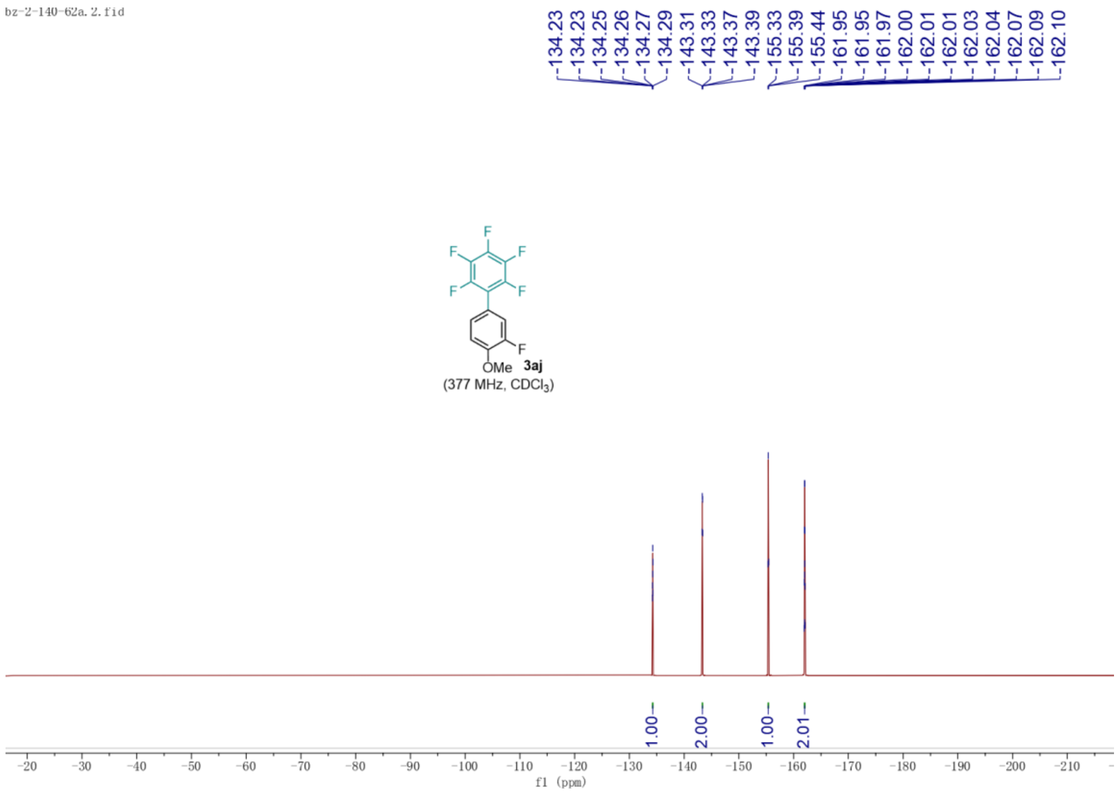
bz-2-140-62a.2.fid
-134.23
-134.23
-134.25
-134.26
-134.27
-134.29
-143.31
-143.33
-143.37
-143.39
-155.33
-155.39
-155.44
-161.95
-161.95
-161.97
-162.00
-162.01
-162.01
-162.03
-162.04
-162.07
-162.09
-162.10
OMe 3aj
(377 MHz, CDCl3)
1.00
2.00
1.00
2.01
f1 (ppm)

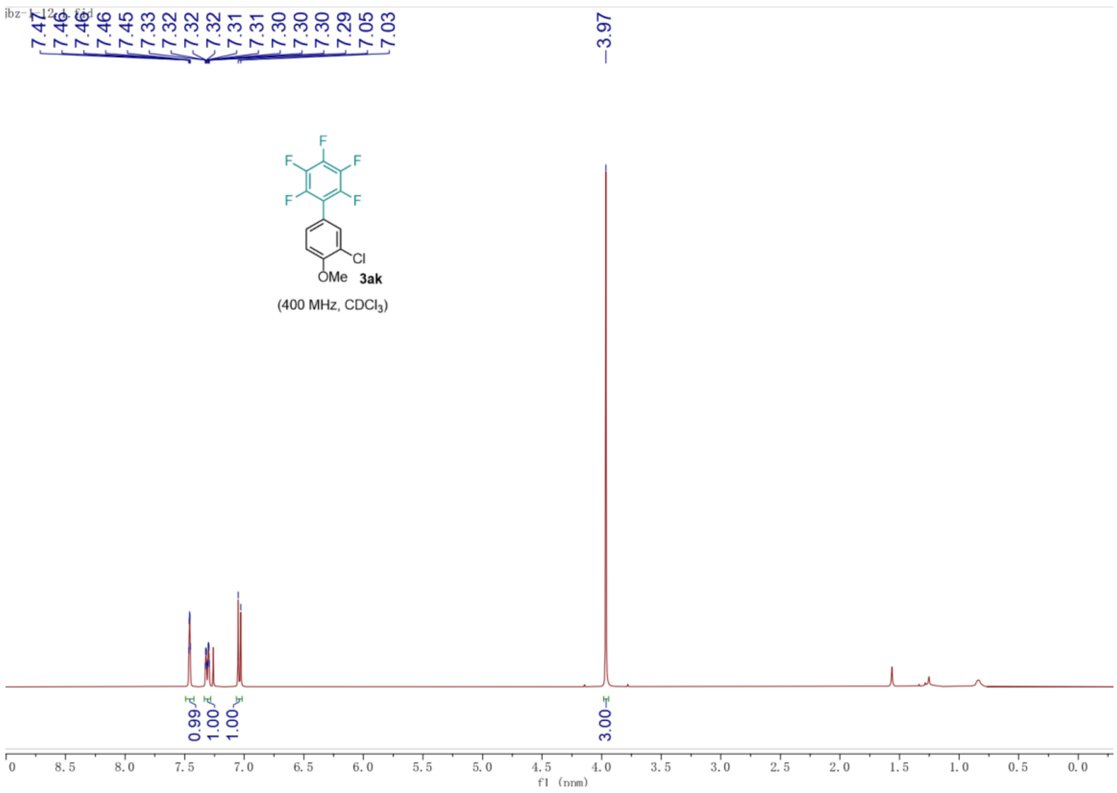
jbz-1-12-1.fid
7.47
7.46
7.46
7.46
7.45
7.33
7.32
7.32
7.32
7.31
7.31
7.30
7.30
7.30
7.29
7.05
7.03
3.97
F
F
F
F
F
Cl
OMe
3ak
(400 MHz, CDCl3)
0.99
1.00
1.00
3.00
f1 (ppm)

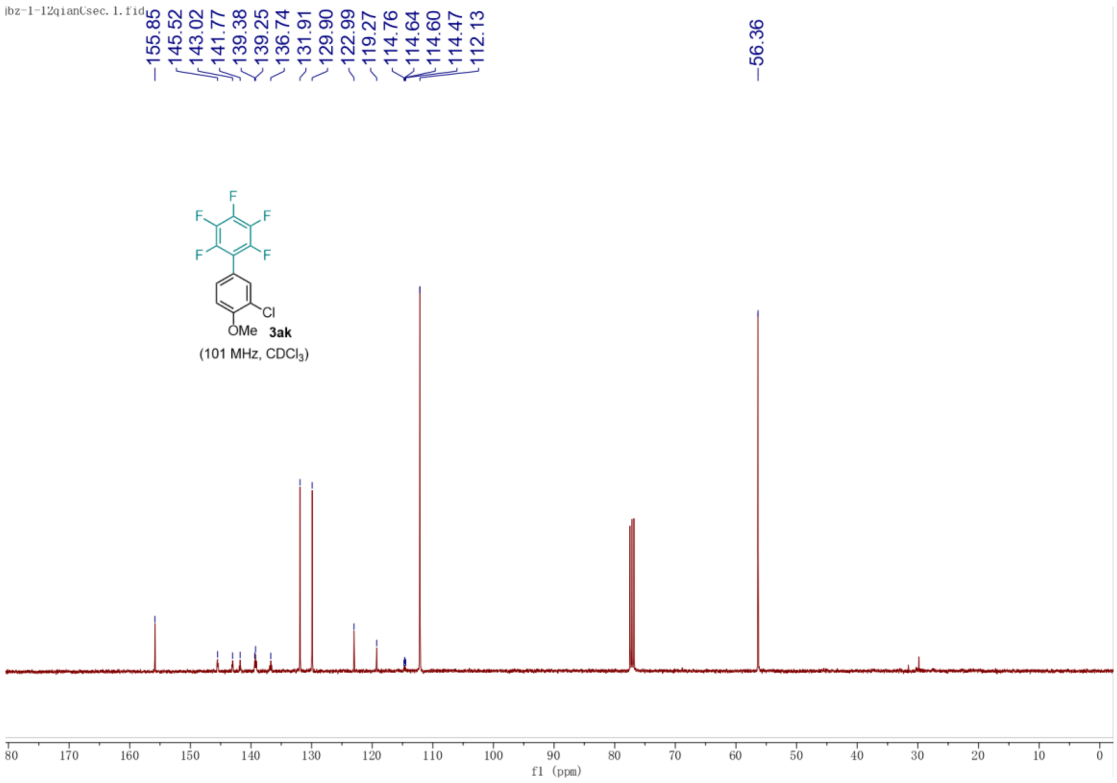
jbz-1-12qianCsec.1.fid
155.85
145.52
143.02
141.77
139.38
139.25
136.74
131.91
129.90
122.99
119.27
114.76
114.64
114.60
114.47
112.13
56.36
F
F
F
F
F
Cl
OMe
3ak
(101 MHz, CDCl3)
f1 (ppm)

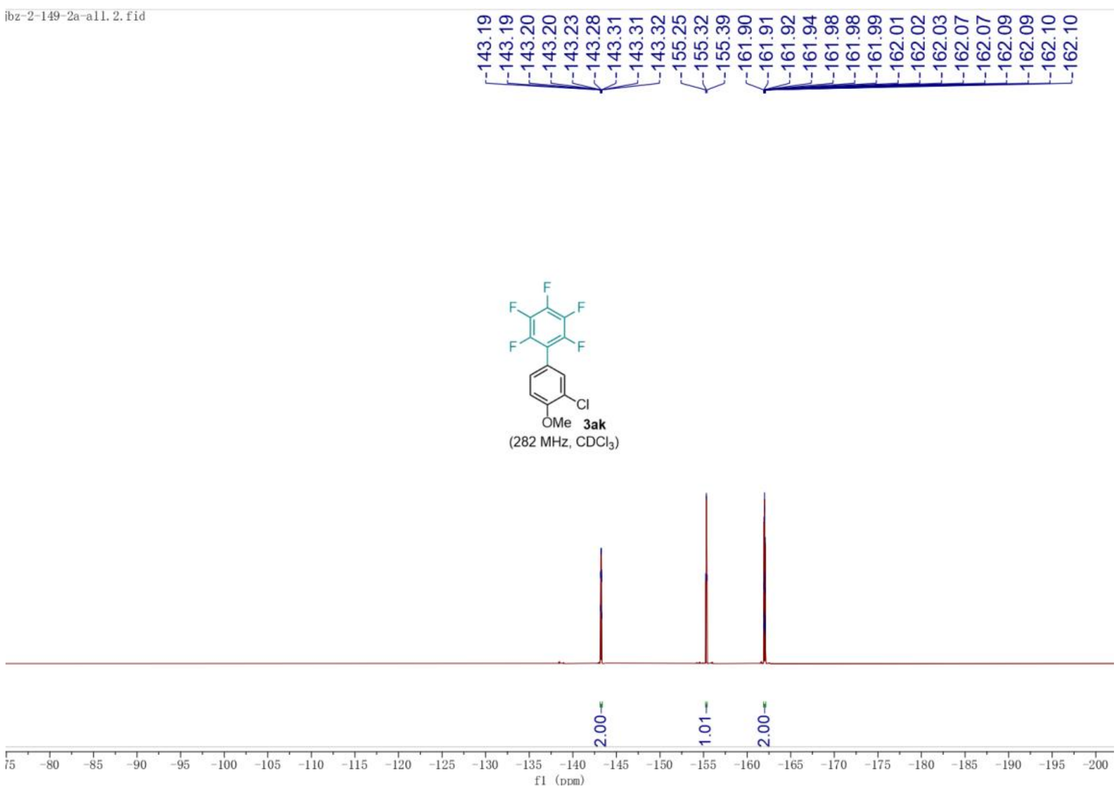
jbz-2-149-2a-all.2.fid
-143.19
-143.19
-143.20
-143.23
-143.28
-143.31
-143.31
-143.32
-155.25
-155.32
-155.39
-161.90
-161.91
-161.92
-161.94
-161.98
-161.98
-161.99
-162.01
-162.02
-162.03
-162.07
-162.07
-162.09
-162.09
-162.10
-162.10
F
F
F
F
F
Cl
OMe
3ak
(282 MHz, CDCl3)
2.00
1.01
2.00
f1 (ppm)

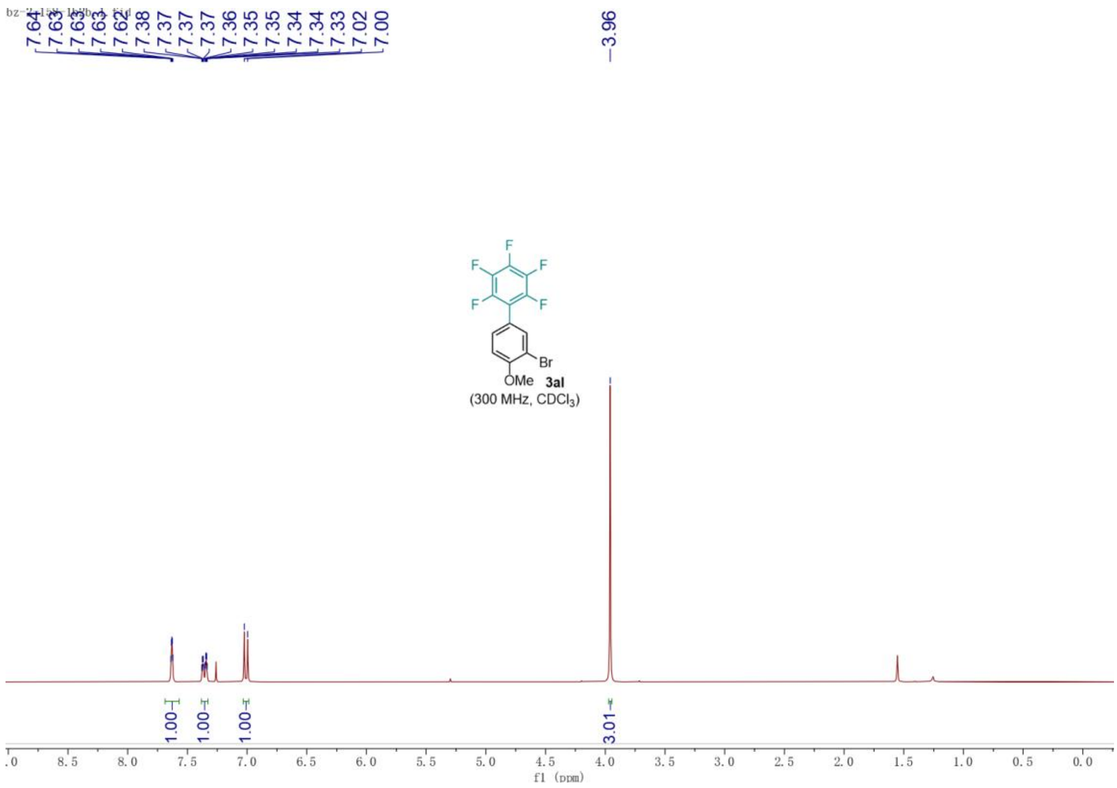
7.64
7.63
7.63
7.63
7.62
7.38
7.37
7.37
7.37
7.36
7.35
7.35
7.34
7.34
7.33
7.02
7.00
3.96
F
F
F
F
F
Br
OMe
3al
(300 MHz, CDCl3)
1.00
1.00
1.00
3.01
f1 (ppm)

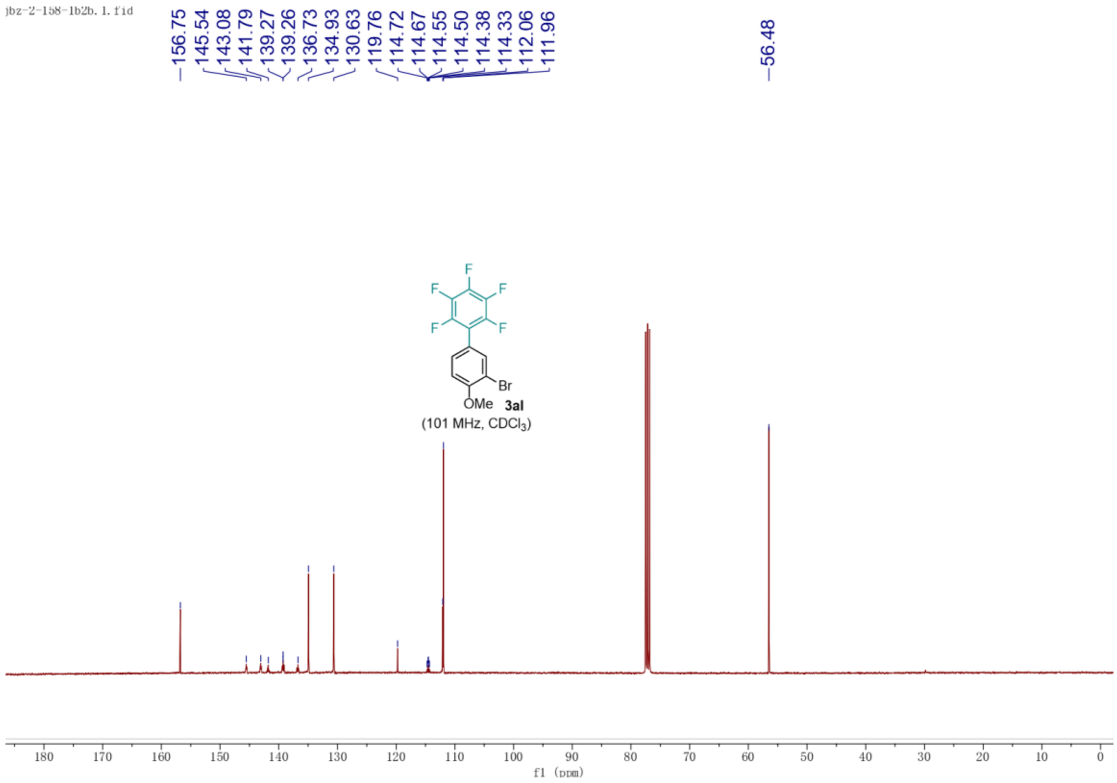
jbz-2-158-1b2b.1.fid
156.75
145.54
143.08
141.79
139.27
139.26
136.73
134.93
130.63
119.76
114.72
114.67
114.55
114.50
114.38
114.33
112.06
111.96
56.48
F
Br
OMe
3al
(101 MHz, CDCl3)
f1 (ppm)

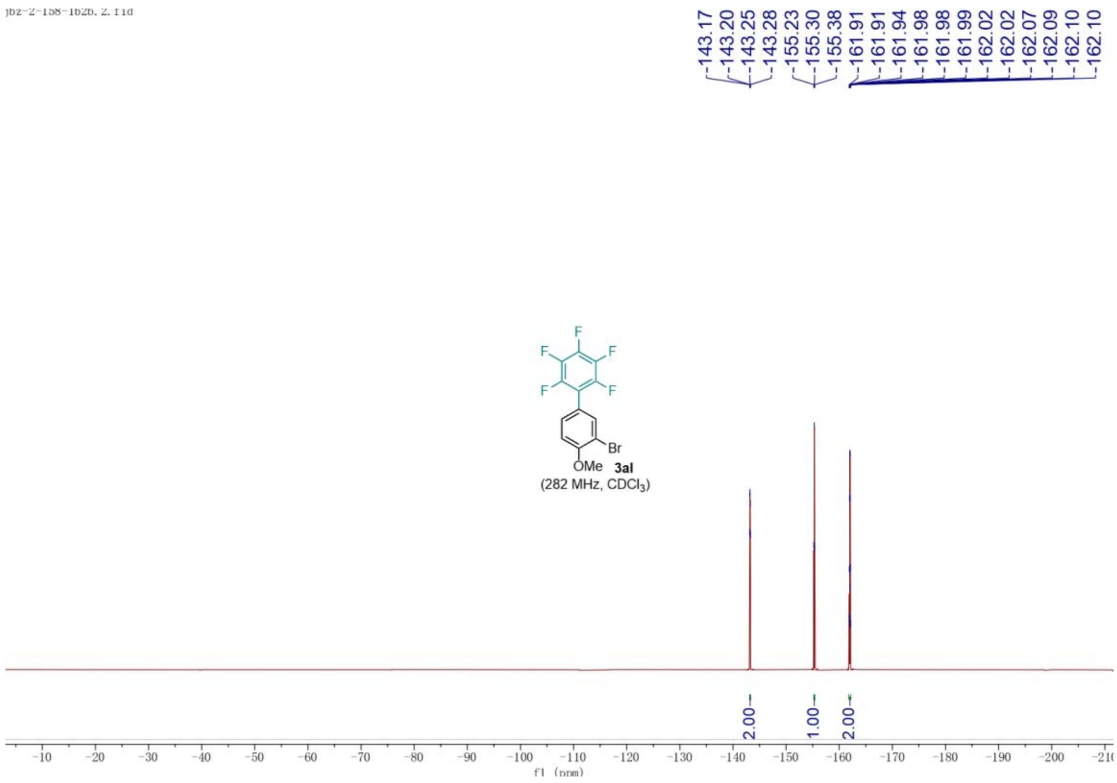
jbz-2-158-1b2b.2.fid
143.17
143.20
143.25
143.28
155.23
155.30
155.38
161.91
161.91
161.94
161.98
161.98
161.99
162.02
162.02
162.07
162.09
162.10
162.10
F
Br
OMe
3al
(282 MHz, CDCl3)
2.00
1.00
2.00
f1 (ppm)

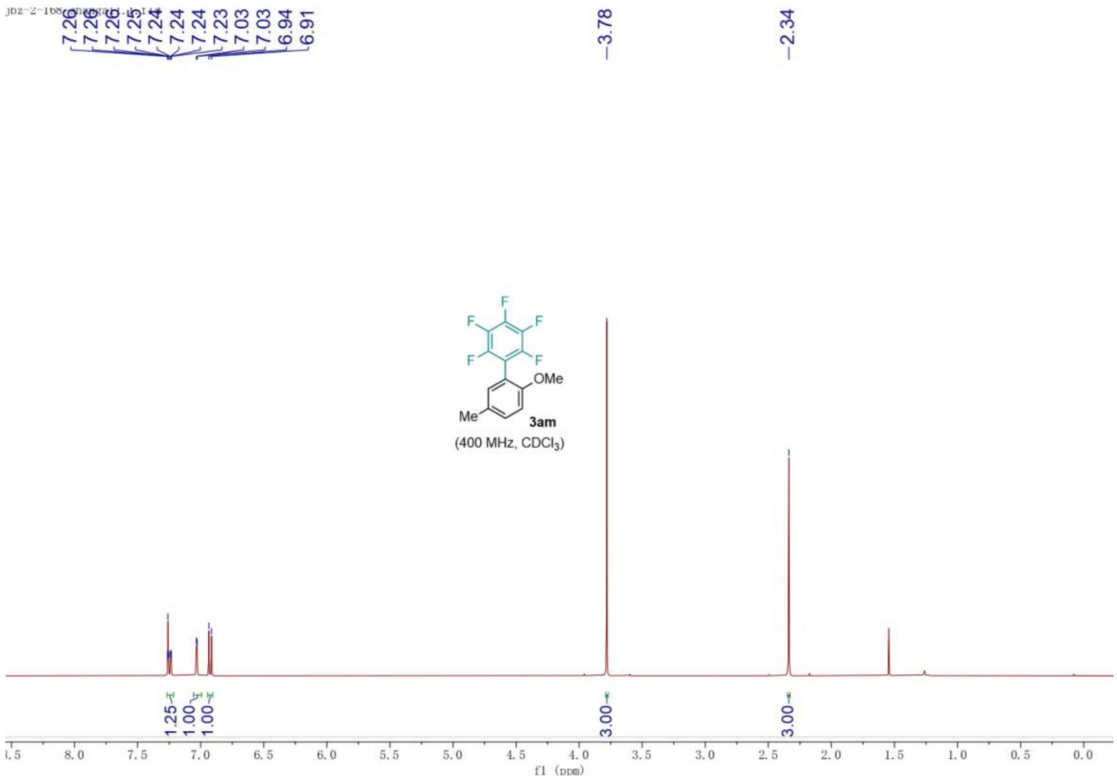
7.26
7.26
7.26
7.25
7.24
7.24
7.23
7.03
7.03
6.94
6.91
3.78
2.34
3am
(400 MHz, CDCl3)
1.25
1.00
1.00
3.00
3.00
f1 (ppm)

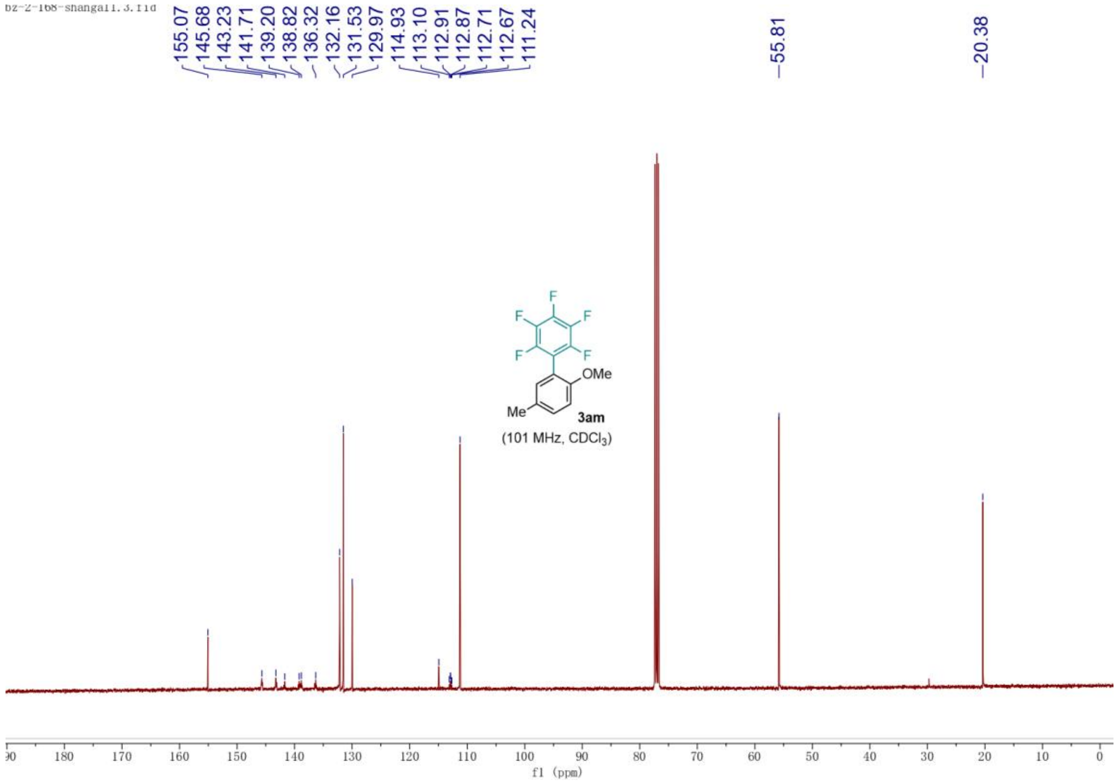
155.07
145.68
143.23
141.71
139.20
138.82
136.32
132.16
131.53
129.97
114.93
113.10
112.91
112.87
112.71
112.67
111.24
55.81
20.38
3am
(101 MHz, CDCl3)
f1 (ppm)

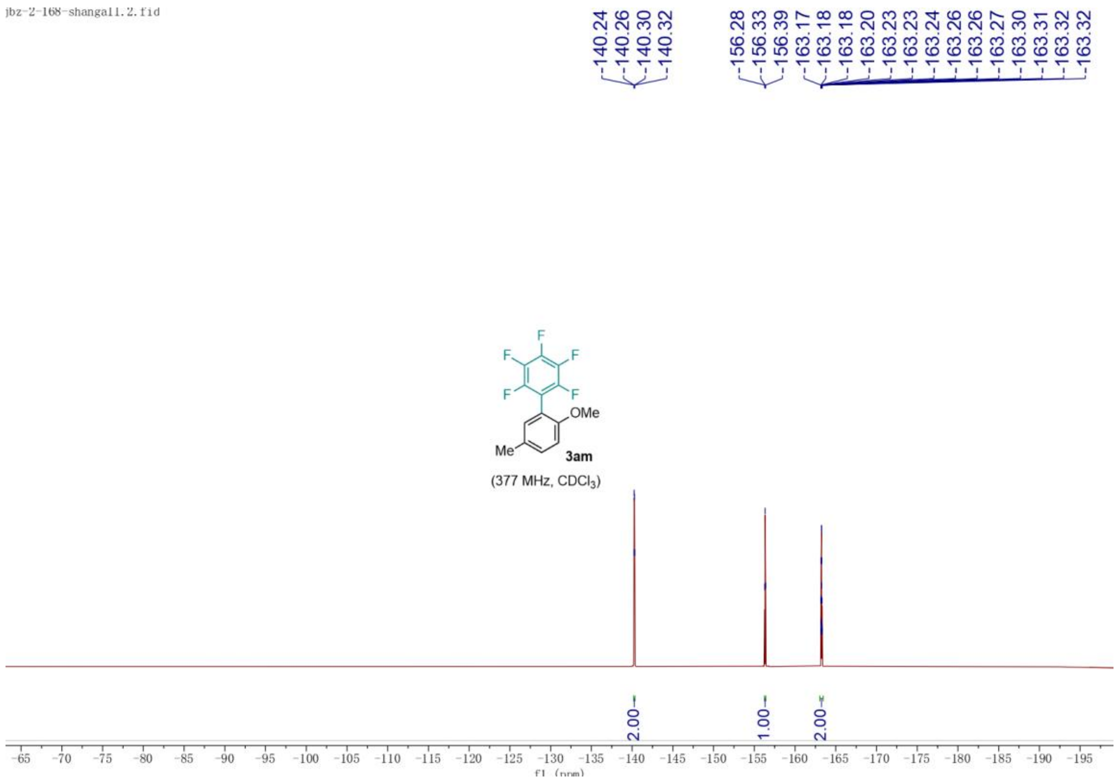
jbz-2-168-shangall.2.fid
-140.24
-140.26
-140.30
-140.32
-156.28
-156.33
-156.39
-163.17
-163.18
-163.18
-163.20
-163.23
-163.23
-163.24
-163.26
-163.26
-163.27
-163.30
-163.31
-163.32
-163.32
OMe
Me
3am
(377 MHz, CDCl3)
2.00
1.00
2.00
f1 (ppm)

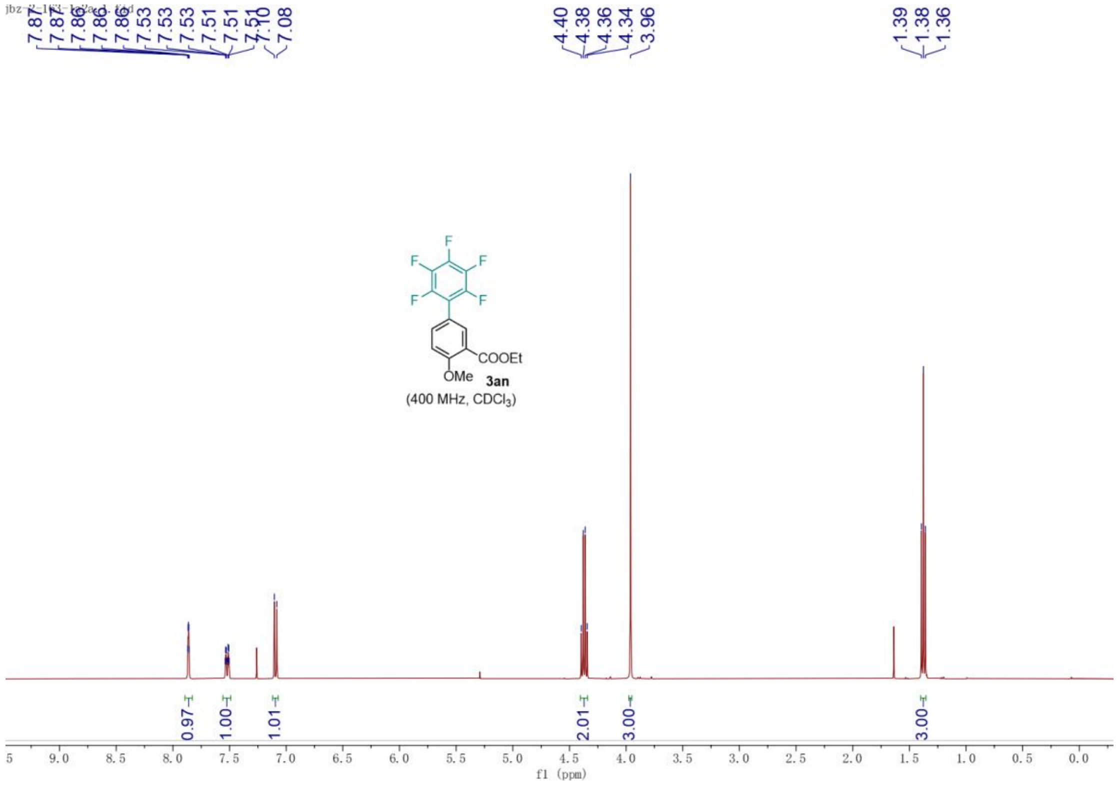
7.87
7.87
7.86
7.86
7.53
7.53
7.53
7.51
7.51
7.10
7.08
4.40
4.38
4.36
4.34
3.96
1.39
1.38
1.36
COOEt
OMe
3an
(400 MHz, CDCl3)
0.97
1.00
1.01
2.01
3.00
3.00
f1 (ppm)

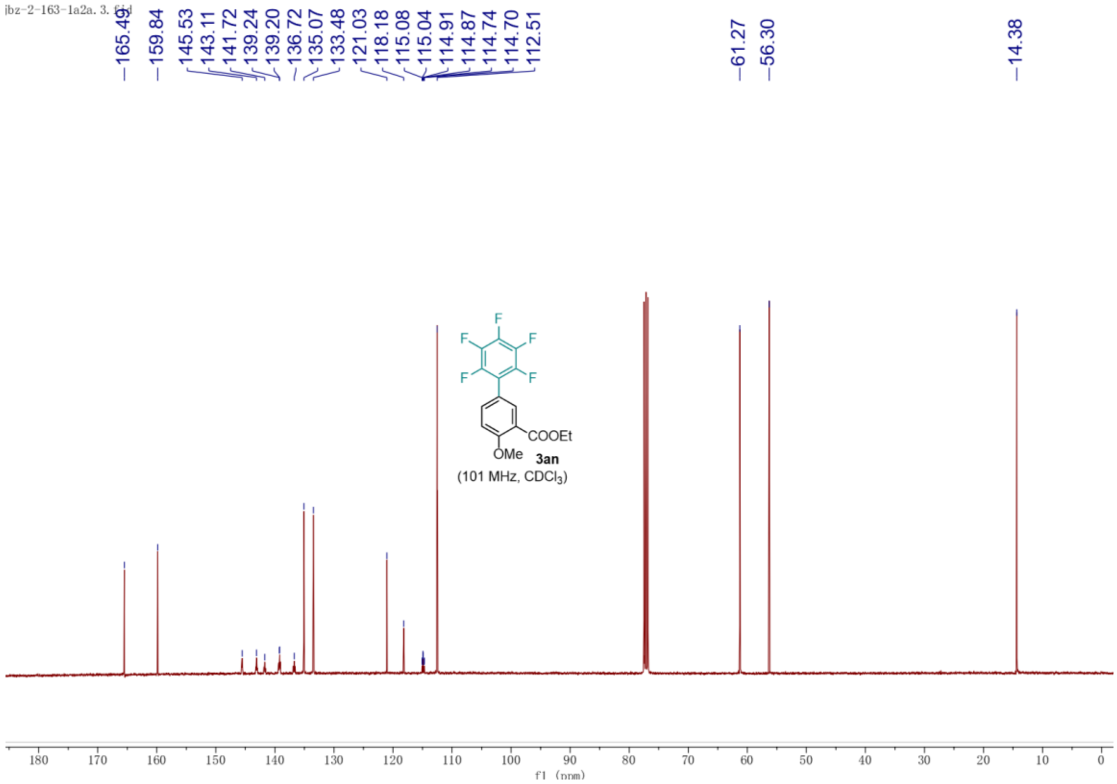
jbz-2-163-1a2a. 3. fid
165.49
159.84
145.53
143.11
141.72
139.24
139.20
136.72
135.07
133.48
121.03
118.18
115.08
115.04
114.91
114.87
114.74
114.70
112.51
61.27
56.30
14.38
COOEt
OMe
3an
(101 MHz, CDCl3)
f1 (ppm)

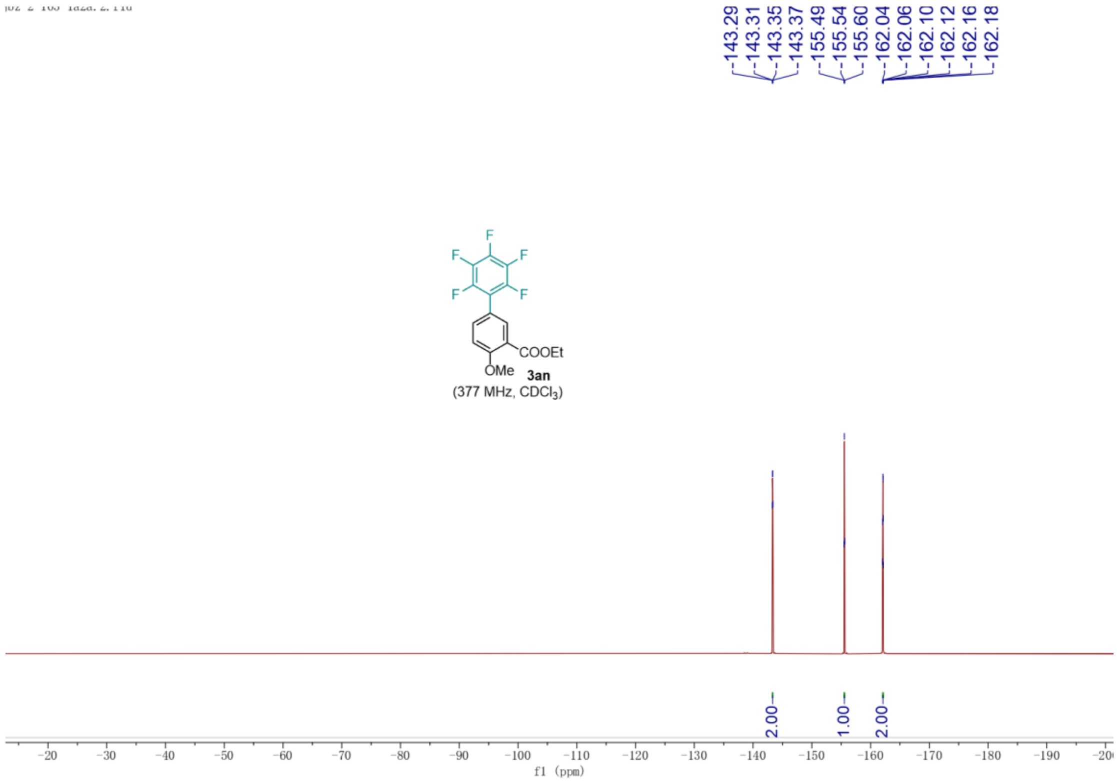
-143.29
-143.31
-143.35
-143.37
-155.49
-155.54
-155.60
-162.04
-162.06
-162.10
-162.12
-162.16
-162.18
COOEt
OMe
3an
(377 MHz, CDCl3)
2.00
1.00
2.00
f1 (ppm)

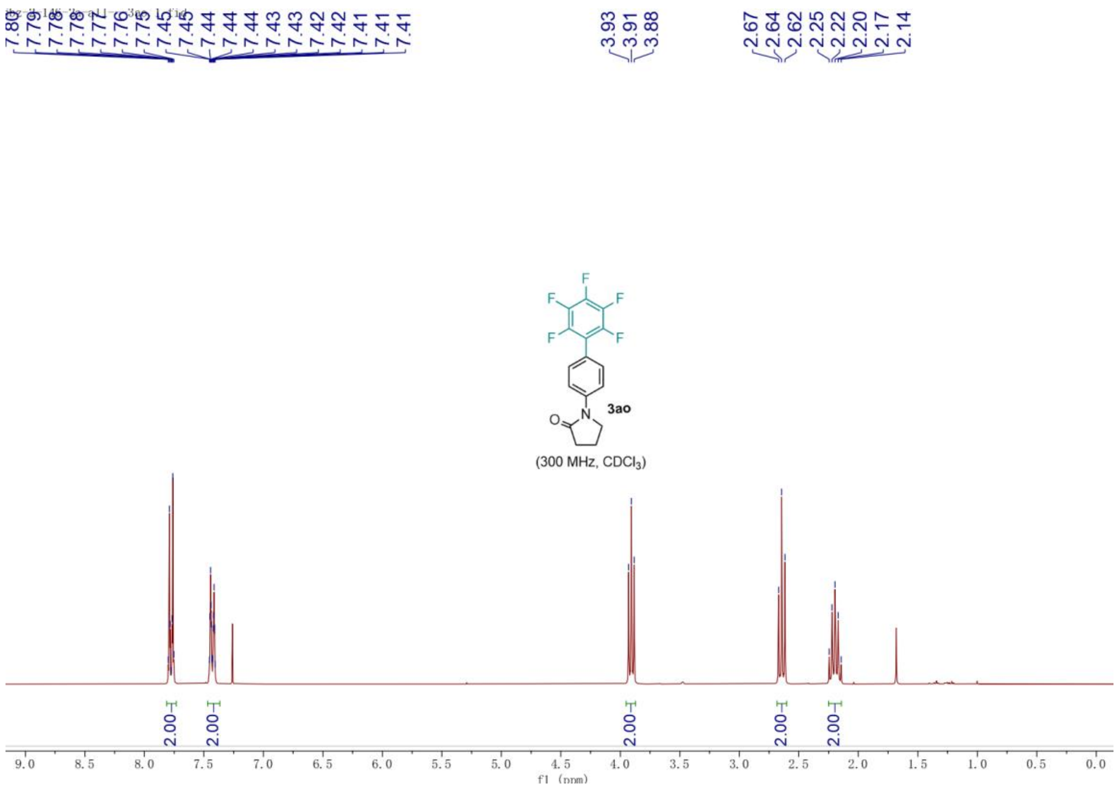
7.80 7.79 7.78 7.78 7.77 7.76 7.75 7.45 7.45 7.44 7.44 7.44 7.43 7.43 7.42 7.42 7.41 7.41 7.41
3.93 3.91 3.88
2.67 2.64 2.62 2.25 2.22 2.20 2.17 2.14
F
F
F
F
F
N
O
3ao
(300 MHz, CDCl3)
2.00
2.00
2.00
2.00
2.00
9.0 8.5 8.0 7.5 7.0 6.5 6.0 5.5 5.0 4.5 4.0 3.5 3.0 2.5 2.0 1.5 1.0 0.5 0.0
f1 (ppm)

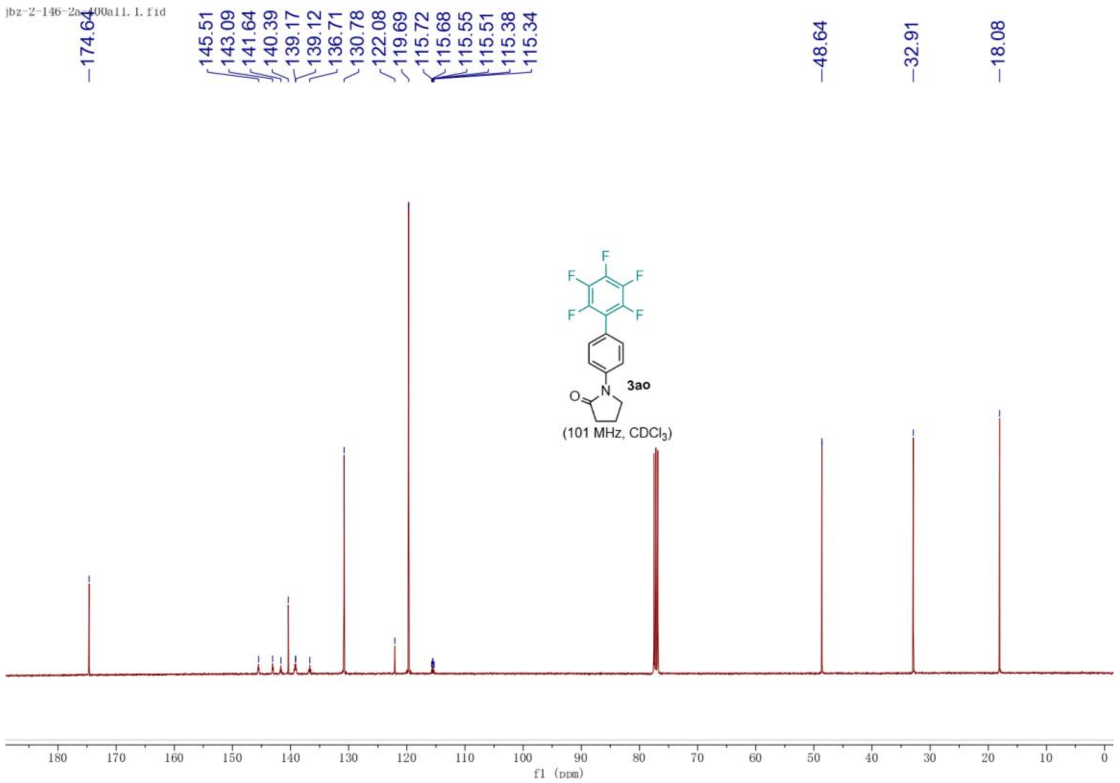
jbz-2-146-2a-400al1.1.fid
174.64
145.51 143.09 141.64 140.39 139.17 139.12 136.71 130.78 122.08 119.69 115.72 115.68 115.55 115.51 115.38 115.34
48.64
32.91
18.08
F
F
F
F
F
N
O
3ao
(101 MHz, CDCl3)
180 170 160 150 140 130 120 110 100 90 80 70 60 50 40 30 20 10 0
f1 (ppm)

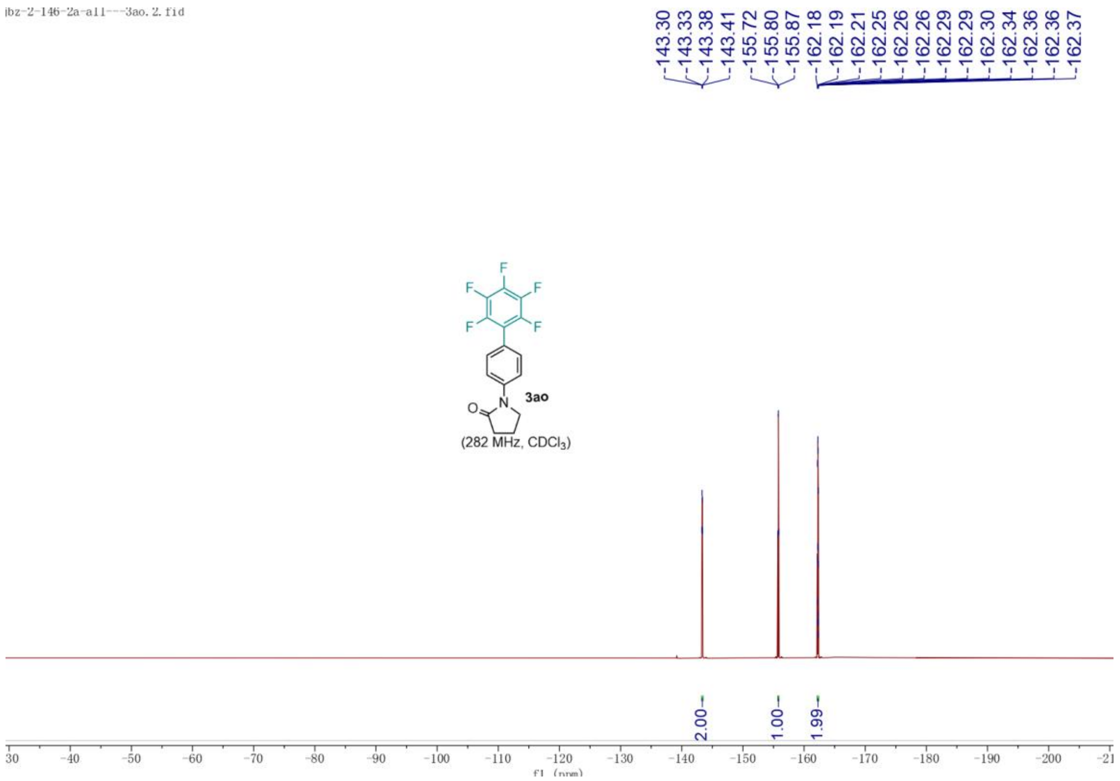
jbz-2-146-2a-all---3ao.2.fid
-143.30
-143.33
-143.38
-143.41
-155.72
-155.80
-155.87
-162.18
-162.19
-162.21
-162.25
-162.26
-162.26
-162.29
-162.29
-162.30
-162.34
-162.36
-162.36
-162.37
3ao
(282 MHz, CDCl3)
2.00
1.00
1.99
f1 (ppm)

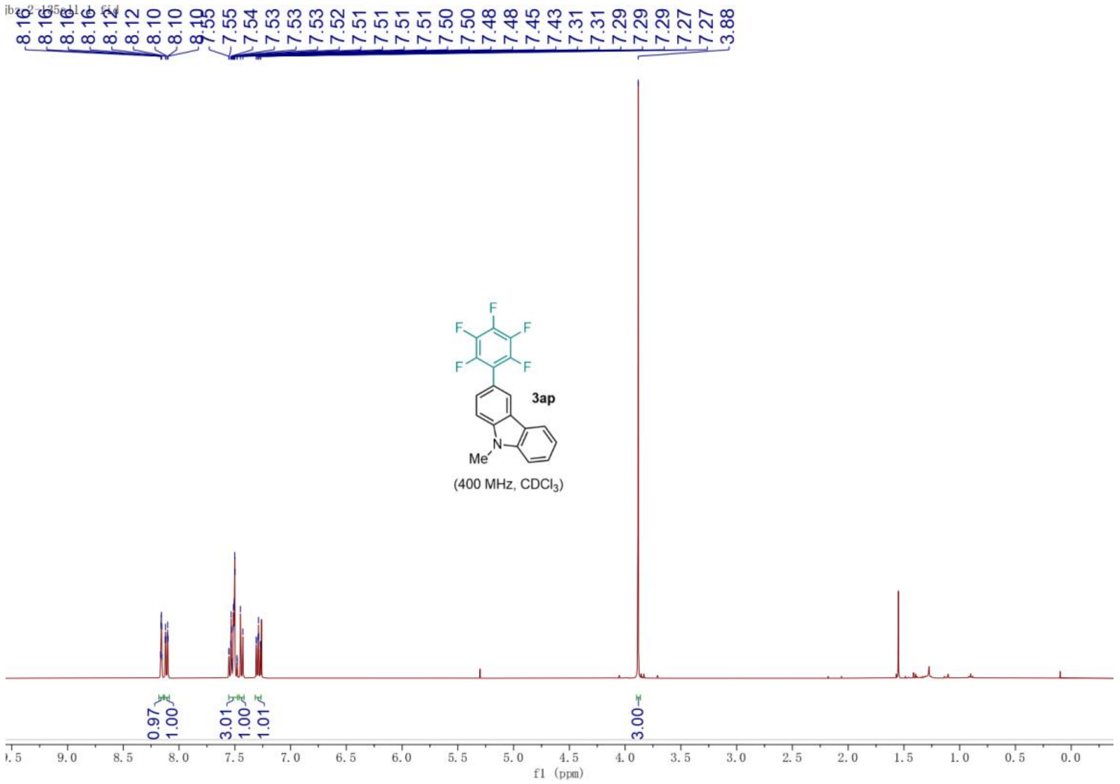
8.16
8.16
8.16
8.16
8.12
8.12
8.10
8.10
8.10
7.55
7.55
7.54
7.53
7.53
7.53
7.52
7.51
7.51
7.51
7.50
7.50
7.48
7.48
7.45
7.43
7.31
7.31
7.29
7.29
7.29
7.27
7.27
3.88
3ap
Me
(400 MHz, CDCl3)
0.97
1.00
3.01
1.00
1.01
3.00
f1 (ppm)

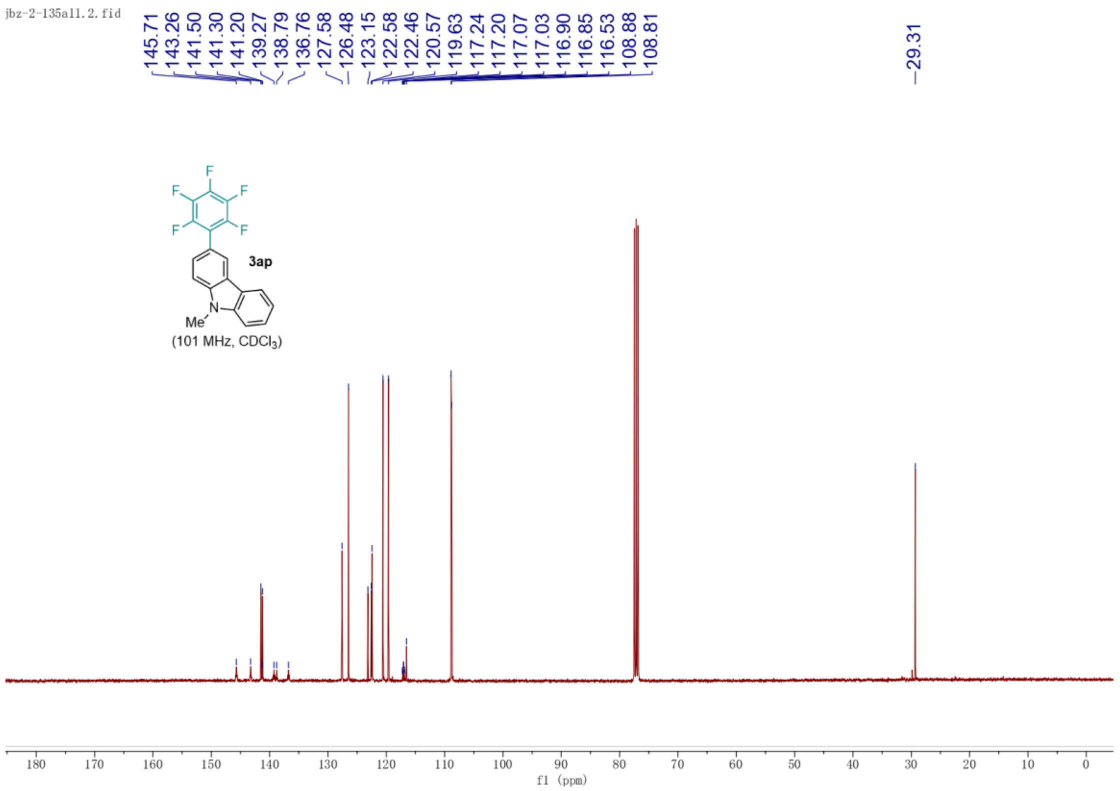
jbz-2-135all.2.fid
145.71
143.26
141.50
141.30
141.20
139.27
138.79
136.76
127.58
126.48
123.15
122.58
122.46
120.57
119.63
117.24
117.20
117.07
117.03
116.90
116.85
116.53
108.88
108.81
29.31
3ap
(101 MHz, CDCl3)
f1 (ppm)

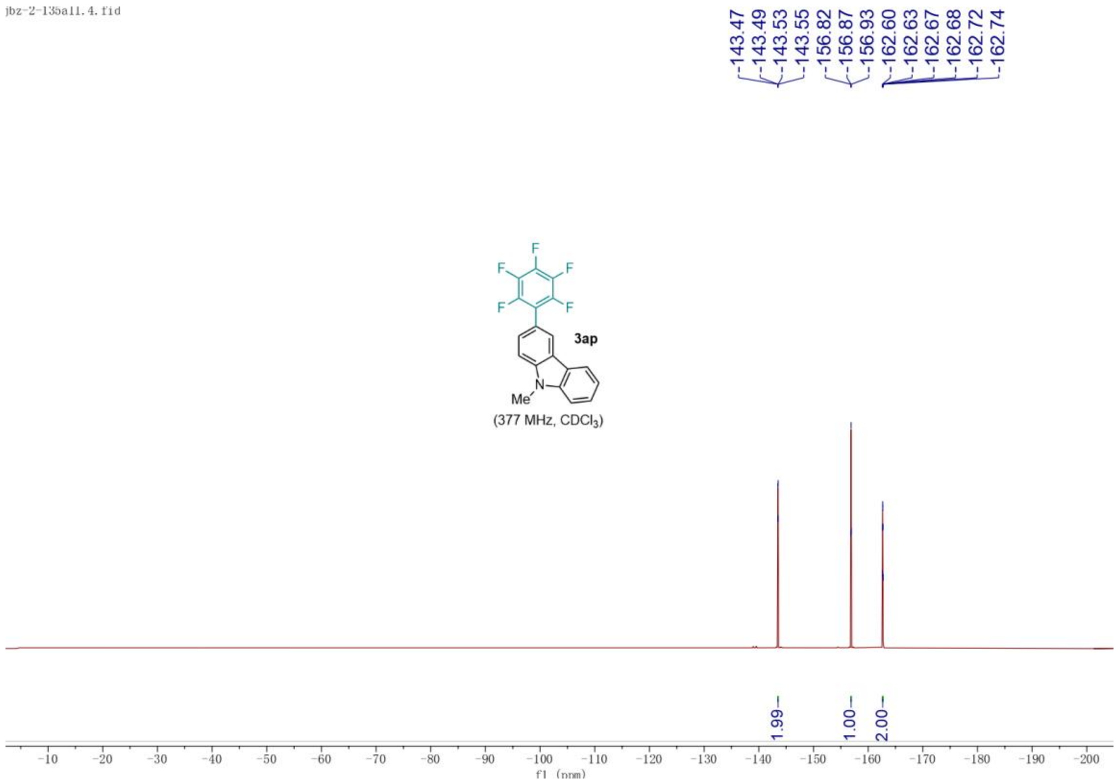
jbz-2-135all.4.fid
-143.47
-143.49
-143.53
-143.55
-156.82
-156.87
-156.93
-162.60
-162.63
-162.67
-162.68
-162.72
-162.74
3ap
(377 MHz, CDCl3)
1.99
1.00
2.00
f1 (ppm)

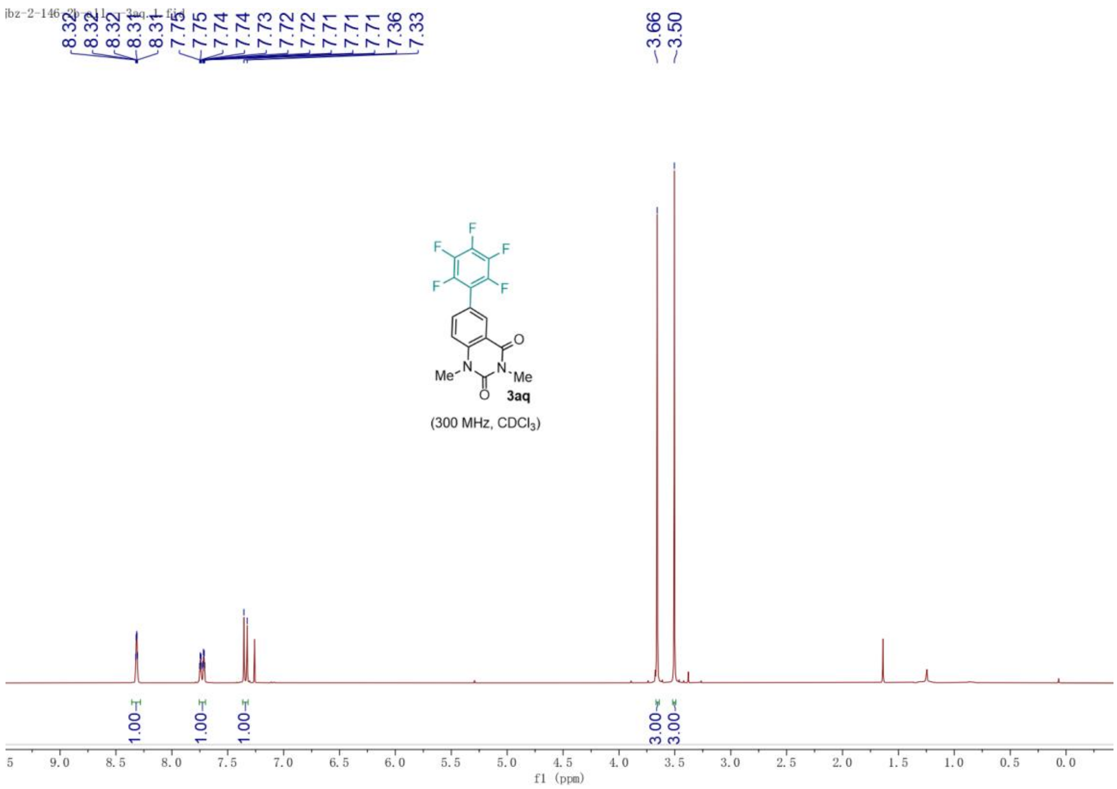

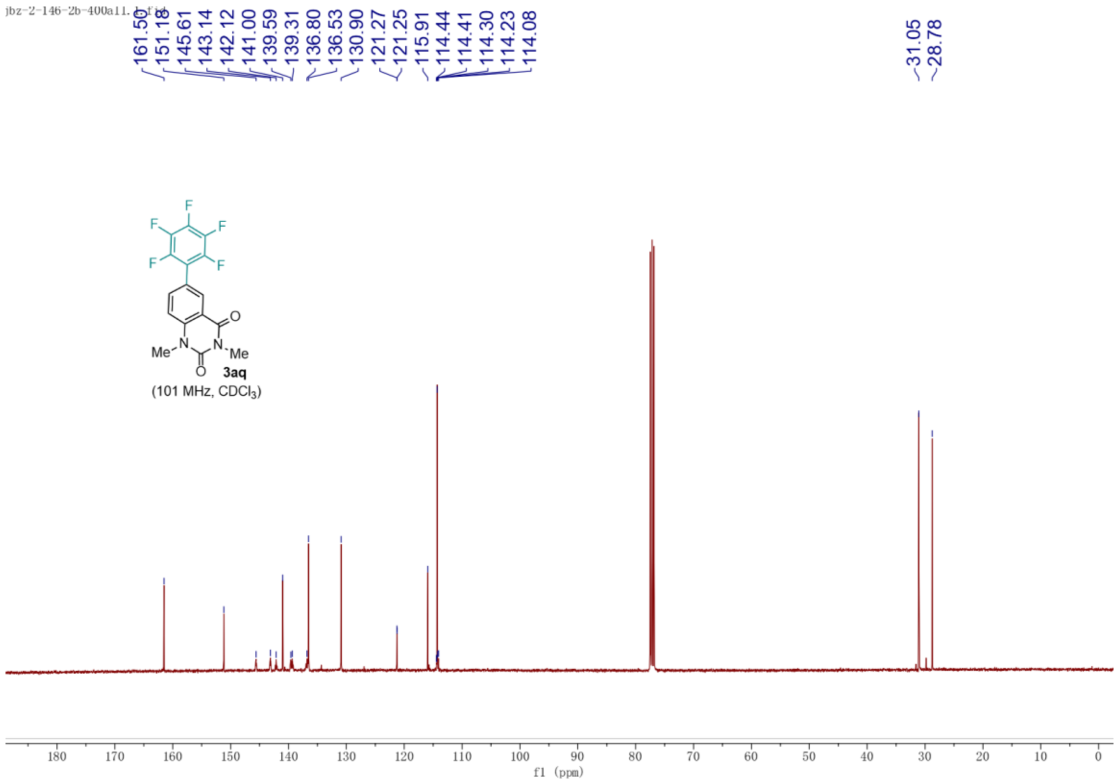

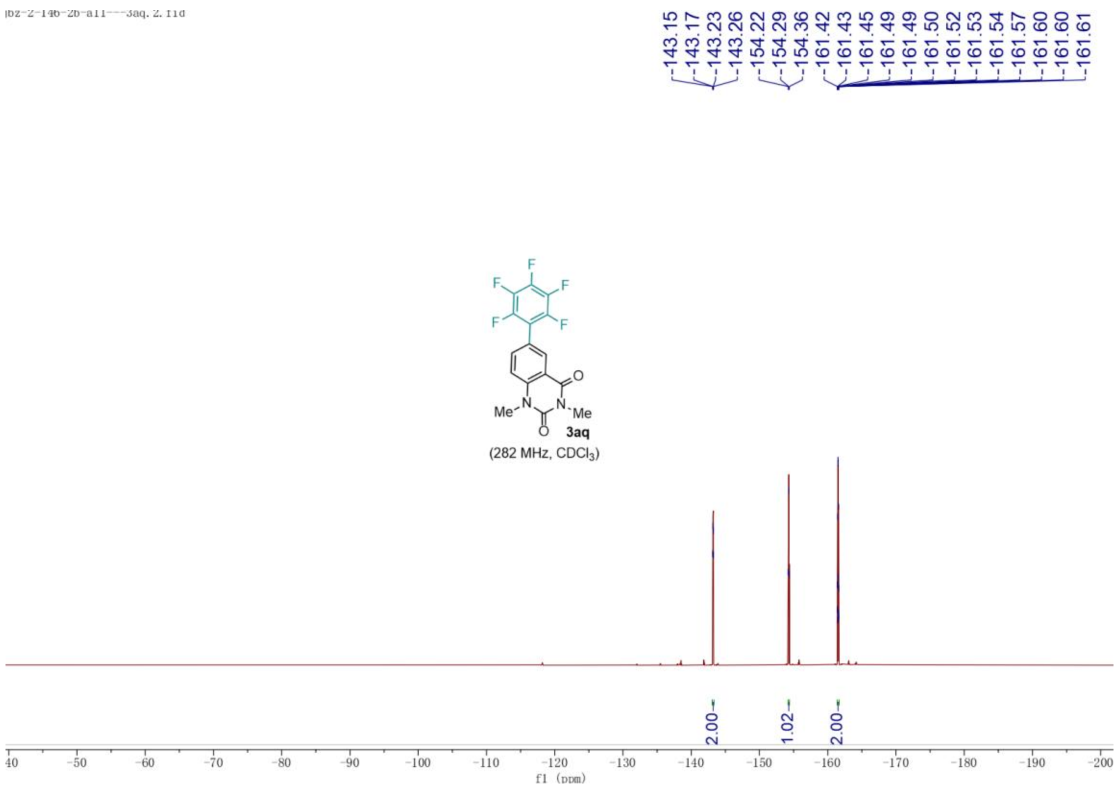

143.15
143.17
143.23
143.26
154.22
154.29
154.36
161.42
161.43
161.45
161.49
161.50
161.52
161.53
161.54
161.57
161.60
161.60
161.61
F
F
F
F
F
O
N
Me
N
Me
O
3aq
(282 MHz, CDCl3)
2.00
1.02
2.00
f1 (ppm)


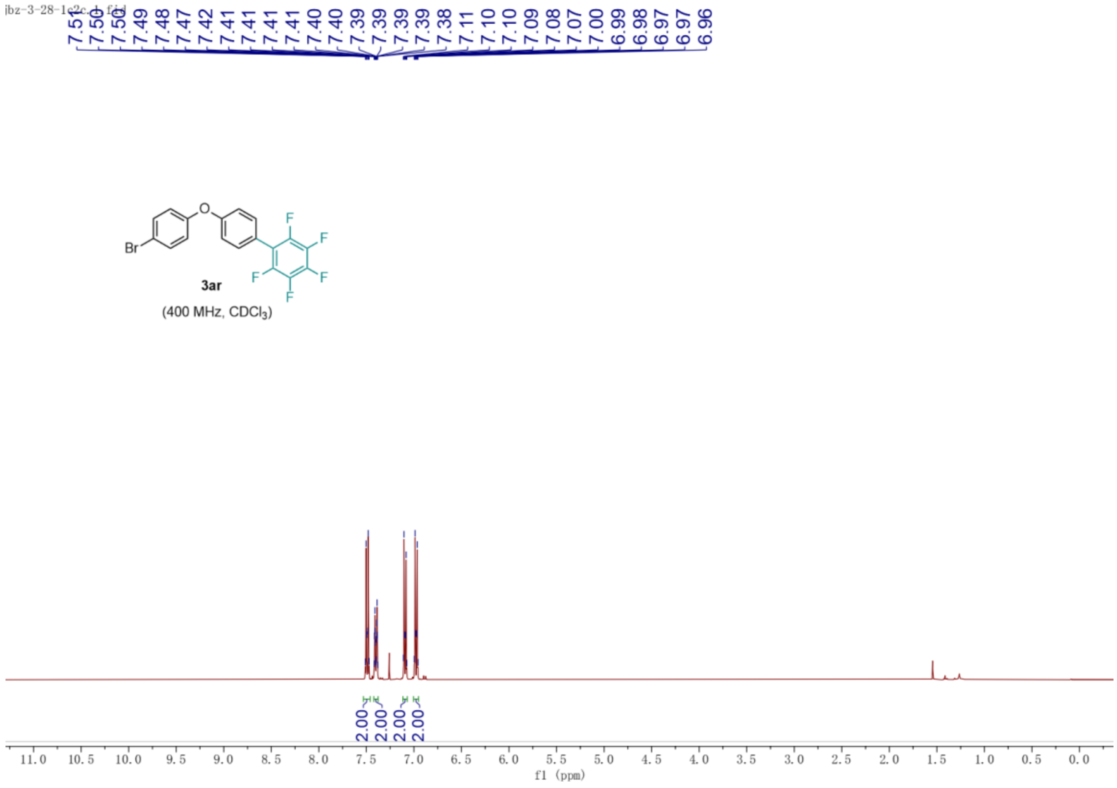

7.51
7.50
7.50
7.49
7.48
7.47
7.42
7.41
7.41
7.41
7.41
7.40
7.40
7.39
7.39
7.39
7.39
7.38
7.11
7.10
7.10
7.09
7.08
7.07
7.00
6.99
6.98
6.97
6.97
6.96
Br
O
F
F
F
F
F
3ar
(400 MHz, CDCl3)
2.00
2.00
2.00
2.00
f1 (ppm)

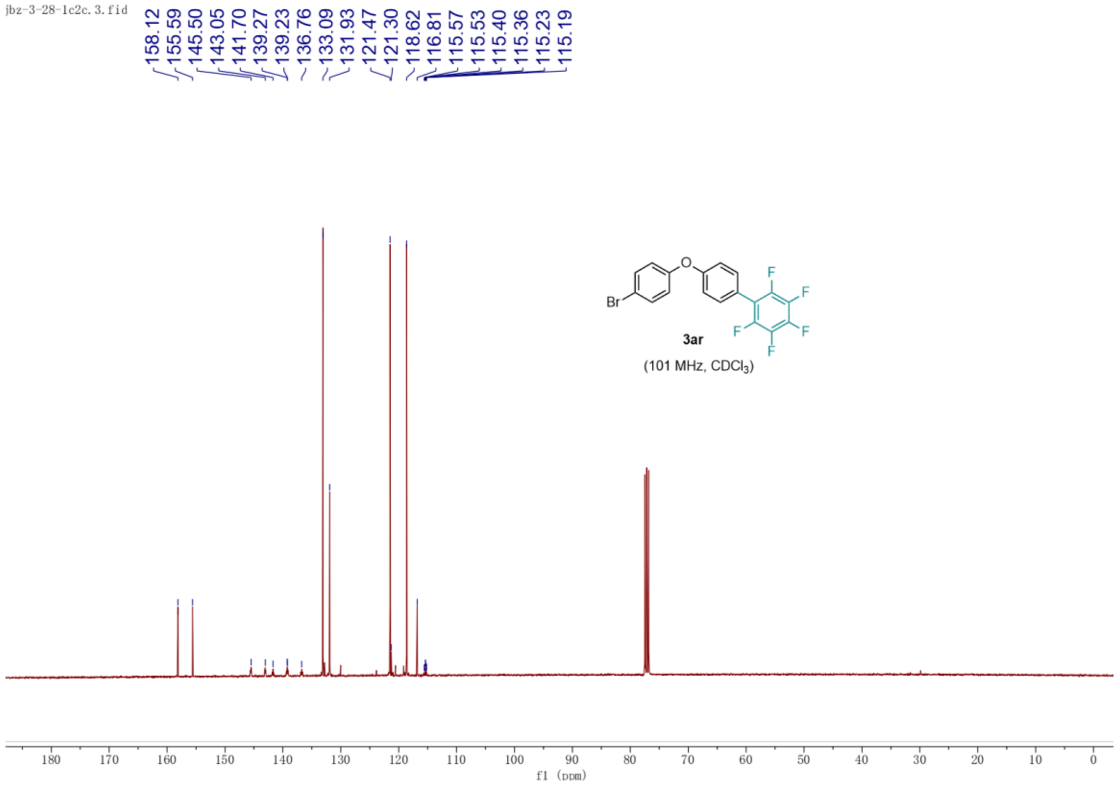
jbz-3-28-1c2c.3.fid
158.12
155.59
145.50
143.05
141.70
139.27
139.23
136.76
133.09
131.93
121.47
121.30
118.62
116.81
115.57
115.53
115.40
115.36
115.23
115.19
Br
O
F
F
F
F
F
3ar
(101 MHz, CDCl3)
f1 (ppm)

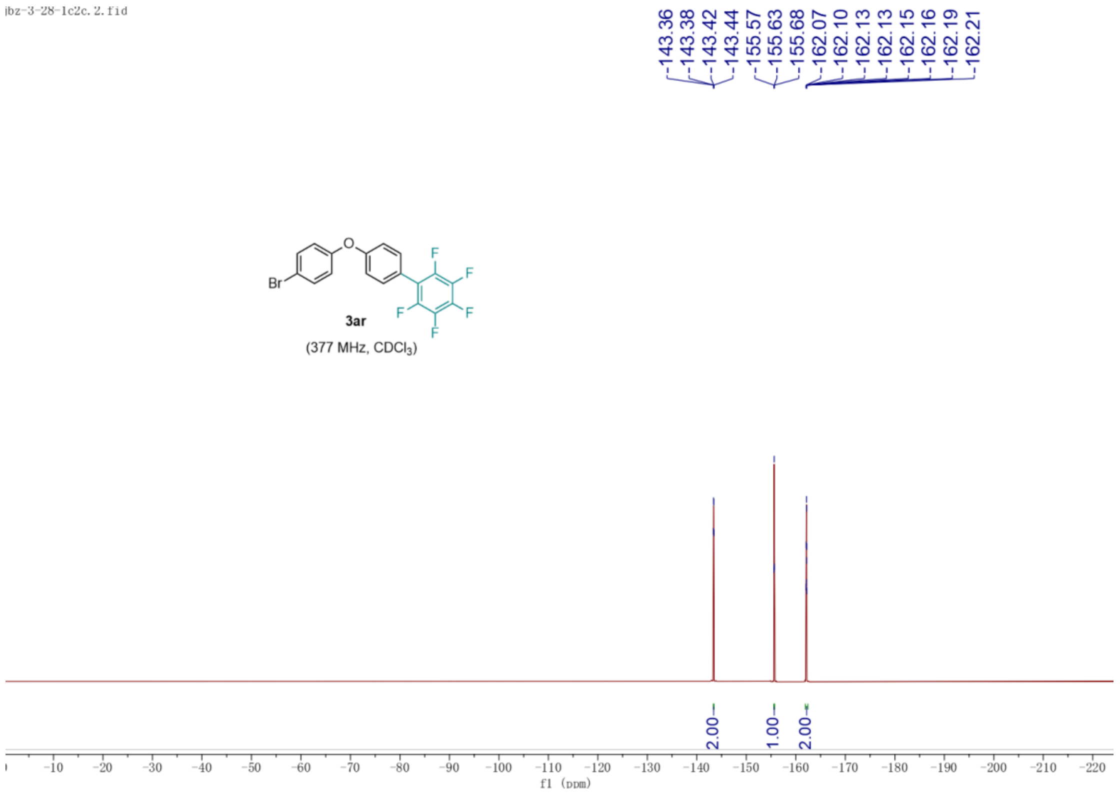
jbz-3-28-1c2c.2.fid
-143.36
-143.38
-143.42
-143.44
-155.57
-155.63
-155.68
-162.07
-162.10
-162.13
-162.13
-162.15
-162.16
-162.19
-162.21
Br
O
F
F
F
F
F
3ar
(377 MHz, CDCl3)
2.00
1.00
2.00
f1 (ppm)

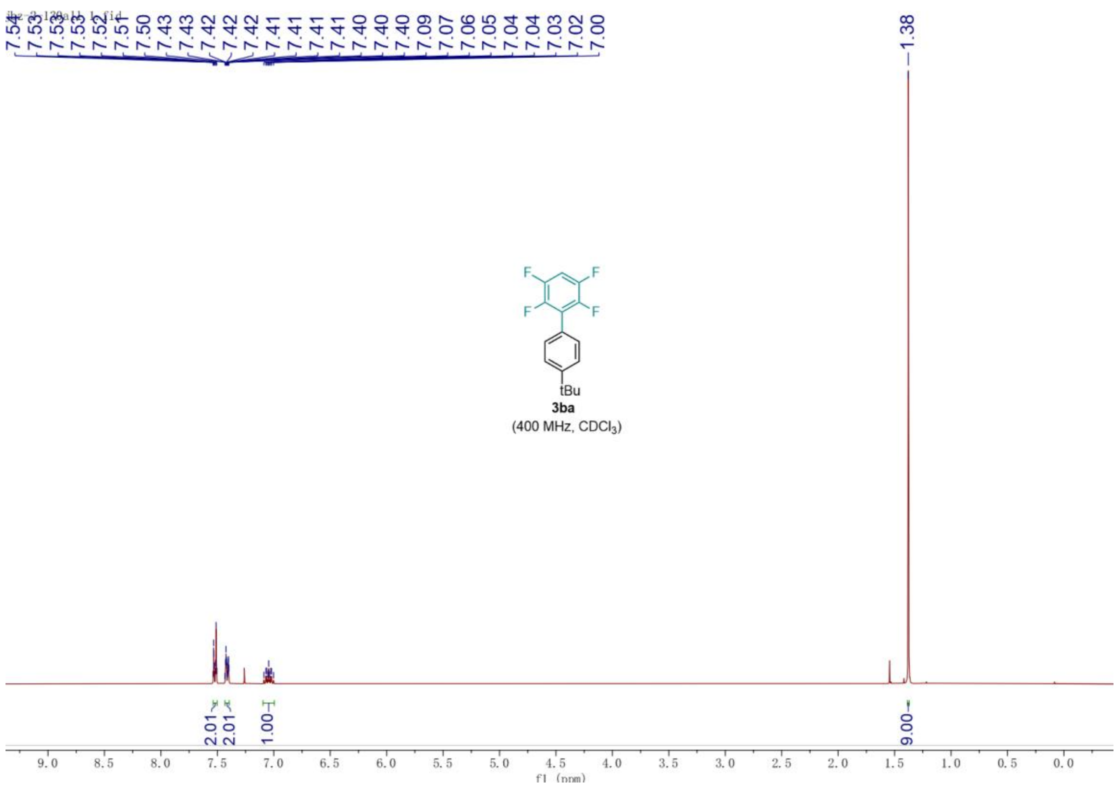
3ba
(400 MHz, CDCl$_3$)
7.54 7.53 7.53 7.53 7.52 7.51 7.50 7.43 7.43 7.42 7.42 7.41 7.41 7.41 7.40 7.40 7.09 7.07 7.06 7.05 7.04 7.04 7.03 7.02 7.00
1.38
2.01 2.01 1.00 9.00
f1 (ppm)

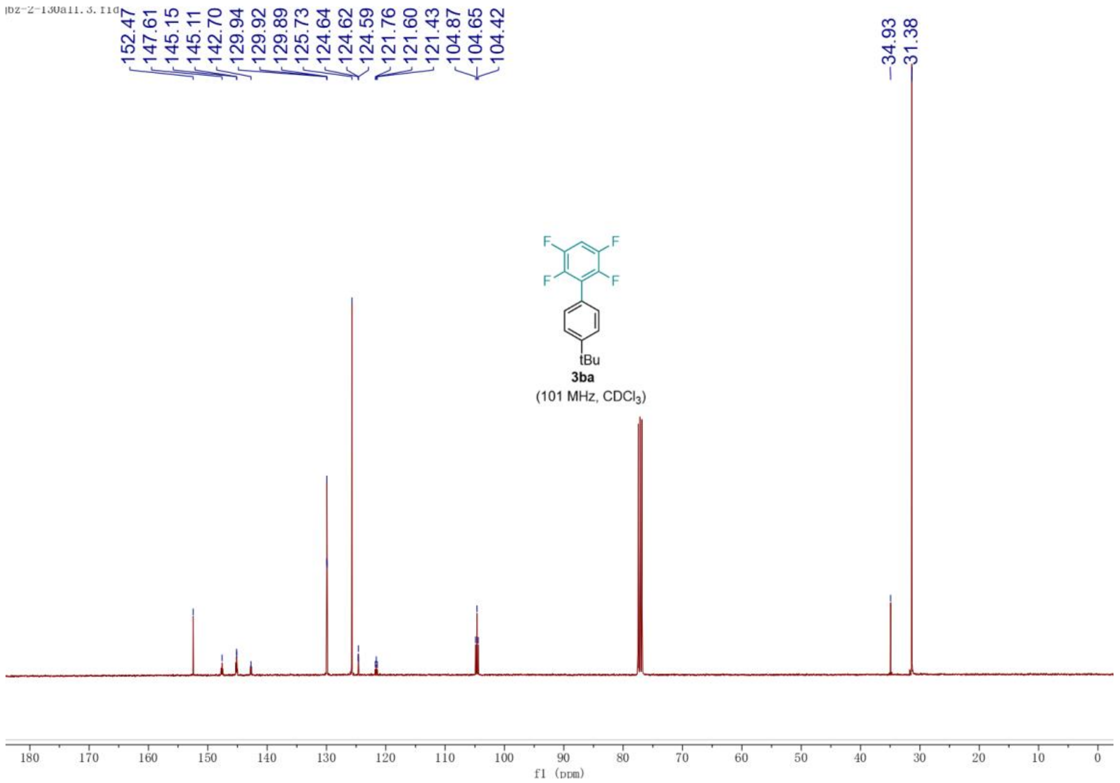
3ba
(101 MHz, CDCl$_3$)
152.47 147.61 145.15 145.11 142.70 129.94 129.92 129.89 125.73 124.64 124.62 124.59 121.76 121.60 121.43 104.87 104.65 104.42
34.93 31.38
f1 (ppm)

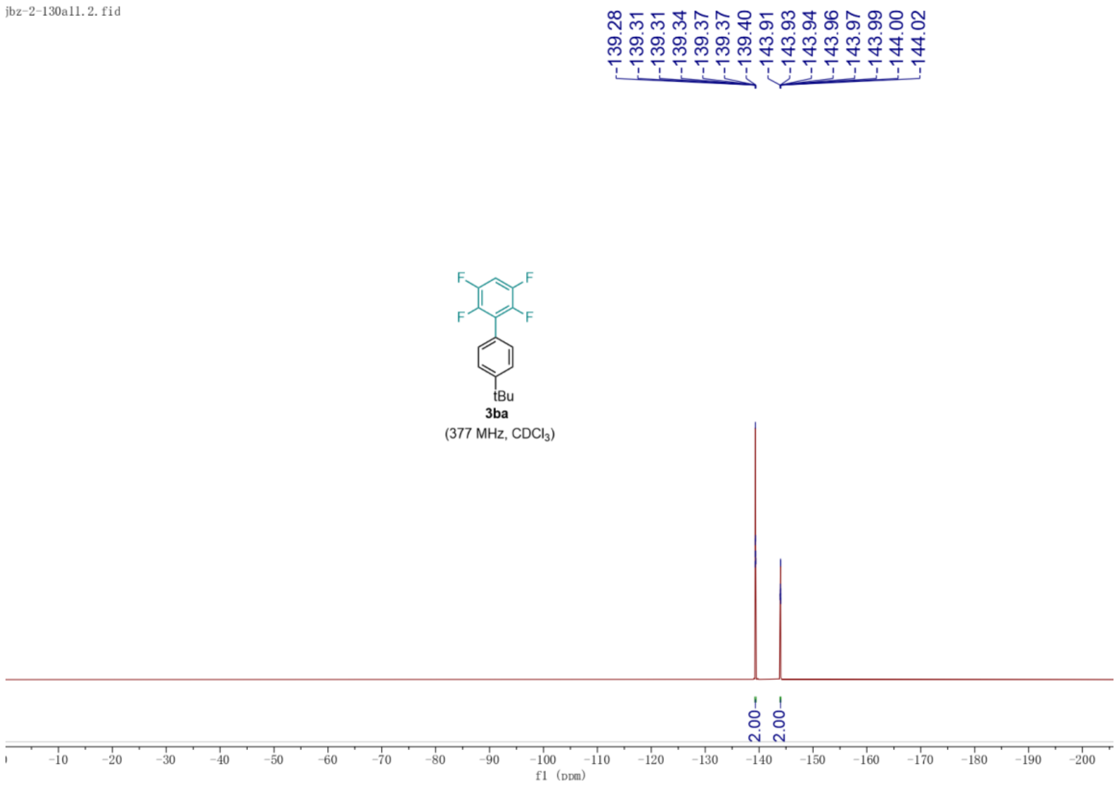
jbz-2-130all.2.fid
139.28
139.31
139.31
139.34
139.37
139.37
139.40
143.91
143.93
143.94
143.96
143.97
143.99
144.00
144.02
tBu
3ba
(377 MHz, CDCl3)
2.00
2.00
f1 (ppm)

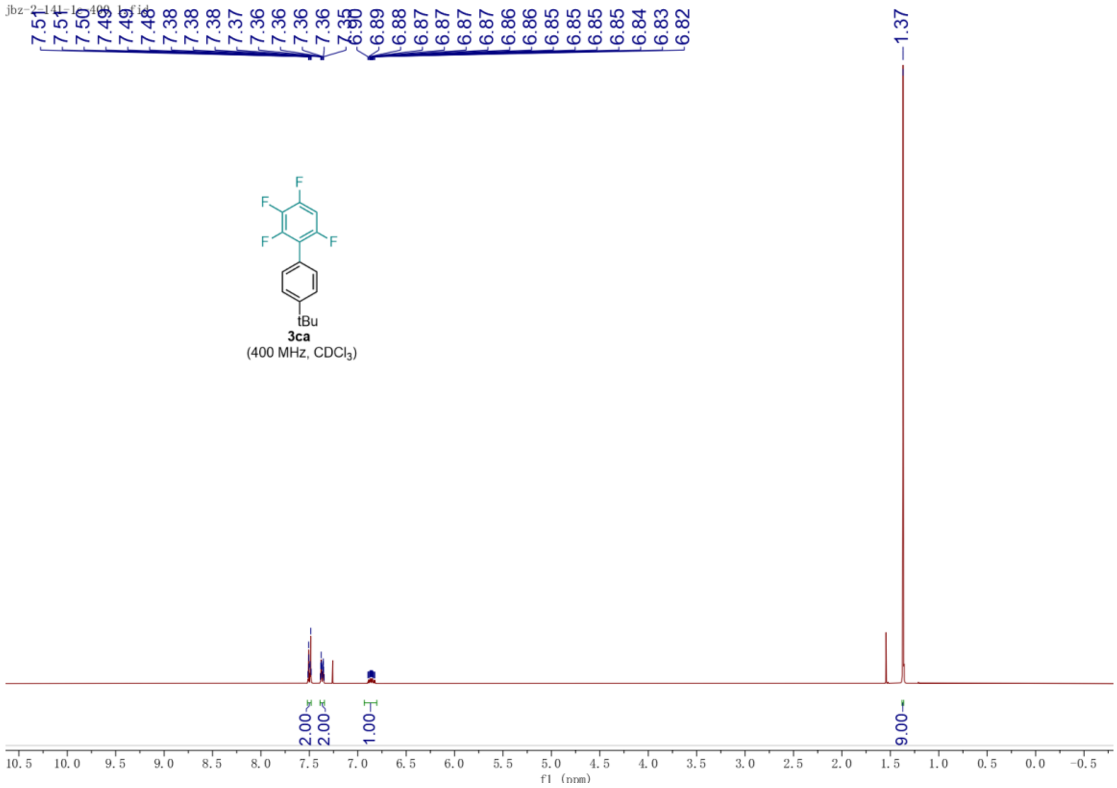
7.51
7.51
7.50
7.49
7.49
7.48
7.38
7.38
7.38
7.37
7.36
7.36
7.36
7.36
7.35
6.90
6.89
6.88
6.87
6.87
6.87
6.86
6.86
6.85
6.85
6.85
6.84
6.83
6.82
1.37
tBu
3ca
(400 MHz, CDCl3)
2.00
2.00
1.00
9.00
f1 (ppm)

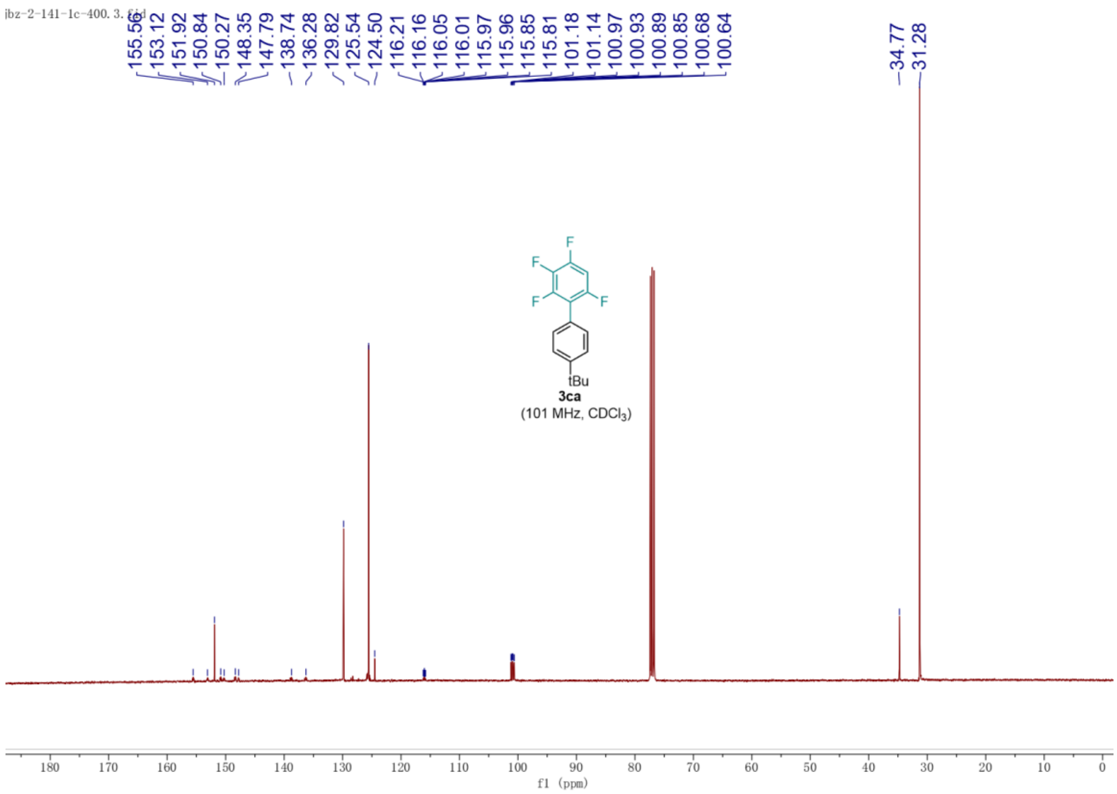

jbz-2-141-1c-400.3.fid
155.56
153.12
151.92
150.84
150.27
148.35
147.79
138.74
136.28
129.82
125.54
124.50
116.21
116.16
116.05
116.01
115.97
115.96
115.85
115.81
101.18
101.14
100.97
100.93
100.89
100.85
100.68
100.64
34.77
31.28
F
F
F
F
tBu
3ca
(101 MHz, CDCl3)
f1 (ppm)


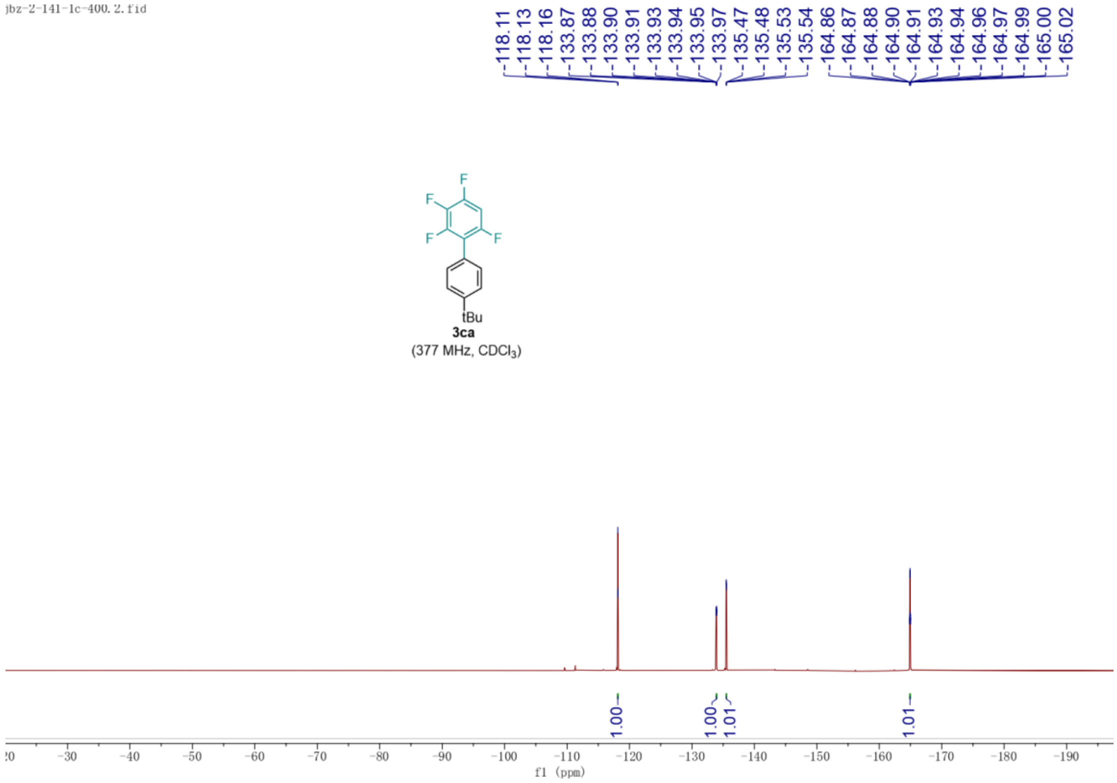

jbz-2-141-1c-400.2.fid
-118.11
-118.13
-118.16
-133.87
-133.88
-133.90
-133.91
-133.93
-133.94
-133.95
-133.97
-135.47
-135.48
-135.53
-135.54
-164.86
-164.87
-164.88
-164.90
-164.91
-164.93
-164.94
-164.96
-164.97
-164.99
-165.00
-165.02
F
F
F
F
tBu
3ca
(377 MHz, CDCl3)
1.00
1.00
1.01
1.01
f1 (ppm)

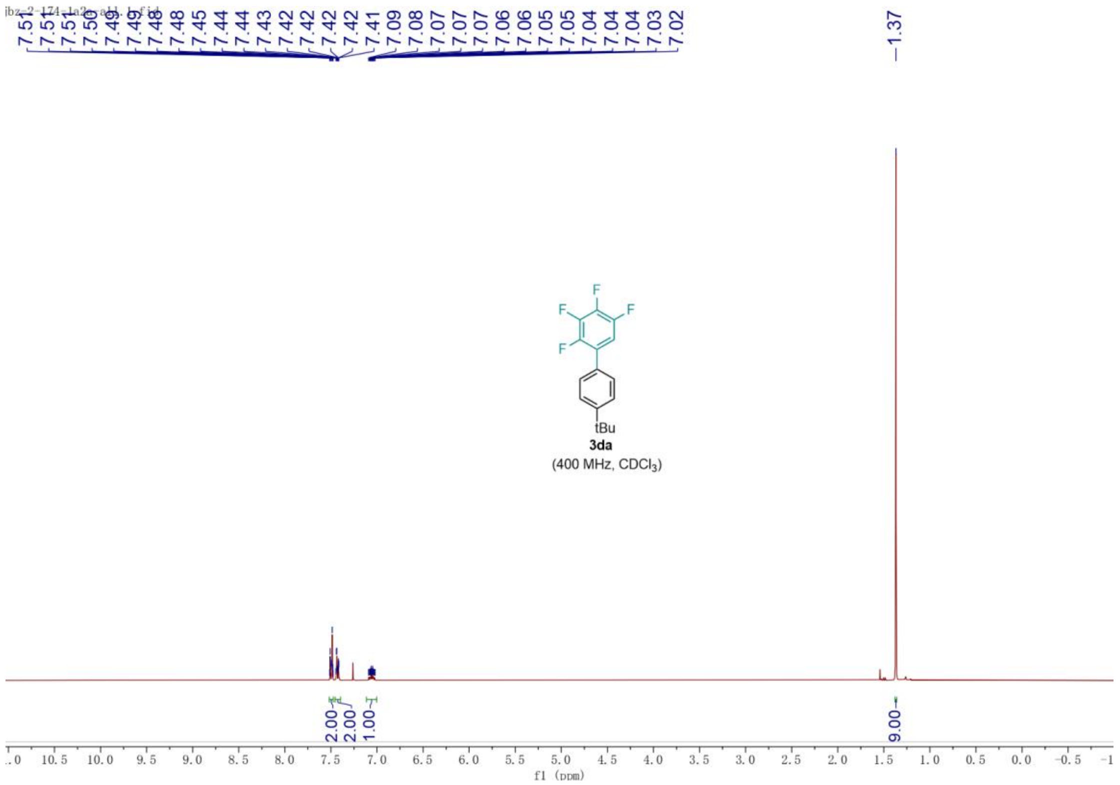
7.51 7.51 7.50 7.49 7.49 7.48 7.48 7.45 7.44 7.44 7.43 7.42 7.42 7.42 7.42 7.41 7.09 7.08 7.07 7.07 7.07 7.06 7.06 7.05 7.05 7.04 7.04 7.04 7.03 7.02
1.37
F
tBu
3da
(400 MHz, CDCl3)
2.00
2.00
1.00
9.00
f1 (ppm)

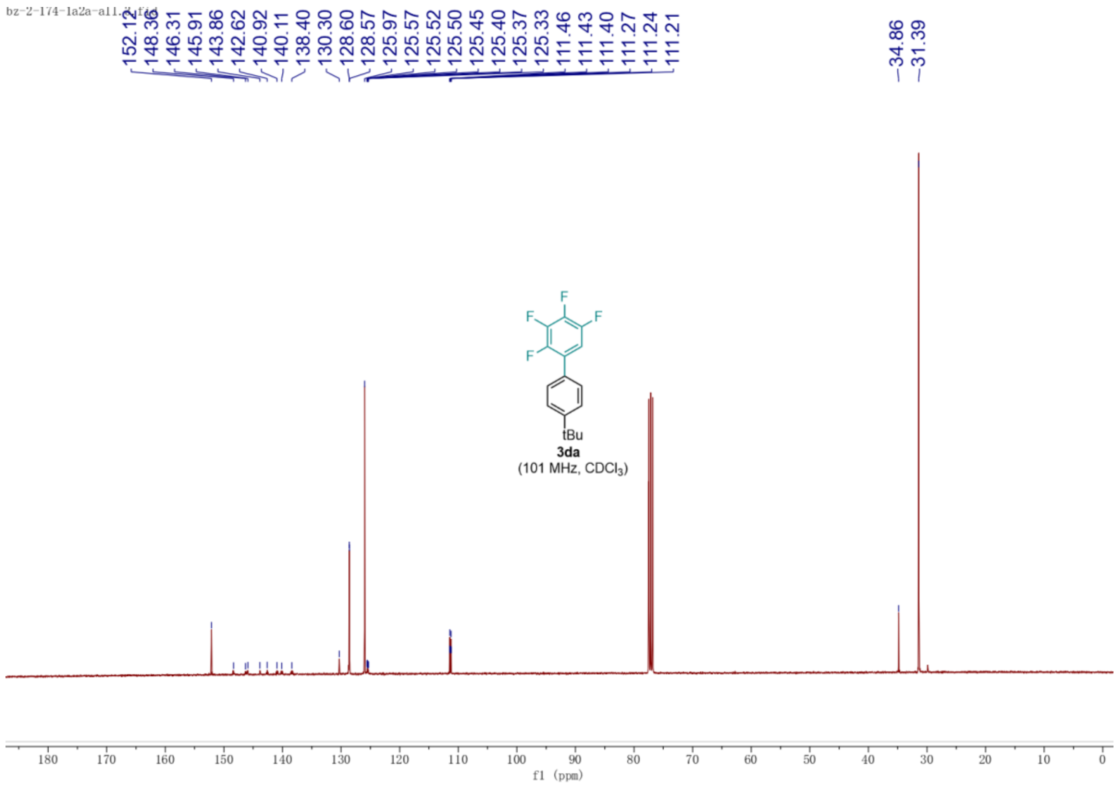
152.12 148.36 146.31 145.91 143.86 142.62 140.92 140.11 138.40 130.30 128.60 128.57 125.97 125.57 125.52 125.50 125.45 125.40 125.37 125.33 111.46 111.43 111.40 111.27 111.24 111.21
34.86
31.39
F
tBu
3da
(101 MHz, CDCl3)
f1 (ppm)

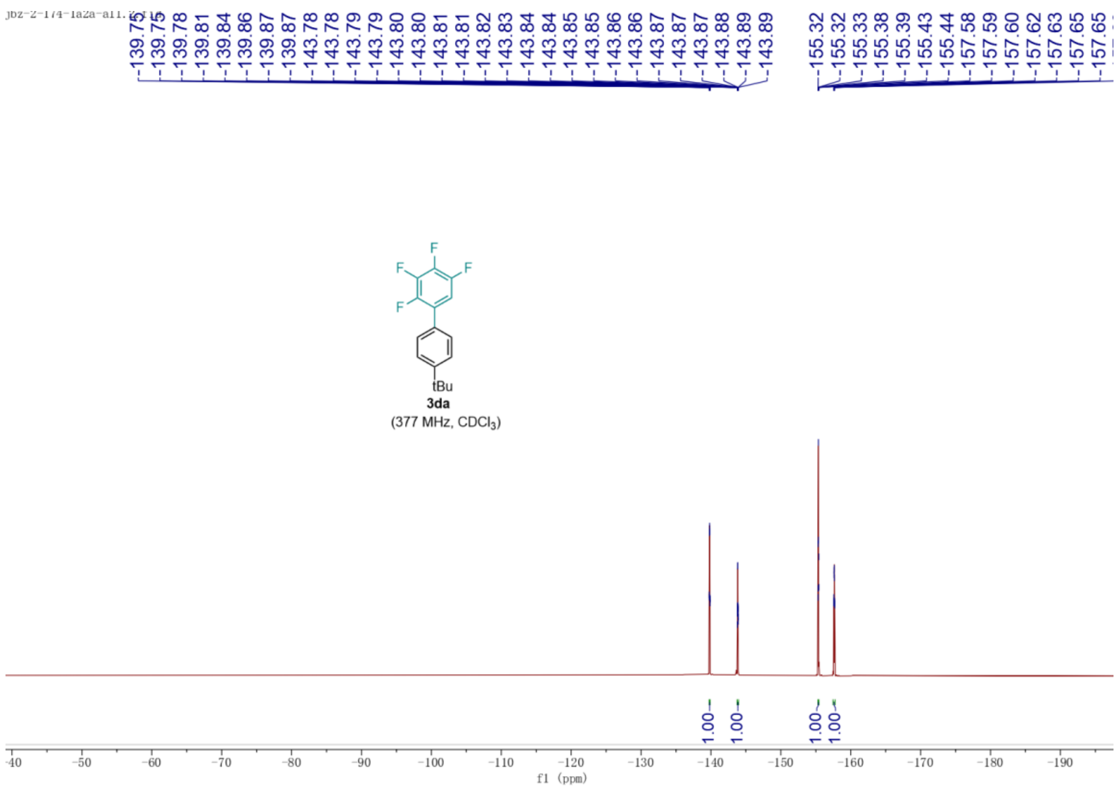

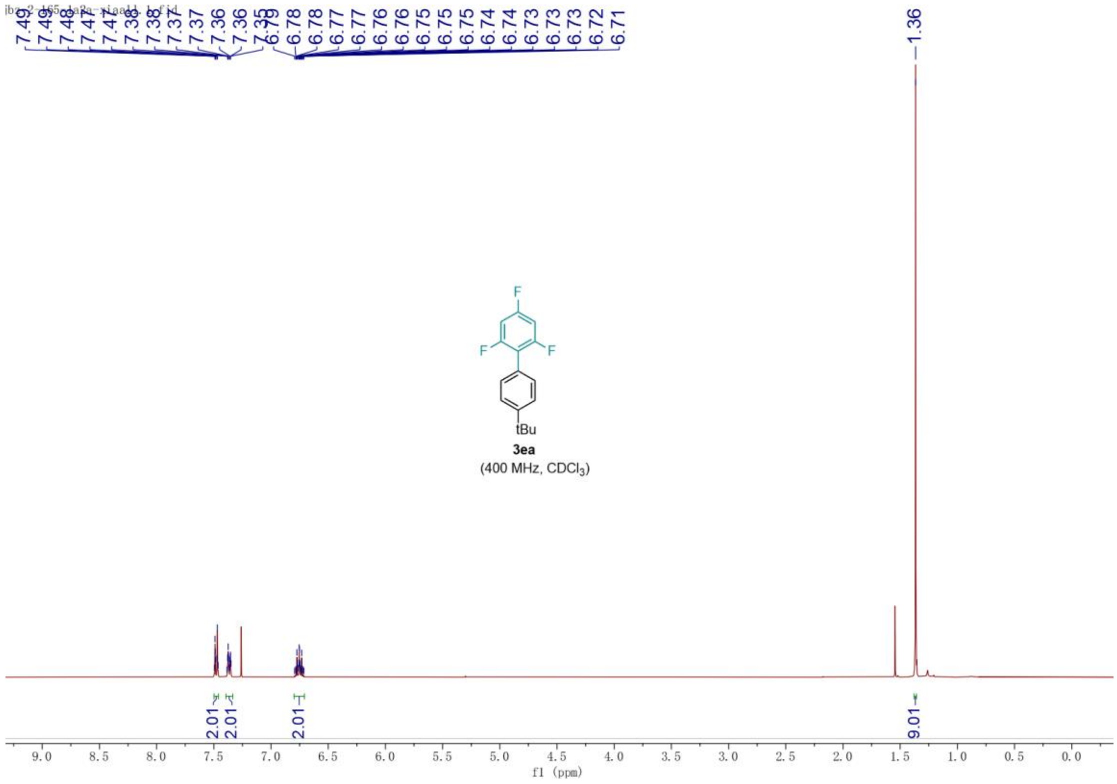

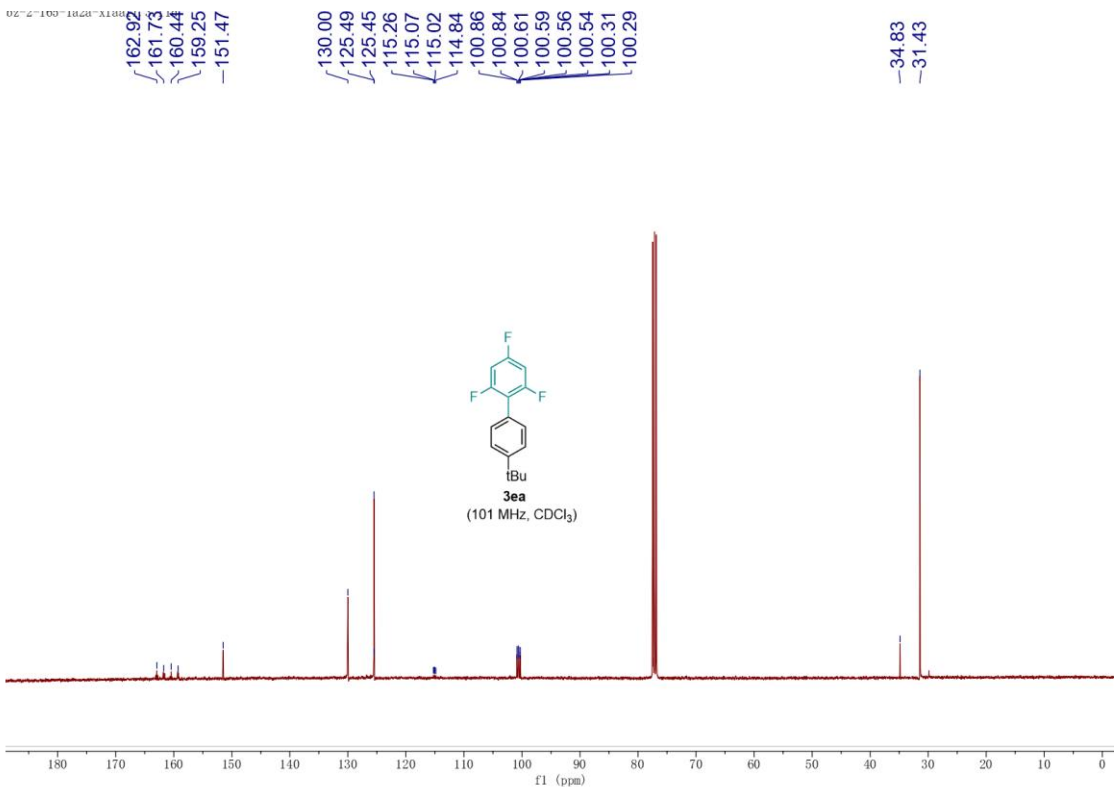
162.92
161.73
160.44
159.25
151.47
130.00
125.49
125.45
115.26
115.07
115.02
114.84
100.86
100.84
100.61
100.59
100.56
100.54
100.31
100.29
34.83
31.43
F
F
F
tBu
3ea
(101 MHz, CDCl3)
f1 (ppm)

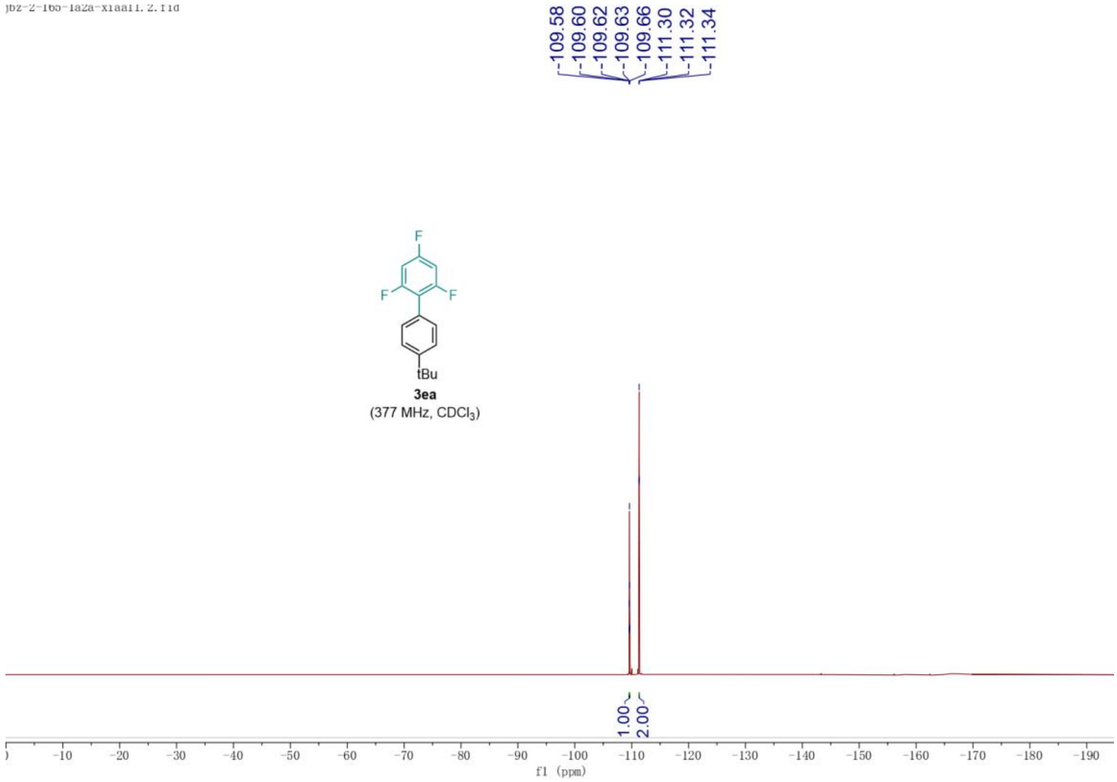
-109.58
-109.60
-109.62
-109.63
-109.66
-111.30
-111.32
-111.34
F
F
F
tBu
3ea
(377 MHz, CDCl3)
1.00
2.00
f1 (ppm)

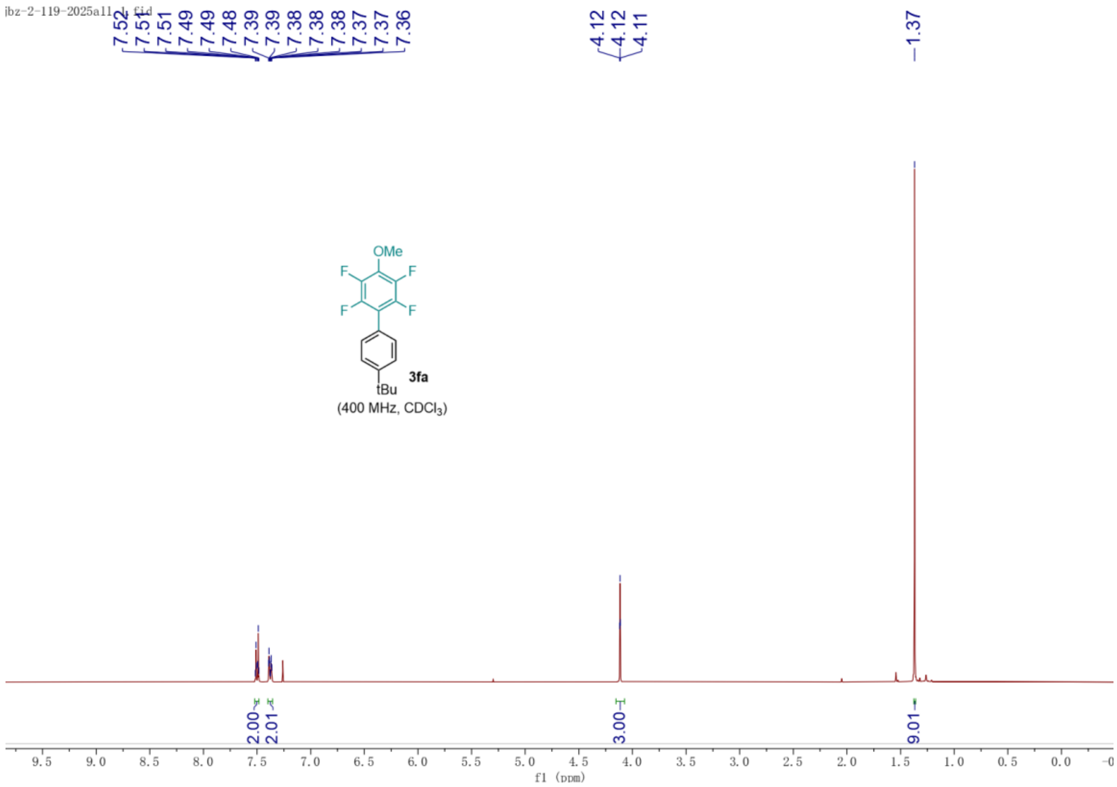
jbz-2-119-2025all.1.fid
7.52 7.51 7.51 7.49 7.49 7.48 7.39 7.39 7.38 7.38 7.38 7.37 7.37 7.36
4.12 4.12 4.11
1.37
OMe
F
F
F
F
tBu
3fa
(400 MHz, CDCl$_3$)
2.00 2.01
3.00
9.01
f1 (ppm)

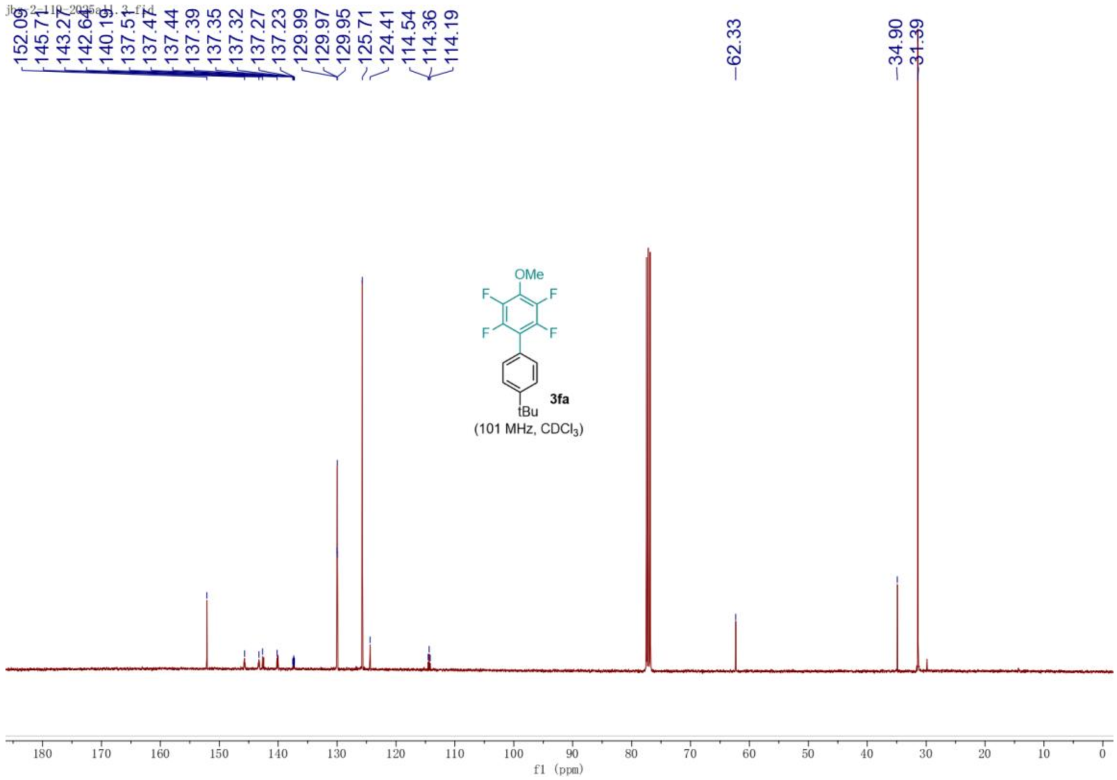
152.09 145.71 143.27 142.64 140.19 137.51 137.47 137.44 137.39 137.35 137.32 137.27 137.23 129.99 129.97 129.95 125.71 124.41 114.54 114.36 114.19
62.33
34.90 31.39
OMe
F
F
F
F
tBu
3fa
(101 MHz, CDCl$_3$)
f1 (ppm)

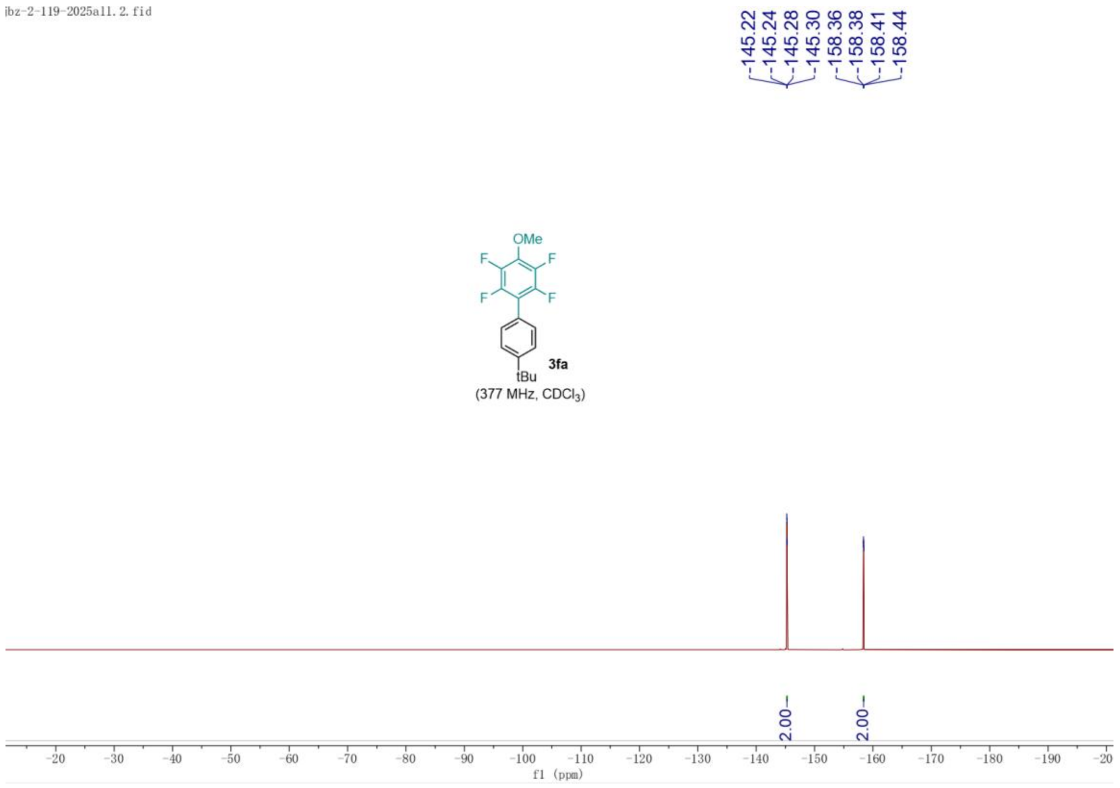
jbz-2-119-2025all.2.fid
-145.22
-145.24
-145.28
-145.30
-158.36
-158.38
-158.41
-158.44
OMe
F
F
F
F
tBu
3fa
(377 MHz, CDCl3)
2.00
2.00
f1 (ppm)

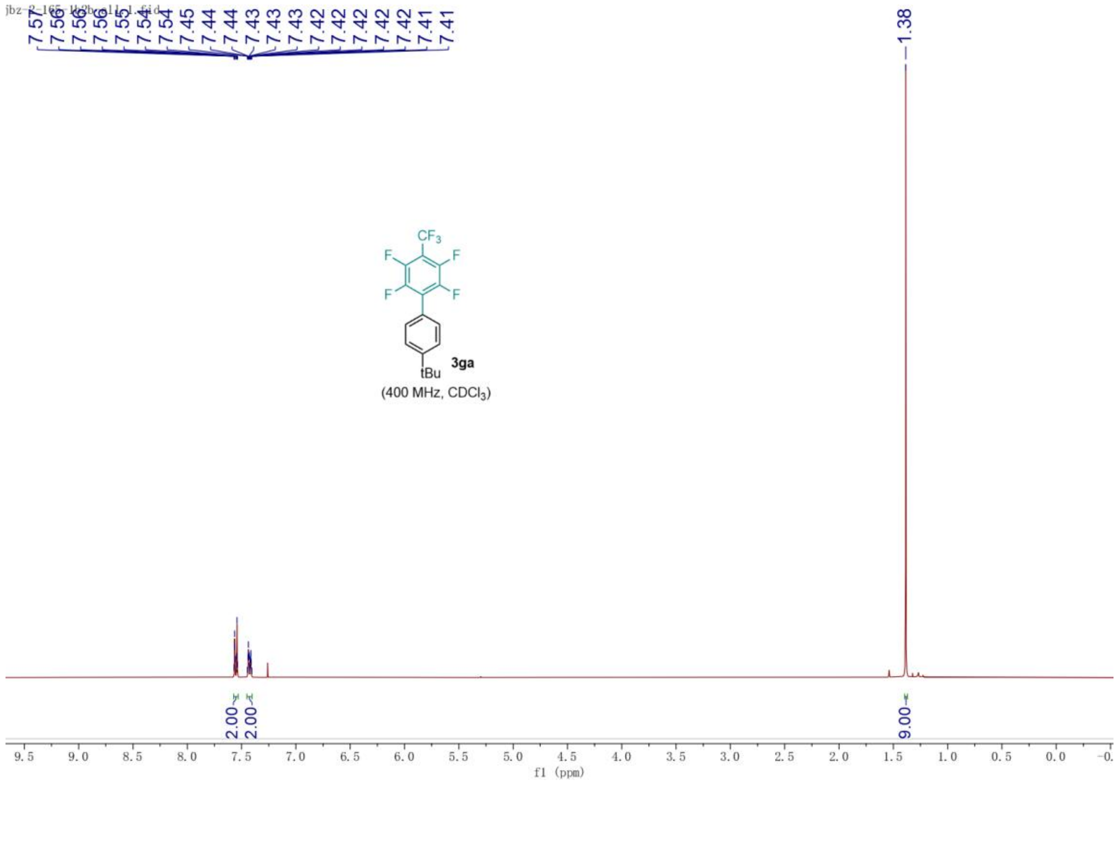
7.57
7.56
7.56
7.56
7.55
7.54
7.54
7.45
7.44
7.44
7.43
7.43
7.43
7.42
7.42
7.42
7.42
7.42
7.41
7.41
1.38
CF3
F
F
F
F
tBu
3ga
(400 MHz, CDCl3)
2.00
2.00
9.00
f1 (ppm)

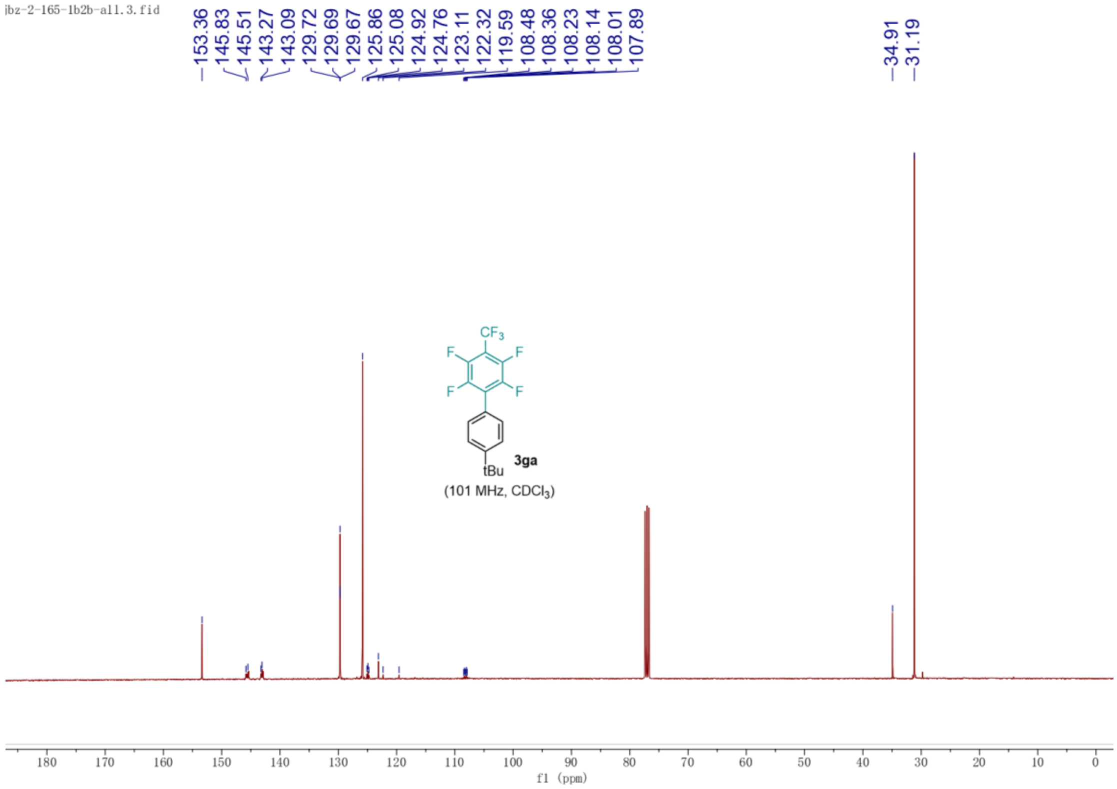
jbz-2-165-1b2b-all.3.fid
153.36
145.83
145.51
143.27
143.09
129.72
129.69
129.67
125.86
125.08
124.92
124.76
123.11
122.32
119.59
108.48
108.36
108.23
108.14
108.01
107.89
34.91
31.19
CF3
F
tBu
3ga
(101 MHz, CDCl3)
f1 (ppm)

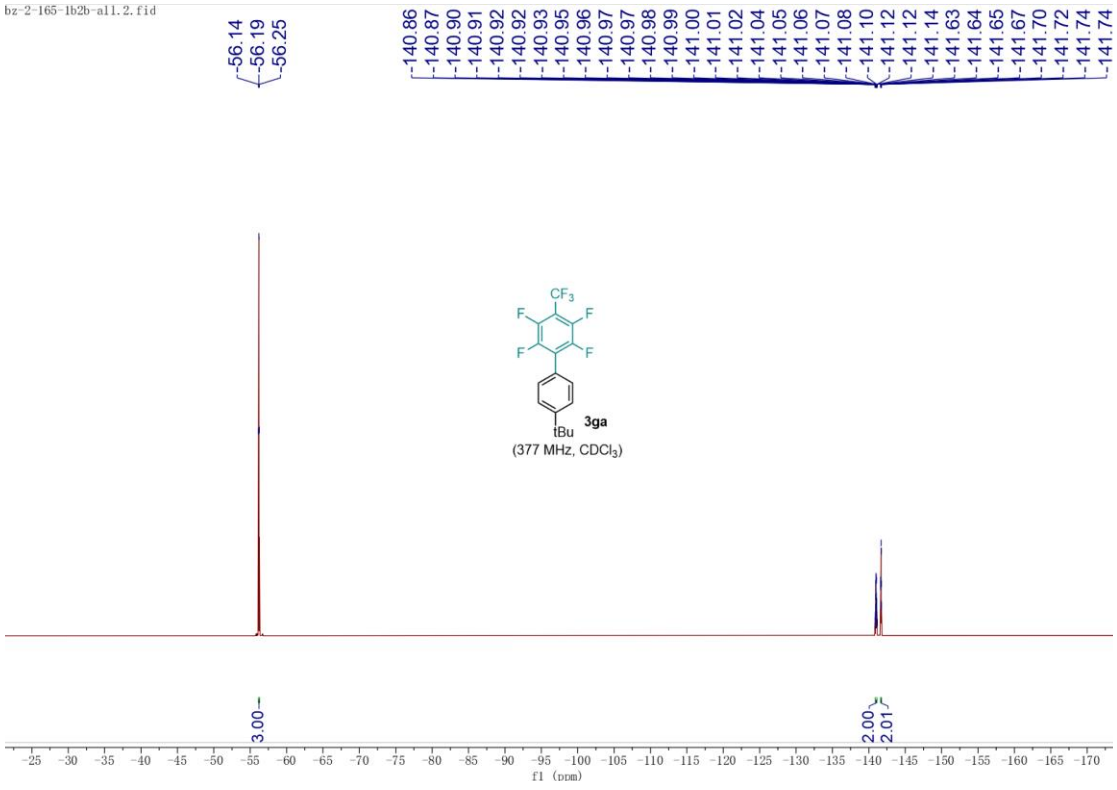
bz-2-165-1b2b-all.2.fid
-56.14
-56.19
-56.25
-140.86
-140.87
-140.90
-140.91
-140.92
-140.92
-140.93
-140.95
-140.96
-140.97
-140.97
-140.98
-140.99
-141.00
-141.01
-141.02
-141.04
-141.05
-141.06
-141.07
-141.08
-141.10
-141.12
-141.12
-141.14
-141.63
-141.64
-141.65
-141.67
-141.70
-141.72
-141.74
-141.74
CF3
F
tBu
3ga
(377 MHz, CDCl3)
3.00
2.00
2.01
f1 (ppm)

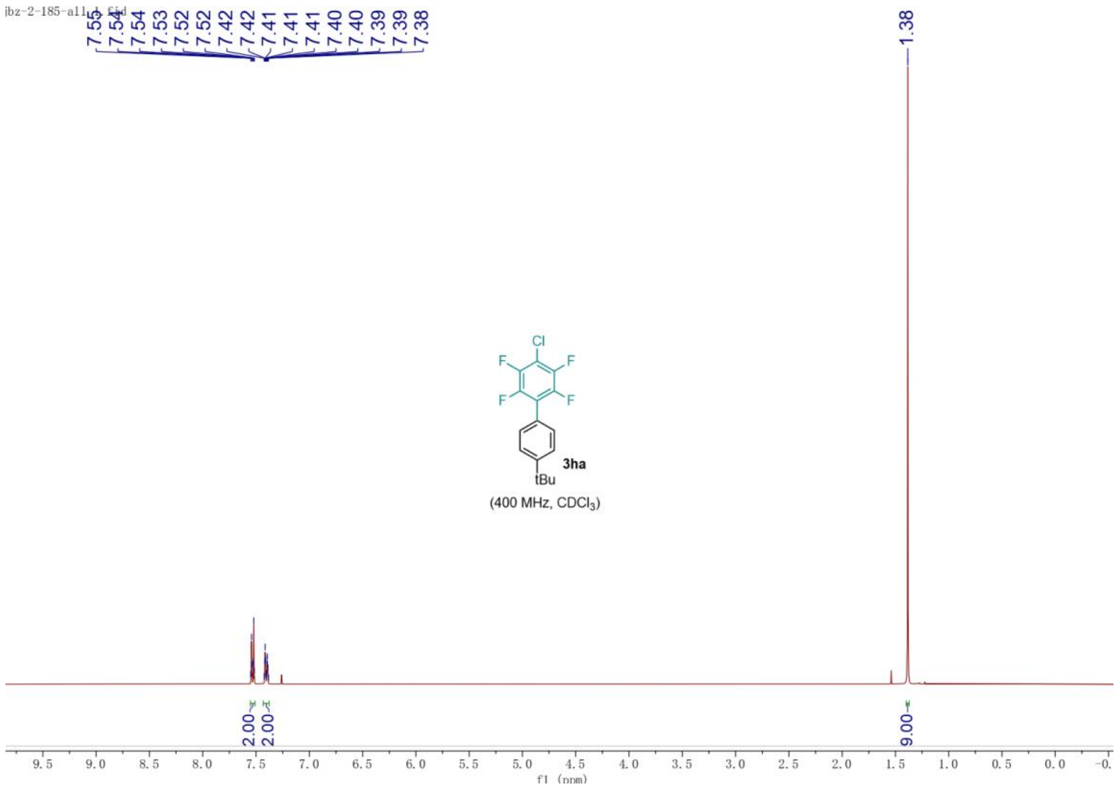
jbz-2-185-all.1.fid
7.55 7.54 7.54 7.53 7.52 7.52 7.42 7.41 7.41 7.40 7.40 7.39 7.39 7.38
1.38
Cl
F
F
F
F
3ha
tBu
(400 MHz, CDCl3)
2.00
2.00
9.00
f1 (ppm)

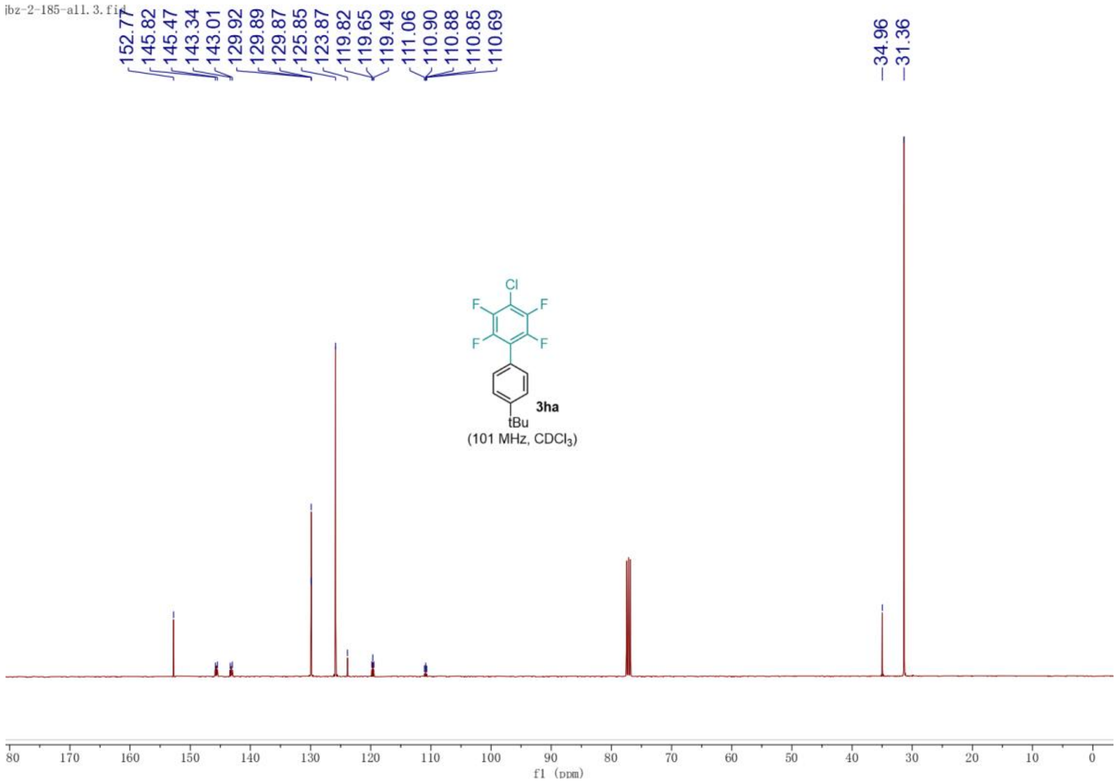
jbz-2-185-all.3.fid
152.77 145.82 145.47 143.34 143.01 129.92 129.89 129.87 125.85 123.87 119.82 119.65 119.49 111.06 110.90 110.88 110.85 110.69
34.96
31.36
Cl
F
F
F
F
3ha
tBu
(101 MHz, CDCl3)
f1 (ppm)

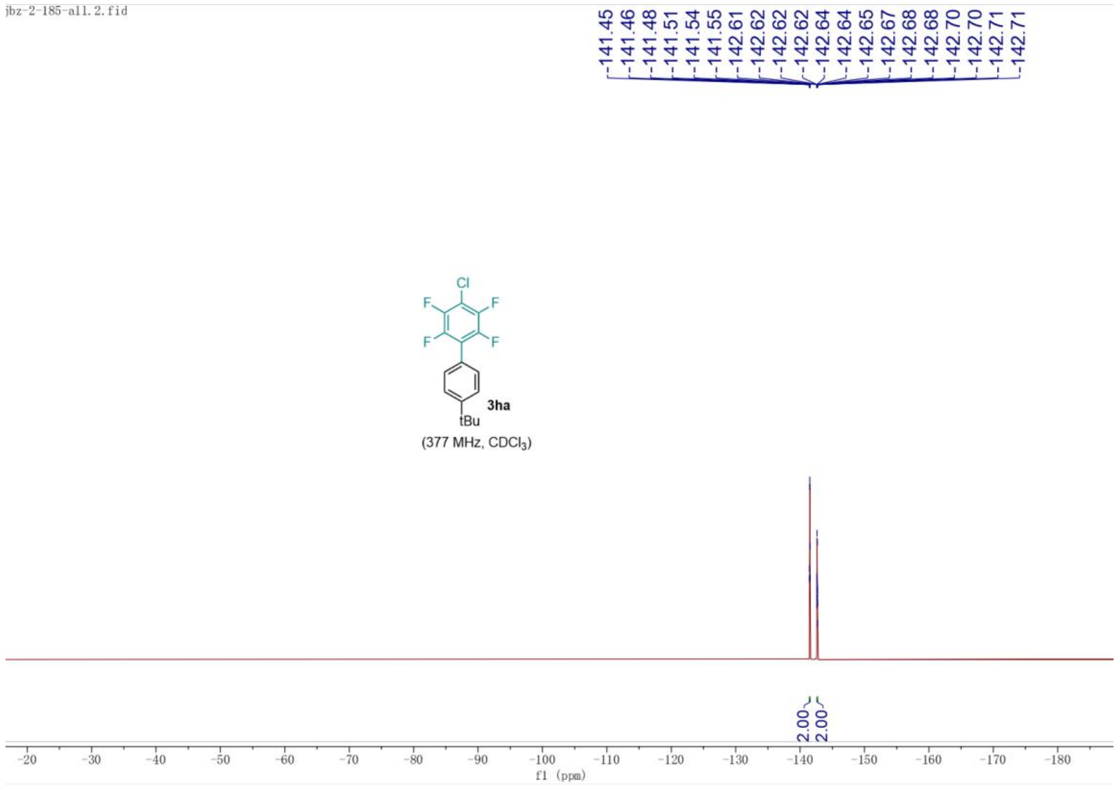
jbz-2-185-all.2.fid
-141.45
-141.46
-141.48
-141.51
-141.54
-141.55
-142.61
-142.62
-142.62
-142.62
-142.64
-142.64
-142.65
-142.67
-142.68
-142.68
-142.70
-142.70
-142.71
-142.71
Cl
F
F
F
F
tBu
3ha
(377 MHz, CDCl3)
2.00
2.00
-20 -30 -40 -50 -60 -70 -80 -90 -100 -110 -120 -130 -140 -150 -160 -170 -180
f1 (ppm)

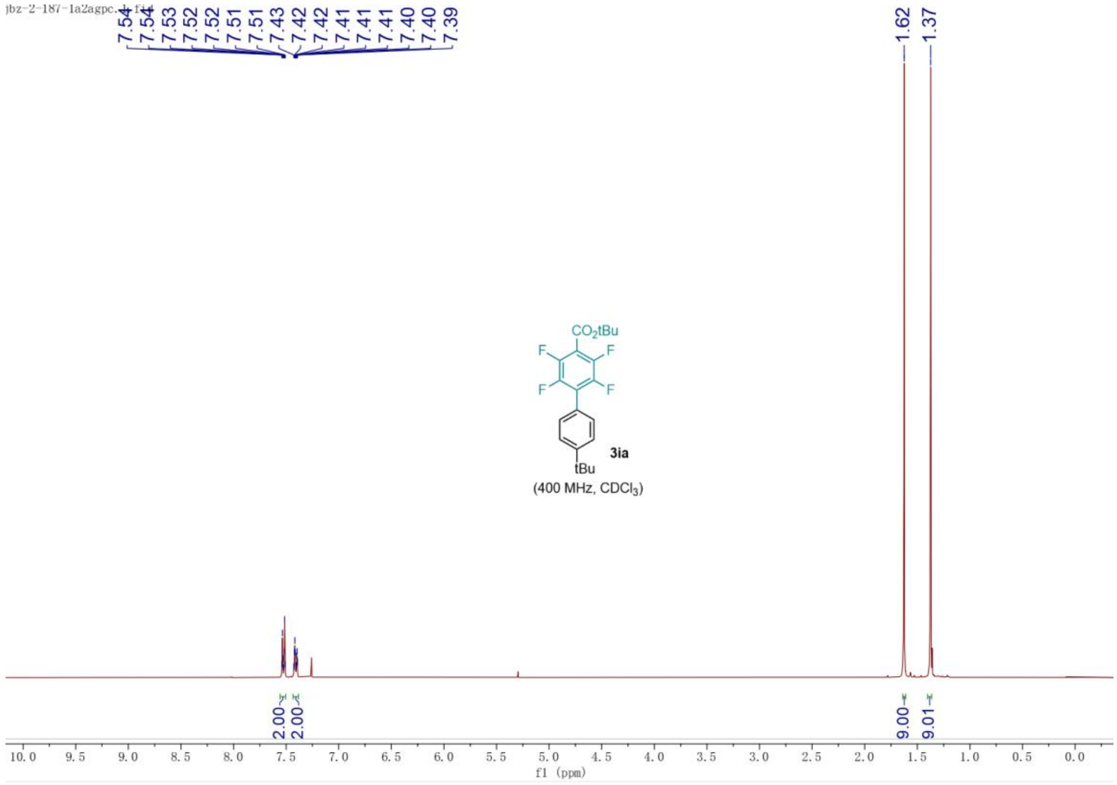
jbz-2-187-1a2agpc.1.fid
7.54
7.54
7.53
7.52
7.52
7.51
7.51
7.43
7.42
7.42
7.41
7.41
7.40
7.40
7.39
1.62
1.37
CO2tBu
F
F
F
F
tBu
3ia
(400 MHz, CDCl3)
2.00
2.00
9.00
9.01
10.0 9.5 9.0 8.5 8.0 7.5 7.0 6.5 6.0 5.5 5.0 4.5 4.0 3.5 3.0 2.5 2.0 1.5 1.0 0.5 0.0
f1 (ppm)

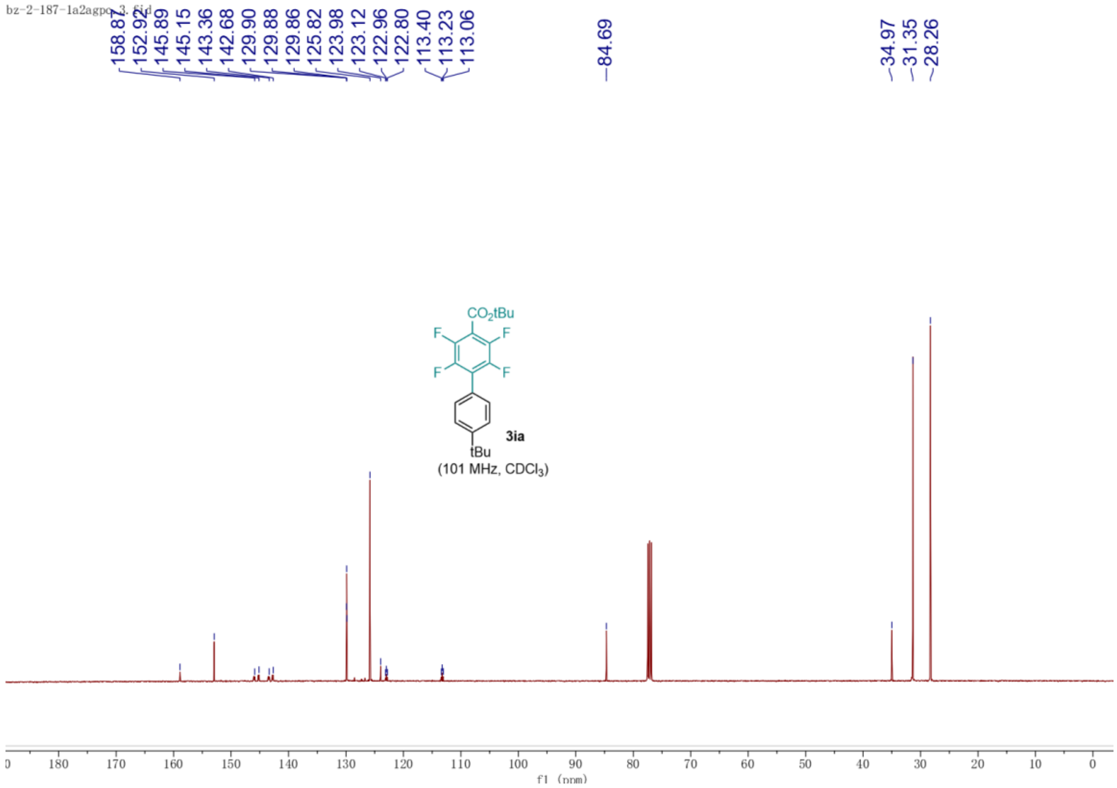

bz-2-187-1a2agpc.3.fid
158.87
152.92
145.89
145.15
143.36
142.68
129.90
129.88
129.86
125.82
123.98
123.12
122.96
122.80
113.40
113.23
113.06
84.69
34.97
31.35
28.26
CO2tBu
F
F
F
F
3ia
tBu
(101 MHz, CDCl3)
f1 (ppm)


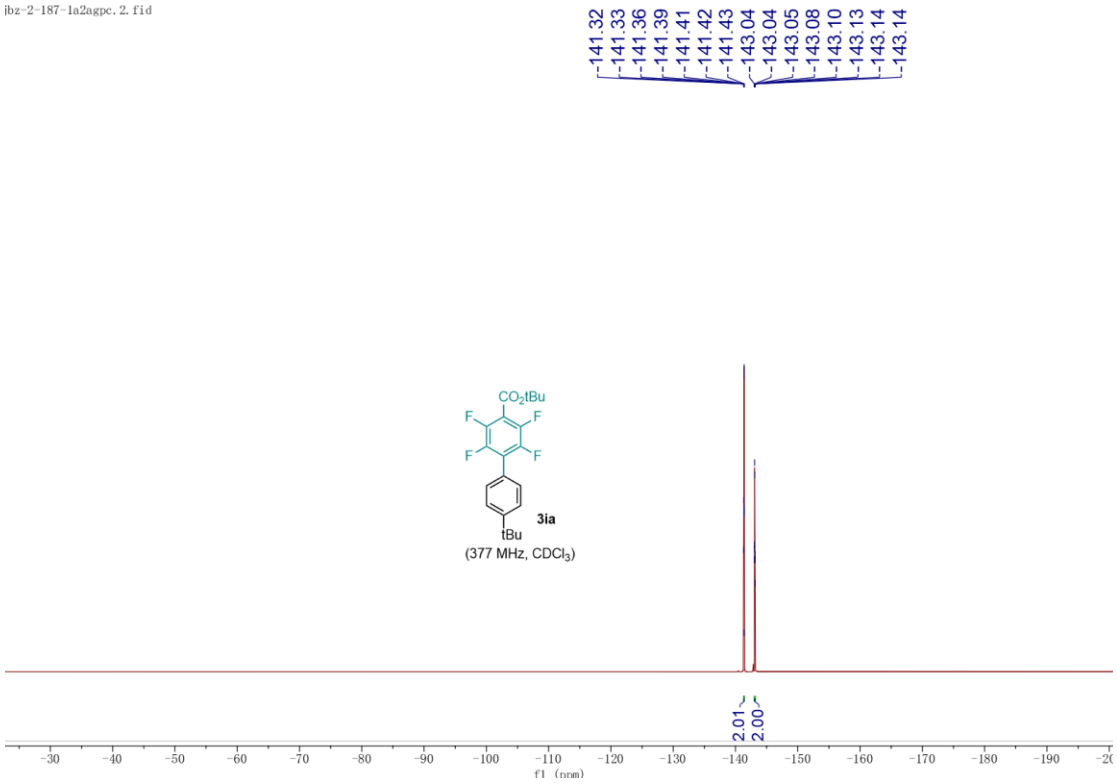

jbz-2-187-1a2agpc.2.fid
-141.32
-141.33
-141.36
-141.39
-141.41
-141.42
-141.43
-143.04
-143.04
-143.05
-143.08
-143.10
-143.13
-143.14
-143.14
CO2tBu
F
F
F
F
3ia
tBu
(377 MHz, CDCl3)
2.01
2.00
f1 (ppm)

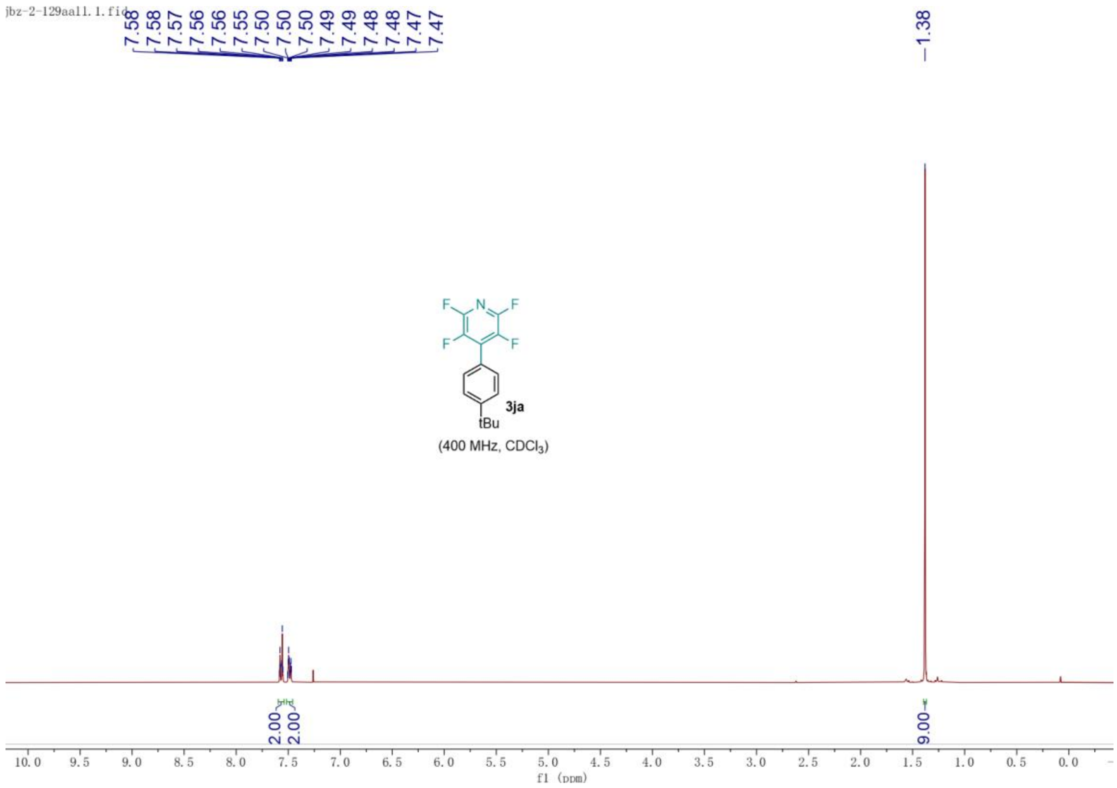
jbz-2-129aall.1.fid
7.58 7.58 7.57 7.56 7.56 7.55 7.50 7.50 7.50 7.49 7.49 7.48 7.48 7.47 7.47
1.38
F N F
F F
3ja
tBu
(400 MHz, CDCl3)
2.00
2.00
9.00
10.0 9.5 9.0 8.5 8.0 7.5 7.0 6.5 6.0 5.5 5.0 4.5 4.0 3.5 3.0 2.5 2.0 1.5 1.0 0.5 0.0
f1 (ppm)

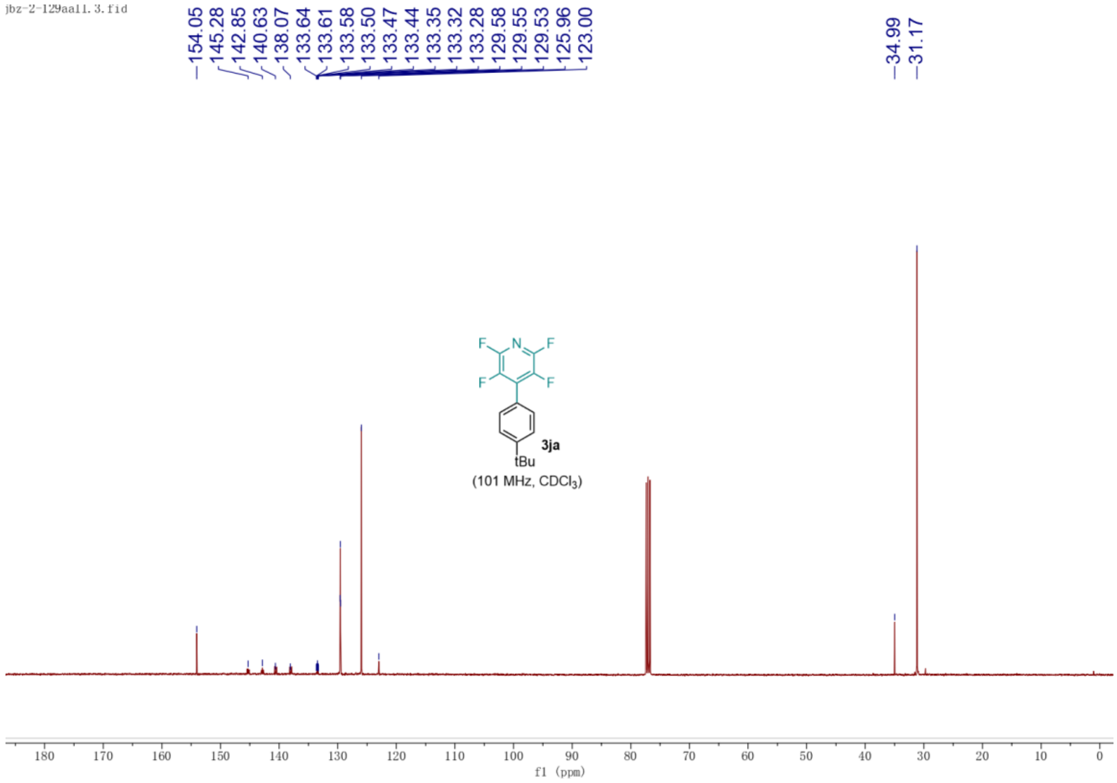
jbz-2-129aall.3.fid
154.05 145.28 142.85 140.63 138.07 133.64 133.61 133.58 133.50 133.47 133.44 133.35 133.32 133.28 129.58 129.55 129.53 125.96 123.00
34.99 31.17
F N F
F F
3ja
tBu
(101 MHz, CDCl3)
180 170 160 150 140 130 120 110 100 90 80 70 60 50 40 30 20 10 0
f1 (ppm)

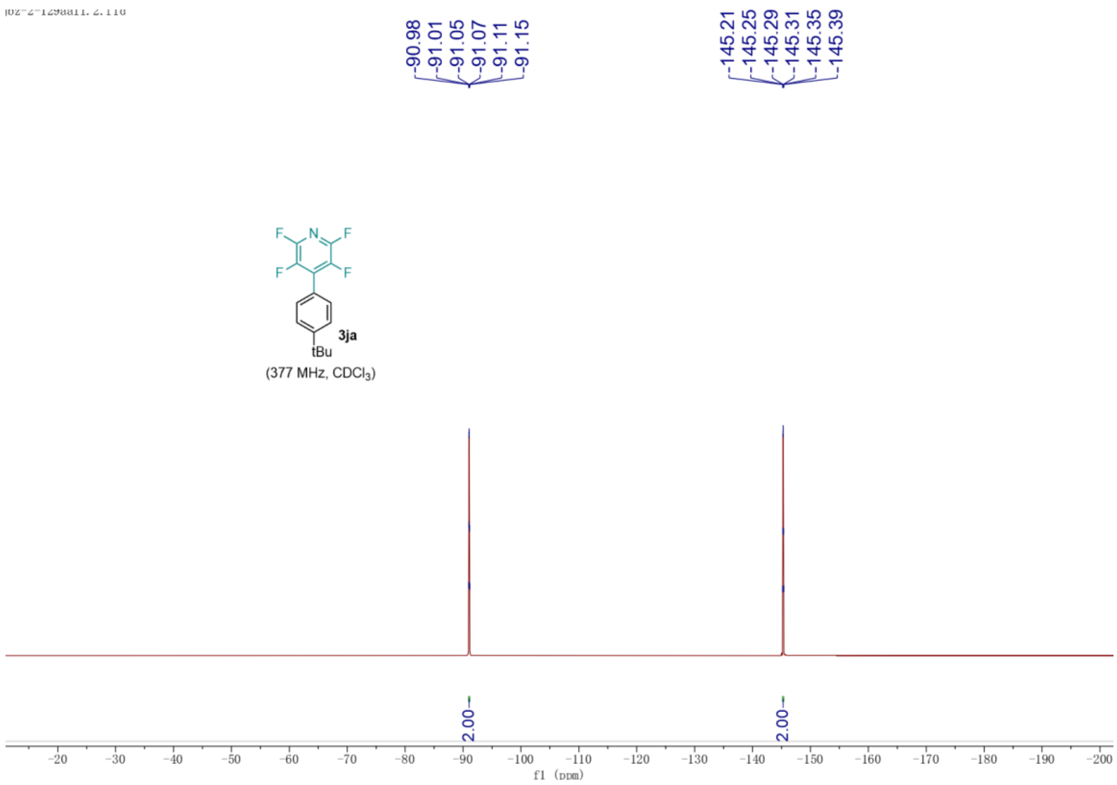
-90.98
-91.01
-91.05
-91.07
-91.11
-91.15
-145.21
-145.25
-145.29
-145.31
-145.35
-145.39
3ja
tBu
(377 MHz, CDCl3)
2.00
2.00
f1 (ppm)

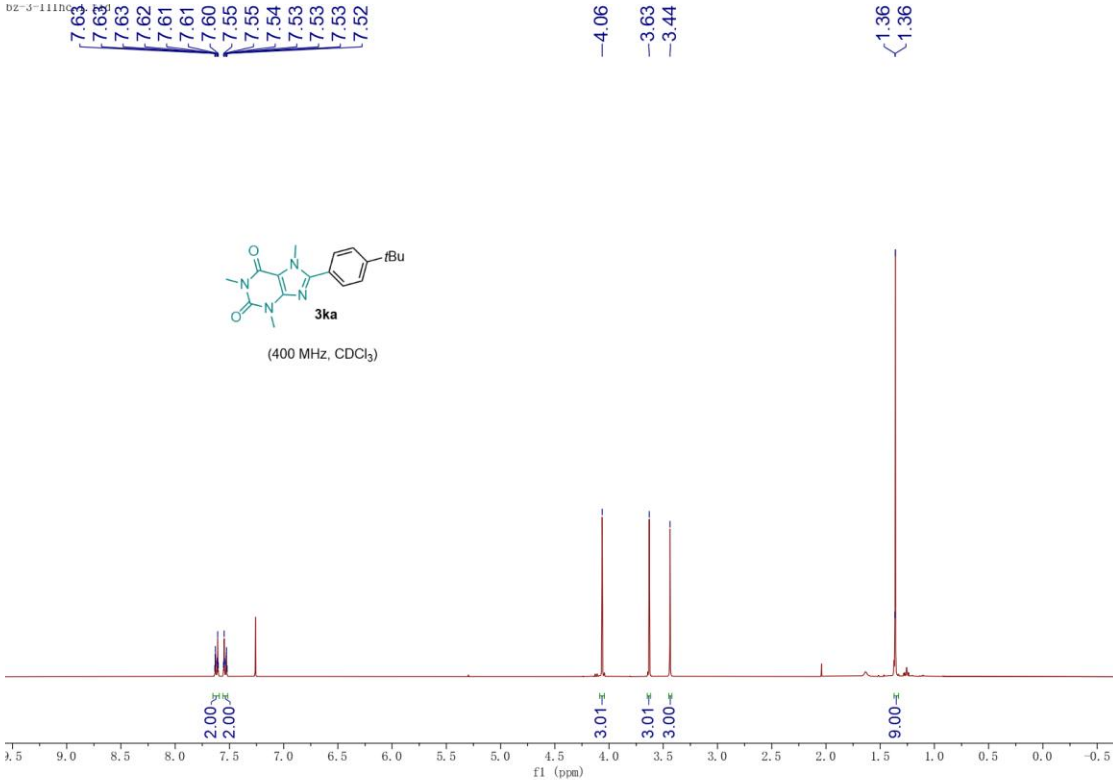
7.63
7.63
7.63
7.62
7.61
7.61
7.60
7.55
7.55
7.54
7.53
7.53
7.53
7.52
4.06
3.63
3.44
1.36
1.36
tBu
3ka
(400 MHz, CDCl3)
2.00
2.00
3.01
3.01
3.00
9.00
f1 (ppm)

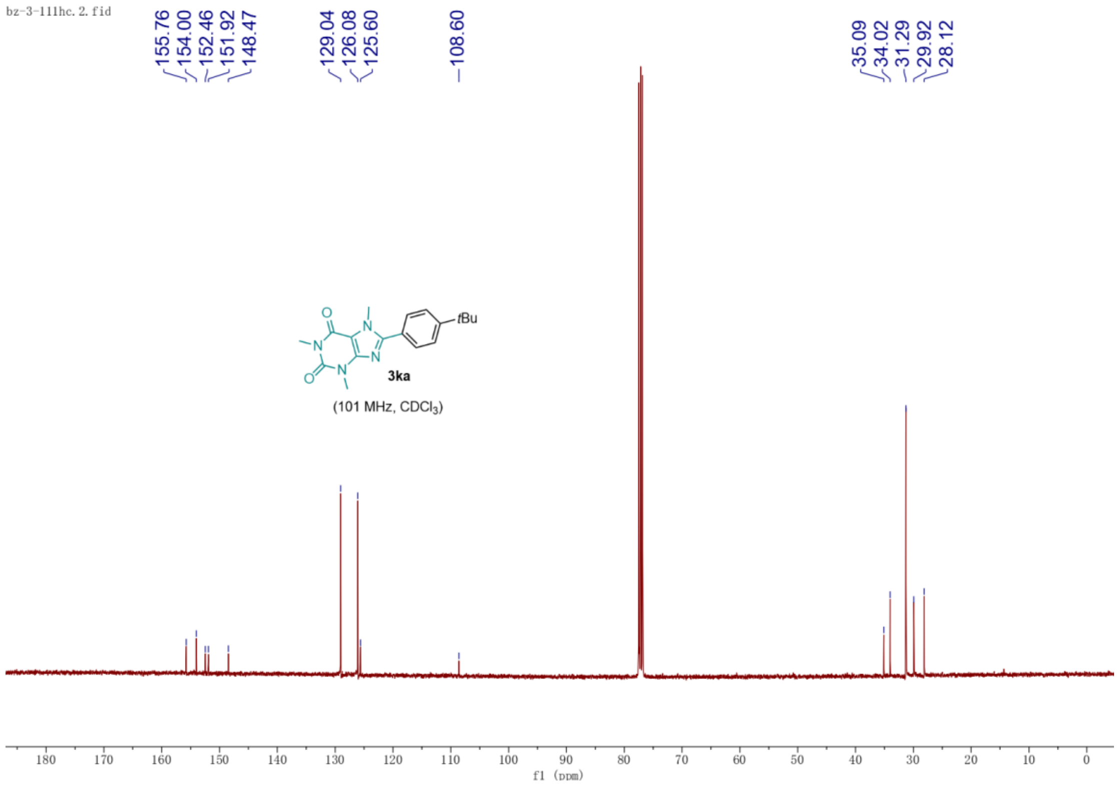
bz-3-111hc.2.fid
155.76
154.00
152.46
151.92
148.47
129.04
126.08
125.60
108.60
35.09
34.02
31.29
29.92
28.12
tBu
3ka
(101 MHz, CDCl3)
f1 (ppm)

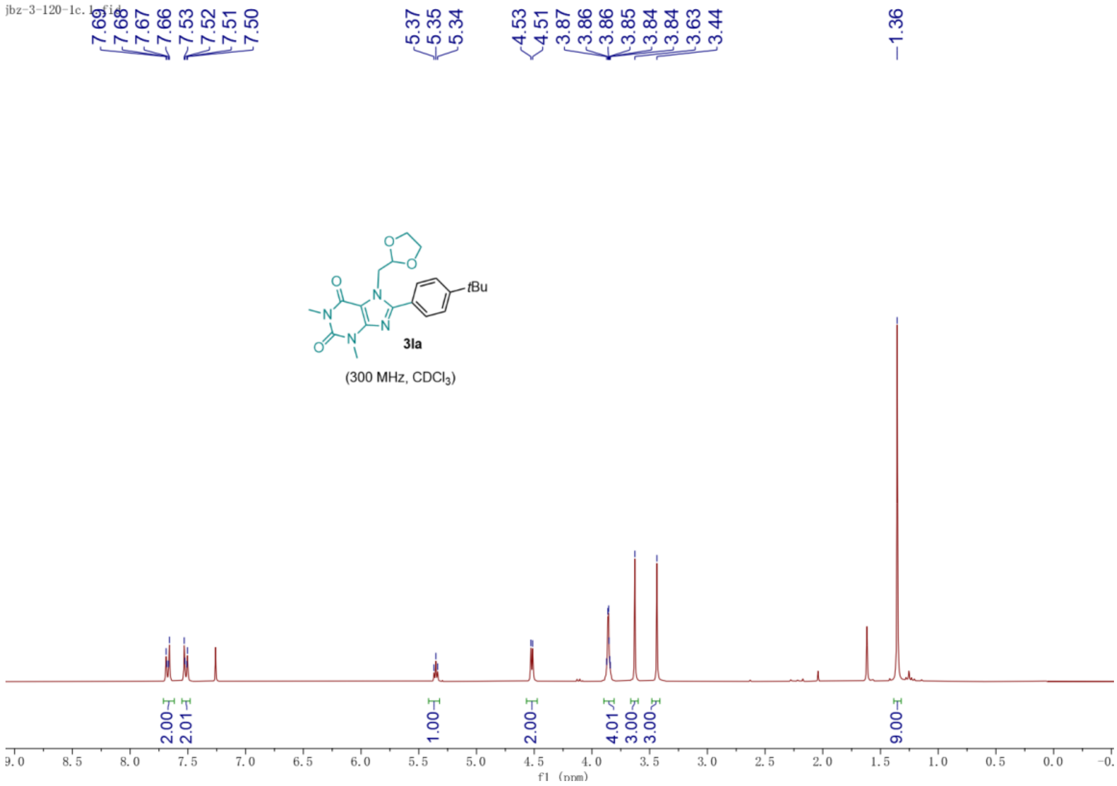
jbz-3-120-1c.1.fid
7.69
7.68
7.67
7.66
7.53
7.52
7.51
7.50
5.37
5.35
5.34
4.53
4.51
3.87
3.86
3.86
3.85
3.84
3.84
3.63
3.44
1.36
tBu
3la
(300 MHz, CDCl3)
2.00
2.01
1.00
2.00
4.01
3.00
3.00
9.00
f1 (ppm)

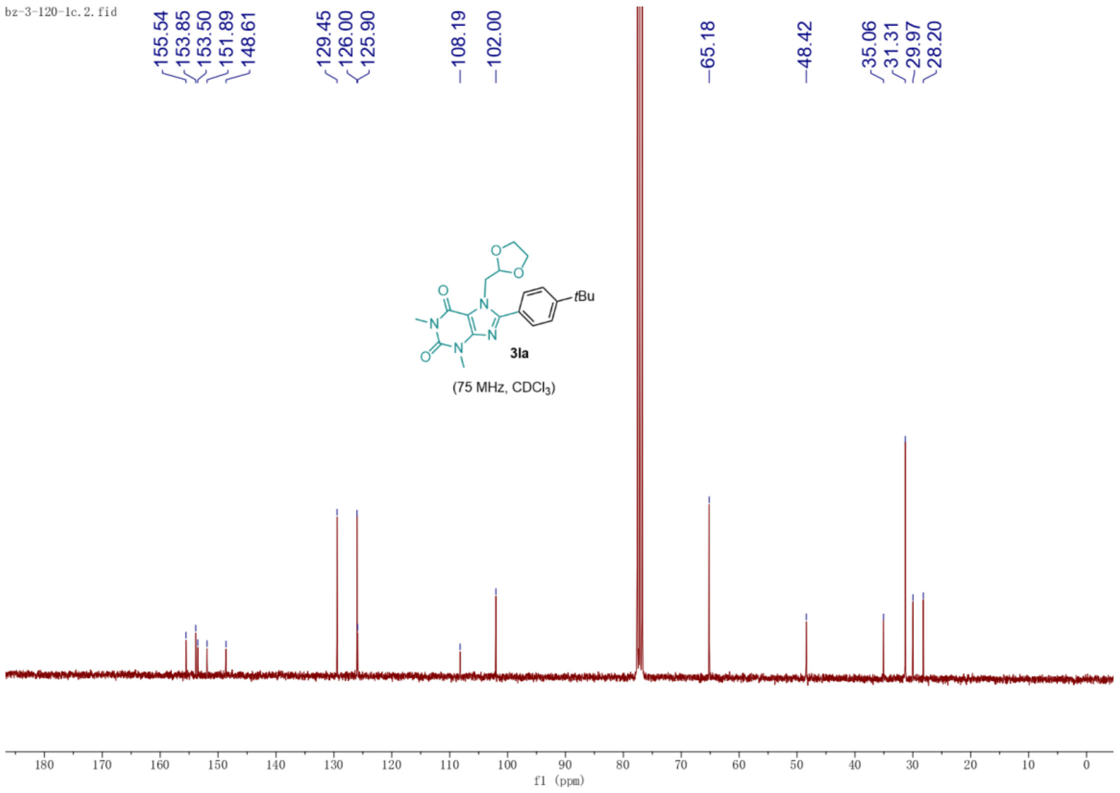
bz-3-120-1c.2.fid
155.54
153.85
153.50
151.89
148.61
129.45
126.00
125.90
108.19
102.00
65.18
48.42
35.06
31.31
29.97
28.20
tBu
3la
(75 MHz, CDCl3)
f1 (ppm)

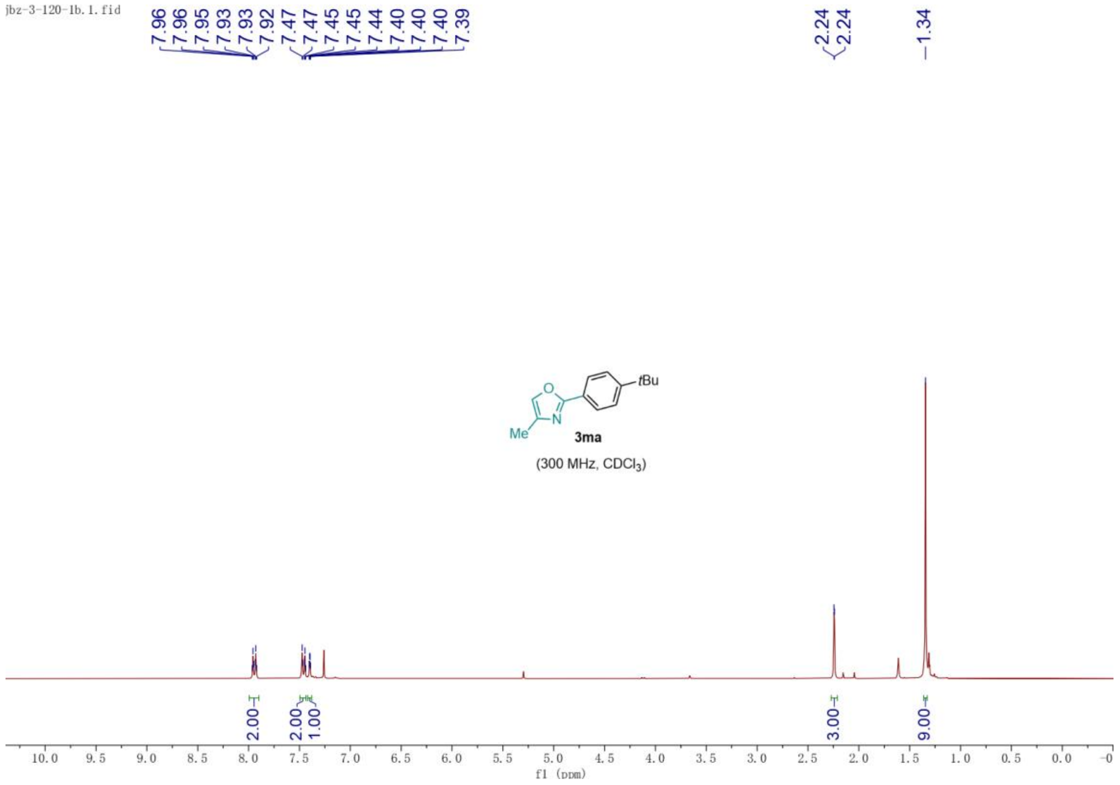
jbz-3-120-1b.1.fid
7.96
7.96
7.95
7.93
7.93
7.92
7.47
7.47
7.45
7.45
7.44
7.40
7.40
7.40
7.39
2.24
2.24
1.34
tBu
Me
3ma
(300 MHz, CDCl3)
2.00
2.00
1.00
3.00
9.00
f1 (ppm)

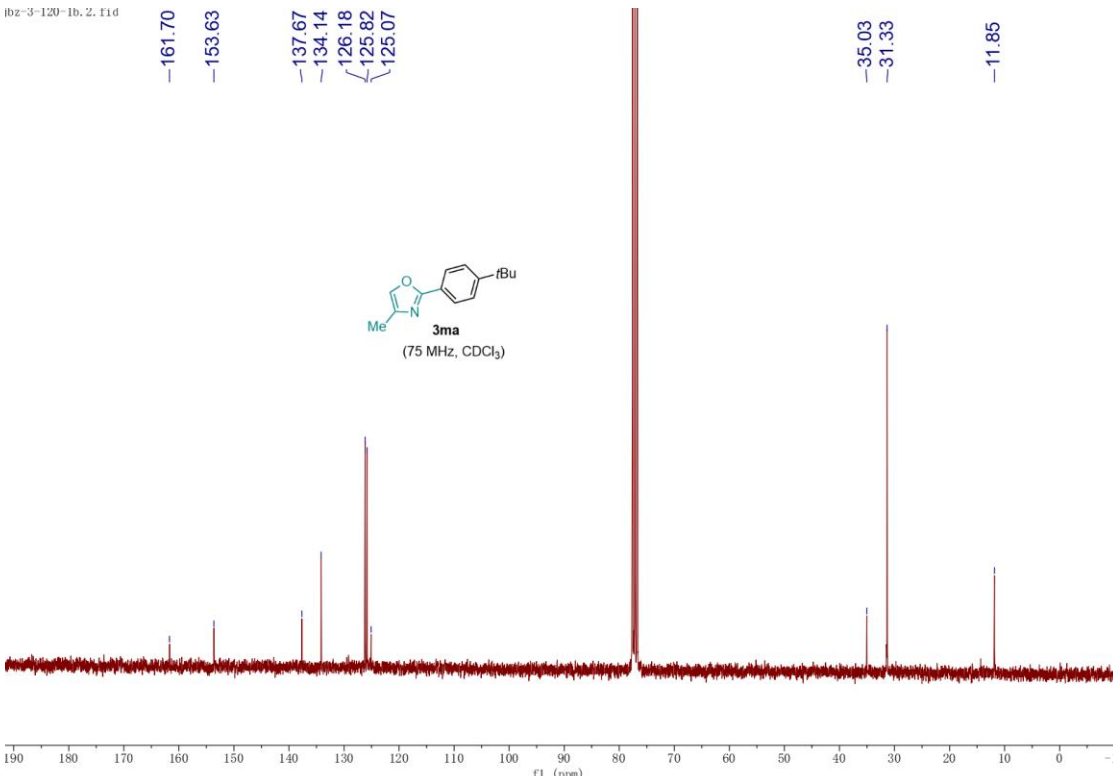
jbz-3-120-1b.2.fid
161.70
153.63
137.67
134.14
126.18
125.82
125.07
35.03
31.33
11.85
tBu
Me
O
N
3ma
(75 MHz, CDCl3)
f1 (ppm)

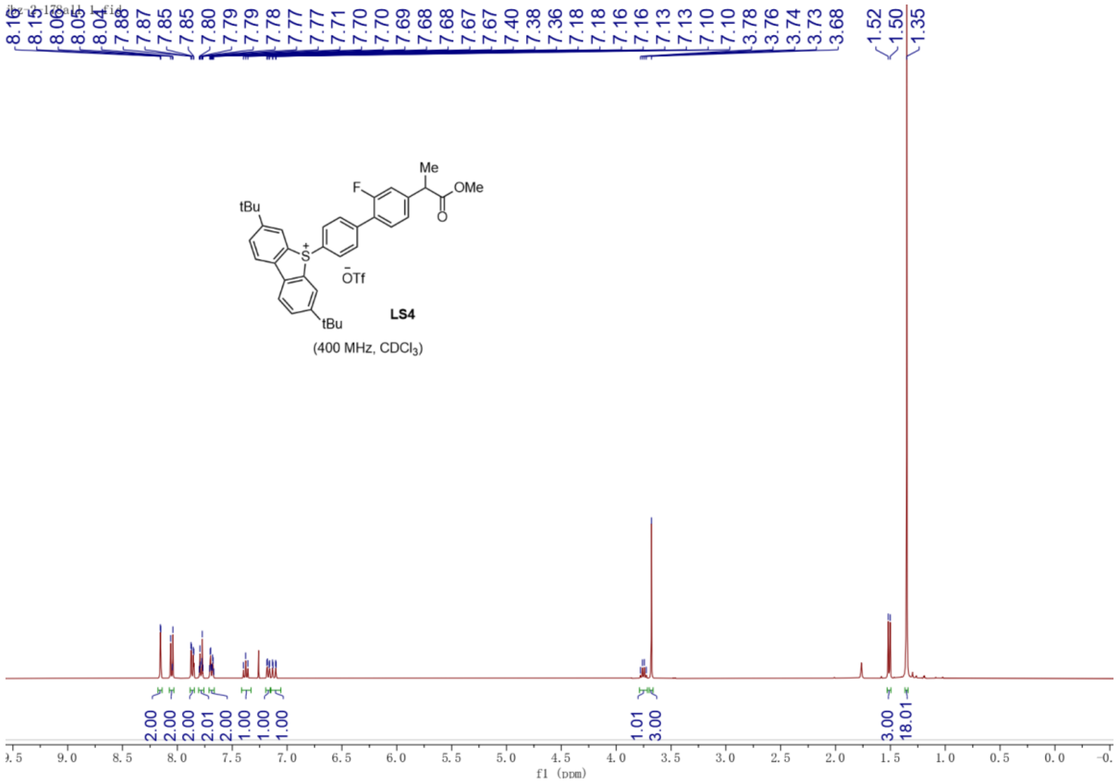
1.52
1.50
1.35
tBu
F
Me
OMe
O
S
OTf
tBu
LS4
(400 MHz, CDCl3)
f1 (ppm)

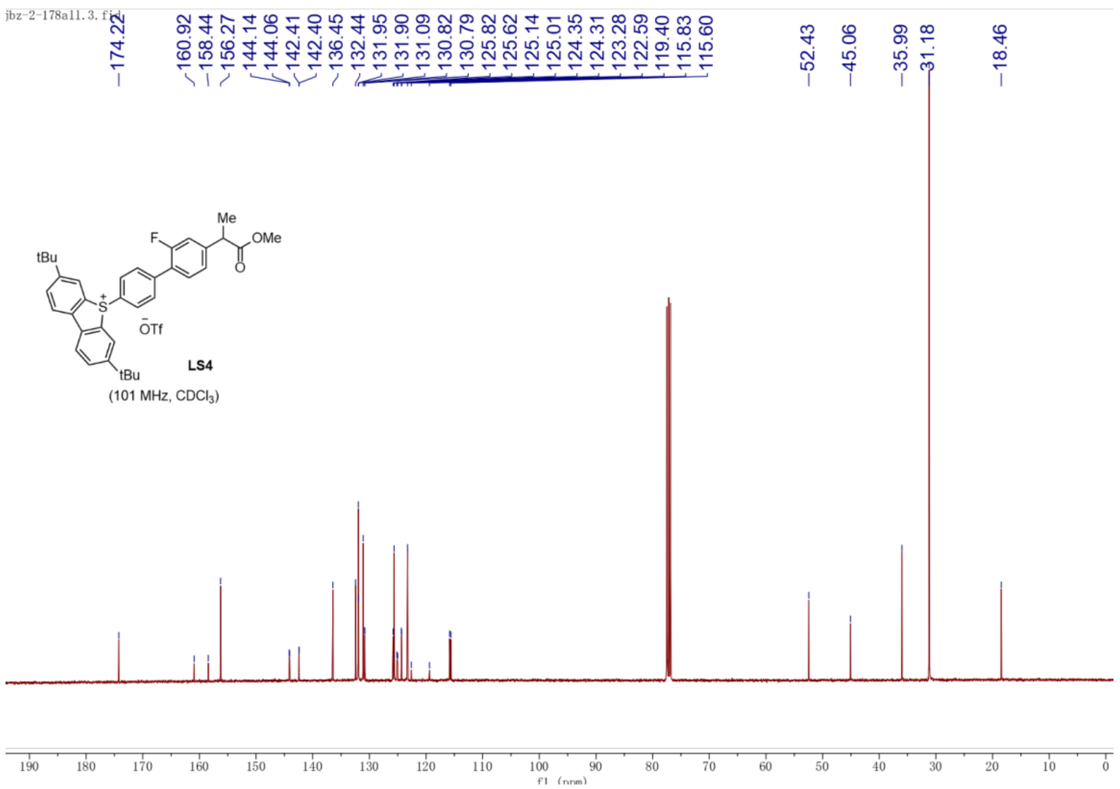
jbz-2-178all.3.fid
174.22
160.92
158.44
156.27
144.14
144.06
142.41
142.40
136.45
132.44
131.95
131.90
131.09
130.82
130.79
125.82
125.62
125.14
125.01
124.35
124.31
123.28
122.59
119.40
115.83
115.60
52.43
45.06
35.99
31.18
18.46
Me
F
OMe
O
tBu
S+
OTf
tBu
LS4
(101 MHz, CDCl3)
f1 (ppm)

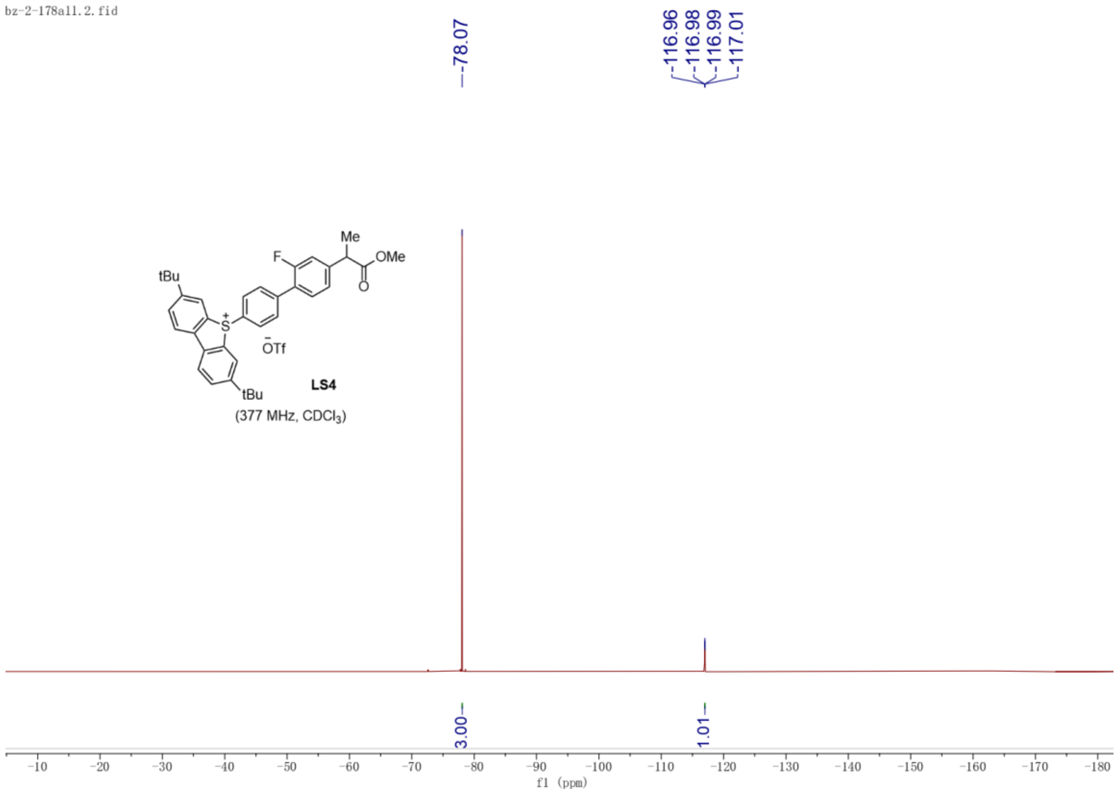
bz-2-178all.2.fid
-78.07
-116.96
-116.98
-116.99
-117.01
Me
F
OMe
O
tBu
S+
OTf
tBu
LS4
(377 MHz, CDCl3)
3.00
1.01
f1 (ppm)

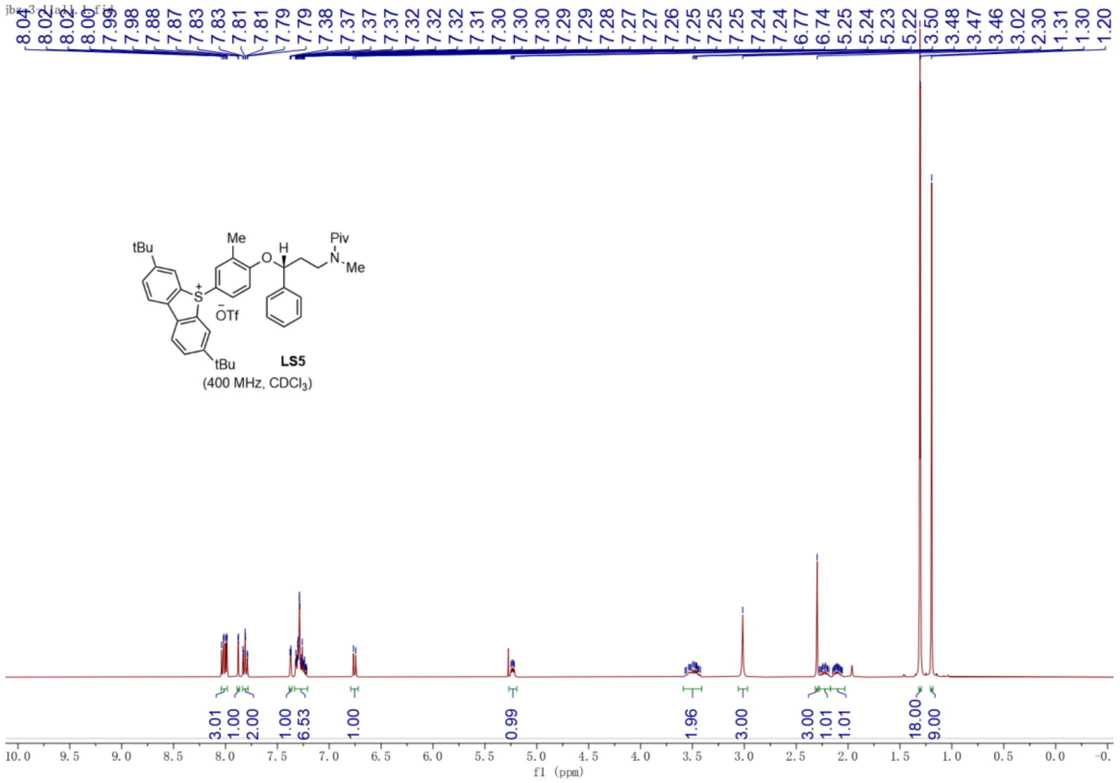
jbz-3-11all.1.fid
tBu
Me
H
O
Piv
N
Me
S+
OTf
tBu
LS5
(400 MHz, CDCl3)
f1 (ppm)

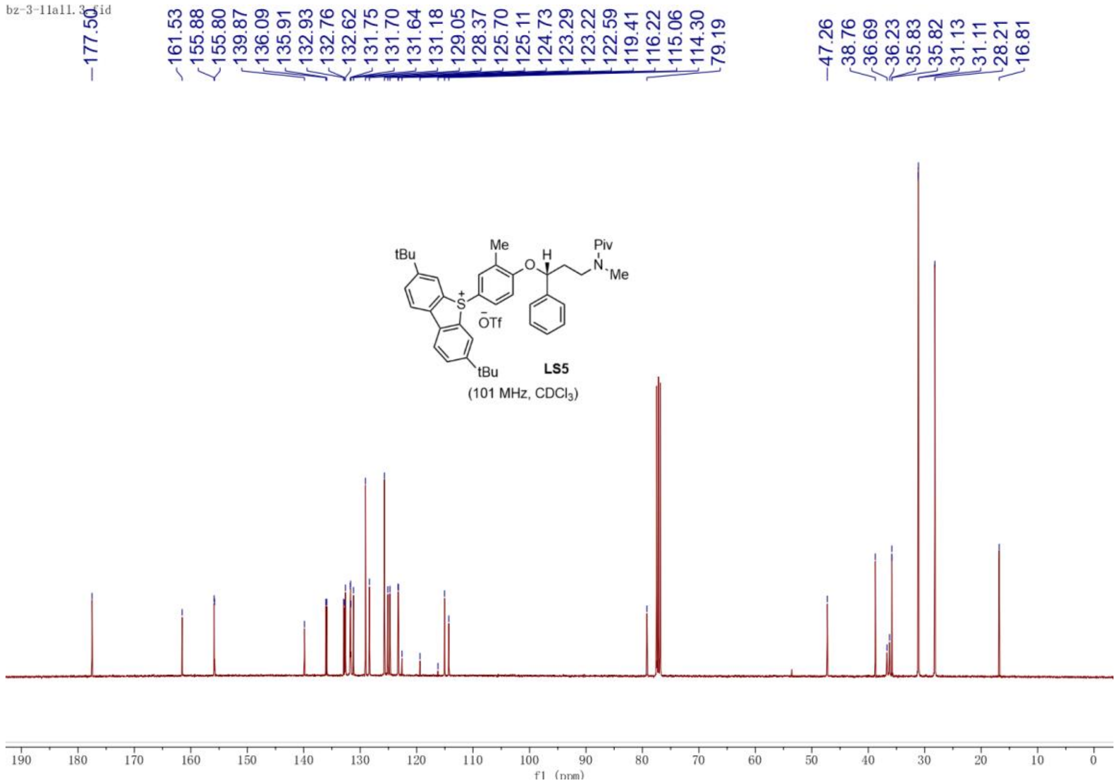
bz-3-11all.3.fid
tBu
Me
H
O
Piv
N
Me
S+
OTf
tBu
LS5
(101 MHz, CDCl3)
f1 (ppm)

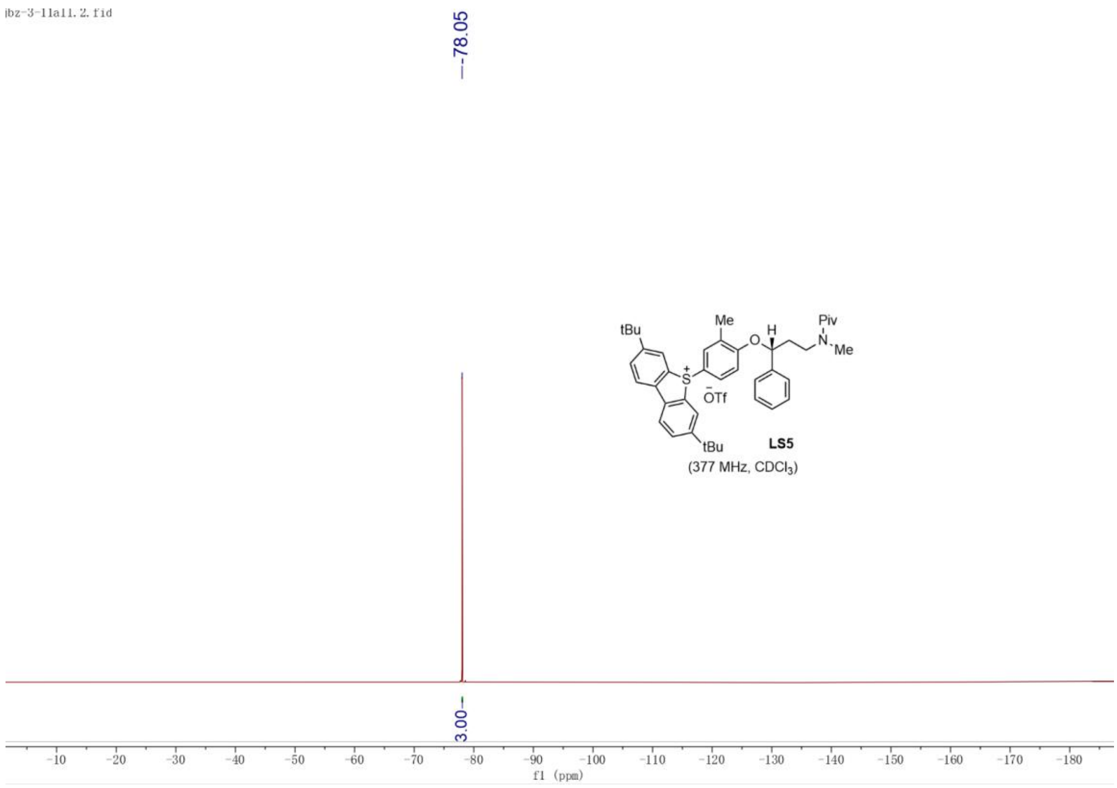
jbz-3-11all.2.fid
-78.05
tBu
Me
H
O
Piv
N
Me
S
OTf
tBu
LS5
(377 MHz, CDCl3)
3.00
f1 (ppm)

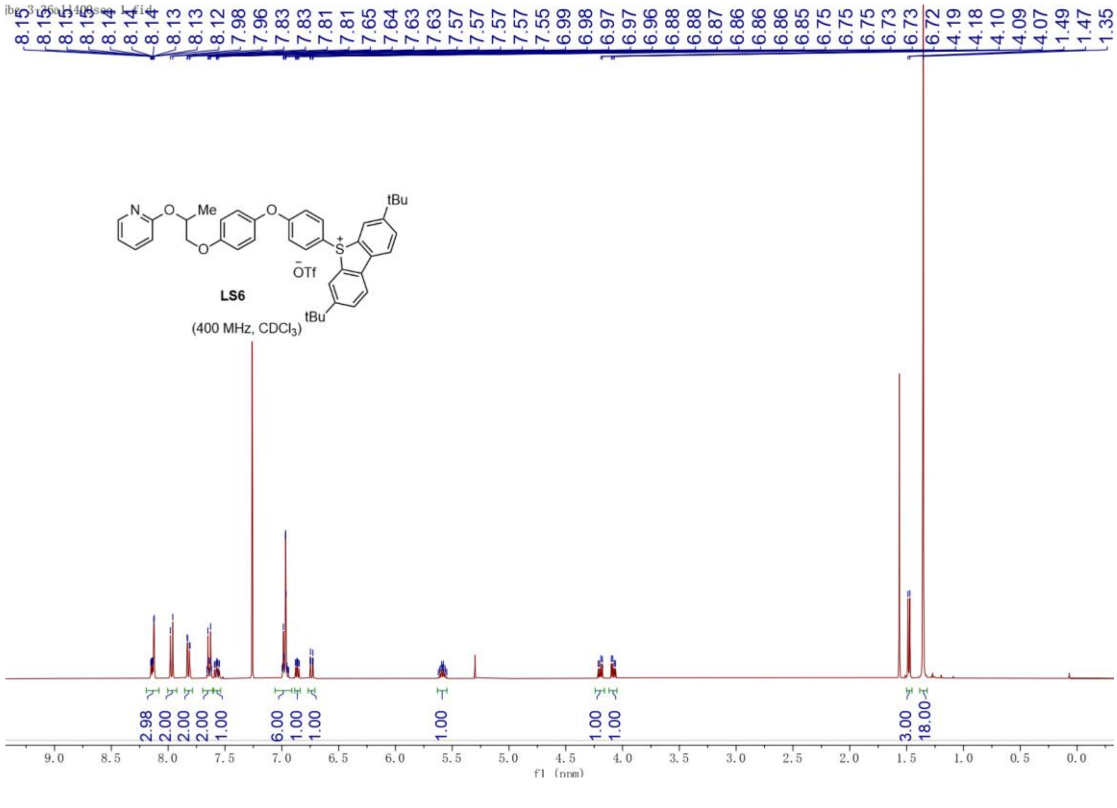
8.15 8.15 8.15 8.15 8.14 8.14 8.14 8.13 8.13 8.12 7.98 7.96 7.83 7.83 7.81 7.81 7.65 7.64 7.63 7.63 7.57 7.57 7.57 7.57 7.55 6.99 6.98 6.97 6.97 6.96 6.88 6.88 6.87 6.86 6.86 6.86 6.85 6.75 6.75 6.75 6.73 6.73 6.72 4.19 4.18 4.10 4.09 4.07 1.49 1.47 1.35
N
O
Me
O
O
tBu
S
OTf
LS6
tBu
(400 MHz, CDCl3)
2.98 2.00 2.00 2.00 1.00 6.00 1.00 1.00 1.00 1.00 1.00 3.00 18.00
f1 (ppm)

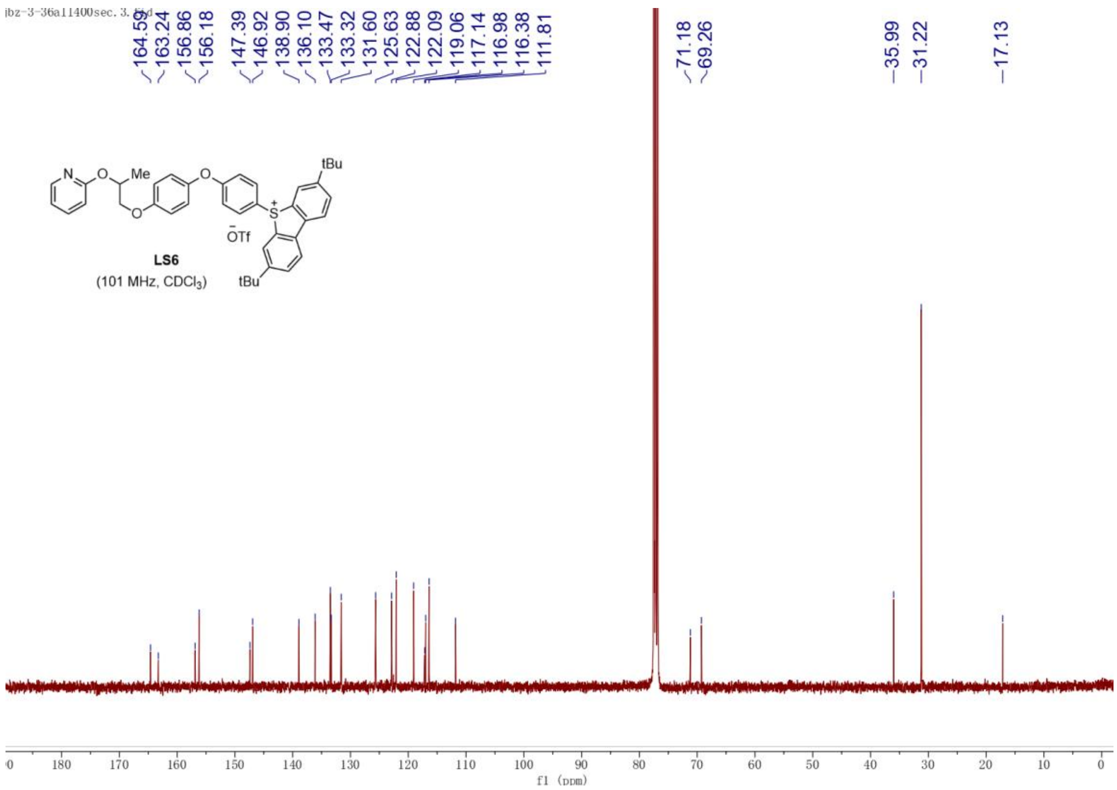
jbz-3-36a11400sec.3.fid
164.59
163.24
156.86
156.18
147.39
146.92
138.90
136.10
133.47
133.32
131.60
125.63
122.88
122.09
119.06
117.14
116.98
116.38
111.81
71.18
69.26
35.99
31.22
17.13
tBu
Me
OTf
LS6
(101 MHz, CDCl3)
tBu
f1 (ppm)

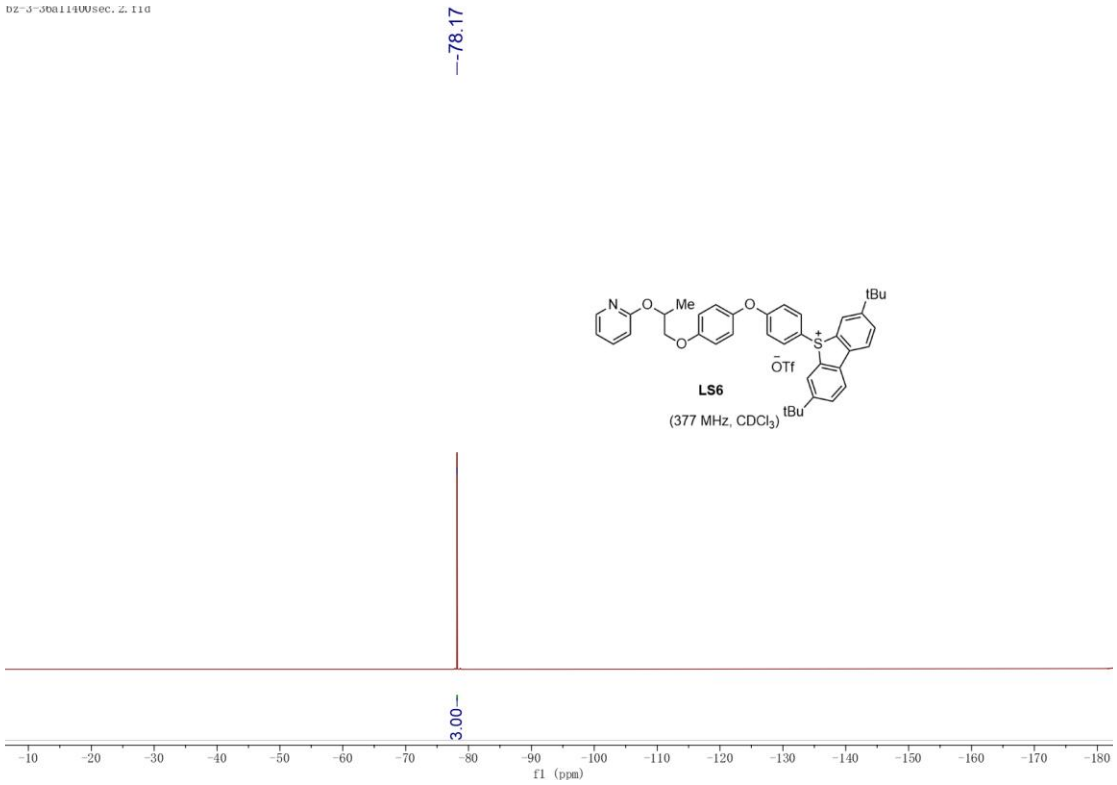
bz-3-36a11400sec.2.fid
-78.17
tBu
Me
OTf
LS6
(377 MHz, CDCl3)
tBu
3.00
f1 (ppm)

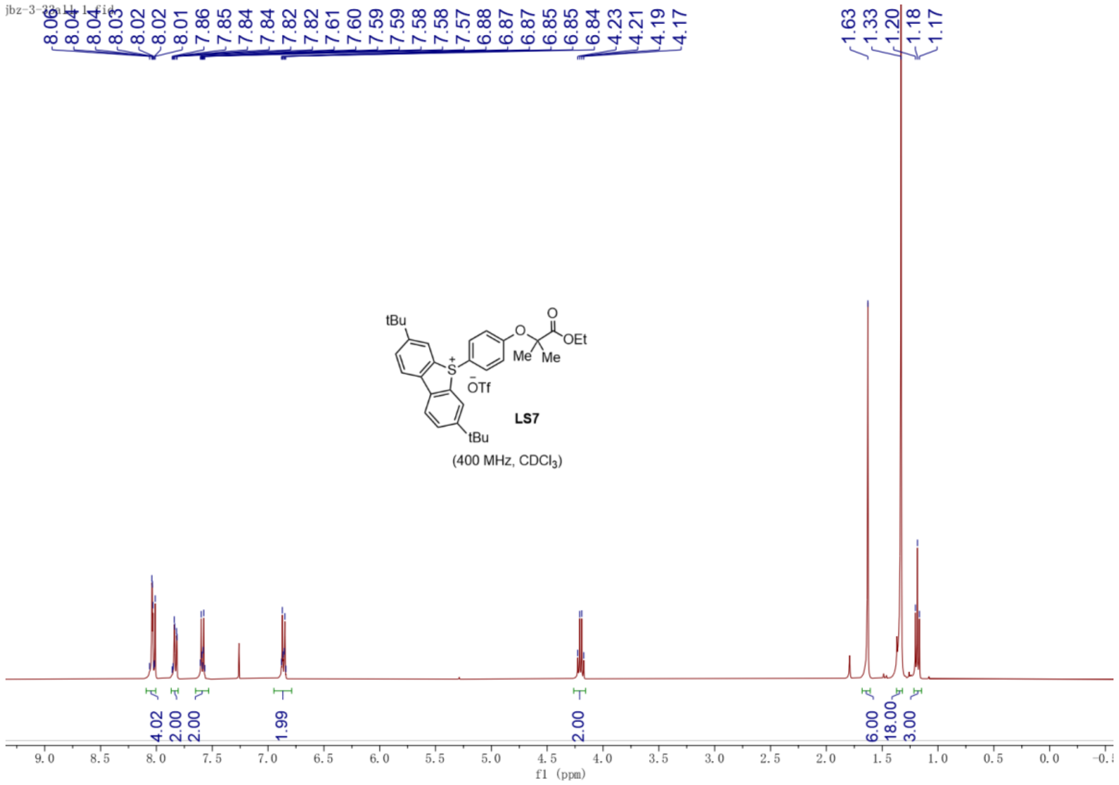
jbz-3-33all.1.fid
8.06
8.04
8.04
8.03
8.02
8.02
8.01
7.86
7.85
7.84
7.84
7.82
7.82
7.61
7.60
7.59
7.59
7.58
7.58
7.57
6.88
6.87
6.87
6.85
6.85
6.84
4.23
4.21
4.19
4.17
1.63
1.33
1.20
1.18
1.17
tBu
OTf
O
OEt
Me
Me
tBu
LS7
(400 MHz, CDCl3)
4.02
2.00
2.00
1.99
2.00
6.00
18.00
3.00
f1 (ppm)

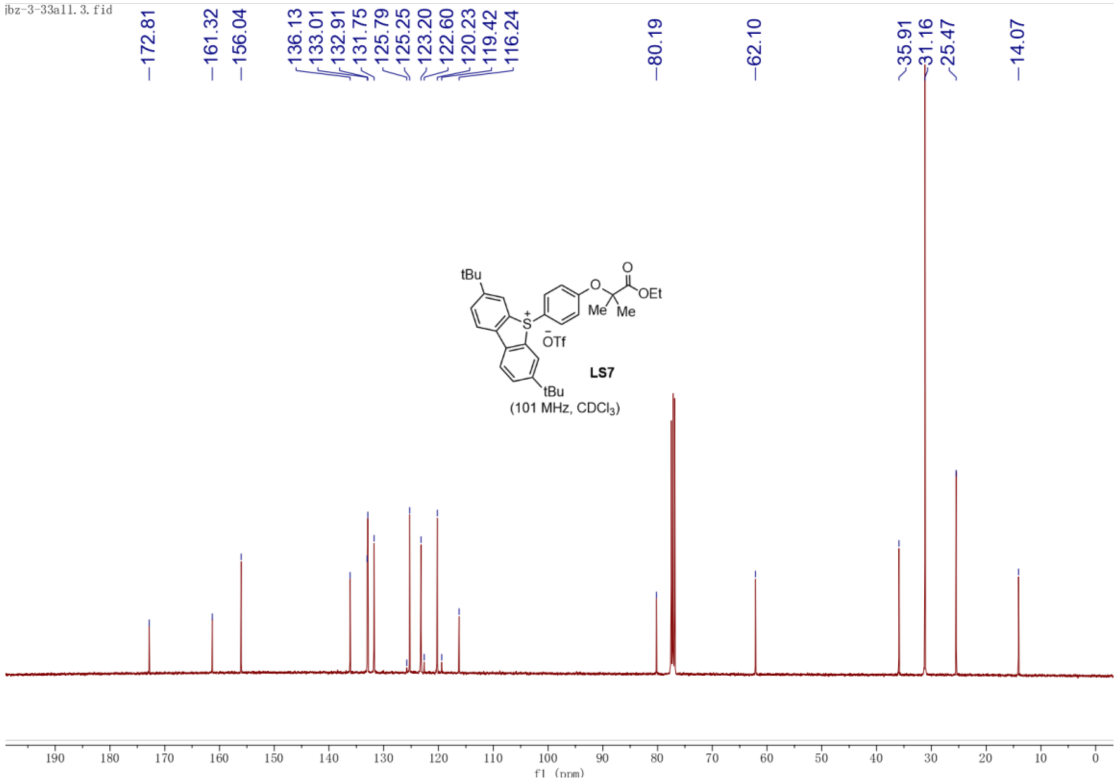
jbz-3-33all.3.fid
172.81
161.32
156.04
136.13
133.01
132.91
131.75
125.79
125.25
123.20
122.60
120.23
119.42
116.24
80.19
62.10
35.91
31.16
25.47
14.07
tBu
OTf
O
OEt
Me
Me
tBu
LS7
(101 MHz, CDCl3)
f1 (ppm)

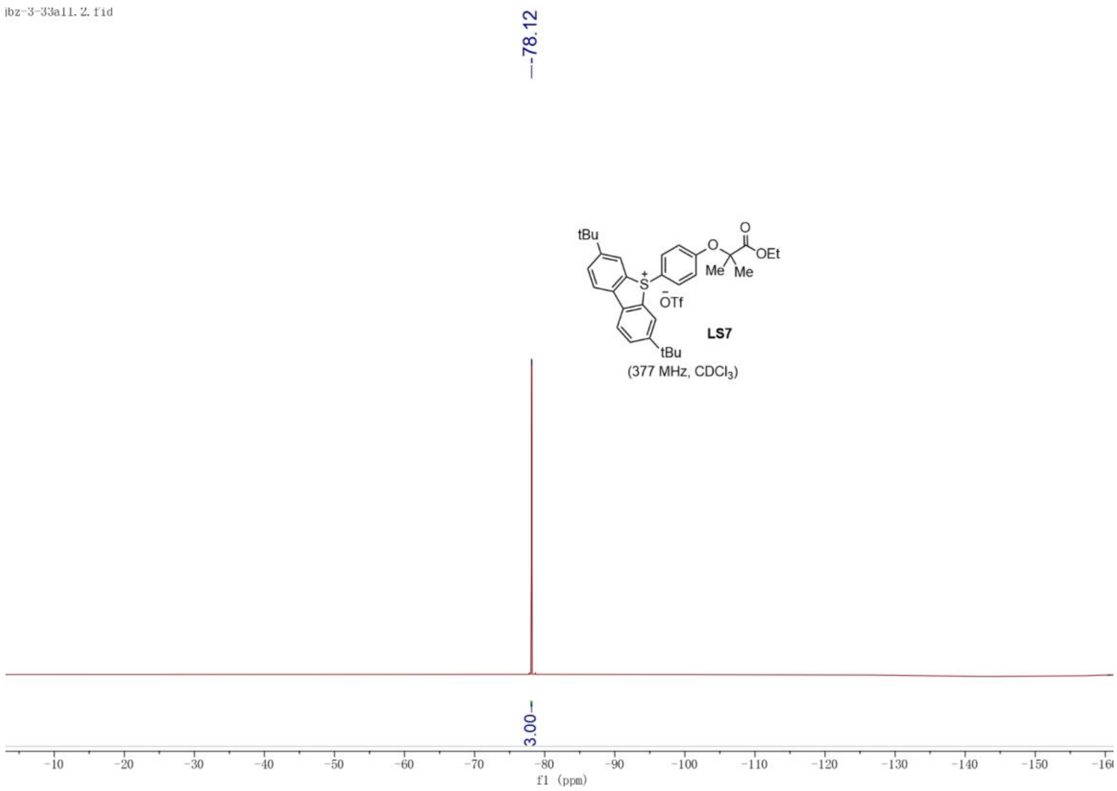
jbz-3-33all.2.fid
-78.12
tBu
OTf
O
OEt
Me
Me
tBu
LS7
(377 MHz, CDCl3)
3.00
f1 (ppm)

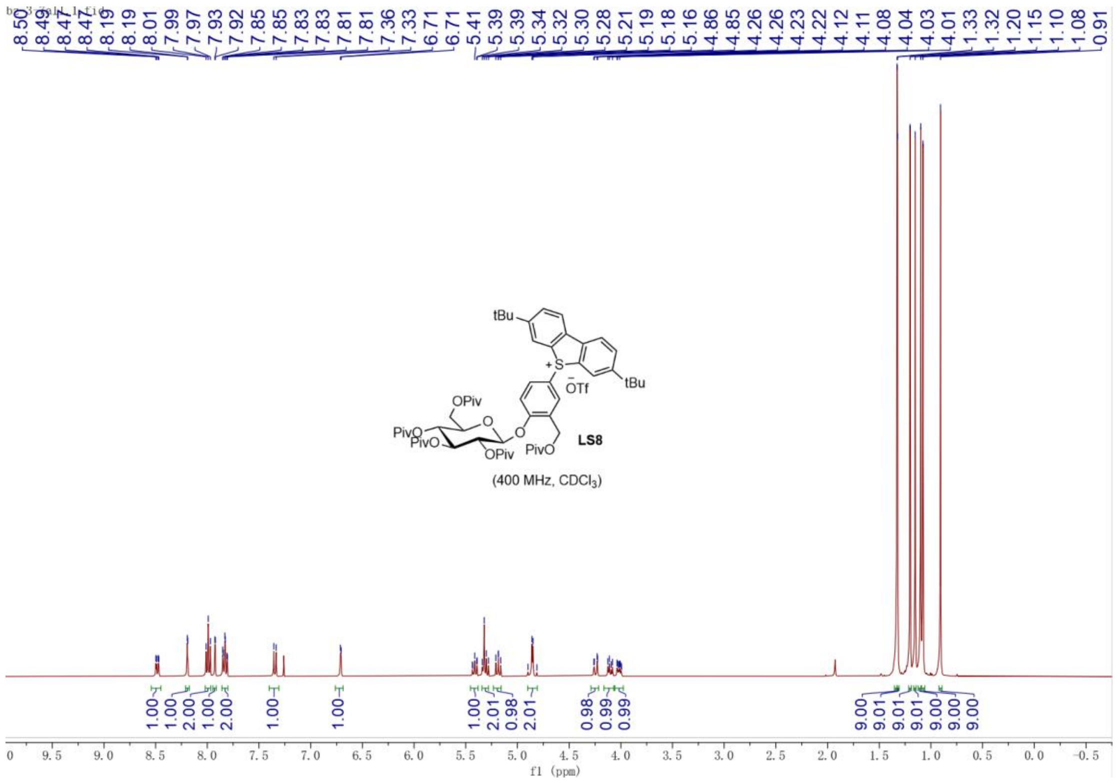
8.50
8.49
8.47
8.47
8.19
8.19
8.01
7.99
7.97
7.93
7.92
7.85
7.83
7.83
7.81
7.81
7.36
7.33
6.71
6.71
5.41
5.39
5.39
5.34
5.32
5.30
5.28
5.21
5.19
5.18
5.16
4.86
4.85
4.26
4.26
4.23
4.22
4.12
4.11
4.08
4.04
4.03
4.01
1.33
1.32
1.20
1.15
1.10
1.08
0.91
tBu
OTf
tBu
OPiv
PivO
PivO
OPiv
PivO
LS8
(400 MHz, CDCl3)
1.00
1.00
2.00
1.00
2.00
1.00
1.00
1.00
2.01
0.98
2.01
0.98
0.99
0.99
9.00
9.01
9.01
9.00
9.00
9.00
f1 (ppm)

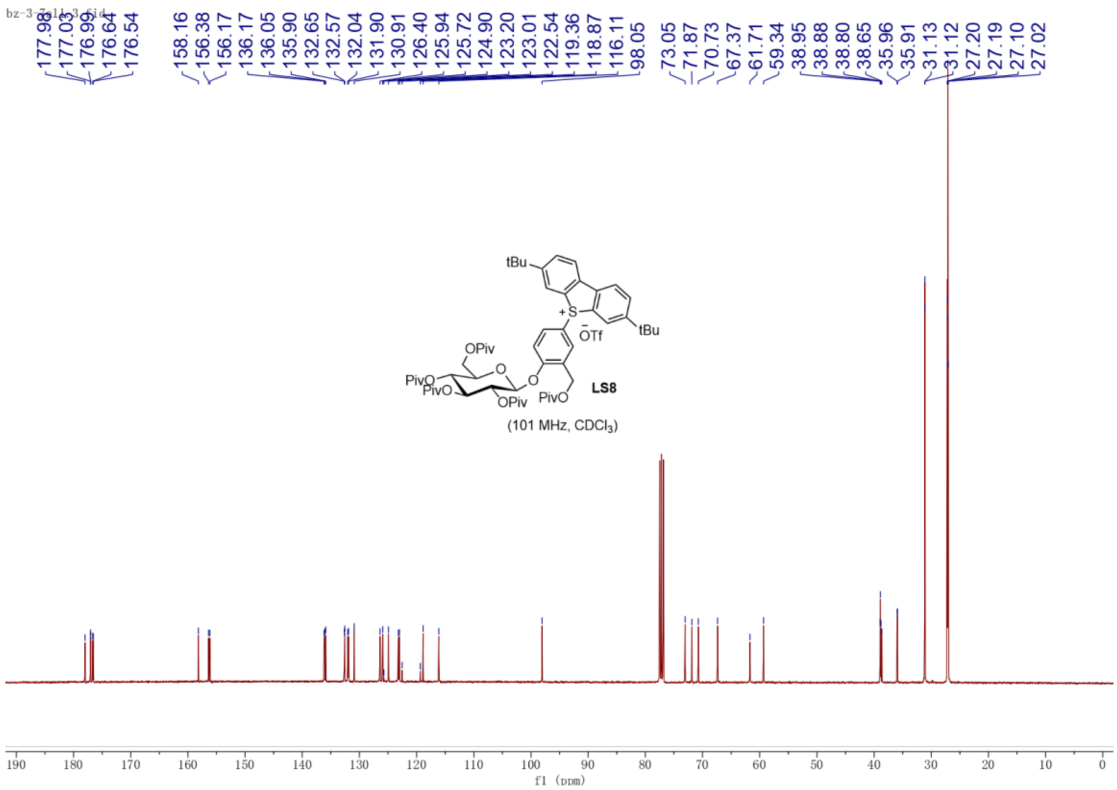
bz-3-7all.3.fid
177.98
177.03
176.99
176.64
176.54
158.16
156.38
156.17
136.17
136.05
135.90
132.65
132.57
132.04
131.90
130.91
126.40
125.94
125.72
124.90
123.20
123.01
122.54
119.36
118.87
116.11
98.05
73.05
71.87
70.73
67.37
61.71
59.34
38.95
38.88
38.80
38.65
35.96
35.91
31.13
31.12
27.20
27.19
27.10
27.02
tBu
S
OTf
tBu
OPiv
O
PivO
PivO
OPiv
O
PivO
LS8
(101 MHz, CDCl3)
f1 (ppm)

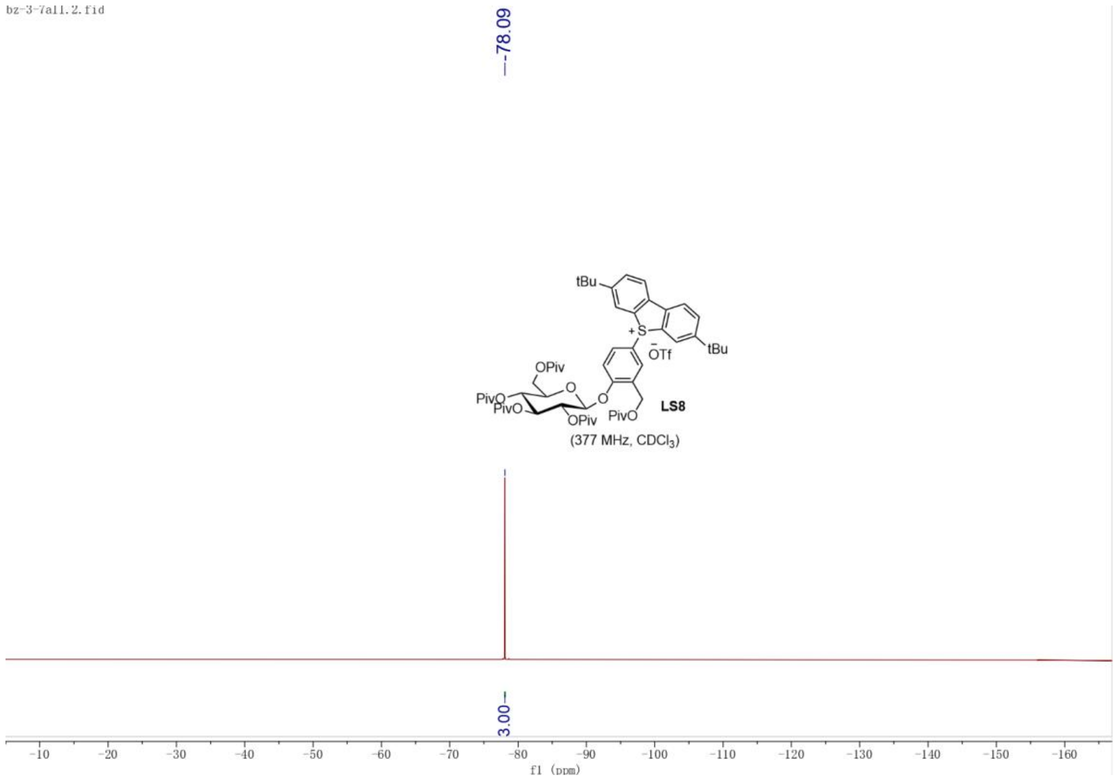
bz-3-7all.2.fid
-78.09
tBu
S
OTf
tBu
OPiv
O
PivO
PivO
OPiv
O
PivO
LS8
(377 MHz, CDCl3)
3.00
f1 (ppm)

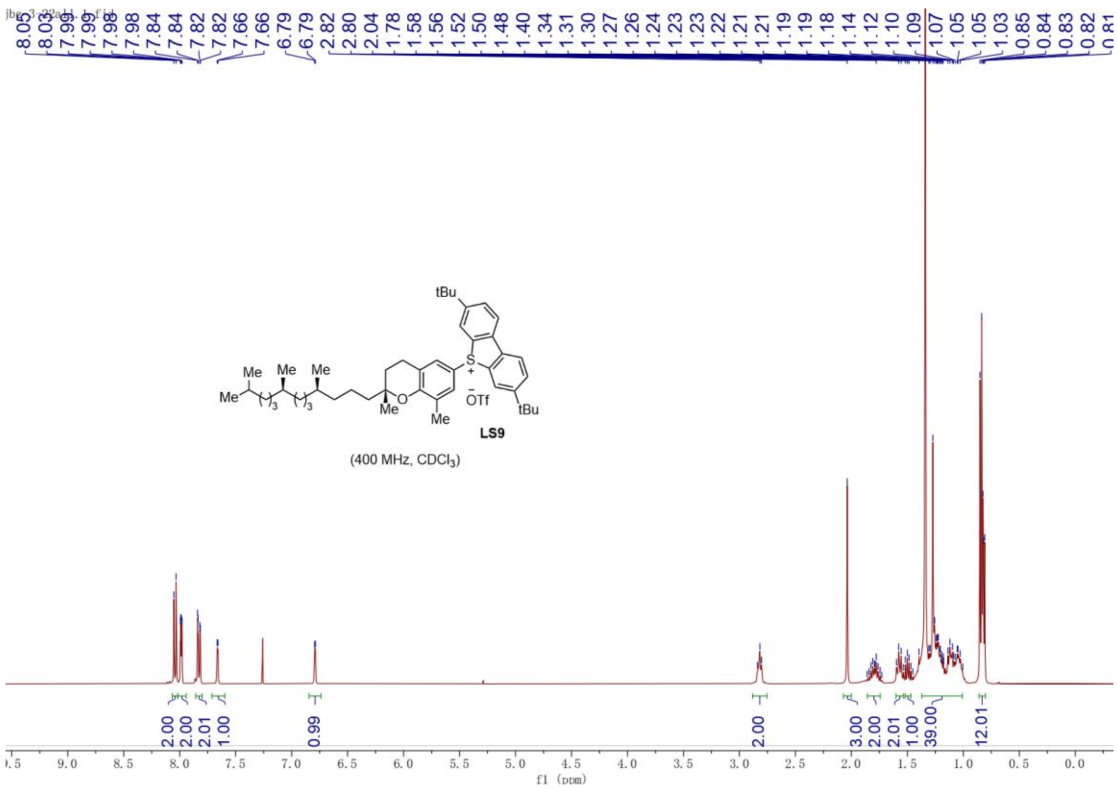

LS9
(400 MHz, CDCl3)


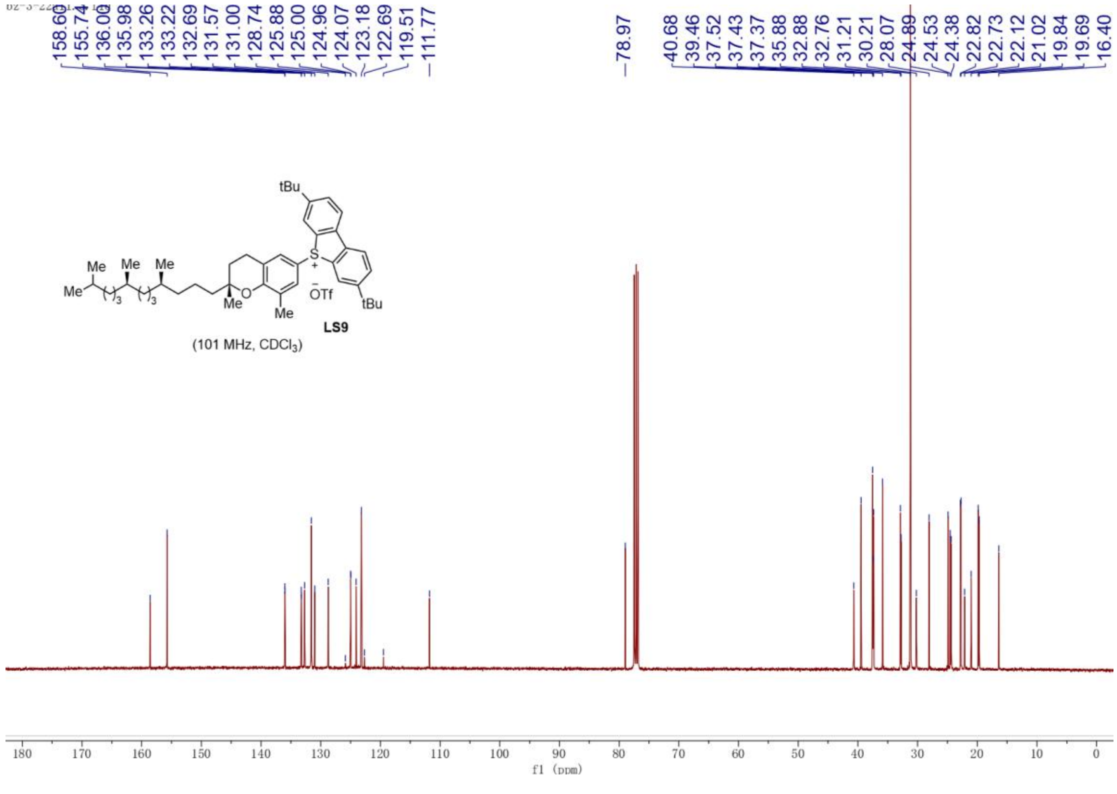

LS9
(101 MHz, CDCl3)

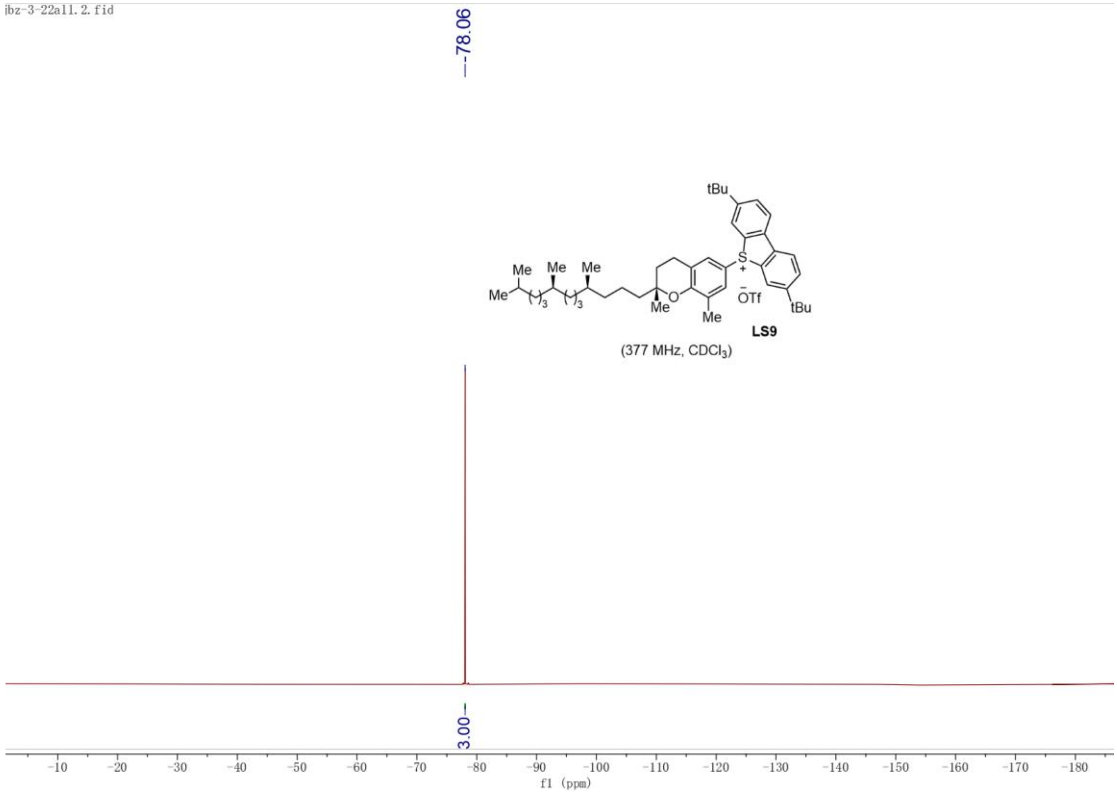
jbz-3-22all.2.fid
-78.06
tBu
S
OTf
tBu
Me
Me
Me
Me
O
Me
Me
LS9
(377 MHz, CDCl3)
3.00
f1 (ppm)

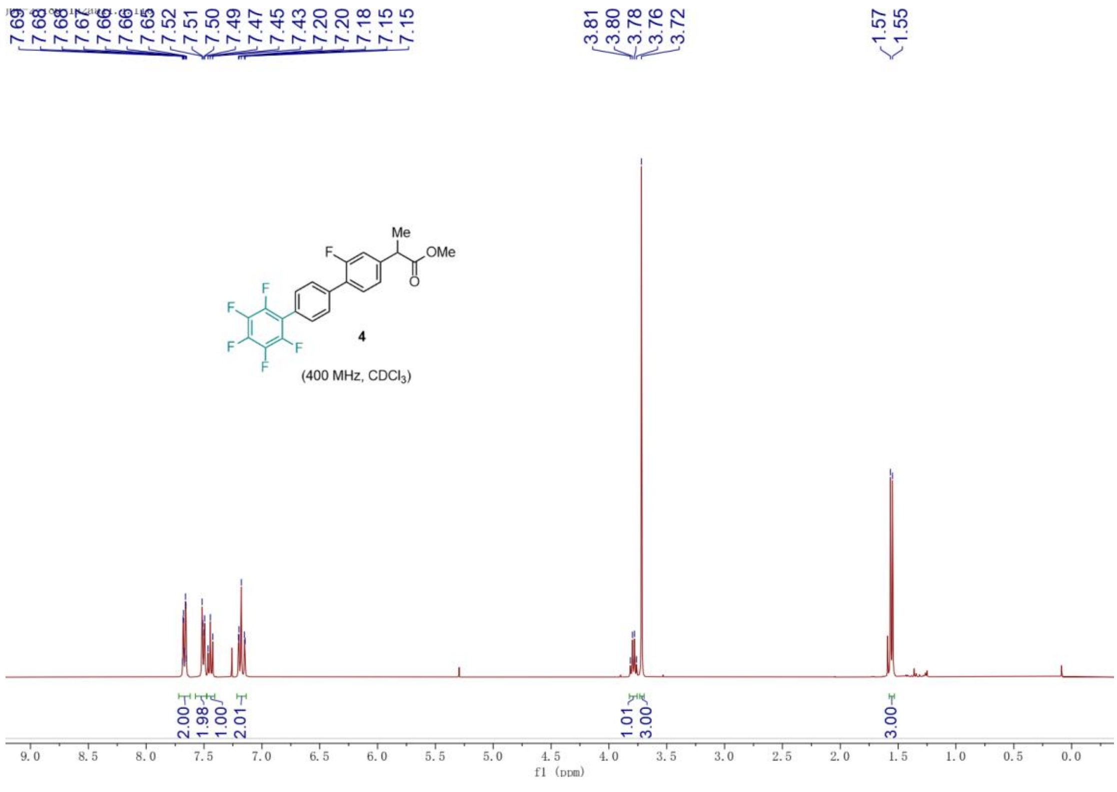
7.69 7.68 7.68 7.67 7.66 7.66 7.65 7.52 7.51 7.50 7.49 7.47 7.45 7.43 7.20 7.20 7.18 7.15 7.15
3.81 3.80 3.78 3.76 3.72
1.57 1.55
Me
F
OMe
O
F
F
F
F
F
4
(400 MHz, CDCl3)
2.00 1.98 1.00 2.01
1.01 3.00
3.00
f1 (ppm)

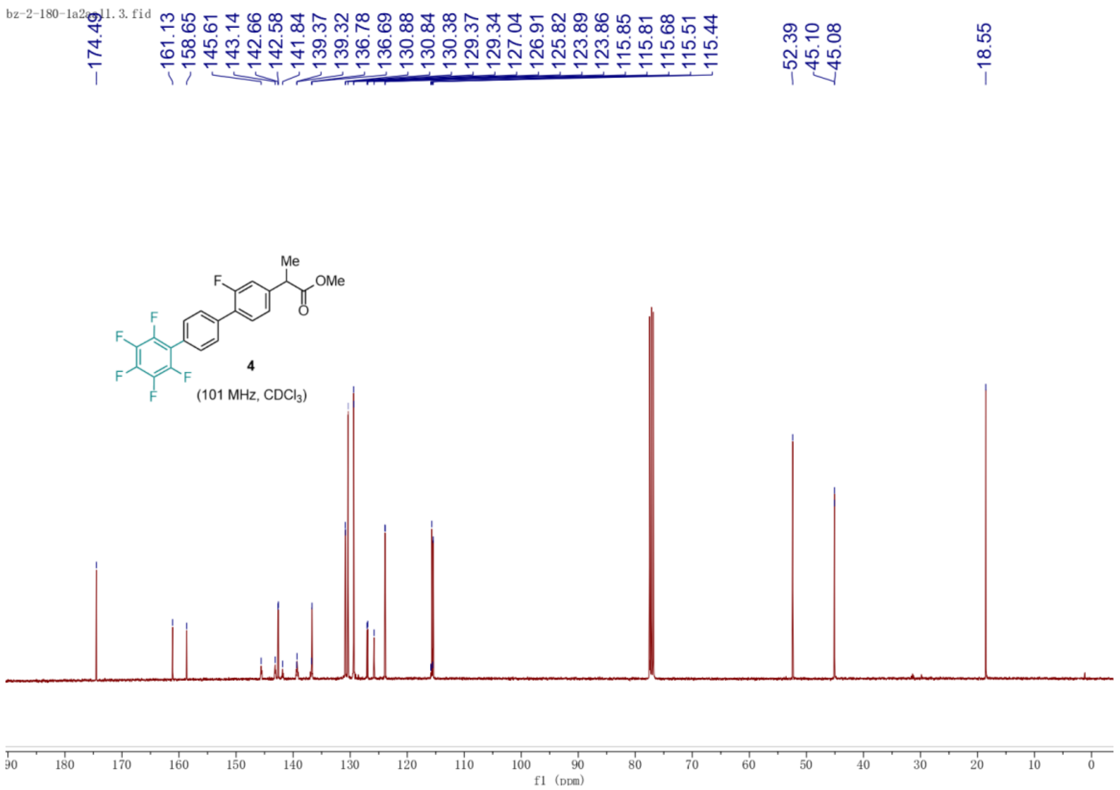
bz-2-180-1a2aall.3.fid
174.49
161.13
158.65
145.61
143.14
142.66
142.58
141.84
139.37
139.32
136.78
136.69
130.88
130.84
130.38
129.37
129.34
127.04
126.91
125.82
123.89
123.86
115.85
115.81
115.68
115.51
115.44
52.39
45.10
45.08
18.55
Me
F
OMe
O
F
F
F
F
F
4
(101 MHz, CDCl3)
f1 (ppm)

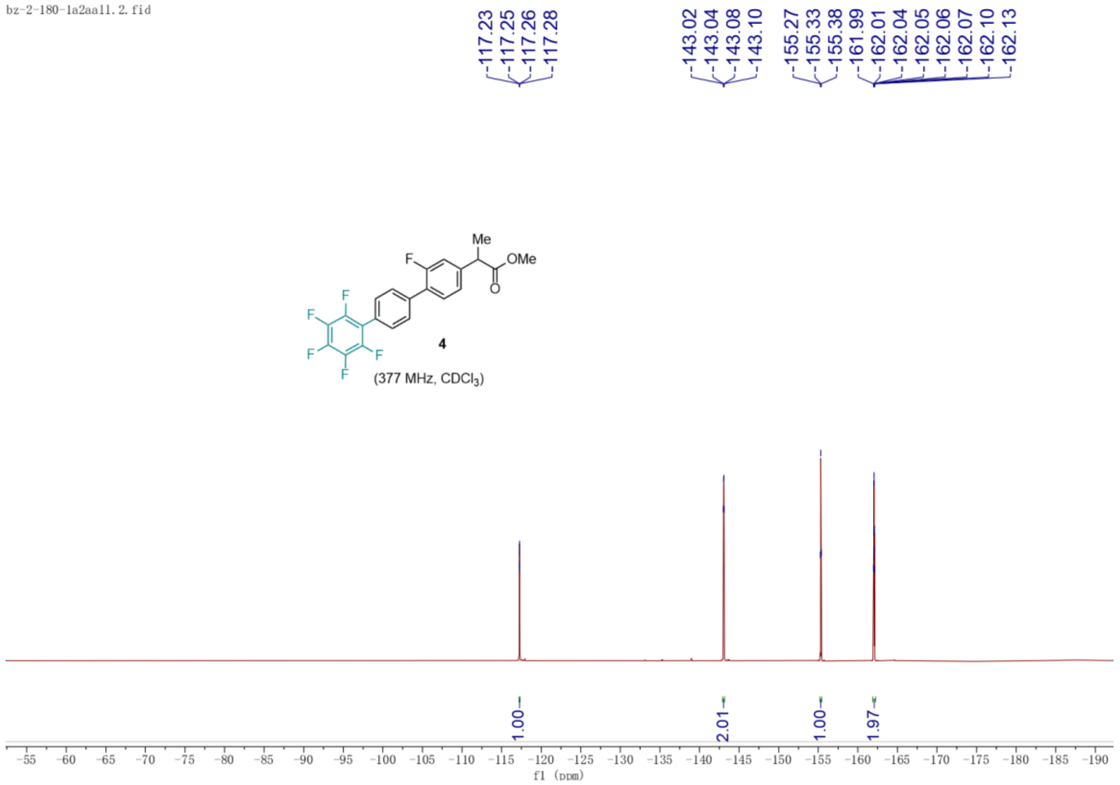
bz-2-180-1a2aall.2.fid
-117.23
-117.25
-117.26
-117.28
-143.02
-143.04
-143.08
-143.10
-155.27
-155.33
-155.38
-161.99
-162.01
-162.04
-162.05
-162.06
-162.07
-162.10
-162.13
Me
F
OMe
O
F
F
F
F
F
4
(377 MHz, CDCl3)
1.00
2.01
1.00
1.97
f1 (ppm)

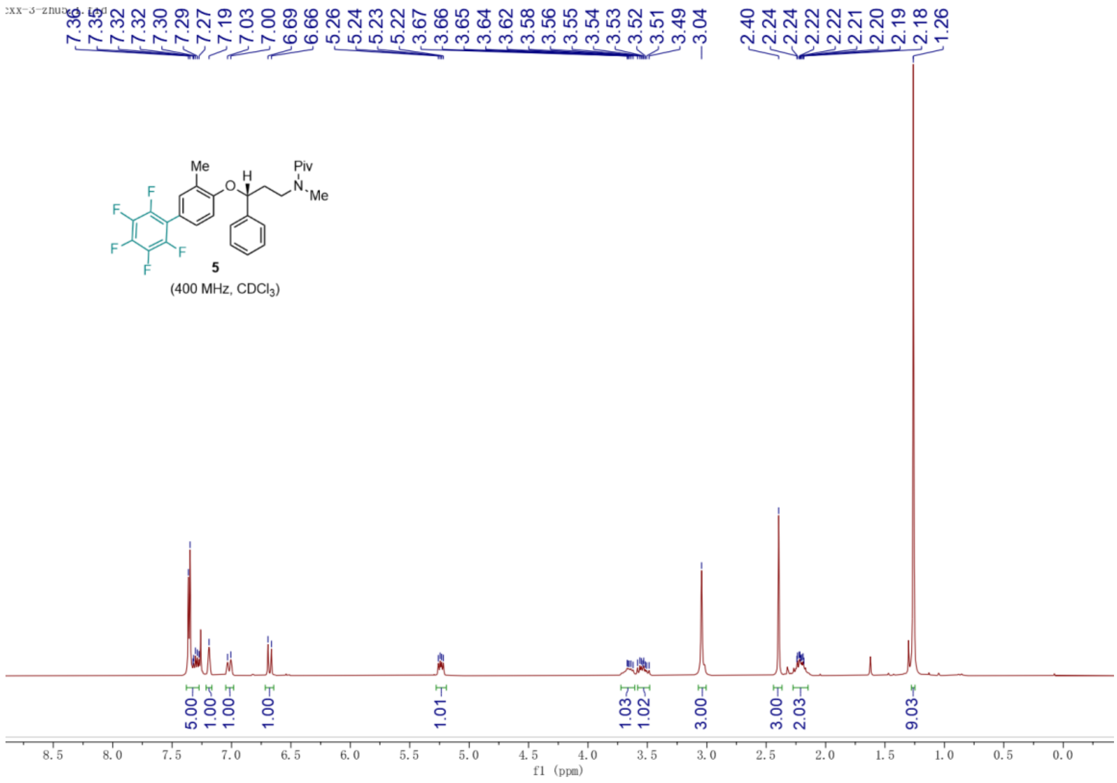
7.36 7.35 7.32 7.32 7.30 7.29 7.27 7.19 7.03 7.00 6.69 6.66 5.26 5.24 5.23 5.22 3.67 3.66 3.65 3.64 3.62 3.58 3.56 3.55 3.54 3.53 3.52 3.51 3.49 3.04 2.40 2.24 2.24 2.22 2.22 2.21 2.20 2.19 2.18 1.26
Me
Piv
H
O
N
Me
F
F
F
F
F
5
(400 MHz, CDCl3)
5.00
1.00
1.00
1.00
1.01
1.03
1.02
3.00
3.00
2.03
9.03
f1 (ppm)

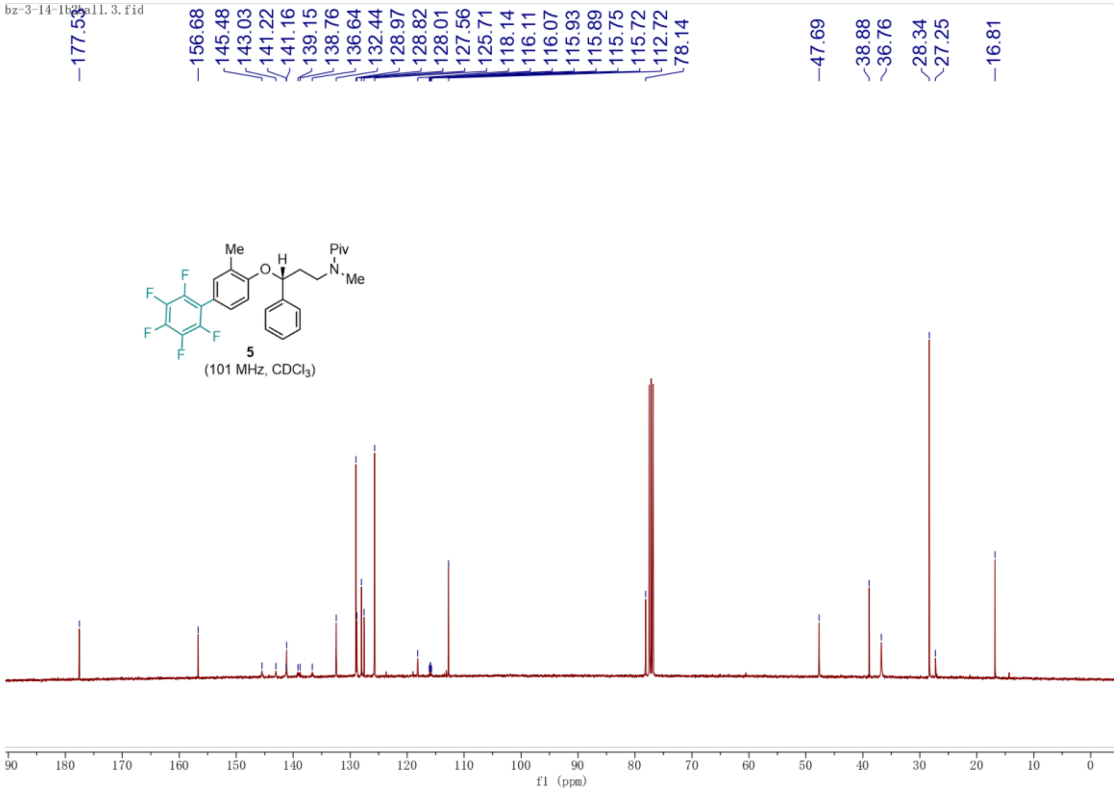
177.53
156.68 145.48 143.03 141.22 141.16 139.15 138.76 136.64 132.44 128.97 128.82 128.01 127.56 125.71 118.14 116.11 116.07 115.93 115.89 115.75 115.72 112.72 78.14
47.69 38.88 36.76 28.34 27.25 16.81
Me
Piv
H
O
N
Me
F
F
F
F
F
5
(101 MHz, CDCl3)
f1 (ppm)

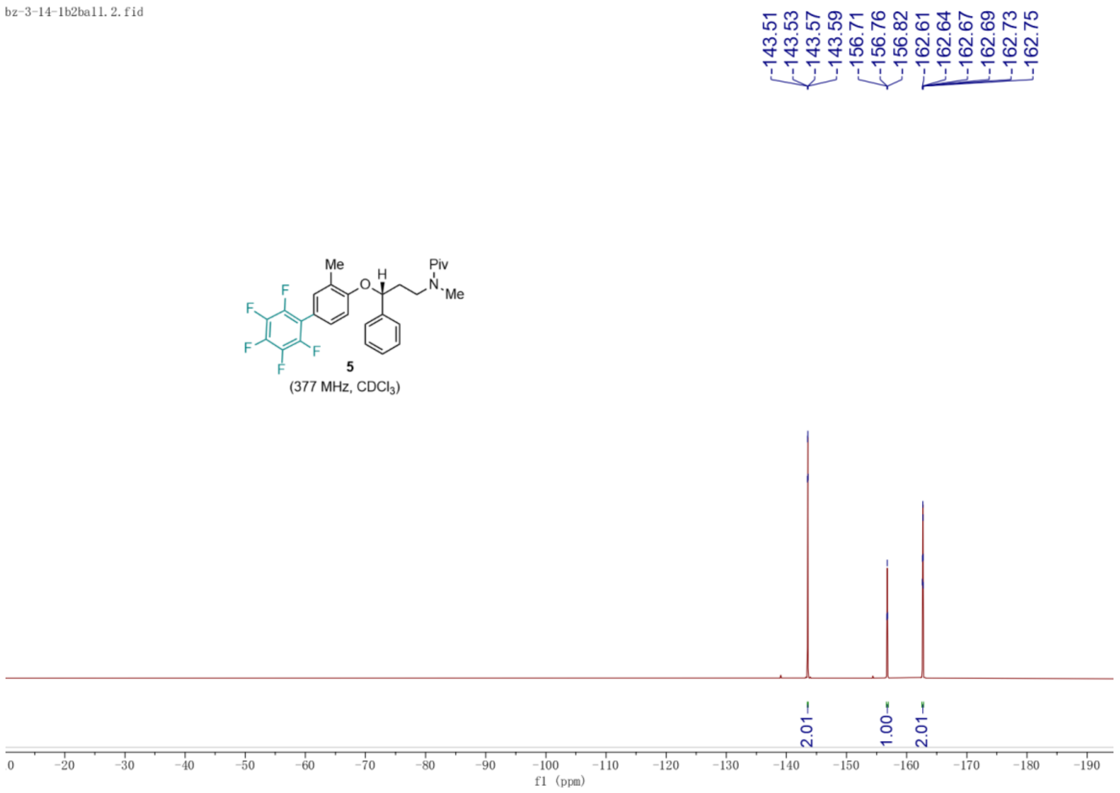
bz-3-14-1b2ba11.2.fid
-143.51
-143.53
-143.57
-143.59
-156.71
-156.76
-156.82
-162.61
-162.64
-162.67
-162.69
-162.73
-162.75
Me
H
O
Piv
N
Me
F
F
F
F
F
5
(377 MHz, CDCl3)
2.01
1.00
2.01
f1 (ppm)

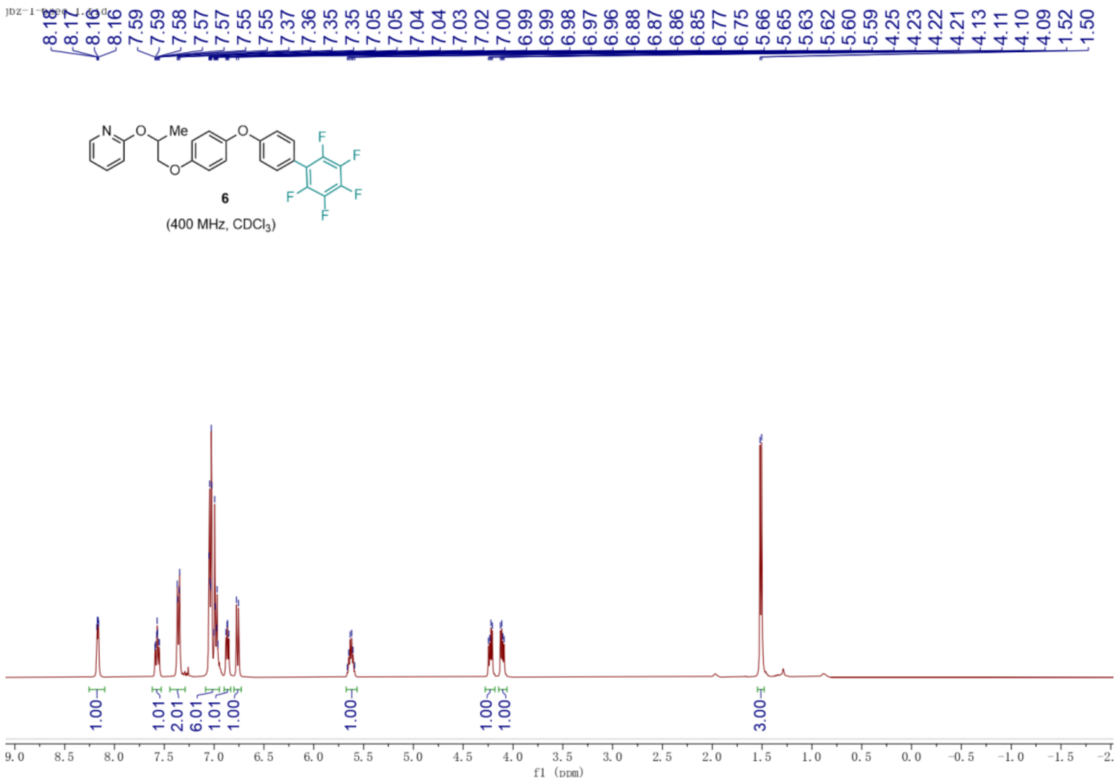
8.18
8.17
8.16
8.16
7.59
7.59
7.58
7.57
7.57
7.55
7.55
7.37
7.36
7.35
7.35
7.05
7.05
7.04
7.04
7.03
7.02
7.00
6.99
6.98
6.97
6.96
6.88
6.87
6.86
6.85
6.77
6.75
5.66
5.65
5.63
5.62
5.60
5.59
4.25
4.23
4.22
4.21
4.13
4.11
4.10
4.09
1.52
1.50
N
O
Me
O
O
F
F
F
F
F
6
(400 MHz, CDCl3)
1.00
1.01
2.01
6.01
1.01
1.00
1.00
1.00
1.00
3.00
f1 (ppm)

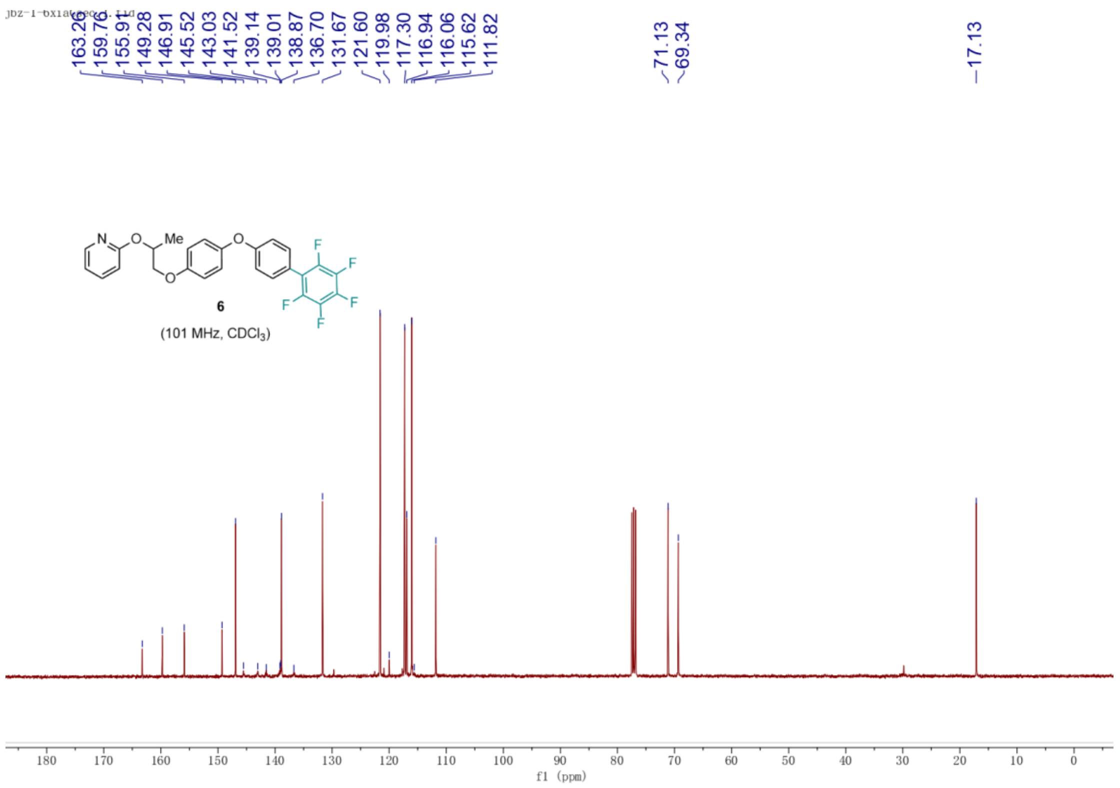

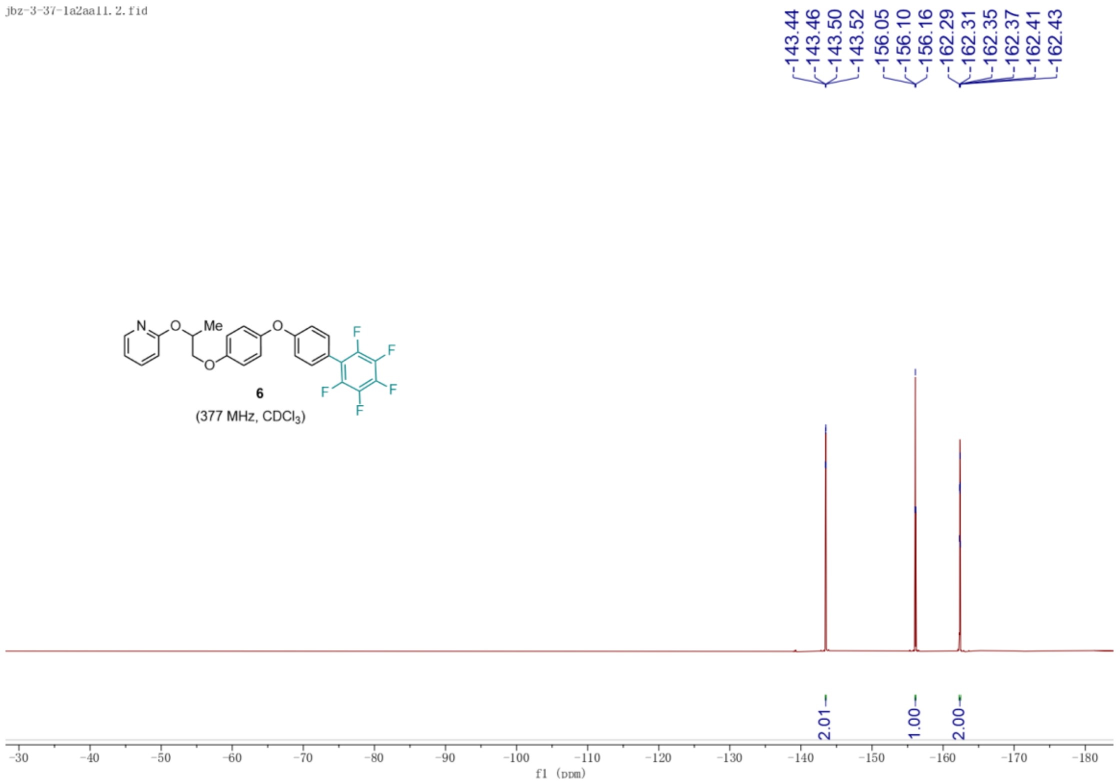

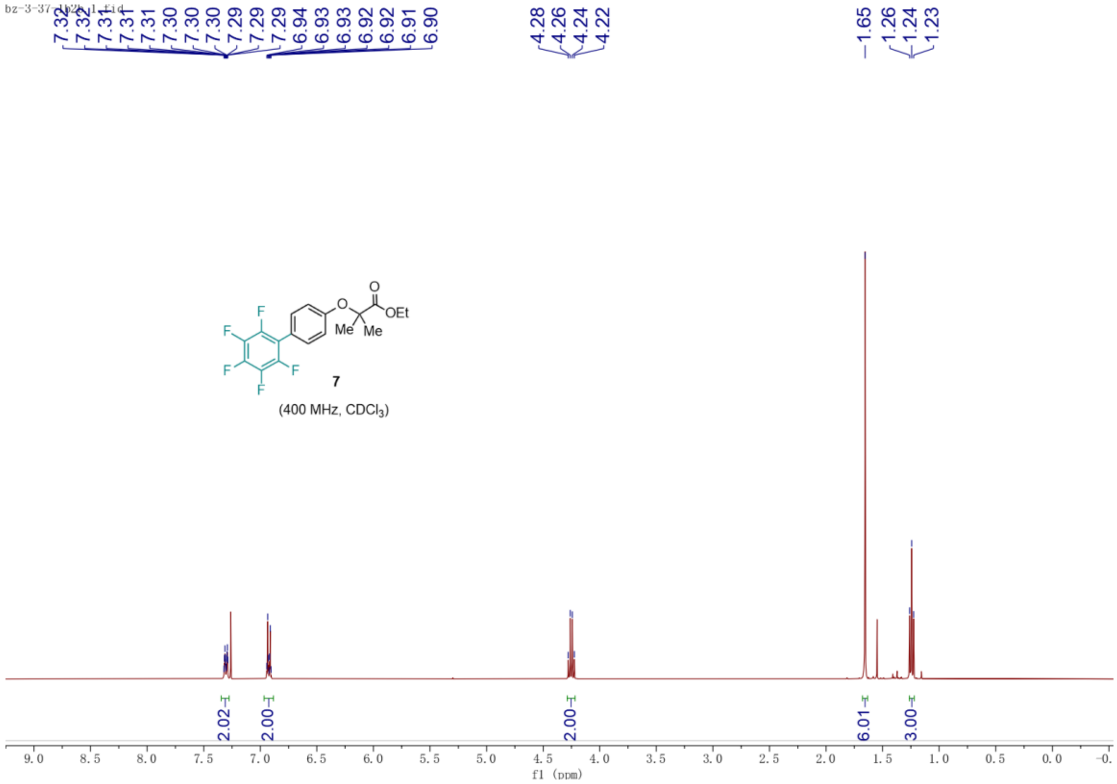

bz-3-37-1b2b.1.fid
7.32 7.32 7.31 7.31 7.30 7.30 7.29 7.29 6.94 6.93 6.93 6.92 6.92 6.91 6.90
4.28 4.26 4.24 4.22
1.65 1.26 1.24 1.23
OEt
Me
Me
F
7
(400 MHz, CDCl3)
2.02
2.00
2.00
6.01
3.00
f1 (ppm)


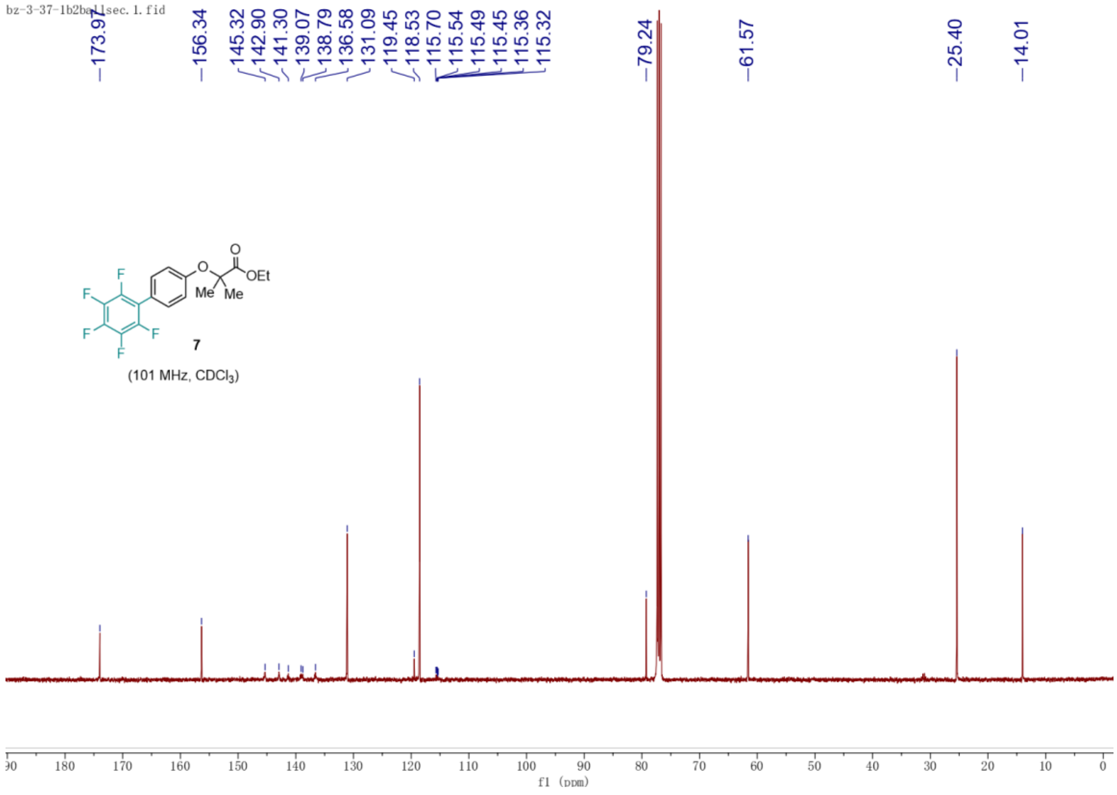

bz-3-37-1b2ball sec.1.fid
173.97
156.34
145.32 142.90 141.30 139.07 138.79 136.58 131.09 119.45 118.53 115.70 115.54 115.49 115.45 115.36 115.32
79.24
61.57
25.40
14.01
OEt
Me
Me
F
7
(101 MHz, CDCl3)
f1 (ppm)

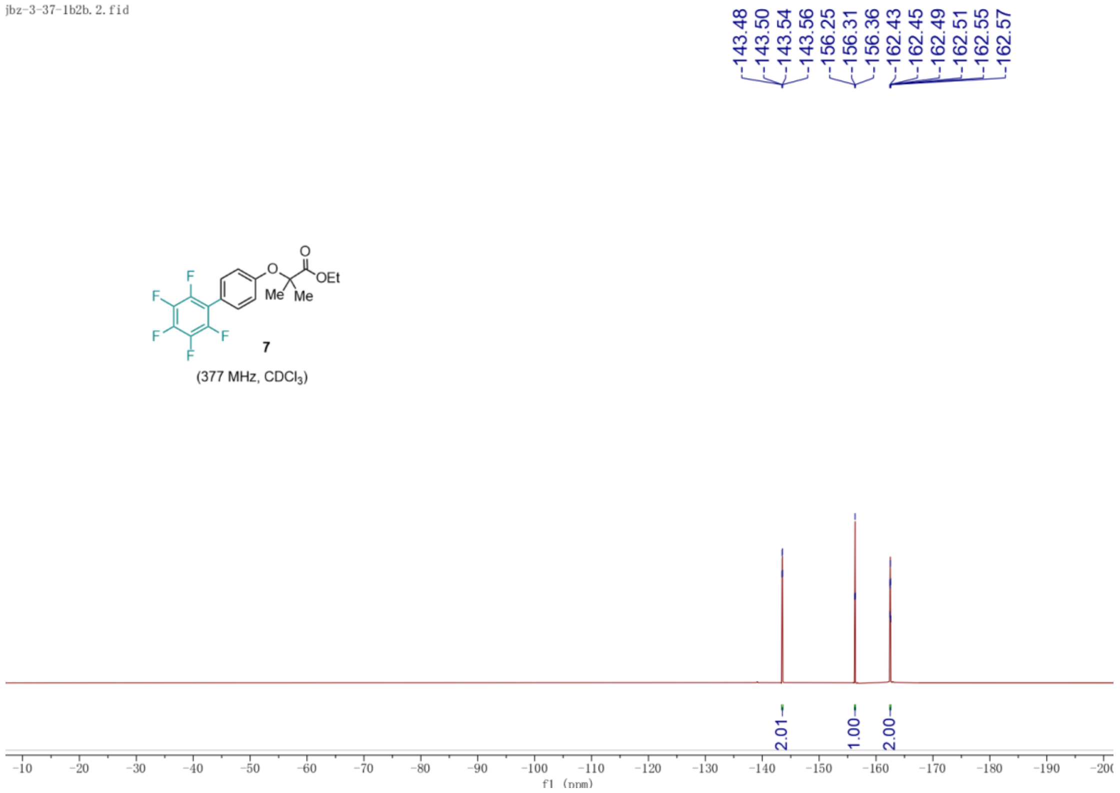
jbz-3-37-1b2b.2.fid
-143.48
-143.50
-143.54
-143.56
-156.25
-156.31
-156.36
-162.43
-162.45
-162.49
-162.51
-162.55
-162.57
F
F
F
F
F
O
O
OEt
Me
Me
7
(377 MHz, CDCl3)
2.01
1.00
2.00
f1 (ppm)

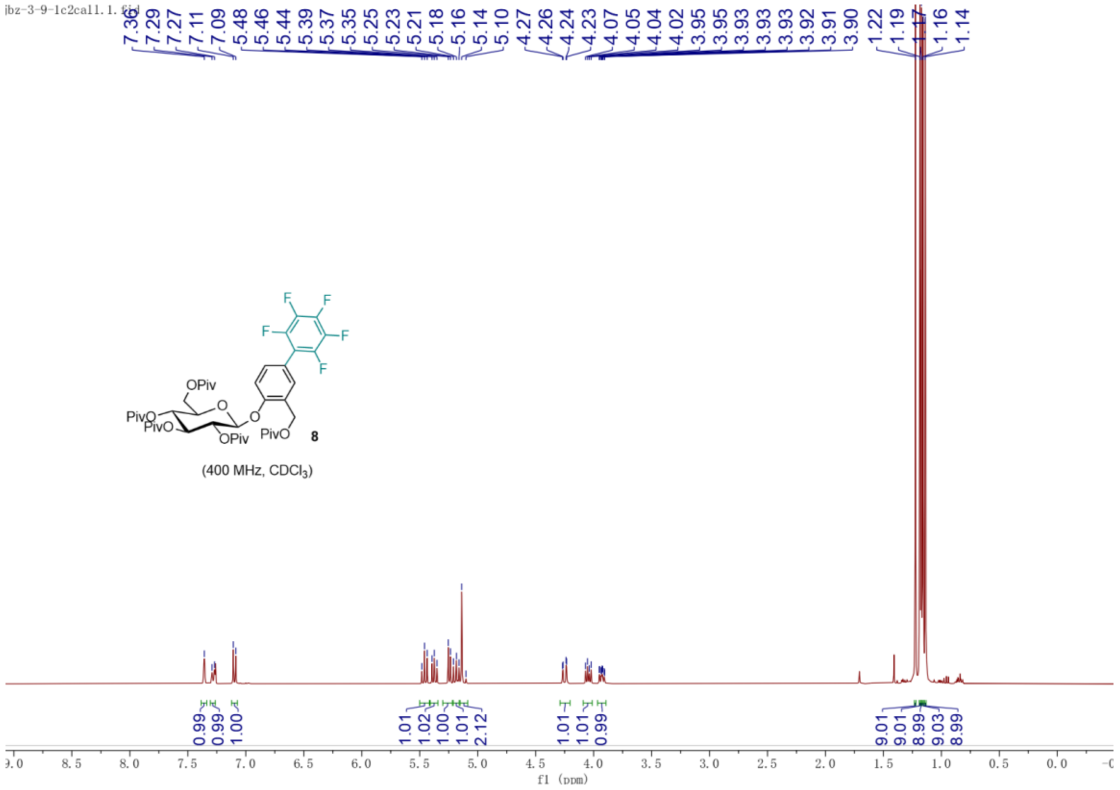
jbz-3-9-1c2call.1.fid
7.36
7.29
7.27
7.11
7.09
5.48
5.46
5.44
5.39
5.37
5.35
5.25
5.23
5.21
5.18
5.16
5.14
5.10
4.27
4.26
4.24
4.23
4.07
4.05
4.04
4.02
3.95
3.93
3.93
3.93
3.92
3.91
3.90
1.22
1.19
1.17
1.16
1.14
OPiv
PivO
PivO
OPiv
PivO
O
O
8
(400 MHz, CDCl3)
0.99
0.99
1.00
1.01
1.02
1.00
1.01
2.12
1.01
1.01
0.99
9.01
9.01
8.99
9.03
8.99
f1 (ppm)

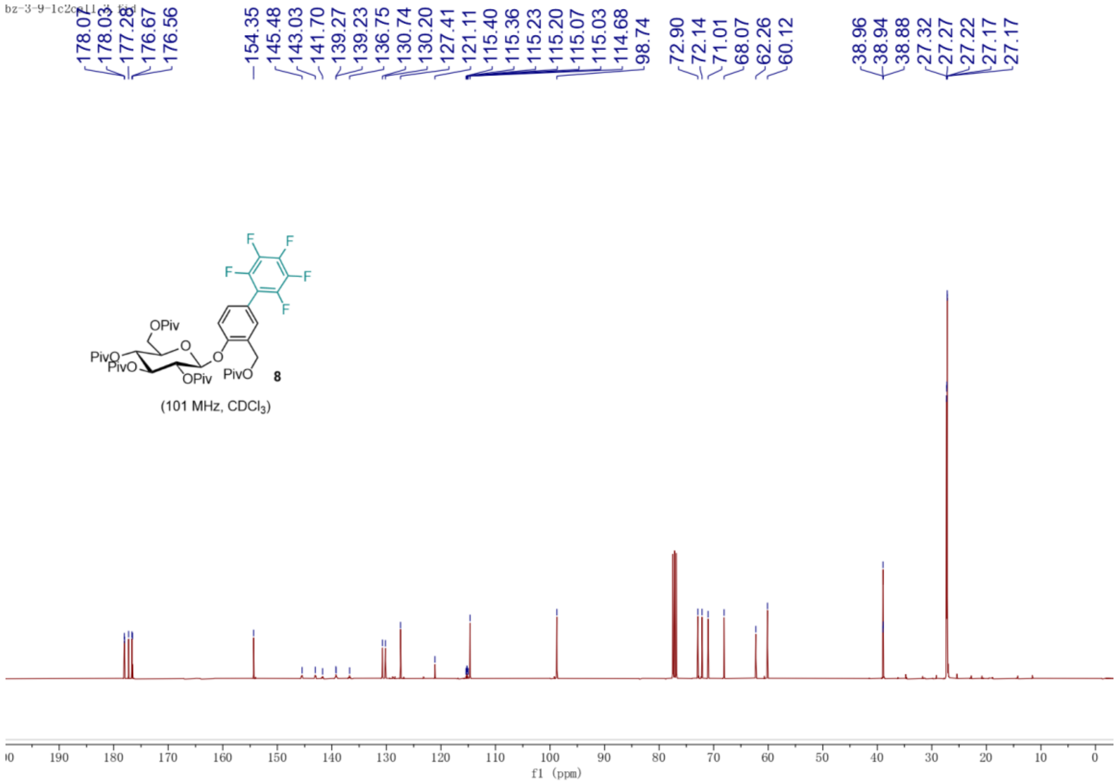

bz-3-9-1c2call.3.fid
178.07
178.03
177.28
176.67
176.56
154.35
145.48
143.03
141.70
139.27
139.23
136.75
130.74
130.20
127.41
121.11
115.40
115.36
115.23
115.20
115.07
115.03
114.68
98.74
72.90
72.14
71.01
68.07
62.26
60.12
38.96
38.94
38.88
27.32
27.27
27.22
27.17
27.17
8
(101 MHz, CDCl3)
f1 (ppm)


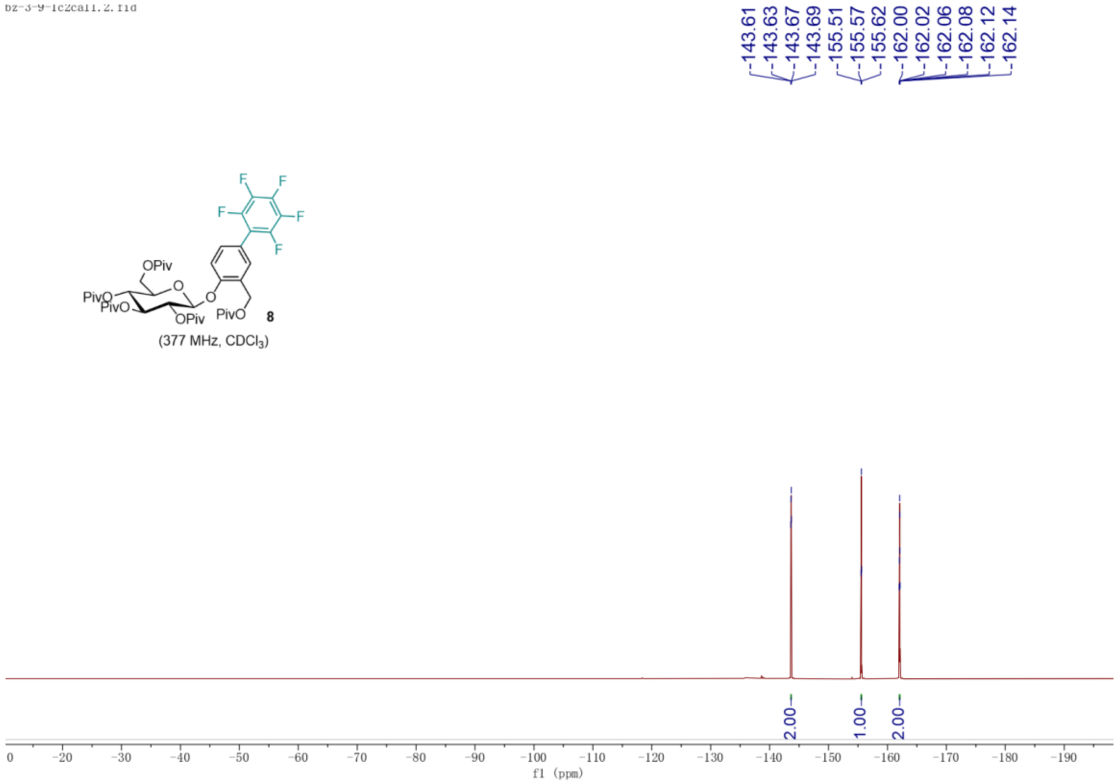

bz-3-9-1c2call.2.fid
-143.61
-143.63
-143.67
-143.69
-155.51
-155.57
-155.62
-162.00
-162.02
-162.06
-162.08
-162.12
-162.14
8
(377 MHz, CDCl3)
2.00
1.00
2.00
f1 (ppm)

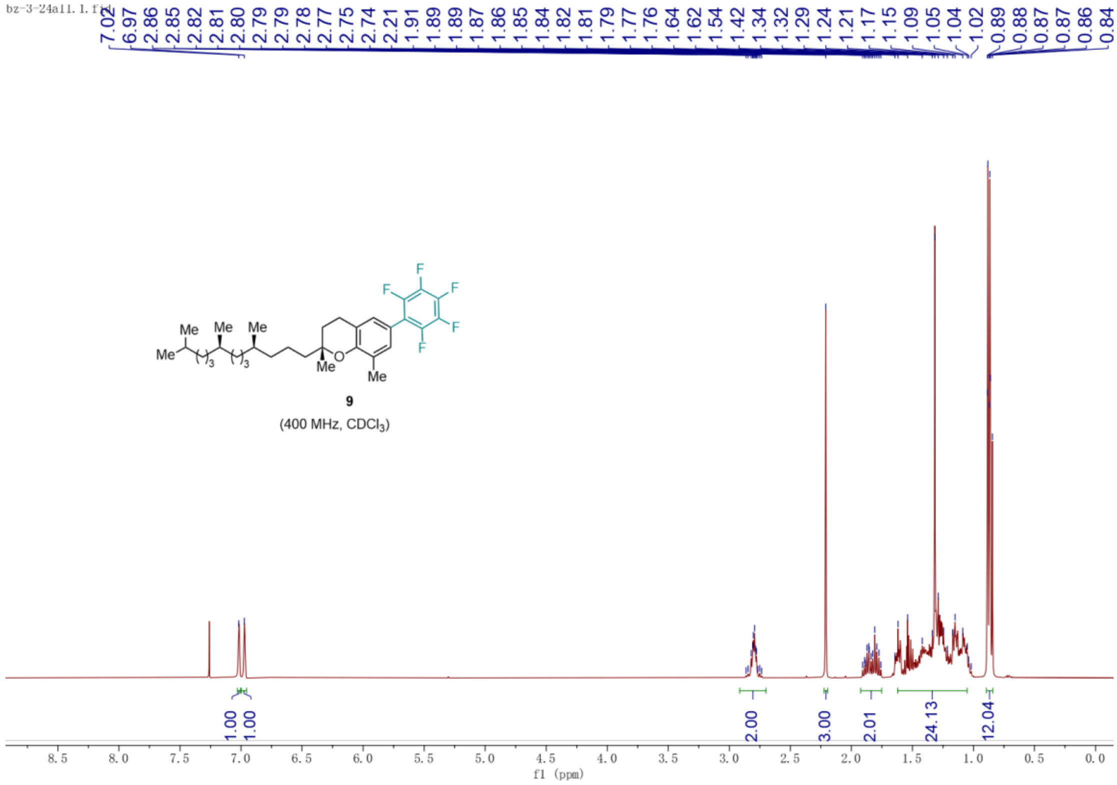
bz-3-24all.1.fid
7.02
6.97
2.86
2.85
2.82
2.81
2.80
2.79
2.79
2.78
2.77
2.75
2.74
2.21
1.91
1.89
1.89
1.87
1.86
1.85
1.84
1.82
1.81
1.79
1.77
1.76
1.64
1.62
1.54
1.42
1.34
1.32
1.29
1.24
1.21
1.17
1.15
1.09
1.05
1.04
1.02
0.89
0.88
0.87
0.87
0.86
0.84
Me
Me
Me
Me
O
Me
Me
F
F
F
F
F
9
(400 MHz, CDCl3)
1.00
1.00
2.00
3.00
2.01
24.13
12.04
f1 (ppm)

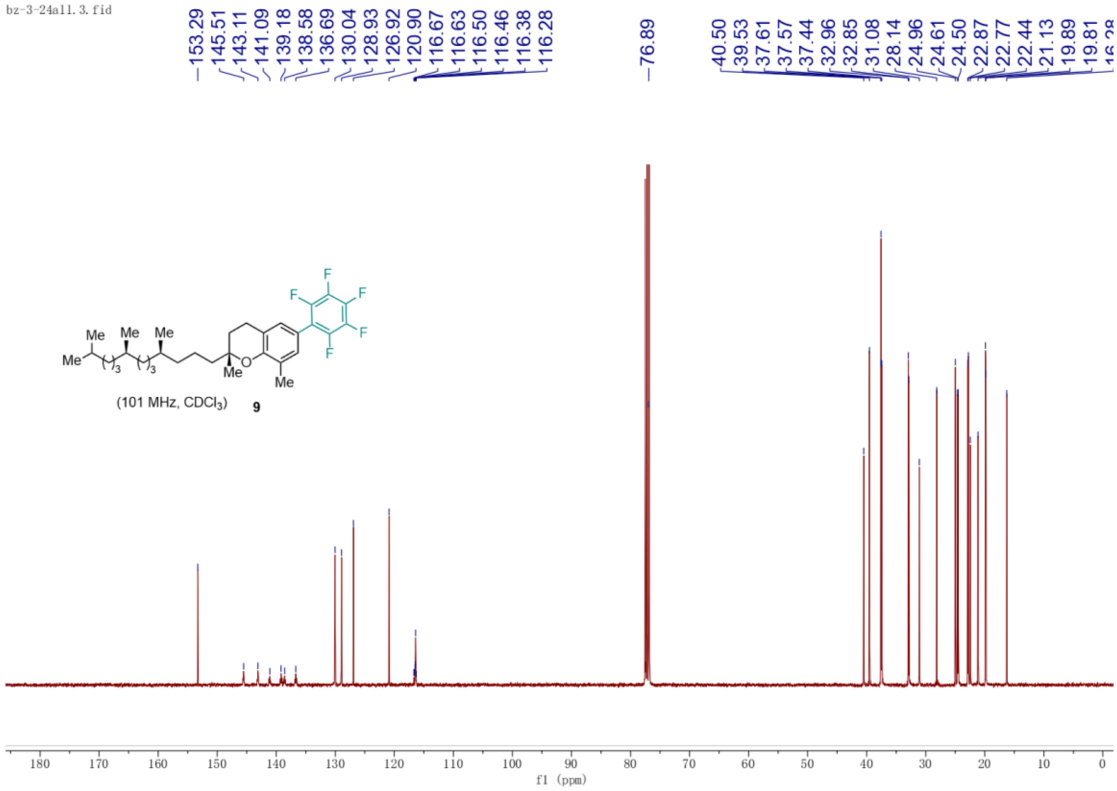
bz-3-24all.3.fid
153.29
145.51
143.11
141.09
139.18
138.58
136.69
130.04
128.93
126.92
120.90
116.67
116.63
116.50
116.46
116.38
116.28
76.89
40.50
39.53
37.61
37.57
37.44
32.96
32.85
31.08
28.14
24.96
24.61
24.50
22.87
22.77
22.44
21.13
19.89
19.81
(101 MHz, CDCl3)
9
f1 (ppm)

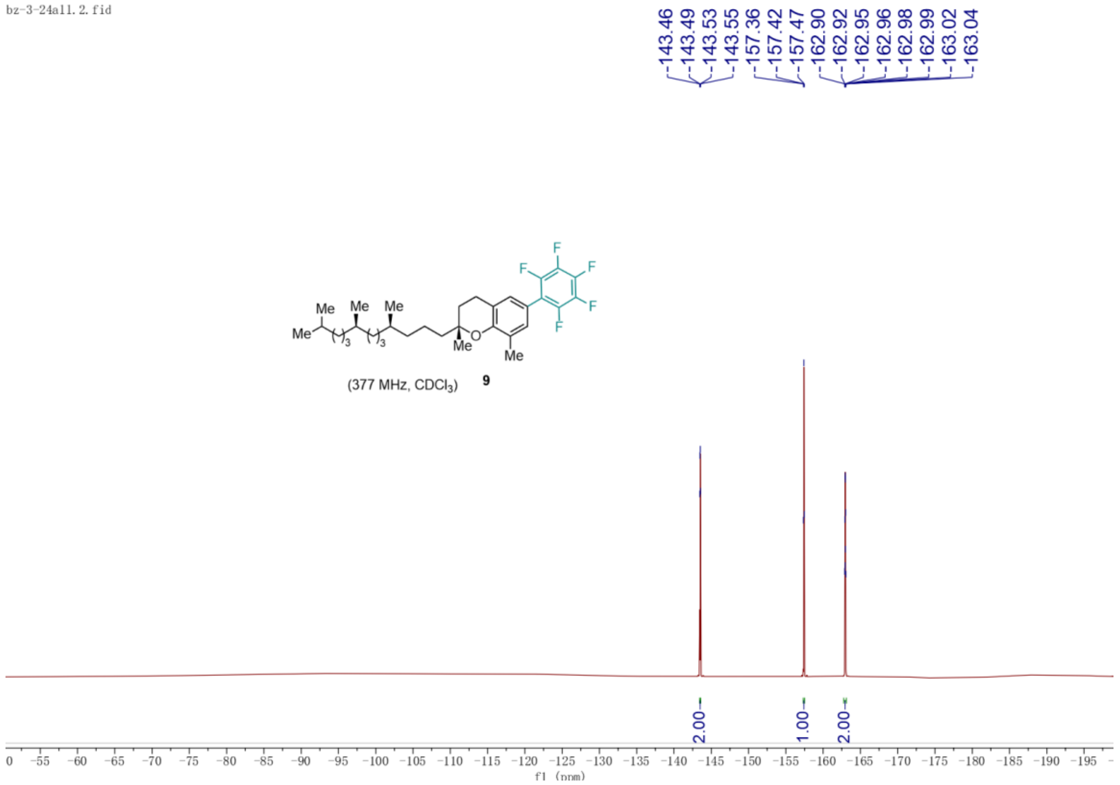
bz-3-24a11.2.fid
-143.46
-143.49
-143.53
-143.55
-157.36
-157.42
-157.47
-162.90
-162.92
-162.95
-162.96
-162.98
-162.99
-163.02
-163.04
(377 MHz, CDCl3)
9
2.00
1.00
2.00
f1 (ppm)

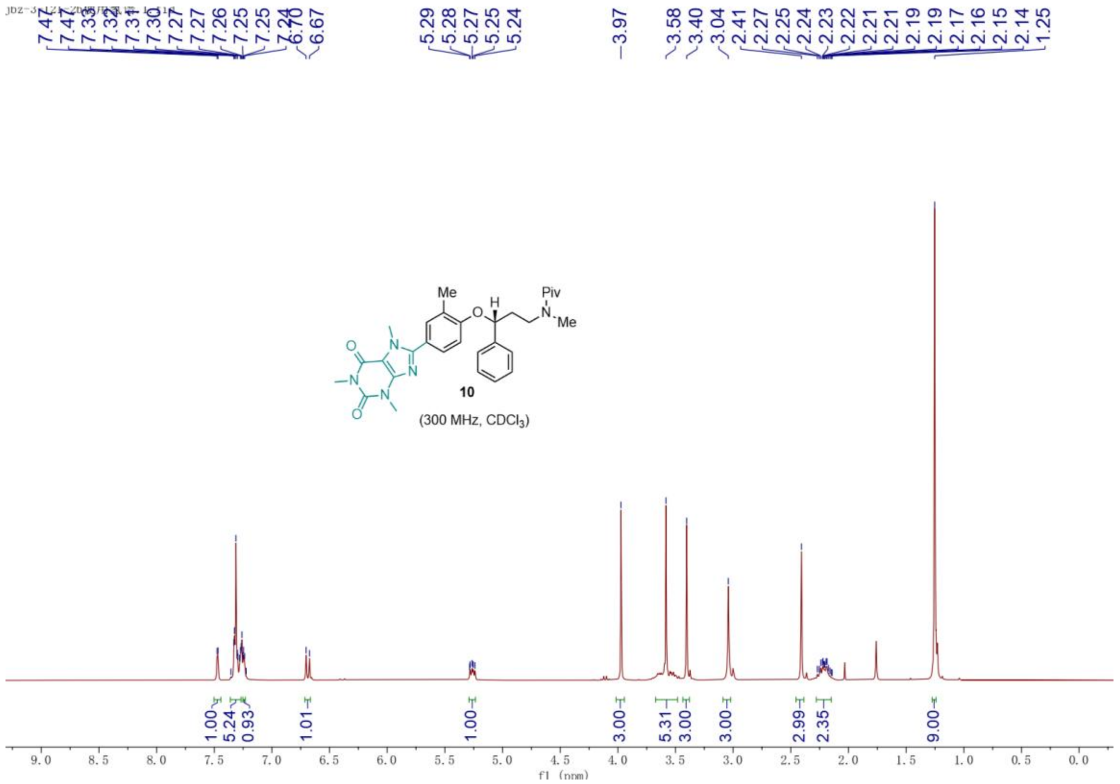
7.47
7.47
7.33
7.32
7.31
7.30
7.27
7.27
7.26
7.25
7.25
7.24
6.70
6.67
5.29
5.28
5.27
5.25
5.24
3.97
3.58
3.40
3.04
2.41
2.27
2.25
2.24
2.23
2.22
2.21
2.21
2.19
2.17
2.16
2.15
2.14
1.25
10
(300 MHz, CDCl3)
1.00
5.24
0.93
1.01
1.00
3.00
5.31
3.00
3.00
2.99
2.35
9.00
f1 (ppm)

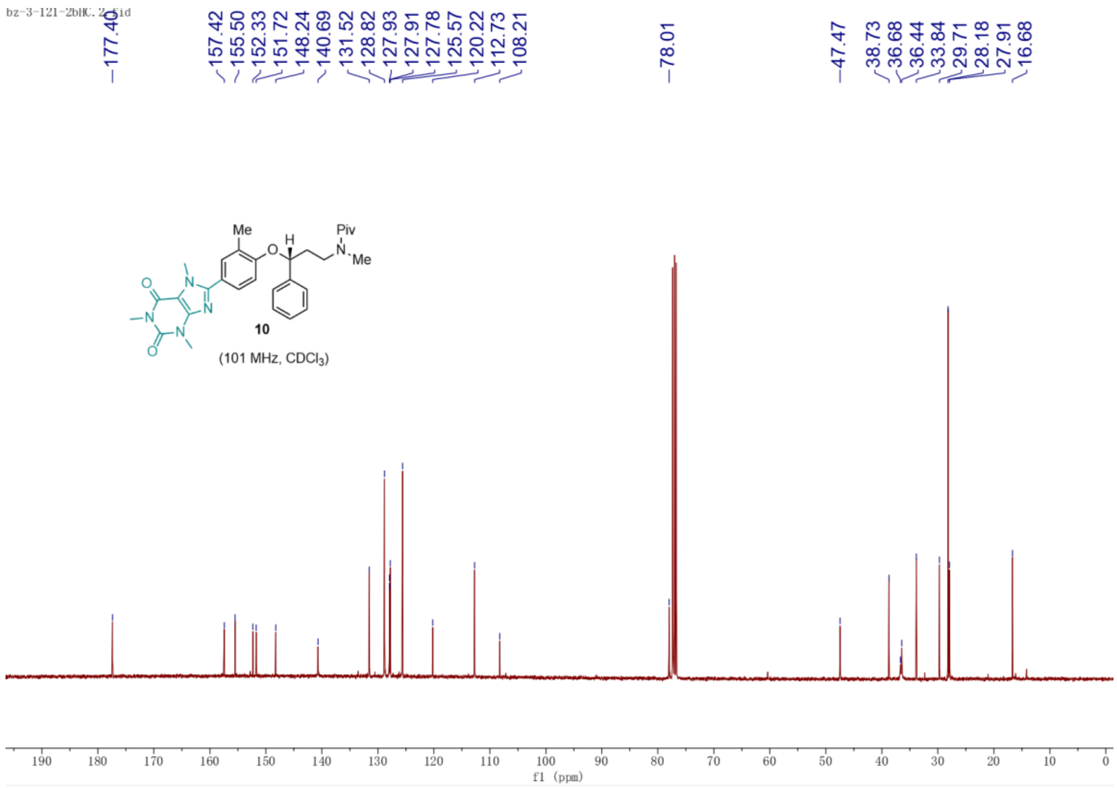
bz-3-121-2bHC.2.fid
177.40
157.42
155.50
152.33
151.72
148.24
140.69
131.52
128.82
127.93
127.91
127.78
125.57
120.22
112.73
108.21
78.01
47.47
38.73
36.68
36.44
33.84
29.71
28.18
27.91
16.68
Me
Piv
H
O
N
Me
O
N
N
N
N
O
10
(101 MHz, CDCl3)
f1 (ppm)

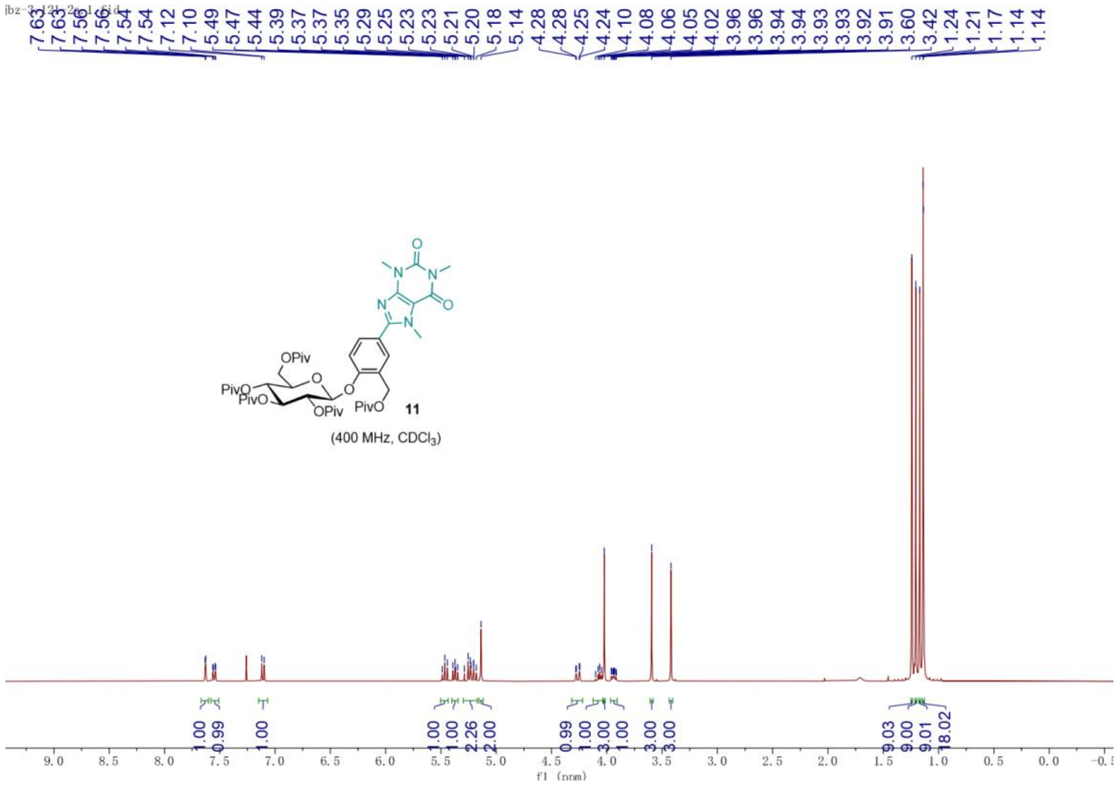
7.63
7.63
7.56
7.56
7.54
7.54
7.12
7.10
5.49
5.47
5.44
5.39
5.37
5.37
5.35
5.29
5.25
5.23
5.23
5.21
5.20
5.18
5.14
4.28
4.25
4.24
4.10
4.08
4.06
4.05
4.02
3.96
3.96
3.94
3.93
3.93
3.92
3.91
3.60
3.42
1.24
1.21
1.17
1.14
1.14
OPiv
PivO
PivO
OPiv
PivO
11
(400 MHz, CDCl3)
1.00
0.99
1.00
1.00
1.00
2.26
2.00
0.99
1.00
3.00
1.00
3.00
3.00
9.03
9.00
9.01
18.02
f1 (ppm)

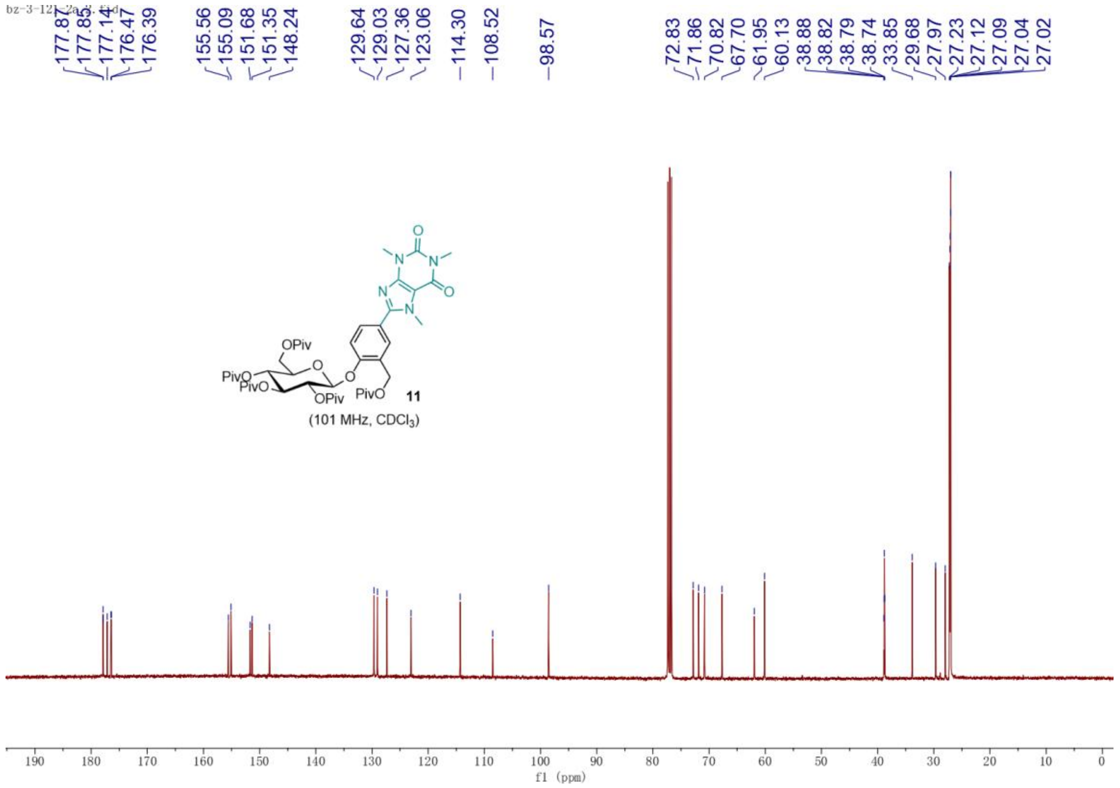
177.87
177.85
177.14
176.47
176.39
155.56
155.09
151.68
151.35
148.24
129.64
129.03
127.36
123.06
114.30
108.52
98.57
72.83
71.86
70.82
67.70
61.95
60.13
38.88
38.82
38.79
38.74
33.85
29.68
27.97
27.23
27.12
27.09
27.04
27.02
OPiv
PivO
PivO
OPiv
PivO
O
N
11
(101 MHz, CDCl3)
f1 (ppm)

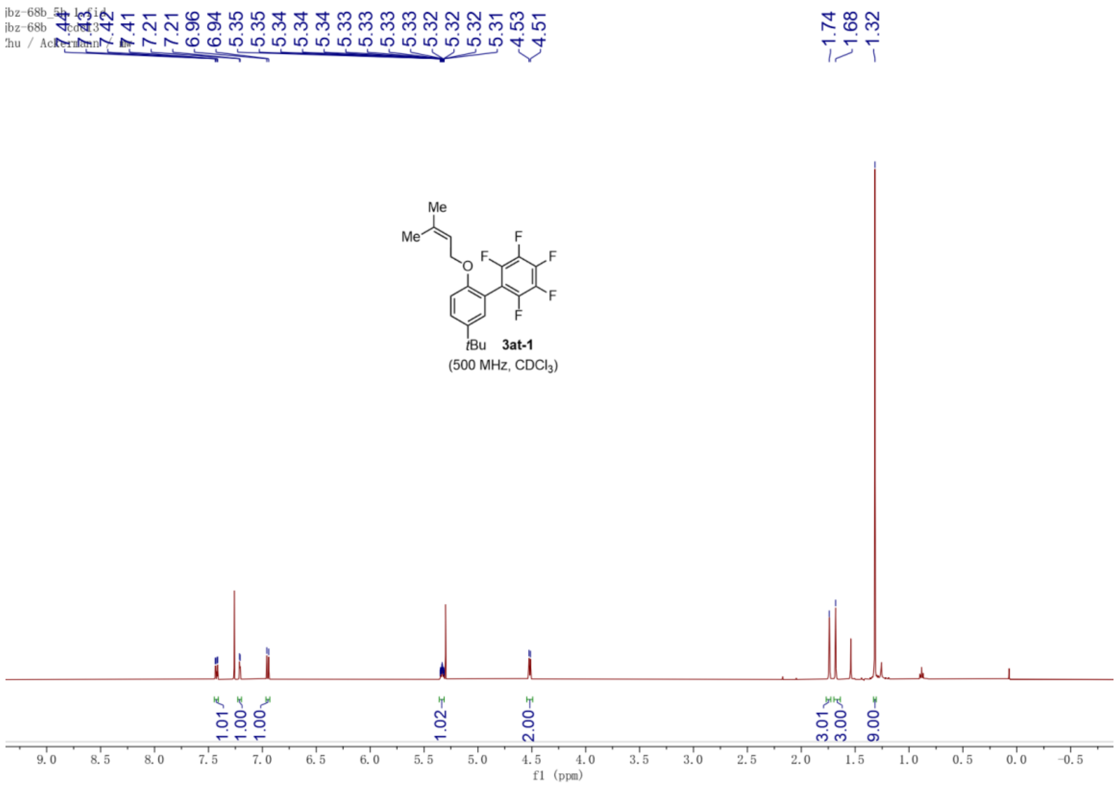
7.43
7.42
7.41
7.21
7.21
6.96
6.94
5.35
5.35
5.34
5.34
5.33
5.33
5.33
5.32
5.32
5.31
4.53
4.51
1.74
1.68
1.32
Me
Me
O
F
F
F
F
F
tBu
3at-1
(500 MHz, CDCl3)
1.01
1.00
1.00
1.02
2.00
3.01
3.00
9.00
f1 (ppm)

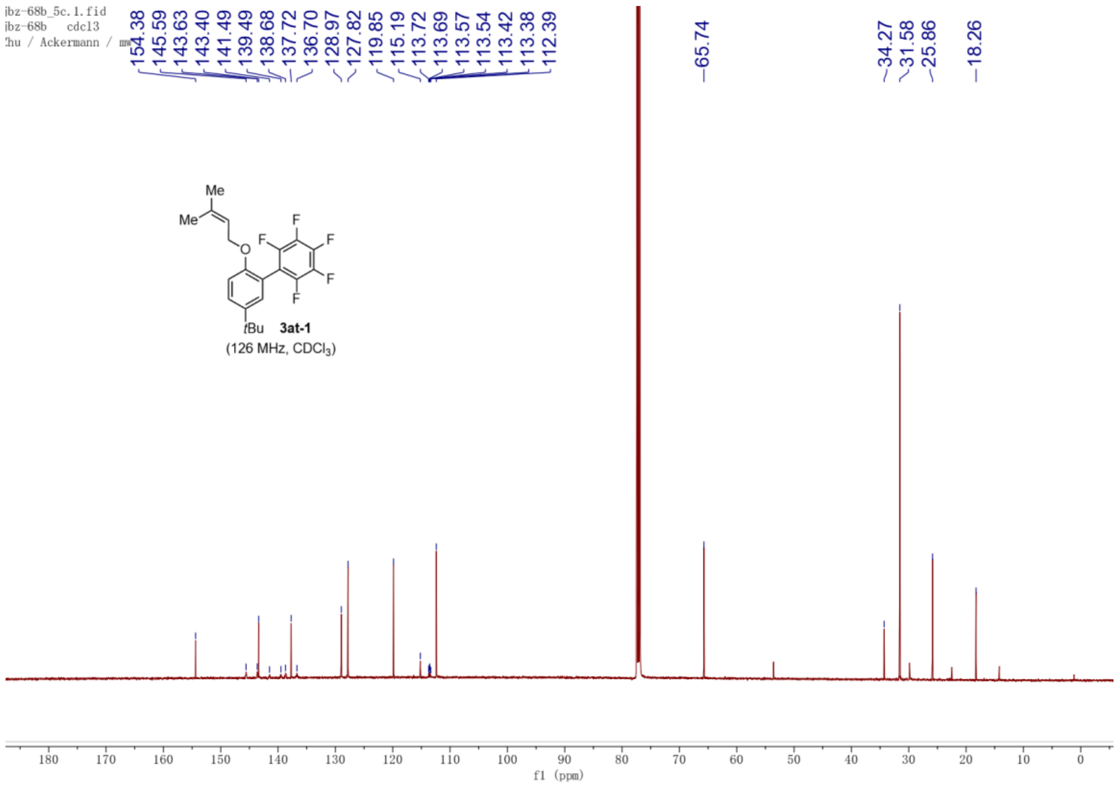
jbz-68b_5c.1.fid
jbz-68b cdcl3
Zhu / Ackermann / mw
154.38 145.59 143.63 143.40 141.49 139.49 138.68 137.72 136.70 128.97 127.82 119.85 115.19 113.72 113.69 113.57 113.54 113.42 113.38 112.39
65.74
34.27 31.58 25.86
18.26
Me
Me
O
F
F
F
F
F
tBu 3at-1
(126 MHz, CDCl3)
180 170 160 150 140 130 120 110 100 90 80 70 60 50 40 30 20 10 0
f1 (ppm)

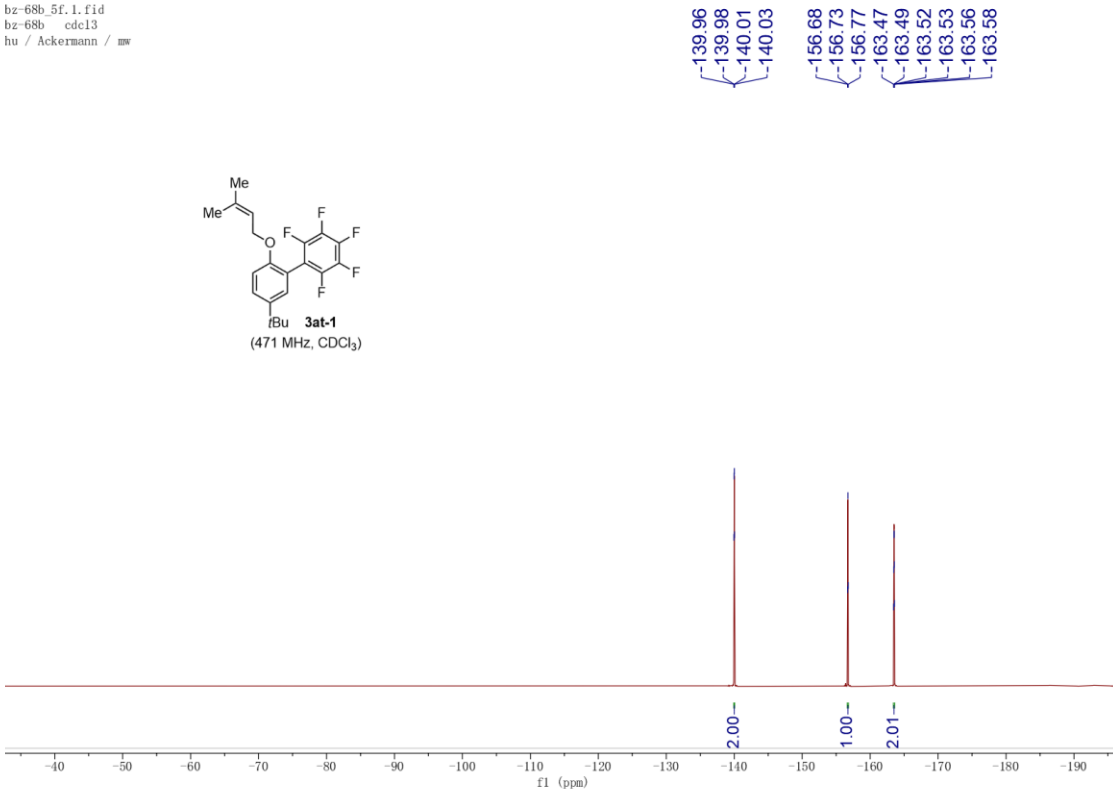
bz-68b_5f.1.fid
bz-68b cdcl3
hu / Ackermann / mw
139.96 139.98 140.01 140.03
156.68 156.73 156.77
163.47 163.49 163.52 163.53 163.56 163.58
Me
Me
O
F
F
F
F
F
tBu 3at-1
(471 MHz, CDCl3)
2.00
1.00
2.01
-40 -50 -60 -70 -80 -90 -100 -110 -120 -130 -140 -150 -160 -170 -180 -190
f1 (ppm)

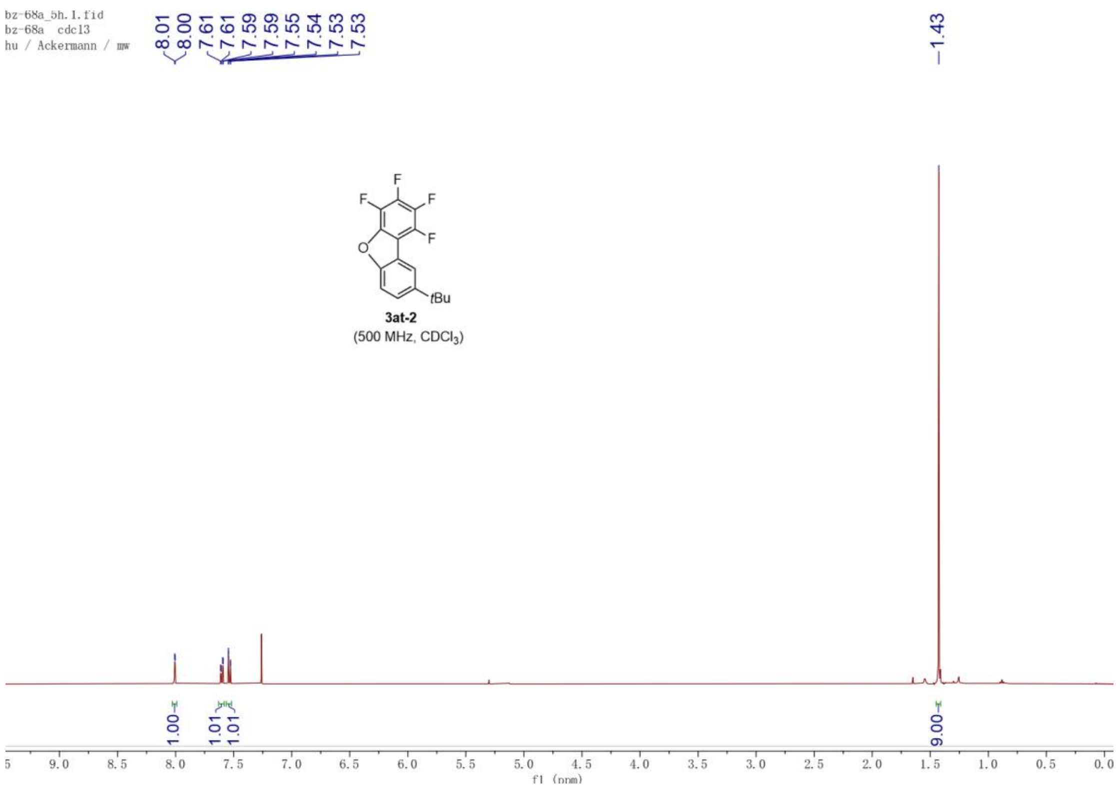
bz-68a_5h.1.fid
bz-68a cdcl3
hu / Ackermann / mw
8.01
8.00
7.61
7.61
7.59
7.59
7.55
7.54
7.53
7.53
1.43
F
F
F
F
O
tBu
3at-2
(500 MHz, CDCl3)
1.00
1.01
1.01
9.00
f1 (ppm)

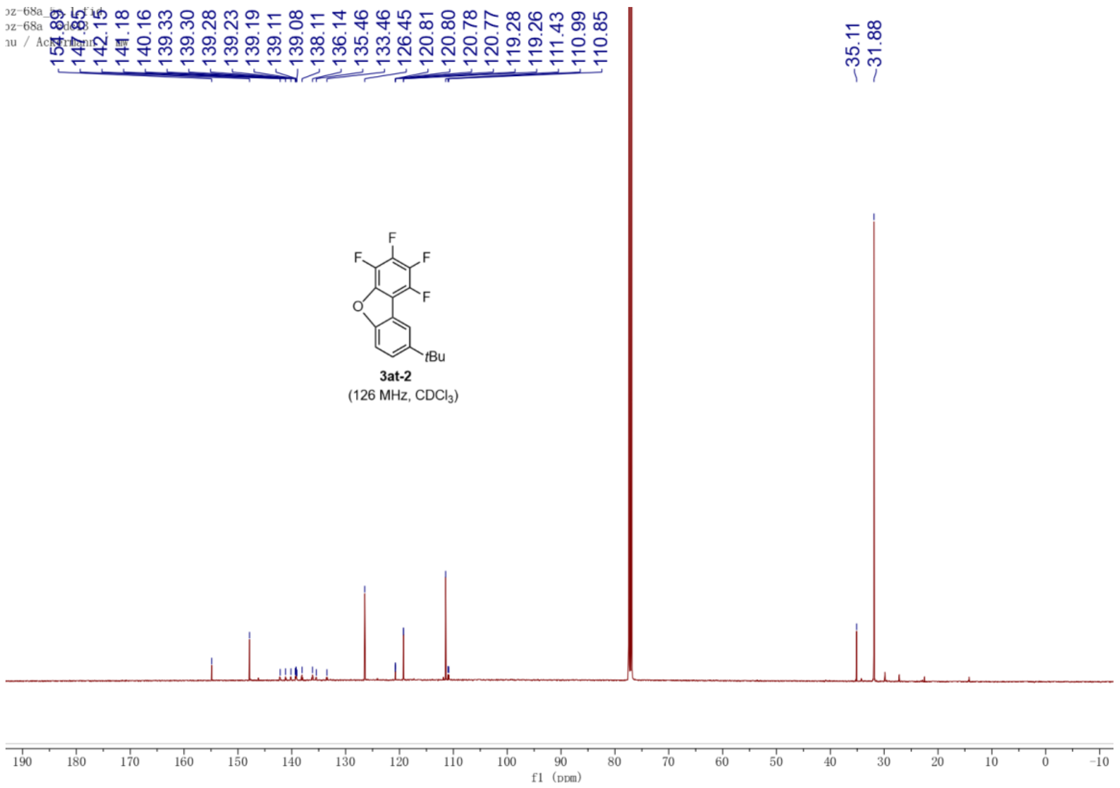
142.15
141.18
140.16
139.33
139.30
139.28
139.23
139.19
139.11
139.08
138.11
136.14
135.46
133.46
126.45
120.81
120.80
120.78
120.77
119.28
119.26
111.43
110.99
110.85
35.11
31.88
F
F
F
F
O
tBu
3at-2
(126 MHz, CDCl3)
f1 (ppm)

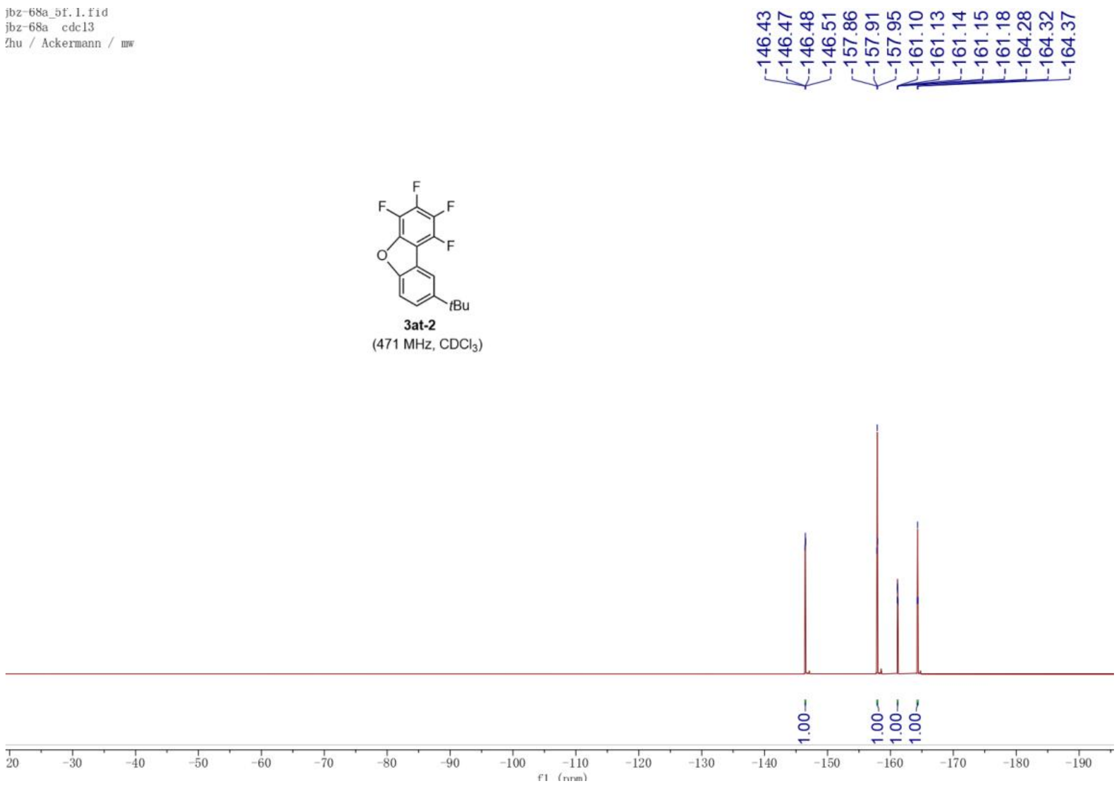

jbz-68a_5f.1.fid
jbz-68a cdcl3
Zhu / Ackermann / mw
-146.43
-146.47
-146.48
-146.51
-157.86
-157.91
-157.95
-161.10
-161.13
-161.14
-161.15
-161.18
-164.28
-164.32
-164.37
F
F
F
F
O
tBu
3at-2
(471 MHz, CDCl3)
1.00
1.00
1.00
1.00
-20
-30
-40
-50
-60
-70
-80
-90
-100
-110
-120
-130
-140
-150
-160
-170
-180
-190
f1 (ppm)


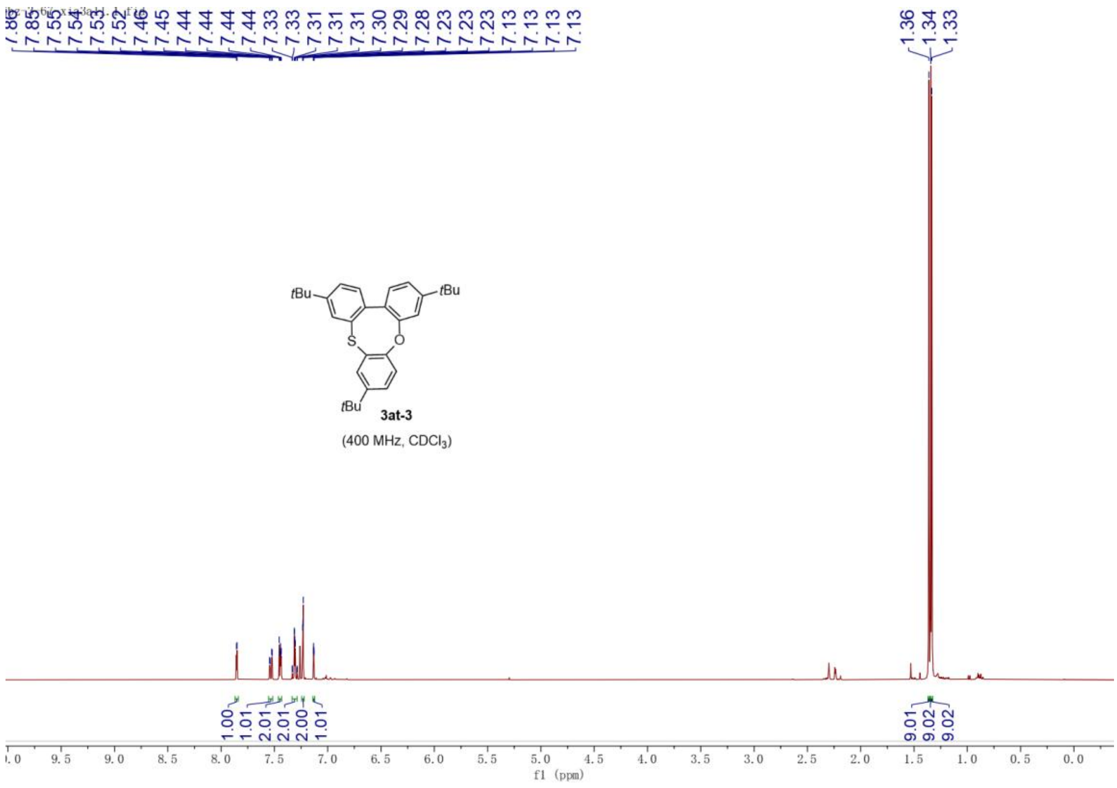

7.86
7.85
7.55
7.54
7.53
7.52
7.46
7.45
7.44
7.44
7.44
7.44
7.33
7.33
7.31
7.31
7.30
7.29
7.28
7.23
7.23
7.23
7.13
7.13
7.13
7.13
1.36
1.34
1.33
tBu
tBu
S
O
tBu
3at-3
(400 MHz, CDCl3)
1.00
1.01
2.01
2.01
2.00
1.01
9.01
9.02
9.02
9.5
9.0
8.5
8.0
7.5
7.0
6.5
6.0
5.5
5.0
4.5
4.0
3.5
3.0
2.5
2.0
1.5
1.0
0.5
0.0
f1 (ppm)

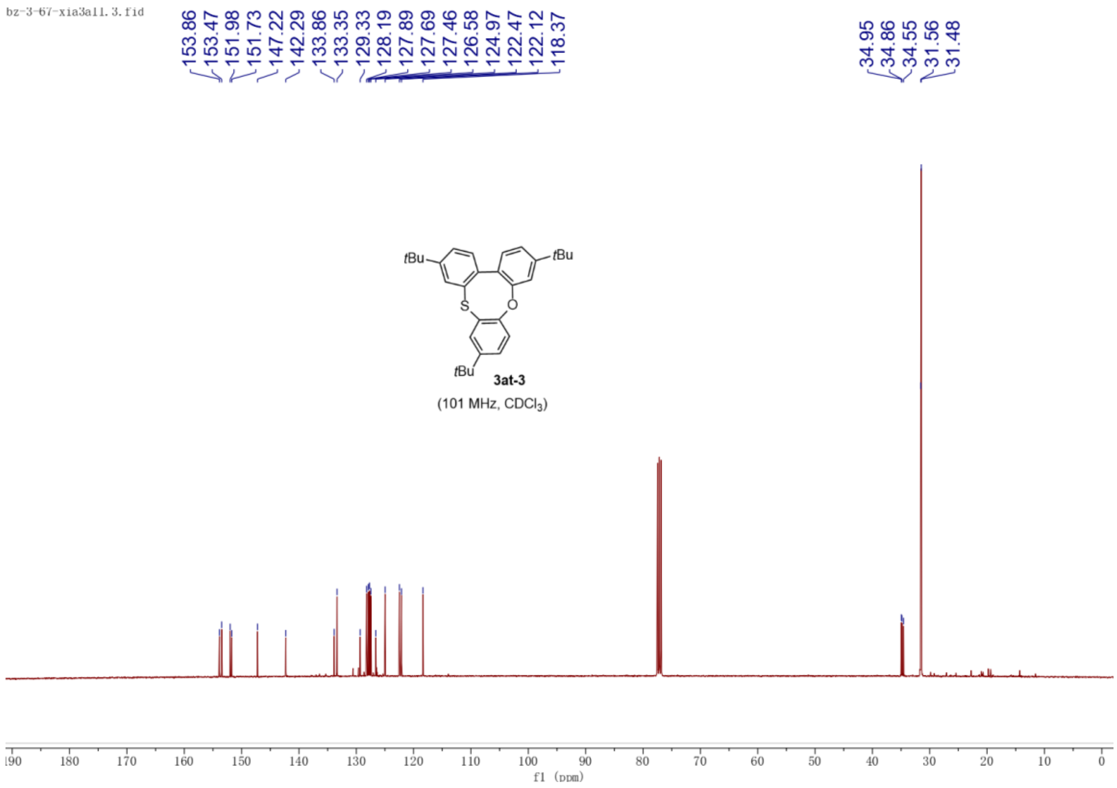

bz-3-67-xia3a11.3.fid
153.86
153.47
151.98
151.73
147.22
142.29
133.86
133.35
129.33
128.19
127.89
127.69
127.46
126.58
124.97
122.47
122.12
118.37
34.95
34.86
34.55
31.56
31.48
tBu
tBu
S
O
tBu
3at-3
(101 MHz, CDCl3)
190
180
170
160
150
140
130
120
110
100
90
80
70
60
50
40
30
20
10
0
f1 (ppm)
