# Supplementary figures and images for: Undirected ruthenium-catalyzed C–H activation using arylsulfonium salts: direct arylation without ruthenacycle intermediates revealed by computation and data science
Source: Chem Sci. 2026 Jan 23;17(12):6138–46. doi: 10.1039/d5sc08962j (PMC12865703; doi:10.1039/d5sc08962j)

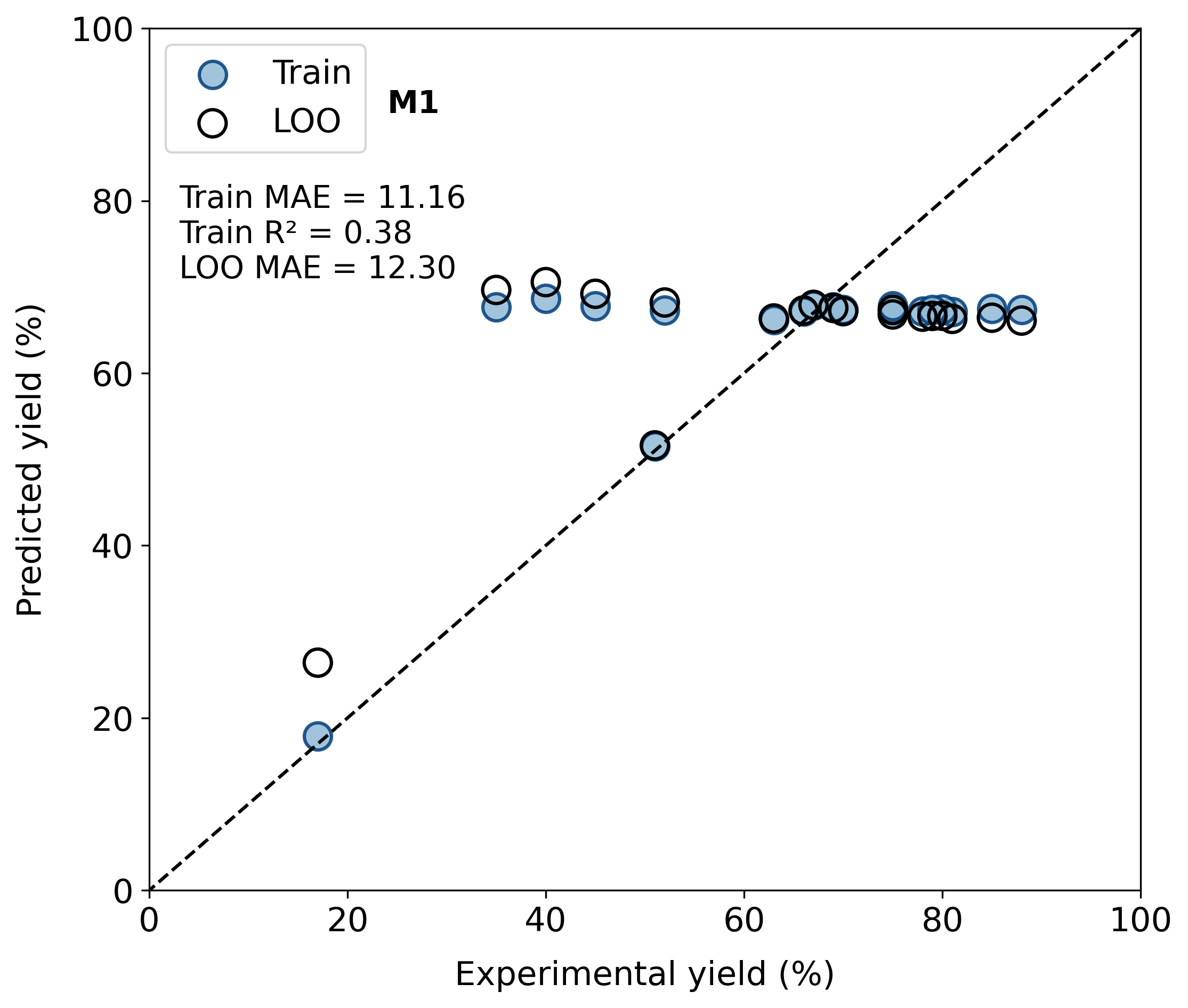

Supplement: SC-017-D5SC08962J-s002 [file SC-017-D5SC08962J-s002.zip › SI_MVLR_Studies/MVLR_Ru_DBT_19samples/M1_model1.png]

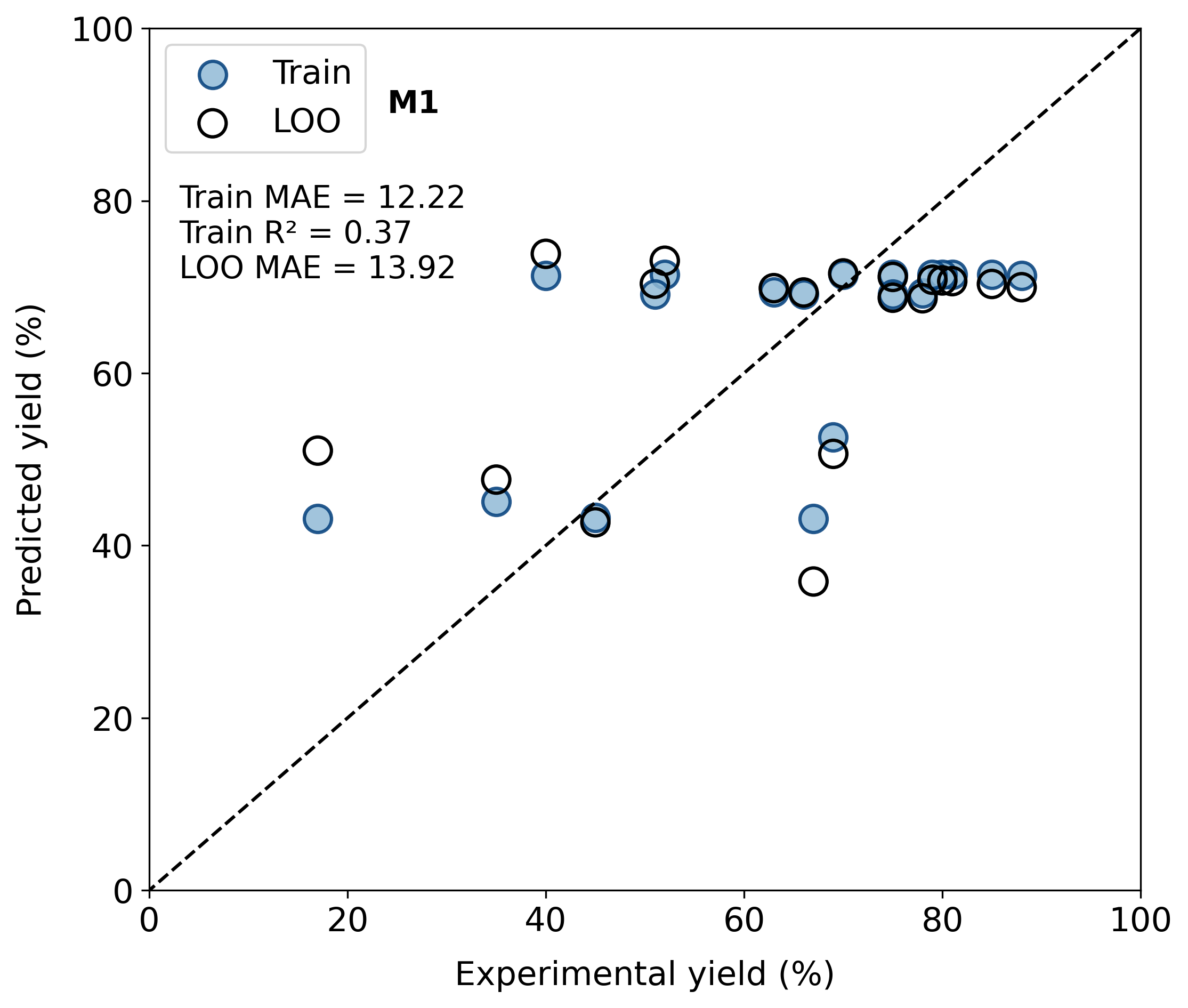

Supplement: SC-017-D5SC08962J-s002 [file SC-017-D5SC08962J-s002.zip › SI_MVLR_Studies/MVLR_Ru_DBT_19samples/M1_model2.png]

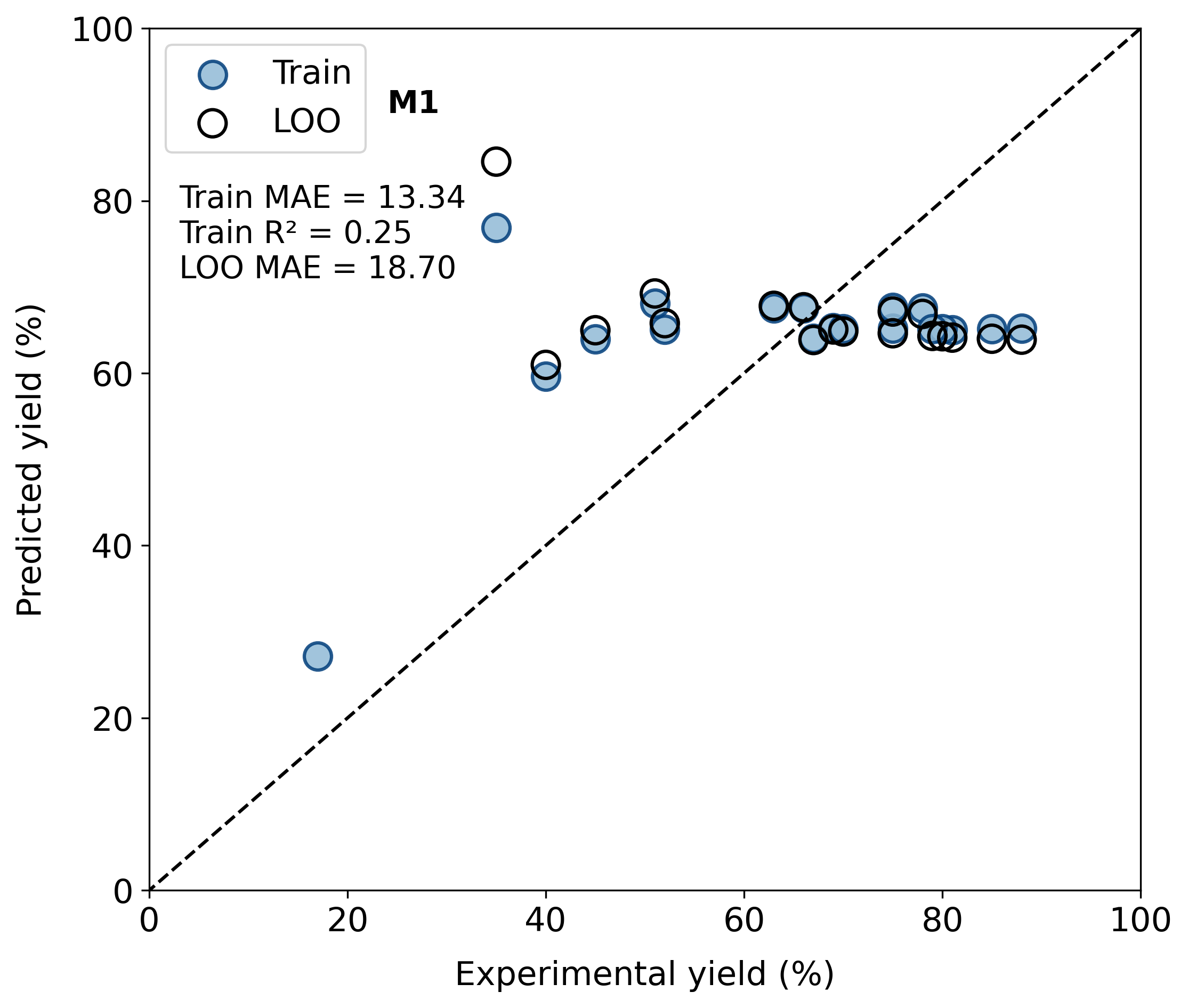

Supplement: SC-017-D5SC08962J-s002 [file SC-017-D5SC08962J-s002.zip › SI_MVLR_Studies/MVLR_Ru_DBT_19samples/M1_model3.png]

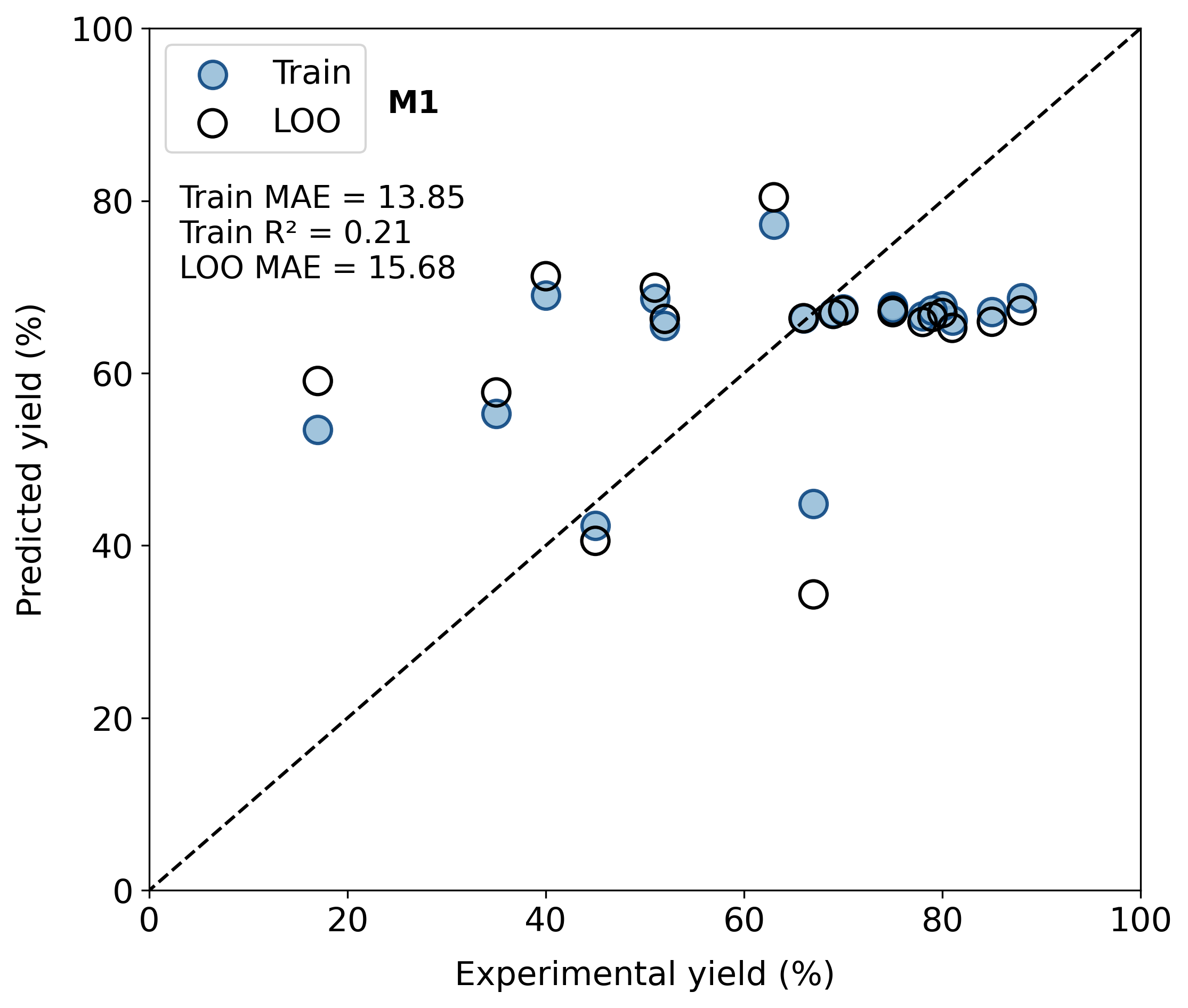

Supplement: SC-017-D5SC08962J-s002 [file SC-017-D5SC08962J-s002.zip › SI_MVLR_Studies/MVLR_Ru_DBT_19samples/M1_model4.png]

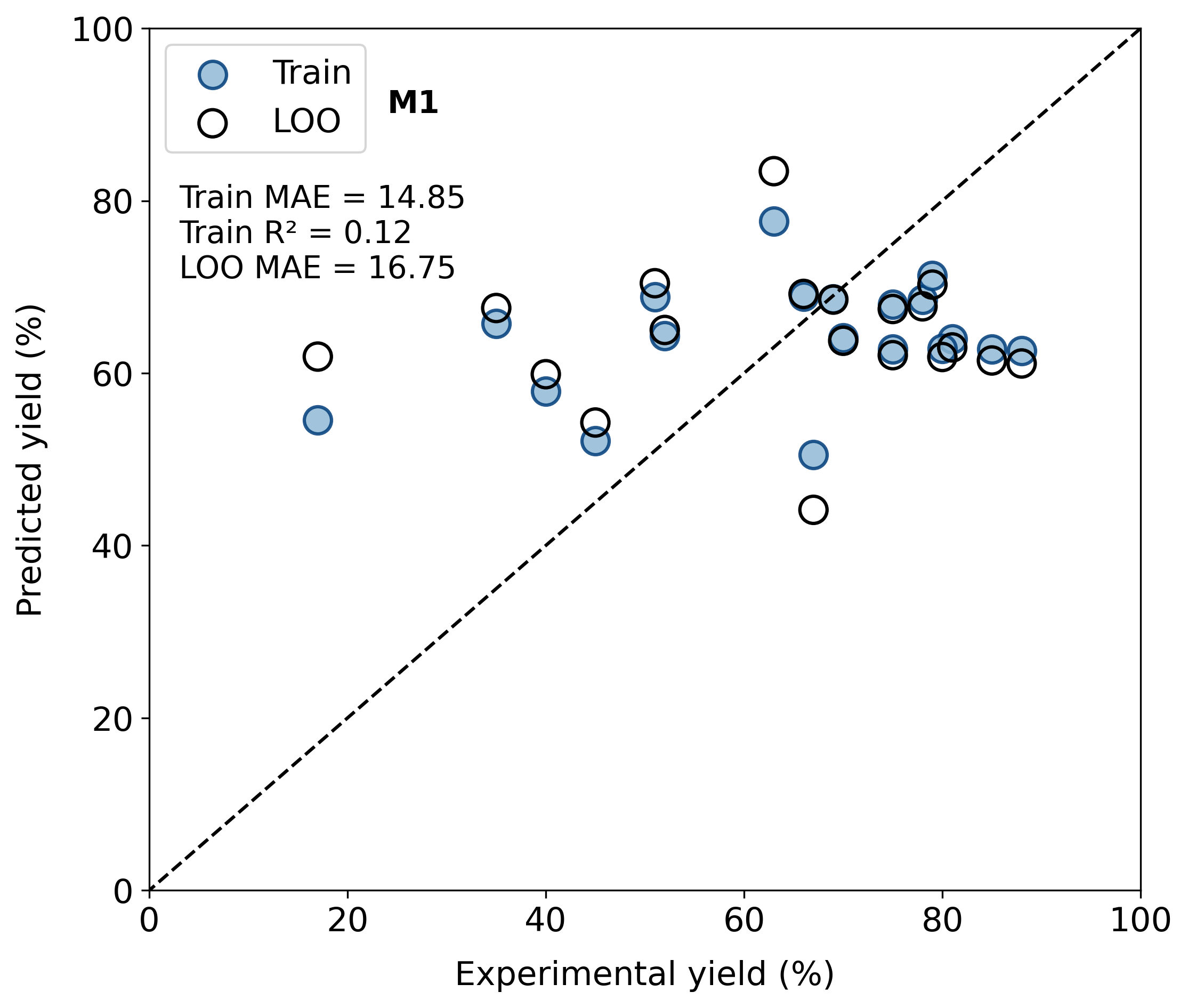

Supplement: SC-017-D5SC08962J-s002 [file SC-017-D5SC08962J-s002.zip › SI_MVLR_Studies/MVLR_Ru_DBT_19samples/M1_model5.png]

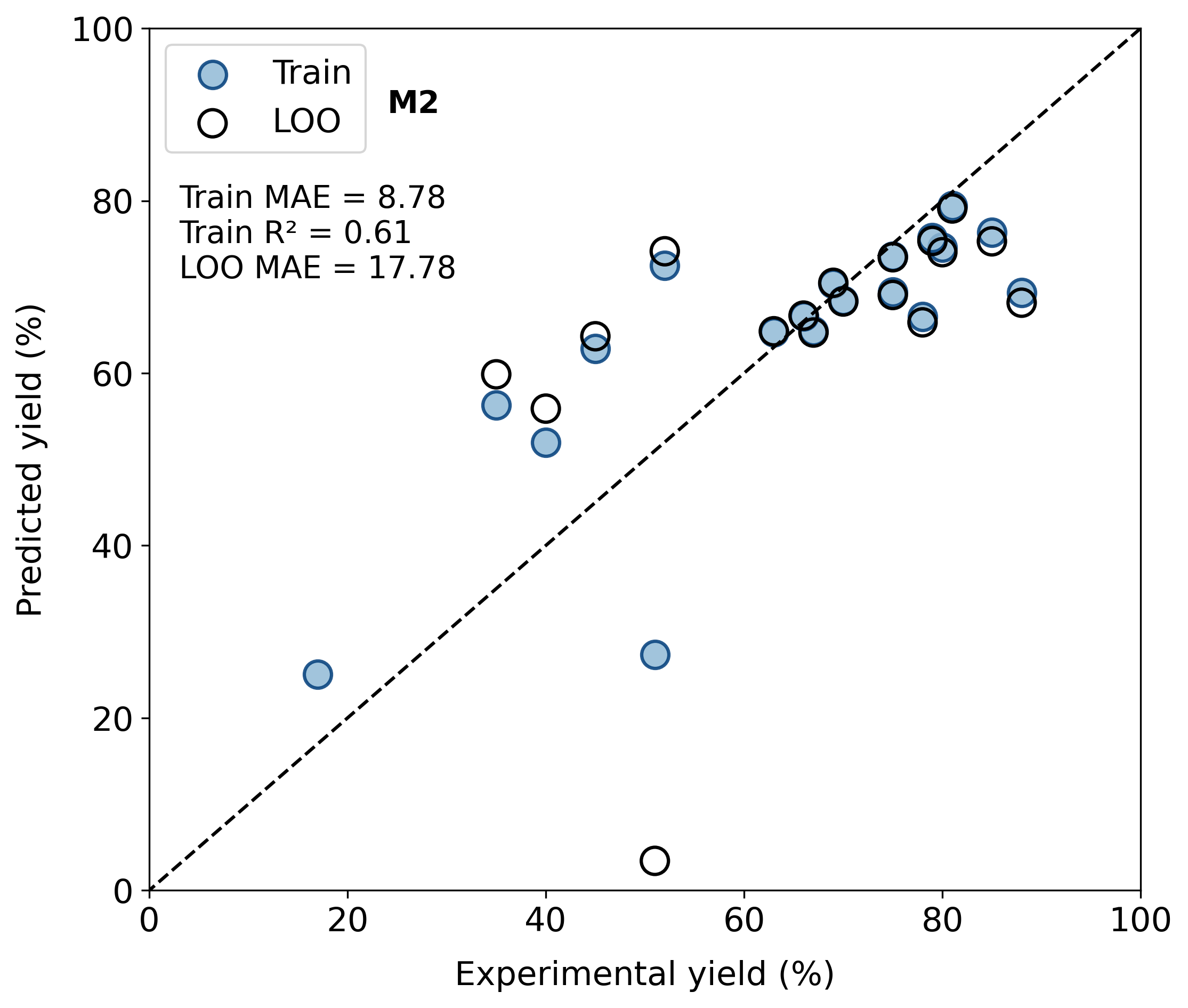

Supplement: SC-017-D5SC08962J-s002 [file SC-017-D5SC08962J-s002.zip › SI_MVLR_Studies/MVLR_Ru_DBT_19samples/M2_model1.png]

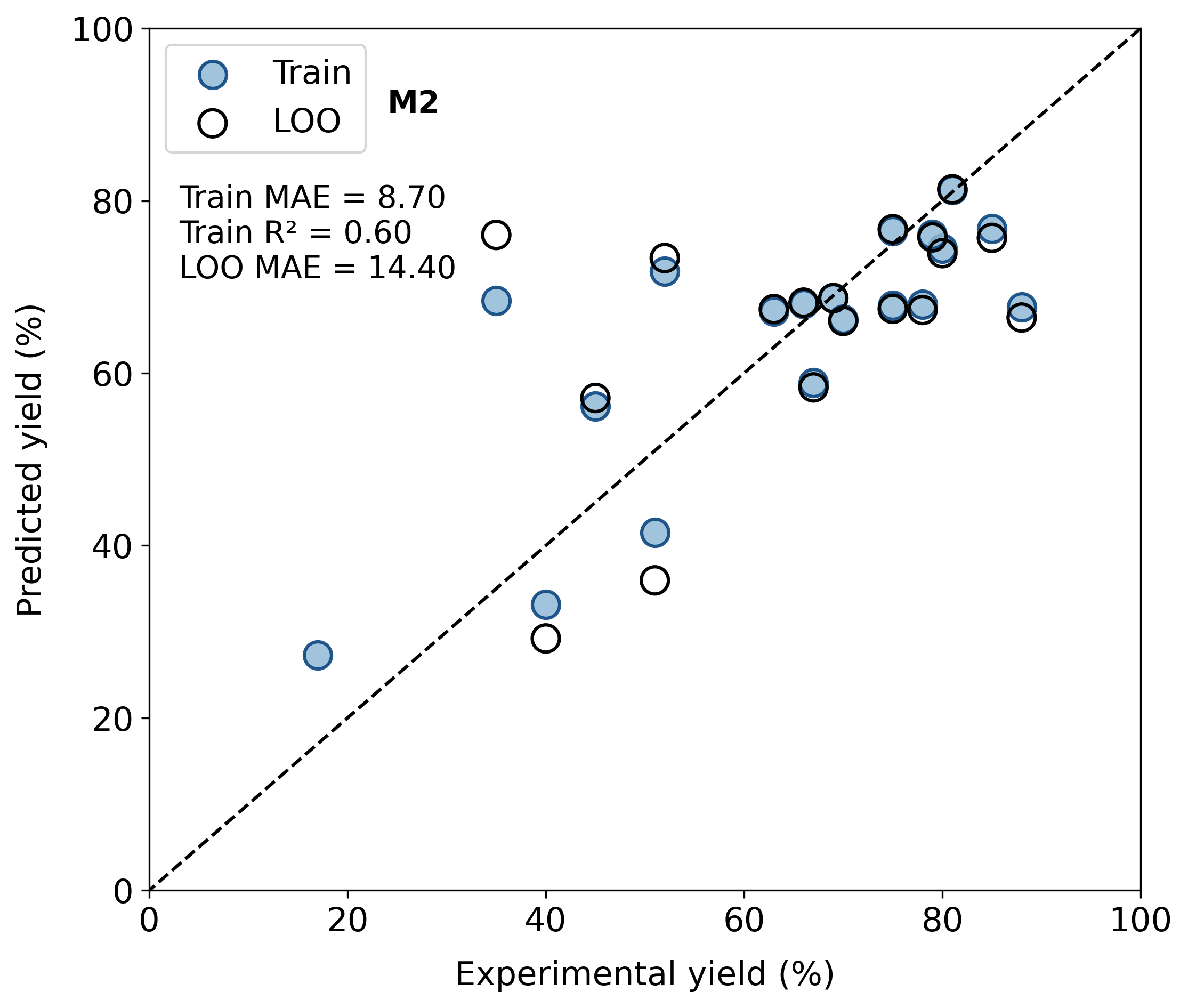

Supplement: SC-017-D5SC08962J-s002 [file SC-017-D5SC08962J-s002.zip › SI_MVLR_Studies/MVLR_Ru_DBT_19samples/M2_model2.png]

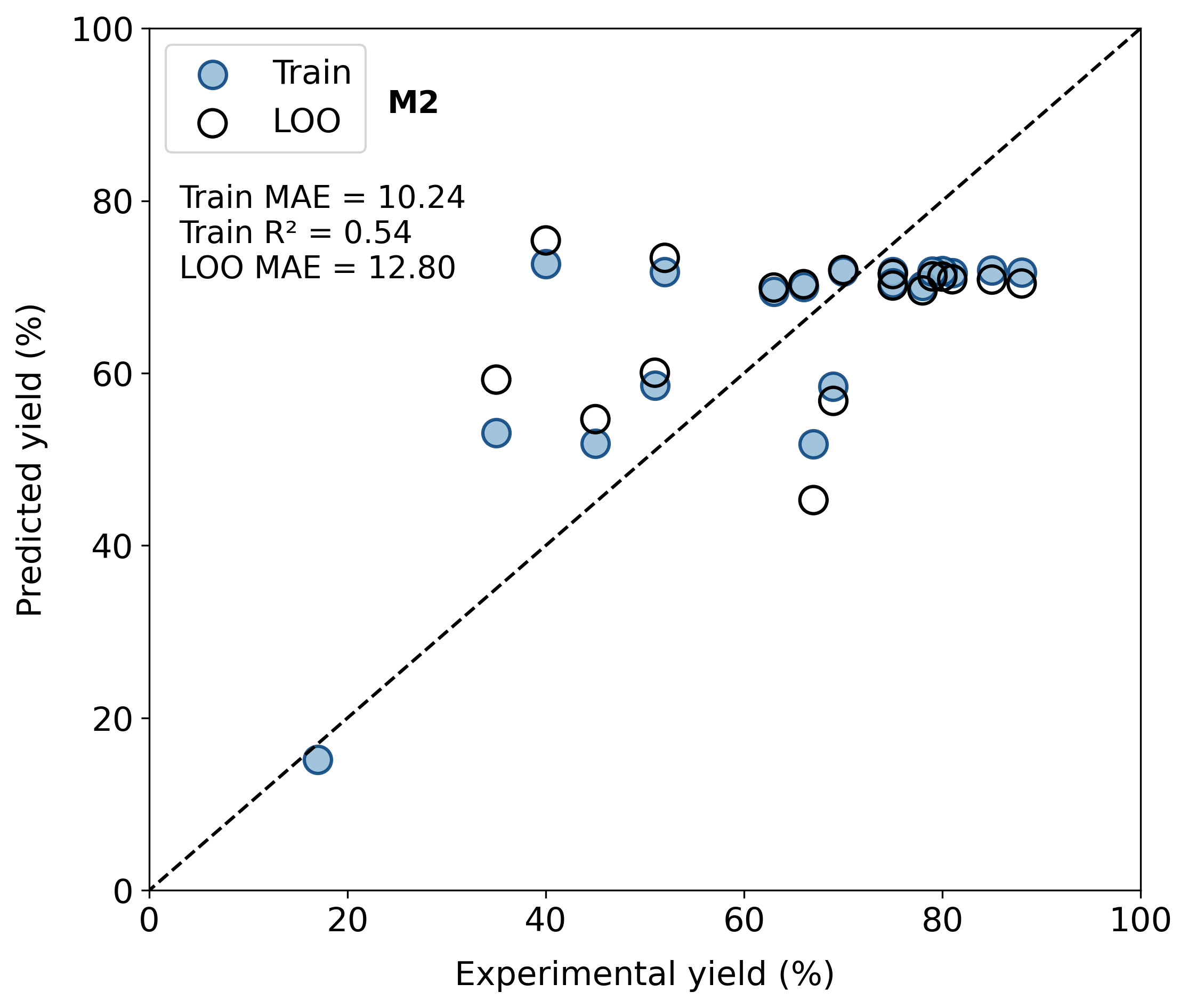

Supplement: SC-017-D5SC08962J-s002 [file SC-017-D5SC08962J-s002.zip › SI_MVLR_Studies/MVLR_Ru_DBT_19samples/M2_model3.png]

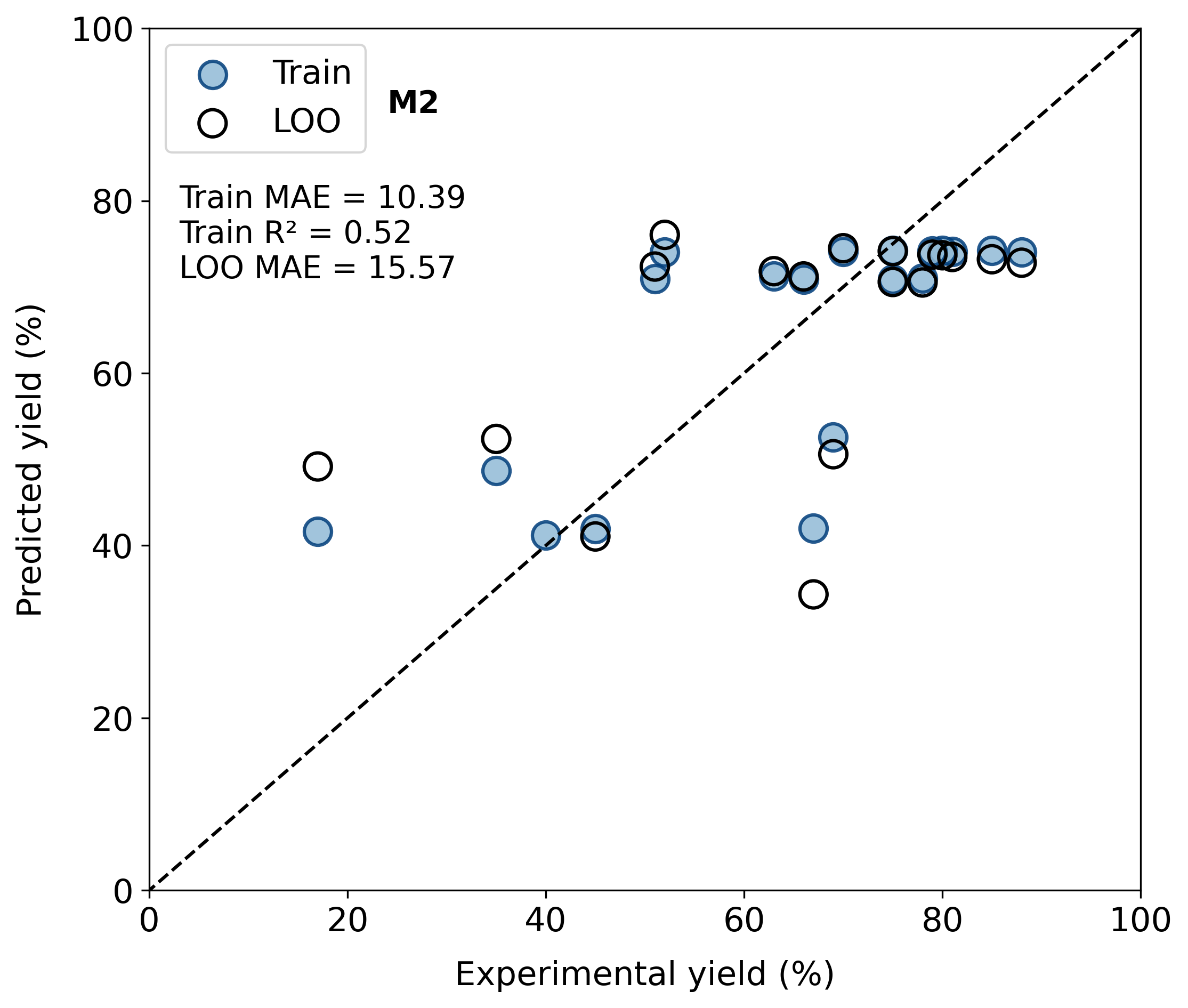

Supplement: SC-017-D5SC08962J-s002 [file SC-017-D5SC08962J-s002.zip › SI_MVLR_Studies/MVLR_Ru_DBT_19samples/M2_model4.png]

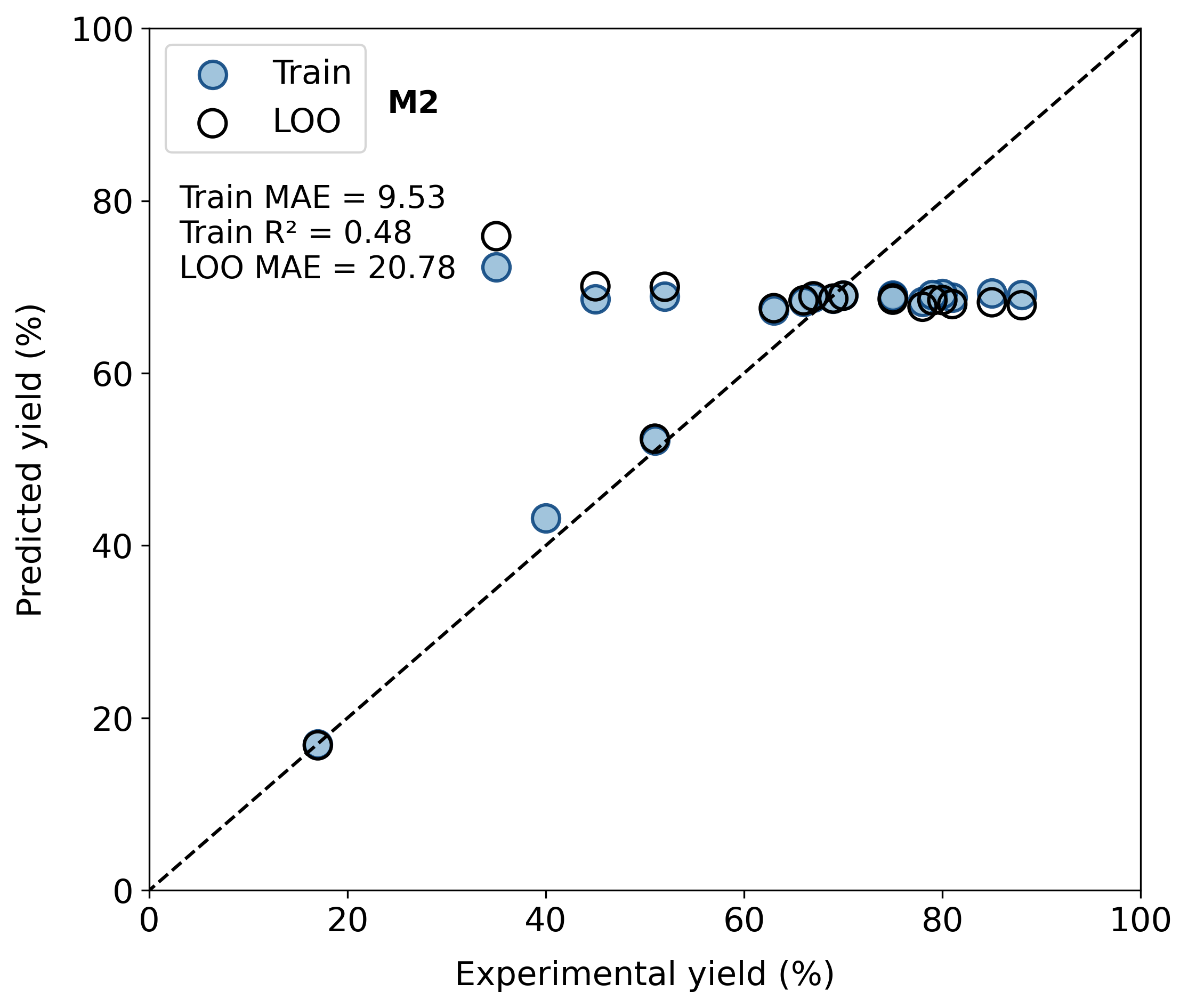

Supplement: SC-017-D5SC08962J-s002 [file SC-017-D5SC08962J-s002.zip › SI_MVLR_Studies/MVLR_Ru_DBT_19samples/M2_model5.png]

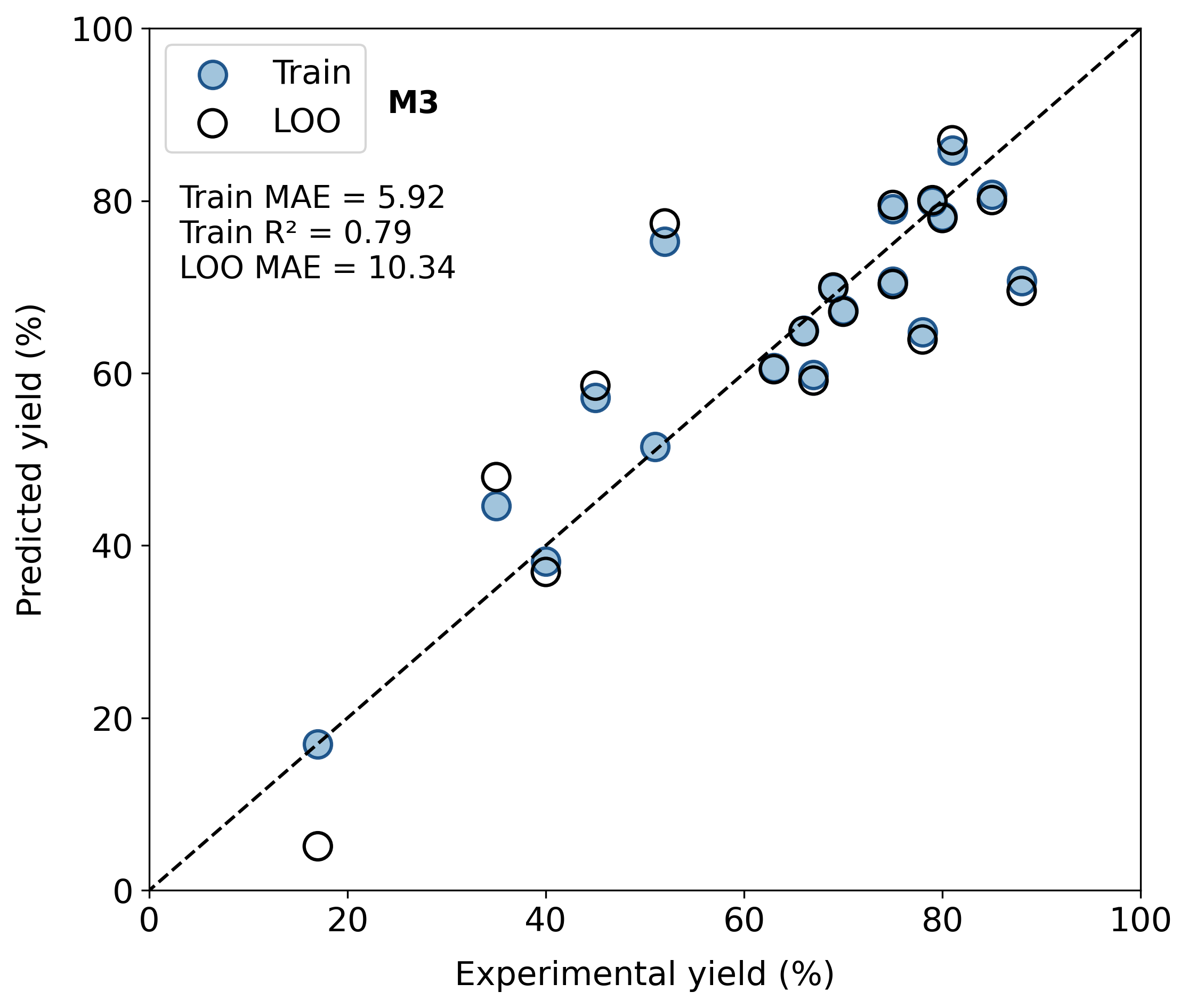

Supplement: SC-017-D5SC08962J-s002 [file SC-017-D5SC08962J-s002.zip › SI_MVLR_Studies/MVLR_Ru_DBT_19samples/M3_model1.png]

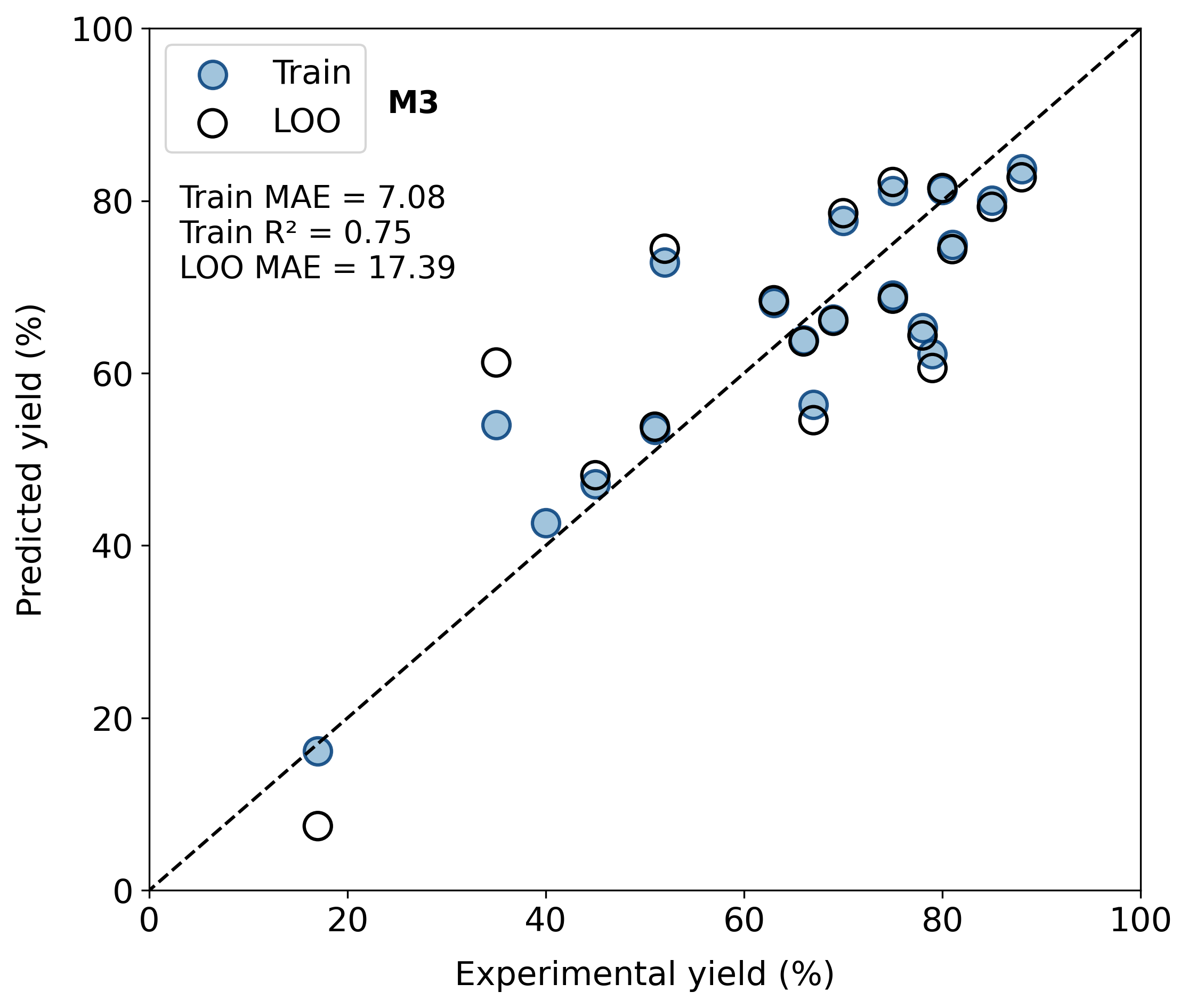

Supplement: SC-017-D5SC08962J-s002 [file SC-017-D5SC08962J-s002.zip › SI_MVLR_Studies/MVLR_Ru_DBT_19samples/M3_model2.png]

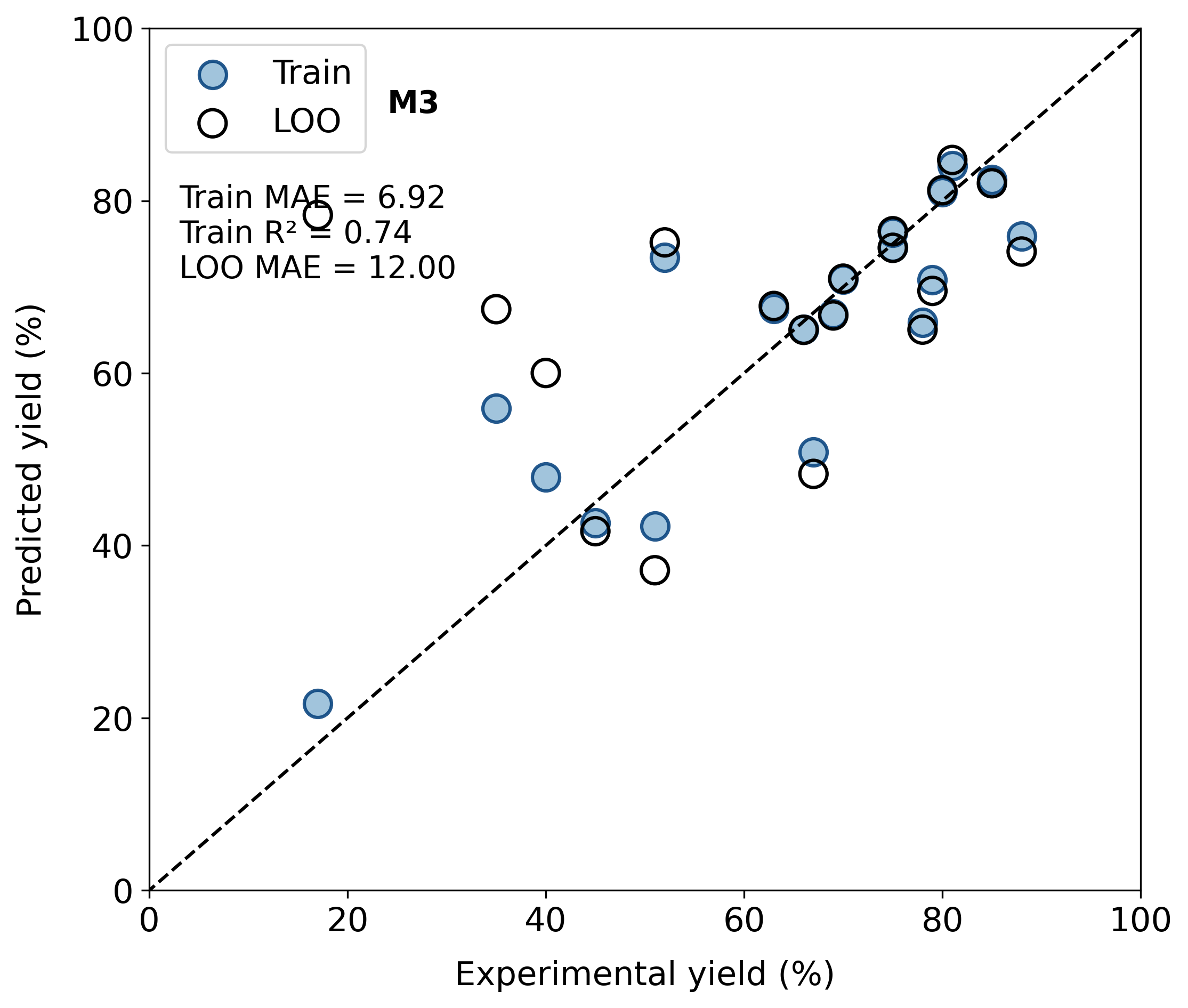

Supplement: SC-017-D5SC08962J-s002 [file SC-017-D5SC08962J-s002.zip › SI_MVLR_Studies/MVLR_Ru_DBT_19samples/M3_model3.png]

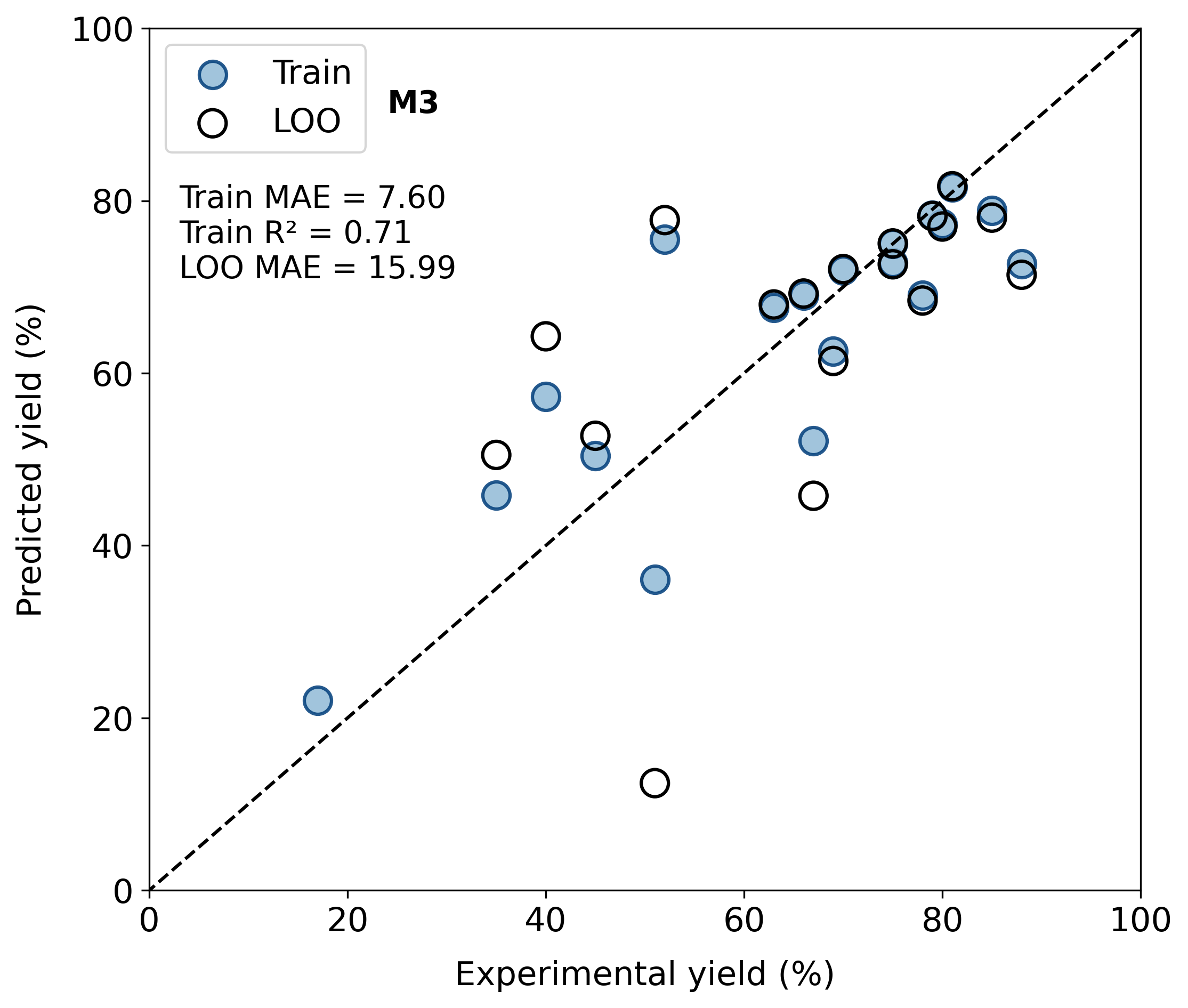

Supplement: SC-017-D5SC08962J-s002 [file SC-017-D5SC08962J-s002.zip › SI_MVLR_Studies/MVLR_Ru_DBT_19samples/M3_model4.png]

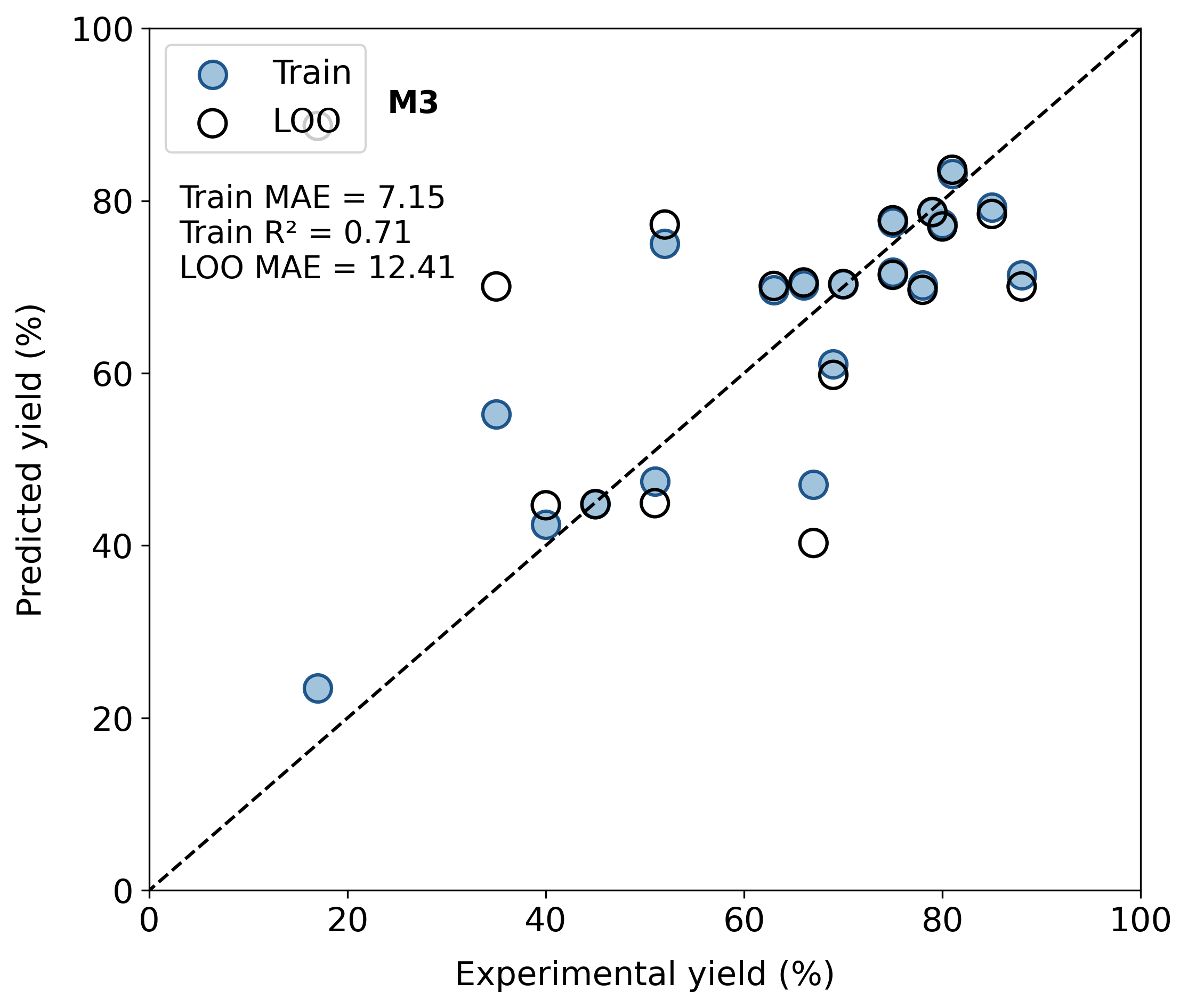

Supplement: SC-017-D5SC08962J-s002 [file SC-017-D5SC08962J-s002.zip › SI_MVLR_Studies/MVLR_Ru_DBT_19samples/M3_model5.png]

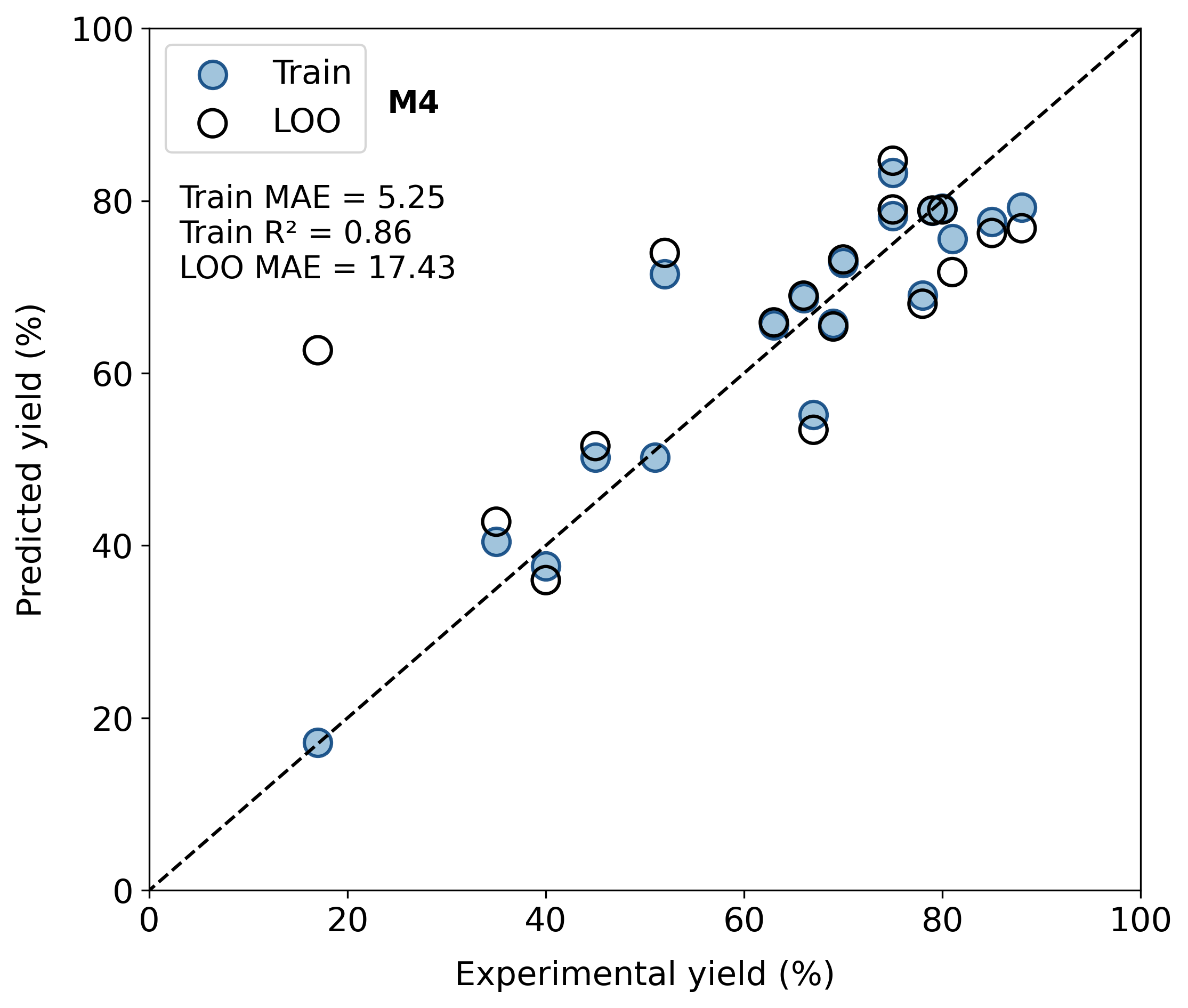

Supplement: SC-017-D5SC08962J-s002 [file SC-017-D5SC08962J-s002.zip › SI_MVLR_Studies/MVLR_Ru_DBT_19samples/M4_model1.png]

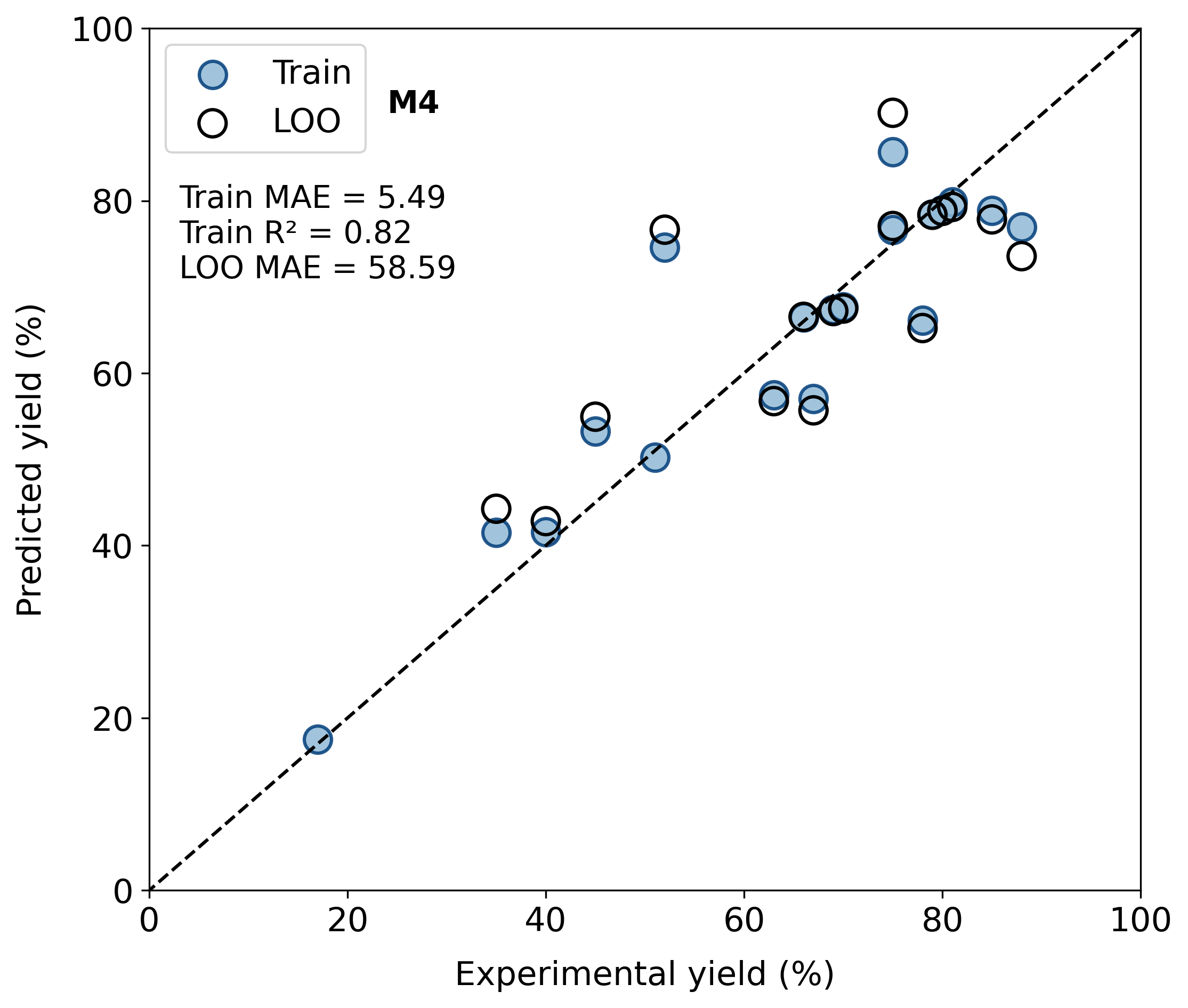

Supplement: SC-017-D5SC08962J-s002 [file SC-017-D5SC08962J-s002.zip › SI_MVLR_Studies/MVLR_Ru_DBT_19samples/M4_model2.png]

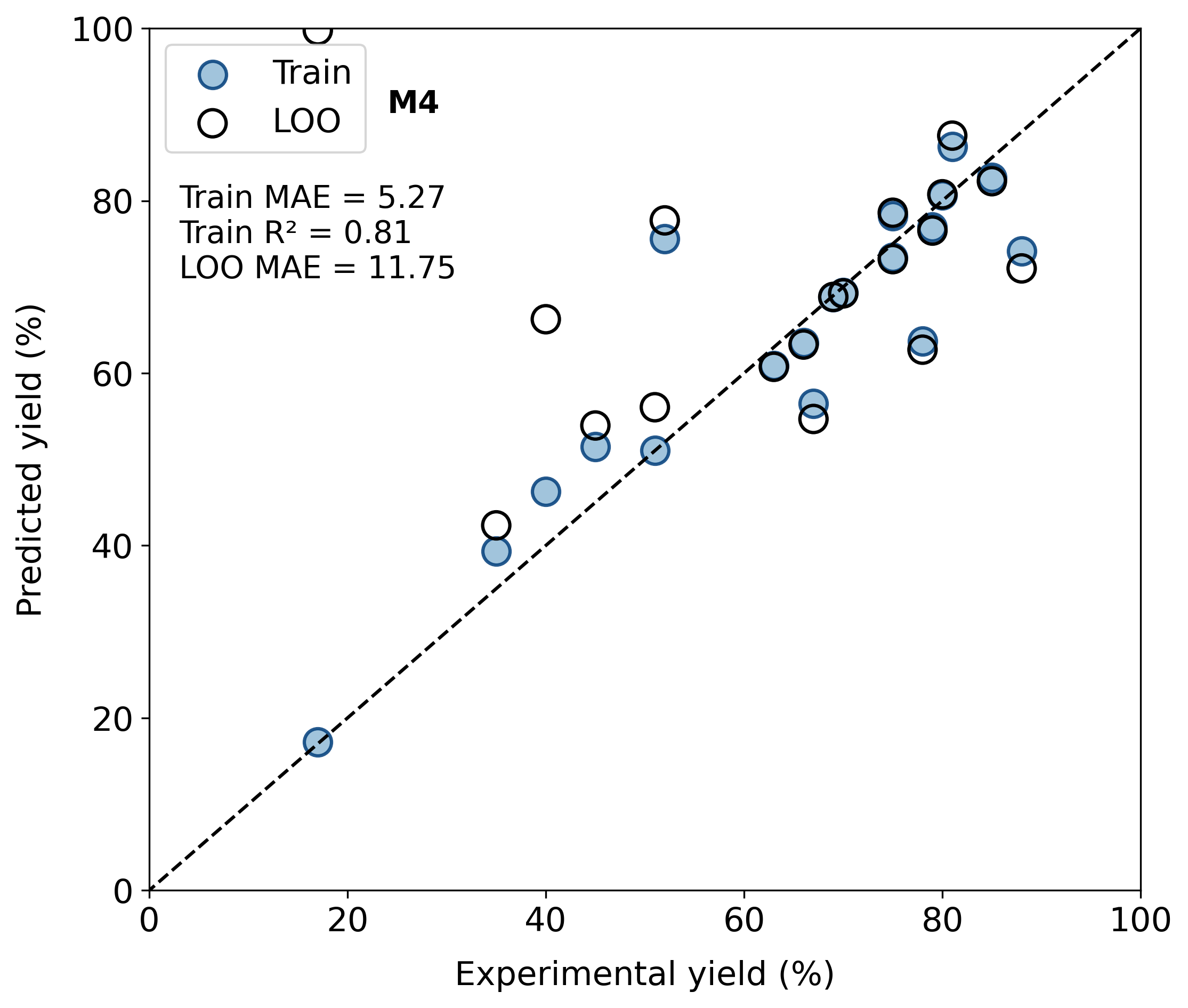

Supplement: SC-017-D5SC08962J-s002 [file SC-017-D5SC08962J-s002.zip › SI_MVLR_Studies/MVLR_Ru_DBT_19samples/M4_model3.png]

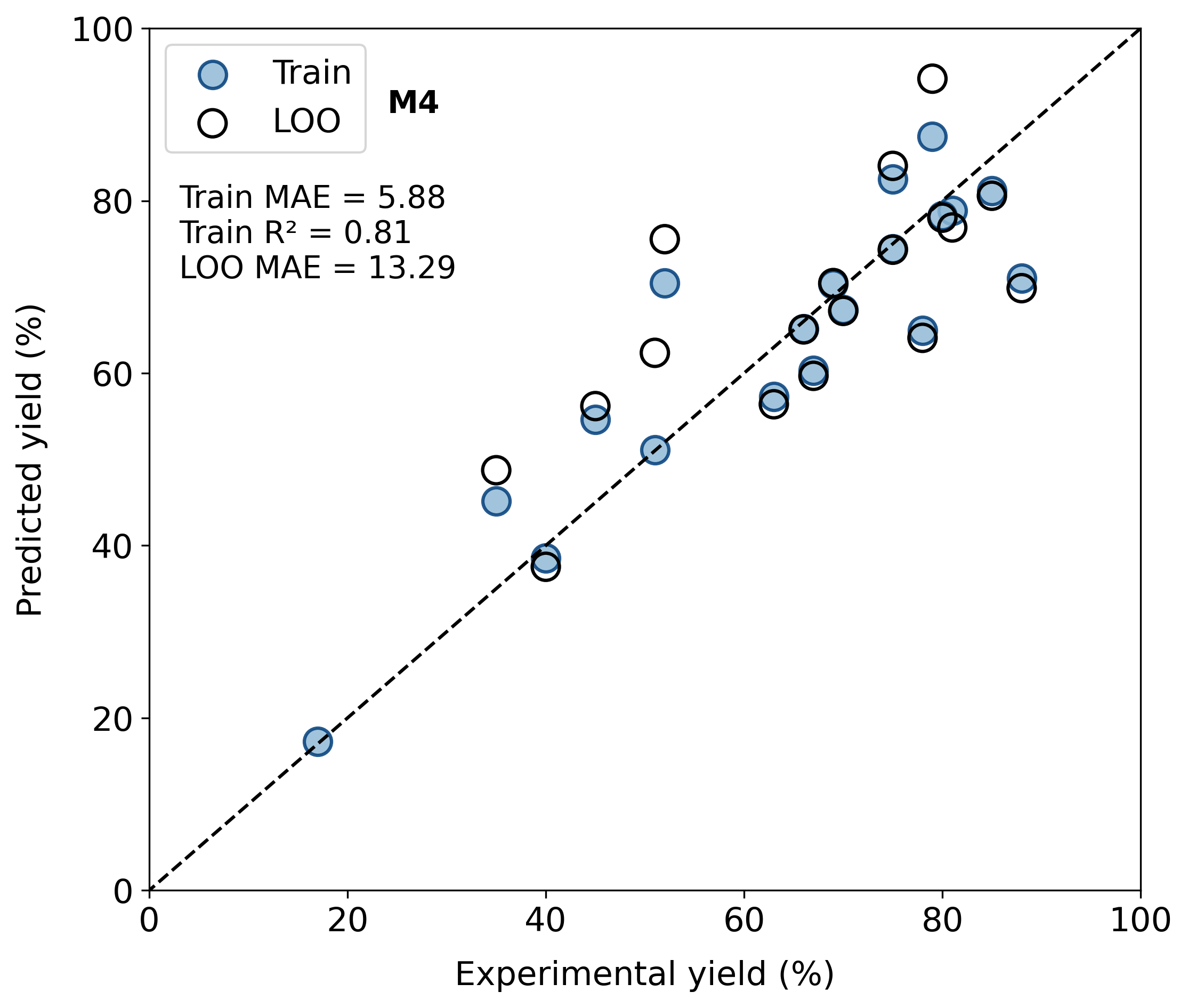

Supplement: SC-017-D5SC08962J-s002 [file SC-017-D5SC08962J-s002.zip › SI_MVLR_Studies/MVLR_Ru_DBT_19samples/M4_model4.png]

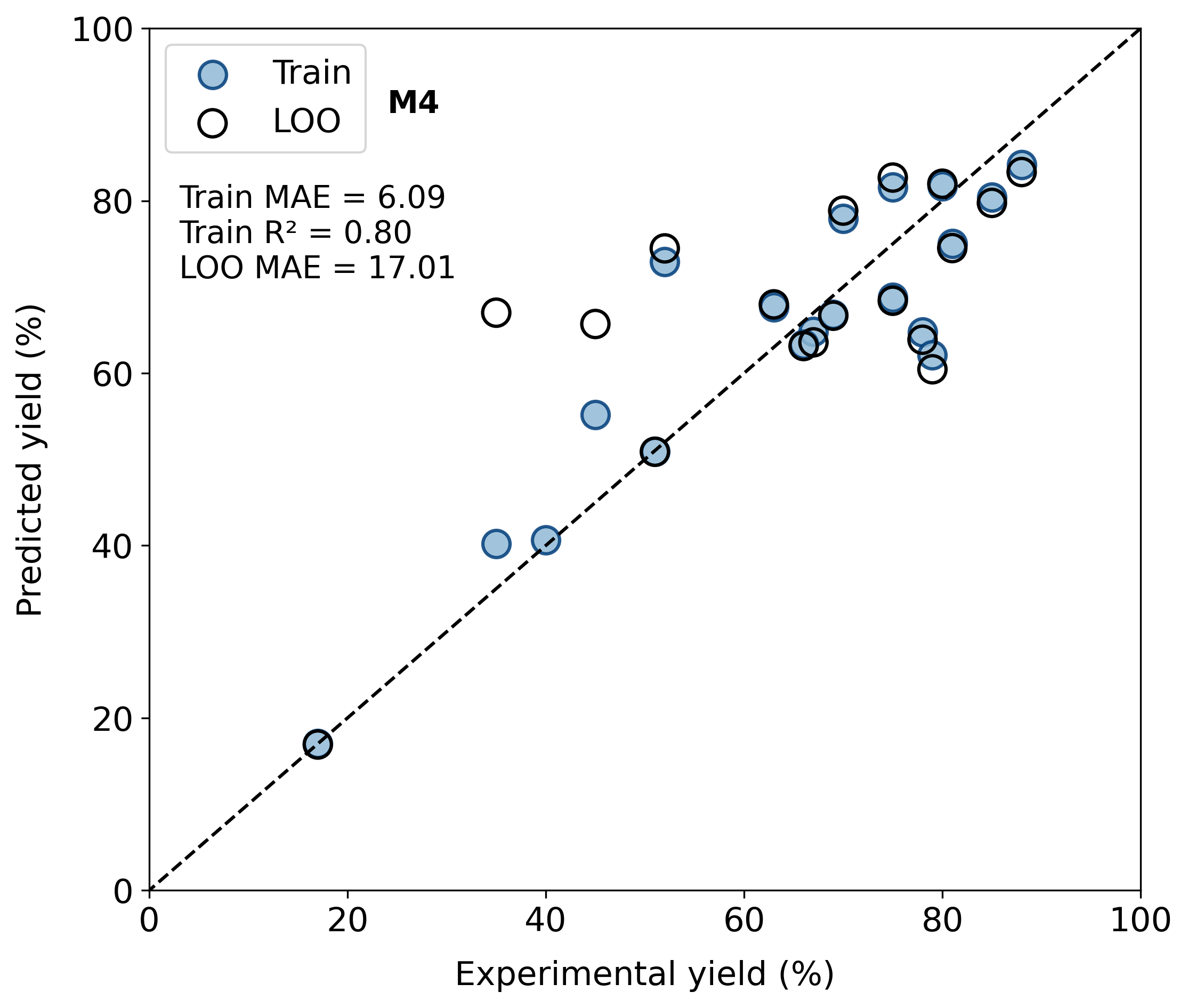

Supplement: SC-017-D5SC08962J-s002 [file SC-017-D5SC08962J-s002.zip › SI_MVLR_Studies/MVLR_Ru_DBT_19samples/M4_model5.png]

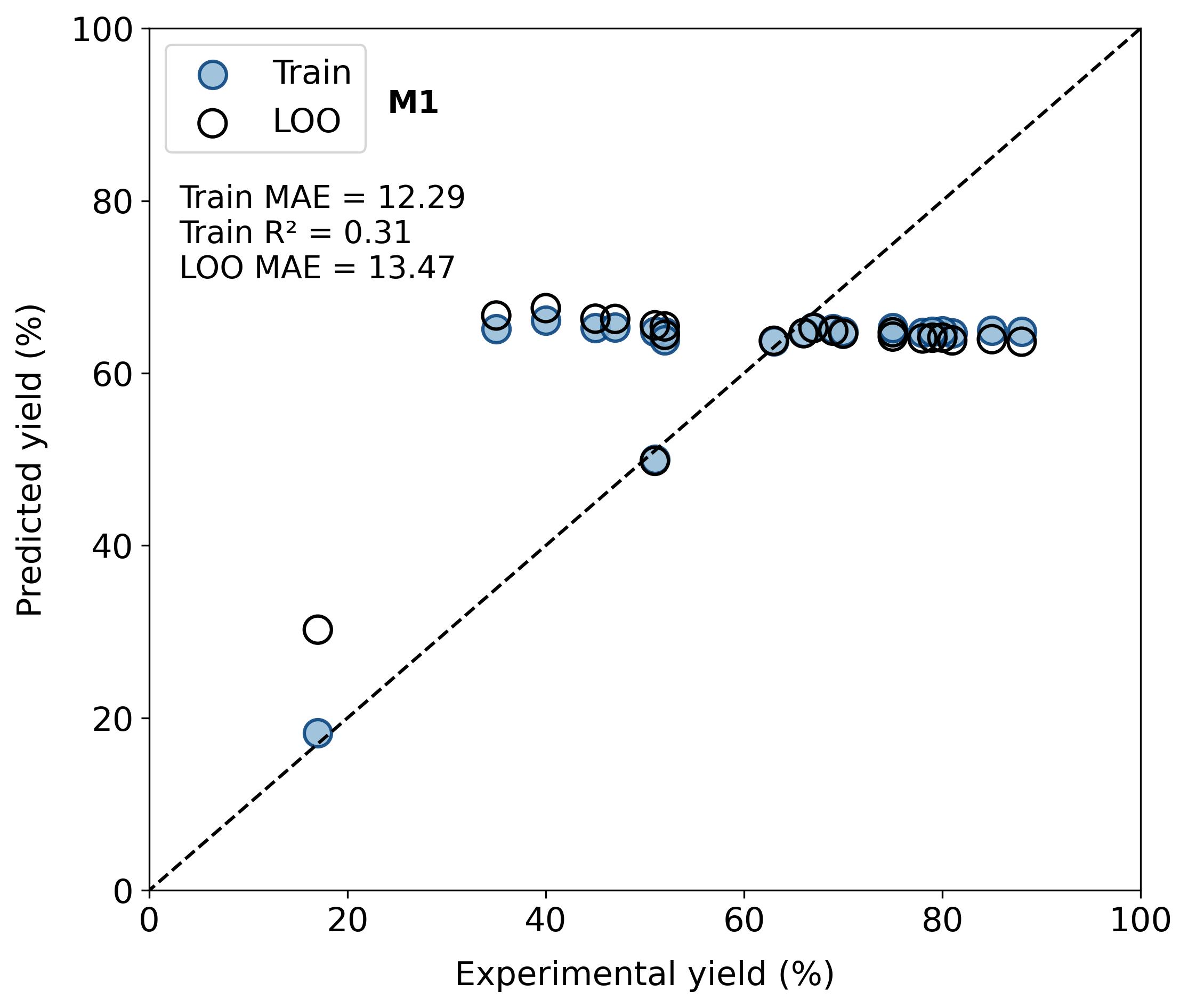

Supplement: SC-017-D5SC08962J-s002 [file SC-017-D5SC08962J-s002.zip › SI_MVLR_Studies/MVLR_Ru_DBT_22samples/M1_model1.png]

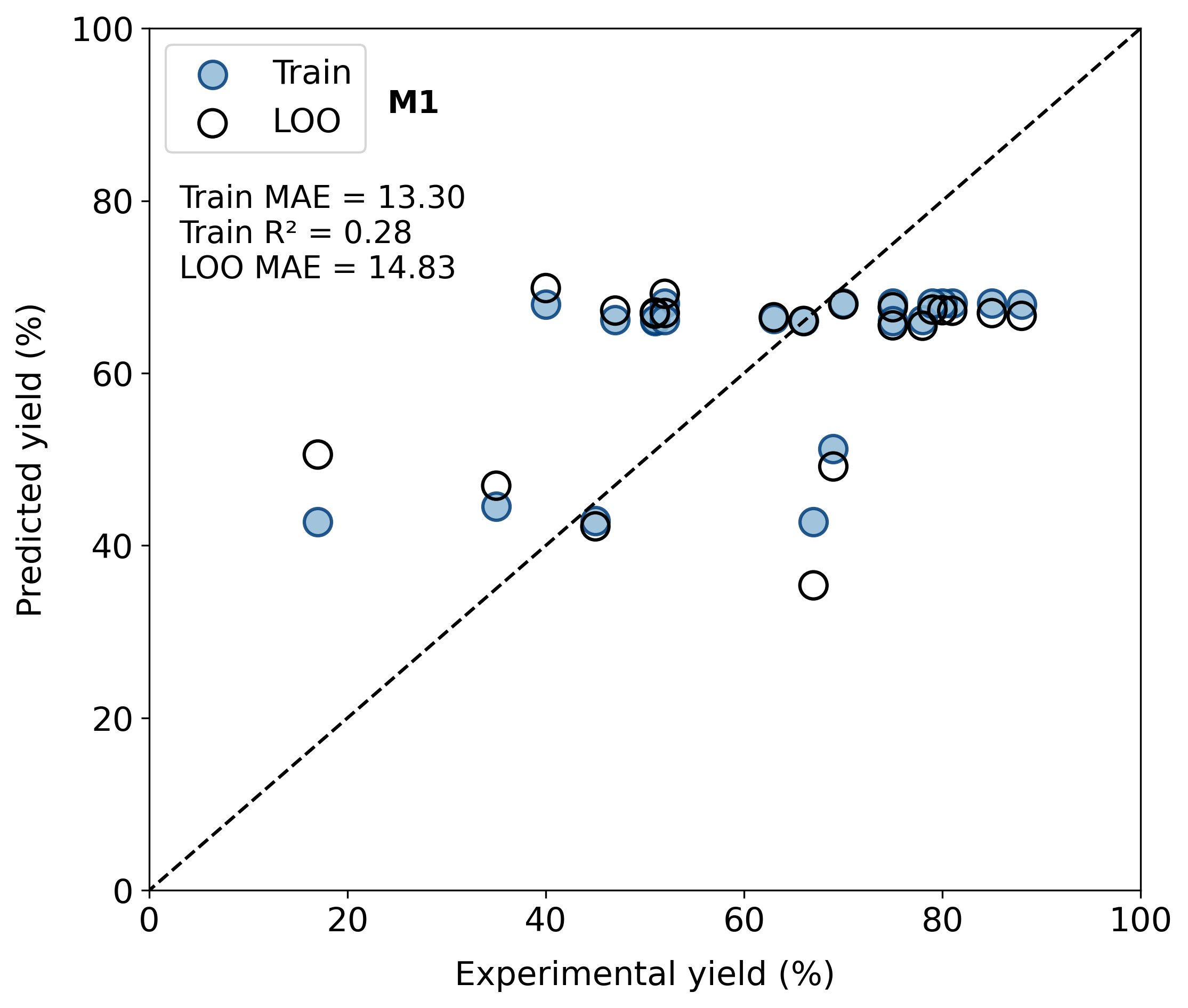

Supplement: SC-017-D5SC08962J-s002 [file SC-017-D5SC08962J-s002.zip › SI_MVLR_Studies/MVLR_Ru_DBT_22samples/M1_model2.png]

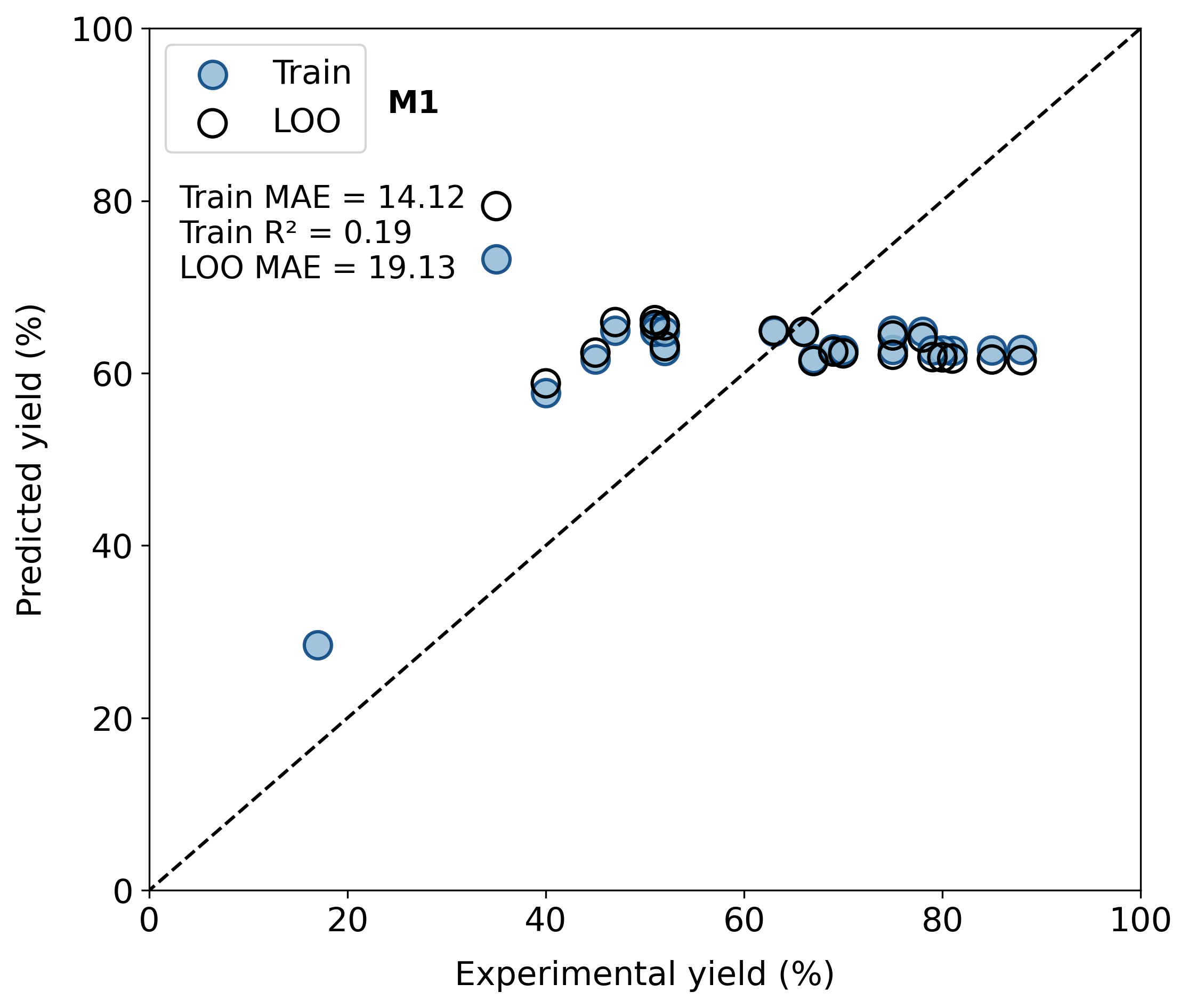

Supplement: SC-017-D5SC08962J-s002 [file SC-017-D5SC08962J-s002.zip › SI_MVLR_Studies/MVLR_Ru_DBT_22samples/M1_model3.png]

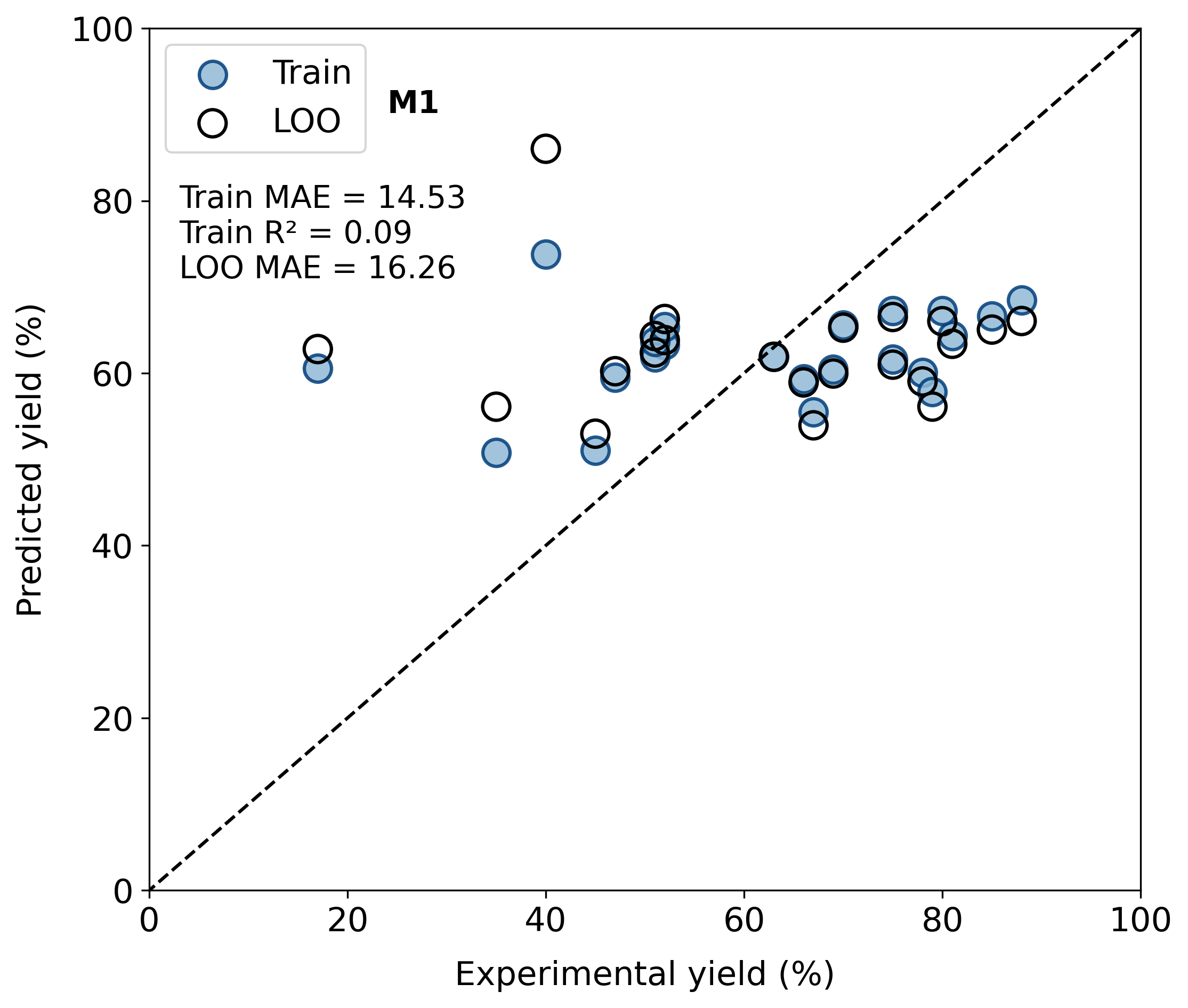

Supplement: SC-017-D5SC08962J-s002 [file SC-017-D5SC08962J-s002.zip › SI_MVLR_Studies/MVLR_Ru_DBT_22samples/M1_model4.png]

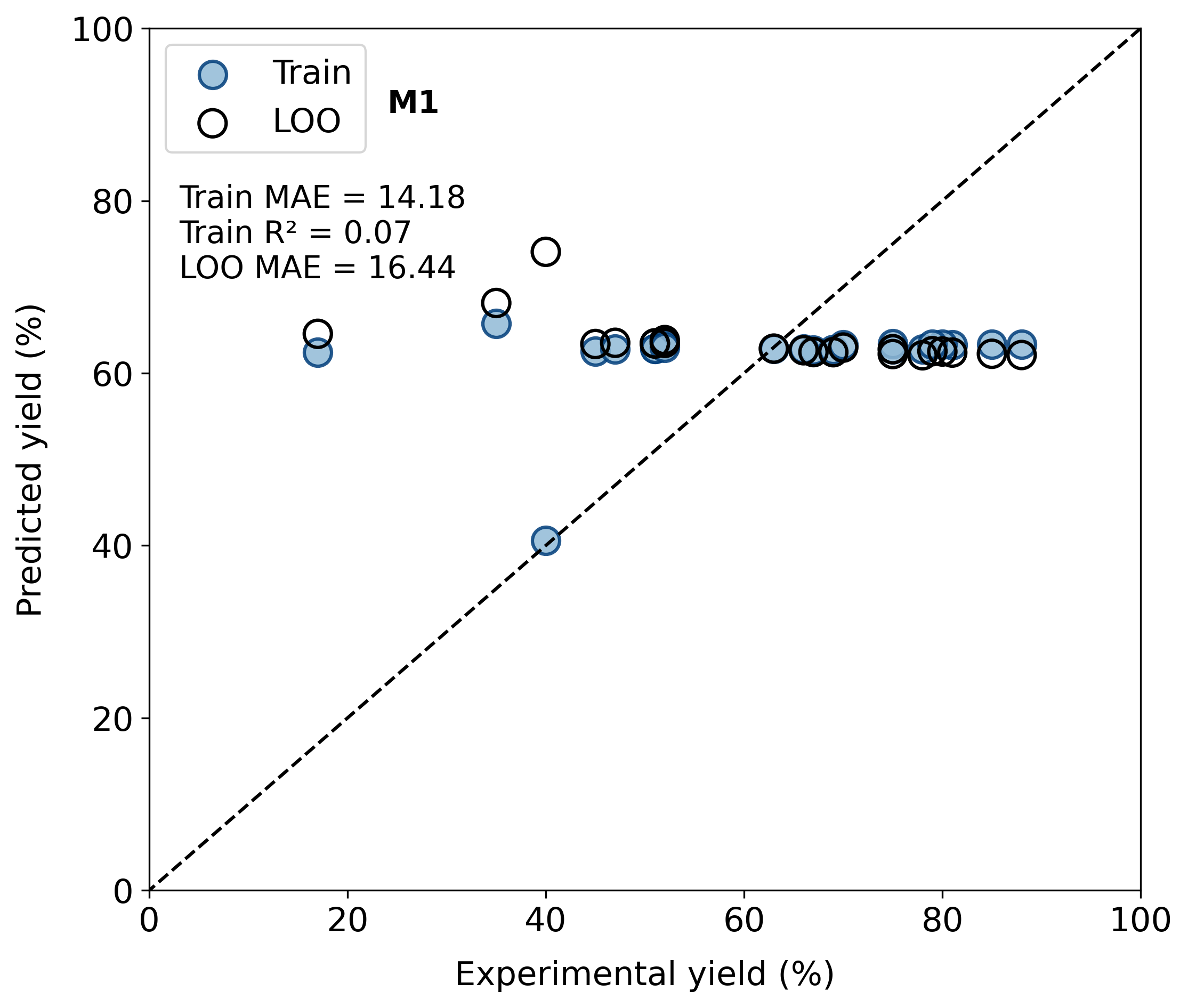

Supplement: SC-017-D5SC08962J-s002 [file SC-017-D5SC08962J-s002.zip › SI_MVLR_Studies/MVLR_Ru_DBT_22samples/M1_model5.png]

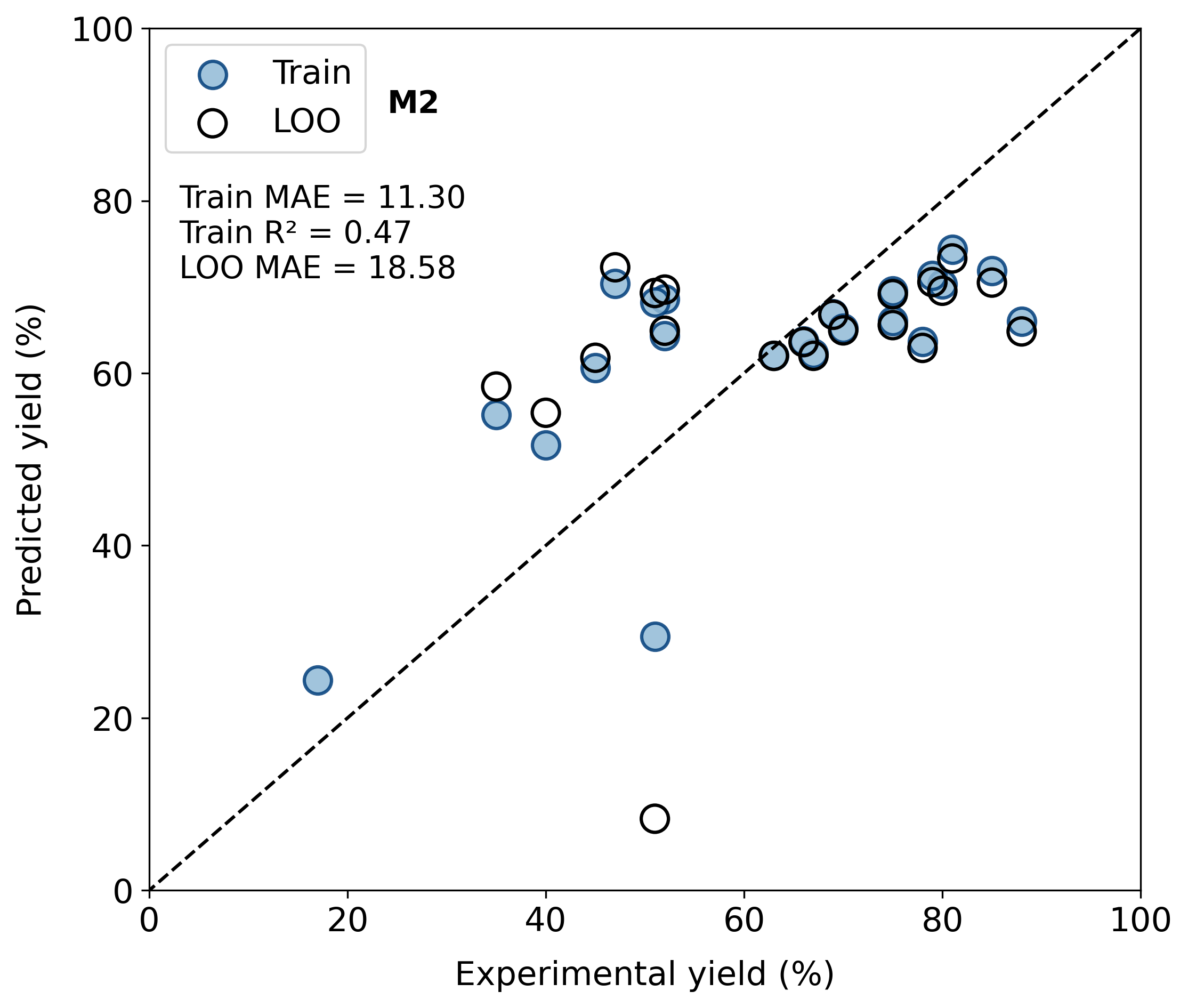

Supplement: SC-017-D5SC08962J-s002 [file SC-017-D5SC08962J-s002.zip › SI_MVLR_Studies/MVLR_Ru_DBT_22samples/M2_model1.png]

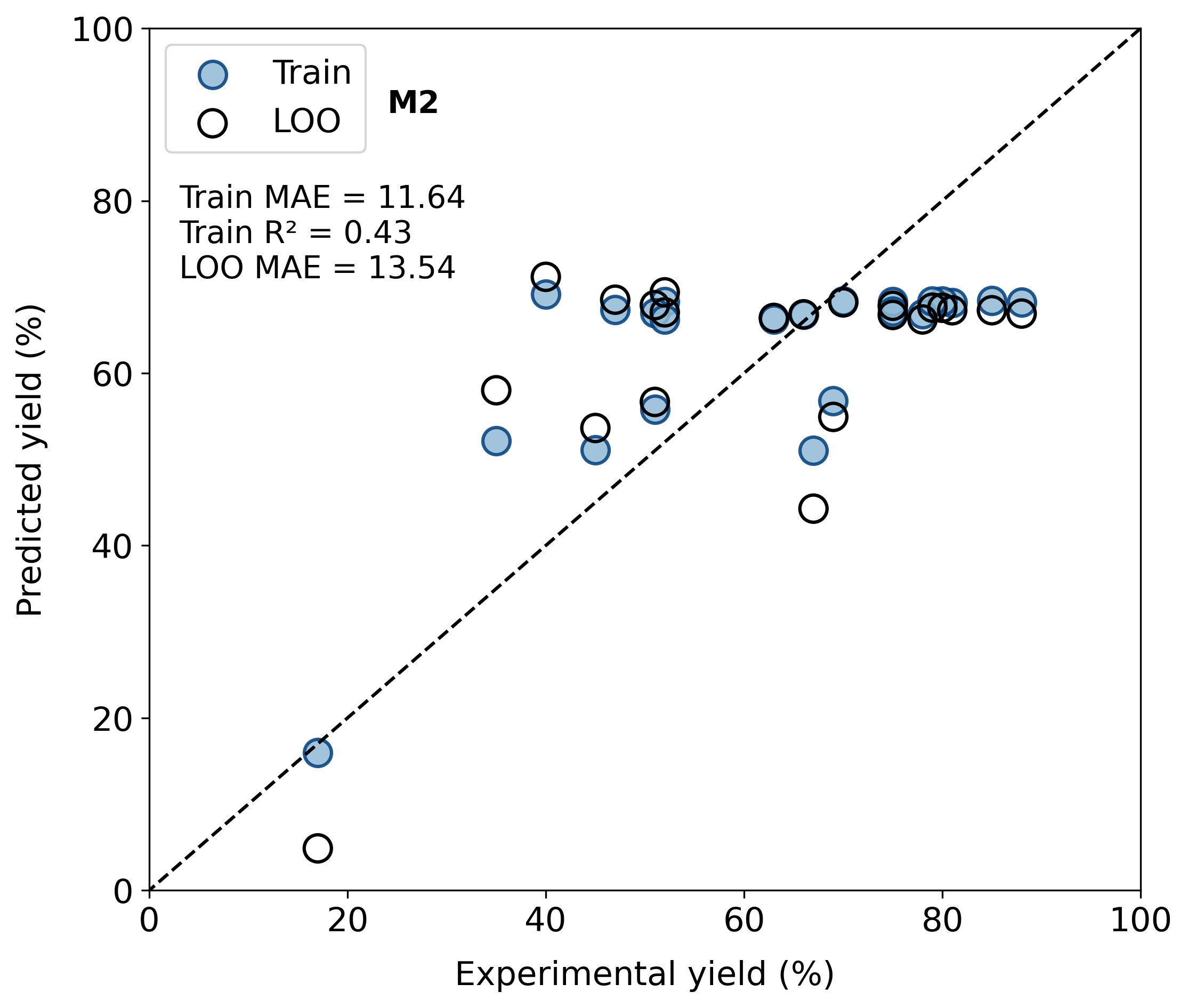

Supplement: SC-017-D5SC08962J-s002 [file SC-017-D5SC08962J-s002.zip › SI_MVLR_Studies/MVLR_Ru_DBT_22samples/M2_model2.png]

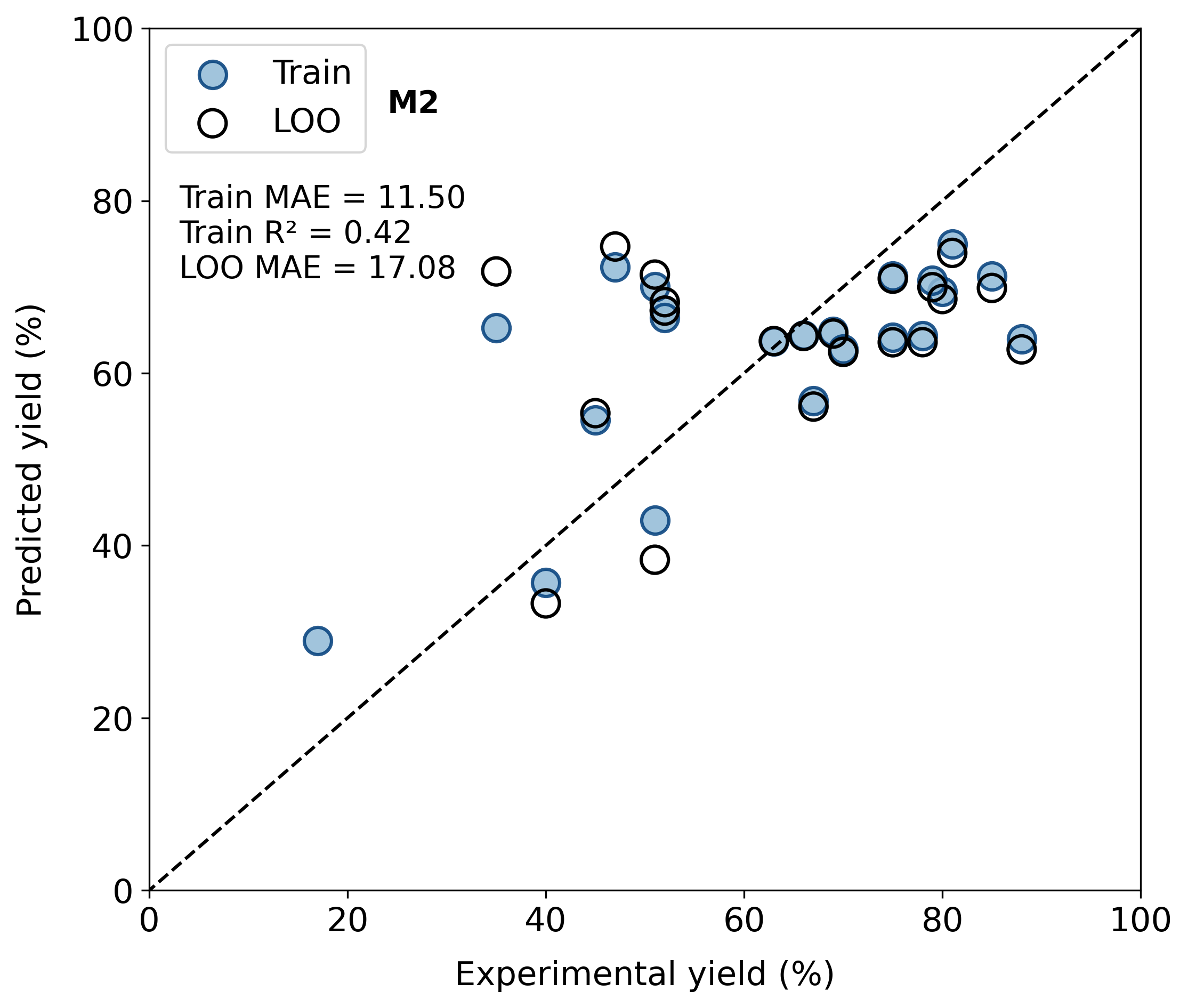

Supplement: SC-017-D5SC08962J-s002 [file SC-017-D5SC08962J-s002.zip › SI_MVLR_Studies/MVLR_Ru_DBT_22samples/M2_model3.png]

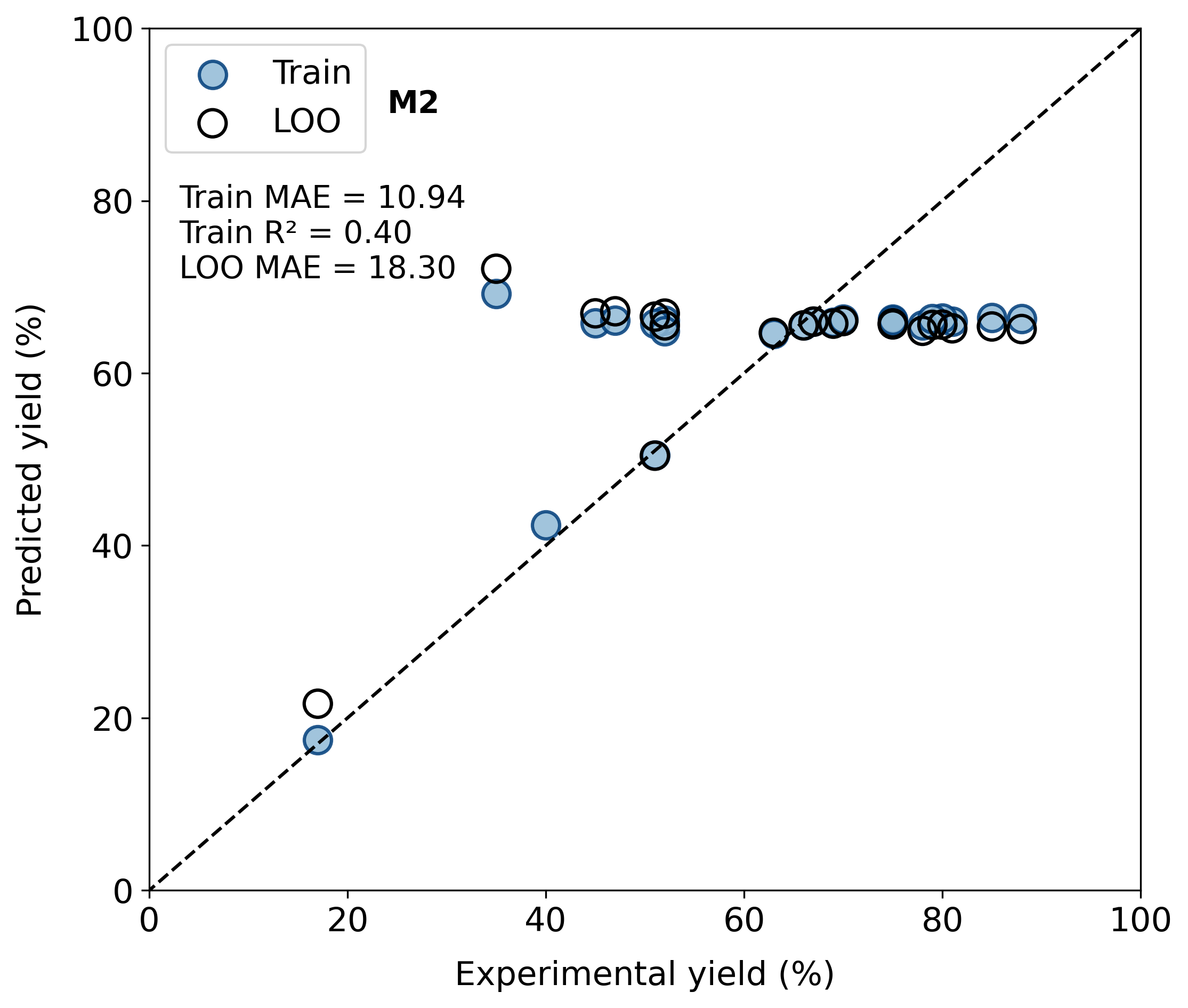

Supplement: SC-017-D5SC08962J-s002 [file SC-017-D5SC08962J-s002.zip › SI_MVLR_Studies/MVLR_Ru_DBT_22samples/M2_model4.png]

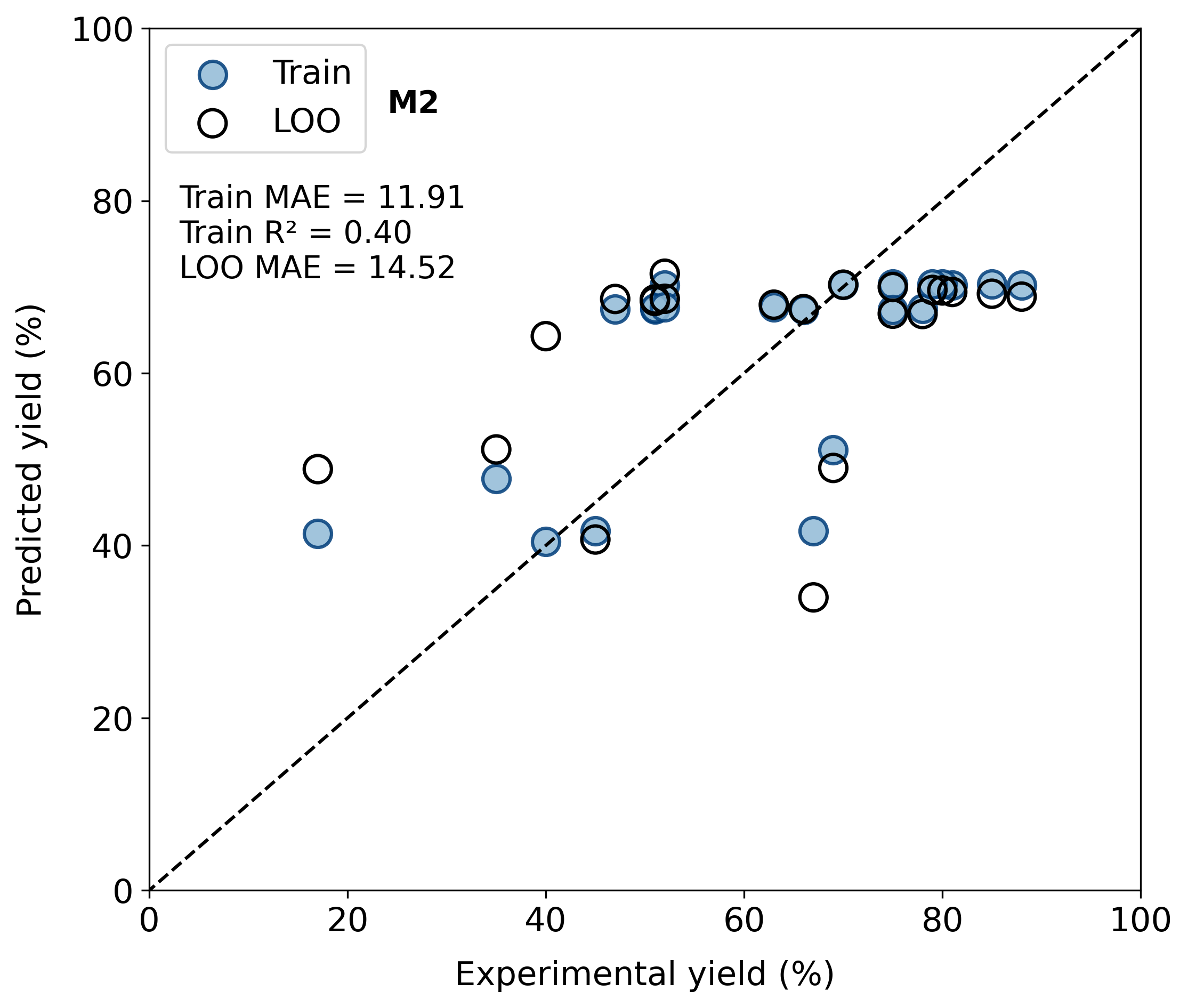

Supplement: SC-017-D5SC08962J-s002 [file SC-017-D5SC08962J-s002.zip › SI_MVLR_Studies/MVLR_Ru_DBT_22samples/M2_model5.png]

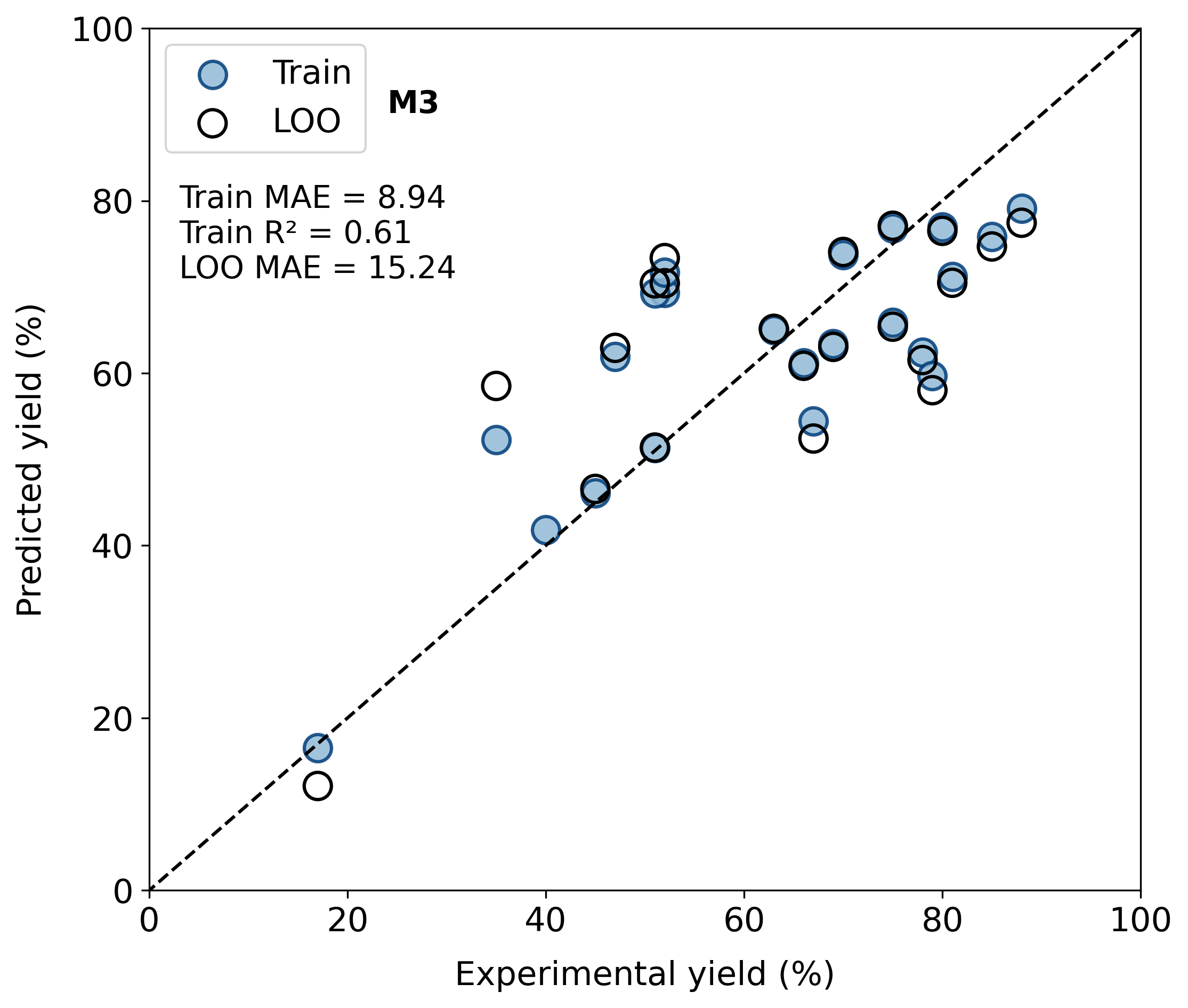

Supplement: SC-017-D5SC08962J-s002 [file SC-017-D5SC08962J-s002.zip › SI_MVLR_Studies/MVLR_Ru_DBT_22samples/M3_model1.png]

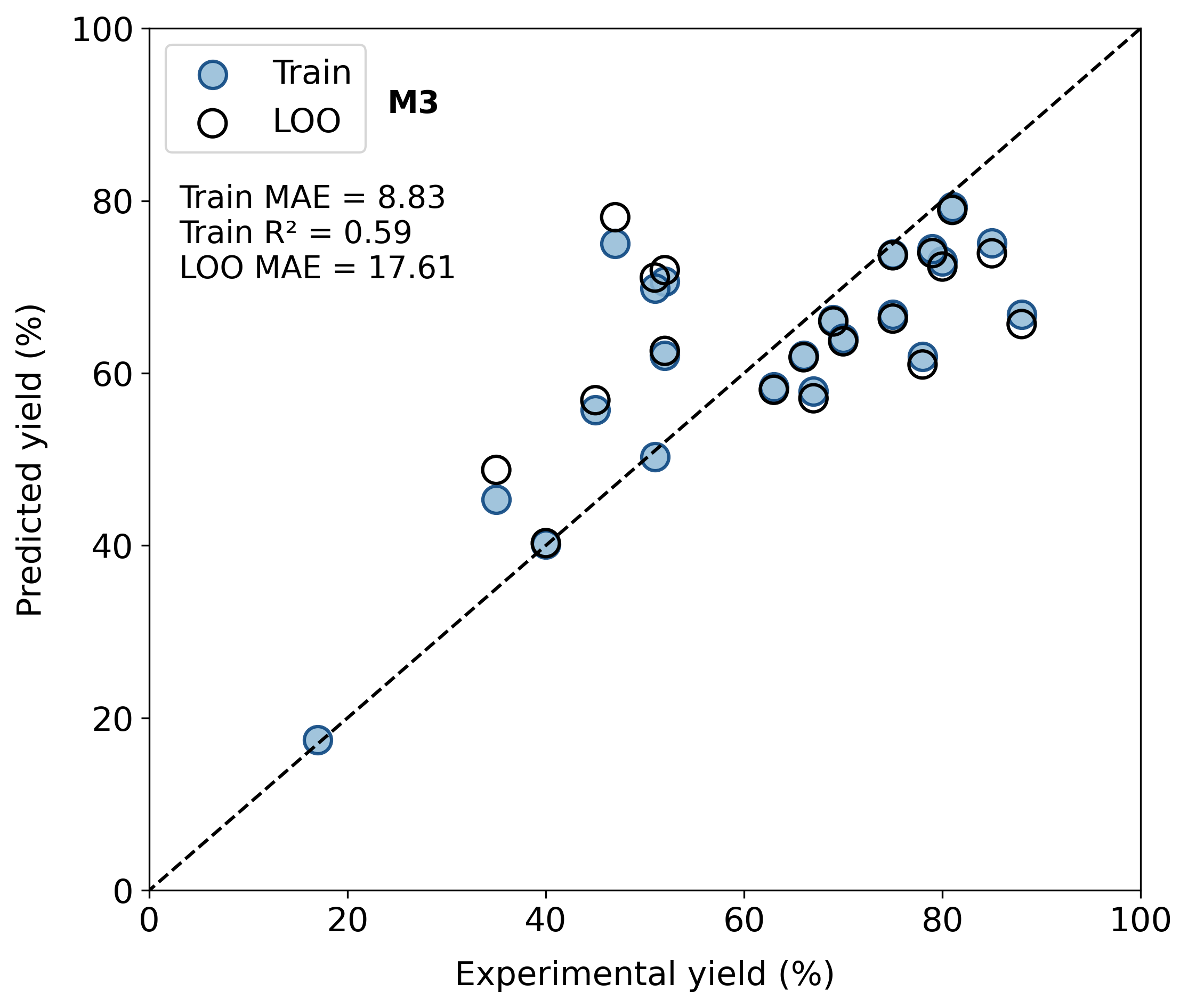

Supplement: SC-017-D5SC08962J-s002 [file SC-017-D5SC08962J-s002.zip › SI_MVLR_Studies/MVLR_Ru_DBT_22samples/M3_model2.png]

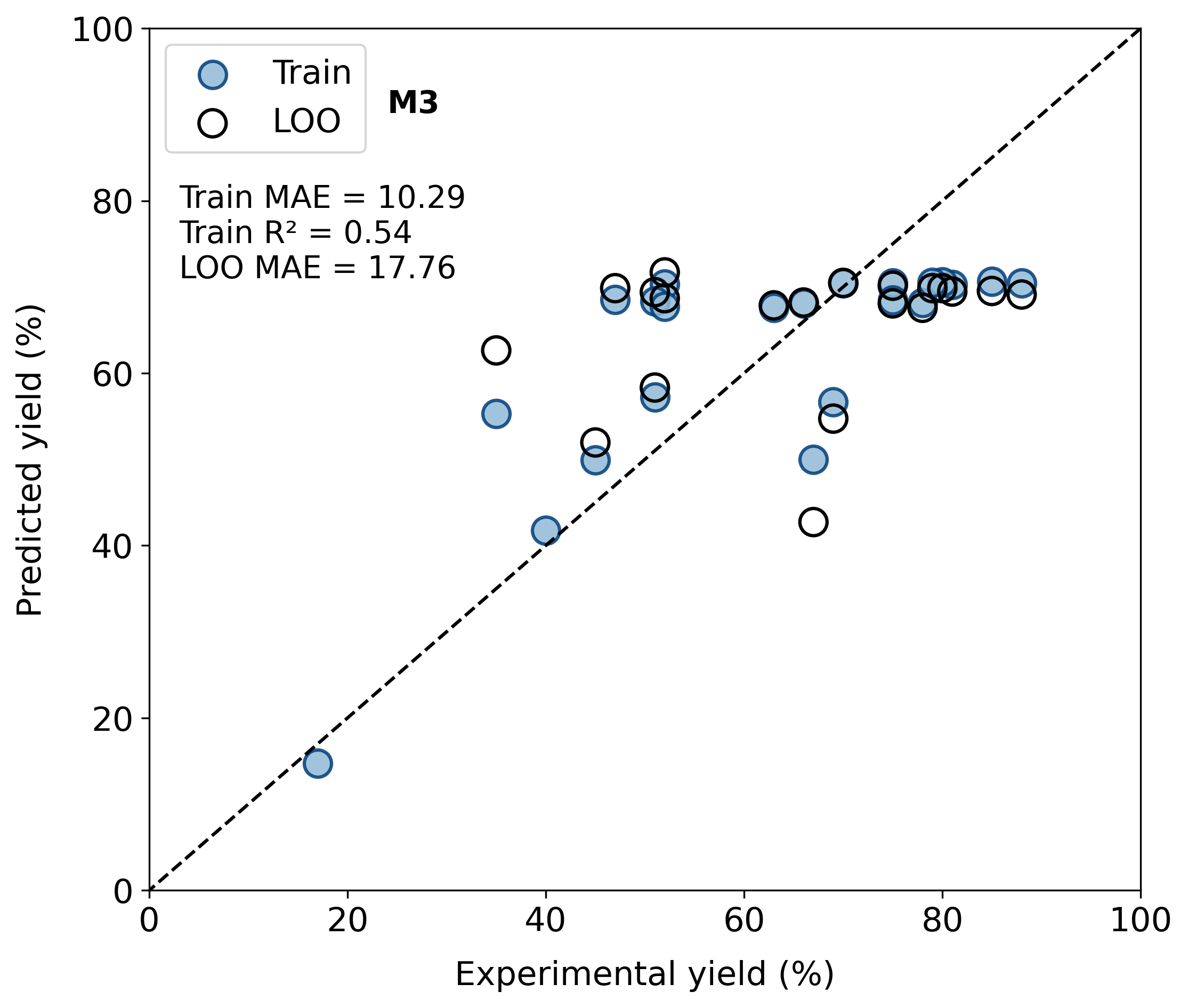

Supplement: SC-017-D5SC08962J-s002 [file SC-017-D5SC08962J-s002.zip › SI_MVLR_Studies/MVLR_Ru_DBT_22samples/M3_model3.png]

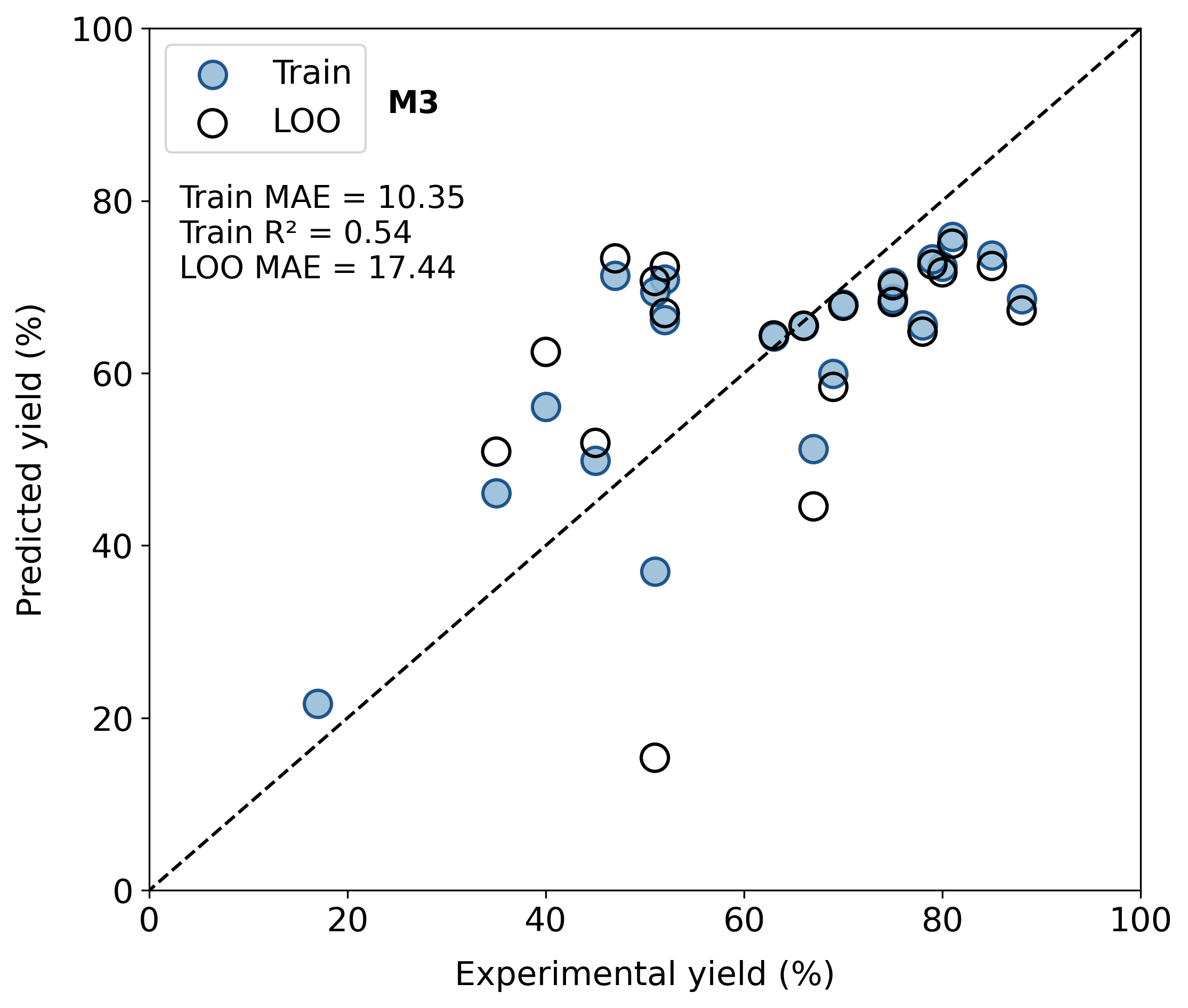

Supplement: SC-017-D5SC08962J-s002 [file SC-017-D5SC08962J-s002.zip › SI_MVLR_Studies/MVLR_Ru_DBT_22samples/M3_model4.png]

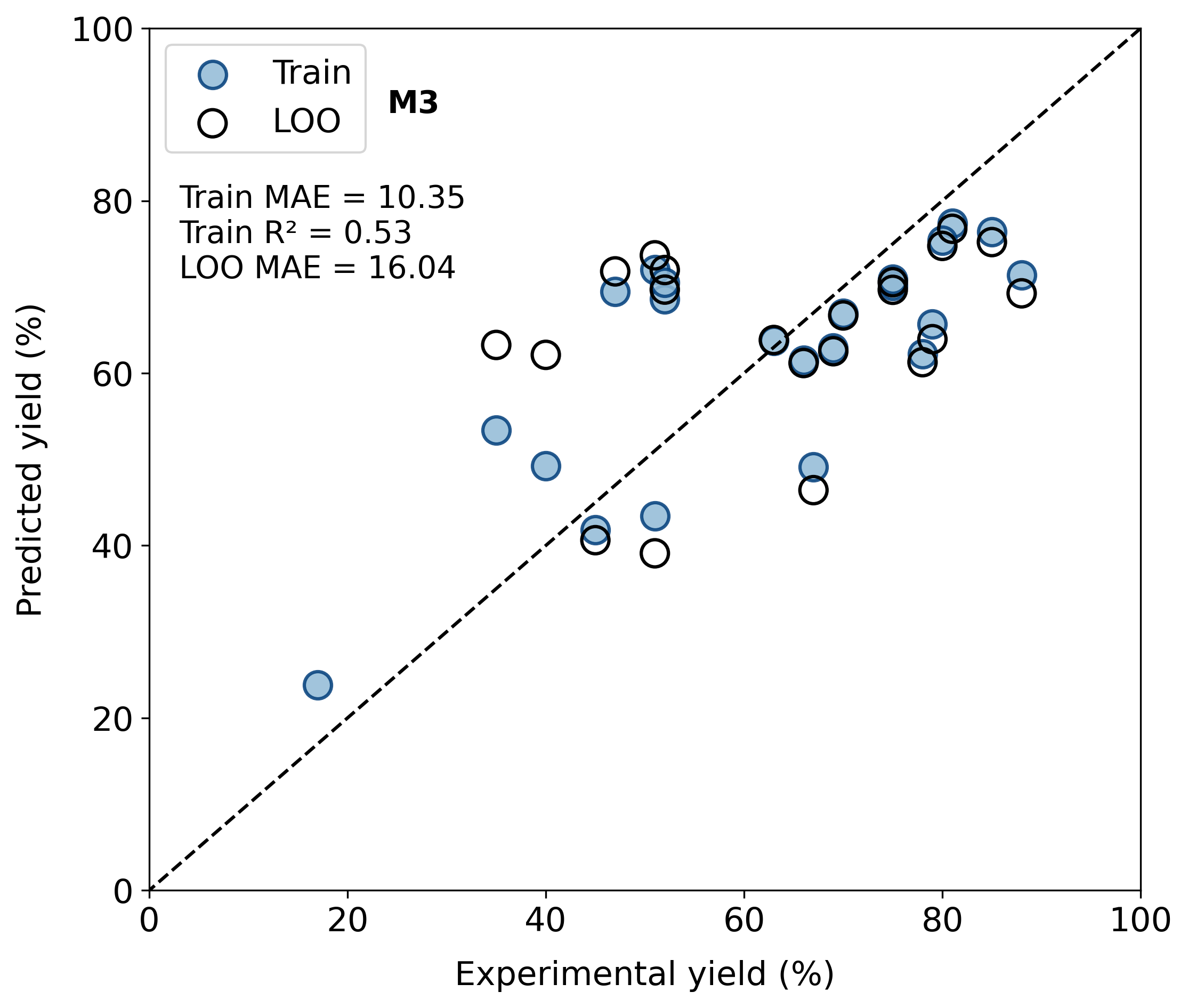

Supplement: SC-017-D5SC08962J-s002 [file SC-017-D5SC08962J-s002.zip › SI_MVLR_Studies/MVLR_Ru_DBT_22samples/M3_model5.png]

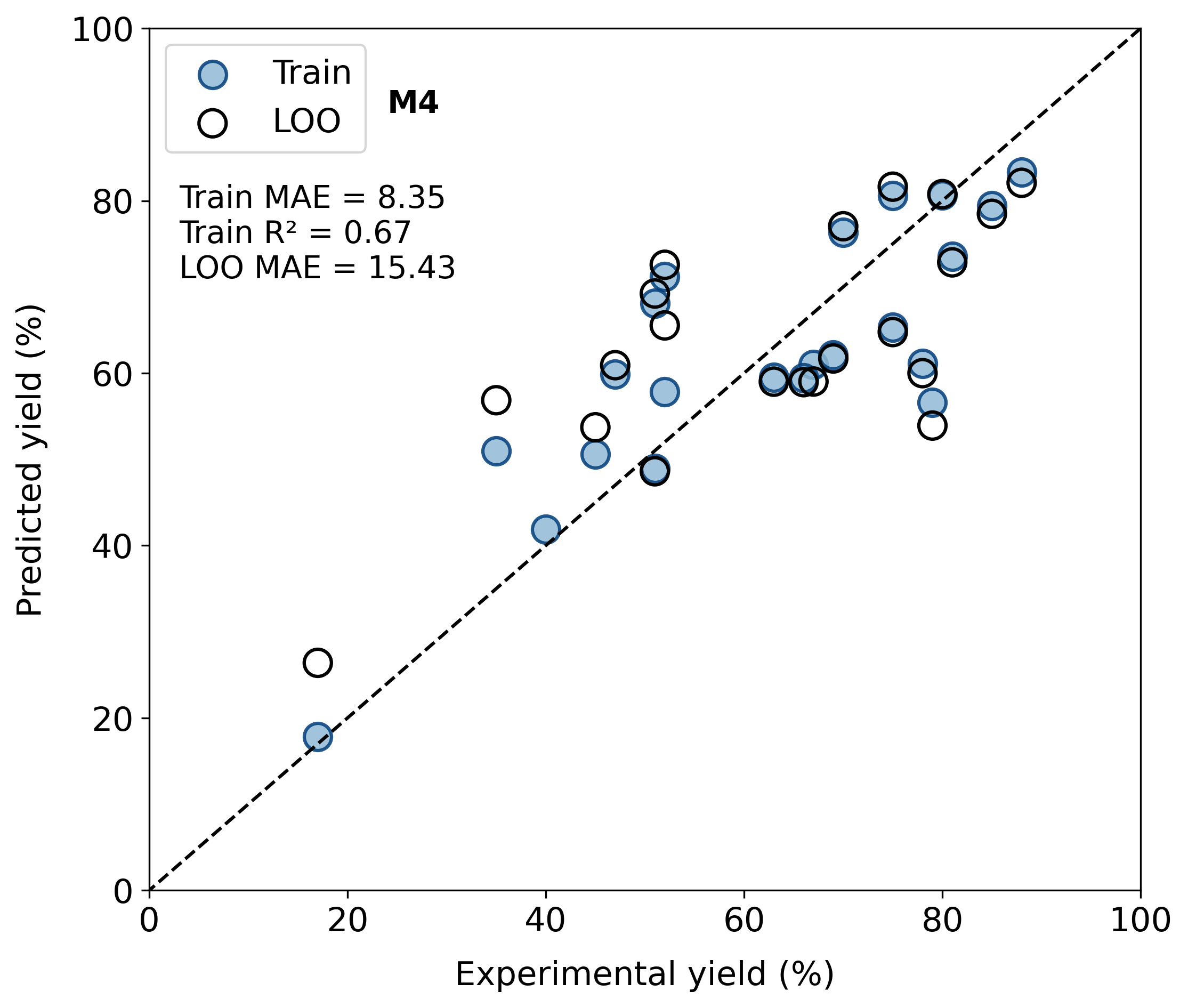

Supplement: SC-017-D5SC08962J-s002 [file SC-017-D5SC08962J-s002.zip › SI_MVLR_Studies/MVLR_Ru_DBT_22samples/M4_model1.png]

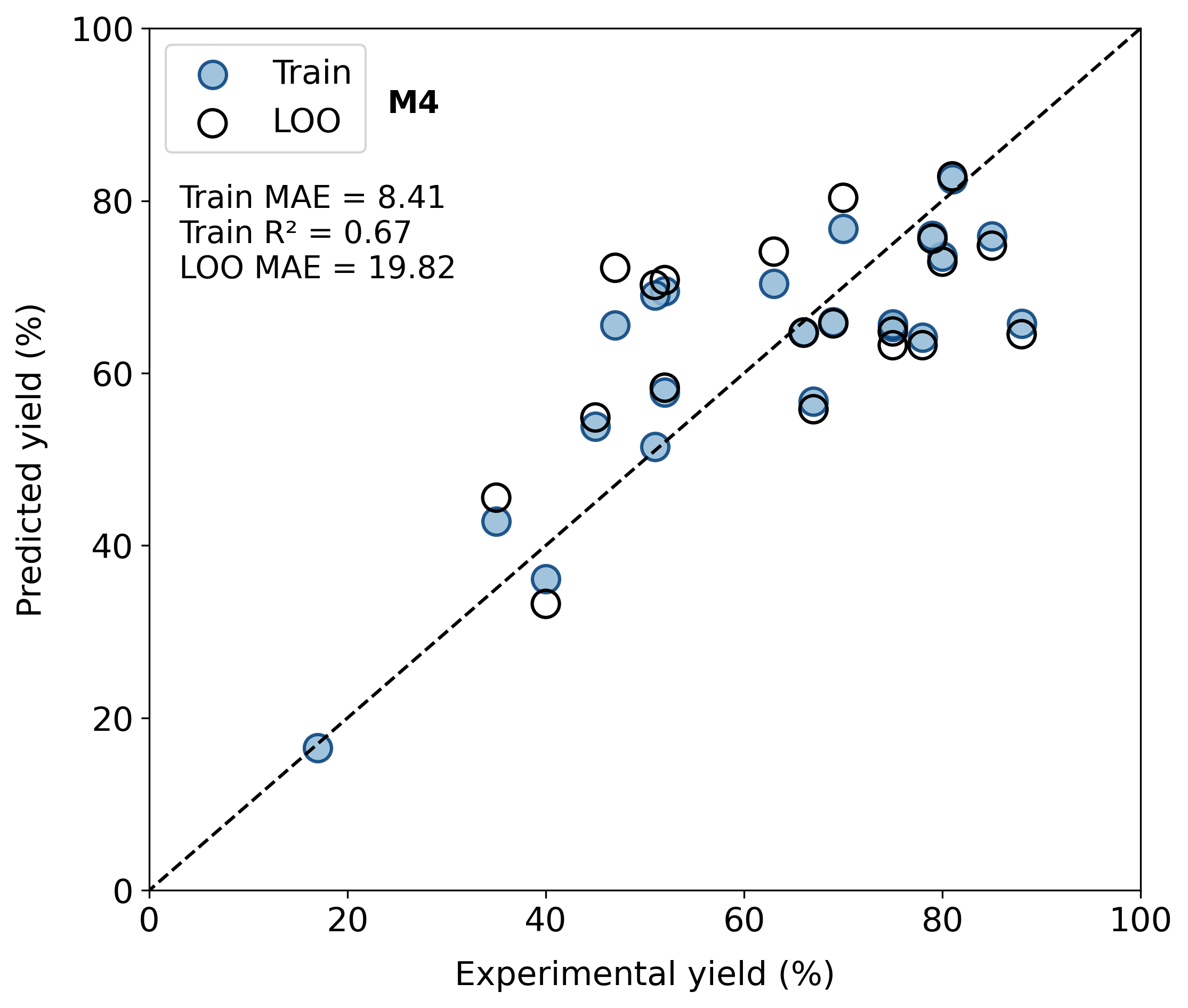

Supplement: SC-017-D5SC08962J-s002 [file SC-017-D5SC08962J-s002.zip › SI_MVLR_Studies/MVLR_Ru_DBT_22samples/M4_model4.png]

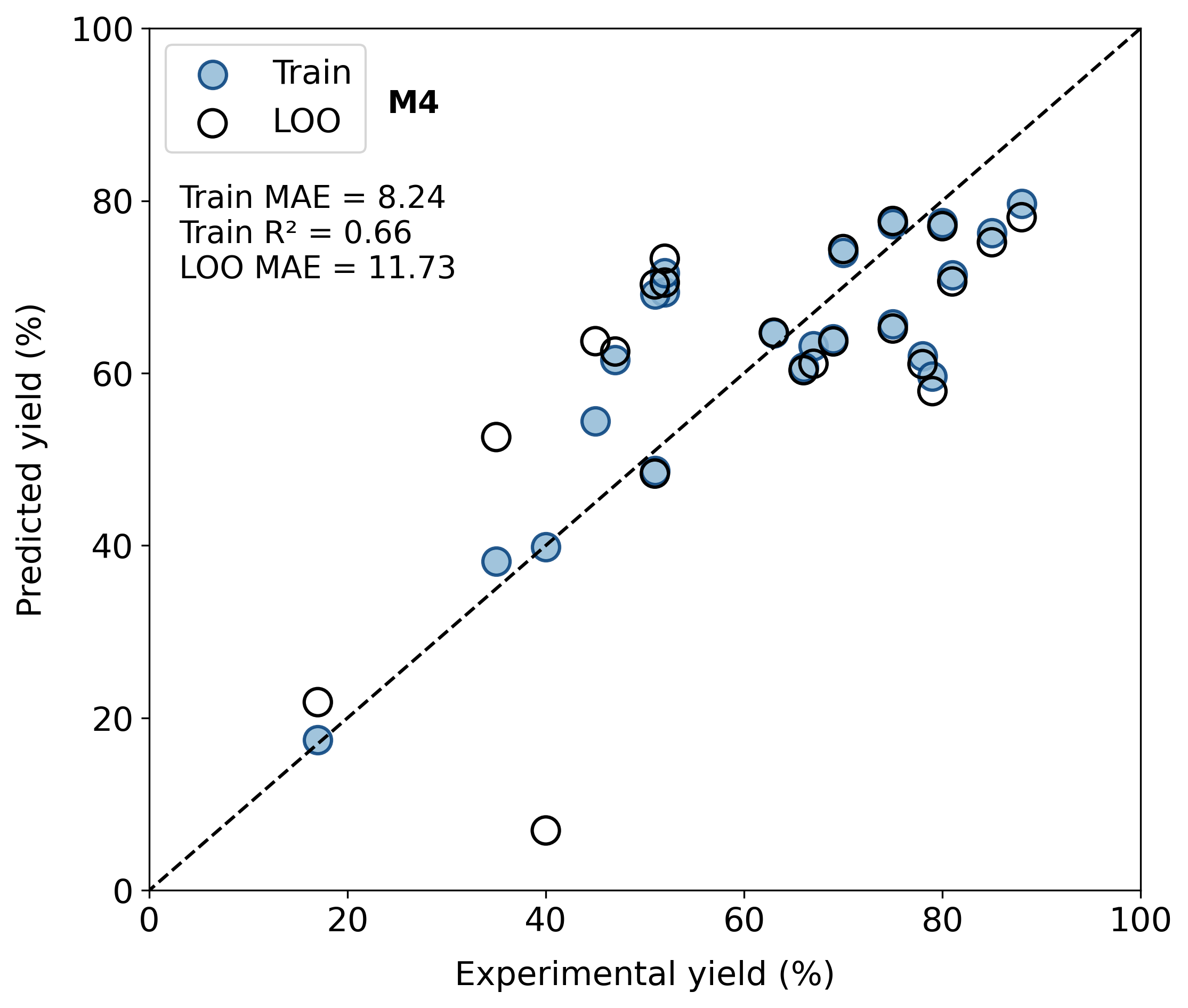

Supplement: SC-017-D5SC08962J-s002 [file SC-017-D5SC08962J-s002.zip › SI_MVLR_Studies/MVLR_Ru_DBT_22samples/M4_model5.png]
